# Protic Ionic Liquid as Reagent, Catalyst, and Solvent: 1‐Methylimidazolium Thiocyanate

**DOI:** 10.1002/anie.202016593

**Published:** 2021-02-26

**Authors:** Ivan A. Andreev, Nina K. Ratmanova, André U. Augustin, Olga A. Ivanova, Irina I. Levina, Victor N. Khrustalev, Daniel B. Werz, Igor V. Trushkov

**Affiliations:** ^1^ Dmitry Rogachev National Medical Research Center of Pediatric Hematology, Oncology and Immunology Samory Mashela 1 117997 Moscow Russian Federation; ^2^ N. D. Zelinsky Institute of Organic Chemistry, Russian Academy of Sciences Leninsky pr. 47 119991 Moscow Russian Federation; ^3^ Technische Universität Braunschweig Institute of Organic Chemistry Hagenring 30 38106 Braunschweig Germany; ^4^ Department of Chemistry Lomonosov Moscow State University Leninskie Gory 1–3 119991 Moscow Russian Federation; ^5^ Institute of Biochemical Physics Russian Academy of Sciences Kosygina 4 119334 Moscow Russian Federation; ^6^ Faculty of Science RUDN University Miklukho-Maklaya 6 117198 Moscow Russian Federation

**Keywords:** (iso)thiocyanates, donor-acceptor cyclopropanes, ionic liquids, nitrogen heterocycles, small ring systems

## Abstract

We propose a new concept of the triple role of protic ionic liquids with nucleophilic anions: a) a regenerable solvent, b) a Brønsted acid inducing diverse transformations via general acid catalysis, and c) a source of a nucleophile. The efficiency of this strategy was demonstrated using thiocyanate‐based protic ionic liquids for the ring‐opening of donor‐acceptor cyclopropanes. A wide variety of activated cyclopropanes were found to react with 1‐methylimidazolium thiocyanate under mild metal‐free conditions via unusual nitrogen attack of the ambident thiocyanate ion on the electrophilic center of the three‐membered ring affording pyrrolidine‐2‐thiones bearing donor and acceptor substituents at the C(5) and C(3) atoms, respectively, in a single time‐efficient step. The ability of 1‐methylimidazolium thiocyanate to serve as a triplex reagent was exemplarily illustrated by (4+2)‐annulation with 1‐acyl‐2‐(2‐hydroxyphenyl)cyclopropane, epoxide ring‐opening and other organic transformations.

## Graphical abstract

A resplendent trio: Protic ionic liquids (PILs) with nucleophilic anions served in concert as regenerable reaction medium, Brønsted acid, and a source of nucleophile. Heating of donor–acceptor cyclopropanes with 1‐methylimidazolium thiocyanate (B:⋅HNCS) afforded products of formal (3+2)‐ and (4+2)‐cycloaddition of isothiocyanic acid.

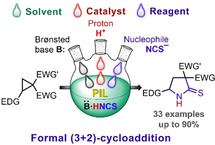

## Introduction

Modern demands of synthetic chemistry require the selection of reaction conditions providing both high yields of the target products due to high chemo‐, regio‐, and stereoselectivity of the processes employed and conformity with the fundamental principles of green chemistry.^[1]^ Among them, the essential ones are concepts such as atom and step economy,^[2]^ design and application of less hazardous reagents and catalysts, as well as minimization of waste production.[^3^, ^4^]

**Scheme 1:**
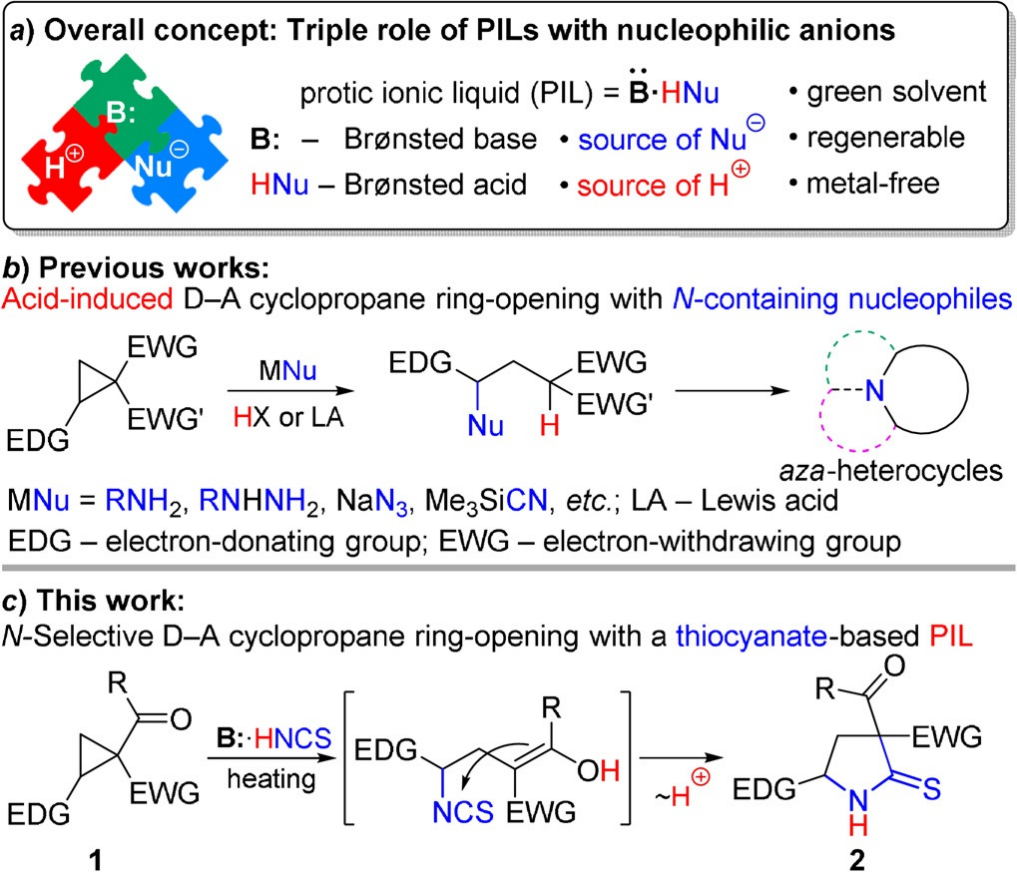
General strategy of using PIL as solvent, reagent, and catalyst and its application for the ring‐opening of D‐A cyclopropanes with thiocyanate ion.

One of the most attractive solutions to all these problems is the use of protic ionic liquids (PILs), i.e., low‐melting salts of Brønsted acid and base.^[5]^ On the one hand, PILs can pursue a threefold role serving cooperatively as: a) an excellent reaction medium, dissolving both hydrophobic and hydrophilic molecules, b) process initiator via acid‐base catalysis, and c) reagent, if the PIL contains highly nucleophilic species, red‐ox agent, etc. On the other hand, PILs can be easily recycled and employed repeatedly, satisfying environmental demands.^[5]^ So much more astonishing, to the best of our knowledge, is the absence of reports on the use of this triple role of PILs in organic synthesis (Scheme 1 a).

To test this new strategy, we selected transformations of donor‐acceptor (D‐A) cyclopropanes^[6]^ due to our long‐standing interest in this rapidly growing area of synthetic chemistry, which gave birth to a plethora of reactions, from quite simple to very unusual ones,^[7]^ and continues to inspire and guide the elaboration of interesting original processes. Thus, we and others have developed ring‐openings of D‐A cyclopropanes with diverse nitrogen‐containing nucleophiles followed by a new ring formation as a powerful tool for the synthesis of *N*‐heterocycles,^[8]^ including pharmacologically important ones (Scheme 1 b). Nevertheless, the reaction of D‐A cyclopropanes with various sources of thiocyanate ion has not been reported yet.

We believe that this reaction can be performed using a thiocyanate‐based PIL as Brønsted acids are known to induce reactions of D‐A cyclopropanes.[^8b^, ^8e^, ^9^] PILs provide a high concentration of both nucleophile and proton, facilitating a transformation. Moreover, the protonation of an intermediate enolate ion in the acidic media allows the avoidance of: a) the process reversibility, proved in the related ring‐opening with azide ion,^[10]^ and b) CH‐acid elimination, detected in the reaction of D‐A cyclopropanes with sodium cyanide.^[11]^

Herein, we describe the synthesis of relevant PILs and their utilization for the chemoselective ring‐opening of D‐A cyclopropanes **1** via nitrogen attack on the three‐membered ring affording pyrrolidine‐2‐thiones **2** bearing two acceptor groups at the C(3) and donor substituent at the C(5) atom in a single time‐efficient step (Scheme 1 c). Such behavior of ambident thiocyanate ion differs crucially from the typical reactivity of this nucleophile with saturated carbon atoms when *S*‐attack proceeds predominantly or exclusively.^[12]^ It should be noted that our previous two‐step transformation of cyclopropanes **1** to pyrrolidine‐2‐thiones **2** required prolonged heating and tremendous combined reaction times, provided much lower yields and involved toxic reagents.^[13]^

## Results and Discussion

We started this work by searching for optimal reaction conditions. To be a stable reaction medium, PIL should have a full proton transfer from an acid to a base;^[5]^ full ionicity is also desired for the highest nucleophilicity of the thiocyanate ion in the PIL. This is best achieved when the difference of the p*K*_a_ values (Δp*K*_a_) of the two PIL‐forming components is larger than 8.^[14]^ For thiocyanate‐derived PILs, isothiocyanic acid (HNCS) should be considered as an acidic component; its p*K*_a_ was determined to be −1.28,^[15]^ therefore, BH^+^ should have a p*K*_a_>6.7. Nevertheless, the PIL should be acidic enough to induce a ring‐opening of D‐A cyclopropanes, i.e., p*K*_a_ (BH^+^) value should be as low as possible. Alkyl‐, dialkyl‐, and trialkylammonium thiocyanates, which were typically studied as PILs in electrochemistry, chromatography, etc.,[^5^, ^16^] have p*K*_a_ (BH^+^) values between 9.5 and 11;^[17]^ these PILs are expected to have low activity in initiating reactions of D‐A cyclopropanes. To check these assumptions, we decided to investigate PILs with three different basic components: 1‐methylimidazole (Mim, p*K*_a_ (BH^+^) 7.1^[18]^) as a base of choice; triethylamine as non‐nucleophilic species with basicity, typical for alkylamines (p*K*_a_ (BH^+^) 10.7);^[19]^ and *N*,*N*,*N*′,*N*′‐tetramethylguanidine (Tmg) as an amine with increased basicity (p*K*_a_ (BH^+^) 13.6).^[19]^

**Scheme 2:**
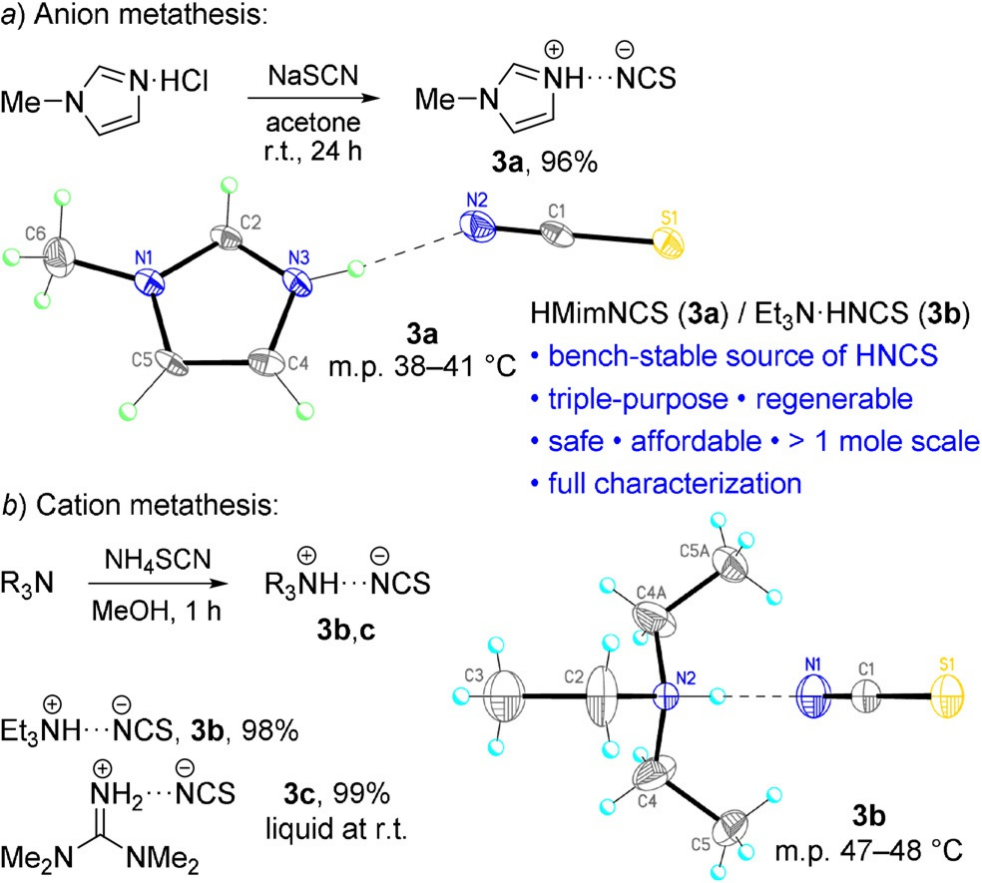
Syntheses of PILs **3** and single‐crystal X‐ray structures of HMimNCS (**3 a**) and Et_3_N⋅HNCS (**3 b**); thermal ellipsoids are shown at 50 % ellipsoid probability.

To obtain salts formed by (iso)thiocyanic acid with amines, two main approaches are usually applied: anion metathesis by a reaction of the corresponding ammonium halides with metal thiocyanate[^16b^, ^20^] and cation metathesis by the displacement of ammonia in NH_4_SCN with more basic amines.^[21]^ For the preparation of 1‐methylimidazolium thiocyanate (HMimNCS, **3 a**), we applied the first method, using the modified procedure for the reaction of commercial HMimCl with sodium thiocyanate.^[22]^ On the contrary to the original report, wherein the product was poorly purified and almost uncharacterized, we obtained HMimNCS on a molar scale as an analytically pure bench‐stable compound and proved its structure by single‐crystal X‐ray analysis (Scheme 2 a).[^23^, ^24^]

Two other thiocyanate‐based PILs Et_3_N⋅HNCS (**3 b**)^[21b]^ and HTmgNCS (**3 c**) were synthesized by the reaction of ammonium thiocyanate with the corresponding amines; triethylammonium thiocyanate was characterized by single‐crystal X‐ray analysis (Scheme 2 b). We found that the completeness of the cation metathesis can be controlled by an “ammonium test”: if the dissolution of the obtained PIL (ca. 150 mg) in CDCl_3_ (ca. 550 μL) during the preparation of an NMR sample was not accompanied by the appearance of a visible suspension of NH_4_SCN, the PIL contained no residual ammonium ion.

Using the obtained PILs, we optimized reaction conditions for the transformation of D‐A cyclopropane **1 a** as a model substrate. Under heating in HMimNCS, **1 a** was found to produce 5‐aryl‐2‐thioxopyrrolidine‐3,3‐diester **2 a** in good yield. The best result (81 %) was achieved when cyclopropane **1 a** was heated in HMimNCS (**3 a**) at 70 °C for 1 h (Table 1, entry 1). Product **2 a** was obtained with the same yield when the reaction was performed at 50 °C for 2 h (Table 1, entry 2). Oppositely, the increase of the reaction temperature afforded **2 a** contaminated with inseparable side products (Table 1, entries 3–5).

**Table 1:** Optimization of reaction conditions for the transformation of **1 a** to **2 a**.^[a]^ 
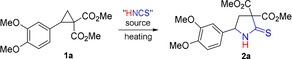

Entry	“HNCS” source	*T* [°C]	*t* [h]	Yield of **2 a** ^[b]^
1	HMimNCS	70	1	81^[c]^
2	HMimNCS	50	2	81^[c]^
3	HMimNCS	80	2	79^[d]^
4	HMimNCS	100	1.5	65^[c,d]^
5	HMimNCS	150	1	–^[e]^
6	HMimNCS (2 M solution of **1 a**)	70	1	66^[e]^
7	Et_3_N⋅HNCS	70	13.5	58^[f]^
8	HTmgNCS	70	1	–^[g]^
9	HTmgNCS	100	4	–^[e]^
10	NaSCN (2 equiv), Et_3_N⋅HCl (2 equiv), DMF	100	4	62^[h]^
11	NaSCN (2 equiv), Et_3_N⋅HCl (2 equiv), DMF	100	17	58^[c]^
12	NaSCN (2 equiv), HMimCl (2 equiv), DMF	70	6	56^[h]^
13	NaSCN (2 equiv), Et_3_N⋅3AcOH	100	4	–^[e]^
14	NaSCN (2 equiv), Et_3_N⋅TFA	100	5.5	–^[e]^

[a] 1 M solution of **1 a** in PIL; 0.5 M solution in DMF. [b] NMR yield in [%]. [c] Isolated yield. [d] Contaminated by 3–7 % of inseparable side products. [e] Complex mixture. [f] 59 % conversion. [g] No conversion. [h] ca. 70 % conversion.

The above optimizations were performed at 1 M concentration of **1 a** in HMimNCS (ca. 8 equiv), which ensured the homogeneity of reaction mixtures. The attempt to decrease PIL quantity resulted in a diminished yield of **2 a** (Table 1, entry 6). This outcome can be explained by the fact that the excess of **3 a** not only facilitates the reaction but also allows suppression of side‐processes as D‐A cyclopropanes possess numerous modes of reactivity, including rearrangements and dimerizations. Thus, the optimal excess of HMimNCS corresponded to 1 M solution of D‐A cyclopropane **1 a** in PIL.

As for other less acidic PILs, at 70 °C, the conversion of **1 a** was moderate in triethylammonium thiocyanate (**3 b**) and very low, if at all, in HTmgNCS (**3 c**) (Table 1, entries 7, 8); the increase of reaction temperature led to decomposition only (Table 1, entry 9).

We also tested reaction conditions, similar to those employed for the ring‐opening of D‐A cyclopropanes with sodium azide.^[10]^ However, heating of **1 a** with sodium thiocyanate and Et_3_N⋅HCl as a proton source in *N*,*N*‐dimethylformamide (DMF) produced crude **2 a** in moderate yield and conversion even after 4 h at 100 °C (Table 1, entry 10). A tremendous increase in the reaction time allowed us to achieve almost complete conversion of the starting material (Table 1, entry 11). However, the yield of **2 a** was even lower than that after 4 h. The attempt to improve the above results by switching from Et_3_N⋅HCl to HMimCl was unsuccessful (Table 1, entry 12). Thus, the conventional reaction conditions were rendered unsuitable for the title transformation. Finally, the reaction of **1 a** with a combination of NaSCN and PILs containing no nucleophilic anion (Table 1, entries 13, 14) produced a complex mixture of non‐identified products.

**Scheme 3:**
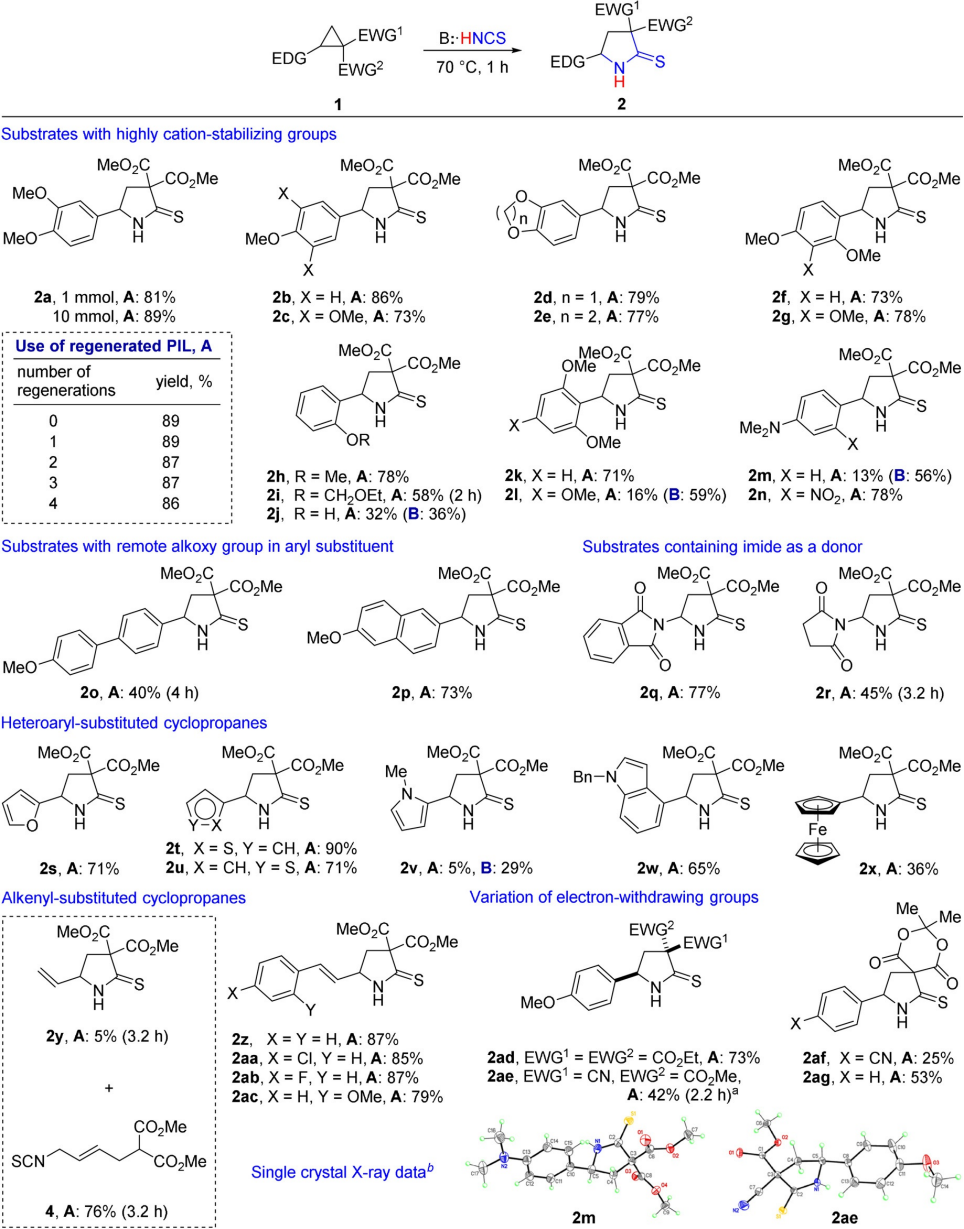
Scope of the formal (3+2)‐cycloaddition of isothiocyanic acid with D‐A cyclopropanes **1** affording pyrrolidine‐2‐thiones **2**. Reaction conditions: **Method A**: 1 M Solution of **1** in HMimNCS, 70 °C, 1 h if not otherwise specified. **Method B**: **1** was added in small portions for 40–45 min to Et_3_N⋅HNCS preheated to 70 °C, then stirred at the same temperature for 15–20 min (total reaction time −1 h). All yields are given for analytically pure compounds. ^*a*^Crude second diastereomer was also obtained in 27 % yield. ^*b*^Thermal ellipsoids are shown at 50 % probability.

With the optimized conditions in hand (method **A**), we investigated the scope of this formal (3+2)‐cycloaddition of D‐A cyclopropanes to the C=N bond of unstable isothiocyanic acid. We found that a broad range of cyclopropane‐1,1‐dicarboxylates bearing electron‐releasing groups at the C(2) atom of the three‐membered ring efficiently participate in the disclosed reaction (Scheme 3).

Good yields of **2** were obtained in reactions of D‐A cyclopropanes containing mono‐, di‐ and trialkoxyphenyl groups (**1 a**–**h,k**). The lower yields of pyrrolidine‐2‐thiones **2 i,j** can be explained by the partial hydrolysis of the acetal moiety during the reaction followed by side processes involving the formed phenolic group.^[25]^

D‐A cyclopropanes **1 l** and **1 m** afforded the target products **2 l** and **2 m**, respectively, in low yields (16 % and 13 %). These substrates are too reactive and undergo a significant decomposition under the reaction conditions. However, in a less acidic PIL Et_3_N⋅HNCS, yields of pyrrolidines **2 l** and **2 m** ^[23]^ were much better (59 % and 56 %), especially when substrates were slowly added to preheated PIL (method **B**), that allowed minimization of side product formation. Oppositely, cyclopropane **1 n**, bearing the 4‐dimethylamino‐2‐nitrophenyl group, produced pyrrolidine **2 n** in good yield.

When the electron‐releasing effect of methoxy group is attenuated by the involvement of an additional aromatic ring between donor group and three‐membered ring (**1 o**), the yield of **2 o** dropped to 40 %. Oppositely, the efficiency of the transformation of 6‐methoxy‐2‐naphthyl‐substituted cyclopropane **1 p** did not principally differ from that of substrates **1 c**–**1 h** and **1 k**. A reasonably good yield of the target product was also obtained in the reaction of phthalimido derivative **1 q**, but the corresponding aminal **2 r** bearing a succinimidyl group was formed in moderate yield (45 %). These results are well consistent with a much higher reactivity of **1 q** vs. **1 r** in the Lewis acid‐catalyzed (3+2)‐cycloaddition with aldehydes.^[26]^

Heteroaryl‐substituted cyclopropanes **1 s**–**1 u** containing 2‐furyl, 2‐ and 3‐thienyl groups as donor as well as (1‐benzylindol‐4‐yl)‐derived cyclopropane **1 w** were found to participate in the discussed transformation providing the target heterocycles in reasonable to excellent yields. On the contrary, (1‐methylpyrrol‐2‐yl)‐substituted D‐A cyclopropane **1 v** was found to be too reactive, producing a significant quantity of admixtures. Similarly to the behavior of **1 l** and **1 m**, the replacement of HMimNCS by the less acidic triethylammonium thiocyanate allowed for increasing yield of the target product, however, to 29 % only. 2‐Vinylcyclopropane‐1,1‐diester **1 y** reacted with HMimNCS via both S_*N*_2‐like and S_*N*_2′‐like processes, affording the product of *N*‐attack on the terminal CH_2_ group **4** in 76 % yield together with a small quantity (5 %) of 5‐vinylpyrrolidine‐2‐thione **2 y**. Oppositely, a series of styryl‐substituted cyclopropanes **1 z**–**1 ac** gave rise to the target pyrrolidinethiones **2 z**–**2 ac** in good yields of 79–87 %.

Unfortunately, dimethyl cyclopropane‐1,1‐dicarboxylates bearing aryl groups, which stabilize the cationic center only in a moderate manner, such as *para*‐methyl‐ or *para*‐fluorophenyl, produced a complex mixture of products under heating in both HMimNCS and Et_3_N⋅HNCS.^[24]^ A similar difference in the reactivity of diversely substituted D‐A cyclopropanes was earlier pointed out in some other transformations.[^11^, ^26^, ^27^] This problem was usually solved by a proper selection of Lewis or Brønsted acid for the process initiation. This work is currently in progress.

The effect of electron‐withdrawing groups on the reaction efficiency is more ambiguous. The substitution of methoxycarbonyl functionalities by ethoxycarbonyl ones influenced insignificantly the reaction efficiency providing **2 ad** in 73 % yield. Cyanoester **1 ae** produced two diastereomeric products in a similar total yield. The structure of the major diastereomer was unambiguously proved by single‐crystal X‐ray analysis.^[23]^ On the other hand, Meldrum's acid‐derived D‐A cyclopropanes **1 af,ag** were transformed into pyrrolidines **2 af,ag** despite the presence of the phenyl and even *para*‐cyano‐phenyl group as the aryl substituent decelerating cyclopropane reactivity significantly.

Therefore, reactive D‐A cyclopropanes **1** can be efficiently transformed into **2** by heating in 1‐methylimidazolium thiocyanate, which serves as a synthetic equivalent of isothiocyanic acid, at 70 °C. If substrates were extremely active (**1 l**, **1 m**, **1 v**, etc.), the target products were obtained with low yields as various side reactions proceeded. We showed that tuning of PIL allows for solving this problem. In the less acidic triethylammonium thiocyanate, products **1 l** and **1 m** were obtained in reasonable yields. A variety of acceptor substituents in cyclopropanes **1** are tolerated by the reaction conditions. Moreover, we showed that D‐A cyclopropanes **1** bearing donor groups with lower cation‐stabilizing ability could be introduced into this formal (3+2)‐cycloaddition if more efficient electron acceptors were present in the molecule. Namely, Meldrum's acid‐derived cyclopropanes **1 af,ag** underwent a transformation into pyrrolidines **2 af,ag**, which can be easily converted into the corresponding methyl diesters.^[28]^

The developed procedure can be scaled up without the loss of efficiency and sustainability.[^24^, ^29^] Thus, when 10 mmol (3 g) of **1 a** were introduced into the reaction, product **2 a** was isolated in 89 % yield. Moreover, PILs **3 a** and **3 b** can be efficiently recovered. In particular, the outcome of **2 a** did not change after 4 cycles of regeneration, the yield of regenerated **3 a** being close to quantitative.[^24^, ^29^]

**Scheme 4:**
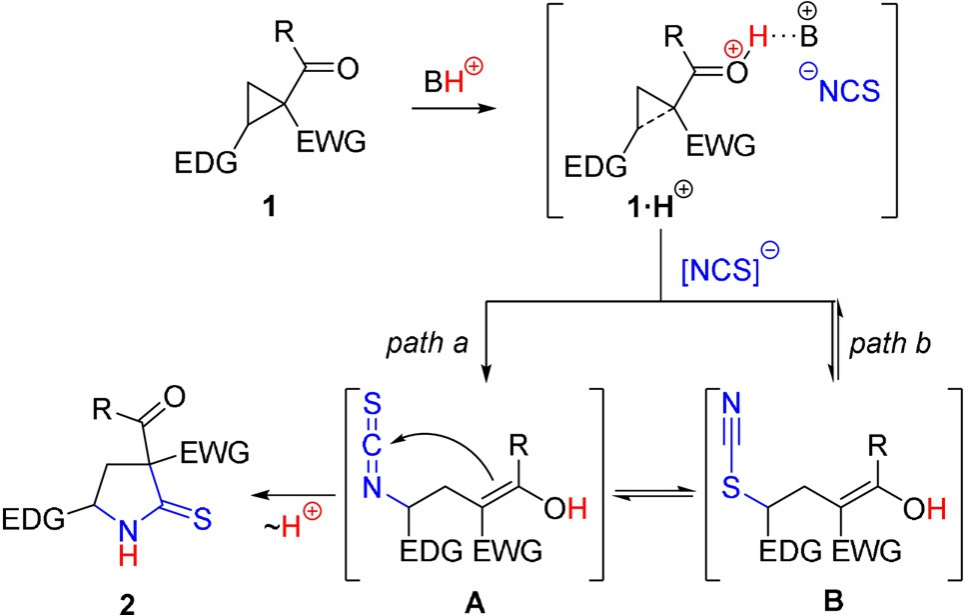
Proposed mechanistic pathways for the formation of pyrrolidines **2**.

Pyrrolidine‐2‐thiones **2** are products of the formal (3+2)‐cycloaddition of HNCS with D‐A cyclopropanes **1**. However, (3+2)‐cycloadditions of D‐A cyclopropanes with diverse alkyl and aryl isothiocyanates afforded 2‐iminotetrahydrothiophenes, but not pyrrolidine‐2‐thiones.[^27g^, ^30^] Both these literature data and our results demonstrate that products **2** are formed by a stepwise mechanism, including protonation of an acceptor group in the D‐A cyclopropane followed by an S_*N*_2‐like attack on the protonated substrate **1⋅H^+^** with thiocyanate ion leading to intermediate **A**. The intramolecular attack of the emerging enol functionality on the isothiocyanate moiety and proton shift accomplished the pyrrolidine‐2‐thione formation (Scheme 4, path *a*).

Based on multiple reports that *S*‐attack of thiocyanate is kinetically preferred in both S_*N*_2 and S_*N*_1 reactions,[^12^, ^31^] the alternative mechanism including an initial formation of thiocyanate **B** followed by its isomerization to **A** could also be supposed (Scheme 4, path *b*). The cyclization of intermediate **A** to the target pyrrolidine‐2‐thione **2** ^[13]^ is a key step for both possible mechanisms. Our failure to identify thiocyanates **B** in reaction mixtures supports path *a*, but cannot be considered as an argument against the alternative route.

**Scheme 5:**
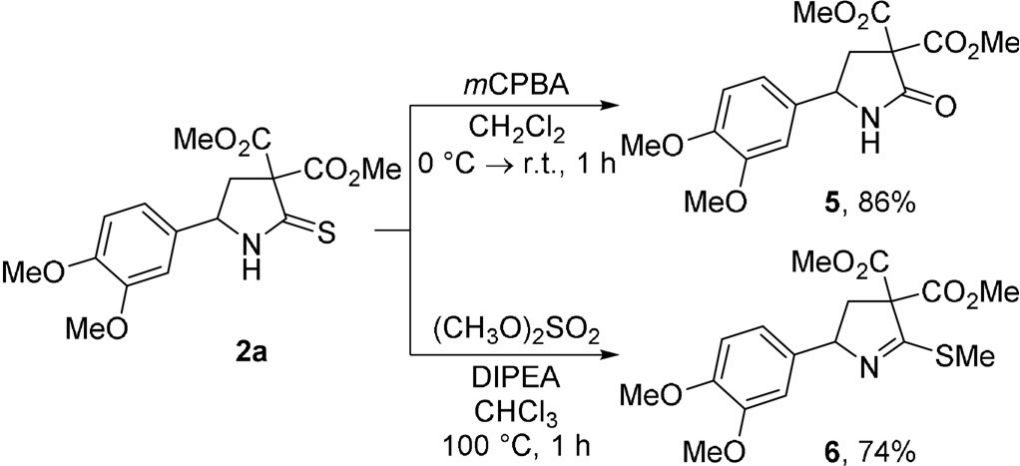
Follow‐up chemistry. DIPEA: *N*,*N*‐diisopropylethylamine.

Additionally, we showed that the obtained pyrrolidine‐2‐thiones **2** could be easily modified to prepare other important products. In particular, we found that the oxidation of **2 a** with *meta*‐chloroperbenzoic acid (*m*CPBA) led to the corresponding pyrrolidone **5**, which can be considered as the product of the formal (3+2)‐cycloaddition of D‐A cyclopropane **1 a** with HNCO (Scheme 5). Moreover, we demonstrated that the alkylation of **2 a** with dimethyl sulfate afforded thioimidate **6** (Scheme 5). This compound corresponds to the product of the formal (3+2)‐cycloaddition of D‐A cyclopropanes **1** with alkyl thiocyanates, a process that has not been studied yet.

**Scheme 6:**
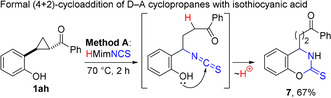
Triple role of 1‐methylimidazolium thiocyanate in the transformation of cyclopropane **1ah** to [1,3]benzoxazine‐2‐thione **7**.

To achieve further proof of concept for the triple role of PILs, we employed 1‐methylimidazolium thiocyanate to perform the annulation of isothiocyanic acid with 2‐hydroxyphenyl‐derived cyclopropane **1 ah**,^[32]^ serving as the equivalent of the corresponding *ortho*‐quinone methide.^[33]^ Indeed, under heating in HMimNCS at 70 °C, cyclopropane **1 ah** was smoothly transformed into [1,3]benzoxazine‐2‐thione **7** in reasonable yield. We believe that this reaction proceeds by a similar stepwise mechanism, including the nucleophilic opening of a three‐membered ring with thiocyanate ion followed by an attack of the *ortho*‐hydroxy group on the formed isothiocyanate moiety (Scheme 6). This process, together with the reported transformations of 2‐hydroxyphenyl‐containing D‐A cyclopropanes,[^33^, ^34^] could be responsible for the diminished yields of pyrrolidine‐2‐thiones **2 i** and **2 j** in the reactions of the corresponding cyclopropane‐1,1‐diesters **1 i,j** (Scheme 3).

**Scheme 7:**
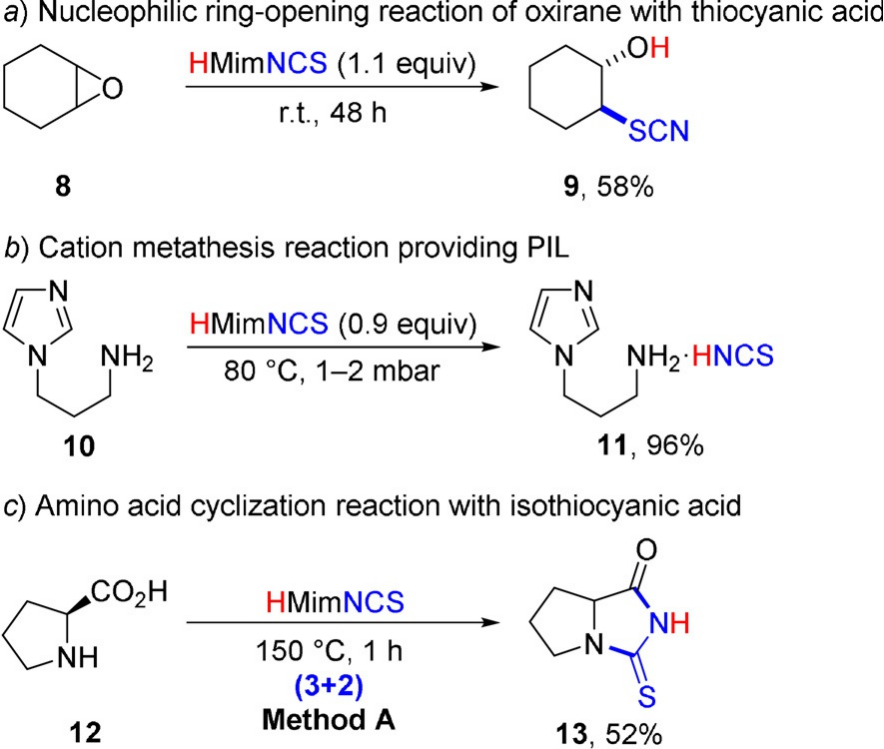
Conventional transformations involving 1‐methylimidazolium thiocyanate.

Taking into account the somewhat sophisticated character of the D‐A cyclopropanes **1** as substrates, we opted to transfer the triple role PILs concept onto organic transformations proceeding with conventional starting materials (Scheme 7). First, the treatment of cyclohexene oxide (**8**) with a stoichiometric amount of HMimNCS at r.t. resulted in the *S*‐selective nucleophilic ring‐opening producing 2‐hydroxycyclohexyl thiocyanate **9** ^[35]^ (Scheme 7 a). Heating of HMimNCS with 1‐(3‐aminopropyl)imidazole (**10**) at 80 °C led to the displacement of 1‐methylimidazole in HMimNCS with the more basic amine providing new protic ionic liquid **11** via a cation metathesis reaction (Scheme 7 b). This compound may find broad use as a room temperature PIL; further, it might possess interesting biological properties accounting for the nature of the cationic part.^[36]^ Under harsher conditions, L‐proline **12** underwent formal (3+2)‐cycloaddition with isothiocyanic acid affording bicyclic 2‐thiohydantoin **13** (Scheme 7 c). Yields of products **9** and **13** were unoptimized as our goal was only to demonstrate a principal possibility for these transformations to proceed in triple‐role protic ionic liquids. Nevertheless, it is noteworthy that the described synthesis of **13** was carried out under much milder conditions compared to the single reported one‐step preparation of this compound. Namely, the reaction of l‐proline with thiourea required heating of an inhomogeneous mixture at 170–210 °C under careful control of the reaction, as local over‐heating led to significant decrease of the yield.^[37]^ This indicates the high potential for further applications of PIL in various transformations.

## Conclusion

In summary, we have demonstrated that protic ionic liquids containing nucleophilic anions are able to serve in concert as a resplendent trio, namely, as recoverable reaction medium, as Brønsted acid, initiating the process as a catalyst, and as a source of the nucleophile. The efficiency of this strategy was exemplarily shown for the ring‐opening of donor‐acceptor cyclopropanes with the thiocyanate ion. 1‐Methylimidazolium thiocyanate was selected as an appropriate PIL possessing an almost perfect balance of acid‐base properties of components forming this reagent that can be regarded as a bench‐stable surrogate of isothiocyanic acid. Unusual chemoselectivity of the ambident thiocyanate ion was found for this process: attack of the nitrogen rather than the sulfur on the activated three‐membered ring produced isothiocyanate, which underwent immediate cyclization affording 3,5‐disubstituted pyrrolidine‐2‐thiones, products of the formal (3+2)‐cycloaddition of D‐A cyclopropanes with isothiocyanic acid. A broad scope of D‐A cyclopropanes was successfully employed. Scaling up and PIL recovery were demonstrated. Other applications of thiocyanate‐containing PILs as triplex reagents in diverse reactions were also investigated. Further development of the triple‐role PILs is in progress now; the results will be reported in due course.

## Conflict of interest

The authors declare no conflict of interest.

## Supporting information

As a service to our authors and readers, this journal provides supporting information supplied by the authors. Such materials are peer reviewed and may be re‐organized for online delivery, but are not copy‐edited or typeset. Technical support issues arising from supporting information (other than missing files) should be addressed to the authors.

Supplementary

Supplementary
