# Supplementary material for: Protic Ionic Liquid as Reagent, Catalyst, and Solvent: 1‐Methylimidazolium Thiocyanate
Source: Angew Chem Int Ed Engl. 2021 Feb 26;60(14):7927–34. doi: 10.1002/anie.202016593 (PMC8048580; doi:10.1002/anie.202016593)

Angewandte Chemie
Eine Zeitschrift der Gesellschaft Deutscher Chemiker

Supporting Information

Protic Ionic Liquid as Reagent, Catalyst, and Solvent: 1-Methylimidazolium Thiocyanate

Ivan A. Andreev[+], Nina K. Ratmanova[+], André U. Augustin, Olga A. Ivanova, Irina I. Levina, Victor N. Khrustalev, Daniel B. Werz,* and Igor V. Trushkov**

anie_202016593_sm_miscellaneous_information.pdf
anie_202016593_sm_cif.zip

Table of Contents

General Information	S2
Synthesis and characterization of thiocyanate ion-containing protic ionic liquids **3**	S2
Synthesis of D–A cyclopropanes **1**	S4
General procedure (**GP1**) for the synthesis of alkylidenemalonates **S1**	S4
General procedure (**GP2**) for the synthesis of D–A cyclopropanes **1**	S7
General procedure (**GP3**) for D–A cyclopropanes **1** opening with thiocyanate ion	S9
Regeneration of thiocyanate ion-containing protic ionic liquids	S21
Restrictions of the method (**GP3**): the list of inappropriate substrates	S21
Preliminary estimation of environmental factors (**EF**)	S22
References	S23
Copies of NMR spectra	S24

General information

NMR spectra were acquired on Bruker Avance 500 spectrometers at room temperature; the chemical shifts *δ* were measured in ppm with respect to solvent (^1H: $CDCl_3$, *δ* = 7.26 ppm; DMSO-d_6: *δ* = 2.50 ppm; acetone-d_6: 2.05 ppm; ^{13}C: $CDCl_3$, *δ* = 77.2 ppm; DMSO-d_6: *δ* = 39.5 ppm; acetone-d_6: 29.8 ppm). ^{19}F NMR spectra were recorded at 470 MHz with fluorobenzene (*ca*. 1 equiv) as an internal reference (δ = –112.96 ppm in $CDCl_3$). Splitting patterns are designated as s, singlet; d, doublet; t, triplet; q, quadruplet; quint, quintet; m, multiplet; dd, double doublet, br., broad. Coupling constants (*J*) are given in Hertz. The structures of synthesized compounds were elucidated with the aid of ^1H, ^{13}C, ^{19}F, APT, DEPT-135Q, ^1H–^{13}C HSQC, ^1H–^{13}C HMBC NMR spectroscopy. Infrared spectra were recorded on InfraLUM FT-801 and Bruker FTIR spectrometer ALPHA II. High resolution and accurate mass measurements were carried out using a BrukermicrOTOF-Q™ ESI-TOF (Electro Spray Ionization/Time of Flight) and Thermo Scientific* LTQ Orbitrap mass spectrometers. Elemental analyses were performed with Fisons EA-1108 CHNS elemental analyzer instrument. Melting points were determined using the Stuart® SMP3 melting point apparatus.

X-ray diffraction studies were performed on the X-ray Structural Analysis beamline using a Rayonix SX165 CCD-detector (*T* = 100 K, λ = 0.78790 or 0.96260 Å, φ-scanning mode with an oscillation angle of 1.0°) at the Kurchatov Synchrotron Radiation Source (NRC “Kurchatov Institute”, Moscow), and on a three-circle Bruker D8 QUEST PHOTON-III CCD diffractometer (*T* = 100 K, λ(MoK_α)-radiation, graphite monochromator, φ and ω scanning mode) at the Department of Structural Studies of Zelinsky Institute of Organic Chemistry (Moscow, Russia). Frame indexing and integration were performed using the iMOSFLM utility from the CCP4 package[S1] or SAINT program[S2]. Semi-empirical absorption correction was performed using the Scala[S3] or SADABS program[S4]. The structures were solved using the intrinsic phasing modification of direct methods and refined by full-matrix least-square method within the anisotropic approximation for all non-H atoms. All calculations were carried out using the SHELXTL program suite.[S5] Crystallographic data have been deposited with the Cambridge Crystallographic Data Center. Copies of this information may be obtained free of charge from the Director, CCDC, 12 Union Road, Cambridge CB2 1EZ, UK (fax: +44 1223 336033; e-mail: deposit@ccdc.cam.ac.uk or www.ccdc.cam.ac.uk).

Analytical thin-layer chromatography (TLC) was carried out with silica gel plates (silica gel 60, F_{254}, supported on aluminum); the revelation was done by UV lamp (254 nm) or chemical staining applying iodine vapor or aqueous potassium permanganate solution as developing agents. Flash column chromatography was performed on Macherey–Nagel silica gel (40–63 μm; 230–400 mesh). All reagents were purchased at the highest commercial quality and used without further purification unless otherwise stated. All reactions were carried out using freshly distilled and dry solvents in well-cleaned oven-dried glassware with magnetic stirring. Experimental densities of protic ionic liquids (PILs) were determined by weighing *ca*. 500 μL samples; the values are uncorrected and used only for the rough estimation of the PILs equivalents in the ring opening reactions.

Synthesis and characterization of thiocyanate ion-containing protic ionic liquids 3

Caution! 1-Methylimidiazolium chloride (HMimCl), NaSCN, and NH_4SCN are hygroscopic. These reagents should be handled as fast as possible with the minimum exposure both to air and steel weighing instruments.

Caution! In cation metathesis reactions, the initial addition of an amine to ammonium thiocyanate is negligibly exothermic, while heating of reaction mixtures was accompanied by massive ammonia evolution. Therefore, these reactions should be carried out in a fume hood.

Transferring of neat viscous PILs into other flasks may be very troublesome; pre-weighed flasks were used for the synthesis of PILs **3b**, **3c**.

The synthesized PILs were distributed in equal portions to 40 mL vials in order to prolong the storage and simplify the melting and sampling of the PIL during the reaction setup.

Comments to the characterization of PILs: For all PILs, signals of acidic protons in ^1H NMR spectra are significantly broadened; their shifts are functions of concentration and could not be estimated accurately. Shifts of other signals have small concentration dependence. To ensure the reproducibility of elemental analysis, the sample should be prepared under low indoor humidity.

Growing single crystals for X-ray analysis of PILs 3a, 3b: Colorless long needles appropriate for single-crystal X-ray analysis were achieved upon mechanical impact with a spatula on a melted PIL cooled to *ca*. 30 °C. Separated crystals were quickly drawn out with a spatula from the amorphous crystallizing mass before the complete solidification occurred. The resulting crystals had a similar m.p. and satisfactory elemental analysis.

1-Methylimidazolium thiocyanate (HMimNCS, 3a)

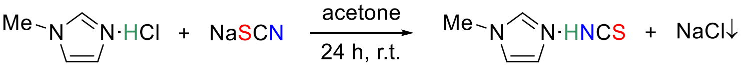

1-Methylimidazolium thiocyanate was prepared according to the modified literature procedure.[S6] A mixture of HMimCl (50.0 g, 422 mmol) and sodium thiocyanate (34.2 g, 422 mmol) in abs. acetone (420 mL) was vigorously stirred for 24 h at r.t. The precipitated NaCl was filtered off and washed with acetone (3 × 33 mL). The filtrate was concentrated on a rotary evaporator. The resulting warm red-orange oily liquid was washed with EtOAc (3 × 50 mL) and CH_2Cl_2 (2 × 33 mL); in each case, the upper organic layer was decanted off and discarded. The resulting PIL was dried at 80 °C (5 mbar) until the constant weight was achieved (5–6 h), affording HMimNCS as an orange moderately viscous liquid (59.0 g, 99%, $d_{exp.}$ ≈ 1.21). Being cooled to r.t., PIL becomes supersaturated. Spontaneous solidification (Fig. S1) accompanied by a heat evolution occurred upon mechanical impact (shaking, touching with a spatula or a pipette tip during NMR preparation), resulting in an orange-yellow solid; m.p. 38–41 °C.

The preparation of HMimNCS was successfully scaled up threefold according to the above conditions providing 177.5 g (> 99% yield) of crude PIL. It was washed with EtOAc and CH_2Cl_2 and dried under a high vacuum affording HMimNCS (171.3 g, 96%).
^{1}H NMR (500 MHz, DMSO-d$_6$): δ = 3.86 (s, 3H, CH_3), 7.62 (dd, ^3J = 1.7 Hz, ^4J = 1.4 Hz, 1H, CH, Im), 7.66 (dd, ^3J = 1.7 Hz, ^4J = 1.4 Hz, 1H, CH, Im), 9.00 (br. s, 1H, CH, Im), 12.60 (br. s, 1H, NH^+···[NCS]).
^{13}C NMR (126 MHz, DMSO-d$_6$): δ = 35.5 (CH_3), 119.6 (CH, Im), 123.2 (CH, Im), 130.1 (NCS), 135.7 (CH, Im).
IR (KBr): ṽ = 3467 (s), 3135 (vs), 2210 (w), 2052 (m, NCS), 1584 (m), 1550 (m), 1461 (m), 1381 (w), 1332 (w), 1316 (w), 1281 (m), 1188 (w), 1087 (m), 1016 (w), 943 (m), 902 (m), 838 (s), 755 (s) cm^{-1}.
HRMS (ESI-TOF): *m/z* calcd for $C_4H_7N_2^+$: 83.0604 [HMim]$^+$; found: 83.0601.
Elemental analysis calcd (%) for $C_5H_7N_3S$: C 42.53, H 5.00, N 29.76; found: C 42.52, H 4.99, N 29.81.

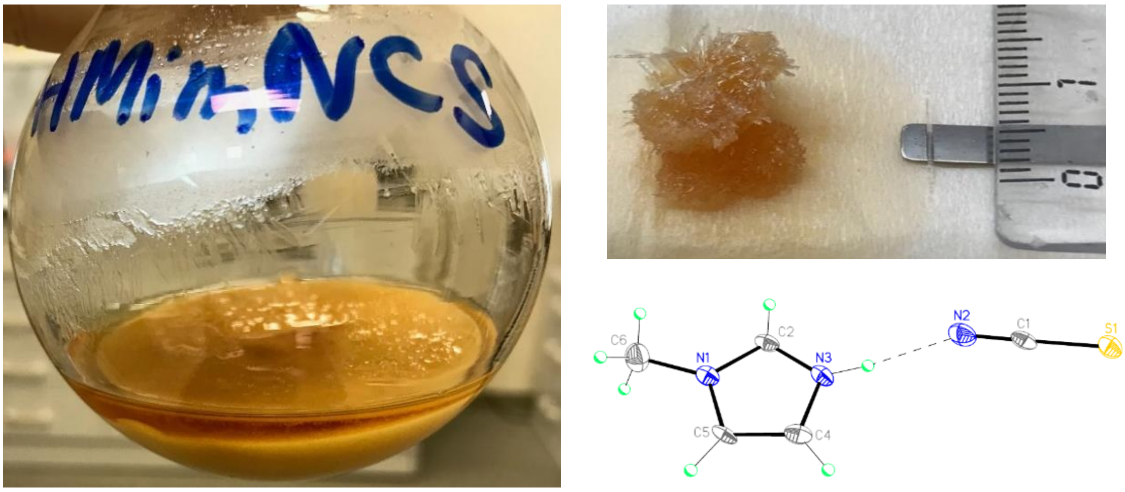

Figure S1. Single-crystal X-ray structure of 1-methylimidazolium thiocyanate **3a** (thermal ellipsoids are shown at 50% probability; CCDC 1995823) and its appearance.

Triethylammonium thiocyanate (Et$_3$N·HNCS, 3b)

$$\ddot{\mathrm{N}}\mathrm{Et_3} + \mathrm{NH_4SCN} \xrightarrow[\text{1 h, }\Delta]{\text{MeOH}} \mathrm{Et_3N\cdot HNCS}$$

Triethylammonium thiocyanate was prepared by a combination of literature procedures applied earlier for the synthesis of various ammonium thiocyanates.[S7, S8] After almost complete dissolution of ammonium thiocyanate (74.5 g, 978 mmol) in MeOH (200 mL, HPLC quality), the reaction mixture was treated under stirring with Et_3N (150 mL, 1.08 mol) in a single portion. The resulting solution was refluxed for 1–1.5 h. The reaction mixture was concentrated on a rotary evaporator. The residual PIL was dried at 80 °C (5 mbar) until the constant weight was achieved (5–6 h), affording Et_3N·HNCS as an orange oily liquid (154.3 g, 98%, $d_{exp.}$ ≈ 0.99–1.00; Fig. S2). The product was cooled to r.t. Spontaneous solidification accompanied by a heat evolution occurred upon mechanical impact resulting in a yellow-orange solid; m.p. = 47–48 °C (lit. = 50.5–51.5 °C, EtOAc/EtOH).[S7] The completeness of the cation metathesis was controlled by the "ammonium test" during the ^{13}C NMR sample preparation. Cation metathesis can be referred to as complete if the dissolution of Et_3N·HNCS (*ca.* 150 mg) in $CDCl_3$ (*ca.* 550 µL) was not accompanied by the appearance of a visible suspension of NH_4SCN. Spectral data are well consistent with the published ones.[S7]
^{1}H NMR (500 MHz, $CDCl_3$): δ = 0.96 (t, ^3J = 7.3 Hz, 9H, 3 × CH_3), 2.77 (dq, ^3J = 7.3 Hz, ^3J = 4.9 Hz, 6H, 3 × CH_2), 9.44 (br. s, 1H, NH^+···[NCS]).
^{13}C NMR (126 MHz, $CDCl_3$): δ = 8.0 (3 × CH_3), 45.7 (3 × CH_2), 131.8 (NCS).
IR (KBr): ṽ = 3467 (w), 2985 (m), 2682 (m), 2498 (m), 2053 (vs, NCS), 1727 (w), 1658 (w), 1476 (s), 1396 (m), 1162 (m), 1034 (m), 945 (w), 838 (m), 795 (w), 765 (w) cm^{-1}.
HRMS (ESI-TOF): *m/z* calcd for $C_6H_{16}N^+$: 102.1277 [Et$_3$NH]$^+$; found: 102.1279.
Elemental analysis calcd (%) for $C_7H_{16}N_2S$: C 52.46, H 10.06, N 17.48; found: C 52.54, H 10.24, N 17.71.

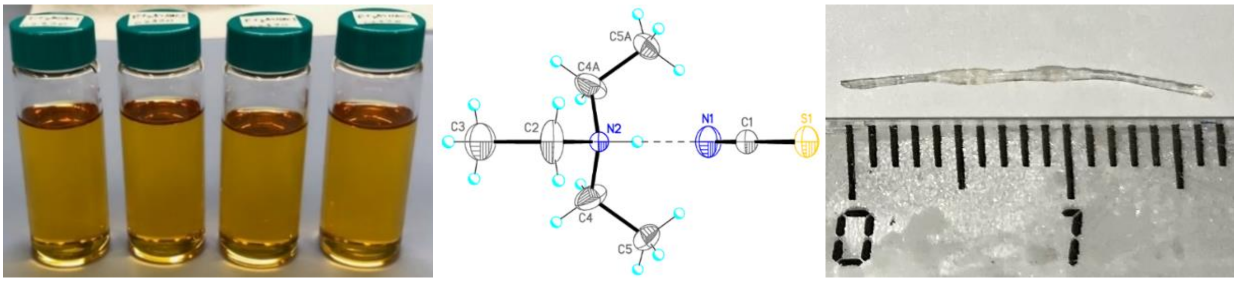

Figure S2. The single-crystal X-ray structure of triethylammonium thiocyanate **3b** (thermal ellipsoids are shown at 50% probability; CCDC 1995824) and its appearance in a melted state.

The preparation of $Et_3N{\cdot}HNCS$ by an anion metathesis reaction

Despite numerous synthetic efforts, the anion metathesis reaction performed under the analogous to the HMimNCS preparation conditions provided $Et_3N{\cdot}HNCS$ reagent of inferior quality. The obtained samples contained unreacted $Et_3N{\cdot}HCl$ as a suspension of a colorless solid in the orange PIL melt (Fig. S3). Attempts to grow crystals for X-ray analysis resulted only in the triethylammonium chloride crystallization.

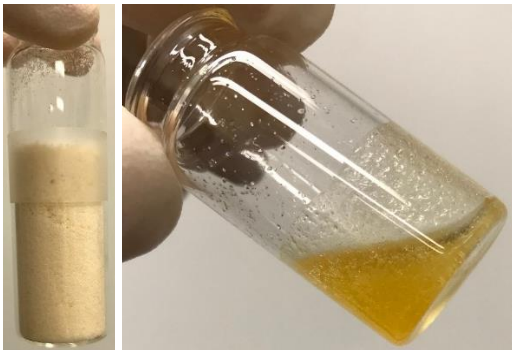

Figure S3. The appearance of $Et_3N{\cdot}HNCS$ (**3b**) prepared by an anion metathesis reaction at r.t. and in a melted state.

***N*,*N*,*N'*,*N'*-Tetramethylguanidinium thiocyanate (HTmgNCS, 3c)**

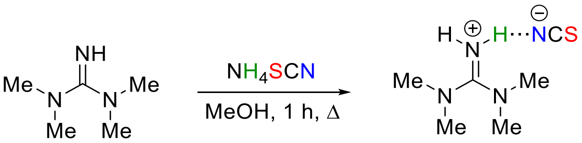

Tetramethylguanidinium thiocyanate **3c** was prepared by the procedure analogous to that described above for triethylammonium thiocyanate using ammonium thiocyanate (4.62 g, 60.7 mmol), tetramethylguanidine (8.0 mL, 63.8 mmol), and MeOH (12.1 mL). The resulting pale-yellow solution was stirred for 1 h at r.t. and concentrated on a rotary evaporator. The residual PIL was dried at 80 °C (3–4 mbar) until the constant weight was achieved (5–6 h), affording HTmgNCS as a colorless extremely viscous oil (10.5 g, 99%; $d_{exp.} \approx 1.09$ at *ca*. 50 °C). All sampling operations were performed with a pre-heated to 45–50 °C PIL to minimize difficulties associated with its high viscosity. PIL passed the ammonium test.

^{1}H NMR (500 MHz, $CDCl_3$): δ = 2.84 (s, 12H, 4 × CH_3), 7.13 (br. s, 2H, NH_2).
^{13}C NMR (126 MHz, $CDCl_3$): δ = 39.6 (4 × CH_3), 132.2 (br. s, NCS), 160.9 ($H_2N{=}C^+$).
IR (film): ṽ = 3341 (s), 3179 (s), 2728 (w), 2527 (w), 2057 (s, NCS), 1657 (s, C=N), 1611 (s, C=N), 1565 (s), 1453 (m), 1411 (s), 1319 (w), 1263 (m), 1142 (m), 1063 (m), 1039 (m), 945 (w), 875 (w), 717 (m) cm^{-1}.
HRMS (ESI-TOF): *m/z* calcd for $C_5H_{14}N_3^+$: 116.1182 [HTmg]$^+$; found: 116.1178.

Synthesis of D–A cyclopropanes 1

Starting D–A cyclopropanes **1** were prepared by Knoevenagel/Corey–Chaykovsky reactions sequence from the corresponding aldehydes according to the modified literature procedures.[S9, S10] Some compounds were fully characterized earlier; characterization of other compounds is given below.

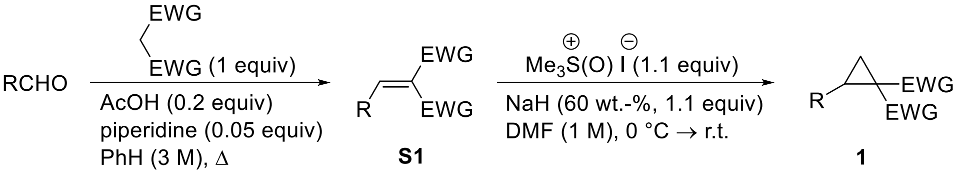

General procedure (GP1) for the synthesis of alkylidenemalonates S1

The solution of aldehyde, active methylene compound (AMC, 1 equiv), AcOH (0.2 equiv), and piperidine (0.05 equiv) in benzene (aldehyde concentration *ca.* 3 M) was refluxed with a Dean–Stark trap for 4–5 h with dimethyl malonate and 1–3 h with other AMCs. (Dean–Stark trap was filled with benzene in advance to prevent the solvent loss in the flask during the azeotropic distillation of water). In syntheses of **S1n**, **S1o**, **S1v**, triethylammonium triacetate[S11] (0.1 equiv) was added to the reaction mixture to facilitate the water separation. The reaction mixture was concentrated on a rotary evaporator and dried at 60 °C. The residual dark-colored viscous oil was dissolved in isopropyl alcohol (IPA, 1 mL per 1 g of substrate, 1 vol.) and treated with petroleum ether (PE, 1 vol.). The resulting mixture was placed into a freezer and kept overnight. The precipitated product was crushed with a spatula, filtered, washed with PE (3–4 times by 2 vol. each). If the precipitate of **S1** was dark-colored or sticky, it was washed with chilled IPA (1–2 × 2 vol.) prior to PE washings. The product was dried on air to afford Knoevenagel adduct **S1** as a solid. In the case of liquid products, crude **S1** was either purified by flash column chromatography or subjected to the next step without purification.

Dimethyl 2-(2,4-dimethoxybenzylidene)malonate (S1f) was synthesized according to the **GP1** from 2,4-dimethoxybenzaldehyde (30.00 g, 181 mmol). Product **S1f** was obtained as a yellowish-white solid (48.02 g, 95%); R_f = 0.30 (PE/EtOAc, 3:1); m.p. = 75–76 °C.

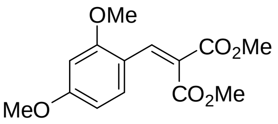

^{1}H NMR (500 MHz, $CDCl_3$):[S12] δ = 3.80 (s, 3H, CH_3O), 3.81 (s, 3H, CH_3O), 3.82 (s, 3H, CH_3O), 3.83 (s, 3H, CH_3O), 6.42 (d, ^4J = 2.3 Hz, 1H, Ar), 6.45 (dd, ^3J = 8.6 Hz, ^4J = 2.3 Hz, 1H, Ar), 7.29 (d, ^3J = 8.6 Hz, 1H, Ar), 8.06 (s, 1H, CH=).
^{13}C NMR (126 MHz, $CDCl_3$): δ = 52.4 (2 × CH_3O), 55.4 (CH_3O), 55.5 (CH_3O), 98.2 (CH), 105.3 (CH), 114.9 (C), 122.5 (C), 130.3 (CH), 138.4 (CH), 159.8 (C), 163.4 (C), 165.1 (*C*O_2Me), 167.8 (*C*O_2Me).
IR (KBr): ṽ = 3428 (w), 2954 (w), 2842 (w), 2139 (w), 2052 (w), 1720 (s, C=O), 1601 (s, C=C), 1508 (s), 1461 (s), 1439 (s), 1376 (m), 1325 (s), 1287 (s), 1214 (vs), 1171 (s), 1115 (vs), 1068 (s), 1028 (vs), 984 (s), 940 (s), 824 (vs), 763 (m), 716 (m) cm^{-1}.
HRMS (ESI-TOF): *m/z* calcd for $C_{14}H_{17}O_6$: 281.1020 $[M+H]^+$; found: 281.1015.
Elemental analysis calcd (%) for $C_{14}H_{16}O_6$: C 59.99, H 5.75; found: C 60.08, H 5.76.

Dimethyl 2-(2,6-dimethoxybenzylidene)malonate (S1k) was synthesized according to the **GP1** from 2,6-dimethoxybenzaldehyde (10.16 g, 61.2 mmol). The residual dark-orange viscous oil was dissolved in isopropanol (IPA, 10 mL) and treated with PE (10 mL). The resulting solution was placed into a freezer and kept overnight. The precipitated product was crushed with a spatula, filtered, washed with PE (4 × 20 mL), and dried on air to afford **S1k** as an ivory solid (17.02 g, 99%); R_f = 0.16 (PE/EtOAc, 3:1); m.p. = 91–94 °C.

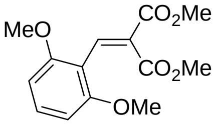

^{1}H NMR (500 MHz, $CDCl_3$): δ = 3.64 (s, 3H, CH_3O), 3.73 (s, 6H, 2 × CH_3O), 3.77 (s, 3H, CH_3O), 6.47 (d, ^3J = 8.4 Hz, 2H, Ar), 7.24 (t, ^3J = 8.4 Hz, 1H, Ar), 7.93 (s, 1H, CH=).
^{13}C NMR (126 MHz, $CDCl_3$): δ = 51.6 (CH_3O), 52.3 (CH_3O), 55.4 (2 × CH_3O), 103.4 (2 × CH), 111.7 (C), 126.3 (CH), 132.0 (CH), 137.4 (2 × C), 158.4 (CH), 165.6 (*C*O_2Me), 166.3 (*C*O_2Me).
IR (KBr): ṽ = 3005 (w), 2952 (m), 2846 (w), 1744 (s, C=O), 1721 (s, C=O), 1596 (m, C=C), 1474 (m), 1435 (m), 1386 (m), 1312 (m), 1266 (s), 1249 (s), 1117 (s), 1063 (s), 1028 (m), 779 (m) cm^{-1}.
HRMS (ESI-TOF): *m/z* calcd for $C_{14}H_{17}O_6$: 281.1020 $[M+H]^+$; found: 281.1022.

Dimethyl 2-(4-dimethylamino-2-nitrobenzylidene)malonate (S1n) was synthesized according to the **GP1** from 4-dimethylamino-2-nitrobenzaldehyde (2.00 g, 10.3 mmol). The reaction mixture was diluted to 30 mL with benzene, treated with PE (90 mL), and cooled in an ice bath. Filtration, washing with PE (4 × 20 mL), and drying on air afforded **S1n** as a bright-red voluminous solid (2.94 g, 92%); R_f = 0.18 (PE/EtOAc, 3:1); m.p. = 127–128 °C.

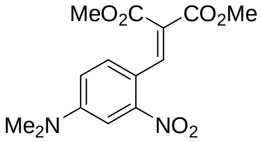

^{1}H NMR (500 MHz, $CDCl_3$): δ = 3.02 (s, 6H, $(CH_3)_2N$), 3.68 (s, 3H, CH_3O), 3.79 (s, 3H, CH_3O), 6.77 (dd, ^3J = 8.8 Hz, ^4J = 2.4 Hz, 1H, Ar), 7.19–7.26 (m, 2H, Ar), 7.99 (s, 1H, CH=).
^{13}C NMR (126 MHz, $CDCl_3$): δ = 40.0 ($(CH_3)_2N$), 52.5 (CH_3O), 52.6 (CH_3O), 107.0 (CH), 115.0 (C), 115.5 (CH), 125.0 (C), 130.7 (CH), 140.7 (CH), 149.3 (C), 151.3 (C), 164.4 (*C*O_2Me), 166.7 (*C*O_2Me).
IR (KBr): ṽ = 2951 (w), 2654 (w), 2053 (w), 1731 (vs, C=O), 1720 (vs, C=O), 1626 (s, C=C), 1603 (vs, C=C), 1545 (s), 1514 (s), 1434 (s), 1387 (m), 1354 (m), 1266 (vs), 1221 (vs), 1168 (s), 1072 (s), 986 (w), 881 (m), 847 (w), 809 (m), 766 (m), 746 (w), 711 (w) cm^{-1}.
HRMS (ESI-TOF): *m/z* calcd for $C_{14}H_{17}N_2O_6$: 309.1081 $[M+H]^+$; found: 309.1081.

Dimethyl 2-{[4′-methoxy-(1,1′-biphenyl)-4-yl]methylene}malonate (S1o)

4′-Methoxy-[1,1′-biphenyl]-4-carbaldehyde was prepared by conventional Suzuki cross-coupling. A 50 mL Schlenk tube was charged with (4-methoxyphenyl)boronic acid (2.34 g, 15.4 mmol), 4-bromobenzaldehyde (2.37 g, 12.8 mmol), K_2CO_3 (5.32 g, 38.5 mmol), and 50% aq. EtOH (26 mL). Pd(dppf)Cl_2 (94 mg, 0.13 mmol) was added under stream of Ar. The resulting reaction mixture was refluxed under an inert atmosphere for *ca*. 3.5 h and poured into an excess of cold water, causing massive precipitation. Solids were filtered off, washed with cold water (5 × 20 mL), and dissolved in Et_2O (4 × 25 mL). The solution was filtered. The organic layer was separated, dried with anhydrous Na_2SO_4, and concentrated under reduced pressure on a rotary evaporator, providing 4′-methoxy-[1,1′-biphenyl]-4-carbaldehyde as a pale-brown solid (2.64 g, 97%). Spectral data are well consistent with the published ones.[S13]

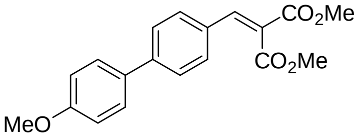

Compound **S1o** was synthesized according to the **GP1** from 4′-methoxy-[1,1′-biphenyl]-4-carbaldehyde (2.00 g, 9.42 mmol). The reaction mixture was left overnight at r.t. Filtration of the precipitate, washing with PE (4 × 15 mL), and drying on air afforded **S1o** as a sparkling brown solid (2.70 g, 88%); R_f = 0.47 (PE/EtOAc, 3:1); m.p. = 139–141 °C.
^{1}H NMR (500 MHz, $CDCl_3$): δ = 3.857 (s, 3H, CH_3O), 3.859 (s, 3H, CH_3O), 3.88 (s, 3H, CH_3O), 6.99 (d, ^3J = 8.8 Hz, 2H, Ar), 7.48 (d, ^3J = 8.3 Hz, 2H, Ar), 7.55 (d, ^3J = 8.8 Hz, 2H, Ar), 7.58 (d, ^3J = 8.3 Hz, 2H, Ar), 7.79 (s, 1H, CH=).
^{13}C NMR (126 MHz, $CDCl_3$): δ = 52.65 (CH_3O), 52.73 (CH_3O), 55.3 (CH_3O), 114.4 (2 × CH, Ar), 124.8 (C=), 126.9 (2 × CH, Ar), 128.2 (2 × CH, Ar), 130.1 (2 × CH, Ar), 130.9 (C, Ar), 132.2 (C, Ar), 142.6 (CH=), 143.1 (C, Ar), 159.8 (C, Ar), 164.6 (*C*O_2Me), 167.3 (*C*O_2Me).
IR (KBr): ṽ = 3449 (w), 3003 (w), 2951 (w), 1917 (w), 1727 (vs, C=O), 1624 (s, C=C), 1602 (s, C=C), 1523 (m), 1496 (s), 1434 (s), 1319 (m), 1263 (s), 1226 (s), 1189 (s), 1178 (s), 1129(m), 1085 (m), 1037 (m), 853 (m), 833 (s), 815 (s), 785 (w), 725 (w) cm^{-1}.
HRMS (ESI-TOF): *m/z* calcd for $C_{19}H_{19}O_5$: 327.1227 $[M+H]^+$; found: 327.1225.

Dimethyl 2-[(6-methoxynaphthalen-2-yl)methylene]malonate (S1p) was synthesized according to the **GP1** from 6-methoxy-2-naphthaldehyde (5.00 g, 26.9 mmol). Upon cooling to r.t., the product precipitated; it was refrigerated overnight. Filtration, crushing with a spatula, washing with PE (4 × 20 mL), and drying on air afforded **S1p** as a pale-orange solid (7.63 g, 95%); R_f = 0.47 (PE/EtOAc, 3:1); m.p. = 109–110 °C.

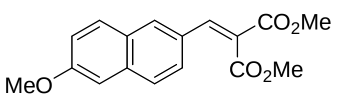

^{1}H NMR (500 MHz, $CDCl_3$): δ = 3.85 (s, 3H, CH_3O), 3.877 (s, 3H, CH_3O), 3.883 (s, 3H, CH_3O), 7.07 (d, ^4J = 2.5 Hz, 1H, Ar), 7.14 (dd, ^3J = 9.0 Hz, ^4J = 2.5 Hz, 1H, Ar), 7.42 (dd, ^3J = 8.6 Hz, ^4J = 1.8 Hz, 1H, Ar), 7.66 (d, ^3J = 8.6 Hz, 1H, Ar), 7.69 (d, ^3J = 9.0 Hz, 1H, Ar), 7.82 (br. s, 1H, Ar), 7.88 (s, 1H, CH=).

^{13}C NMR (126 MHz, $CDCl_3$): δ = 52.6 (CH_3O), 52.7 (CH_3O), 55.4 (CH_3O), 105.8 (CH), 119.7 (CH), 124.2 (C), 125.7 (CH), 127.4 (CH), 128.0 (C), 128.5 (C), 130.4 (CH), 131.2 (CH), 135.8 (C), 143.2 (CH), 159.3 (C), 164.7 (CO$_2$Me), 167.5 (CO$_2$Me).

IR (KBr): ṽ = 3425 (w), 3002 (m), 2954 (m), 2844 (m), 2047 (w), 1930 (w), 1720 (s, C=O), 1610 (s, C=C), 1482 (m), 1435 (m), 1395 (m), 1342 (m), 1254 (s), 1215 (s), 1173 (s), 1085 (s), 1028 (s), 991 (m), 865 (m), 803 (m), 755 (m), 727 (w) cm^{-1}.

HRMS (ESI-TOF): *m/z* calcd for $C_{17}H_{17}O_5$: 301.1071 $[M+H]^+$; found: 301.1068.

Dimethyl 2-[(1-methyl-1*H*-pyrrol-2-yl)methylene]malonate (S1v) was synthesized according to the **GP1** from *N*-methylpyrrole-2-carboxaldehyde (5.00 g, 45.8 mmol). Product **S1v** was obtained as a dark-pink solid (9.32 g, 91%); R_f = 0.35 (PE/EtOAc, 3:1); m.p. = 87–88 °C (lit. = 87–87.5 °C, MeOH).[S14]

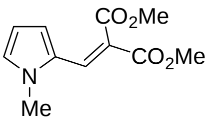

^{1}H NMR (500 MHz, $CDCl_3$): δ = 3.67 (s, 3H, CH_3N), 3.77 (s, 3H, CH_3O), 3.85 (s, 3H, CH_3O), 6.15 (dd, ^3J = 3.5 Hz, ^3J = 2.9 Hz, 1H, C(4)H, Pyr), 6.56 (br. d, ^3J = 3.5 Hz, 1H, C(3)H, Pyr), 6.78 (br. s, 1H, C(5)H, Pyr), 7.60 (s, 1H, CH=).

^{13}C NMR (126 MHz, $CDCl_3$): δ = 34.1 (CH_3N), 52.3 (CH_3O), 52.6 (CH_3O), 110.3 (C(4)H, Pyr), 114.9 (C(3)H, Pyr), 117.8 (C=), 126.4 (C(2), Pyr), 128.2 (C(5)H, Pyr), 129.4 (CH=), 165.2 (CO$_2$Me), 167.7 (CO$_2$Me).

IR (KBr): ṽ = 3435 (w), 3123 (m), 3000 (w), 2951 (m), 2057 (w), 1725 (s, C=O), 1686 (s, C=O), 1609 (s, C=C), 1486 (m), 1440 (m), 1412 (m), 1319 (m), 1245 (s), 1215 (s), 1065 (m), 981 (m), 944 (m), 743 (m) cm^{-1}.

HRMS (ESI-TOF): *m/z* calcd for $C_{11}H_{14}NO_4$: 224.0917 $[M+H]^+$; found: 224.0919.

Dimethyl 2-[(ferrocenyl)methylene]malonate (S1x) was synthesized according to the **GP1** from ferrocenecarboxaldehyde (5.00 g, 23.4 mmol). Product **S1x** was obtained as a reddish-brown solid (6.58 g, 86%); R_f = 0.57 (PE/EtOAc, 3:1); m.p. = 97–99 °C (dec.).

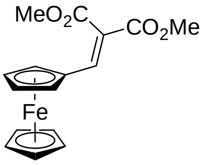

^{1}H NMR (500 MHz, $CDCl_3$): δ = 3.80 (s, 3H, CH_3O), 3.89 (s, 3H, CH_3O), 4.20 (br. s, 5H, Cp), 4.44 (br. s, 2H, Cp), 4.48 (br. s, 2H, Cp), 7.62 (s, 1H, CH=).

^{13}C NMR (126 MHz, $CDCl_3$): δ = 52.3 (CH_3O), 52.4 (CH_3O), 70.0 (5 × CH, Cp), 70.5 (2 × CH, Cp), 72.1 (2 × CH, Cp), 75.2 (C, Cp), 120.5 (C=), 144.5 (CH=), 164.8 (CO$_2$Me), 167.7 (CO$_2$Me).

IR (KBr): ṽ = 3094 (w), 2056 (w), 1891 (w), 1734 (s, C=O), 1715 (s, C=O), 1616 (s, C=C), 1438 (m), 1384 (w), 1367 (w), 1331 (w), 1271 (s), 1250 (s), 1214 (s), 1183 (s), 1106 (w), 1067 (m), 1051 (m), 987 (w), 953 (w), 838 (w), 820 (m), 762 (w), 749 (w) cm^{-1}.

HRMS (ESI-TOF): *m/z* calcd for $C_{16}H_{16}FeO_4$: 328.0393 $[M]^+$; found: 328.0406.

Dimethyl 2-[(*E*)-3-(4-fluorophenyl)prop-2-en-1-ylidene]malonate (S1ab) was synthesized according to the **GP1** from *trans*-4-fluorocinnamaldehyde (5.01 g, 33.4 mmol). A dark-orange solid residue was subjected to the crystallization from IPA affording **S1ab** as a yellow-orange solid (6.91 g, 78%); R_f = 0.55 (PE/EtOAc, 3:1); m.p. = 88–89 °C.

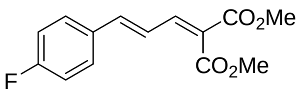

^{1}H NMR (500 MHz, $CDCl_3$): δ = 3.78 (s, 3H, CH_3O), 3.86 (s, 3H, CH_3O), 6.96 (d, ^3J = 15.5 Hz, 1H, CH=), 7.02 (dd, ^3J = 8.8 Hz, $^3J_{HF}$ = 8.5 Hz, 2H, Ar), 7.16 (dd, ^3J = 15.5 Hz, ^3J = 11.6 Hz, 1H, CH=), 7.44 (dd, ^3J = 8.8 Hz, $^4J_{HF}$ = 5.4 Hz, 2H, Ar), 7.50 (d, ^3J = 11.6 Hz, 1H, CH=).

^{13}C NMR (126 MHz, $CDCl_3$): δ = 52.3 (CH_3O), 52.4 (CH_3O), 116.0 (d, $^2J_{CF}$ = 22 Hz, 2 × CH, Ar), 123.0 (d, $^6J_{CF}$ = 3 Hz, CH=), 124.1 (C=), 129.7 (d, $^3J_{CF}$ = 9 Hz, 2 × CH, Ar), 131.8 (d, $^4J_{CF}$ = 4 Hz, C, Ar), 143.7 (CH=), 146.0 (CH=), 163.6 (d, $^1J_{CF}$ = 252 Hz, C, Ar), 165.1 (CO$_2$Me), 165.7 (CO$_2$Me).

^{19}F NMR (470 MHz, $CDCl_3$): δ = –109.8.

IR (KBr): ṽ = 3422 (w), 3038 (m), 2952 (m), 2845 (w), 2455 (w), 2304 (w), 2049 (w), 1897 (w), 1712 (s, C=O), 1619 (m, C=C), 1593 (s, C=C), 1508 (m), 1439 (m), 1246 (s), 1153 (s), 1067 (s), 1000 (m), 932 (m), 869 (w), 835 (m), 812 (m), 765 (m), 730 (m) cm^{-1}.

HRMS (ESI-TOF): *m/z* calcd for $C_{14}H_{14}FO_4$: 265.0871 $[M+H]^+$; found: 265.0863.

4-[(2,2-Dimethyl-4,6-dioxo-1,3-dioxan-5-ylidene)methyl]benzonitrile (S1af)[S15] was synthesized according to the **GP1** from 4-cyanobenzaldehyde (3.00 g, 22.9 mmol) and Meldrum's acid (3.30 g, 22.9 mmol). The reaction mixture was cooled in an ice bath to induce precipitation, then refrigerated for 2–3 h. Filtration, washing with PE (4 × 25 mL), and drying on air afforded **S1af** as a light-yellow solid (5.56 g, 94%); R_f = 0.44 (PE/EtOAc, 3:1); m.p. = 160–162 °C (lit. = 161–162 °C, MeOH;[S16] 169 °C;[S15] 180 °C, EtOH).[S17] Spectral data are well consistent with the published ones.[S15, S16]

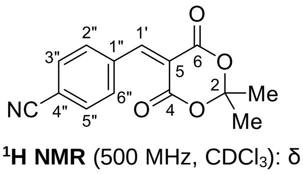

^{1}H NMR (500 MHz, $CDCl_3$): δ = 1.82 (s, 6H, 2 × CH_3), 7.75 (d, ^3J = 8.2 Hz, 2H, C(2'')H, C(6'')H, Ar), 8.02 (d, ^3J = 8.2 Hz, 2H, C(3'')H, C(5'')H, Ar), 8.40 (s, 1H, CH=).

^{13}C NMR (126 MHz, $CDCl_3$): δ = 27.7 (2 × CH_3), 105.2 (C(2)Me$_2$), 115.5 (C(1''), Ar), 117.9 (C≡N), 118.0 (C=), 132.1 (2 × CH, C(2'')H, C(6'')H, Ar), 132.6 (2 × CH, C(3'')H, C(5'')H, Ar), 135.7 (C(4''), Ar), 154.9 (C(1')H=), 159.0 (CO_2R), 162.1 (CO_2R).

General procedure (GP2) for the synthesis of D–A cyclopropanes 1

Sodium hydride (60% suspension in mineral oil; 1.1 equiv) was added in small portions under Ar atmosphere, vigorous stirring, and external ice-bath cooling to dry dimethylformamide (DMF, 1 vol.). The resulting suspension was treated in small portions with trimethylsulfoxonium iodide (1.1 equiv). **Caution!** *The addition of trimethylsulfoxonium iodide was accompanied by hydrogen and heat evolution. Thus, the next portion was introduced after the H_2 evolution from the previous portion had ceased.* The gray suspension was stirred for additional 10–15 min and treated in a few portions with **S1** (1 equiv, the final concentration of **S1** was *ca.* 1 M). The cooling was removed, and the resulting viscous suspension was stirred at r.t. for 2–3 h until the full consumption of the starting material. The reaction mixture was carefully poured into the mixture of cold water (1 vol.), ice (1 vol.), and AcOH (1.2 equiv) and extracted with EtOAc (3 × 2 vol.). The combined organic fractions were washed successively with saturated $NaHCO_3$ solution (2 × 1 vol.), H_2O (2 × 1 vol.), and saturated NaCl solution (1 vol.). The organic extracts were dried with anhydrous Na_2SO_4 and concentrated under reduced pressure on a rotary evaporator. The residual turbid oil was dissolved in hot Et_2O (2 mL per 1 g of crude D–A cyclopropane), rubbed with a spatula, placed in a freezer, and left overnight. The precipitated product was crushed with a spatula, filtered, washed with PE (4 × vol. of Et_2O). If the initially filtered precipitate of cyclopropane **1** was colored or sticky, it was washed with chilled Et_2O (1–2 × 1 vol.) prior to PE washings. The residue was dried on air to afford **1** as a solid. This crystallization procedure was optimal for diverse alkoxyphenyl- and *p*-(dialkylamino)phenyl-substituted D–A cyclopropanes. In the case of liquid or low-melting D–A cyclopropanes, crude **1** was purified by flash column chromatography.

The reverse-addition procedure. In the case of specified D–A cyclopropanes, the suspension of freshly prepared dimethylsulfoxonium methylide (1 M in DMF) was added in small portions to 1 M solution of **S1** in DMF under ice-bath cooling. The reaction mixture was poured into ice-cold saturated NH_4Cl solution (294 g/L, Σ DMF vol.) and worked-up as usual.

Dimethyl 2-(2,4-dimethoxyphenyl)cyclopropane-1,1-dicarboxylate (1f) was synthesized according to the **GP2** from **S1f** (30.00 g, 107 mmol), affording **1f** as a yellowish-white solid (16.77 g, 53%). The second crop was isolated from the evaporated filtrate. The orange residue (*ca*. 8 g) was dissolved in hot IPA (16 mL), cooled to r.t., seeded with the first portion, placed in a freezer, and left overnight. Filtration, washing with chilled IPA (3 × 20 mL), PE (3 × 20 mL), and drying on air provided a pale-yellow solid (2.97 g, *ca*. 9%). The total yield of **1f** was 19.74 g, 63%. R_f = 0.36 (PE/EtOAc, 3:1); m.p. = 72–74 °C.

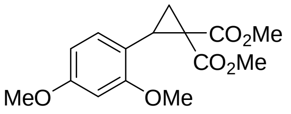

¹H NMR (500 MHz, $CDCl_3$): δ = 1.70 (dd, ^2J = 5.0 Hz, ^3J = 9.2 Hz, 1H, CH_2), 2.16 (dd, ^2J = 5.0 Hz, ^3J = 8.4 Hz, 1H, CH_2), 3.24 (dd, ^3J = 9.2 Hz, ^3J = 8.4 Hz, 1H, CH), 3.36 (s, 3H, CH_3O), 3.776 (s, 3H, CH_3O), 3.782 (s, 3H, CH_3O), 3.79 (s, 3H, CH_3O), 6.37 (dd, ^3J = 8.4 Hz, ^4J = 2.3 Hz, 1H, Ar), 6.40 (d, ^4J = 2.3 Hz, 1H, Ar), 6.88 (d, ^3J = 8.4 Hz, 1H, Ar).
¹³C NMR (126 MHz, $CDCl_3$): δ = 18.7 (CH_2), 28.4 (CH), 36.2 (C), 52.1 (CH_3O), 52.6 (CH_3O), 55.3 (CH_3O), 55.6 (CH_3O), 98.2 (CH, Ar), 103.4 (CH, Ar), 115.5 (C, Ar), 128.5 (CH, Ar), 160.1 (C, Ar), 160.3 (C, Ar), 167.4 (CO_2Me), 170.5 (CO_2Me).
IR (KBr): ṽ = 3436 (w), 2994 (m), 2953 (m), 2841 (m), 2505 (w), 2234 (w), 2065 (w), 1884 (w), 1729 (s, C=O), 1609 (s), 1589 (m), 1512 (s), 1434 (s), 1381 (m), 1279 (s), 1212 (s), 1137 (s), 1111 (s), 1037 (s), 942 (m), 895 (m), 863 (m), 825 (s), 770 (m) cm^{-1}.
HRMS (ESI-TOF): *m/z* calcd for $C_{15}H_{19}O_6$: 295.1176 $[M+H]^+$; found: 295.1178.
Elemental analysis calcd (%) for $C_{15}H_{18}O_6$: C 61.22, H 6.16; found: C 61.22, H 6.22.

Dimethyl 2-(2,6-dimethoxyphenyl)cyclopropane-1,1-dicarboxylate (1k) was synthesized according to the **GP2** from **S1k** (10.07 g, 35.9 mmol), affording **1k** as an orange-yellow solid (7.94 g, 75%); R_f = 0.23 (PE/EtOAc, 10:1); m.p. = 76–78 °C.

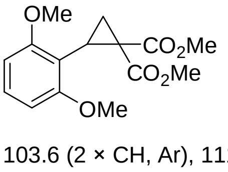

¹H NMR (500 MHz, $CDCl_3$): δ = 1.81 (dd, ^2J = 4.8 Hz, ^3J = 9.6 Hz, 1H, CH_2), 2.40 (dd, ^2J = 4.8 Hz, ^3J = 8.7 Hz, 1H, CH_2), 2.91 (dd, ^3J = 9.6 Hz, ^3J = 8.7 Hz, 1H, CH), 3.36 (s, 3H, CH_3O), 3.74 (s, 6H, 2 × CH_3O), 3.76 (s, 3H, CH_3O), 6.46 (d, ^3J = 8.4 Hz, 2H, Ar), 7.11 (t, ^3J = 8.4 Hz, 1H, Ar).
¹³C NMR (126 MHz, $CDCl_3$): δ = 22.6 (CH), 25.7 (CH_2), 34.9 (C), 51.7 (CH_3O), 52.5 (CH_3O), 55.6 (2 × CH_3O), 103.6 (2 × CH, Ar), 111.4 (C, Ar), 128.5 (CH, Ar), 159.7 (2 × C, Ar), 168.2 (CO_2Me), 170.9 (CO_2Me).
IR (KBr): ṽ = 3441 (w), 3108 (w), 2998 (m), 2949 (m), 2839 (m), 2536 (w), 1732 (s, C=O), 1715 (s, C=O), 1595 (s), 1475 (s), 1434 (s), 1379 (m), 1335 (s), 1279 (s), 1255 (s), 1218 (s), 1116 (s), 1034 (m), 977 (m), 850 (w), 765 (m) cm^{-1}.
HRMS (ESI-TOF): *m/z* calcd for $C_{15}H_{19}O_6$: 295.1176 $[M+H]^+$; found: 295.1182.

Dimethyl 2-(4-dimethylamino-2-nitrophenyl)cyclopropane-1,1-dicarboxylate (1n) was synthesized according to the **GP2** (reverse addition) from **S1n** (2.00 g, 4.69 mmol), affording **1n** as a yellow-orange solid (1.34 g, 64%); R_f = 0.31 (PE/EtOAc, 3:1); m.p. = 90–91 °C.

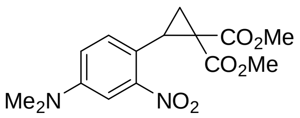

¹H NMR (500 MHz, $CDCl_3$): δ = 1.76 (dd, ^2J = 5.1 Hz, ^3J = 9.0 Hz, 1H, CH_2), 2.03 (dd, ^2J = 5.1 Hz, ^3J = 8.2 Hz, 1H, CH_2), 2.96 (s, 6H, 2 × CH_3N), 3.36 (s, 3H, CH_3O), 3.57 (dd, ^3J = 9.0 Hz, ^3J = 8.2 Hz, 1H, CH), 3.75 (s, 3H, CH_3O), 6.76 (dd, ^3J = 8.7 Hz, ^4J = 2.6 Hz, 1H, Ar), 7.09 (d, ^3J = 8.7 Hz, 1H, Ar), 7.24 (d, ^4J = 2.6 Hz, 1H, Ar).
¹³C NMR (126 MHz, $CDCl_3$): δ = 19.6 (CH_2), 30.7 (CH), 35.6 (C), 40.1 (2 × CH_3), 52.4 (CH_3O), 52.8 (CH_3O), 107.5 (CH, Ar), 115.8 (CH, Ar), 116.4 (C, Ar), 131.6 (CH, Ar), 150.0 (C, Ar), 150.7 (C, Ar), 167.4 (CO_2Me), 169.9 (CO_2Me).
IR (KBr): ṽ = 2955 (m), 1729 (vs, C=O), 1625 (m), 1534 (vs), 1435 (s), 1372 (s), 1350 (s), 1288 (s), 1205 (m), 1174 (m), 1132 (s), 1067 (m), 1017 (w), 975 (w), 920 (m), 876 (m), 822 (m), 797 (m), 715 (w) cm^{-1}.
HRMS (ESI-TOF): *m/z* calcd for $C_{15}H_{19}N_2O_6$: 323.1238 $[M+H]^+$; found: 323.1229.

Dimethyl 2-[4′-methoxy-(1,1′-biphenyl)-4-yl]cyclopropane-1,1-dicarboxylate (1o) was synthesized according to the **GP2** from **S1o** (2.00 g, 6.13 mmol). The residue (*ca*. 1.5 g) obtained after the work-up procedure was dissolved in hot IPA (10 mL), cooled to r.t., and diluted with additional IPA (10 mL). Filtration, washing with IPA (2 × 10 mL), PE (3 × 10 mL), and drying on air afforded **1o** as a brown solid (587 mg, 28%); R_f = 0.59 (PE/EtOAc, 3:1); m.p. = 102–104 °C.

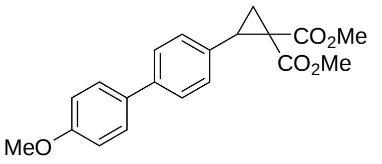

The second crop was isolated from the evaporated filtrate. A solid brown residue was dissolved in hot IPA (5 mL), placed in a freezer, and left overnight. Filtration, washing with chilled IPA (3 × 5 mL), PE (3 × 5 mL), and drying on air provided a grayish-brown solid (348 mg, 17%) of inferior purity.

^{1}H NMR (500 MHz, $CDCl_3$): δ = 1.77 (dd, ^{2}J = 5.2 Hz, ^{3}J = 9.2 Hz, 1H, CH_2), 2.23 (dd, ^{2}J = 5.2 Hz, ^{3}J = 7.9 Hz, 1H, CH_2), 3.25 (dd, ^{3}J = 9.2 Hz, ^{3}J = 7.9 Hz, 1H, CH), 3.40 (s, 3H, CH_3O), 3.80 (s, 3H, CH_3O), 3.85 (s, 3H, CH_3O), 6.96 (d, ^{3}J = 8.8 Hz, 2H, Ar), 7.23 (d, ^{3}J = 8.2 Hz, 2H, Ar), 7.47 (d, ^{3}J = 8.2 Hz, 2H, Ar), 7.51 (d, ^{3}J = 8.8 Hz, 2H, Ar).

^{13}C NMR (126 MHz, $CDCl_3$): δ = 19.3 (CH_2), 32.4 (CH), 37.4 (C), 52.4 (CH_3O), 52.9 (CH_3O), 55.4 (CH_3O), 114.3 (2 × CH, Ar), 126.4 (2 × CH, Ar), 128.0 (2 × CH, Ar), 128.9 (2 × CH, Ar), 133.0 (C, Ar), 133.1 (C, Ar), 139.8 (C, Ar), 159.3 (C, Ar), 167.1 (CO_2Me), 170.3 (CO_2Me).

IR (KBr): ṽ = 3432 (w), 3035 (w), 2954 (m), 2844 (w), 1920 (w), 1891 (w), 1740 (s, C=O), 1728 (s, C=O), 1607 (m), 1503 (s), 1376 (m), 1338 (s), 1287 (s), 1212 (s), 1138 (s), 1095 (s), 1066 (m), 1034 (s), 1013 (m), 941 (m), 885 (m), 825 (s), 781 (m) cm^{-1}.

HRMS (ESI-TOF): *m/z* calcd for $C_{20}H_{21}O_5$: 341.1384 $[M+H]^+$; found: 341.1379.

Dimethyl 2-(6-methoxynaphthalen-2-yl)cyclopropane-1,1-dicarboxylate (1p) was synthesized according to the **GP2** from **S1p** (5.00 g, 16.6 mmol). The residue (*ca*. 5 g) obtained after the work-up procedure was dissolved in warm MeOH (10 mL), cooled in an ice bath, rubbed with a spatula, placed in a freezer, and left overnight. Filtration, washing with chilled MeOH (3 × 10 mL), PE (3 × 15 mL), and drying on air afforded **1p** as a light-yellow solid (2.26 g, 43%); R_f = 0.53 (PE/EtOAc, 3:1); m.p. = 108–110 °C.

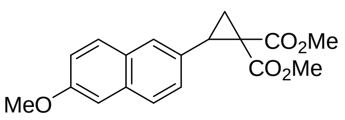

The second crop was isolated from the evaporated filtrate in the same manner as an off-white solid (267 mg, 5%) of inferior purity.

^{1}H NMR (500 MHz, $CDCl_3$): δ = 1.81 (dd, ^{2}J = 5.2 Hz, ^{3}J = 9.2 Hz, 1H, CH_2), 2.31 (dd, ^{2}J = 5.2 Hz, ^{3}J = 7.9 Hz, 1H, CH_2), 3.30 (s, 3H, CH_3O), 3.35 (dd, ^{3}J = 9.2 Hz, ^{3}J = 7.9 Hz, 1H, CH), 3.81 (s, 3H, CH_3O), 3.91 (s, 3H, CH_3O), 7.09 (d, ^{4}J = 2.5 Hz, 1H, Ar), 7.13 (dd, ^{3}J = 9.0 Hz, ^{4}J = 2.5 Hz, 1H, Ar), 7.29 (dd, ^{3}J = 8.5 Hz, ^{4}J = 1.8 Hz, 1H, Ar), 7.56 (br. s, 1H, Ar), 7.64 (d, ^{3}J = 8.5 Hz, 1H, Ar), 7.67 (d, ^{3}J = 9.0 Hz, 1H, Ar).

^{13}C NMR (126 MHz, $CDCl_3$): δ = 19.3 (CH_2), 32.8 (CH), 37.4 (C), 52.2 (CH_3O), 52.8 (CH_3O), 55.3 (CH_3O), 105.6 (CH, Ar), 119.0 (CH, Ar), 126.7 (CH, Ar), 127.0 (CH, Ar), 127.2 (CH, Ar), 128.6 (C, Ar), 129.3 (CH, Ar), 129.7 (C, Ar), 133.9 (C, Ar), 157.8 (C, Ar), 167.1 (CO_2Me), 170.3 (CO_2Me).

IR (KBr): ṽ = 3455 (w), 2908 (w), 2041 (w), 1727 (s, C=O), 1635 (m), 1611 (m), 1493 (m), 1438 (s), 1370 (m), 1317 (s), 1296 (s), 1266 (s), 1219 (s), 1164 (s), 1136 (s), 1026 (m), 1000 (m), 977 (w), 939 (m), 906 (m), 886 (m), 861 (s), 822 (m), 765 (w) cm^{-1}.

HRMS (ESI-TOF): *m/z* calcd for $C_{18}H_{19}O_5$: 315.1227 $[M+H]^+$; found: 315.1221.

Dimethyl 2-(ferrocenyl)cyclopropane-1,1-dicarboxylate (1x)[S18] was synthesized according to the **GP2** from **S1x** (5.00 g, 15.2 mmol). Very dark orange viscous oil (*ca*. 5.4 g) obtained after the work-up procedure was dissolved in hot MTBE (*ca*. 30 mL), decanted from an undissolved residue, cooled to r.t., placed in a freezer, and left overnight. Rubbing with a spatula, filtration, washing with PE (3 × 10 mL), and drying on air afforded **1x** as a dark off-orange solid (2.14 g, 41%) with dark inclusions. This solid was dissolved in hot MTBE (2 × 10 mL), immediately filtered from an undissolved residue through cotton wool, treated with PE (20 mL), rubbed with a spatula, placed in a freezer, and left overnight. Filtration (without washing) and drying on air provided **1x** as a dark orange-yellow voluminous solid (1.53 g, 29%); R_f = 0.63 (PE/EtOAc, 3:1); m.p. = 102–104 °C (lit. = 89–91 °C).[S18] Spectral data are well consistent with the published ones.[S18]

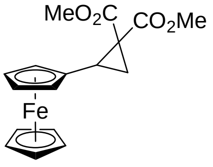

^{1}H NMR (500 MHz, $CDCl_3$): δ = 1.72 (dd, ^{2}J = 5.0 Hz, ^{3}J = 9.3 Hz, 1H, CH_2), 1.84 (dd, ^{2}J = 5.0 Hz, ^{3}J = 7.9 Hz, 1H, CH_2), 2.90 (dd, ^{3}J = 9.3 Hz, ^{3}J = 7.9 Hz, 1H, CH), 3.45 (s, 3H, CH_3O), 3.74 (s, 3H, CH_3O), 3.90–3.93 (m, 1H, Cp_2Fe), 4.02–4.04 (m, 1H, Cp_2Fe), 4.08–4.11 (m, 1H, Cp_2Fe), 4.16 (br. s, 5H, Cp_2Fe), 4.24–4.27 (m, 1H, Cp_2Fe).

^{13}C NMR (126 MHz, $CDCl_3$): δ = 20.3 (CH_2), 29.5 (CH), 38.4 (C), 52.3 (CH_3O), 52.7 (CH_3O), 66.2 (CH, Cp_2Fe), 67.4 (CH, Cp_2Fe), 68.5 (CH, Cp_2Fe), 68.8 (5 × CH, Cp_2Fe), 69.5 (CH, Cp_2Fe), 81.8 (C, Cp_2Fe), 167.3 (CO_2Me), 170.2 (CO_2Me).

HRMS (ESI-TOF): *m/z* calcd for $C_{17}H_{18}FeO_4$: 342.0555 $[M]^+$; found: 342.0543.

Dimethyl 2-[(*E*)-2-(4-fluorophenyl)ethenyl]cyclopropane-1,1-dicarboxylate (1ab) was synthesized according to the **GP2** from **S1ab** (5.00 g, 18.9 mmol). The crude product was isolated after the work-up procedure as a dark-orange turbid mobile oil (4.96 g). Flash chromatography (PE/EtOAc, 10:1) afforded **1ab** as a bright yellow oil (3.24 g, 62%), which solidified into a cream solid upon refrigeration; R_f = 0.24 (PE/EtOAc, 10:1); m.p. = 46–48 °C.

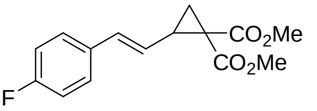

^{1}H NMR (500 MHz, $CDCl_3$): δ = 1.69 (dd, ^{2}J = 4.9 Hz, ^{3}J = 9.0 Hz, 1H, CH_2), 1.83 (dd, ^{2}J = 4.9 Hz, ^{3}J = 7.6 Hz, 1H, CH_2), 2.69–2.76 (m, 1H, CH), 3.73 (s, 3H, CH_3O), 3.76 (s, 3H, CH_3O), 5.73 (dd, ^{3}J = 15.8 Hz, ^{3}J = 8.7 Hz, 1H, CH=), 6.60 (d, ^{3}J = 15.8 Hz, 1H, CH=), 6.95–7.00 (m, 2H, Ar), 7.26 (dd, ^{3}J = 8.7 Hz, $^{4}J_{HF}$ = 5.4 Hz, 2H, Ar).

^{13}C NMR (126 MHz, $CDCl_3$): δ = 21.2 (CH_2), 31.5 (CH), 36.0 (C), 52.7 (CH_3O), 52.8 (CH_3O), 115.5 (d, $^{2}J_{CF}$ = 22 Hz, 2 × CH, Ar), 124.3 (d, $^{6}J_{CF}$ = 2 Hz, CH=), 127.7 (d, $^{3}J_{CF}$ = 8 Hz, 2 × CH, Ar), 132.6 (CH=), 132.9 (d, $^{4}J_{CF}$ = 4 Hz, C, Ar), 162.3 (d, $^{1}J_{CF}$ = 247 Hz, C, Ar), 167.9 (CO_2Me), 169.9 (CO_2Me).

^{19}F NMR (470 MHz, $CDCl_3$): δ = –114.2 (ArF).

IR (KBr): ṽ = 3040 (m), 3005 (m), 2954 (m), 2848 (w), 1727 (vs, C=O), 1601 (m, C=C), 1509 (s), 1438 (m), 1377 (w), 1332 (m), 1283 (s), 1251 (m), 1226 (m), 1130 (m), 1062 (w), 965 (m), 857 (w), 822 (m), 779 (w), 704 (w) cm^{-1}.
HRMS (ESI-TOF): *m/z* calcd for $C_{15}H_{16}FO_4$: 279.1027 $[M+H]^+$; found: 279.1026.

4-(6,6-Dimethyl-4,8-dioxo-5,7-dioxaspiro[2.5]octan-1-yl)benzonitrile (1af) was synthesized according to the **GP2** (reverse addition) from **S1af**[S17] (3.00 g, 11.7 mmol). The crude product was isolated after the work-up procedure as a light-yellow solid (1.49 g, 47%), which was treated with Et_2O (10 mL) and rubbed with a spatula. Filtration, washing with Et_2O (2 × 10 mL), PE (4 × 5 mL), and drying on a rotary evaporator (80 °C, 5 mbar) until the constant weight was achieved, afforded **1af** as a cream solid (1.29 g, 41%); R_f = 0.24 (PE/EtOAc, 3:1); m.p. = 168–169 °C (dec.).

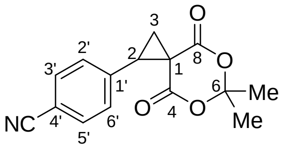

^{1}H NMR (500 MHz, $CDCl_3$): δ = 1.71 (s, 3H, CH_3), 1.75 (s, 3H, CH_3), 2.57 (dd, ^2J = 4.8 Hz, ^3J = 9.4 Hz, 1H, $C(3)H_2$), 2.65 (dd, ^2J = 4.8 Hz, ^3J = 9.2 Hz, 1H, $C(3)H_2$), 3.47 (dd, ^3J = 9.4 Hz, ^3J = 9.2 Hz, 1H, C(2)H), 7.44 (d, ^3J = 8.2 Hz, 2H, C(2')H, C(6')H, Ar), 7.64 (d, ^3J = 8.2 Hz, 2H, C(3')H, C(5')H, Ar).
^{13}C NMR (126 MHz, acetone-d_6): δ = 23.4 ($C(3)H_2$), 27.7 (CH_3), 28.2 (CH_3), 33.6 (C(1)), 42.4 (C(2)H), 105.8 ($C(6)Me_2$), 112.7 (C(1'), Ar), 119.1 (C≡N), 131.7 (2 × CH, C(2')H, C(6')H, Ar), 132.6 (2 × CH, C(3')H, C(5')H, Ar), 138.8 (C(4'), Ar), 164.2 (CO_2R), 167.6 (CO_2R).
IR (KBr): ṽ = 3413 (br. m), 3111 (m), 3063 (m), 3008 (m), 2987 (m), 2940 (m), 2225 (s, C≡N), 2057 (m), 1932 (w), 1764 (s, C=O), 1737 (s, C=O), 1672 (m), 1610 (s), 1506 (m), 1437 (s), 1395 (s), 1306 (s), 1199 (s), 1130 (m), 1048 (s), 1015 (s), 968 (s), 881 (m), 834 (s), 741 (m), 728 (m) cm^{-1}.
HRMS (ESI-TOF): *m/z* calcd for $C_{15}H_{17}N_2O_4$: 289.1183 $[M+NH_4]^+$; found: 289.1192.

General procedure (GP3) for D–A cyclopropanes 1 opening with thiocyanate ion

A 4 mL vial was charged with D–A cyclopropane **1** (*ca*. 1 mmol). Thiocyanate-based PIL preliminary melted with a heat-gun was added (method **A**: HMimNCS **3a**, *ca*. 8.6 equiv or method **A'**: $Et_3N{\cdot}HNCS$ **3b**, *ca*. 6.2 equiv; corresponding to *ca*. 1 M final concentration of **1** in PIL). The vial was tightly sealed, placed into the preheated oil bath (70 °C), and vigorously stirred for 1 h unless otherwise stated.[1] The vial was removed from the bath and allowed to cool to ambient temperature. The reaction mixture was dissolved in CH_2Cl_2 (20 mL) and washed successively with distilled water (20 mL),[2] tap water (20 mL), and saturated NaCl solution (20 mL). The organic fraction was dried with anhydrous Na_2SO_4, loaded on silica, concentrated under reduced pressure, and purified by flash chromatography (generally eluting with CH_2Cl_2/EtOAc mixtures) to afford pyrrolidine-2-thiones **2** as oils. Upon the concentration of combined chromatographical fractions, some products **2** could spontaneously froth into the bump trap. Thus, unevaporated EtOAc should always be present in the flask, and the vacuum should not be reduced lower than 100 mbar. In half of the cases, the crystallization occurred from the residuary EtOAc. In the remaining cases, the crude residues after evaporation of most of the eluent were treated with MTBE and rubbed with a spatula or irradiated with ultrasound to induce the crystallization/solidification. The resulting suspension was evaporated at a low vacuum (400–500 mbar, to prevent bumping) and then dried at a high vacuum at 60 °C for several hours to afford solid pyrrolidine-2-thiones **2**. Spectral data of known compound **2z** matched with the reported data.

Slow addition procedure (method B). D–A cyclopropane **1** was added in small portions to a preheated to 70 °C $[NCS^-]$-based PIL in approximately 35–40 min providing *ca.* 1 M final concentration of **1** in PIL. The reaction mixture was stirred at the same temperature for additional 20–25 min (total reaction time: 1 h). The work-up procedure was the same as in method **A** (**A'**).

Dimethyl 5-(3,4-dimethoxyphenyl)-2-thioxopyrrolidine-3,3-dicarboxylate (2a) was synthesized according to the **GP3** from **1a**[S19] (method **A**: 300 mg, 1.02 mmol, 1 h). Flash chromatography (CH_2Cl_2/EtOAc, 15:1) afforded **2a** as an off-white solid (290 mg, 81%); R_f = 0.18 (CH_2Cl_2/EtOAc, 15:1); m.p. = 166.5–168 °C.

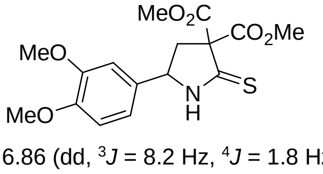

^{1}H NMR (500 MHz, $CDCl_3$): δ = 2.88 (dd, ^2J = 13.4 Hz, ^3J = 8.7 Hz, 1H, CH_2), 3.20 (dd, ^2J = 13.4 Hz, ^3J = 6.7 Hz, 1H, CH_2), 3.83 (s, 3H, CH_3O), 3.87 (s, 3H, CH_3O), 3.88 (s, 3H, CH_3O), 3.89 (s, 3H, CH_3O), 4.91 (dd, ^3J = 8.7 Hz, ^3J = 6.7 Hz, 1H, CH), 6.80 (d, ^4J = 1.8 Hz, 1H, Ar), 6.84 (d, ^3J = 8.2 Hz, 1H, Ar), 6.86 (dd, ^3J = 8.2 Hz, ^4J = 1.8 Hz, 1H, Ar), 8.06 (br. s, 1H, NH).
^{13}C NMR ($CDCl_3$): δ = 42.6 (CH_2), 53.6 (CH_3O), 53.8 (CH_3O), 56.05 (CH_3O), 56.13 (CH_3O), 62.9 (CH), 71.9 (C), 109.4 (CH, Ar), 111.5 (CH, Ar), 119.0 (CH, Ar), 130.6 (C, Ar), 149.5 (C, Ar), 149.6 (C, Ar), 167.6 (CO_2Me), 167.7 (CO_2Me), 197.1 (C=S).
IR (KBr): ṽ = 3456 (w), 3164 (br. s, NH), 3003 (s), 2948 (s), 2844 (m), 1749 (s, C=O), 1594 (s), 1515 (s), 1459 (s), 1380 (m), 1262 (s), 1192 (s), 1081 (s), 941 (m), 854 (m), 821 (m), 711 (m) cm^{-1}.
HRMS (ESI-TOF): *m/z* calcd for $C_{16}H_{20}NO_6S$: 354.1006 $[M+H]^+$; found: 354.1009.
Elemental analysis calcd (%) for $C_{16}H_{19}NO_6S$: C 54.38, H 5.42, N 3.96; found: C 54.15, H 5.62, N 4.08.

The gram-scale experiment was carried out under identical conditions (method **A**: 3.00 g, 10.2 mmol, 1 h). Purification by flash chromatography (two successive runs, CH_2Cl_2/EtOAc, 15:1 → 10:1) afforded **2a** as an off-white solid (3.21 g, 89%). Distilled water washings after the standard work-up procedure were subjected to the *representative regeneration procedure* (see below).

Performing the reaction in the regenerated HMimNCS under standard conditions from **1a** (2.50 g, 8.50 mmol) furnished **2a** as an off-white solid (2.66 g, 89%).

1) The TLC control of an aliquot dissolved in CH_2Cl_2 showed the complete consumption of the starting material.
2) The distilled water washings after each run were combined in order to be subjected to the regeneration procedure (see below).

Using doubly-regenerated HMimNCS under standard conditions, **1a** (2.00 g, 6.80 mmol) was transformed into **2a** in 87% yield (2.08 g). Using HMimNCS regenerated three times, **1a** (1.50 g, 5.10 mmol) produced the target **2a** in 87% yield (1.56 g). With HMimNCS regenerated four times, **1a** (1.00 g, 3.40 mmol) afforded **2a** in 86% yield (1.03 g).

Performing the reaction under method **A'** conditions resulted in an incomplete conversion of **1a** (*ca*. 60% conversion after 13.5 h at 70 °C).

Alternatively, pyrrolidine-2-thione **2a** was synthesized from **1a** using a two-step procedure including D–A cyclopropane ring opening with azide ion.[S20] The treatment of the formed dimethyl 2-[2-azido-2-(3,4-dimethoxyphenyl)ethyl]malonate with triphenylphosphine and CS_2 for 65 h at r.t.[S21] afforded **2a** as a dark-beige solid (142 mg, 60%). Therefore, the developed method allows for synthesizing pyrrolidine-2-thione **2** in one step in 1 h only and in much higher yield than employing a two-step sequence, which requires 70 h total.

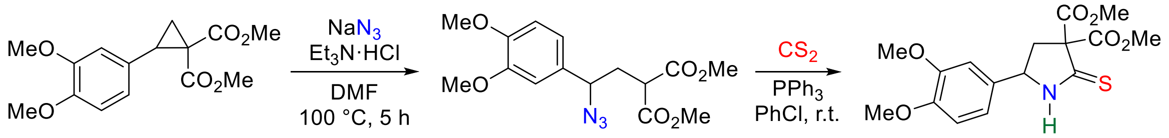

1a 79% (ref. S19) **2a**, 60%

Dimethyl 5-(4-methoxyphenyl)-2-thioxopyrrolidine-3,3-dicarboxylate (2b) was synthesized according to the **GP3** from **1b**[S22] (method **A**: 295 mg, 1.12 mmol, 1 h). Flash chromatography (CH_2Cl_2/EtOAc, 30:1) afforded **2b** as an off-white solid (312 mg, 86%); R_f = 0.24 (CH_2Cl_2/EtOAc, 30:1); m.p. = 173–175 °C.

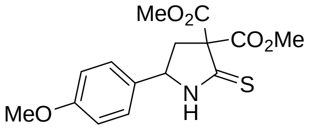

^{1}H NMR (500 MHz, $CDCl_3$): δ = 2.89 (dd, ^2J = 13.4 Hz, ^3J = 8.7 Hz, 1H, CH_2), 3.19 (dd, ^2J = 13.4 Hz, ^3J = 6.7 Hz, 1H, CH_2), 3.81 (s, 3H, CH_3O), 3.84 (s, 3H, CH_3O), 3.87 (s, 3H, CH_3O), 4.91 (dd, ^3J = 8.7 Hz, ^3J = 6.7 Hz, 1H, CH), 6.91 (d, ^3J = 8.7 Hz, 2H, Ar), 7.25 (d, ^3J = 8.7 Hz, 2H, Ar), 7.90 (br. s, 1H, NH).
^{1}H NMR (500 MHz, DMSO-d_6): δ = 2.62 (dd, ^2J = 13.3 Hz, ^3J = 7.9 Hz, 1H, CH_2), 3.08 (dd, ^2J = 13.3 Hz, ^3J = 6.9 Hz, 1H, CH_2), 3.67 (s, 3H, CH_3O), 3.749 (s, 3H, CH_3O), 3.752 (s, 3H, CH_3O), 4.95 (dd, ^3J = 7.9 Hz, ^3J = 6.9 Hz, 1H, CH), 6.96 (d, ^3J = 8.6 Hz, 2H, Ar), 7.22 (d, ^3J = 8.6 Hz, 2H, Ar), 11.07 (br. s, 1H, NH).
^{13}C NMR (126 MHz, DMSO-d_6): δ = 42.1 (CH_2), 53.0 (CH_3O), 53.2 (CH_3O), 55.2 (CH_3O), 61.5 (CH), 72.0 (C), 114.1 (2 × CH, Ar), 127.6 (2 × CH, Ar), 131.3 (C, Ar), 159.2 (C, Ar), 167.55 (CO_2Me), 167.59 (CO_2Me), 195.7 (C=S).
IR (KBr): ṽ = 3287 (br. s, N–H), 3010 (m), 2956 (m), 2837 (m), 2057 (w), 1889 (w), 1720 (s, C=O), 1613 (s), 1584 (m), 1503 (s), 1430 (s), 1328 (s), 1254 (s), 1100 (s), 827 (s), 778 (s) cm^{-1}.
HRMS (ESI-TOF): *m/z* calcd for $C_{15}H_{18}NO_5S$: 324.0900 $[M+H]^+$; found: 324.0896.
Elemental analysis calcd (%) for $C_{15}H_{17}NO_5S$: C 55.71, H 5.30, N 4.33; found: C 55.71, H 5.39, N 4.20.

Dimethyl 5-(3,4,5-trimethoxyphenyl)-2-thioxopyrrolidine-3,3-dicarboxylate (2c) was synthesized according to the **GP3** from **1c**[S23] (method **A**: 301 mg, 0.93 mmol, 1 h). Flash chromatography (CH_2Cl_2/EtOAc, 15:1 → 10:1) afforded **2c** as a beige solid (260 mg, 73%); R_f = 0.19 (CH_2Cl_2/EtOAc, 15:1); m.p. = 168–170 °C.

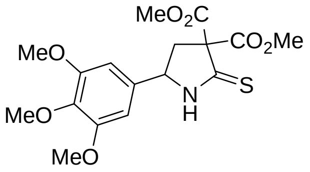

^{1}H NMR (500 MHz, $CDCl_3$): δ = 2.77 (dd, ^2J = 13.4 Hz, ^3J = 8.5 Hz, 1H, CH_2), 3.13 (dd, ^2J = 13.4 Hz, ^3J = 6.7 Hz, 1H, CH_2), 3.74 (s, 3H, CH_3O), 3.75 (s, 3H, CH_3O), 3.78 (s, 6H, 2 × CH_3O), 3.79 (s, 3H, CH_3O), 4.82 (dd, ^3J = 8.5 Hz, ^3J = 6.7 Hz, 1H, CH), 6.38 (s, 2H, Ar), 9.00 (br. s, 1H, NH).
^{13}C NMR (126 MHz, $CDCl_3$): δ = 42.3 (CH_2), 53.4 (CH_3O), 53.6 (CH_3O), 56.1 (2 × CH_3O), 60.7 (CH_3O), 63.2 (CH), 71.8 (C), 103.2 (2 × CH, Ar), 134.0 (C, Ar), 137.7 (C, Ar), 153.4 (2 × C, Ar), 167.6 (2 × CO_2Me), 196.9 (C=S).
IR (KBr): ṽ = 3302 (s, NH), 2990 (m), 2956 (s), 2841 (m), 1752 (s, C=O), 1730 (s, C=O), 1598 (s), 1512 (s), 1465 (s), 1367 (m), 1335 (m), 1279 (s), 1243 (s), 1205 (s), 1126 (s), 1049 (m), 998 (s), 922 (m), 883 (w), 854 (m), 832 (m), 780 (m), 738 (m) cm^{-1}.
HRMS (ESI-TOF): *m/z* calcd for $C_{17}H_{22}NO_7S$: 384.1111 $[M+H]^+$; found: 384.1108.

Dimethyl 5-(benzo[*d*][1,3]dioxol-5-yl)-2-thioxopyrrolidine-3,3-dicarboxylate (2d) was synthesized according to the **GP3** from **1d**[S24] (method **A**: 302 mg, 1.09 mmol, 1 h). Flash chromatography (CH_2Cl_2/EtOAc, 100:1 → 50:1) afforded **2d** (the crystallization occurred straight from the residual CH_2Cl_2 without any additional EtOAc) as an off-white solid (290 mg, 79%); R_f = 0.20 (CH_2Cl_2/EtOAc, 50:1); m.p. = 181–183 °C.

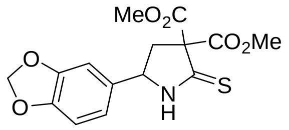

^{1}H NMR (500 MHz, DMSO-d_6): δ = 2.60 (dd, ^2J = 13.3 Hz, ^3J = 7.7 Hz, 1H, CH_2), 3.09 (dd, ^2J = 13.3 Hz, ^3J = 7.0 Hz, 1H, CH_2), 3.67 (s, 3H, CH_3O), 3.74 (s, 3H, CH_3O), 4.94 (dd, ^3J = 7.7 Hz, ^3J = 7.0 Hz, 1H, CH), 6.03 (d, ^2J = 3.0 Hz, 2H, OCH_2O), 6.78 (dd, ^3J = 8.0 Hz, ^4J = 1.6 Hz, 1H, Ar), 6.83 (d, ^4J = 1.6 Hz, 1H, Ar), 6.92 (d, ^3J = 8.0 Hz, 1H, Ar), 11.07 (br. s, 1H, NH).
^{13}C NMR (126 MHz, DMSO-d_6): δ = 42.0 (CH_2), 53.0 (CH_3O), 53.2 (CH_3O), 61.7 (CH), 71.9 (C), 101.2 (OCH_2O), 106.6 (CH, Ar), 108.3 (CH, Ar), 119.7 (CH, Ar), 133.3 (C, Ar), 147.1 (C, Ar), 147.6 (C, Ar), 167.5 (CO_2Me), 167.6 (CO_2Me), 195.8 (C=S).
IR (KBr): ṽ = 3284 (m, NH), 2974 (w), 2950 (w), 2894 (w), 1747 (s, C=O), 1723 (s, C=O), 1609 (w), 1493 (s), 1452 (m), 1302 (m), 1260 (s), 1238 (s), 1177 (m), 1083 (m), 1044 (s), 932 (m), 875 (w), 834 (m), 748 (m), 700 (m) cm^{-1}.
HRMS (ESI-TOF): *m/z* calcd for $C_{15}H_{16}NO_6S$: 338.0693 $[M+H]^+$; found: 338.0693.

Dimethyl 5-(2,3-dihydrobenzo[*b*][1,4]dioxin-6-yl)-2-thioxopyrrolidine-3,3-dicarboxylate (2e) was synthesized according to the **GP3** from **1e**[S19] (method **A**: 301 mg, 1.03 mmol, 1 h). Flash chromatography (CH_2Cl_2/EtOAc, 30:1 → 20:1) afforded **2e** (the crystallization occurred straight from the residual CH_2Cl_2 without any additional EtOAc) as a pale-yellow solid (279 mg, 77%); R_f = 0.22 (CH_2Cl_2/EtOAc, 30:1); m.p. = 200 °C (dec.).

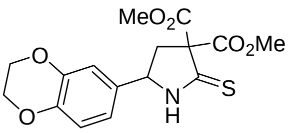

^{1}H NMR (500 MHz, DMSO-d$_6$): δ = 2.61 (dd, ^2J = 13.3 Hz, ^3J = 7.8 Hz, 1H, CH_2), 3.09 (dd, ^2J = 13.3 Hz, ^3J = 7.0 Hz, 1H, CH_2), 3.68 (s, 3H, CH_3O), 3.75 (s, 3H, CH_3O), 4.23 (br. s, 4H, $O(CH_2)_2O$), 4.91 (dd, ^3J = 7.8 Hz, ^3J = 7.0 Hz, 1H, CH), 6.76 (dd, ^3J = 8.3 Hz, ^4J = 2.0 Hz, 1H, Ar), 6.79 (d, ^4J = 2.0 Hz, 1H, Ar), 6.87 (d, ^3J = 8.3 Hz, 1H, Ar), 11.06 (br. s, 1H, NH).
^{13}C NMR (126 MHz, DMSO-d$_6$): δ = 42.0 (CH_2), 53.0 (CH_3O), 53.2 (CH_3O), 61.4 (CH), 64.06 (CH_2O), 64.10 (CH_2O), 71.9 (C), 115.1 (CH, Ar), 117.3 (CH, Ar), 119.0 (CH, Ar), 132.5 (C, Ar), 143.2 (C, Ar), 143.3 (C, Ar), 167.5 (CO_2Me), 167.6 (CO_2Me), 195.7 (C=S).
IR (KBr): ṽ = 3291 (m, NH), 2953 (w), 2452 (w), 1748 (m), 1721 (s, C=O), 1591 (w), 1509 (s), 1459 (m), 1434 (m), 1289 (s), 1243 (m), 1177 (m), 1099 (w), 1064 (w), 1041 (w), 1014 (w), 886 (m), 781 (w) cm^{-1}.
HRMS (ESI-TOF): *m/z* calcd for $C_{16}H_{18}NO_6S$: 352.0849 [M+H]$^+$; found: 352.0848.

Dimethyl 5-(2,4-dimethoxyphenyl)-2-thioxopyrrolidine-3,3-dicarboxylate (2f) was synthesized according to the **GP3** from **1f** (method **A**: 301 mg, 1.02 mmol, 1 h). Flash chromatography (CH_2Cl_2/EtOAc, 40:1) afforded **2f** as a dark-orange oil (336 mg) which upon treatment with MTBE provided an off-white solid (265 mg, 73%); R_f = 0.23 (CH_2Cl_2/EtOAc, 40:1); m.p. = 137–138 °C.

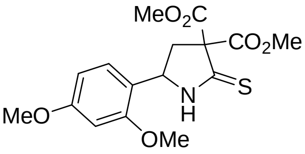

^{1}H NMR (500 MHz, $CDCl_3$): δ = 2.92 (dd, ^2J = 13.1 Hz, ^3J = 7.6 Hz, 1H, CH_2), 3.16 (dd, ^2J = 13.1 Hz, ^3J = 7.0 Hz, 1H, CH_2), 3.74 (s, 3H, CH_3O), 3.76 (s, 3H, CH_3O), 3.78 (s, 3H, CH_3O), 3.82 (s, 3H, CH_3O), 5.15 (dd, ^3J = 7.6 Hz, ^3J = 7.0 Hz, 1H, CH), 6.42 (br. s, 1H, Ar), 6.43 (dd, ^3J = 7.9 Hz, ^4J = 2.4 Hz, 1H, Ar), 7.10 (d, ^3J = 7.9 Hz, 1H, Ar), 8.63 (br. s, 1H, NH).
^{13}C NMR (126 MHz, $CDCl_3$): δ = 39.7 (CH_2), 53.4 (CH_3O), 53.6 (CH_3O), 55.4 (CH_3O), 55.5 (CH_3O), 57.4 (CH), 71.6 (C), 98.8 (CH, Ar), 104.4 (CH, Ar), 118.2 (C, Ar), 127.0 (CH, Ar), 157.9 (C, Ar), 161.1 (C, Ar), 167.75 (CO_2Me), 167.83 (CO_2Me), 196.9 (C=S).
IR (KBr): ṽ = 3114 (br. m, NH), 2964 (m), 2839 (m), 2052 (w), 1743 (s, C=O), 1728 (s, C=O), 1616 (s), 1586 (m), 1511 (s), 1437 (m), 1384 (w), 1266 (s), 1211 (s), 1161 (s), 1080 (m), 1045 (s), 945 (m), 855 (m), 798 (m) cm^{-1}.
HRMS (ESI-TOF): *m/z* calcd for $C_{16}H_{20}NO_6S$: 354.1006 [M+H]$^+$; found: 354.1001.

Dimethyl 5-(2,3,4-trimethoxyphenyl)-2-thioxopyrrolidine-3,3-dicarboxylate (2g) was synthesized according to the **GP3** from **1g**[S25] (method **A**: 303 mg, 0.93 mmol, 1 h). Flash chromatography (CH_2Cl_2/EtOAc, 20:1) afforded **2g** as a pale-yellow oil, which upon treatment with MTBE provided a pale-gray solid (281 mg, 78%); R_f = 0.22 (CH_2Cl_2/EtOAc, 20:1); m.p. = 119.5–121 °C.

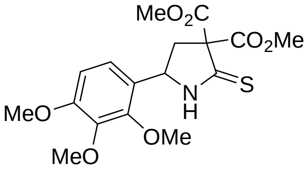

^{1}H NMR (500 MHz, $CDCl_3$): δ = 2.96 (dd, ^2J = 13.2 Hz, ^3J = 8.2 Hz, 1H, CH_2), 3.22 (dd, ^2J = 13.2 Hz, ^3J = 6.8 Hz, 1H, CH_2), 3.82 (s, 3H, CH_3O), 3.85 (s, 3H, CH_3O), 3.86 (s, 3H, CH_3O), 3.88 (s, 3H, CH_3O), 3.94 (s, 3H, CH_3O), 5.17 (dd, ^3J = 8.2 Hz, ^3J = 6.8 Hz, 1H, CH), 6.66 (d, ^3J = 8.7 Hz, 1H, Ar), 6.93 (d, ^3J = 8.7 Hz, 1H, Ar), 8.08 (br. s, 1H, NH).
^{13}C NMR (126 MHz, $CDCl_3$): δ = 40.7 (CH_2), 53.5 (CH_3O), 53.7 (CH_3O), 56.0 (CH_3O), 57.8 (CH), 60.8 (CH_3O), 61.2 (CH_3O), 71.7 (C), 107.2 (CH, Ar), 120.9 (CH, Ar), 123.5 (C, Ar), 142.0 (C, Ar), 151.3 (C, Ar), 154.1 (C, Ar), 167.7 (CO_2Me), 167.8 (CO_2Me), 196.8 (C=S).
IR (KBr): ṽ = 3271 (s, NH), 2940 (m), 2836 (m), 1747 (s, C=O), 1718 (s, C=O), 1602 (m), 1496 (s), 1469 (s), 1283 (s), 1201 (m), 1175 (m), 1093 (s), 1057 (m), 1031 (m), 1011 (m), 977 (w), 956 (w), 785 (m), 747 (m) cm^{-1}
HRMS (ESI-TOF): *m/z* calcd for $C_{17}H_{22}NO_7S$: 384.1111 [M+H]$^+$; found: 384.1103.

Dimethyl 5-(2-methoxyphenyl)-2-thioxopyrrolidine-3,3-dicarboxylate (2h) was synthesized according to the **GP3** from **1h**[S26] (method **A**: 303 mg, 1.15 mmol, 1 h). Flash chromatography (CH_2Cl_2/EtOAc, 50:1) afforded **2h** as a yellow viscous oil, which upon prolonged drying on a rotary evaporator (50 °C, 2 mbar) provided a pale-yellow solid (290 mg, 78%); R_f = 0.24 (CH_2Cl_2/EtOAc, 50:1); m.p. = 158–160 °C.

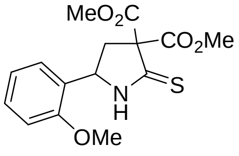

^{1}H NMR (500 MHz, $CDCl_3$): δ = 3.01 (dd, ^2J = 13.2 Hz, ^3J = 7.8 Hz, 1H, CH_2), 3.27 (dd, ^2J = 13.2 Hz, ^3J = 7.0 Hz, 1H, CH_2), 3.79 (s, 3H, CH_3O), 3.86 (s, 3H, CH_3O), 3.88 (s, 3H, CH_3O), 5.26 (dd, ^3J = 7.8 Hz, ^3J = 7.0 Hz, 1H, CH), 6.91 (d, ^3J = 8.2 Hz, 1H, Ar), 6.96–7.00 (m, 1H, Ar), 7.24 (dd, ^3J = 7.6 Hz, ^4J = 0.9 Hz, 1H, Ar), 7.30–7.34 (m, 1H, Ar), 8.11 (br. s, 1H, NH).
^{1}H NMR (500 MHz, DMSO-d$_6$): δ = 2.61 (dd, ^2J = 13.2 Hz, ^3J = 6.3 Hz, 1H, CH_2), 3.14 (dd, ^2J = 13.2 Hz, ^3J = 7.6 Hz, 1H, CH_2), 3.59 (s, 3H, CH_3O), 3.74 (s, 3H, CH_3O), 3.80 (s, 3H, CH_3O), 5.16 (dd, ^3J = 7.6 Hz, ^3J = 6.3 Hz, 1H, CH), 6.95–7.00 (m, 1H, Ar), 7.04 (d, ^3J = 8.2 Hz, 1H, Ar), 7.12 (br. d, ^3J = 7.3 Hz, 1H, Ar), 7.29–7.35 (m, 1H, Ar), 11.05 (s, 1H, NH).
^{13}C NMR (126 MHz, $CDCl_3$/DMSO-d$_6$, 2:1): δ = 39.7 (CH_2), 52.3 (CH_3O), 52.7 (CH_3O), 54.7 (CH_3O), 56.9 (CH), 71.2 (C), 109.9 (CH, Ar), 119.8 (CH, Ar), 125.3 (CH, Ar), 126.5 (C, Ar), 128.6 (CH, Ar), 155.7 (C, Ar), 167.2 (CO_2Me), 167.3 (CO_2Me), 196.4 (C=S).
^{13}C NMR (126 MHz, DMSO-d$_6$): δ = 40.1 (CH_2), 52.9 (CH_3O), 53.2 (CH_3O), 55.6 (CH_3O), 57.2 (CH), 71.7 (C), 111.1 (CH, Ar), 120.3 (CH, Ar), 126.0 (CH, Ar), 127.0 (C, Ar), 129.3 (CH, Ar), 156.2 (C, Ar), 167.6 (CO_2Me), 167.7 (CO_2Me), 196.1 (C=S).
IR (KBr): ṽ = 3296 (s, NH), 3003 (m), 2956 (m), 2837 (w), 1757 (s, C=O), 1720 (m, C=O), 1599 (w), 1515 (s), 1496 (s), 1437 (m), 1374 (w), 1283 (s), 1256 (s), 1194 (m), 1046 (m), 967 (w), 926 (w), 772 (m), 726 (m) cm^{-1}.
HRMS (ESI-TOF): *m/z* calcd for $C_{15}H_{18}NO_5S$: 324.0900 [M+H]$^+$; found: 324.0904.
Elemental analysis calcd (%) for $C_{15}H_{17}NO_5S$: C 55.72, H 5.30, N 4.33; found: C 55.95, H 5.61, N 4.09.

Dimethyl 5-[2-(ethoxymethoxy)phenyl]-2-thioxopyrrolidine-3,3-dicarboxylate (2i) was synthesized according to the **GP3** from **1i**[S27] (method **A**: 302 mg, 0.98 mmol, 2 h). Flash chromatography (PE/EtOAc, 3:1) afforded **2i** as a pale-yellow oil, which upon storage overnight provided a pale-yellow solid (209 mg, 58%); R_f = 0.19 (PE/EtOAc, 3:1); m.p. = 123–124 °C.

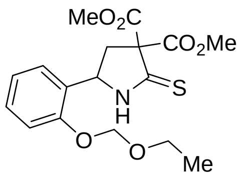

Performing the reaction under method **A'** conditions resulted in an insignificant conversion of **1i** (97% recovery after 3 h at 70 °C).

^{1}H NMR (500 MHz, $CDCl_3$): δ = 1.23 (t, ^3J = 7.1 Hz, 3H, CH_3CH_2O), 3.01 (dd, ^2J = 13.3 Hz, ^3J = 7.6 Hz, 1H, CH_2), 3.25 (dd, ^2J = 13.3 Hz, ^3J = 7.0 Hz, 1H, CH_2), 3.72 (q, ^3J = 7.1 Hz, 2H, MeCH_2O), 3.78 (s, 3H, CH_3O), 3.87 (s, 3H, CH_3O), 5.25–5.30 (m, 3H, CH + OCH_2O), 7.00–7.05 (m, 1H, Ar), 7.17 (br. d, ^3J = 8.3 Hz, 1H, Ar), 7.24–7.32 (m, 2H, Ar), 8.13 (br. s, 1H, NH).

^{13}C NMR (126 MHz, $CDCl_3$): δ = 15.1 (CH_3CH_2O), 40.0 (CH_2), 53.4 (CH_3O), 53.7 (CH_3O), 57.9 (CH), 64.7 (MeCH_2O), 71.6 (C), 93.2 (OCH_2O), 114.3 (CH, Ar), 121.9 (CH, Ar), 126.2 (CH, Ar), 126.7 (C, Ar), 129.8 (CH, Ar), 154.6 (C, Ar), 167.7 (CO_2Me), 167.8 (CO_2Me), 197.3 (C=S).

IR (KBr): ṽ = 3474 (w), 3316 (m), 3150 (m), 2975 (m), 2912 (m), 1736 (s, C=O), 1603 (m), 1522 (s), 1417 (s), 1277 (s), 1043 (s), 992 (s), 931 (m), 758 (s) cm^{-1}.

HRMS (ESI-TOF): *m/z* calcd for $C_{17}H_{22}NO_6S$: 368.1162 $[M+H]^+$; found: 368.1166.

Elemental analysis calcd (%) for $C_{17}H_{21}NO_6S$: C 55.57, H 5.76, N 3.81; found: C 55.91, H 5.91, N 3.66.

Dimethyl 5-(2-hydroxyphenyl)-2-thioxopyrrolidine-3,3-dicarboxylate (2j) was synthesized according to the **GP3** from **1j**[S27] (method **A**: 257 mg, 1.03 mmol, 1 h). Flash chromatography (CH_2Cl_2/EtOAc, 20:1 → 15:1) afforded **2j** as a pale-orange oil (145 mg), which was dissolved in EtOAc (1 mL) and treated with PE (10 mL), resulting in a colorless oil. The obtained mixture was treated with CH_2Cl_2 (1 mL) and rubbed with a spatula. The concentration of the solvent on a rotary evaporator at r.t. furnished an off-gray semi-solid residue, which upon treatment with MTBE (1–2 mL), rubbing, the concentration of the solvent at r.t., and drying on a rotary evaporator at 60–70 °C provided a sticky brown solid (102 mg, 32%); R_f = 0.22 (CH_2Cl_2/EtOAc, 15:1); m.p. = 166–168 °C.

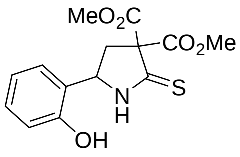

Performing the reaction under method **A'** conditions starting from **1j** (300 mg, 1.20 mmol) resulted in **2j** in similar chromatographic yield (182 mg, 49%). However, we failed to obtain an analytically pure product *via* additional chromatography or crystallization.

Using method **B**, in HMimNCS D–A cyclopropane **1j** (200 mg, 0.80 mmol) was transformed into a yellow viscous oil (101 mg), which was dissolved in MTBE (2 mL) and diluted with PE (2 mL). The resulting mixture containing a small amount of precipitated off-yellow oil was treated with liquid N_2 and rubbed with a spatula. The concentration of the solvent at r.t. and drying on a rotary evaporator at 60–70 °C provided a yellowish-white solid (89 mg, 36%).

^{1}H NMR (500 MHz, DMSO-d_6): δ = 2.64 (dd, ^2J = 13.1 Hz, ^3J = 6.7 Hz, 1H, CH_2), 3.11 (dd, ^2J = 13.1 Hz, ^3J = 7.4 Hz, 1H, CH_2), 3.59 (s, 3H, CH_3O), 3.74 (s, 3H, CH_3O), 5.13 (dd, ^3J = 7.4 Hz, ^3J = 6.7 Hz, 1H, CH), 6.77–6.88 (m, 2H, Ar), 7.04 (d, ^3J = 7.3 Hz, 1H, Ar), 7.10–7.17 (m, 1H, Ar), 9.80 (br. s, 1H, OH), 11.03 (br. s, 1H, NH).

^{13}C NMR (126 MHz, DMSO-d_6): δ = 40.3 (CH_2), 52.9 (CH_3O), 53.3 (CH_3O), 57.3 (CH), 71.8 (C), 115.3 (CH, Ar), 119.0 (CH, Ar), 125.4 (C, Ar), 126.2 (CH, Ar), 128.9 (CH, Ar), 154.4 (C, Ar), 167.7 (CO_2Me), 167.8 (CO_2Me), 196.0 (C=S).

IR (KBr): ṽ = 3503 (br. vs, OH), 3303 (br. vs, NH), 3010 (s), 2954 (s), 2840 (m), 1963 (w), 1929 (w), 1738 (s, C=O), 1721 (s, C=O), 1601 (m), 1510 (s), 1459 (s), 1432 (m), 1345 (m), 1289 (m), 1108 (m), 1088 (m), 1031 (m), 1013 (m), 932 (w), 881 (w), 849 (w), 811 (w), 783 (w), 762 (m), 701 (m) cm^{-1}.

HRMS (ESI-TOF): *m/z* calcd for $C_{14}H_{16}NO_5S$: 310.0744 $[M+H]^+$; found: 310.0746.

Elemental analysis calcd (%) for $C_{14}H_{15}NO_5S$: C 54.36, H 4.89, N 4.53; found: C 54.62, H 4.80, N 4.25.

Dimethyl 5-(2,6-dimethoxyphenyl)-2-thioxopyrrolidine-3,3-dicarboxylate (2k) was synthesized according to the **GP3** from **1k** (method **A**: 302 mg, 1.03 mmol, 1 h). Flash chromatography (CH_2Cl_2/EtOAc, 30:1) afforded **2k** as an orange oil, which was dissolved in EtOAc (10–15 mL). Slow evaporation at 50 mbar and drying on a rotary evaporator provided a yellowish-white solid (259 mg, 71%); R_f = 0.28 (CH_2Cl_2/EtOAc, 30:1); m.p. = 169–171 °C.

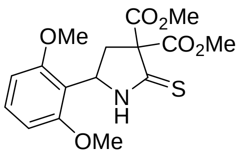

^{1}H NMR (500 MHz, $CDCl_3$): δ = 2.98 (dd, ^2J = 13.0 Hz, ^3J = 7.6 Hz, 1H, CH_2), 3.26 (dd, ^2J = 13.0 Hz, ^3J = 8.6 Hz, 1H, CH_2), 3.75 (s, 6H, 2 × CH_3O), 3.806 (s, 3H, CH_3O), 3.810 (s, 3H, CH_3O), 5.57 (dd, ^3J = 8.6 Hz, ^3J = 7.6 Hz, 1H, CH), 6.50 (d, ^3J = 8.4 Hz, 2H, Ar), 7.20 (t, ^3J = 8.4 Hz, 1H, Ar), 8.24 (br. s, 1H, NH).

^{13}C NMR (126 MHz, $CDCl_3$): δ = 38.0 (CH_2), 53.1 (CH_3O), 53.6 (CH_3O), 53.8 (CH), 55.8 (2 × CH_3O), 71.9 (C), 104.2 (2 × CH, Ar), 112.5 (C, Ar), 130.3 (CH, Ar), 158.8 (2 × C, Ar), 167.6 (CO_2Me), 168.4 (CO_2Me), 195.7 (C=S).

IR (KBr): ṽ = 3437 (br. w), 3121 (m), 3017 (m), 2951 (m), 2841 (m), 1759 (s, C=O), 1727 (s, C=O), 1596 (s), 1547 (s), 1477 (s), 1436 (m), 1377 (m), 1277 (s), 1255 (s), 1198 (s), 1172 (s), 1115 (s), 1055 (s), 969 (w), 823 (m), 810 (m), 786 (m), 735 (m) cm^{-1}.

HRMS (ESI-TOF): *m/z* calcd for $C_{16}H_{20}NO_6S$: 354.1006 $[M+H]^+$; found: 354.1002.

Dimethyl 2-thioxo-5-(2,4,6-trimethoxyphenyl)pyrrolidine-3,3-dicarboxylate (2l) was synthesized according to the **GP3** from **1l**[S19] (method **A**: 300 mg, 0.92 mmol, 1 h). Flash chromatography (CH_2Cl_2/EtOAc, 20:1) afforded **2l** as a pale-yellow solid (58 mg, 16%).

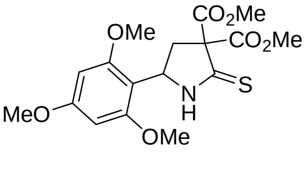

Performing the reaction under method **A'** conditions, **1l** (301 mg, 0.93 mmol) afforded **2l** as a pale-yellow solid (218 mg). The residue was treated with MTBE (*ca.* 2 mL) at 45 °C under ultrasound irradiation and chilled in a freezer; the yellowish MTBE layer was withdrawn with a Pasteur pipette

and discarded. The treatment with MTBE was repeated once more. Filtration, washing with PE, and drying on air provided **2l** as an off-white solid (159 mg, 45%); R_f = 0.17 (CH_2Cl_2/EtOAc, 20:1); m.p. = 169–171 °C.

Performing the reaction under method **B** conditions in $Et_3N·HNCS$, **1l** (300 mg, 0.92 mmol) produced **2l** as a yellowish-white solid of analytical purity directly after chromatography in 59% yield (206 mg).

^{1}H NMR (500 MHz, $CDCl_3$): δ = 2.98 (dd, ^2J = 13.1 Hz, ^3J = 7.4 Hz, 1H, CH_2), 3.32 (dd, ^2J = 13.1 Hz, ^3J = 8.8 Hz, 1H, CH_2), 3.78 (s, 6H, 2 × CH_3O), 3.81 (s, 3H, CH_3O), 3.86 (s, 6H, 2 × CH_3O), 5.52 (dd, ^3J = 8.8 Hz, ^3J = 7.4 Hz, 1H, CH), 6.10 (s, 2H, Ar), 7.67 (br. s, 1H, NH).

^{13}C NMR (126 MHz, $CDCl_3$): δ = 38.3 (CH_2), 53.3 (CH_3O), 53.7 (CH_3O), 53.8 (CH), 55.5 (CH_3O), 55.9 (2 × CH_3O), 72.0 (C), 90.9 (2 × CH, Ar), 105.0 (C, Ar), 159.7 (2 × C, Ar), 161.9 (C, Ar), 167.8 (CO_2Me), 168.6 (CO_2Me), 195.9 (C=S).

IR (KBr): ṽ = 3466 (w), 3432 (w), 3155 (br. m, NH), 3006 (m), 2950 (m), 2837 (m), 1737 (s, C=O), 1609 (s), 1593 (s), 1548 (s), 1461 (m), 1433 (m), 1378 (m), 1338 (m), 1271 (s), 1202 (s), 1124 (s), 1048 (s), 951 (m), 876 (w), 827 (m), 788 (m), 720 (w) cm^{-1}.

HRMS (ESI-TOF): *m/z* calcd for $C_{17}H_{22}NO_7S$: 384.1111 $[M+H]^+$; found: 384.1103.

Elemental analysis calcd (%) for $C_{17}H_{21}NO_7S$: C 53.25, H 5.52, N 3.65; found: C 53.00, H 5.57, N 3.55.

Dimethyl 5-[4-(dimethylamino)phenyl]-2-thioxopyrrolidine-3,3-dicarboxylate (2m) was synthesized according to the **GP3** from **1m**[S23] (method **A**: 301 mg, 1.09 mmol, 1 h). Flash chromatography (CH_2Cl_2/EtOAc, 30:1 → 20:1) afforded **2m** as earth-yellow flakes (47 mg, 13%).

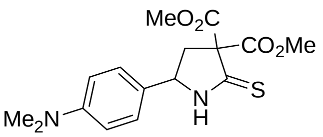

Performing the reaction under method **A'** conditions, **1m** (301 mg, 1.09 mmol) gave rise to **2m** as a pale-yellow solid (162 mg, 44%); m.p. = 177–179 °C (dec.). Distilled water washings after the standard work-up procedure were subjected to the *representative regeneration procedure* (see below).

Performing the reaction under method **B** conditions in $Et_3N·HNCS$, **1m** (200 mg, 0.72 mmol) afforded **2m** as a pale-yellow solid (135 mg, 56%) of analytical purity directly after chromatography; R_f = 0.26 (CH_2Cl_2/EtOAc, 20:1); m.p. = 185–187 °C (dec.).

In the regenerated $Et_3N·HNCS$ under method **B** conditions, compound **1m** (200 mg, 0.72 mmol) furnished **2m** of analytical purity directly after chromatography in the same yield (56%).

^{1}H NMR (500 MHz, $CDCl_3$): δ = 2.90 (dd, ^2J = 13.4 Hz, ^3J = 9.1 Hz, 1H, CH_2), 2.96 (s, 6H, $(CH_3)_2N$), 3.14 (dd, ^2J = 13.4 Hz, ^3J = 6.4 Hz, 1H, CH_2), 3.84 (s, 3H, CH_3O), 3.87 (s, 3H, CH_3O), 4.86 (dd, ^3J = 9.1 Hz, ^3J = 6.4 Hz, 1H, CH), 6.70 (d, ^3J = 8.9 Hz, 2H, Ar), 7.18 (d, ^3J = 8.9 Hz, 2H, Ar), 7.89 (br. s, 1H, NH).

^{13}C NMR (126 MHz, $CDCl_3$): δ = 40.4 ($(CH_3)_2N$), 42.7 (CH_2), 53.5 (CH_3O), 53.6 (CH_3O), 62.7 (CH), 72.0 (C), 112.6 (2 × CH, Ar), 125.1 (C, Ar), 127.5 (2 × CH, Ar), 150.9 (C, Ar), 167.7 (CO_2Me), 167.8 (CO_2Me), 196.7 (C=S).

IR (KBr): ṽ = 3320 (m, NH), 3010 (w), 2953 (m), 2892 (m), 2810 (m), 1890 (w), 1745 (s, C=O), 1720 (s, C=O), 1615 (m), 1524 (s), 1509 (s), 1442 (m), 1359 (m), 1291 (s), 1251 (m), 1190 (m), 1094 (m), 1040 (m), 1006 (m), 972 (w), 822 (m), 788 (m), 705 (m) cm^{-1}.

HRMS (ESI-TOF): *m/z* calcd for $C_{16}H_{21}N_2O_4S$: 337.1217 $[M+H]^+$; found: 337.1216.

Elemental analysis calcd (%) for $C_{16}H_{20}N_2O_4S$: C 57.13, H 5.99, N 8.33; found: C 56.98, H 6.07, N 8.13.

Colorless platelets appropriate for single-crystal X-ray analysis were achieved upon slow evaporation of **2m** solution in EtOAc on air.

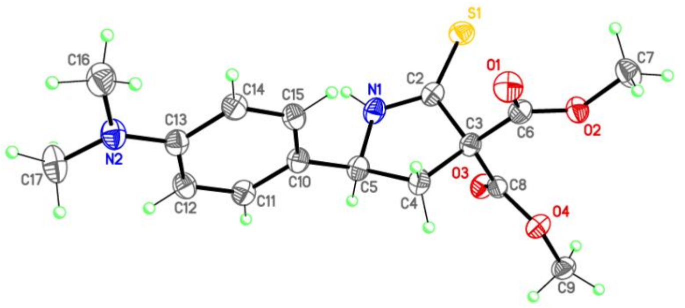

Figure S4. Structure of **2m** determined by single-crystal X-ray analysis (thermal ellipsoids are shown at 50% probability; CCDC 1995822).

Dimethyl 5-(4-dimethylamino-2-nitrophenyl)-2-thioxopyrrolidine-3,3-dicarboxylate (2n) was synthesized according to the **GP3** from **1n** (method **A**: 301 mg, 0.93 mmol, 1 h). Flash chromatography (CH_2Cl_2/EtOAc, 30:1) afforded **2n** (the crystallization occurred straight from the residual CH_2Cl_2 without any additional EtOAc) as sparkling orange crystals (276 mg, 78%); R_f = 0.23 (CH_2Cl_2/EtOAc, 30:1); m.p. = 186–188 °C.

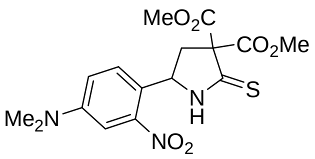

^{1}H NMR (500 MHz, $CDCl_3$): δ = 2.91 (dd, ^2J = 13.8 Hz, ^3J = 6.7 Hz, 1H, CH_2), 3.04 (s, 6H, $(CH_3)_2N$), 3.49 (dd, ^2J = 13.8 Hz, ^3J = 7.5 Hz, 1H, CH_2), 3.79 (s, 3H, CH_3O), 3.87 (s, 3H, CH_3O), 5.41 (dd, ^3J = 7.5 Hz, ^3J = 6.7 Hz, 1H, CH), 6.91 (dd, ^3J = 8.8 Hz, ^4J = 2.8 Hz, 1H, Ar), 7.23 (d, ^4J = 2.8 Hz, 1H, Ar), 7.40 (d, ^3J = 8.8 Hz, 1H, Ar), 8.01 (br. s, 1H, NH).

^{1}H NMR (500 MHz, DMSO-d_6, 46 °C): δ = 2.62 (dd, ^2J = 13.6 Hz, ^3J = 6.3 Hz, 1H, CH_2), 2.99 (s, 6H, $(CH_3)_2N$), 3.28 (dd, ^2J = 13.6 Hz, ^3J = 7.6 Hz, 1H, CH_2), 3.64 (s, 3H, CH_3O), 3.75 (s, 3H, CH_3O), 5.27 (dd, ^3J = 7.6 Hz, ^3J = 6.3 Hz, 1H, CH), 7.12 (dd, ^3J = 8.9 Hz, ^4J = 2.8 Hz, 1H, Ar), 7.20 (d, ^4J = 2.8 Hz, 1H, Ar), 7.23 (d, ^3J = 8.9 Hz, 1H, Ar), 10.97 (br. s, 1H, NH).

^{13}C NMR (126 MHz, DMSO-d$_6$): δ = 39.7 ((CH$_3$)$_2$N), 41.6 (CH$_2$), 53.1 (CH$_3$O), 53.3 (CH$_3$O), 58.0 (CH), 71.5 (C), 106.5 (CH, Ar), 116.9 (CH, Ar), 120.1 (C, Ar), 127.9 (CH, Ar), 148.6 (C, Ar), 150.1 (C, Ar), 167.5 (*C*O$_2$Me), 167.6 (*C*O$_2$Me), 196.2 (C=S).
IR (KBr): ṽ = 3268 (s, NH), 2952 (m), 1755 (s, C=O), 1720 (s, C=O), 1625 (m), 1536 (s), 1507 (s), 1450 (s), 1281 (s), 1239 (s), 1175 (m), 1097 (m), 1068 (m), 1043 (m), 879 (w), 827 (w), 785 (w), 758 (w), 734 (w) cm^{-1}.
HRMS (ESI-TOF): *m/z* calcd for $C_{16}H_{20}N_3O_6S$: 382.1067 [M+H]$^+$; found: 382.1061.

Dimethyl 5-[4′-methoxy-(1,1′-biphenyl)-4-yl]-2-thioxopyrrolidine-3,3-dicarboxylate (2o) was synthesized according to the **GP3** from **1o** (method **A**: 300 mg, 0.88 mmol, 4 h). Flash chromatography (CH$_2$Cl$_2$/EtOAc, 50:1) afforded **2o** as a dirty white solid (199 mg). The residue was grounded with a spatula, treated with boiling MTBE (5 mL), and cooled to r.t. Filtration, washing with MTBE (2 × 5 mL), and drying on air provided an off-white solid (89 mg, 25%). The second crop was isolated from the evaporated filtrate. The residue was treated with MTBE (2 mL), filtered, washed with MTBE (2 × 2 mL), and dried on air, providing an off-white solid (50 mg, 14%). The total yield of **2o** – 139 mg, *ca*. 40%. R_f = 0.25 (CH$_2$Cl$_2$/EtOAc, 50:1); m.p. = 159–161 °C.

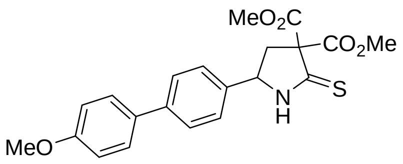

^{1}H NMR (500 MHz, CDCl$_3$): δ = 2.94 (dd, ^2J = 13.4 Hz, ^3J = 8.6 Hz, 1H, CH$_2$), 3.26 (dd, ^2J = 13.4 Hz, ^3J = 6.8 Hz, 1H, CH$_2$), 3.84 (s, 3H, CH$_3$O), 3.85 (s, 3H, CH$_3$O), 3.89 (s, 3H, CH$_3$O), 5.00 (dd, ^3J = 8.6 Hz, ^3J = 6.8 Hz, 1H, CH), 6.98 (d, ^3J = 8.8 Hz, 2H, Ar), 7.37 (d, ^3J = 8.2 Hz, 2H, Ar), 7.51 (d, ^3J = 8.8 Hz, 2H, Ar), 7.57 (d, ^3J = 8.2 Hz, 2H, Ar), 8.06 (br. s, 1H, NH).
^{13}C NMR (126 MHz, CDCl$_3$): δ = 42.7 (CH$_2$), 53.6 (CH$_3$O), 53.8 (CH$_3$O), 55.4 (CH$_3$O), 62.7 (CH), 71.9 (C), 114.4 (2 × CH, Ar), 126.9 (2 × CH, Ar), 127.3 (2 × CH, Ar), 128.2 (2 × CH, Ar), 132.7 (C, Ar), 136.5 (C, Ar), 141.5 (C, Ar), 159.5 (C, Ar), 167.6 (*C*O$_2$Me), 167.7 (*C*O$_2$Me), 197.4 (C=S).
IR (KBr): ṽ = 3417 (m), 3304 (w), 2958 (m), 2840 (w), 1741 (s, C=O), 1606 (m), 1481 (s), 1434 (s), 1287 (s), 1244 (s), 1092 (m), 1036 (m), 1012 (m), 927 (w), 821 (s), 767 (w), 745 (w) cm^{-1}.
HRMS (ESI-TOF): *m/z* calcd for $C_{21}H_{22}NO_5S$: 400.1213 [M+H]$^+$; found: 400.1209.
Elemental analysis calcd (%) for $C_{21}H_{21}NO_5S$: C 63.14, H 5.30, N 3.51; found: C 63.26, H 5.29, N 3.54.

Dimethyl 5-(6-methoxynaphthalen-2-yl)-2-thioxopyrrolidine-3,3-dicarboxylate (2p) was synthesized according to the **GP3** from **1p** (method **A**: 301 mg, 0.96 mmol, 1 h). Flash chromatography (CH$_2$Cl$_2$/EtOAc, 100:1 → 50:1) afforded **2p** (the crystallization occurred straight from the residual CH$_2$Cl$_2$ without any additional EtOAc) as a yellowish-white solid (261 mg, 73%); R_f = 0.31 (CH$_2$Cl$_2$/EtOAc, 50:1); m.p. = 166–167 °C.

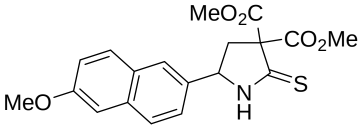

^{1}H NMR (500 MHz, CDCl$_3$): δ = 2.99 (dd, ^2J = 13.4 Hz, ^3J = 8.7 Hz, 1H, CH$_2$), 3.28 (dd, ^2J = 13.4 Hz, ^3J = 6.6 Hz, 1H, CH$_2$), 3.83 (s, 3H, CH$_3$O), 3.89 (s, 3H, CH$_3$O), 3.92 (s, 3H, CH$_3$O), 5.09 (dd, ^3J = 8.7 Hz, ^3J = 6.6 Hz, 1H, CH), 7.13 (d, ^4J = 2.5 Hz, 1H, Ar), 7.18 (dd, ^3J = 9.0 Hz, ^4J = 2.5 Hz, 1H, Ar), 7.38 (dd, ^3J = 8.5 Hz, ^4J = 1.8 Hz, 1H, Ar), 7.69 (br. s, 1H, Ar), 7.72 (d, ^3J = 9.0 Hz, 1H, Ar), 7.78 (d, ^3J = 8.5 Hz, 1H, Ar), 8.03 (br. s, 1H, NH).
^{13}C NMR (126 MHz, CDCl$_3$/DMSO-d$_6$, 4:1): δ = 41.3 (CH$_2$), 52.2 (CH$_3$O), 52.4 (CH$_3$O), 54.3 (CH$_3$O), 61.9 (CH), 71.3 (C), 104.8 (CH, Ar), 118.4 (CH, Ar), 123.5 (CH, Ar), 124.4 (CH, Ar), 126.7 (CH, Ar), 127.4 (C, Ar), 128.3 (CH, Ar), 133.2 (C, Ar), 133.4 (C, Ar), 157.0 (C, Ar), 166.75 (*C*O$_2$Me), 166.81 (*C*O$_2$Me), 195.7 (C=S).
IR (KBr): ṽ = 3468 (w), 3288 (s, NH), 1742 (s, C=O), 1718 (s, C=O), 1633 (m), 1609 (s), 1507 (s), 1487 (s), 1438 (s), 1390 (m), 1270 (s), 1238 (s), 1197 (s), 1179 (s), 1101 (m), 1081 (m), 1029 (s), 950 (w), 894 (m), 870 (m), 853 (m), 809 (m), 785 (m), 739 (m) cm^{-1}.
HRMS (ESI-TOF): *m/z* calcd for $C_{19}H_{20}NO_5S$: 374.1057 [M+H]$^+$; found: 374.1059.
Elemental analysis calcd (%) for $C_{19}H_{19}NO_5S$: C 61.11, H 5.13, N 3.75; found: C 60.99, H 5.21, N 3.66.

Dimethyl 5-(1,3-dioxoisoindolin-2-yl)-2-thioxopyrrolidine-3,3-dicarboxylate (2q) was synthesized according to the **GP3** from **1q**[S28] (method **A**: 152 mg, 0.50 mmol, 1 h). The work-up procedure afforded **2q** as a yellowish-brown solid (157 mg), which upon treatment with hot MTBE (3 × 2 mL) followed by drying on a rotary evaporator (60 °C, 3 mbar) provided a pale brown solid (140 mg, 77%); R_f = 0.08 (PE/EtOAc, 3:1); m.p. = 197–199 °C.

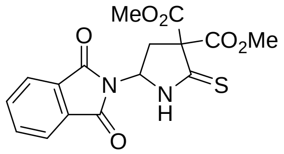

^{1}H NMR (500 MHz, DMSO-d$_6$): δ = 3.12 (dd, ^2J = 13.9 Hz, ^3J = 7.9 Hz, 1H, CH$_2$), 3.46 (dd, ^2J = 13.9 Hz, ^3J = 6.6 Hz, 1H, CH$_2$), 3.75 (s, 3H, CH$_3$O), 3.77 (s, 3H, CH$_3$O), 5.99 (dd, ^3J = 7.9 Hz, ^3J = 6.6 Hz, 1H, CH), 7.87–7.96 (m, 4H, Ar), 10.87 (br. s, 1H, NH).
^{13}C NMR (126 MHz, DMSO-d$_6$): δ = 35.2 (CH$_2$), 53.0 (CH$_3$O), 53.6 (CH$_3$O), 63.4 (CH), 71.2 (C), 123.5 (2 × CH, Ar), 131.2 (2 × C, Ar), 135.0 (2 × CH, Ar), 166.7 (2 × C=O), 166.8 (*C*O$_2$Me), 167.7 (*C*O$_2$Me), 195.6 (C=S).
IR (KBr): ṽ = 3478 (m), 3449 (w), 3113 (br. s, NH), 3023 (s), 3007 (s), 2549 (w), 2055 (br. w), 1980 (w), 1950 (w), 1779 (s, C=O), 1730 (br. s, C=O), 1611 (m), 1546 (s), 1443 (s), 1380 (s), 1337 (s), 1295 (br. s), 1136 (s), 1073 (s), 1019 (m), 962 (s), 933 (m), 872 (m), 857 (m), 802 (m), 745 (m), 717 (s) cm^{-1}.
HRMS (ESI-TOF): *m/z* calcd for $C_{16}H_{15}N_2O_6S$: 363.0645 [M+H]$^+$; found: 363.0644.

Dimethyl 5-(2,5-dioxopyrrolidin-1-yl)-2-thioxopyrrolidine-3,3-dicarboxylate (2r) was synthesized according to the **GP3** from **1r**[S29] (method **A**: 150 mg, 0.59 mmol, 3.2 h). The work-up procedure afforded **2aa** as a light-green foam (155 mg), which was treated with MTBE/CH$_2$Cl$_2$ (4 mL, 1:1), rubbed with a spatula and placed in a freezer overnight. Filtration, washing with chilled MTBE/CH$_2$Cl$_2$ (2 × 2 mL, 1:1), followed by PE (4 × 2 mL), and drying on air provided an off-white solid (83 mg, 45%); R_f = 0.34 (CH$_2$Cl$_2$/EtOAc, 1:1); m.p. = 196–197 °C.

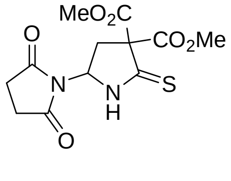

^{1}H NMR (500 MHz, DMSO-d$_6$): δ = 2.63 (br. s, 4H, 2 × CH_2CO), 3.03 (dd, ^2J = 13.8 Hz, ^3J = 8.2 Hz, 1H, CH_2), 3.24 (dd, ^2J = 13.8 Hz, ^3J = 5.6 Hz, 1H, CH_2), 3.71 (s, 3H, CH_3O), 3.72 (s, 3H, CH_3O), 5.78 (dd, ^3J = 8.2 Hz, ^3J = 5.6 Hz, 1H, CH), 10.78 (br. s, 1H, NH).
^{13}C NMR (126 MHz, DMSO-d$_6$): δ = 27.9 (2 × CH_2CO), 34.4 (CH_2), 52.9 (CH_3O), 53.5 (CH_3O), 63.4 (CH), 71.3 (C), 166.8 (*C*O_2Me), 167.9 (*C*O_2Me), 176.7 (2 × C=O), 196.0 (C=S).
IR (KBr): ṽ = 3321 (br. s, NH), 3011 (w), 2954 (m), 2568w, 1775 (s, C=O) 1747 (s, C=O), 1704 (s, C=O), 1515 (s), 1428 (m), 1376 (s), 1300 (s), 1282 (s), 1194 (s), 1174 (s), 1096 (m), 1061 (m), 1034 (m), 952 (m), 877 (m), 815 (m), 795 (m), 773 (m), 729 (m) cm^{-1}.
HRMS (ESI-TOF): *m/z* calcd for $C_{12}H_{15}N_2O_6S$: 315.0645 $[M+H]^+$; found: 315.0640.

Dimethyl 5-(furan-2-yl)-2-thioxopyrrolidine-3,3-dicarboxylate (2s) was synthesized according to the **GP3** from **1s**[S30] (method **A**: 300 mg, 1.34 mmol, 1 h). Flash chromatography (CH_2Cl_2/EtOAc, 40:1) afforded **2s** as a dark-yellow oil, which upon treatment with Et_2O (2 mL) and rubbing with a spatula provided a yellowish-white solid (269 mg, 71%); R_f = 0.26 (CH_2Cl_2/EtOAc, 40:1); m.p. = 102–104 °C.

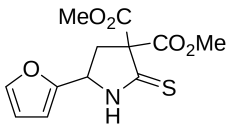

Performing the reaction under method **A'** conditions, **1s** (301 mg, 1.34 mmol) afforded **2s** as a yellow viscous oil (109 mg). The residue was dissolved in Et_2O (2 mL), treated with PE (5 mL), and rubbed with a spatula providing a pale orange-yellow solid (91 mg, 24%); m.p. = 97–99 °C. The unreacted **1s** was also isolated (130 mg, 43%).
^{1}H NMR (500 MHz, $CDCl_3$): δ = 3.15 (dd, ^2J = 13.3 Hz, ^3J = 7.6 Hz, 1H, CH_2), 3.21 (dd, ^2J = 13.3 Hz, ^3J = 7.0 Hz, 1H, CH_2), 3.83 (s, 3H, CH_3O), 3.86 (s, 3H, CH_3O), 5.03 (dd, ^3J = 7.6 Hz, ^3J = 7.0 Hz, 1H, CH), 6.31–6.38 (m, 2H, Fu), 7.41 (br. s, 1H, Fu), 8.14 (br. s, 1H, NH).
^{13}C NMR (126 MHz, $CDCl_3$): δ = 38.2 (CH_2), 53.5 (CH_3O), 53.8 (CH_3O), 56.1 (CH), 71.4 (C), 108.5 (CH, Fu), 110.6 (CH, Fu), 143.3 (CH, Fu), 149.8 (C, Fu), 167.4 (*C*O_2Me), 167.5 (*C*O_2Me), 196.8 (C=S).
IR (KBr): ṽ = 3282 (s, NH), 3146 (m), 2958 (s), 2848 (m), 2644 (w), 1744 (s, C=O), 1725 (s, C=O), 1509 (s), 1437 (s), 1269 (br. s), 1180 (m), 1144 (m), 1088 (m), 1065 (m), 1042 (m), 944 (w), 909 (w), 878 (w), 824 (w), 780 (m), 758 (m), 739 (m) cm^{-1}.
HRMS (ESI-TOF): *m/z* calcd for $C_{12}H_{14}NO_5S$: 284.0587 $[M+H]^+$; found: 284.0586.

Dimethyl 5-(thiophen-2-yl)-2-thioxopyrrolidine-3,3-dicarboxylate (2t) was synthesized according to the **GP3** from **1t**[S24] (method **A**: 302 mg, 1.26 mmol, 1 h). Flash chromatography (CH_2Cl_2/EtOAc, 50:1) afforded **2t** (the crystallization occurred after the addition of EtOAc) as an off-white solid (337 mg, 90%); R_f = 0.37 (CH_2Cl_2/EtOAc, 50:1); m.p. = 148–150 °C.

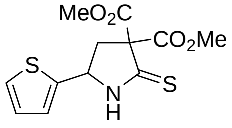

^{1}H NMR (500 MHz, $CDCl_3$): δ = 3.02 (dd, ^2J = 13.4 Hz, ^3J = 8.5 Hz, 1H, CH_2), 3.29 (dd, ^2J = 13.4 Hz, ^3J = 6.6 Hz, 1H, CH_2), 3.83 (s, 3H, CH_3O), 3.87 (s, 3H, CH_3O), 5.26 (dd, ^3J = 8.5 Hz, ^3J = 6.6 Hz, 1H, CH), 7.00 (dd, ^3J = 4.7 Hz, ^3J = 3.8 Hz, 1H, Th), 7.08 (br. d, ^3J = 3.8 Hz, 1H, Th), 7.33 (br. d, ^3J = 4.7 Hz, 1H, Th), 8.15 (br. s, 1H, NH).
^{13}C NMR (126 MHz, $CDCl_3$/DMSO-d$_6$, 6:1): δ = 41.9 (CH_2), 52.7 (CH_3O), 52.9 (CH_3O), 57.8 (CH), 71.4 (C), 125.2 (CH, Th), 125.4 (CH, Th), 126.5 (CH, Th), 141.5 (C, Th), 166.92 (*C*O_2Me), 166.94 (*C*O_2Me), 195.5 (C=S).
IR (KBr): ṽ = 3474 (m), 3425 (m), 3278 (br. s, NH), 3106 (s), 3086 (m), 2998 (m), 2955 (s), 2845 (m), 2080 (w), 1744 (s, C=O), 1723 (s, C=O), 1502 (s), 1439 (s), 1270 (br. s), 1082 (m), 1034 (m), 941 (m), 849 (m), 814 (m), 772 (m), 717 (br. m) cm^{-1}.
HRMS (ESI-TOF): *m/z* calcd for $C_{12}H_{14}NO_4S_2$: 300.0359 $[M+H]^+$; found: 300.0362.

Dimethyl 5-(thiophen-3-yl)-2-thioxopyrrolidine-3,3-dicarboxylate (2u) was synthesized according to the **GP3** from **1u**[S31] (method **A**: 302 mg, 1.26 mmol, 1 h). Flash chromatography (CH_2Cl_2/EtOAc, 50:1) afforded **2u** as a yellow oil (305 mg), which upon treatment with MTBE (2 mL) and rubbing with a spatula provided an off-white solid (268 mg, 71%); R_f = 0.27 (CH_2Cl_2/EtOAc, 50:1); m.p. = 114–116 °C.

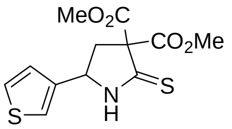

^{1}H NMR (500 MHz, $CDCl_3$): δ = 2.94 (dd, ^2J = 13.3 Hz, ^3J = 8.2 Hz, 1H, CH_2), 3.23 (dd, ^2J = 13.3 Hz, ^3J = 6.8 Hz, 1H, CH_2), 3.81 (s, 3H, MeO), 3.86 (s, 3H, MeO), 5.09 (dd, ^3J = 8.2 Hz, ^3J = 6.8 Hz, 1H, CH), 7.07 (dd, ^3J = 5.0 Hz, ^4J = 1.4 Hz, 1H, Th), 7.26–7.28 (m, 1H, Th), 7.38 (dd, ^3J = 5.0 Hz, ^4J = 2.9 Hz, 1H, Th), 8.23 (br. s, 1H, NH).
^{13}C NMR (126 MHz, $CDCl_3$): δ = 41.4 (CH_2), 53.5 (CH_3O), 53.7 (CH_3O), 58.7 (CH), 71.7 (C), 122.8 (CH, Th), 125.5 (CH, Th), 127.4 (CH, Th), 139.1 (C, Th), 167.5 (*C*O_2Me), 167.6 (*C*O_2Me), 196.8 (C=S).
IR (KBr): ṽ = 3445 (w), 3301 (s, NH), 3104 (m), 3001 (m), 2952 (m), 2842 (w), 1739 (s, C=O), 1714 (s, C=O), 1499 (s), 1433 (s), 1296 (s), 1240 (s), 1181 (m), 1101 (m), 1042 (m), 1014 (m), 936 (m), 860 (w), 789 (m), 710 (m) cm^{-1}.
HRMS (ESI-TOF): *m/z* calcd for $C_{12}H_{14}NO_4S_2$: 300.0359 $[M+H]^+$; found: 300.0351.

Dimethyl 5-(1-methylpyrrol-2-yl)-2-thioxopyrrolidine-3,3-dicarboxylate (2v) was synthesized according to the **GP3** from **1v**[S31] (method **A**: 300 mg, 1.26 mmol, 1 h). Flash chromatography (CH_2Cl_2/EtOAc, 40:1 → 30:1) afforded **2v** as an impure rose-orange viscous oil (18 mg, 5%).

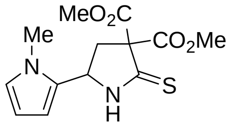

Performing the reaction under method **A'** conditions, **1v** (302 mg, 1.27 mmol) afforded **2v** as a pale-yellow solid (178 mg). The residue was treated with hot MTBE (2 mL), rubbed with a spatula, and placed in a freezer overnight. Filtration, washing with chilled Et_2O (2 × 2 mL), followed by PE (4 × 2 mL), and drying on air provided an off-white solid (97 mg, 26%); R_f = 0.18 (CH_2Cl_2/EtOAc, 40:1); m.p. = 161–163 °C.

Performing the reaction under method **B** conditions, **1v** (200 mg, 0.84 mmol) furnished **2v** as a pale-yellow solid (126 mg, 50%), which was treated as above, providing a colorless solid (73 mg, 29%) of analytical purity.
^{1}H NMR (500 MHz, $CDCl_3$): δ = 3.05 (dd, ^2J = 13.3 Hz, ^3J = 8.5 Hz, 1H, CH_2), 3.18 (dd, ^2J = 13.3 Hz, ^3J = 6.7 Hz, 1H, CH_2), 3.63 (s, 3H, CH_3N), 3.83 (s, 3H, CH_3O), 3.87 (s, 3H, CH_3O), 5.05 (dd, ^3J = 8.5 Hz, ^3J = 6.7 Hz, 1H, CH), 6.08 (dd, ^3J = 3.5 Hz, ^4J = 2.8 Hz, 1H, Pyr), 6.17 (dd, ^3J = 3.5 Hz, ^4J = 1.5 Hz, 1H, Pyr), 6.60–6.64 (m, 1H, HetAr), 8.02 (br. s, 1H, NH).

^{13}C NMR (126 MHz, $CDCl_3$/DMSO-d$_6$, 10:1): δ = 33.8 (CH_3N), 39.3 (CH_2), 53.0 (CH_3O), 53.1 (CH_3O), 54.7 (CH), 71.5 (C), 106.8 (CH, Pyr), 107.3 (CH, Pyr), 123.5 (CH, Pyr), 128.1 (C, Pyr), 167.37 (CO_2Me), 167.41 (CO_2Me), 195.3 (C=S).
IR (KBr): ṽ = 3303 (s, NH), 3101 (w), 3009 (w), 2955 (m), 1743 (s, C=O), 1726 (s, C=O), 1510 (s), 1437 (s), 1296 (s), 1285 (s), 1245 (s), 1198 (s), 1080 (m), 1035 (m), 1007 (m), 934 (m), 878 (w), 781 (m), 727 (br. s) cm^{-1}.
HRMS (ESI-TOF): *m/z* calcd for $C_{13}H_{17}N_2O_4S$: 297.0904 [M+H]$^+$; found: 297.0904.
Elemental analysis calcd (%) for $C_{13}H_{16}N_2O_4S$: C 52.69, H 5.44, N 9.45; found: C 52.48, H 5.66, N 9.28.

Dimethyl 5-(1-benzyl-1*H*-indol-4-yl)-2-thioxopyrrolidine-3,3-dicarboxylate (2w) was synthesized according to the **GP3** from **1w**[S23] (method **A**: 301 mg, 0.83 mmol, 1 h). Flash chromatography (CH_2Cl_2/EtOAc, 50:1) afforded **2w** as a pale-yellow viscous oil (288 mg), which was dissolved in MTBE and treated with PE. The concentration of the solvent on a rotary evaporator furnished a gummy solid residue, which upon treatment with MTBE and rubbing with a spatula provided an off-white solid (226 mg, 65%); R_f = 0.32 (CH_2Cl_2/EtOAc, 50:1); m.p. = 149–150 °C.

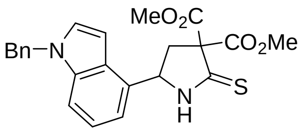

^{1}H NMR (500 MHz, $CDCl_3$): δ = 3.25 (dd, ^2J = 13.4 Hz, ^3J = 8.7 Hz, 1H, C(4)H_2), 3.34 (dd, ^2J = 13.4 Hz, ^3J = 6.9 Hz, 1H, C(4)H_2), 3.84 (s, 3H, CH_3O), 3.91 (s, 3H, CH_3O), 5.30 (dd, ^3J = 8.7 Hz, ^3J = 6.9 Hz, 1H, C(5)H), 5.34 (s, 2H, PhCH_2), 6.62 (d, ^3J = 3.3 Hz, 1H, C(3')H, Ind), 7.05 (d, ^3J = 7.2 Hz, 1H, C(5')H, Ind), 7.09–7.17 (m, 3H, 2 × *ortho*-CH, Ph, C(6')H, Ind), 7.21 (d, ^3J = 3.3 Hz, 1H, C(2')H, Ind), 7.27–7.33 (m, 4H, 2 × *meta*-CH, Ph, *para*-CH, Ph, C(7')H, Ind), 7.91 (br. s, 1H, NH).
^{13}C NMR (126 MHz, $CDCl_3$): δ = 41.1 (C(3)H_2), 50.3 (Ph*C*H_2), 53.5 (CH_3O), 53.8 (CH_3O), 62.2 (C(2)H), 72.0 (C(3)), 99.5 (C(3')H, Ind), 110.7 (C(7')H, Ind), 117.7 (C(5')H, Ind), 121.6 (C(6')H, Ind), 125.8 (C(3'a), Ar), 126.8 (2 × *ortho*-CH, Ph), 127.8 (*para*-CH, Ph), 128.8 (2 × *meta*-CH, Ph), 129.2 (C(2')H, Ind), 129.7 (C(4'), Ind), 136.7 (C(3'a), Ind), 137.1 (*ipso*-C, Ph), 167.7 (*C*O_2Me), 167.8 (*C*O_2Me), 197.2 (C=S).
IR (KBr): ṽ = 3333 (vs, NH), 2954 (w), 2889 (w), 1755 (s, C=O), 1730 (s, C=O), 1603 (w), 1493 (s), 1439 (s), 1369 (m), 1301 (s), 1240 (m), 1172 (m), 1102 (m), 1086 (m), 1034 (w), 1017 (w), 994 (w), 878 (w), 790 (w), 752 (m), 732 (w), 710 (m) cm^{-1}.
HRMS (ESI-TOF): *m/z* calcd for $C_{23}H_{23}N_2O_4S$: 423.1373 [M+H]$^+$; found: 423.1374.
Elemental analysis calcd (%) for $C_{23}H_{22}N_2O_4S$: C 65.39, H 5.25, N 6.63; found: C 65.58, H 5.44, N 6.50.

Dimethyl 5-(1-ferrocenyl)-2-thioxopyrrolidine-3,3-dicarboxylate (2x) was synthesized according to the **GP3** from **1x** (method **A**: 300 mg, 0.88 mmol, 1 h). Flash chromatography (CH_2Cl_2/EtOAc, 100:1 → 50:1) afforded **2x** (the crystallization occurred straight from the residual CH_2Cl_2 without any additional EtOAc) as a yellowish-brown solid (126 mg, 36%); R_f = 0.20 (CH_2Cl_2/EtOAc, 100:1); m.p. = 178–179 °C (dec.).

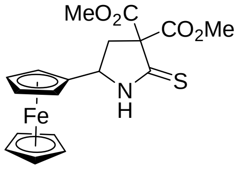

Performing the reaction under method **A'** conditions, **1x** (302 mg, 0.88 mmol) afforded **2x** as a sticky light-brown to orange semi-solid (171 mg). The residue was treated with MTBE (2–3 mL), rubbed with a spatula, and placed in a freezer for weekends. After filtration, washing with PE (3 × 2 mL), and drying on air, a dark-yellow solid was obtained (100 mg, 28%); m.p. = 178–180 °C (dec.).
^{1}H NMR (500 MHz, $CDCl_3$): δ = 2.89 (dd, ^2J = 13.1 Hz, ^3J = 8.9 Hz, 1H, CH_2), 3.14 (dd, ^2J = 13.1 Hz, ^3J = 6.4 Hz, 1H, CH_2), 3.85 (s, 3H, CH_3O), 3.88 (s, 3H, CH_3O), 4.16–4.18 (m, 1H, Cp_2Fe), 4.20–4.23 (m, 7H, Cp_2Fe), 4.23–4.25 (m, 1H, Cp_2Fe), 4.73 (dd, ^3J = 8.9 Hz, ^3J = 6.4 Hz, 1H, CH), 7.89 (br. s, 1H, NH).
^{1}H NMR (500 MHz, DMSO-d$_6$): δ = 2.92 (dd, ^2J = 13.1 Hz, ^3J = 8.2 Hz, 1H, CH_2), 3.09 (dd, ^2J = 13.1 Hz, ^3J = 6.9 Hz, 1H, CH_2), 3.72 (s, 3H, CH_3O), 3.74 (s, 3H, CH_3O), 4.19 (br. s, 2H, Cp_2Fe), 4.22 (br. s, 6H, Cp_2Fe), 4.33 (br. s, 1H, Cp_2Fe), 4.71 (dd, ^3J = 8.2 Hz, ^3J = 6.9 Hz, 1H, CH), 11.02 (br. s, 1H, NH).
^{13}C NMR (126 MHz, DMSO-d$_6$): δ = 39.2 (CH_2), 53.0 (CH_3O), 53.1 (CH_3O), 57.6 (CH), 65.5 (CH, Cp_2Fe), 67.6 (CH, Cp_2Fe), 67.8 (CH, Cp_2Fe), 68.2 (CH, Cp_2Fe), 68.4 (5 × CH, Cp_2Fe), 71.8 (C), 86.8 (C, Cp_2Fe), 167.6 (*C*O_2Me), 167.7 (*C*O_2Me), 194.3 (C=S).
IR (KBr): ṽ = 3149 (br. s, NH), 3009 (m), 2953 (m), 1733 (s, C=O), 1524 (s), 1430 (s), 1367 (m), 1268 (s), 1246 (s), 1188 (s), 1171 (s), 1104 (s), 1046 (m), 949 (m), 823 (s), 748 (m) cm^{-1}.
HRMS (ESI-TOF): *m/z* calcd for $C_{18}H_{19}FeNO_4S$: 401.0379 [M]$^+$; found: 401.0373.

Dimethyl 5-ethenyl-2-thioxopyrrolidine-3,3-dicarboxylate (2y) and **dimethyl (*E*)-2-(4-isothiocyanatobut-2-en-1-yl)malonate (4)** were synthesized according to the **GP3** from **1y**[S32] (method **A**: 300 mg, 1.63 mmol, 3.2 h). Flash chromatography (PE/EtOAc, 5:1 → 3:1) afforded **4** as a yellowish mobile oil (302 mg, 76%); R_f = 0.28 (PE/EtOAc, 5:1). Further elution provided minor product **2y** as a pear oil, which solidified into a pale-yellow solid (20 mg, 5%); R_f = 0.11 (PE/EtOAc, 5:1); m.p. = 102–104 °C.

Dimethyl 5-ethenyl-2-thioxo-5-vinylpyrrolidine-3,3-dicarboxylate (2y)

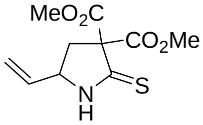

^{1}H NMR (500 MHz, $CDCl_3$): δ = 2.70 (dd, ^2J = 13.3 Hz, ^3J = 7.6 Hz, 1H, CH_2), 3.04 (dd, ^2J = 13.3 Hz, ^3J = 6.9 Hz, 1H, CH_2), 3.82 (s, 3H, CH_3O), 3.84 (s, 3H, CH_3O), 4.36–4.43 (m, 1H, CH), 5.26 (d, ^3J = 10.2 Hz, 1H, CH_2=), 5.33 (d, ^3J = 17.0 Hz, 1H, CH_2=), 5.77 (ddd, ^3J = 17.0 Hz, ^3J = 10.2 Hz, ^3J = 7.5 Hz, 1H, CH=), 8.43 (br. s, 1H, NH).
^{13}C NMR (126 MHz, $CDCl_3$): δ = 39.8 (CH_2), 53.7 (CH_3O), 53.8 (CH_3O), 61.8 (CH), 71.6 (C), 119.5 (CH=), 135.0 (CH_2=), 167.6 (*C*O_2Me), 167.7 (*C*O_2Me), 197.5 (C=S).
IR (KBr): ṽ = 3465 (w), 3172 (br. m, NH), 2997 (w), 2955 (w), 2842 (w), 1731 (s, C=O), 1516 (s), 1434 (m), 1284 (s), 1201 (m), 1145 (w), 1090 (w), 1035 (w), 1007 (w), 954 (w), 791 (w), 768 (w) cm^{-1}.
HRMS (ESI-TOF): *m/z* calcd for $C_{10}H_{14}NO_4S$: 244.0638 [M+H]$^+$; found: 244.0641.

Dimethyl (*E*)-2-(4-isothiocyanatobut-2-en-1-yl)malonate (4)

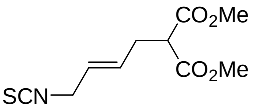

^{1}H NMR (500 MHz, $CDCl_3$): δ = 2.59–2.63 (m, 2H, CH_2CH), 3.42 (t, ^{3}J = 7.0 Hz, 1H, CH), 3.70 (s, 6H, 2 × CH_3O), 4.03 (dd, ^{3}J = 5.4 Hz, ^{4}J = 1.3 Hz, 2H, CH_2C=), 5.52–5.59 (m, 1H, CH=), 5.66–5.73 (m, 1H, CH=).
^{13}C NMR (126 MHz, $CDCl_3$): δ = 31.1 (CH_2), 46.3 (CH_2N), 51.1 (CH), 52.6 (2 × CH_3O), 125.6 (CH=), 129.9 (CH=), 132.2 (br., NCS), 169.0 (2 × CO_2Me).
IR (film): ṽ = 3438 (w), 3004 (w), 2954 (m), 2847 (w), 2086 (br. s, NCS), 1737 (s, C=O), 1641 (w), 1437 (s), 1344 (s), 1270 (s), 1231 (s), 1157 (s), 1026 (m), 972 (m), 856 (w) cm^{-1}.
HRMS (ESI-TOF): *m/z* calcd for $C_{10}H_{14}NO_4S$: 244.0638 $[M+H]^+$; found: 244.0637.

Dimethyl 5-[(*E*)-2-phenylethenyl]-2-thioxopyrrolidine-3,3-dicarboxylate (2z)[S21] was synthesized according to the **GP3** from **1z**[S33] (method **A**: 301 mg, 1.03 mmol, 1 h). Flash chromatography (CH_2Cl_2/EtOAc, 100:1 → 50:1) afforded **2z** as an oil, which upon prolonged drying on a rotary evaporator (50 °C, 2 mbar) provided a cream solid (323 mg, 87%); R_f = 0.23 (CH_2Cl_2/EtOAc, 100:1); m.p. = 137–138 °C (lit. = 138.1–138.6 °C, PE/EtOAc).[S21] Spectral data are well consistent with the reported ones.[S21]

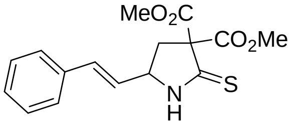

^{1}H NMR (500 MHz, $CDCl_3$): δ = 2.77 (dd, ^{2}J = 13.3 Hz, ^{3}J = 7.5 Hz, 1H, CH_2), 3.07 (dd, ^{2}J = 13.3 Hz, ^{3}J = 6.9 Hz, 1H, CH_2), 3.78 (s, 3H, CH_3O), 3.81 (s, 3H, CH_3O), 4.52–4.60 (m, 1H, CH), 6.05 (dd, ^{3}J = 15.8 Hz, ^{3}J = 7.8 Hz, 1H, CH=), 6.60 (d, ^{3}J = 15.8 Hz, 1H, CH=), 7.23–7.38 (m, 5H, Ar), 8.90 (br. s, 1H, NH).
^{13}C NMR (126 MHz, $CDCl_3$): δ = 40.0 (CH_2), 53.6 (CH_3O), 53.7 (CH_3O), 61.5 (CH), 71.6 (C), 125.7 (CH=), 126.7 (2 × CH, Ar), 128.5 (CH, Ar), 128.7 (2 × CH, Ar), 134.2 (CH=), 135.5 (C, Ar), 167.62 (*C*O_2Me), 167.64 (*C*O_2Me), 197.0 (C=S).
HRMS (ESI-TOF): *m/z* calcd for $C_{16}H_{18}NO_4S$: 320.0951 $[M+H]^+$; found: 320.0942.

Dimethyl 5-[(*E*)-2-(4-chlorophenyl)ethenyl]-2-thioxopyrrolidine-3,3-dicarboxylate (2aa) was synthesized according to the **GP3** from **1aa**[S34] (method **A**: 246 mg, 0.83 mmol, 1 h). Flash chromatography (CH_2Cl_2/EtOAc, 100:1 → 50:1) afforded **2aa** as a yellow oil (*ca*. 300 mg), which upon treatment with MTBE (2 mL) and rubbing with a spatula provided an off-white solid (251 mg, 85%); R_f = 0.18 (CH_2Cl_2/EtOAc, 100:1); m.p. = 149–150 °C.

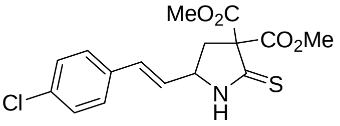

^{1}H NMR (500 MHz, DMSO-d_6): δ = 2.63 (dd, ^{2}J = 13.1 Hz, ^{3}J = 7.3 Hz, 1H, CH_2), 2.93 (dd, ^{2}J = 13.1 Hz, ^{3}J = 6.9 Hz, 1H, CH_2), 3.69 (s, 3H, CH_3O), 3.73 (s, 3H, CH_3O), 4.51–4.60 (m, 1H, CH), 6.27 (dd, ^{3}J = 15.8 Hz, ^{3}J = 7.4 Hz, 1H, CH=), 6.62 (d, ^{3}J = 15.8 Hz, 1H, CH=), 7.40 (d, ^{3}J = 8.5 Hz, 2H, Ar), 7.49 (d, ^{3}J = 8.5 Hz, 2H, Ar), 10.98 (br. s, 1H, NH).
^{13}C NMR (126 MHz, DMSO-d_6): δ = 39.4 (CH_2), 53.1 (CH_3O), 53.2 (CH_3O), 60.8 (CH), 71.7 (C), 128.1 (CH=), 128.3 (2 × CH, Ar), 128.7 (2 × CH, Ar), 131.1 (CH=), 132.5 (C, Ar), 134.7 (C, Ar), 167.5 (*C*O_2Me), 167.6 (*C*O_2Me), 195.7 (C=S).
IR (KBr): ṽ = 3300 (s, NH), 3060 (w), 1744 (s, C=O), 1722 (s, C=O), 1591 (w), 1480 (s), 1432 (s), 1409 (m), 1284 (s), 1244 (s), 1198 (s), 1174 (s), 1107 (s), 1088 (s), 1032 (m), 1011 (m), 979 (m), 933 (m), 862 (m), 811 (m), 787 (m), 750 (m) cm^{-1}.
HRMS (ESI-TOF): *m/z* calcd for $C_{16}H_{17}ClNO_4S$: 354.0561 $[M+H]^+$; found: 354.0548.

Dimethyl 5-[(*E*)-2-(4-fluorophenyl)ethenyl]-2-thioxopyrrolidine-3,3-dicarboxylate (2ab) was synthesized according to the **GP3** from **1ab** (method **A**: 301 mg, 1.08 mmol, 1 h). Flash chromatography (CH_2Cl_2/EtOAc, 50:1) afforded **2ab** as a yellow viscous oil (375 mg), which upon treatment with MTBE (2 mL) and rubbing with a spatula provided a beige solid (316 mg, 87%); R_f = 0.24 (CH_2Cl_2/EtOAc, 50:1); m.p. = 134–136 °C.

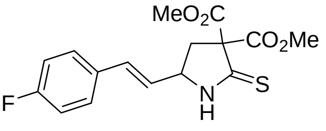

^{1}H NMR (500 MHz, $CDCl_3$): δ = 2.75 (dd, ^{2}J = 13.3 Hz, ^{3}J = 7.6 Hz, 1H, CH_2), 3.05 (dd, ^{2}J = 13.3 Hz, ^{3}J = 6.8 Hz, 1H, CH_2), 3.77 (s, 3H, CH_3O), 3.80 (s, 3H, CH_3O), 4.52–4.59 (m, 1H, CH), 5.97 (dd, ^{3}J = 15.8 Hz, ^{3}J = 7.8 Hz, 1H, CH=), 6.55 (d, ^{3}J = 15.8 Hz, 1H, CH=), 6.98 (dd, $^{3}J_{HF}$ = 8.7 Hz, ^{3}J = 8.5 Hz, 2H, Ar), 7.30 (dd, ^{3}J = 8.5 Hz, $^{4}J_{HF}$ = 5.4 Hz, 2H, Ar), 9.08 (br. s, 1H, NH).
^{13}C NMR (126 MHz, $CDCl_3$): δ = 40.0 (CH_2), 53.6 (CH_3O), 53.7 (CH_3O), 61.5 (CH), 71.6 (C), 115.6 (d, $^{2}J_{CF}$ = 22 Hz, 2 × CH, Ar), 125.4 (CH=), 128.4 (d, $^{3}J_{CF}$ = 8 Hz, 2 × CH, Ar), 131.7 (d, $^{4}J_{CF}$ = 3 Hz, C, Ar), 133.0 (CH=), 162.7 (d, $^{1}J_{CF}$ = 248 Hz, C, Ar), 167.61 (*C*O_2Me), 167.62 (*C*O_2Me), 197.0 (C=S).
^{19}F NMR (470 MHz, $CDCl_3$): δ = –112.5 (ArF).
IR (KBr): ṽ = 3136 (br. s, NH), 2990 (s), 2949 (s), 2896 (m), 1902 (w), 1744 (s, C=O), 1731 (s, C=O), 1601 (m), 1509 (s), 1449 (m), 1434 (s), 1337 (m), 1279 (s), 1243 (s), 1226 (s), 1172 (m), 1102 (m), 1030 (m), 977 (m), 919 (m), 865 (m), 825 (m), 762 (m) cm^{-1}.
HRMS (ESI-TOF): *m/z* calcd for $C_{16}H_{17}FNO_4S$: 338.0857 $[M+H]^+$; found: 338.0856.

Dimethyl 5-[(*E*)-2-(2-methoxyphenyl)ethenyl]-2-thioxopyrrolidine-3,3-dicarboxylate (2ac) was synthesized according to the **GP3** from **1ac**[S34] (method **A**: 301 mg, 1.04 mmol, 1 h). Flash chromatography (CH_2Cl_2/EtOAc, 50:1) afforded **2ac** as an oil, which upon dissolving in hot MTBE (5 mL) provided an off-white solid (287 mg, 79%); R_f = 0.32 (CH_2Cl_2/EtOAc, 50:1); m.p. = 139–140 °C.

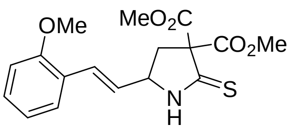

^{1}H NMR (500 MHz, $CDCl_3$): δ = 2.81 (dd, ^{2}J = 13.3 Hz, ^{3}J = 8.0 Hz, 1H, CH_2), 3.09 (dd, ^{2}J = 13.3 Hz, ^{3}J = 6.6 Hz, 1H, CH_2), 3.84 (s, 3H, CH_3O), 3.86 (s, 6H, 2 × CH_3O), 4.52–4.60 (m, 1H, CH), 6.12 (dd, ^{3}J = 15.9 Hz, ^{3}J = 8.2 Hz, 1H, CH=), 6.88 (d, ^{3}J = 8.2 Hz, 1H, Ar), 6.90–6.96 (m, 2H, CH=, Ar), 7.25–7.29 (m, 1H, Ar), 7.38 (br. d, ^{3}J = 7.5 Hz, 1H, Ar), 7.88 (br. s, 1H, NH).
^{13}C NMR (126 MHz, $CDCl_3$): δ = 40.2 (CH_2), 53.5 (CH_3O), 53.6 (CH_3O), 55.4 (CH_3O), 62.2 (CH), 71.6 (C), 110.9 (CH, Ar), 120.7 (CH, Ar), 124.3 (C, Ar), 126.1 (CH=), 127.4 (CH, Ar), 129.5 (CH, Ar), 129.6 (CH=), 156.9 (C, Ar), 167.6 (*C*O_2Me), 167.7 (*C*O_2Me), 196.9 (C=S).

IR (KBr): ṽ = 3131 (br. s, NH), 2998 (s), 2901 (m), 2838 (m), 1748 (s, C=O), 1729 (s, C=O), 1598 (m), 1578 (w), 1510 (s), 1434 (m), 1341 (m), 1282 (s), 1245 (s), 1200 (m), 1172 (m), 1102 (m), 1028 (m), 987 (m), 937 (w), 917 (w), 887 (w), 793 (m), 761 (s) cm^{-1}.
HRMS (ESI-TOF): *m/z* calcd for $C_{17}H_{20}NO_5S$: 350.1057 $[M+H]^+$; found: 350.1048.

Diethyl 5-(4-methoxyphenyl)-2-thioxopyrrolidine-3,3-dicarboxylate (2ad) was synthesized according to the **GP3** from **1ad**[S19] (method **A**: 300 mg, 1.03 mmol, 1 h). Flash chromatography (CH_2Cl_2/EtOAc, 50:1) afforded **2ad** as a yellow oil (289 mg), which was dissolved in MTBE (2 mL) and treated with PE (2 mL). Rubbing with a spatula provided a yellowish-white solid (265 mg, 73%); R_f = 0.17 (CH_2Cl_2/EtOAc, 50:1); m.p. = 97–98 °C.

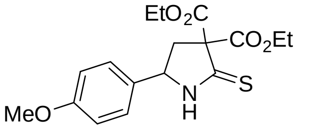

^{1}H NMR (500 MHz, $CDCl_3$): δ = 1.31 (t, ^3J = 7.2 Hz, 3H, CH_3), 1.33 (t, ^3J = 7.2 Hz, 3H, CH_3), 2.86 (dd, ^2J = 13.4 Hz, ^3J = 8.9 Hz, 1H, CH_2), 3.18 (dd, ^2J = 13.4 Hz, ^3J = 6.6 Hz, 1H, CH_2), 3.83 (s, 3H, CH_3O), 4.27–4.36 (m, 4H, 2 × CH_2O), 4.90 (dd, ^3J = 8.9 Hz, ^3J = 6.6 Hz, 1H, CH), 6.91 (d, ^3J = 8.7 Hz, 2H, Ar), 7.25 (d, ^3J = 8.7 Hz, 2H, Ar), 7.89 (br. s, 1H, NH).
^{13}C NMR (126 MHz, $CDCl_3$): δ = 13.89 (CH_3), 13.90 (CH_3), 42.4 (CH_2), 55.3 (CH_3O), 62.4 (CH), 62.5 (CH_2O), 62.7 (CH_2O), 71.7 (C), 114.3 (2 × CH, Ar), 127.8 (2 × CH, Ar), 130.2 (C, Ar), 159.8 (C, Ar), 167.0 (*C*O_2Et), 167.2 (*C*O_2Et), 197.1 (C=S).
IR (KBr): ṽ = 3294 (br. m, NH), 3001 (m), 2948 (m), 2904 (w), 2841 (w), 2053 (w), 1736 (s, C=O), 1725 (s, C=O), 1612 (m), 1584 (w), 1509 (s), 1469 (s), 1390 (m), 1359 (m), 1317 (m), 1253 (s), 1196 (s), 1176 (s), 1092 (m), 1046 (m), 1021 (s), 837 (m), 811 (m), 793 (m), 779 (m), 746 (m), 708 (m) cm^{-1}.
HRMS (ESI-TOF): *m/z* calcd for $C_{17}H_{22}NO_5S$: 352.1213 $[M+H]^+$; found: 352.1211.
Elemental analysis calcd (%) for $C_{17}H_{21}NO_5S$: C 58.10, H 6.02, N 3.99. Found: C 58.24, H 6.02, N 3.79.

Methyl (3*RS*,5*SR*)-3-cyano-5-(4-methoxyphenyl)-2-thioxopyrrolidine-3-carboxylate (2ae) was synthesized according to the **GP3** from **1ae**[S35] (method **A**: 300 mg, 1.30 mmol, 2.2 h). Flash chromatography (PE/EtOAc, 2:1) afforded crude **(3*RS*,5*RS*)-2ae** as a pale brown solid (102 mg); R_f = 0.40 (PE/EtOAc, 2:1) and **(3*RS*,5*SR*)-2ae** as a yellow solid (157 mg, 42%); R_f = 0.28 (PE/EtOAc, 2:1). Minor **(3*RS*,5*RS*)-2ae** could not purified to the analytically pure compound, while major **(3*RS*,5*SR*)-2ae** was obtained in a pure form by the treatment with MTBE (2 mL), rubbing with a spatula, and placing in a freezer overnight. Filtration, washing with chilled MTBE (2 × 2 mL), and drying provided a yellowish-white solid, m.p. = 168–169 °C.

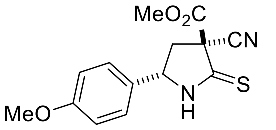

(3*RS*,5*SR*)-2ae:
^{1}H NMR (500 MHz, acetone-d_6): δ = 2.67 (dd, ^2J = 13.6 Hz, ^3J = 8.2 Hz, 1H, C(4)H_2), 3.47 (dd, ^2J = 13.6 Hz, ^3J = 7.0 Hz, 1H, C(4)H_2), 3.81 (s, 3H, CH_3OAr), 3.89 (s, 3H, CH_3O_2C), 5.34 (dd, ^3J = 8.2 Hz, ^3J = 7.0 Hz, 1H, C(5)H), 6.99 (d, ^3J = 8.7 Hz, 2H, C(3')H, C(5')H, Ar), 7.39 (d, ^3J = 8.7 Hz, 2H, C(2')H, C(6')H, Ar), 10.17 (br. s, 1H, NH).
^{13}C NMR (126 MHz, acetone-d_6): δ = 44.2 (C(4)H_2), 54.7 (*C*H_3OAr), 55.7 (*C*H_3O_2C), 63.4 (C(3)), 64.6 (C(5)H), 115.2 (2 × CH, C(3')H, C(5')H, Ar), 117.5 (CN), 128.9 (2 × CH, C(2')H, C(6')H, Ar), 131.5 (C(1'), Ar), 161.0 (C(4'), Ar), 166.3 (*C*O_2Me), 193.8 (C=S).
IR (KBr): ṽ = 3352 (br. s, NH), 3003 (m), 2964 (m), 2921 (m), 2842 (m), 2247 (m, C≡N), 2048 (w), 1900 (w), 1744 (s, C=O), 1727 (s, C=O), 1614 (s), 1589 (s), 1515 (s), 1439 (s), 1379 (m), 1278 (s), 1249 (s), 1180 (s), 1115 (s), 1070 (m), 1028 (s), 958 (m), 875 (m), 831 (m), 777 (m) cm^{-1}.
HRMS (ESI-TOF): *m/z* calcd for $C_{14}H_{15}N_2O_3S$: 291.0798 $[M+H]^+$; found: 291.0803.

The structure of **(3*RS*,5*SR*)-2ae** was unambiguously proved by single-crystal X-ray analysis (Fig. S5). Crystals were grown by slow evaporation of the solution of the compound in EtOAc/PE mixture.

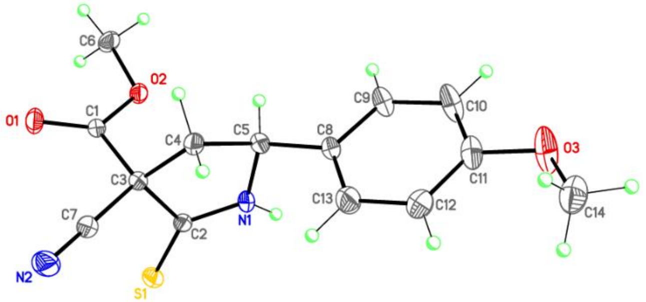

Figure S5. Structure of **2ae** determined by single-crystal X-ray analysis (thermal ellipsoids are shown at 50% probability; CCDC 2005164).

4-(8,8-dimethyl-6,10-dioxo-1-thioxo-7,9-dioxa-2-azaspiro[4.5]decan-3-yl)benzonitrile (2af) was synthesized according to the **GP3** from **1af** (method **A**: 300 mg, 1.11 mmol, 1 h). Flash chromatography (CH_2Cl_2/EtOAc, 30:1) afforded **2af** as a pale greenish-yellow solid (93 mg, 25%); R_f = 0.33 (CH_2Cl_2/EtOAc, 30:1); m.p. = 189–191 °C (dec.).

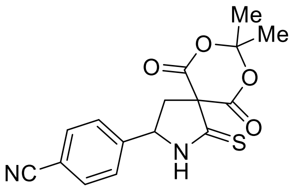

^{1}H NMR (500 MHz, acetone-d_6): δ = 1.77 (s, 3H, CH_3), 1.98 (s, 3H, CH_3), 2.79 (dd, ^2J = 13.6 Hz, ^3J = 8.1 Hz, 1H, C(4)H_2), 3.44 (dd, ^2J = 13.6 Hz, ^3J = 7.6 Hz, 1H, C(4)H_2), 5.56 (dd, ^3J = 8.1 Hz, ^3J = 7.6 Hz, 1H, C(3)H), 7.73 (d, ^3J = 8.2 Hz, 2H, C(2')H, C(6')H, Ar), 7.88 (d, ^3J = 8.2 Hz, 2H, C(3')H, C(5')H, Ar), 10.32 (br. s, 1H, NH).
^{13}C NMR (126 MHz, acetone-d_6): δ = 29.2 (CH_3), 29.8 (CH_3), 44.4 (C(4)H_2), 65.2 (C(3)H), 68.5 (C(5)), 107.9 (C(8)Me_2), 113.2 (C(1'), Ar), 119.0 (C≡N), 128.7 (2 × CH, C(2')H, C(6')H, Ar), 133.7 (2 × CH, C(3')H, C(5')H, Ar), 146.1 (C(4'), Ar), 166.4 (CO_2R), 166.9 (CO_2R), 197.8 (C=S).

IR (KBr): ṽ = 3498 (w), 3277 (br. s, NH), 3075 (w), 3010 (m), 2924 (w), 2234 (s, C≡N), 1933 (w), 1779 (s, C=O), 1743 (s, C=O), 1609 (m), 1524 (s), 1446 (m), 1418 (m), 1391 (s), 1299 (br. s), 1198 (s), 1149 (m), 1110 (m), 1070 (m), 1037 (m), 980 (m), 930 (m), 877 (m), 836 (m), 714 (m) cm^{-1}.
HRMS (ESI-TOF): *m/z* calcd for $C_{16}H_{15}N_2O_4S$: 331.0747 $[M+H]^+$; found: 331.0746.

8,8-dimethyl-3-phenyl-1-thioxo-7,9-dioxa-2-azaspiro[4.5]decane-6,10-dione (2ag) was synthesized according to the **GP3** from **1ag**[S36] (method **A**: 202 mg, 0.82 mmol, 1 h). Flash chromatography (CH_2Cl_2) afforded **2ag** as an off-white solid (133 mg, 53%); R_f = 0.32 (CH_2Cl_2); m.p. = 175–176 °C (dec.).

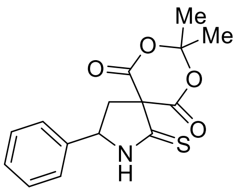

¹H NMR (500 MHz, acetone-d$_6$): δ = 1.77 (d, ^4J = 0.5 Hz, 3H, CH_3), 1.98 (s, ^4J = 0.5 Hz, 3H, CH_3), 2.80 (dd, ^2J = 13.5 Hz, ^3J = 8.5 Hz, 1H, CH_2), 3.35 (dd, ^2J = 13.5 Hz, ^3J = 7.4 Hz, 1H, CH_2), 5.42 (dd, ^3J = 8.5 Hz, ^3J = 7.4 Hz, 1H, CH), 7.37–7.41 (m, 1H, Ar), 7.44–7.48 (m, 2H, Ar), 7.49–7.53 (m, 2H, Ar), 10.24 (br. s, 1H, NH).
¹³C NMR (126 MHz, acetone-d$_6$): δ = 29.2 (CH_3), 29.7 (CH_3), 44.9 (CH_2), 65.9 (CH), 68.6 (C), 107.7 (*C*Me$_2$), 127.6 (2 × CH, C(2')H, C(6')H, Ar), 129.5 (C(4')H, Ar), 129.8 (2 × CH, C(3')H, C(5')H, Ar), 140.5 (C(1'), Ar), 166.5 (CO_2R), 167.1 (CO_2R), 197.1 (C=S).
IR (KBr): ṽ = 3512 (w), 3156 (br. s, NH), 3035 (m), 2931 (w), 1903 (w), 1778 (vs, C=O), 1745 (vs, C=O), 1540 (vs), 1458 (w), 1438 (w), 1395 (m), 1293 (br. vs), 1200 (s), 1127 (s), 1090 (m), 1031 (m), 997 (w), 964 (m), 931 (m), 869 (m), 771 (s), 704 (s) cm^{-1}.
HRMS (ESI-TOF): *m/z* calcd for $C_{15}H_{16}NO_4S$: 306.0795 $[M+H]^+$; found: 306.0793.

Dimethyl 5-(3,4-dimethoxyphenyl)-2-oxopyrrolidine-3,3-dicarboxylate (5)

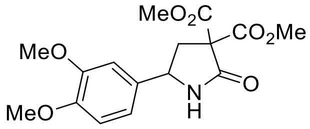

A solution of pyrrolidine-2-thione **2a** (354 mg, 1.00 mmol) in CH_2Cl_2 (10 mL) was treated in small portions with 70% *m*CPBA (741 mg, 3.01 mmol) under external ice-water bath cooling (the complete addition took 15–20 min). The reaction was allowed to warm up to r.t. and stirred for 1 h until the TLC control showed the complete consumption of the starting material. The yellow reaction mixture was slowly treated with saturated $NaHCO_3$ solution (30 mL) until neutral pH. The organic layer was separated, and the aqueous phase was extracted with additional CH_2Cl_2 (2 × 10 mL). The combined organic fractions were washed with saturated NaCl solution (20 mL), dried with anhydrous Na_2SO_4, loaded on silica, and concentrated under reduced pressure. Flash chromatography (CH_2Cl_2/EtOAc, 2:1) afforded **5** as an orange oil, which solidified upon gentle drying on a rotary evaporator (40 °C, 50–100 mbar) providing a beige solid (292 mg, 86%); R_f = 0.28 (CH_2Cl_2/EtOAc, 2:1); m.p. = 150–151 °C.
¹H NMR (500 MHz, $CDCl_3$): δ = 2.65 (dd, ^2J = 13.5 Hz, ^3J = 7.9 Hz, 1H, CH_2), 3.15 (dd, ^2J = 13.5 Hz, ^3J = 6.9 Hz, 1H, CH_2), 3.81 (s, 3H, CH_3O), 3.869 (s, 3H, CH_3O), 3.872 (s, 3H, CH_3O), 3.88 (s, 3H, CH_3O), 4.70 (dd, ^3J = 7.9 Hz, ^3J = 6.9 Hz, 1H, CH), 6.20 (br. s, 1H, NH), 6.81–6.87 (m, 3H, Ar).
¹³C NMR (126 MHz, $CDCl_3$): δ = 40.4 (CH_2), 53.4 (CH_3O), 53.6 (CH_3O), 55.2 (CH), 55.90 (CH_3O), 55.91 (CH_3O), 63.3 (C), 108.8 (CH, Ar), 111.2 (CH, Ar), 118.3 (CH, Ar), 132.8 (C, Ar), 148.9 (C, Ar), 149.4 (C, Ar), 167.4 (*C*O$_2$Me), 167.6 (*C*O$_2$Me), 169.5 (C=O).
IR (KBr): ṽ = 3188 (w), 3086 (w), 3004 (w), 2957 (w), 2876 (w), 2845 (w), 1754 (vs, C=O), 1736 (s, C=O), 1706 (s, C=O), 1611 (w), 1594 (w), 1521 (s), 1468 (m), 1440 (m), 1422 (w), 1354 (w), 1305 (w), 1265 (s), 1244 (s), 1198 (m), 1178 (w), 1161 (m), 1135 (w), 1078 (m), 1024 (m), 951 (w), 857 (w), 798 (m), 750 (w) cm^{-1}.
HRMS (ESI-TOF): *m/z* calcd for $C_{16}H_{20}NO_7$: 338.1234 $[M+H]^+$; found: 338.1230.

Dimethyl 5-(3,4-dimethoxyphenyl)-2-(methylthio)-4,5-dihydro-3*H*-pyrrole-3,3-dicarboxylate (6)

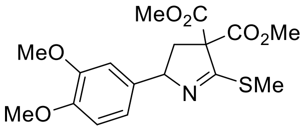

A 16 mL vial was charged with pyrrolidine-2-thione **2a** (300 mg, 0.85 mmol) and $CHCl_3$ (4.25 mL). The resulting solution was treated successively with DIPEA (222 µL, 1.27 mmol) and dimethyl sulfate (161 µL, 1.70 mmol). The vial was tightly sealed, placed into an oil bath, preheated to 100 °C, and vigorously stirred for 1 h. The TLC control showed the complete consumption of the starting material. The yellow reaction mixture was allowed to cool to r.t. and diluted with CH_2Cl_2 (25 mL), washed with water (2 × 20 mL), and saturated NaCl solution (20 mL), dried with anhydrous Na_2SO_4, loaded on silica, and concentrated under reduced pressure. Flash chromatography (CH_2Cl_2/EtOAc, 30:1) afforded **6** as a pale-yellow viscous oil (253 mg), which was dissolved in Et_2O (2 mL), treated with liquid N_2, and rubbed with a spatula. The concentration at r.t. and drying on a rotary evaporator at 60–70 °C provided a colorless solid (232 mg, 74%); R_f = 0.26 (CH_2Cl_2/EtOAc, 30:1); m.p. = 85–86 °C.
¹H NMR (500 MHz, $CDCl_3$): δ = 2.52 (s, 3H, CH_3S), 2.54 (dd, ^2J = 13.5 Hz, ^3J = 8.2 Hz, 1H, CH_2), 3.14 (dd, ^2J = 13.5 Hz, ^3J = 7.1 Hz, 1H, CH_2), 3.79 (s, 3H, CH_3O), 3.83 (s, 3H, CH_3O), 3.86 (s, 3H, CH_3O), 3.87 (s, 3H, CH_3O), 5.09 (dd, ^3J = 8.2 Hz, ^3J = 7.1 Hz, 1H, CH), 6.80 (br. s, 1H, Ar), 6.831 (s, 1H, Ar), 6.834 (s, 1H, Ar).
¹³C NMR (126 MHz, $CDCl_3$): δ = 14.9 (CH_3S), 43.7 (CH_2), 53.2 (CH_3O), 53.4 (CH_3O), 55.9 (CH_3O), 56.0 (CH_3O), 72.5 (C), 73.4 (CH), 109.9 (CH, Ar), 111.3 (CH, Ar), 118.7 (CH, Ar), 135.1 (C, Ar), 148.4 (C, Ar), 149.1 (C, Ar), 168.1 (*C*O$_2$Me), 168.16 (C=N), 168.23 (*C*O$_2$Me).
IR (KBr): ṽ = 3000 (w), 2955 (w), 2838 (w), 1753 (m, C=O), 1728 (vs, C=O), 1580 (m), 1520 (m), 1464 (m), 1425 (m), 1321 (w), 1282 (s), 1251 (s), 1197 (m), 1169 (m), 1138 (m), 1101 (m), 1076 (w), 1027 (s), 986 (w), 852 (w), 819 (m), 783 (w) cm^{-1}.
HRMS (ESI-TOF): *m/z* calcd for $C_{17}H_{22}NO_6S$: 368.1162 $[M+H]^+$; found: 368.1157.

1-phenyl-3-(2-thioxo-3,4-dihydro-2*H*-benz[*e*][1,3]oxazin-4-yl)propan-1-one (7) was synthesized according to the **GP3** from **1ah**[S37] (method **A**: 250 mg, 1.05 mmol, 2 h). Flash chromatography (CH_2Cl_2/EtOAc, 30:1) afforded **7** as a pale-orange viscous oil (233 mg), which was dissolved in hot CCl_4 (1 mL), cooled to r.t., and treated with PE (1 mL). The concentration of the solvent on a rotary evaporator at r.t. furnished a semi-solid residue, which upon treatment with MTBE (2 mL), rubbing under ultrasound irradiation, concentrating the solvent at r.t., and drying on a rotary evaporator at 60 °C provided an off-white solid (208 mg, 67%); R_f = 0.31 (CH_2Cl_2/EtOAc, 30:1); m.p. = 122–124 °C.

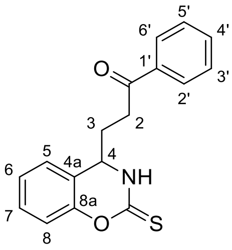

^{1}H NMR (500 MHz, DMSO-d$_6$): δ = 1.98–2.08 (m, 1H, C(3)H$_2$), 2.09–2.18 (m, 1H, C(3)H$_2$), 3.01 (ddd, ^2J = 17.8 Hz, ^3J = 8.7 Hz, ^3J = 5.5 Hz, 1H, C(2)H$_2$), 3.10 (ddd, ^2J = 17.8 Hz, ^3J = 8.7 Hz, ^3J = 6.6 Hz, 1H, C(2)H$_2$), 4.62–4.72 (m, 1H, C(4)H), 7.14 (d, ^3J = 8.0 Hz, 1H, C(8)H, Ar), 7.21–7.26 (m, 1H, C(6)H, Ar), 7.32–7.40 (m, 2H, C(5)H, C(7)H, Ar), 7.50–7.55 (m, 2H, C(3')H, C(5')H, Ph), 7.61–7.66 (m, 1H, C(4')H, Ph), 7.86–7.94 (m, 2H, C(2')H, C(6')H, Ph), 10.52 (br. d, ^3J = 2.8 Hz, 1H, NH).
^{13}C NMR (126 MHz, DMSO-d$_6$): δ = 32.1 (C(3)H$_2$), 32.9 (C(2)H$_2$), 51.6 (C(4)H), 115.6 (C(8)H, Ar), 120.6 (C(4a), Ar), 125.4 (C(6)H, Ar), 126.4 (C(5)H, Ar), 127.8 (2 × CH, C(2')H, C(6')H, Ph), 128.7 (2 × CH, C(3')H, C(5')H, Ar), 129.2 (C(7)H, Ar), 133.2 (C(4')H, Ar), 136.4 (C(1'), Ph), 148.3 (C(8a), Ar), 181.9 (C=S), 198.6 (C=O).
IR (KBr): ṽ = 3203 (m), 3063 (w), 2962 (w), 2935 (w), 1678 (s, C=O), 1626 (w), 1592 (m), 1548 (s), 1489 (w), 1451 (m), 1355 (m), 1258 (m), 1200 (s), 1154 (s), 1101 (m), 993 (w), 933 (w), 752 (s) cm^{-1}.
HRMS (ESI-TOF): *m/z* calcd for $C_{17}H_{16}NO_2S$: 298.0896 [M+H]$^+$; found: 298.0895.

(1*RS*,2*RS*)-2-thiocyanatocyclohexan-1-ol (9)[S38]

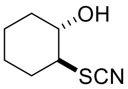

A 4 mL vial was charged with HMimNCS **3a** (307 mg, 2.17 mmol, 1.1 equiv) preliminary melted with a heat-gun. Cyclohexene oxide **8** (200 µL, 1.98 mmol) was added dropwise, and the vial was tightly sealed and vigorously stirred for 48 h at r.t. The reaction mixture was dissolved in CH_2Cl_2 (10 mL) and washed successively with water (10 mL), 10% citric acid solution (10 mL), and saturated NaCl solution (10 mL). The organic fraction was dried with anhydrous Na_2SO_4 and concentrated under reduced pressure at r.t. affording **9** as a dark-yellow viscous oil (174 mg, 56%). Spectral data are well consistent with the reported ones.[S38]
^{1}H NMR (500 MHz, CDCl$_3$): δ = 1.24–1.39 (m, 3H, C(4)H$_2$ + C(5)H$_2$ + C(6)H$_2$), 1.60–1.70 (m, 1H, C(3)H$_2$), 1.72–1.83 (m, 2H, C(4)H$_2$ + C(5)H$_2$), 2.07–2.15 (m, 1H, C(6)H$_2$), 2.20–2.28 (m, 1H, C(3)H$_2$), 2.92 (ddd, J = 12.2, 9.8, 4.1 Hz, 1H, C(2)H), 3.04 (br. s, 1H, OH), 3.52 (td, J = 9.4, 4.5 Hz, 1H, C(1)H).
^{13}C NMR (CDCl$_3$): δ = 24.0 (C(5)H$_2$), 25.9 (C(4)H$_2$), 32.8 (C(3)H$_2$), 35.2 (C(6)H$_2$), 55.2 (C(2)H), 72.7 (C(1)H), 111.2 (SCN).
IR (KBr): ṽ = 3421 (br. s, OH), 2940 (vs), 2861 (s), 2152 (vs, SCN), 1670 (br. w), 1449 (s), 1406 (w), 1357 (m), 1303 (w), 1262 (m), 1206 (m), 1127 (m), 1069 (vs), 959 (s), 896 (w), 866 (m), 795 (w), 719 (m) cm^{-1}.
HRMS (ESI-TOF): *m/z* calcd for $C_7H_{12}NOS$: 158.0634 [M+H]$^+$; found: 158.0637.

3-(1*H*-imidazol-1-yl)propan-1-ammonium thiocyanate (11)

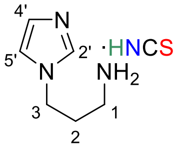

A pre-weighed 10 mL pear-shaped flask was charged with HMimNCS **3a** (215 mg, 1.52 mmol) preliminary melted with a heat-gun. 1-(3-Aminopropyl)imidazole **10** (200 µL, 1.68 mmol, 1.1 equiv) was added in one portion resulting in an exothermic reaction. A prolonged drying of the reaction mixture on a rotary evaporator (80–85 °C, 1–2 mbar) afforded a dark-orange viscous oil (295 mg), which was treated with Et_2O/dioxane (6 mL, 5:1) and rubbed with a spatula under ultrasound irradiation; the obtained suspension was chilled in a fridge. The organic layer was withdrawn with a Pasteur pipette and discarded, and the treatment with Et_2O/dioxane (3 mL, 5:1) was repeated twice. The residue was re-evaporated with CyHex/dioxane (3 mL, 5:1) and dried at 80 °C (3 mbar) until the constant weight was achieved, providing PIL **11** as an orange extremely viscous oil (270 mg, 96%).
^{1}H NMR (500 MHz, DMSO-d$_6$): δ = 1.94 (quint, ^3J = 7.2 Hz, 2H, C(2)H$_2$), 2.64–2.74 (m, 2H, C(1)H$_2$), 4.05 (t, ^3J = 6.9 Hz, 2H, C(3)H$_2$), 6.91 (s, 1H, C(4')H, Im), 7.18 (s, 1H, C(5')H, Im), 7.28 (br. s, 3H, NH$_2$ + NH$^+$···[NCS]), 7.64 (s, 1H, C(2')H, Im).
^{13}C NMR (126 MHz, DMSO-d$_6$): δ = 29.3 (C(2)H$_2$), 36.5 (C(1)H$_2$), 43.2 (C(3)H$_2$), 119.5 (C(5')H, Im), 128.5 (C(4')H, Im), 130.5 (NCS), 137.3 (C(2')H, Im).
IR (KBr): ṽ = 3462 (br. w, NH), 3109 (br. s, NH), 2959 (br. s, NH), 2057 (vs, NCS), 1630 (m), 1514 (s), 1455 (m), 1402 (w), 1284 (w), 1233 (m), 1154 (w), 1108 (m), 1085 (s), 1031 (w), 923 (m), 831 (m), 751 (m), 663 (m) cm^{-1}.
HRMS (ESI-TOF): *m/z* calcd for $C_6H_{12}N_3$: 126.1026 [$M_{B\cdot H}$]$^+$; found: 126.1020.

3-Thioxohexahydro-1*H*-pyrrolo[1,2-*c*]imidazol-1-one (13)[S39] was synthesized according to the **GP3** from L-proline **12** (method **A**: 150 mg, 1.30 mmol, 150 °C, 1 h).[1] Flash chromatography (CH_2Cl_2/EtOAc, 20:1) afforded **13** as a light-beige sparkling solid (107 mg, 52%); R_f = 0.09 (CH_2Cl_2/EtOAc, 20:1); m.p. = 167–168 °C. Spectral data are well consistent with the reported ones.[S39]

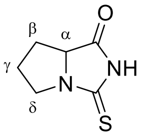

^{1}H NMR (500 MHz, CDCl$_3$): δ = 1.75–1.85 (m, 1H, CH$_2$), 2.12–2.23 (m, 1H, CH$_2$), 2.25–2.34 (m, 2H, CH$_2$), 3.52 (ddd, J = 11.9, 8.9, 3.2 Hz, 1H, CH$_2$), 3.93 (dt, J = 11.9, 8.3 Hz, 1H, CH$_2$), 4.28 (dd, J = 10.3, 6.9 Hz, 1H, CH), 8.58 (br. s, 1H, NH).

1) Due to the high solubility of the product **13** in water, the reaction mixture was dissolved in distilled water (10 mL) and extracted with boiling EtOAc (3 × 10 mL).

^{1}H NMR (500 MHz, acetone-d_6/DMSO-d_6, 10:1): δ = 1.68–1.81 (m, 1H, C(β)H_2), 2.11–2.28 (m, 3H, C(β)H_2 + C(γ)H_2), 3.37–3.47 (m, 1H, C(δ)H_2), 3.78–3.86 (m, 1H, C(δ)H_2), 4.31–4.39 (m, 1H, C(α)H), 11.11 (br. s, 1H, NH).
^{13}C NMR (126 MHz, acetone-d_6/DMSO-d_6, 10:1): δ = 27.0 (C(β)H_2), 27.6 (C(γ)H_2), 48.3 (C(δ)H_2), 67.2 (C(α)H), 175.6 (C=O), 187.4 (C=S).
IR (KBr): $\tilde{\nu}$ = 3433 (w), 3167 (br. s, NH), 2965 (m), 2885 (w), 2788 (w), 2634 (w), 1747 (vs, C=O), 1707 (vs, C=O), 1500 (vs), 1407 (s), 1335 (s), 1293 (w), 1247 (s), 1225 (s), 1144 (s), 1049 (m), 985 (m), 888 (w), 780 (br. m) cm^{-1}.

Regeneration of thiocyanate ion-containing protic ionic liquids

The representative procedure for the regeneration of HMimNCS

The gram-scale preparation of **2a** was carried out in HMimNCS (12.51 g, *ca.* 10.2 mL) according to method **A** (see above). The reaction mixture was dissolved in CH_2Cl_2 (100 mL) and washed with distilled water (2 × 50 mL). The combined aqueous phase was washed successively with EtOAc (2 × 100 mL) and CH_2Cl_2 (2 × 100 mL), concentrated on a rotary evaporator at 60 °C and dried at 80 °C and 4–5 mbar until the constant weight was achieved (*ca.* 8 h), affording HMimNCS as an orange moderately viscous liquid (10.95 g, $\eta_{(regen.\ PIL)} = m_{(regen.\ PIL)}/[m_{(start.\ PIL)}–m_{(PIL,\ 1\ equiv)}]$ = 10.95/(12.51–1.44) = 99%) with satisfactory elemental analysis (Fig. S6). **Elemental analysis** calcd (%) for $C_5H_7N_3S$ (HMimNCS): C 42.53, H 5.00, N 29.76; found: C 42.26, H 4.89, N 29.47. Performing the ring-opening reaction in the regenerated HMimNCS under method **A** conditions furnished **2a** with comparable efficiency (Table S1).

Table S1. HMimNCS regeneration.

Entry	Reaction with regenerated PIL	m (1a), g	v (1a), mmol	η (2a), %	η (regenerated 3a), %
1	new	3.00	10.2	89	99
2	1st	2.50	8.50	89	98
3	2nd	2.00	6.80	87	98
4	3rd	1.50	5.10	87	96
5	4th	1.00	3.40	86	96

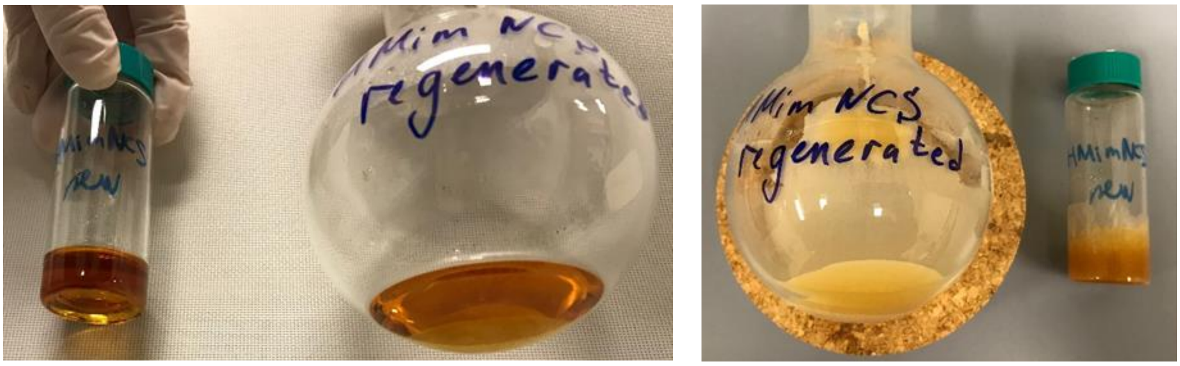

Figure S6. The appearance of freshly synthesized and regenerated PIL in melted and solid states.

The representative procedure for the regeneration of Et_3N·HNCS

The preparation of **2m** was carried out in Et_3N·HNCS (1.035 g, 1.085 mL) according to method **A'** (see above). The reaction mixture was dissolved in CH_2Cl_2 (20 mL) and washed with distilled water (20 mL). The aqueous phase was washed with EtOAc (3 × 10 mL), concentrated on a rotary evaporator at 60 °C and dried at 80 °C and 4–5 mbar until the constant weight was achieved (*ca.* 10 h; upon prolonged drying on a rotary evaporator PIL was partially sublimed into the bump trap), affording Et_3N·HNCS as a yellow to orange oily liquid (789 mg, $\eta_{(regen.\ PIL)} = m_{(regen.\ PIL)}/[m_{(start.\ PIL)}–m_{(PIL,\ 1\ equiv)}]$ = 789/(1035–174) = 92%) with satisfactory elemental analysis. **Elemental analysis** calcd (%) for $C_7H_{16}N_2S$ (Et_3N·HNCS): C 52.46, H 10.06, N 17.48; found: C 52.53, H 10.21, N 17.59. Performing the ring-opening reaction in the regenerated Et_3N·HNCS under method **B** conditions furnished **2m** in the same yield compared to method **B** in freshly prepared PIL.

Restrictions of the method (GP3): the list of inappropriate substrates

When reactions with substrates, given in Fig. S7, were performed under **GP3** conditions, complex mixtures containing unreacted starting material were obtained. The increase in temperature and reaction time led predominantly to the substrate decomposition. An appropriate PIL should be designed for these substrates.

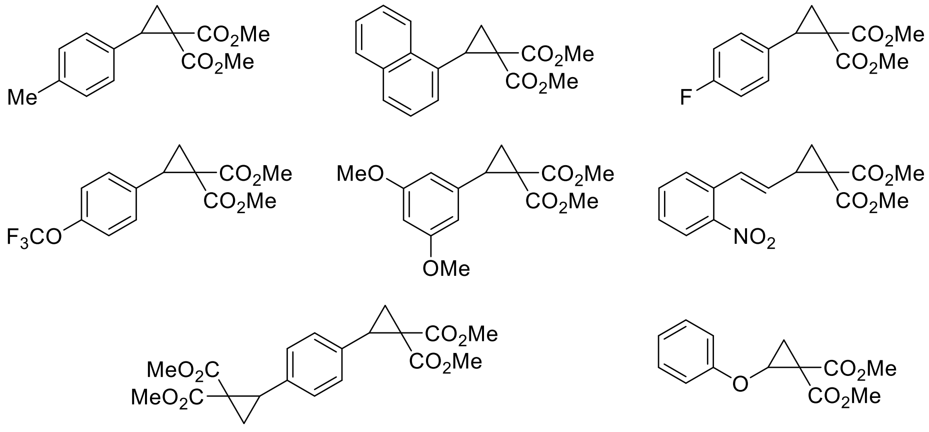

Figure S7. Donor–acceptor cyclopropanes with lowered reactivity in the studied reaction.

The obtained data demonstrate that moderately active D–A cyclopropanes, containing 4-tolyl, 4-fluorophenyl, 1-naphthyl group as a donor, reacted under the studied conditions slower than D–A cyclopropanes, bearing electron-enriched aromatic groups. Similarly, 4-(trifluoromethoxy)phenyl- and 3,5-dimethoxyphenyl-substituted cyclopropanes exhibited lowered reactivity as both *para*-trifluoromethoxy and *meta*-methoxy groups are known to have a moderate electron-withdrawing nature. Moreover, contrary to styryl-, *para*-methoxystyryl-, and *para*-halostyryl-substituted D–A cyclopropanes, *ortho*-nitrostyryl-substituted cyclopropane was found to be inefficient, providing an inseparable mixture of products. Again, the substitution of a good cation-stabilizing group by an electron-deficient one prevented the efficient three-membered ring opening with thiocyanate ion. On the other hand, 2-phenoxycyclopropane-1,1-diester was too reactive and resulted in the full conversion of the substrate but yielded a complex mixture of products.

Finally, we have found that dimethyl 2-(5-nitrofuran-2-yl)cyclopropane-1,1-dicarboxylate did not react at all. Moreover, conversion of 2-(3,4-dimethoxyphenyl)cyclobutane-1,1-dicarboxylate was absent even at 120 °C confirming much lower reactivity of D–A cyclobutanes *vs*. the corresponding D–A cyclopropanes. Unreactive substrates are given in Fig. S8.

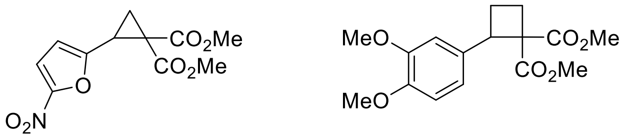

Figure S8. Substrates, which did not participate in the studied reaction.

Preliminary estimation of environmental factors (EF)[S40]

We evaluated the rough E-factor for the 3 gram-scale transformation (**1a**→**2a**). Since the excess of PIL and solvents employed for extraction can be regenerated, a simplified E-factor can be calculated:

$$sEF = \frac{\mathrm{m(waste)}}{\mathrm{m(product)}} = \frac{\mathrm{m}(\mathbf{2a},\ \mathrm{quant} - \eta) + \mathrm{m(Mim,\ 1\ equiv)}}{\mathrm{m}(\mathbf{2a},\ \eta)} = \frac{(3.60\ \mathrm{g} - 3.21\ \mathrm{g}) + 0.84\ \mathrm{g}}{3.21\ \mathrm{g}} = \mathbf{0.38}$$

If we consider the loss of PIL during the regeneration procedure:

$$EF = \frac{\mathrm{m(waste)}}{\mathrm{m(product)}} = \frac{\mathrm{m}(\mathbf{2a},\ \mathrm{quant} - \eta) + \mathrm{m(Mim,\ 1\ equiv)} + \mathrm{m(loss\ PIL,}\ ca.\ 1\%)}{\mathrm{m}(\mathbf{2a},\ \eta)} = \frac{(3.60\ \mathrm{g} - 3.21\ \mathrm{g}) + 0.84\ \mathrm{g} + 0.12\ \mathrm{g}}{3.21\ \mathrm{g}} = \mathbf{0.42}$$

If we additionally estimate the 10 wt.-% loss of CH_2Cl_2 (DCM) during the extraction of the crude product:

$$EF = \frac{\mathrm{m(waste)}}{\mathrm{m(product)}} = \frac{\mathrm{m}(\mathbf{2a},\ \mathrm{quant} - \eta) + \mathrm{m(Mim,\ 1\ equiv)} + \mathrm{m(loss\ PIL,}\ ca.\ 1\%) + \mathrm{w(loss,\ DCM)} \times \mathrm{m(total,\ DCM)}}{\mathrm{m}(\mathbf{2a},\ \eta)} =$$

$$= \frac{(3.60\ \mathrm{g} - 3.21\ \mathrm{g}) + 0.84\ \mathrm{g} + 0.12\ \mathrm{g} + 0.1 \times 133\ \mathrm{g}}{3.21\ \mathrm{g}} = \mathbf{4.56}$$

The calculations for independent PIL regeneration process, including 10 wt.-% loss of solvents for washings, provide:

$$EF = \frac{\mathrm{m(waste)}}{\mathrm{m(product)}} = \frac{\mathrm{m(loss\ PIL,}\ ca.\ 1\%) + \mathrm{w(loss,\ EtOAc)} \times \mathrm{m(total,\ EtOAc)} + \mathrm{w(loss,\ DCM)} \times \mathrm{m(total,\ DCM)}}{\mathrm{m(regen.\ PIL)}} =$$

$$= \frac{0.12\ \mathrm{g} + 0.1 \times 180\ \mathrm{g} + 0.1 \times 266\ \mathrm{g}}{10.95\ \mathrm{g}} = \mathbf{4.08}$$

These preliminary estimations show that for the described processes, calculated E-factors lie in the range of bulk chemicals production (EF < 1–5).[S41]

References

[S1] T. G. G. Battye, L. Kontogiannis, O. Johnson, H. R. Powell, A. G. W. Leslie, *Acta Cryst.* **2011**, *D67,* 271–281.
[S2] Bruker, *APEX-III*. Bruker AXS Inc., Madison, Wisconsin, USA, **2018**.
[S3] P. Evans, *Acta Cryst.* **2006**, *D62,* 72–82.
[S4] L. Krause, R. Herbst-Irmer, G. M. Sheldrick, D. Stalke, *J. Appl. Cryst.* **2015**, *48*, 3-10.
[S5] G. M. Sheldrick, *Acta Cryst.* **2015**, *A71*, 3–8.
[S6] M. M. Vadiyar, S. K. Patil, S. C. Bhise, A. V. Ghule, S.-H. Han, S. S. Kolekar, *Eur. J. Inorg. Chem.* **2015**, 5832–5838.
[S7] M. E. Coddens, K. G. Furton, C. F. Poole, *J. Chromatogr.* **1986**, *356*, 59–77.
[S8] G. D′Andola, L. Szarvas, K. Massonne, V. Stegmann, US2010/0048829 A1, **2010**.
[S9] E. J. Corey, M. Chaykovsky, *J. Am. Chem. Soc.* **1965**, *87*, 1353–1364.
[S10] W. Fraser, C. J. Suckling, H. C. S. Wood, *J. Chem. Soc., Perkin Trans. 1* **1990**, 3137–3144.
[S11] P. Berton, S. P. Kelley, H. Wang, R. D. Rogers, *J. Mol. Liq.* **2018**, *269*, 126–131.
[S12] S. Zhang, K. Cheng, X. Wang, H. Yin, *Bioorg. Med. Chem.* **2012**, *20*, 6073–6079.
[S13] Z.-J. Jiang, Z.-H. Li, J.-B. Yu, W.-K. Su, *J. Org. Chem.* **2016**, *81*, 10049–10055.
[S14] B. Gregory, W. Hinz, R. A. Jones, J. S. Arques, *J. Chem. Res. (M)* **1984**, 2801–2821.
[S15] R. F. C. Brown, F. W. Eastwood, K. J. Harrington, *Aust. J. Chem.* **1974**, *27*, 2373–2384.
[S16] E. Fillion, S. Carret, L. G. Mercier, V. E. Trepanier, *Org. Lett.* **2008**, *10*, 437–440.
[S17] F. von Nussbaum, V. M.-J. Li, S. Allerheiligen, S. Anlauf, L. Bärfacker, M. Bechem, M. Delbeck, M. F. Fitzgerald, M. Gerisch, H. Gielen-Haertwig, H. Haning, D. Karthaus, D. Lang, K. Lustig, D. Meibom, J. Mittendorf, U. Rosentreter, M. Schäfer, S. Schäfer, J. Schamberger, L. A. Telan, A. Tersteegen, *ChemMedChem* **2015**, *10*, 1163–1173.
[S18] F. Gonzalez-Bobes, M. D. B. Fenster, S. Kiau, L. Kolla, S. Kolotuchin, M. Soumeillant, *Adv. Synth. Catal.* **2008**, *350*, 813–816.
[S19] O. A. Ivanova, E. M. Budynina, A. O. Chagarovskiy, I. V. Trushkov, M. Ya. Melnikov, *J. Org. Chem.* **2011**, *76*, 8852–8868.
[S20] K. L. Ivanov, E. V. Villemson, E. M. Budynina, O. A. Ivanova, I. V. Trushkov, M. Ya. Melnikov, *Chem. – Eur. J.* **2015**, *21*, 4975–4987.
[S21] A. S. Pavlova, O. A. Ivanova, A. O. Chagarovskiy, N. S. Stebunov, N. V. Orlov, A. N. Shumsky, E. M. Budynina, V. B. Rybakov, I. V. Trushkov, *Chem. – Eur. J.* **2016**, *22*, 17967–17971.
[S22] A. O. Chagarovskiy, O. A. Ivanova, E. R. Rakhmankulov, E. M. Budynina, I. V. Trushkov, M. Ya. Melnikov, *Adv. Synth. Catal.* **2010**, *352*, 3179–3184.
[S23] A. O. Chagarovskiy, V. V. Kuznetsov, O. A. Ivanova, A. S. Goloveshkin, I. I. Levina, N. N. Makhova, I. V. Trushkov, *Eur. J. Org. Chem.* **2019**, 5475–5485.
[S24] K. Sapeta, M. A. Kerr, *Org. Lett.* **2009**, *11*, 2081–2084.
[S25] O. A. Ivanova, E. M. Budynina, D. A. Skvortsov, M. Limoge, A. V. Bakin, A. O. Chagarovskiy, I. V. Trushkov, M. Ya. Melnikov, *Chem. Commun.* **2013**, *49*, 11482–11484.
[S26] H.-H. Zhang, Y.-C. Luo, H.-P. Wang, W. Chen, P.-F. Xu, *Org. Lett.* **2014**, *16*, 4896–4899.
[S27] O. A. Ivanova, V. A. Andronov, V. S. Vasin, A. N. Shumsky, V. B. Rybakov, L. G. Voskressensky, I. V. Trushkov, *Org. Lett.* **2018**, *20*, 7947–7952.
[S28] F. de Nanteuil, J. Waser, *Angew. Chem.* **2011**, *123*, 12281–12285; *Angew. Chem. Int. Ed.* **2011**, *50*, 12075–12079.
[S29] F. de Nanteuil, E. Serrano, D. Perrotta, J. Waser, *J. Am. Chem. Soc.* **2014**, *136*, 6239–6242.
[S30] R. Talukdar, D. P. Tiwari, A. Saha, M. K. Ghorai, *Org. Lett.* **2014**, *16*, 3954–3957.
[S31] O. A. Ivanova, E. M. Budynina, A. O. Chagarovskiy, A. E. Kaplun, I. V. Trushkov, M. Ya. Melnikov, *Adv. Synth. Catal.* **2011**, *353*, 1125–1134.
[S32] A. T. Parsons, M. J. Campbell, J. S. Johnson, *Org. Lett.* **2008**, *10*, 2541–2544.
[S33] Y. Matsumoto, D. Nakatake, R. Yazaki, T. Ohshima, *Chem. – Eur. J.* **2018**, *24*, 6062–6066.
[S34] O. A. Ivanova, A. O. Chagarovskiy, A. N. Shumsky, V. D. Krasnobrov, I. I. Levina, I. V. Trushkov, *J. Org. Chem.* **2018**, *83*, 543–560.
[S35] H. B. Tukhtaev, K. L. Ivanov, S. I. Bezzubov, D. A. Cheshkov, M. Ya. Melnikov, E. M. Budynina, *Org. Lett.* **2019**, *21*, 1087–1092.
[S36] V. S. Korotkov, O. V. Larionov, A. Hofmeister, J. Magull, A. de Meijere, *J. Org. Chem.* **2007**, *72*, 7504–7510.
[S37] A. A. Fadeev, A. O. Chagarovskiy, A. S. Makarov, I. I. Levina, O. A. Ivanova, M. G. Uchuskin, I. V. Trushkov, *Molecules* **2020**, *25*, art. 5748.
[S38] G. Aghapour, R. Hatefipour, *Synth. Commun.* **2009**, *39*, 1698–1707.
[S39] J. Etxabe, J. Izquierdo, A. Landa, M. Oiarbide, C. Palomo, *Angew. Chem.* **2015**, *127*, 6987–6990; *Angew. Chem. Int. Ed.* **2015**, *54*, 6883–6886.
[S40] K. S. Kozlov, L. V. Romashov, V. P. Ananikov, *Green Chem.* **2019***, 21,* 3464–3468.
[S41] R. A. Sheldon, *Green Chem.* **2007**, *9*, 1273–1283.

Copies of NMR spectra

1-Methylimidazolium thiocyanate (HMimNCS, 3a)

^{1}H NMR (500 MHz, DMSO-d_6)

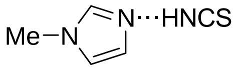

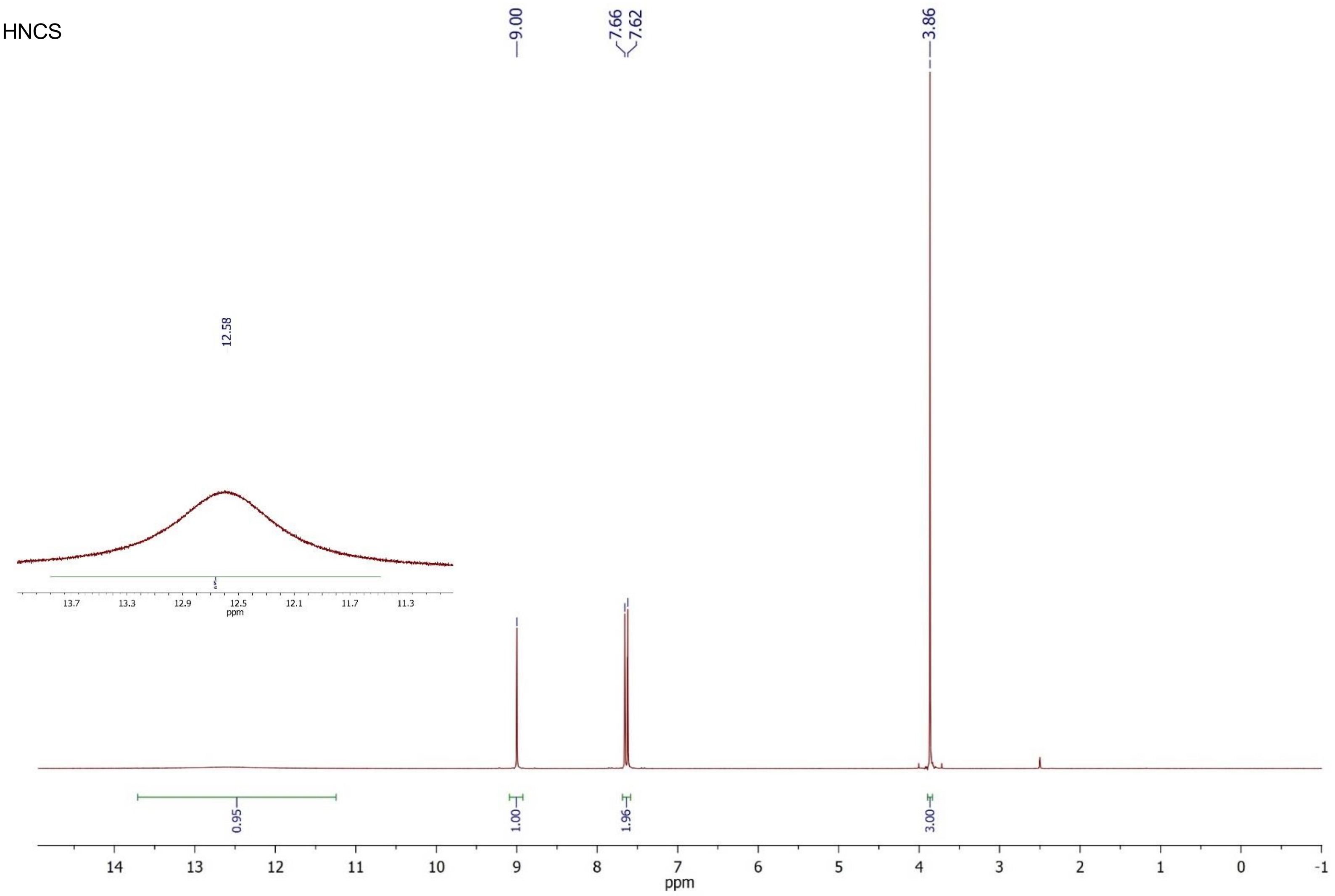

1-Methylimidazolium thiocyanate (HMimNCS, 3a)

^{13}C NMR (126 MHz, DMSO-d$_6$)

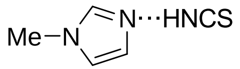

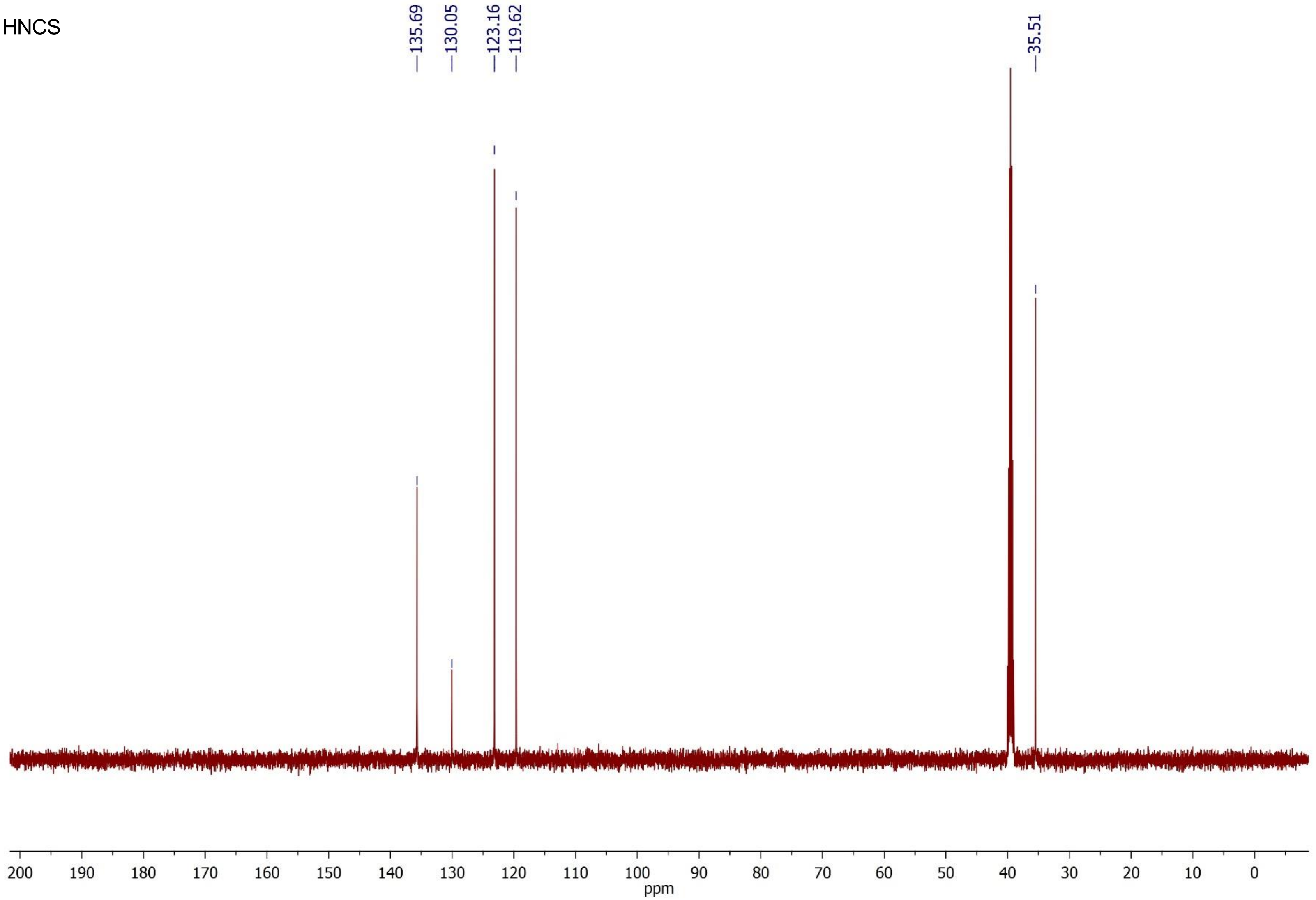

Triethylammonium thiocyanate ($Et_3NH^+NCS^-$, 3b)

^{1}H NMR (500 MHz, $CDCl_3$)

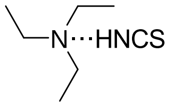

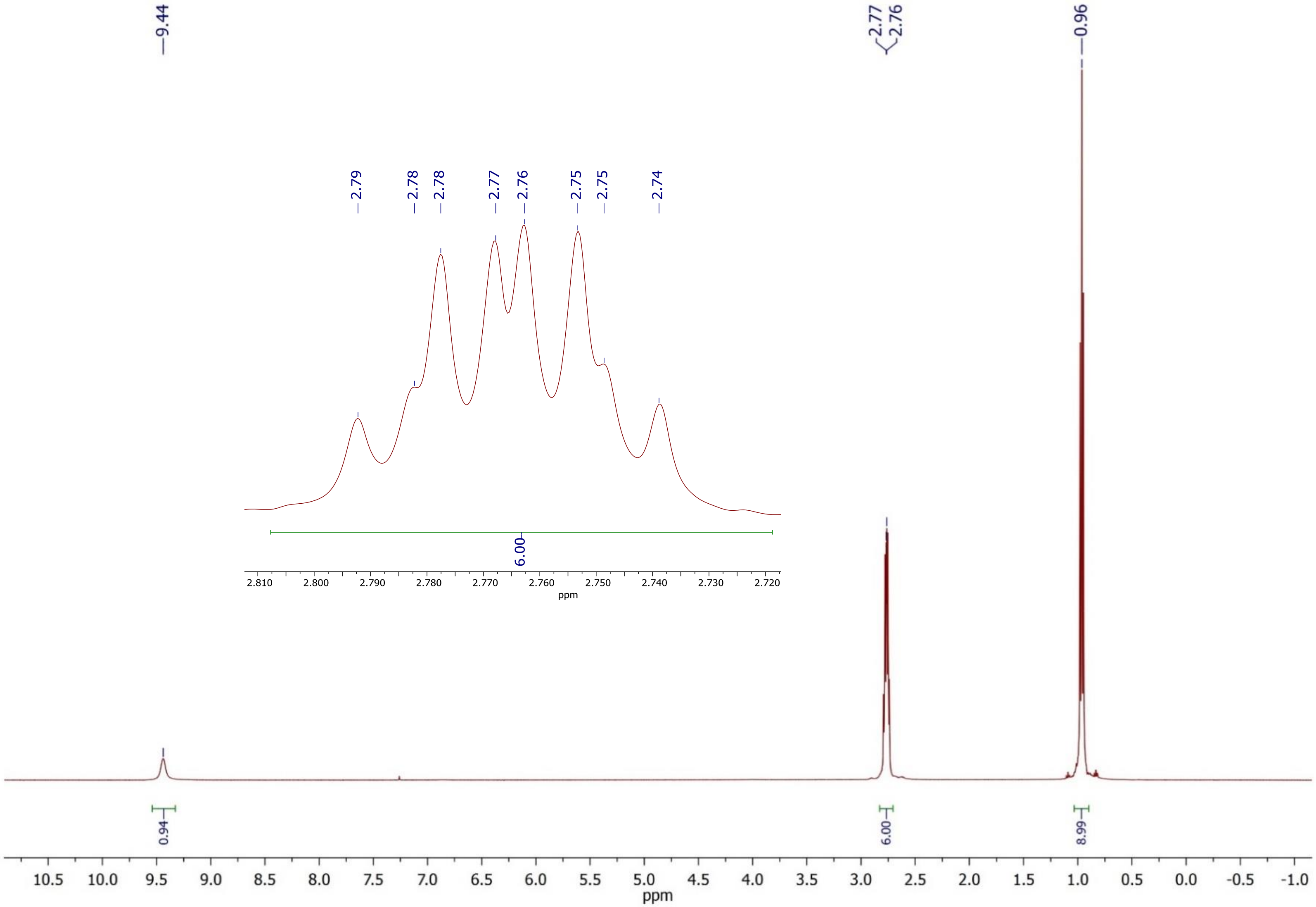

Triethylammonium thiocyanate ($Et_3NH^+NCS^-$, 3b)

^{13}C NMR (126 MHz, $CDCl_3$)

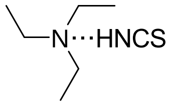

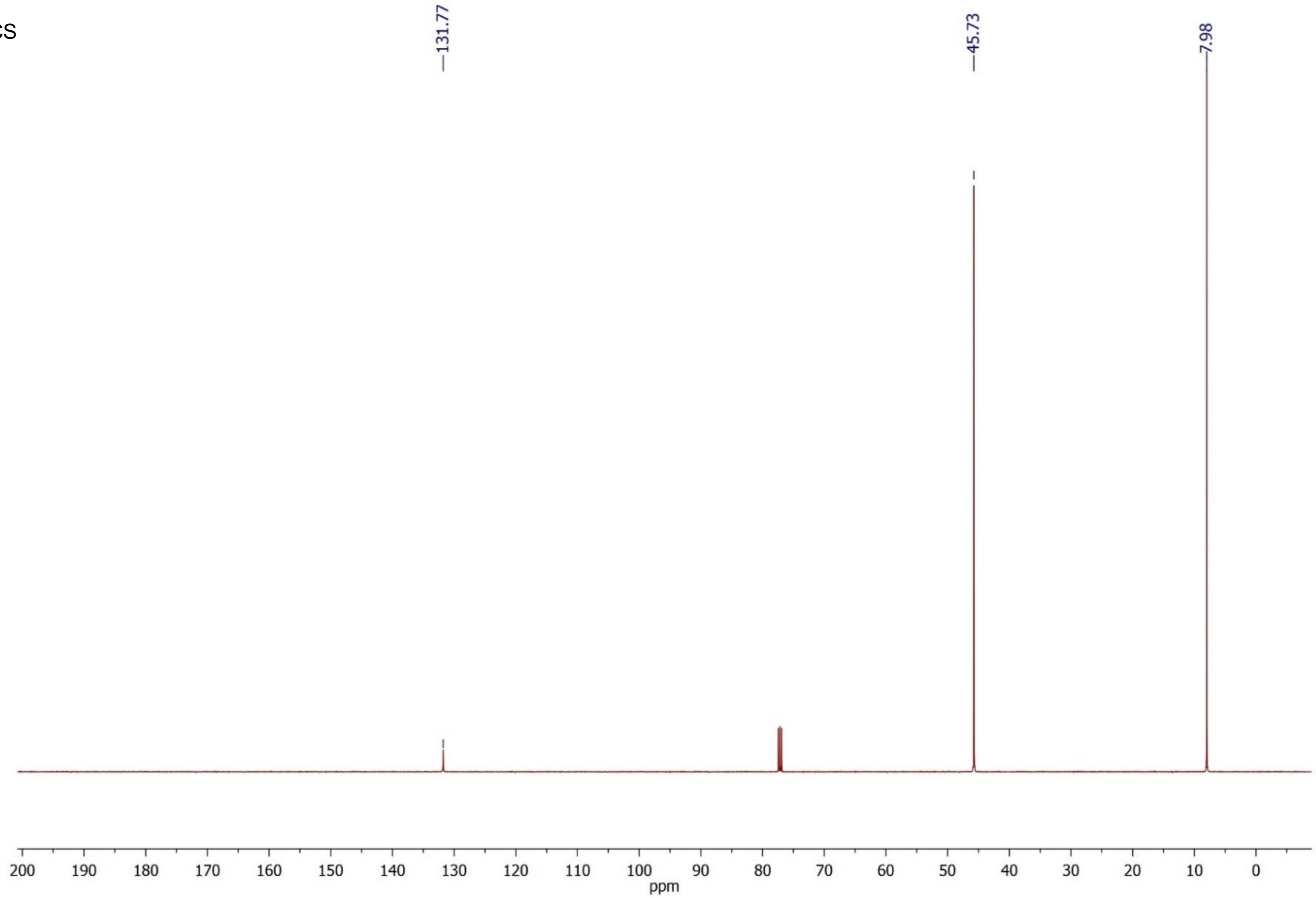

***N*,*N*,*N′*,*N′*-Tetramethylguanidinium thiocyanate (HTmgNCS, 3c)**

^{1}H NMR (500 MHz, $CDCl_3$)

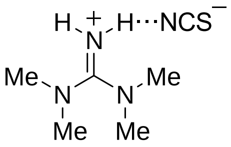

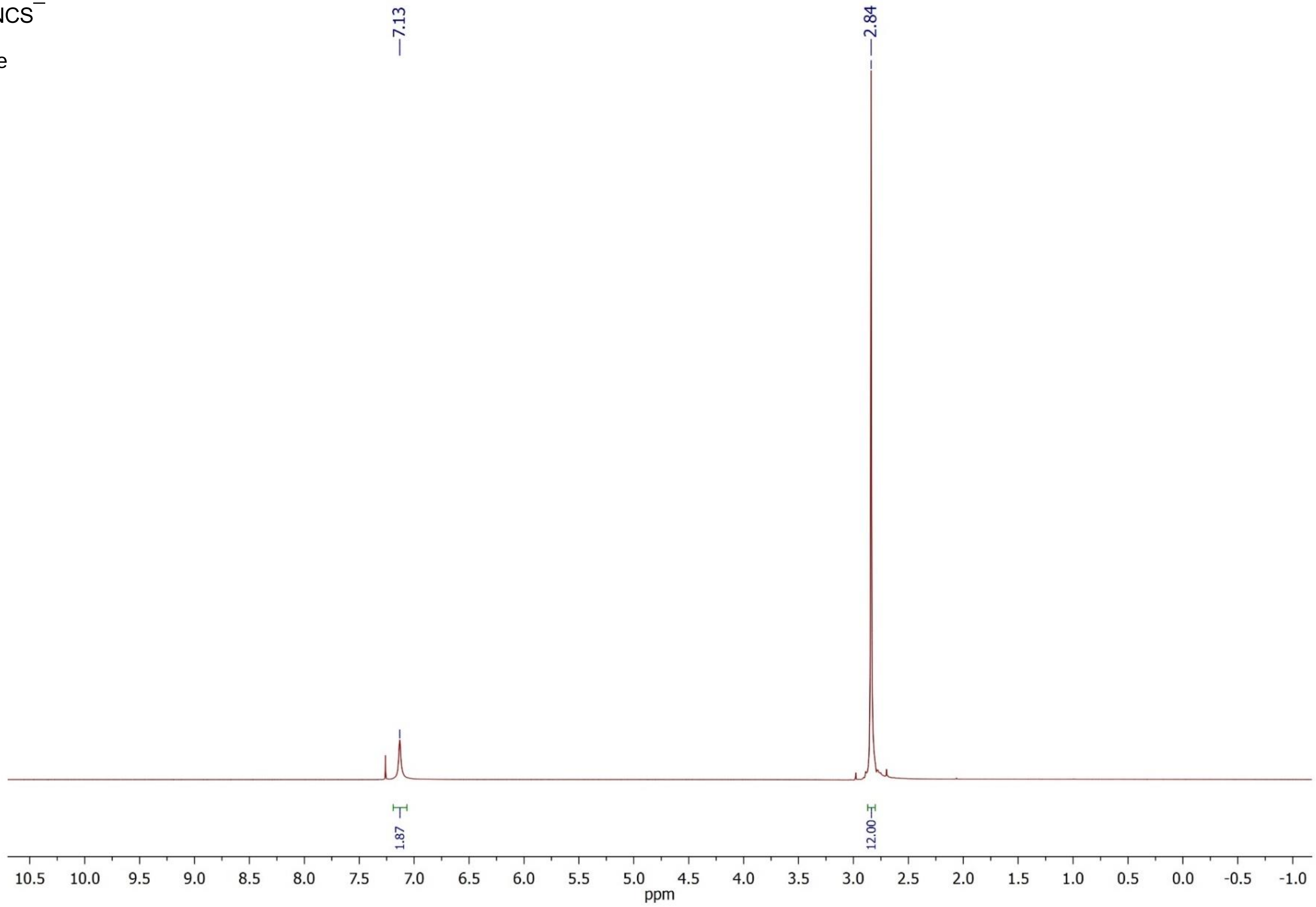

***N*,*N*,*N′*,*N′*-Tetramethylguanidinium thiocyanate (HTmgNCS, 3c)**

^{13}C NMR (126 MHz, $CDCl_3$)

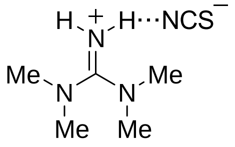

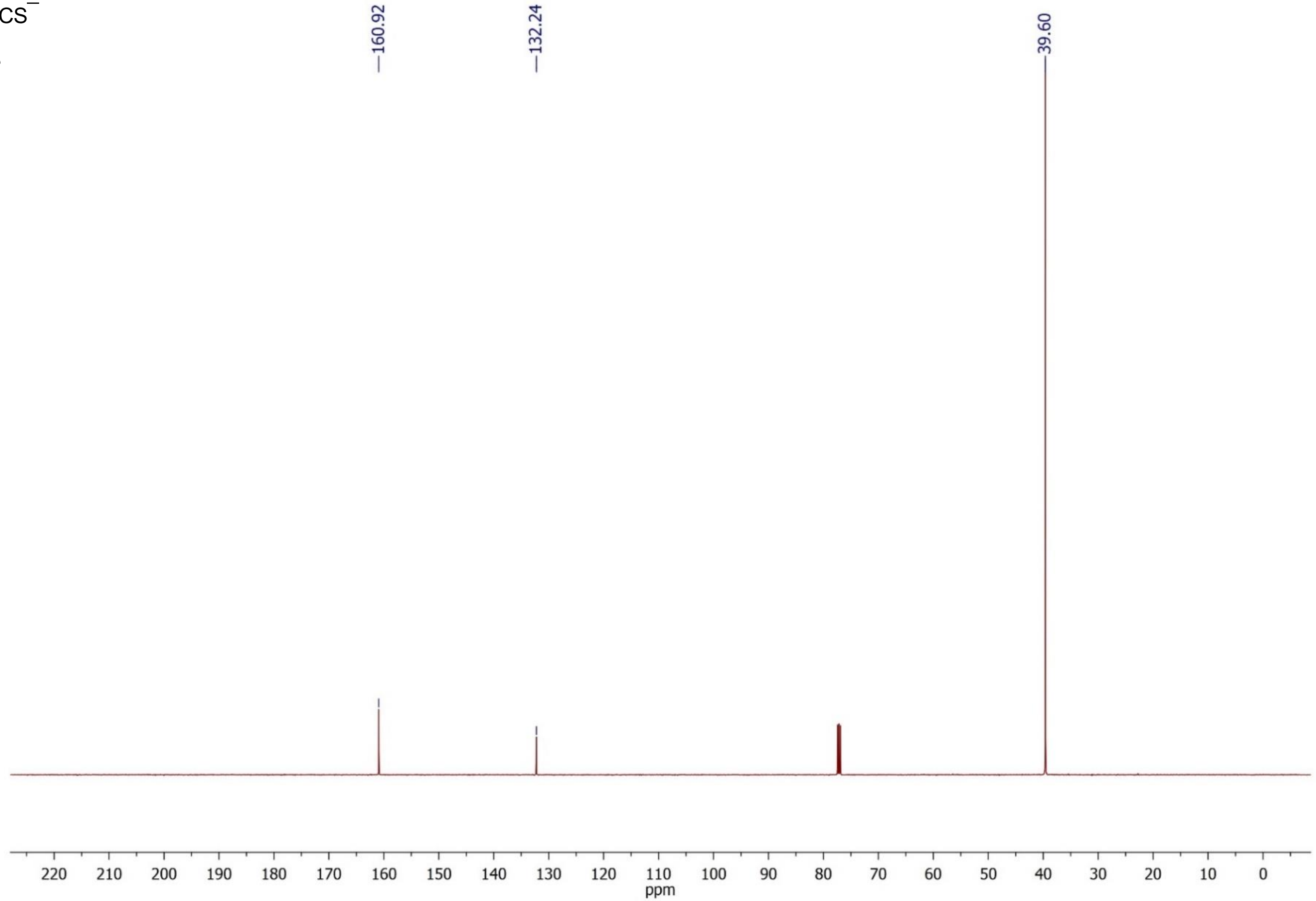

Dimethyl 2-(2,4-dimethoxybenzylidene)malonate (S1f)

^1H NMR (500 MHz, $CDCl_3$)

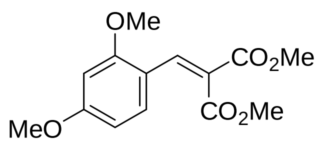

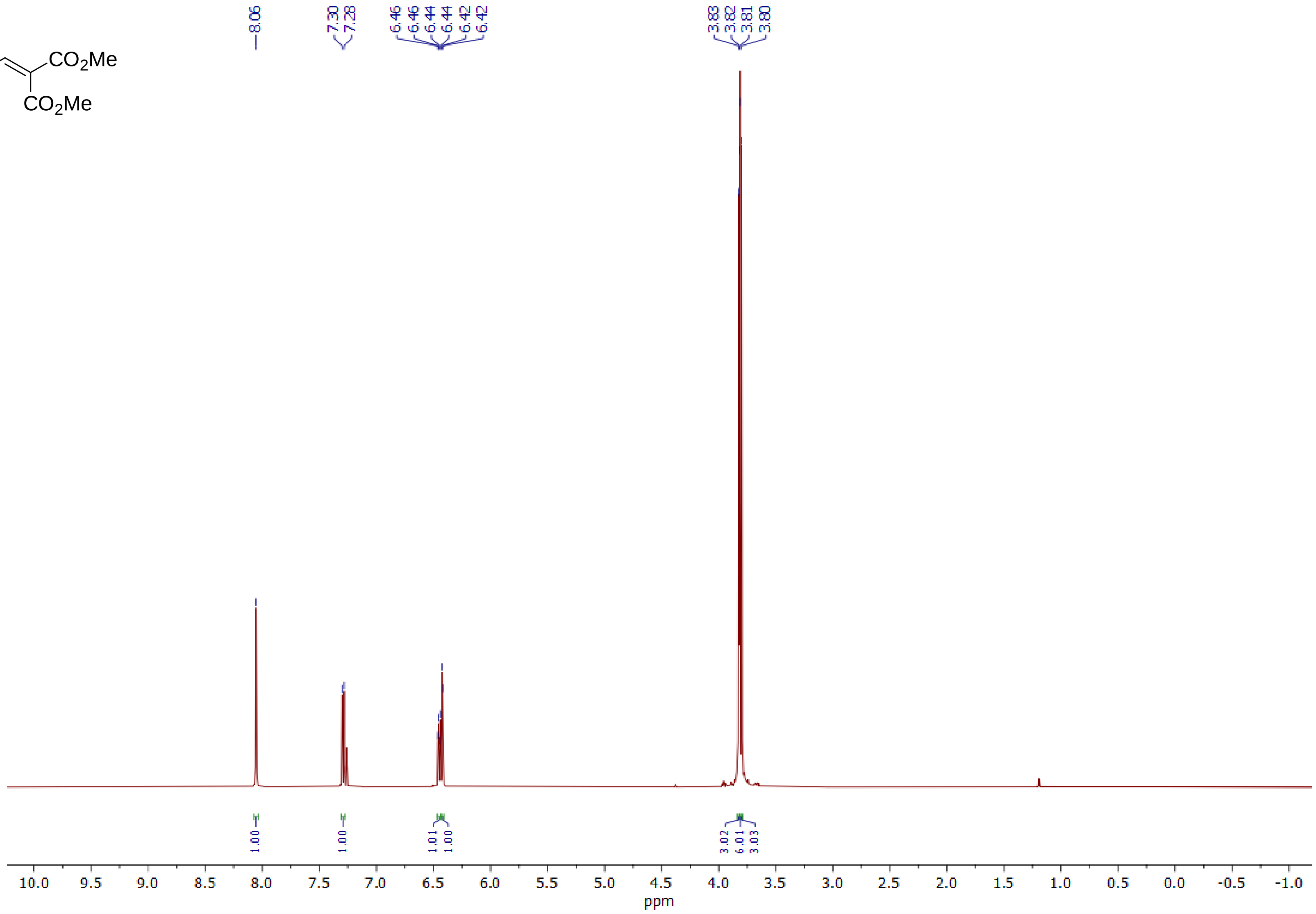

Dimethyl 2-(2,4-dimethoxybenzylidene)malonate (S1f)

^{13}C NMR (126 MHz, $CDCl_3$)

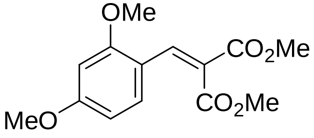

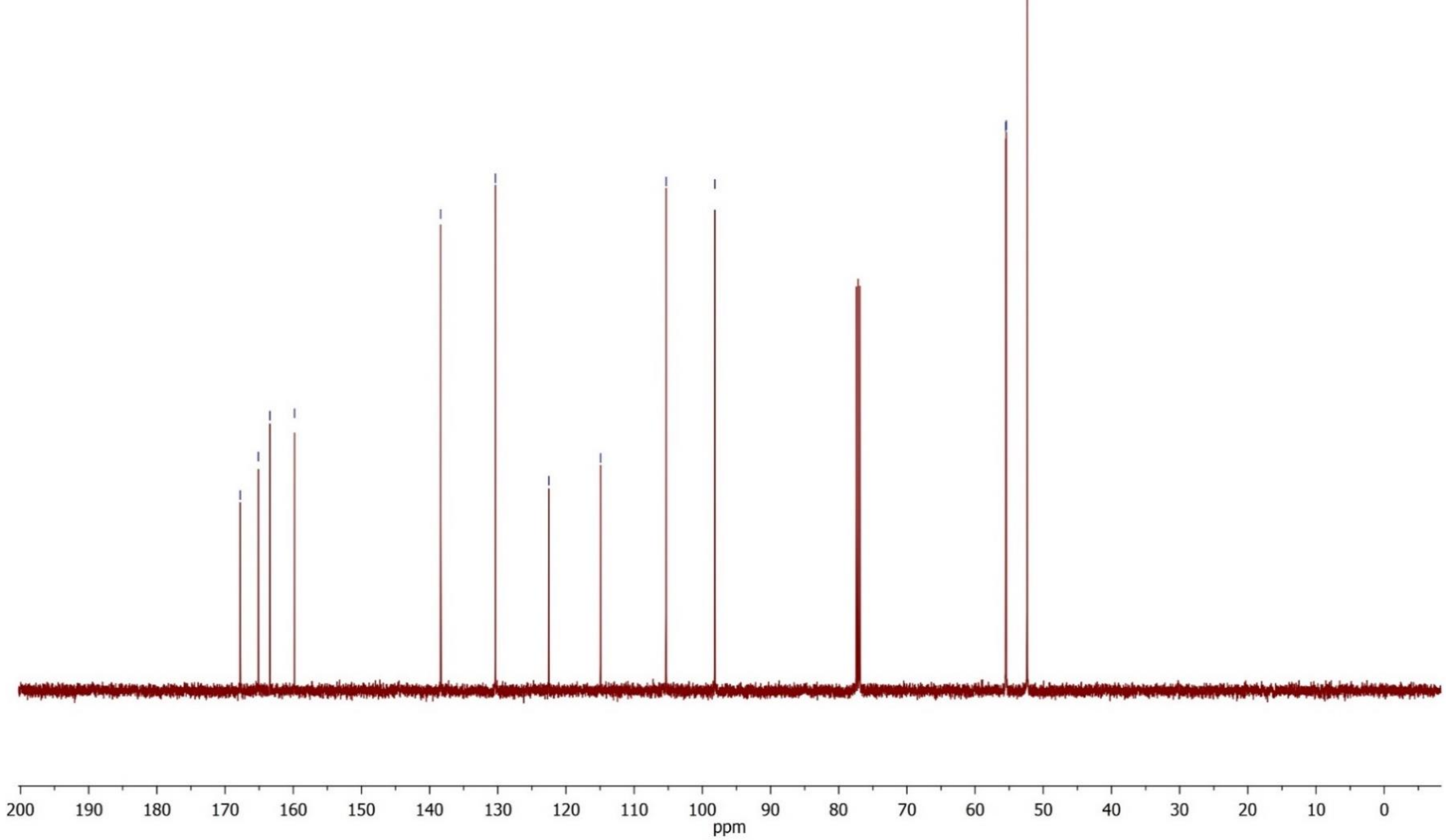

Dimethyl 2-(2,6-dimethoxybenzylidene)malonate (S1k)

^1H NMR (500 MHz, $CDCl_3$)

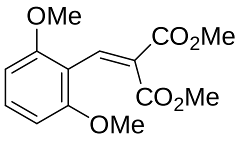

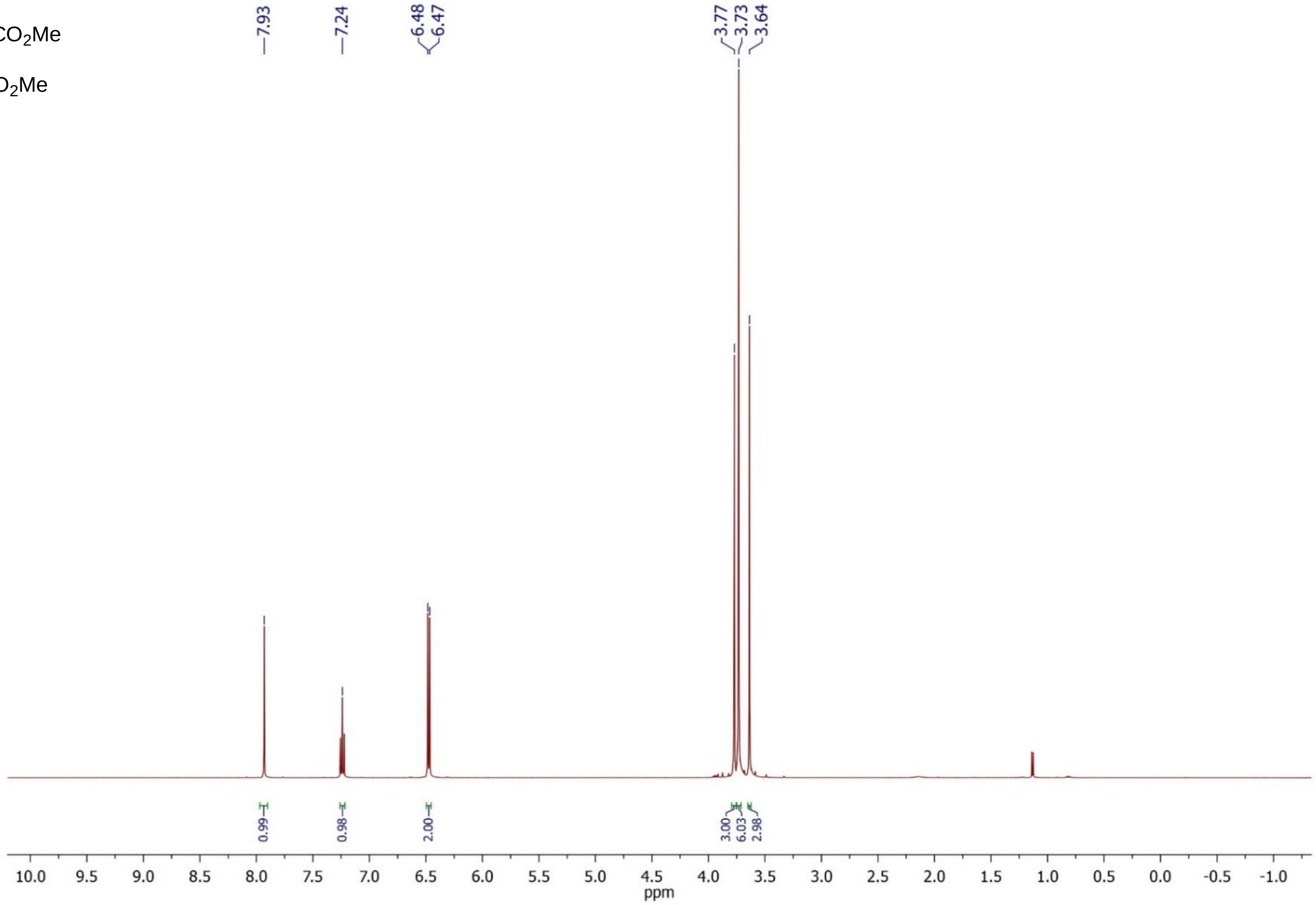

Dimethyl 2-(2,6-dimethoxybenzylidene)malonate (S1k)

^{13}C NMR (126 MHz, $CDCl_3$)

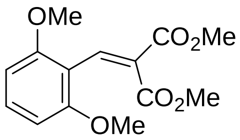

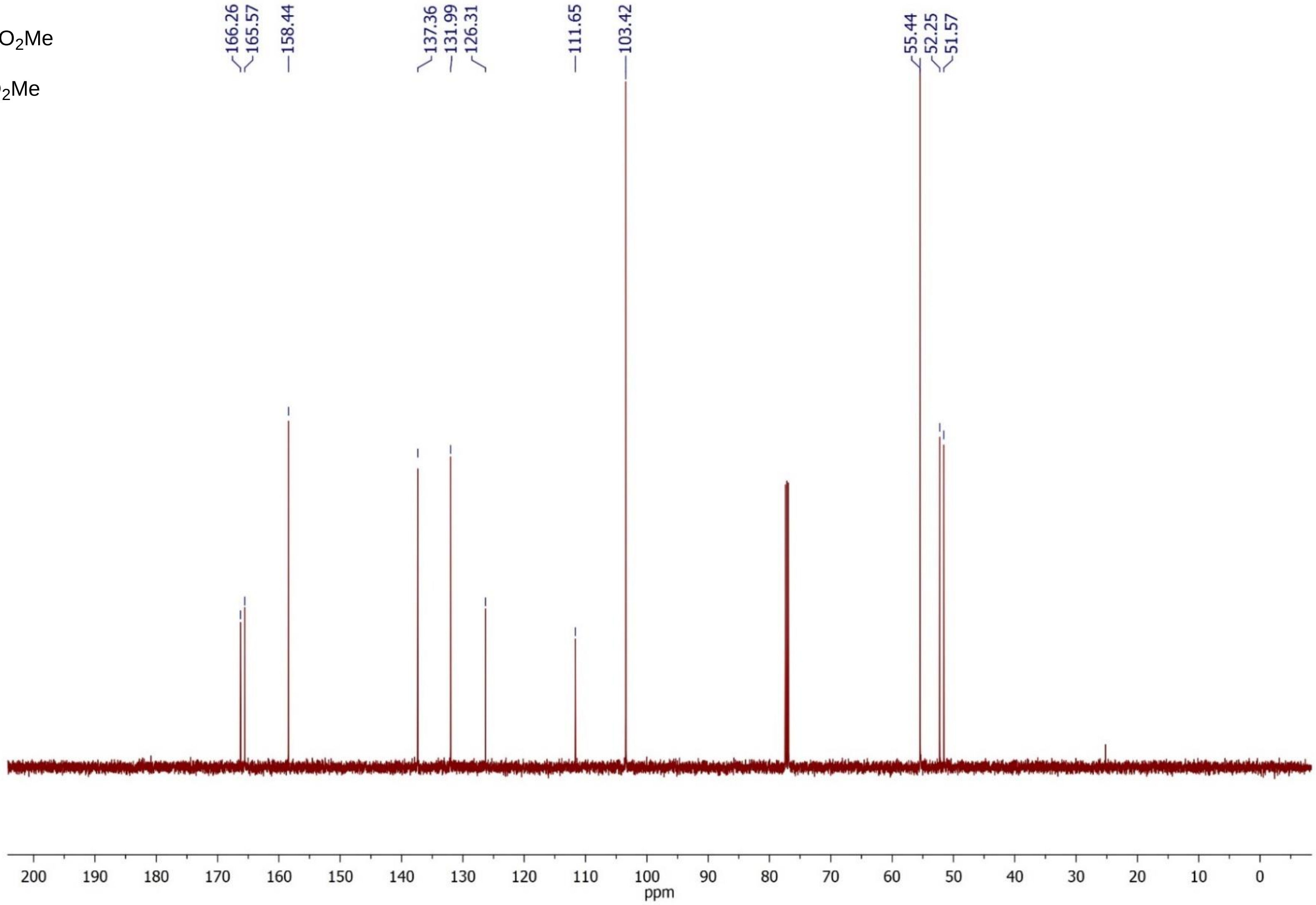

Dimethyl 2-(4-dimethylamino-2-nitrobenzylidene)malonate (S1n)

^1H NMR (500 MHz, $CDCl_3$)

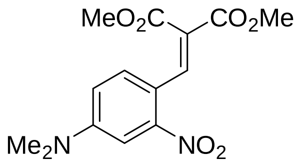

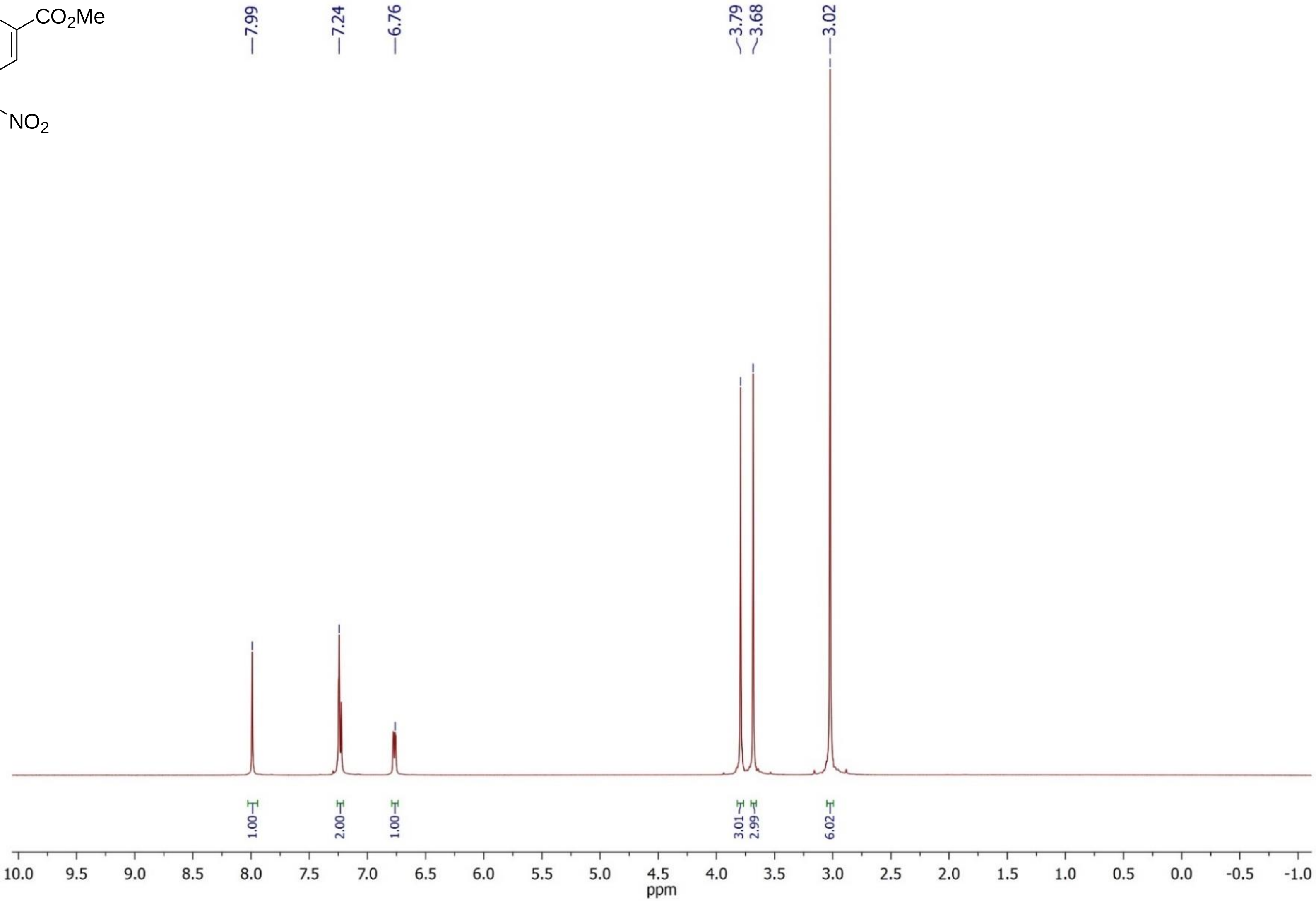

Dimethyl 2-(4-dimethylamino-2-nitrobenzylidene)malonate (S1n)

^{13}C NMR (126 MHz, $CDCl_3$)

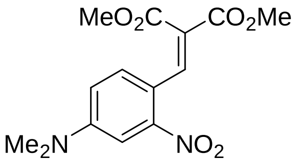

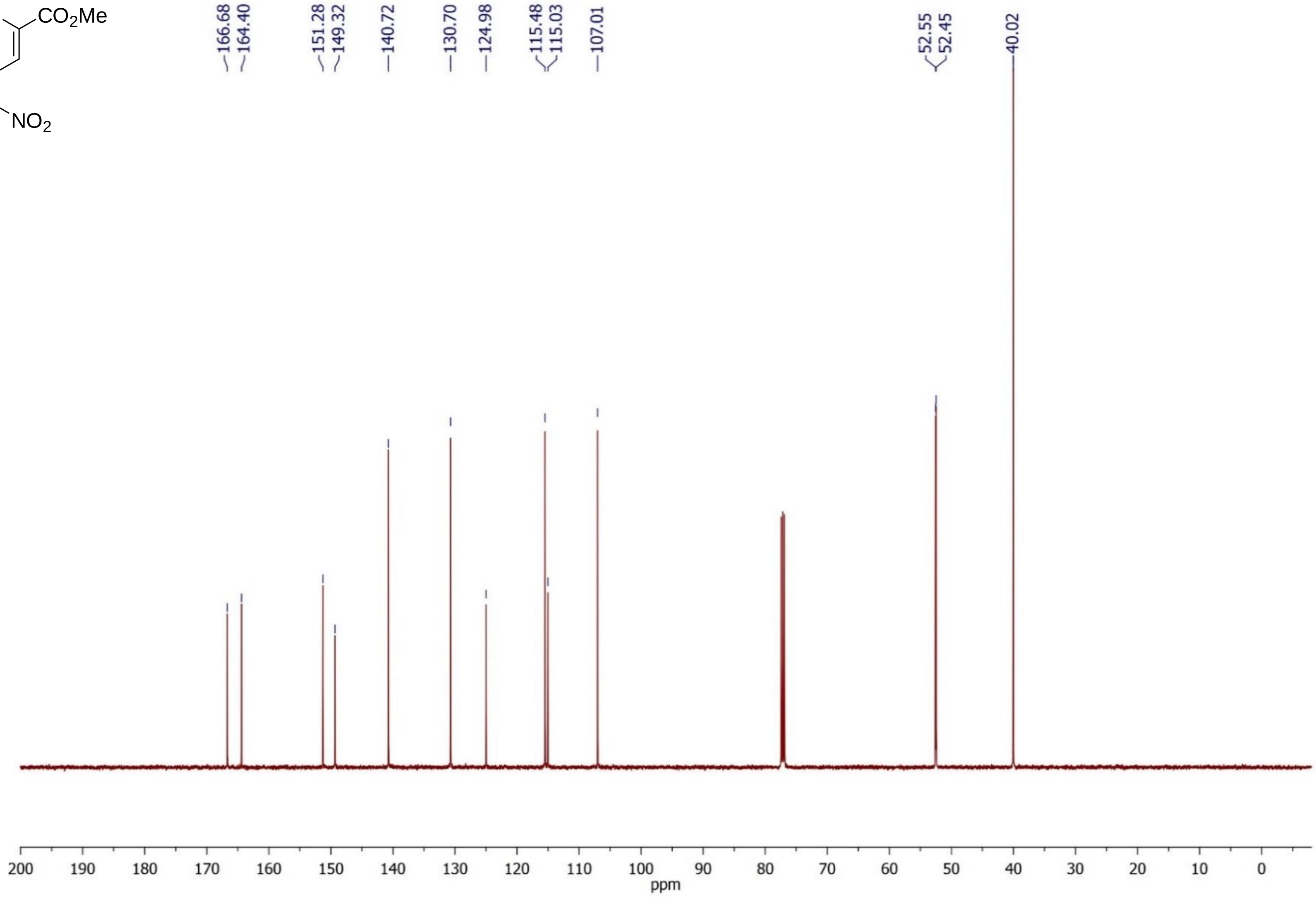

Dimethyl 2-{[4′-methoxy-(1,1′-biphenyl)-4-yl]methylene}malonate (S1o)

^{1}H NMR (500 MHz, $CDCl_3$)

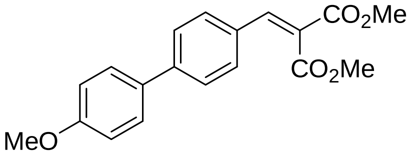

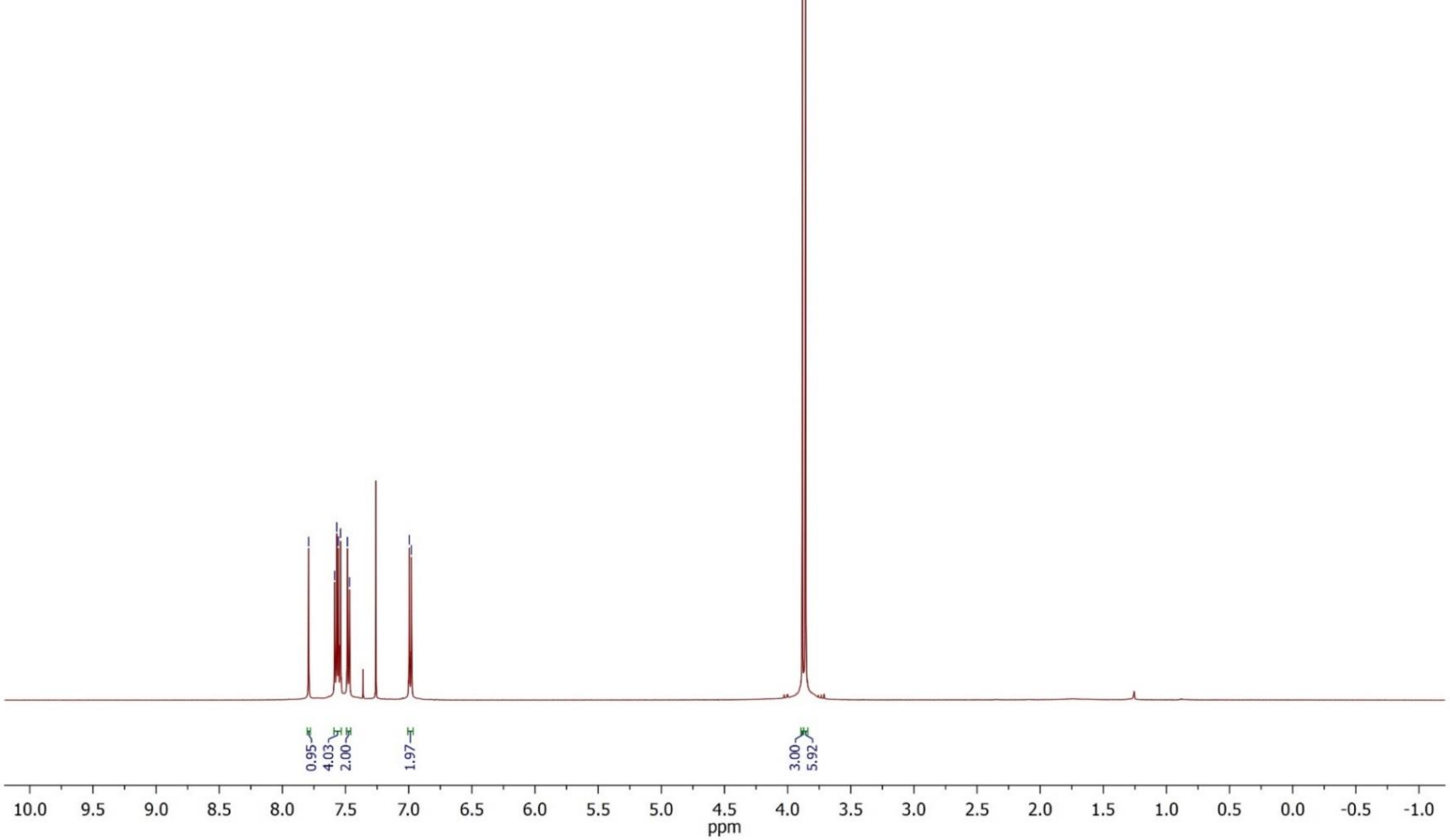

Dimethyl 2-{[4′-methoxy-(1,1′-biphenyl)-4-yl]methylene}malonate (S1o)

^{13}C NMR (126 MHz, $CDCl_3$)

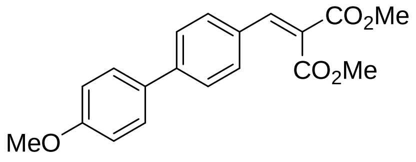

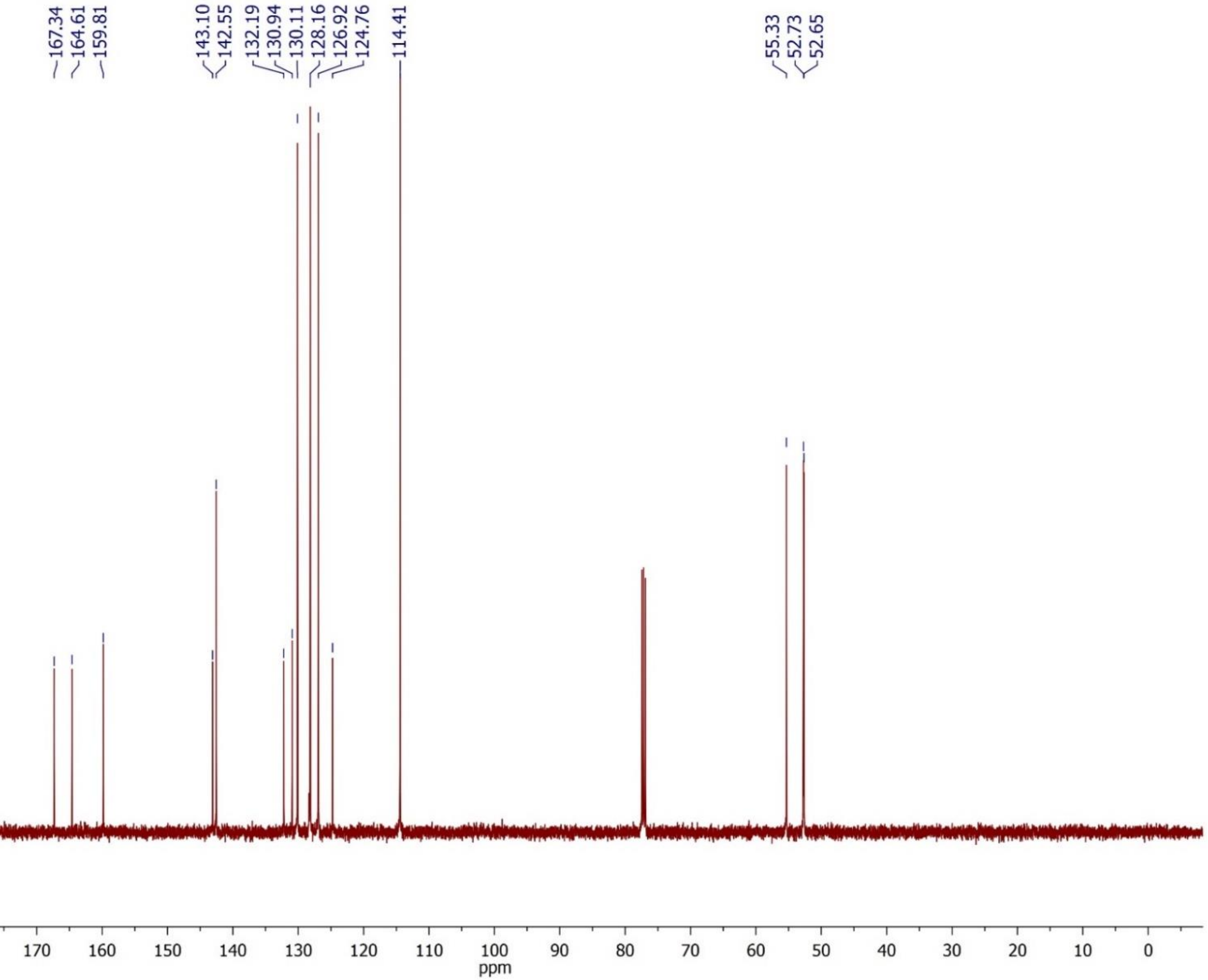

Dimethyl 2-{[4′-methoxy-(1,1′-biphenyl)-4-yl]methylene}malonate (S1o)

^{1}H–^{13}C HSQC ($CDCl_3$)

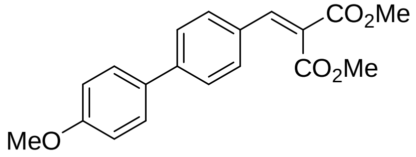

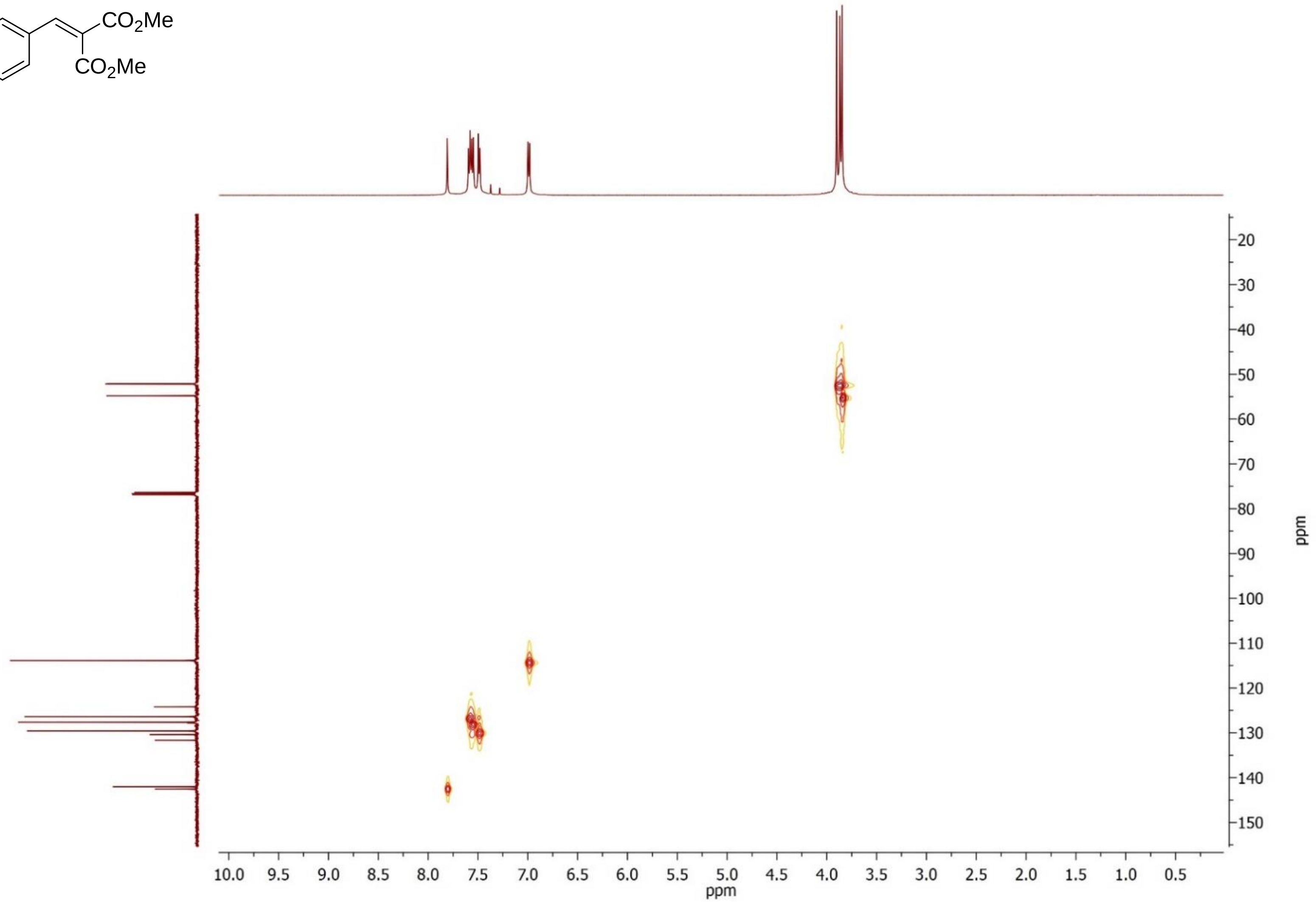

Dimethyl 2-[(6-methoxynaphthalen-2-yl)methylene]malonate (S1p)

^{1}H NMR (500 MHz, $CDCl_3$)

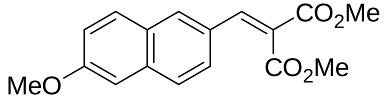

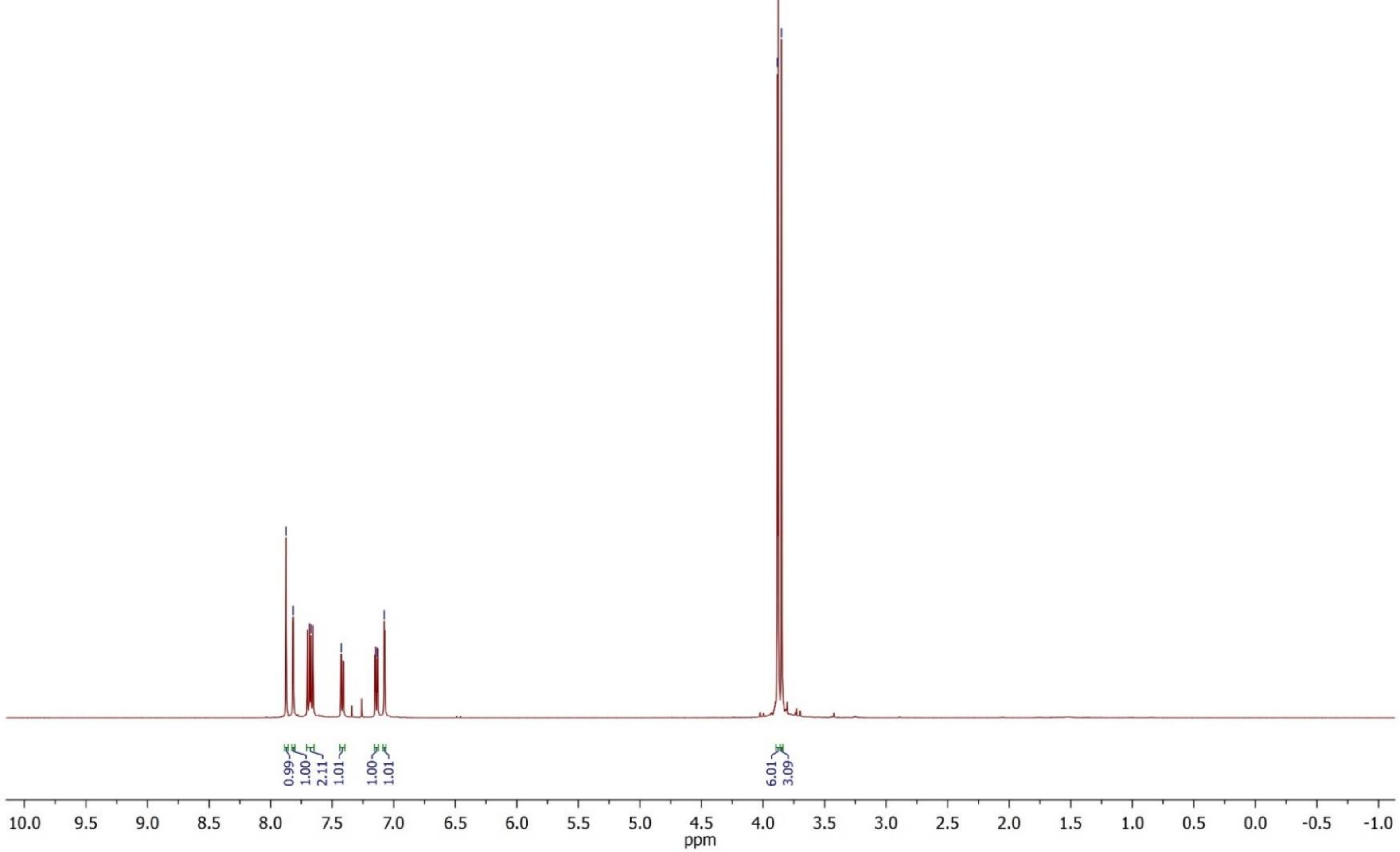

Dimethyl 2-[(6-methoxynaphthalen-2-yl)methylene]malonate (S1p)

^{13}C NMR (126 MHz, $CDCl_3$)

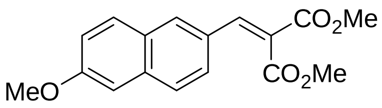

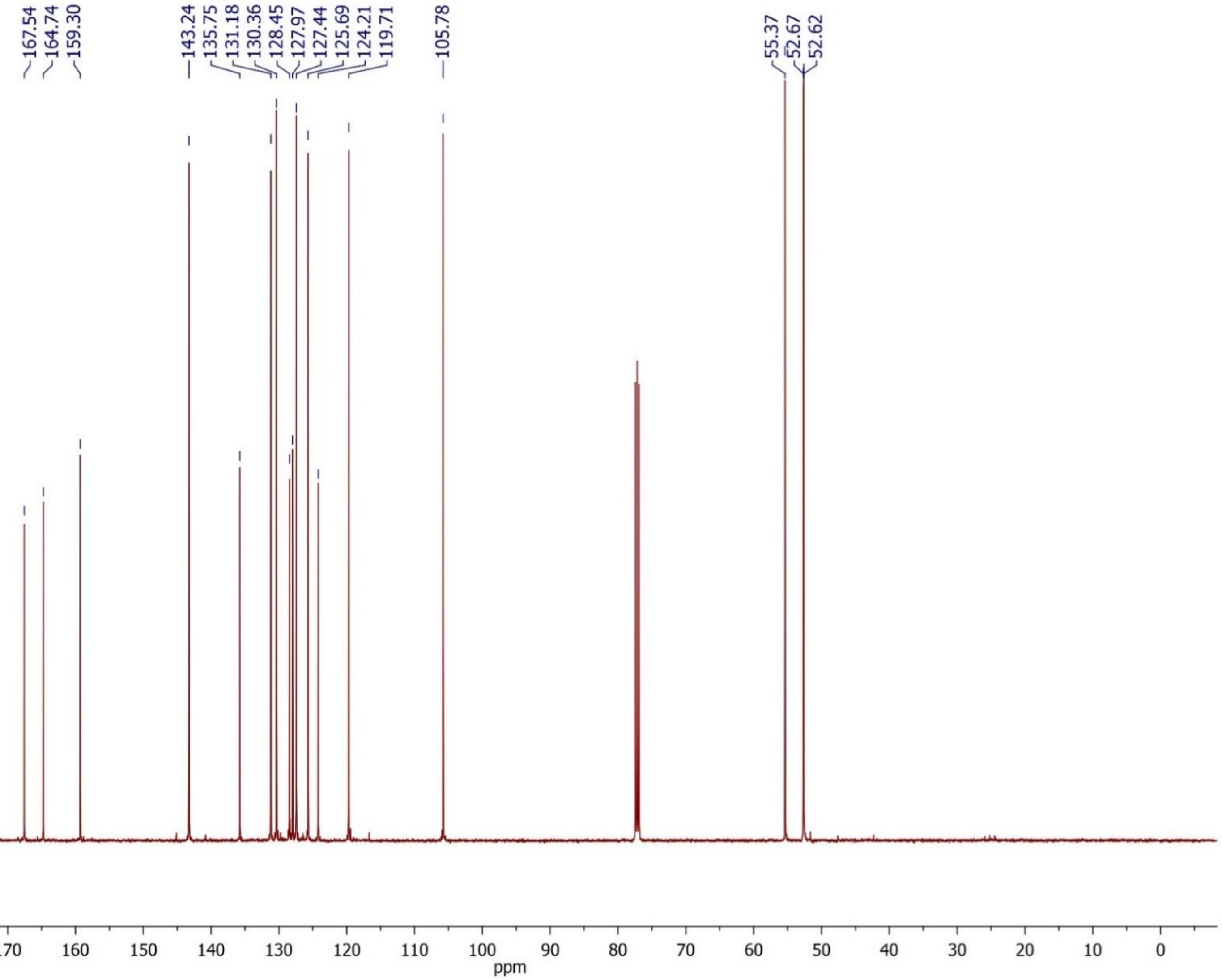

Dimethyl 2-[(1-methyl-1*H*-pyrrol-2-yl)methylene]malonate (S1v)

^{1}H NMR (500 MHz, $CDCl_3$)

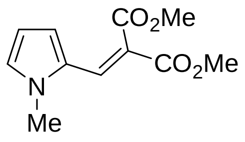

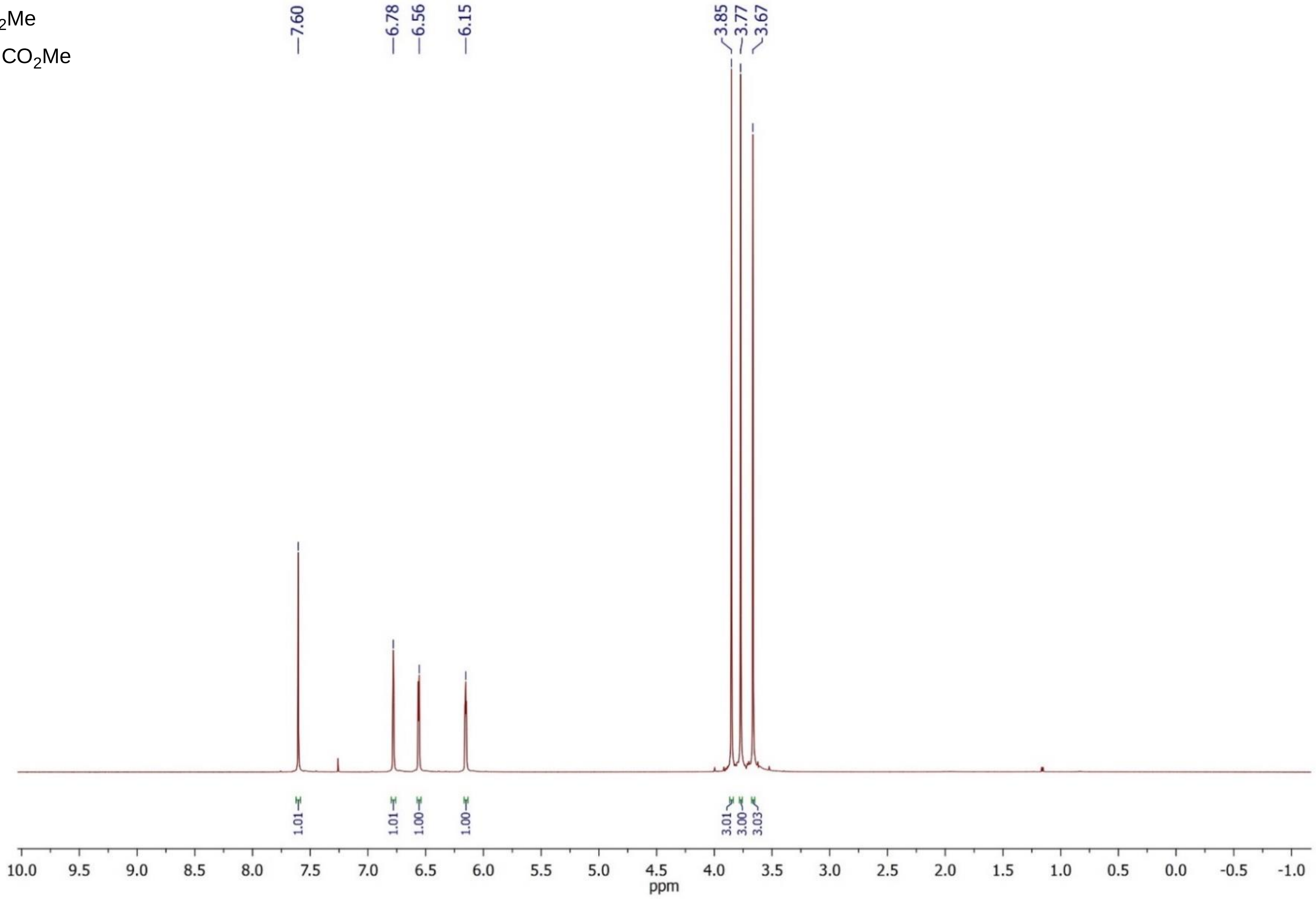

Dimethyl 2-[(1-methyl-1*H*-pyrrol-2-yl)methylene]malonate (S1v)

^{13}C NMR (126 MHz, $CDCl_3$)

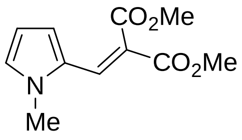

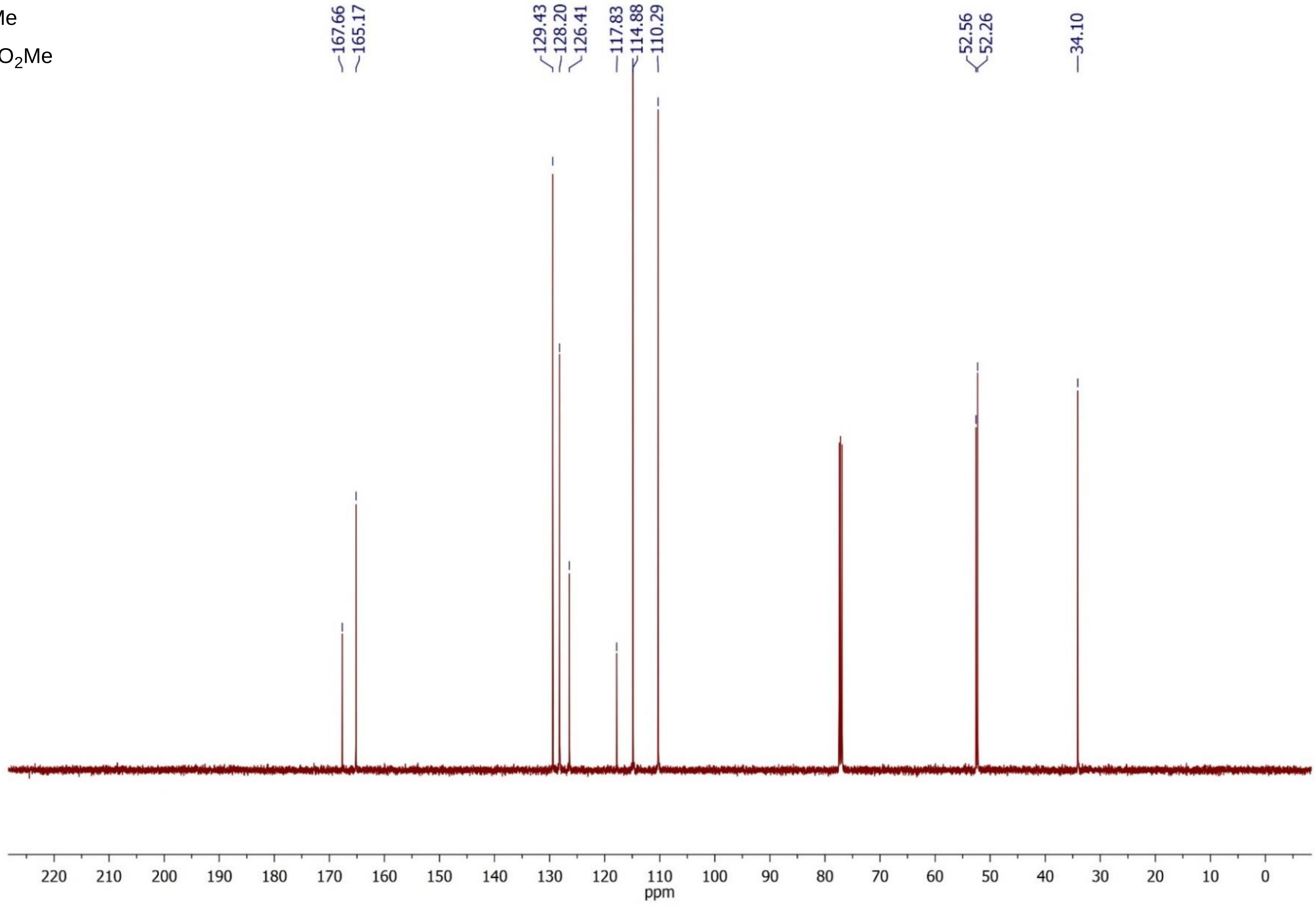

Dimethyl 2-[(1-methyl-1*H*-pyrrol-2-yl)methylene]malonate (S1v)

^{1}H–^{13}C HSQC ($CDCl_3$)

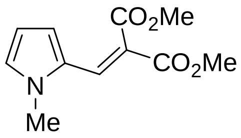

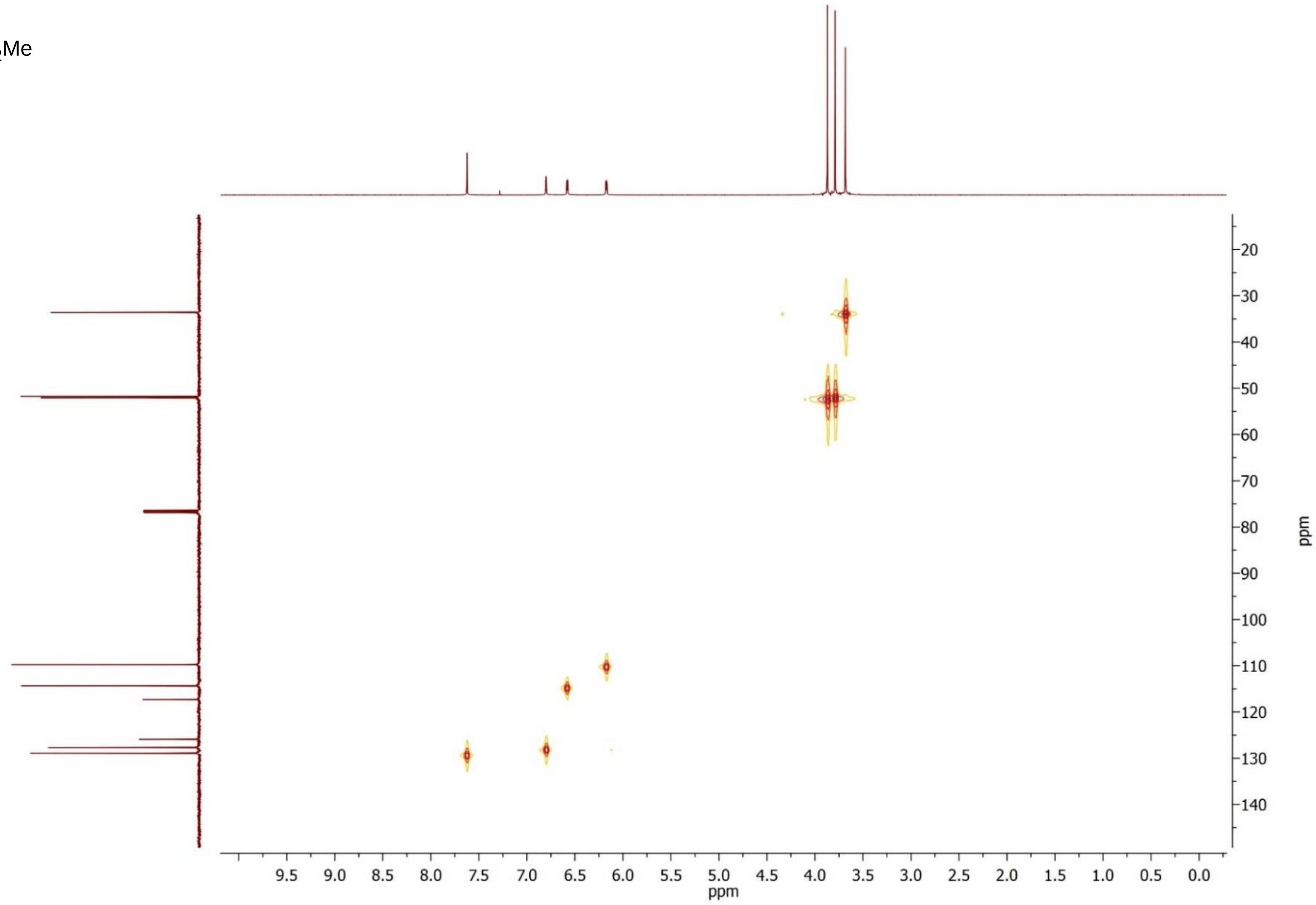

Dimethyl 2-[(1-methyl-1*H*-pyrrol-2-yl)methylene]malonate (S1v)

^{1}H–^{13}C HMBC ($CDCl_3$)

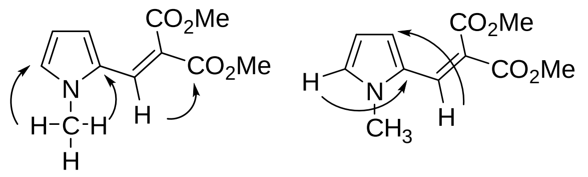

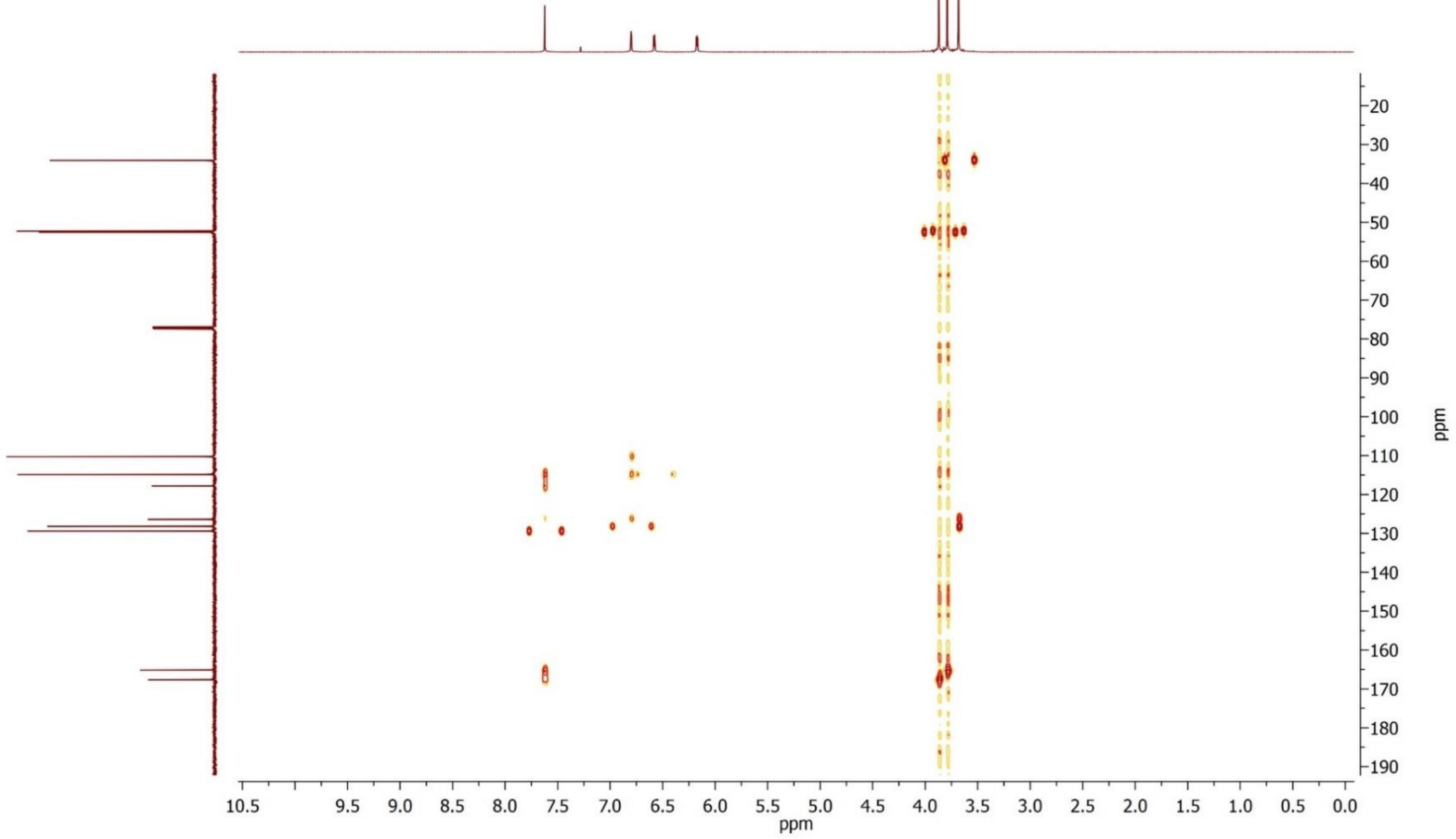

Dimethyl 2-[(ferrocenyl)methylene]malonate (S1x)

^{1}H NMR (500 MHz, $CDCl_3$)

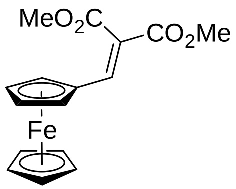

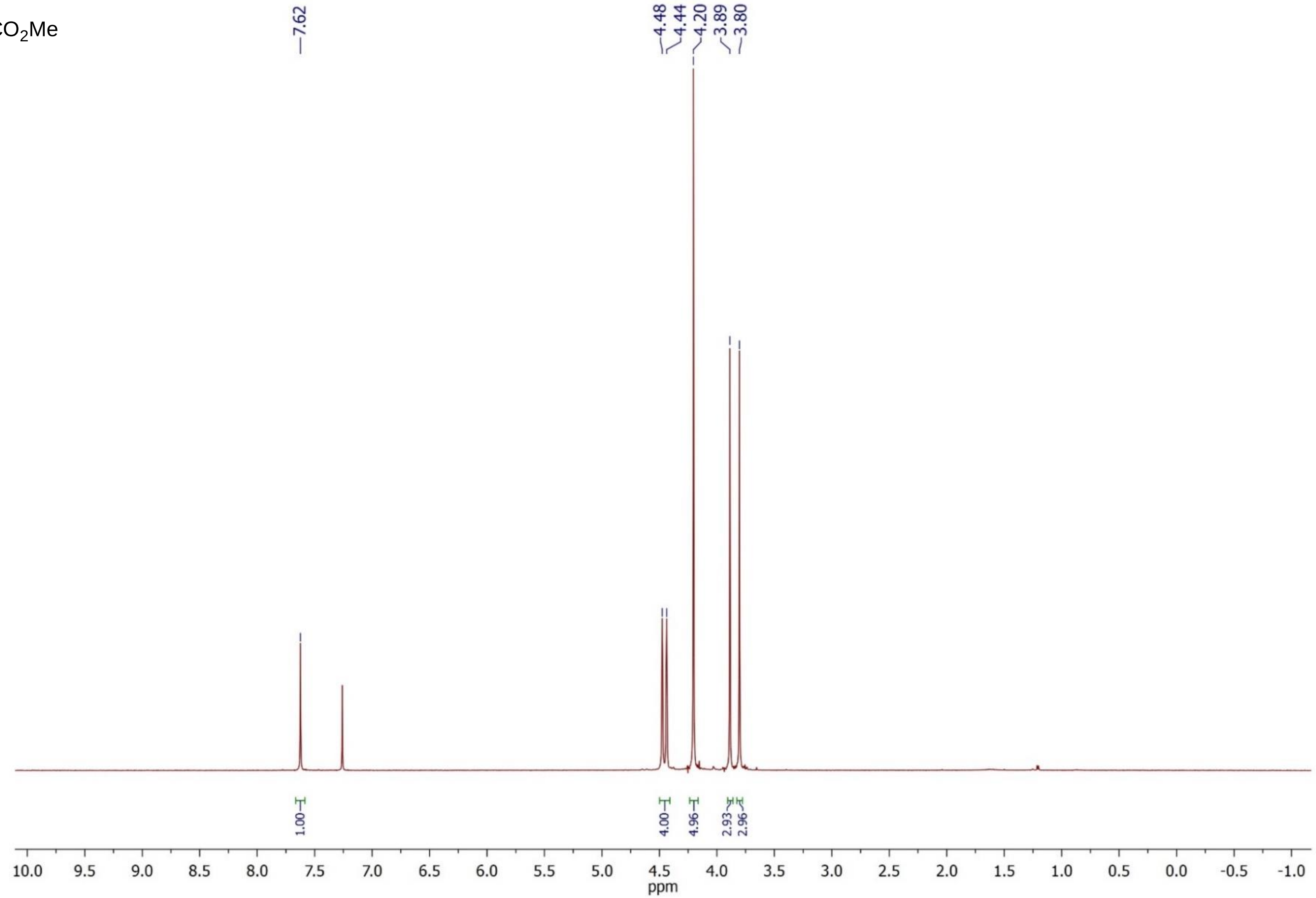

Dimethyl 2-[(ferrocenyl)methylene]malonate (S1x)

^{13}C NMR (126 MHz, $CDCl_3$)

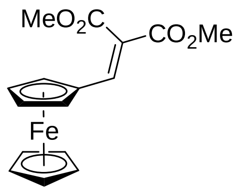

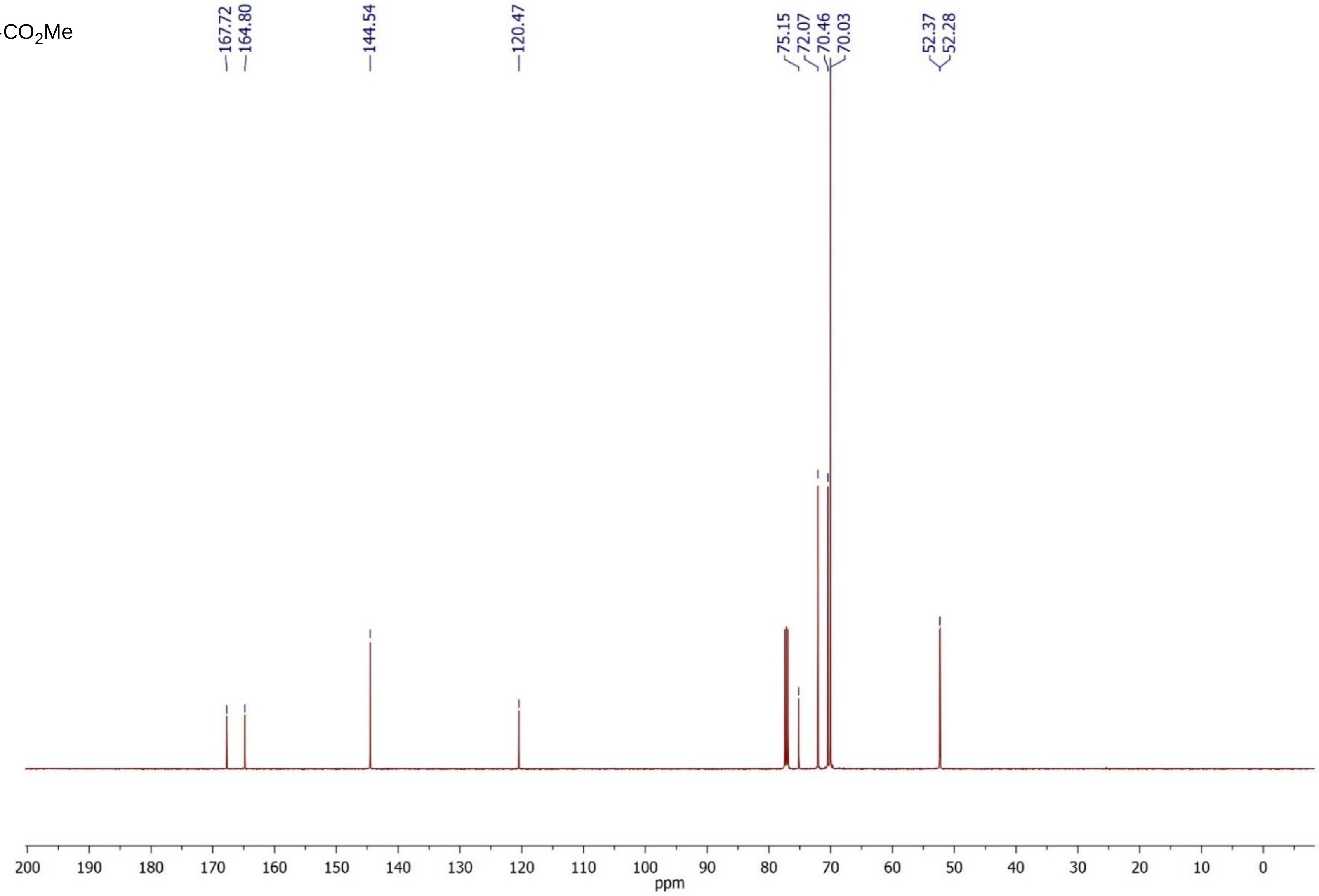

Dimethyl 2-[(*E*)-3-(4-fluorophenyl)prop-2-en-1-ylidene]malonate (S1ab)

^{1}H NMR (500 MHz, $CDCl_3$)

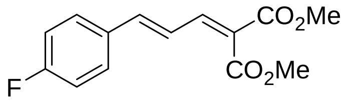

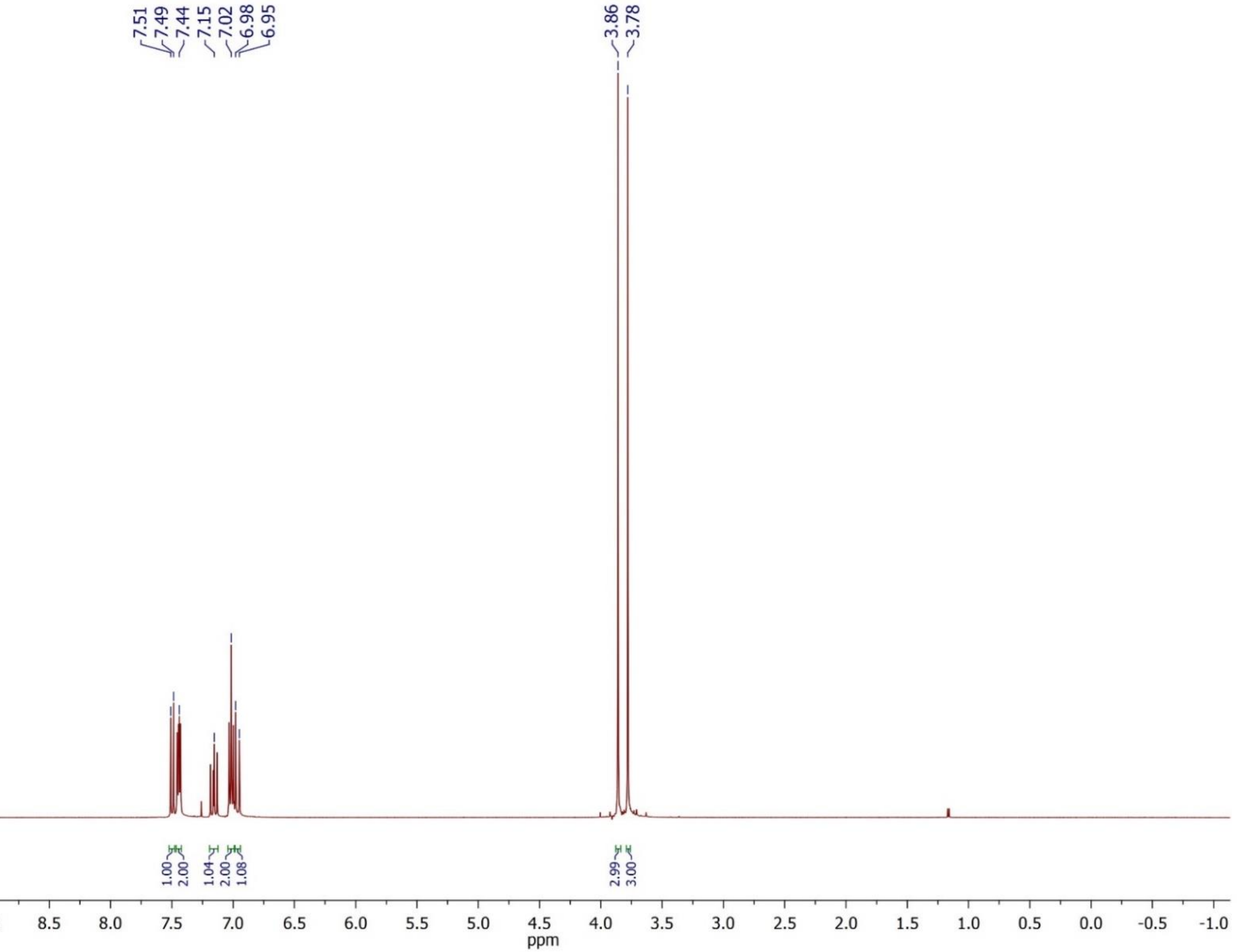

Dimethyl 2-[(*E*)-3-(4-fluorophenyl)prop-2-en-1-ylidene]malonate (S1ab)

^{13}C NMR (126 MHz, $CDCl_3$)

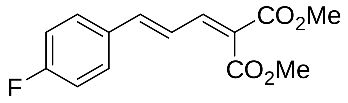

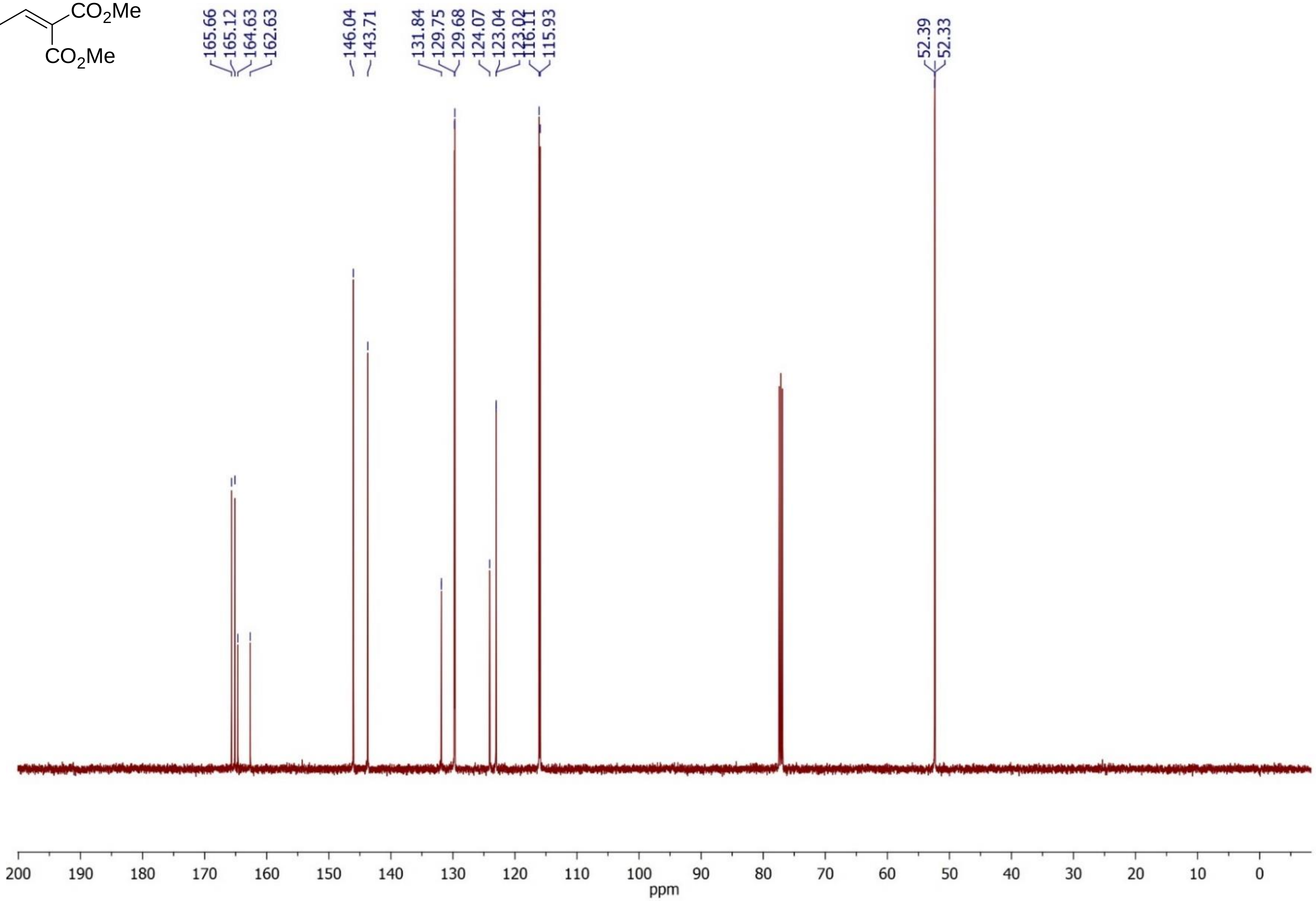

Dimethyl 2-[(*E*)-3-(4-fluorophenyl)prop-2-en-1-ylidene]malonate (S1ab)

^{19}F NMR (470 MHz, $CDCl_3$)

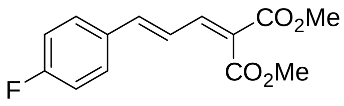

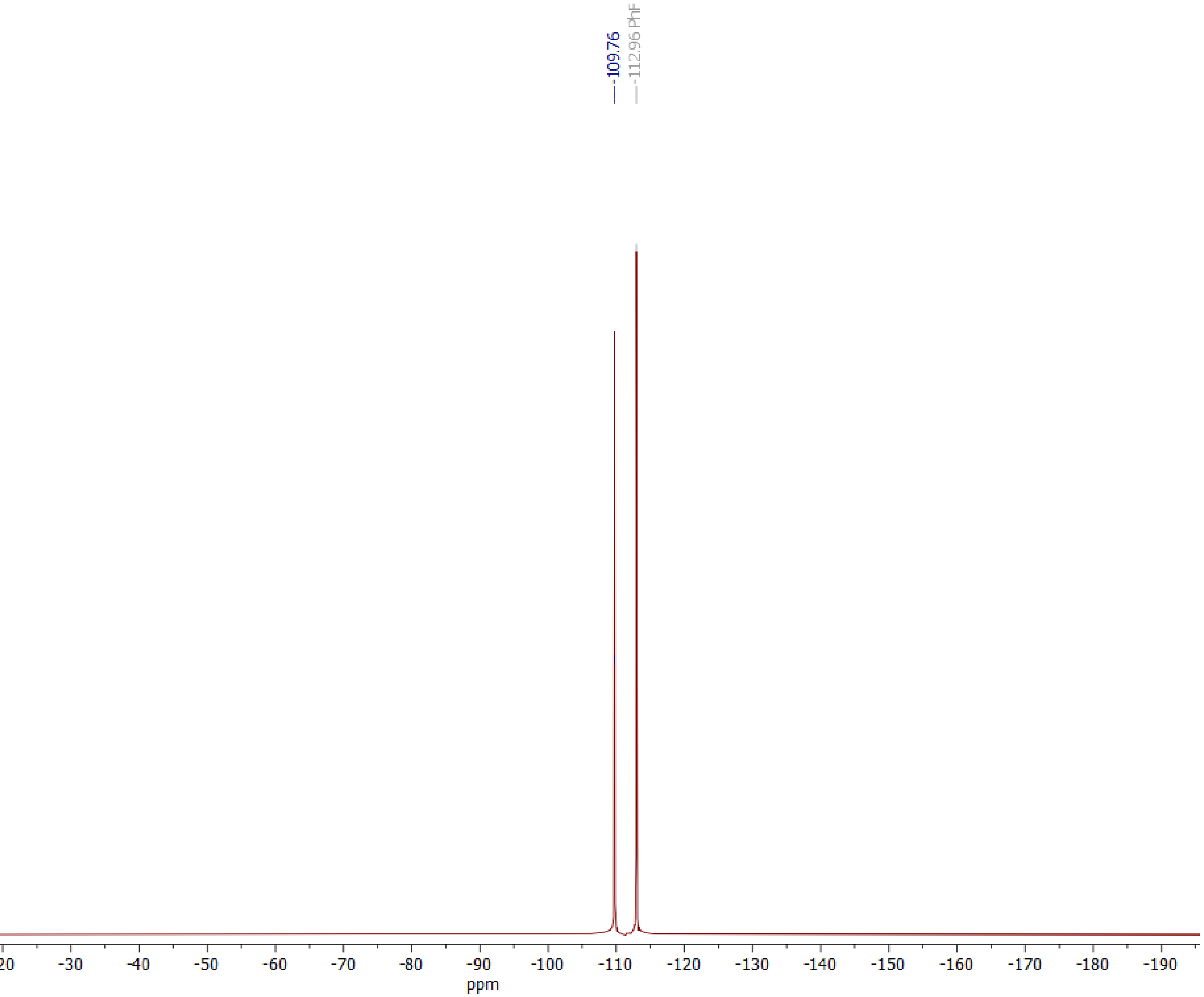

Dimethyl 2-[(*E*)-3-(4-fluorophenyl)prop-2-en-1-ylidene]malonate (S1ab)

^{1}H–^{13}C HSQC ($CDCl_3$)

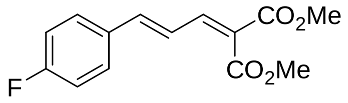

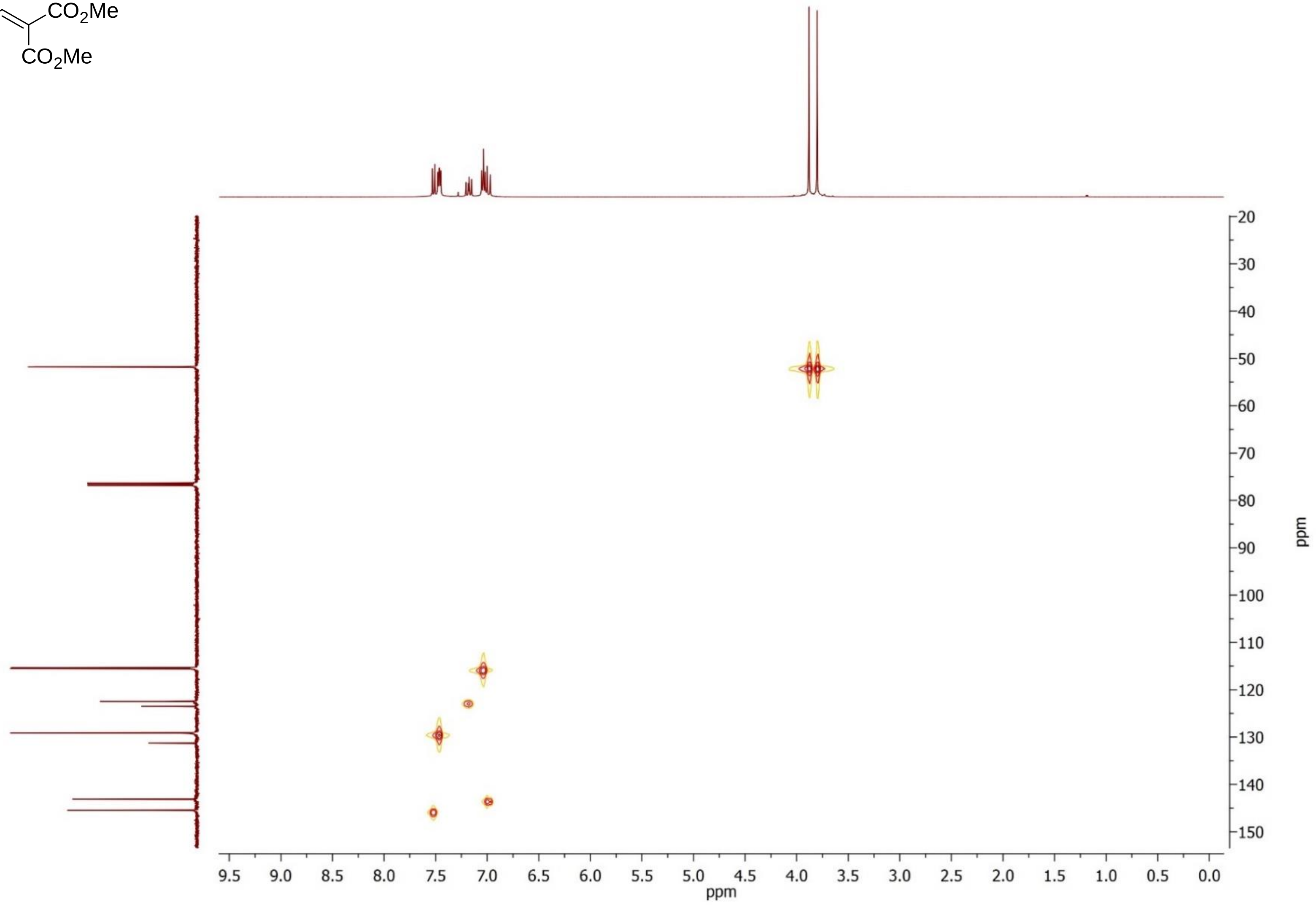

4-[(2,2-Dimethyl-4,6-dioxo-1,3-dioxan-5-ylidene)methyl]benzonitrile (S1af)

^{1}H NMR (500 MHz, $CDCl_3$)

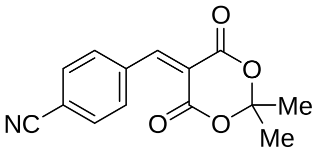

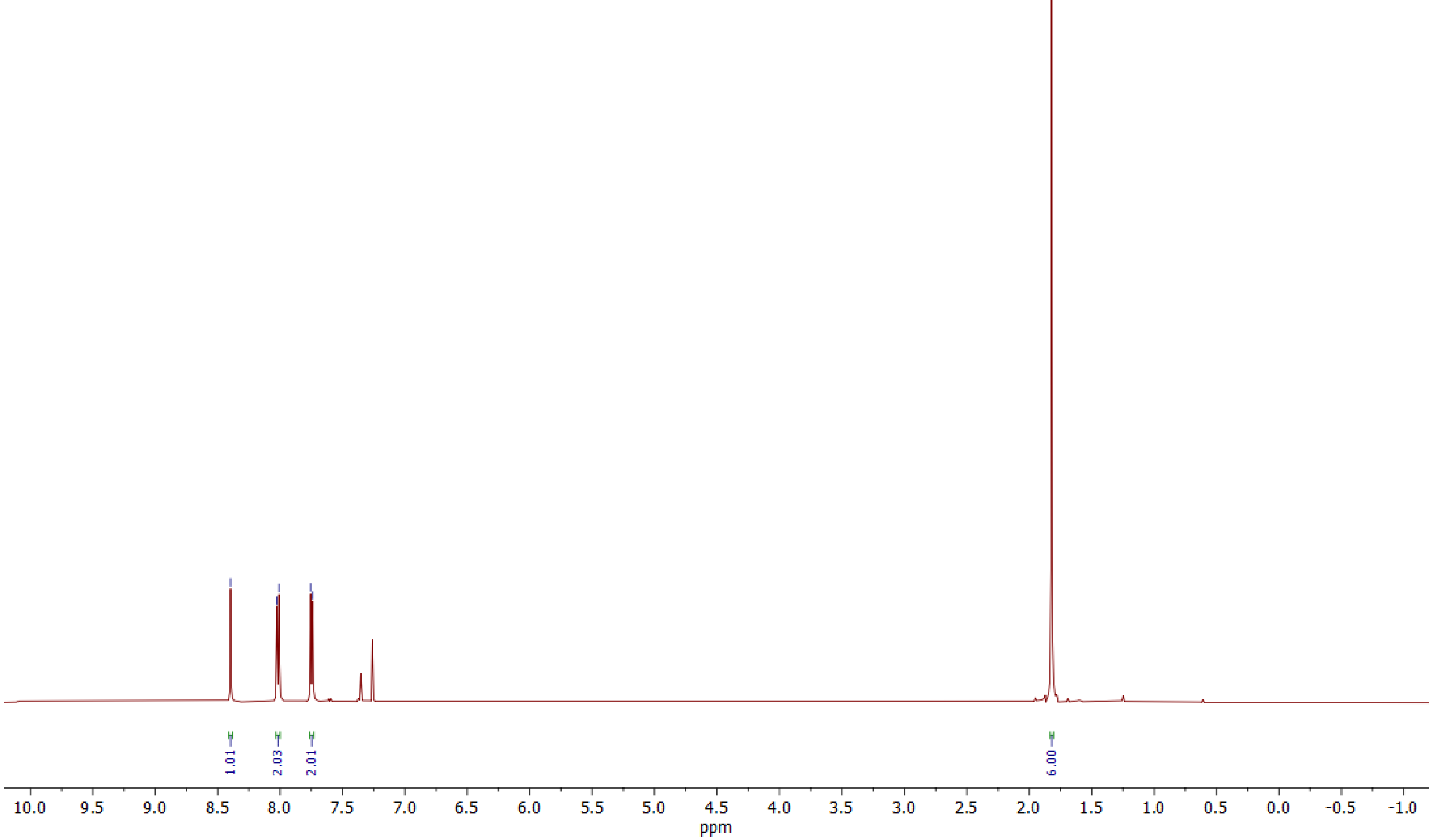

4-[(2,2-Dimethyl-4,6-dioxo-1,3-dioxan-5-ylidene)methyl]benzonitrile (S1af)

^{13}C NMR (126 MHz, $CDCl_3$)

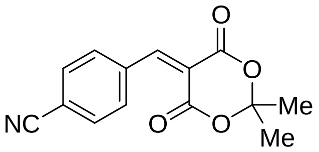

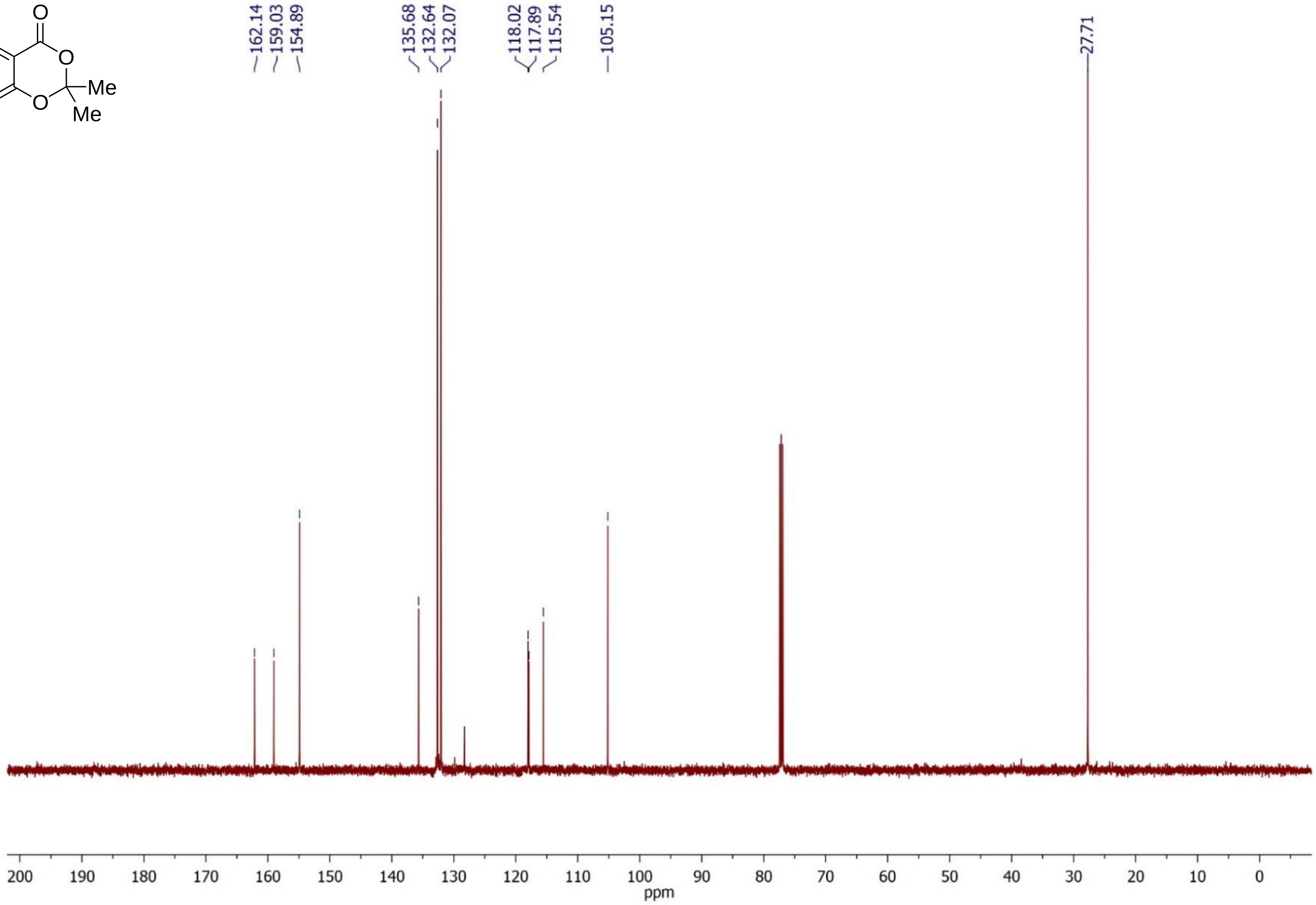

4-[(2,2-Dimethyl-4,6-dioxo-1,3-dioxan-5-ylidene)methyl]benzonitrile (S1af)

^{1}H–^{13}C HSQC ($CDCl_3$)

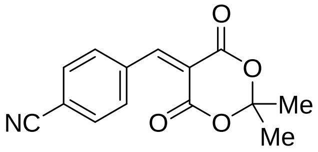

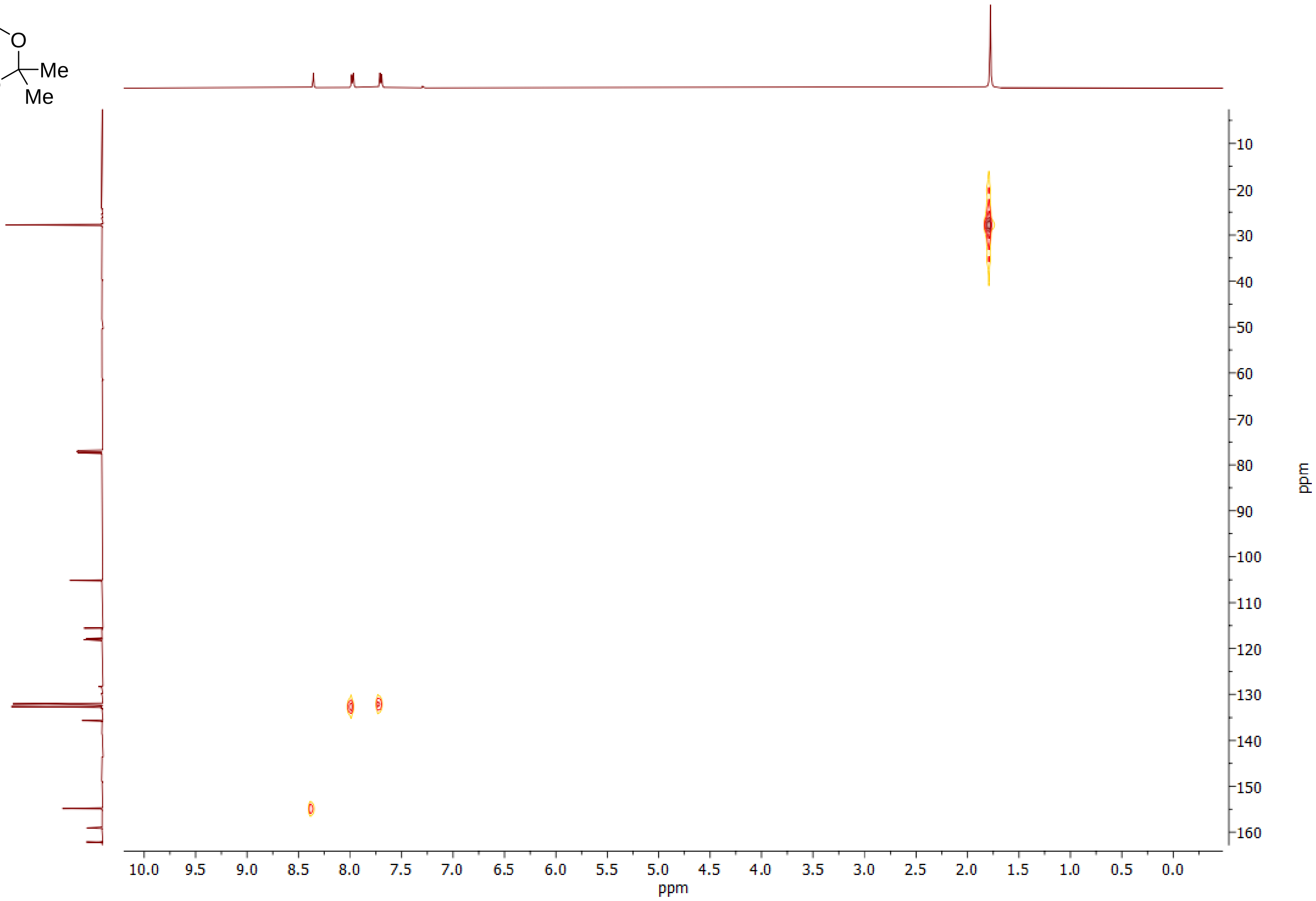

4-[(2,2-Dimethyl-4,6-dioxo-1,3-dioxan-5-ylidene)methyl]benzonitrile (S1af)

^{1}H–^{13}C HMBC ($CDCl_3$)

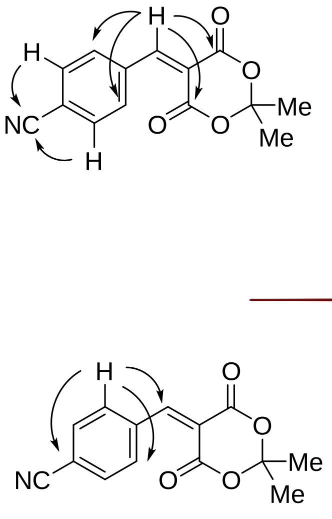

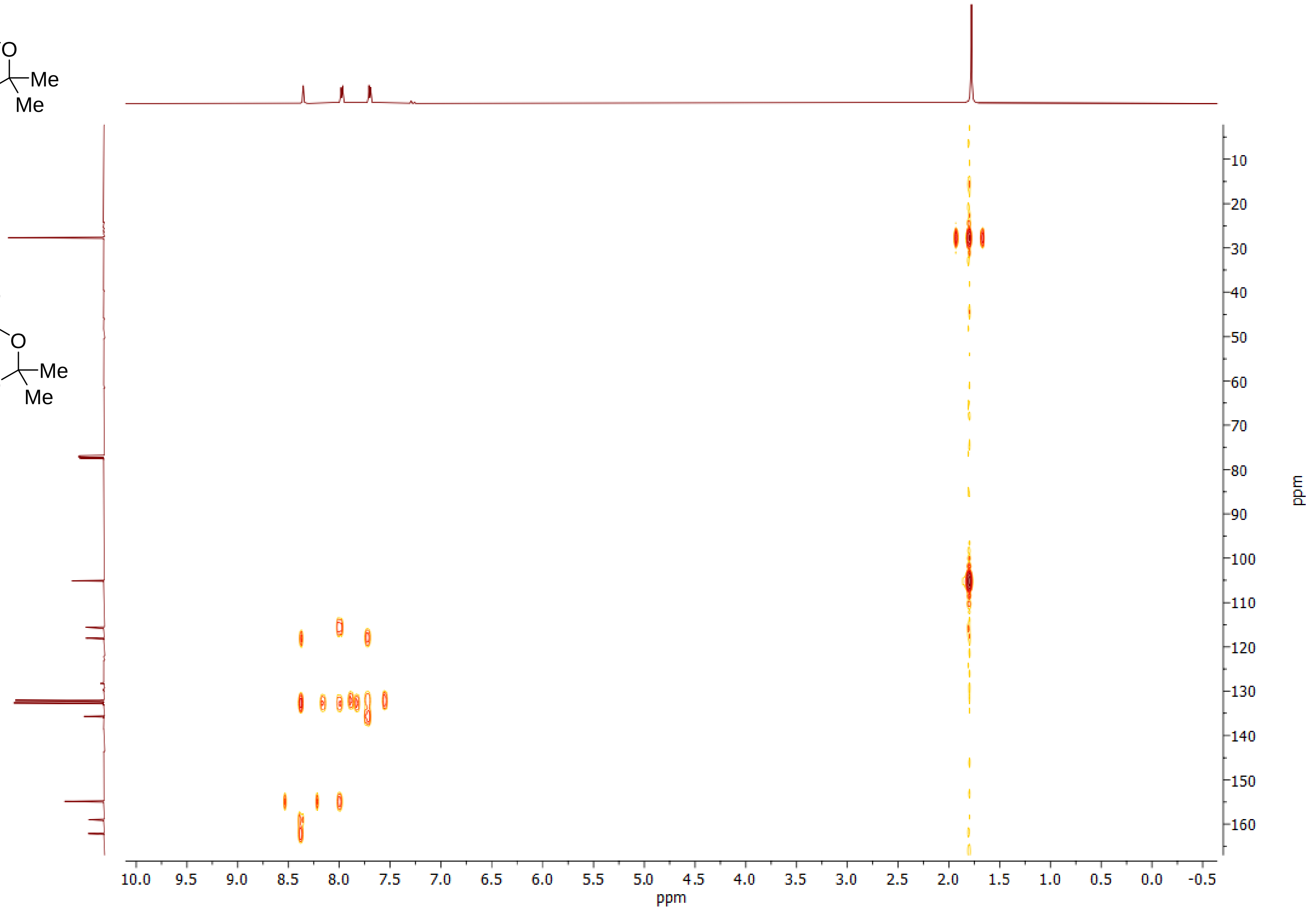

Dimethyl 2-(2,4-dimethoxyphenyl)cyclopropane-1,1-dicarboxylate (1f)

^{1}H NMR (500 MHz, $CDCl_3$)

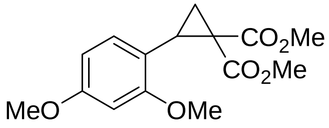

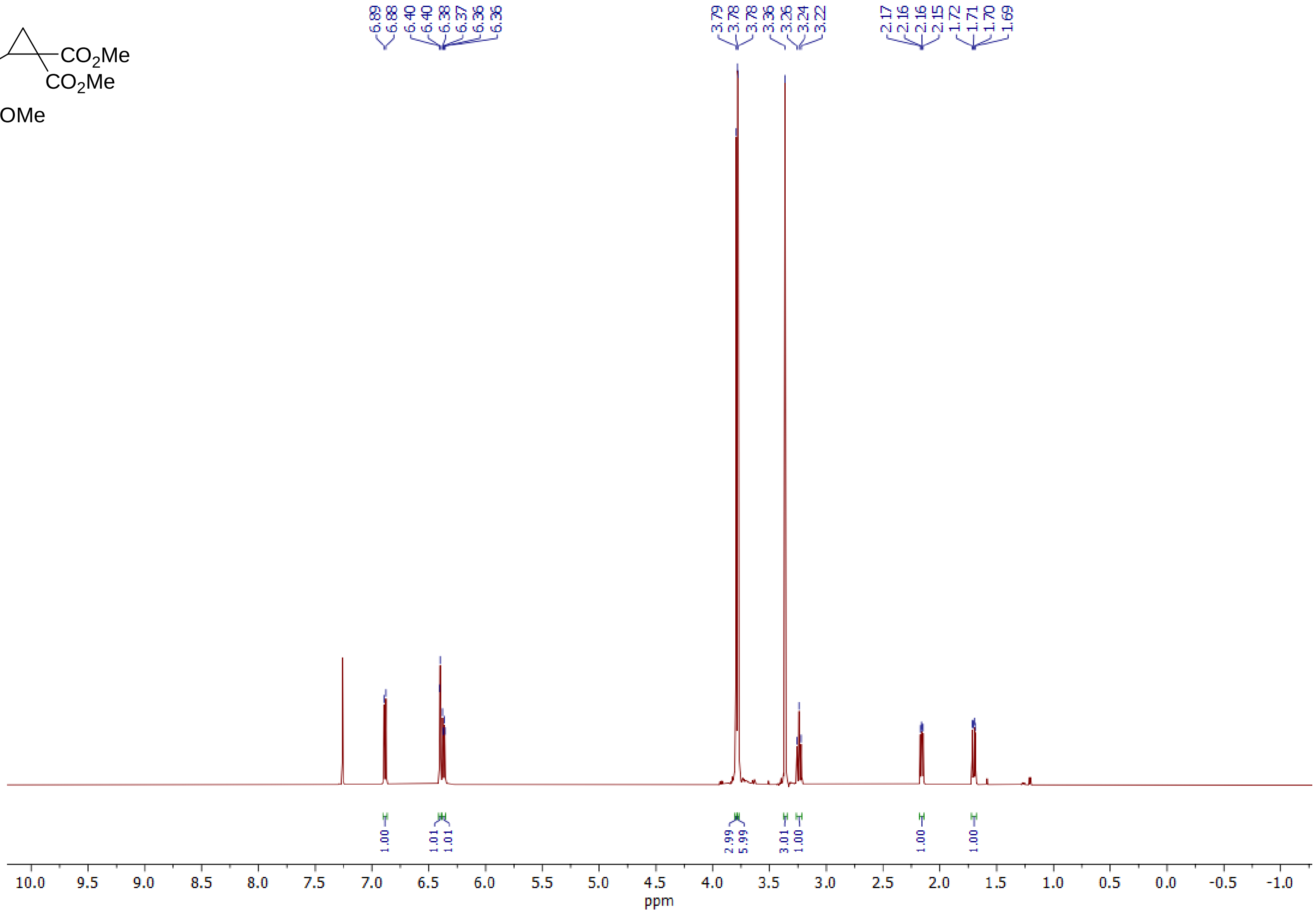

Dimethyl 2-(2,4-dimethoxyphenyl)cyclopropane-1,1-dicarboxylate (1f)

^{13}C NMR (126 MHz, $CDCl_3$)

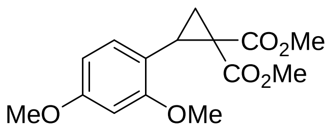

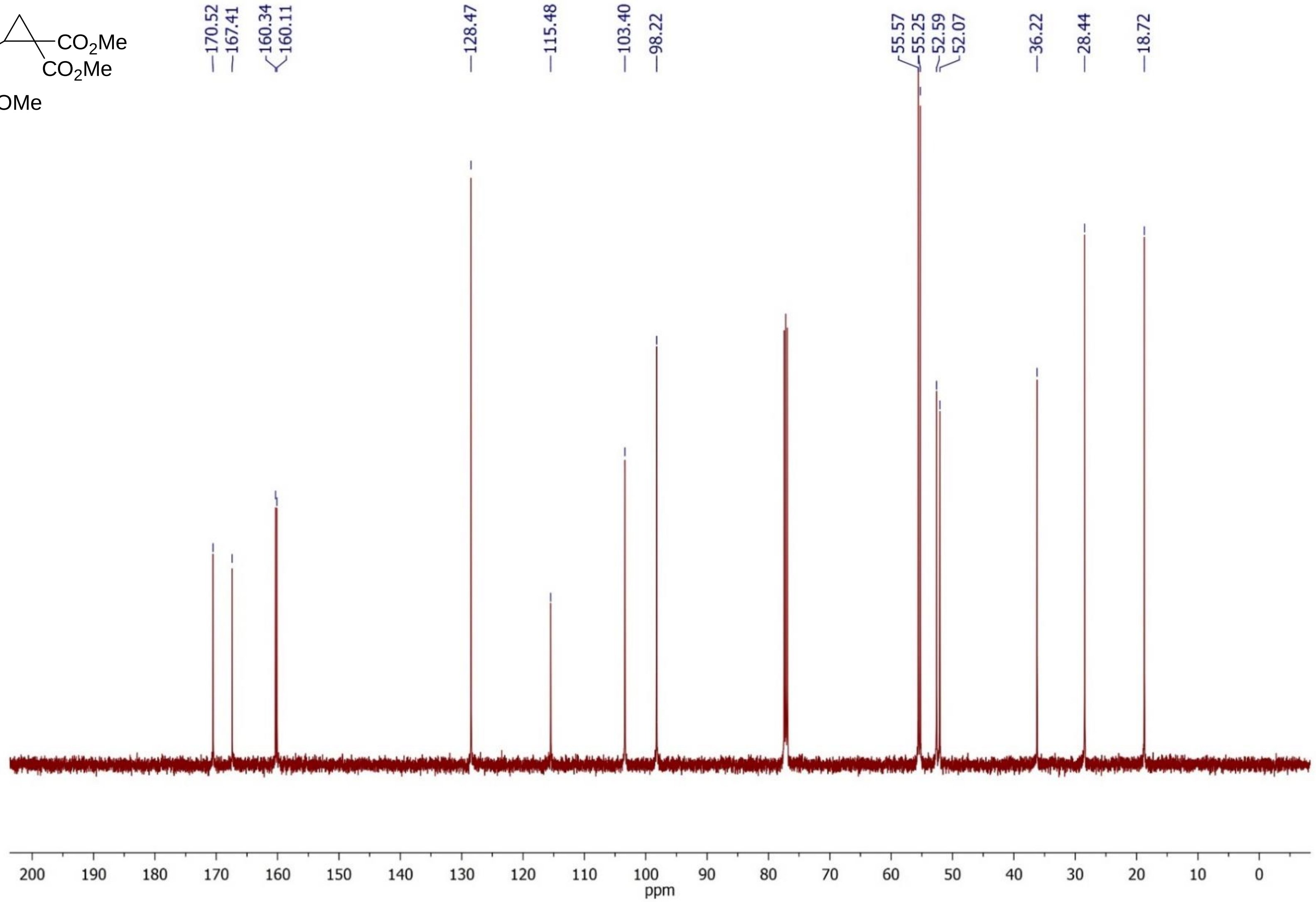

Dimethyl 2-(2,6-dimethoxyphenyl)cyclopropane-1,1-dicarboxylate (1k)

^{1}H NMR (500 MHz, $CDCl_3$)

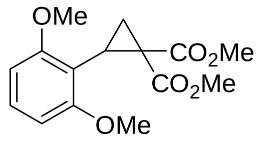

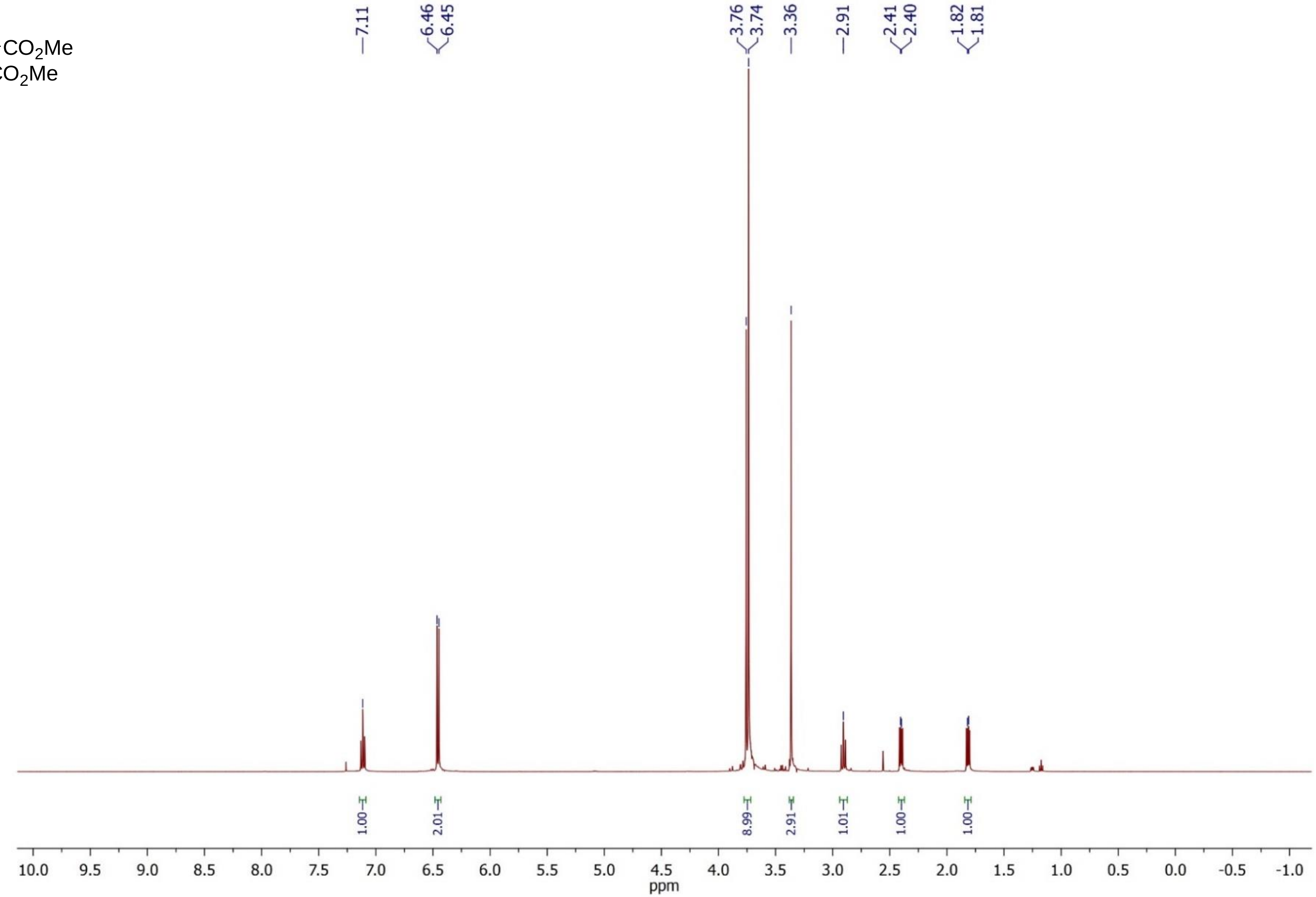

Dimethyl 2-(2,6-dimethoxyphenyl)cyclopropane-1,1-dicarboxylate (1k)

^{13}C NMR (126 MHz, $CDCl_3$)

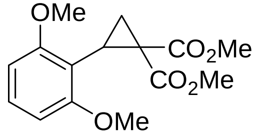

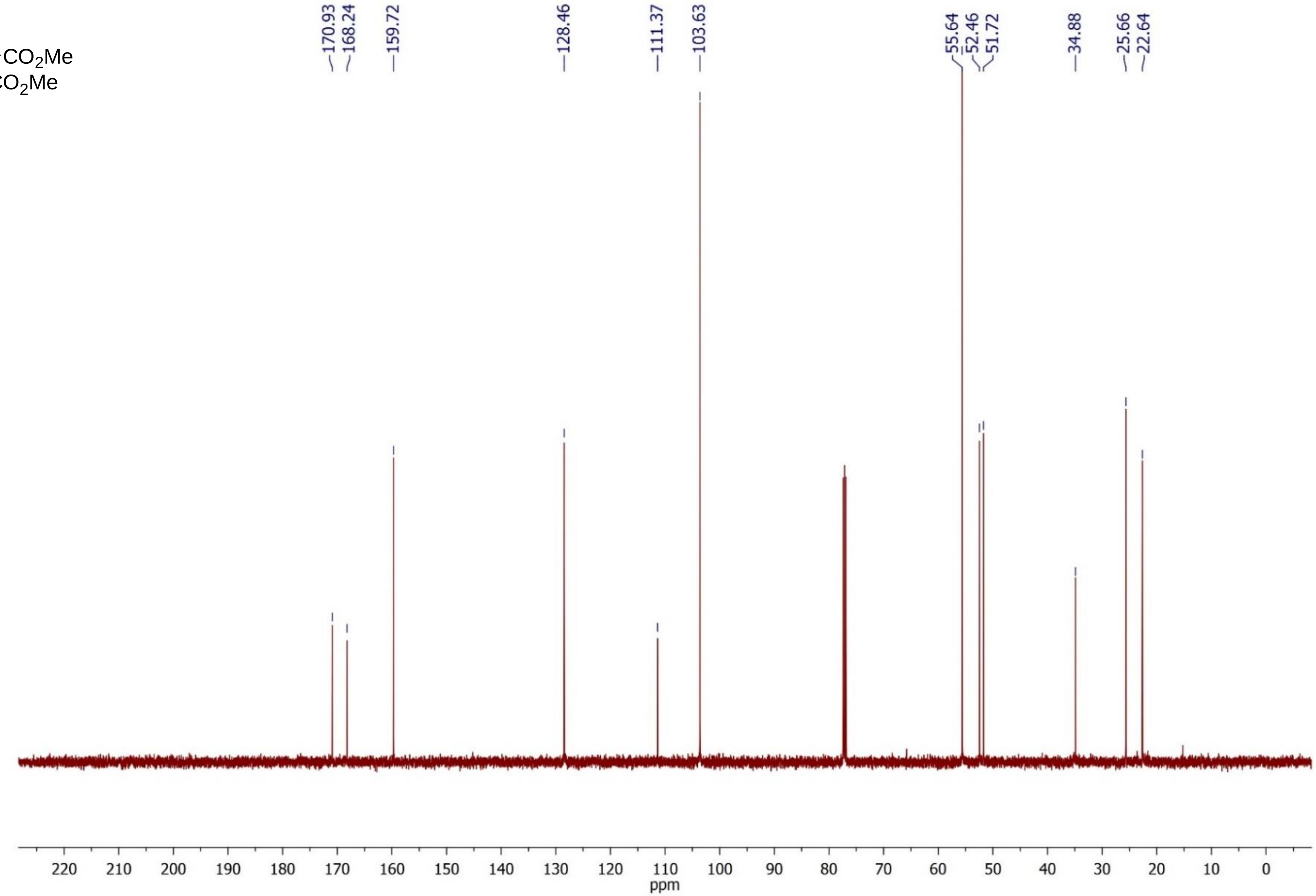

Dimethyl 2-(4-dimethylamino-2-nitrophenyl)cyclopropane-1,1-dicarboxylate (1n)

^{1}H NMR (500 MHz, $CDCl_3$)

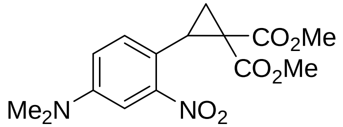

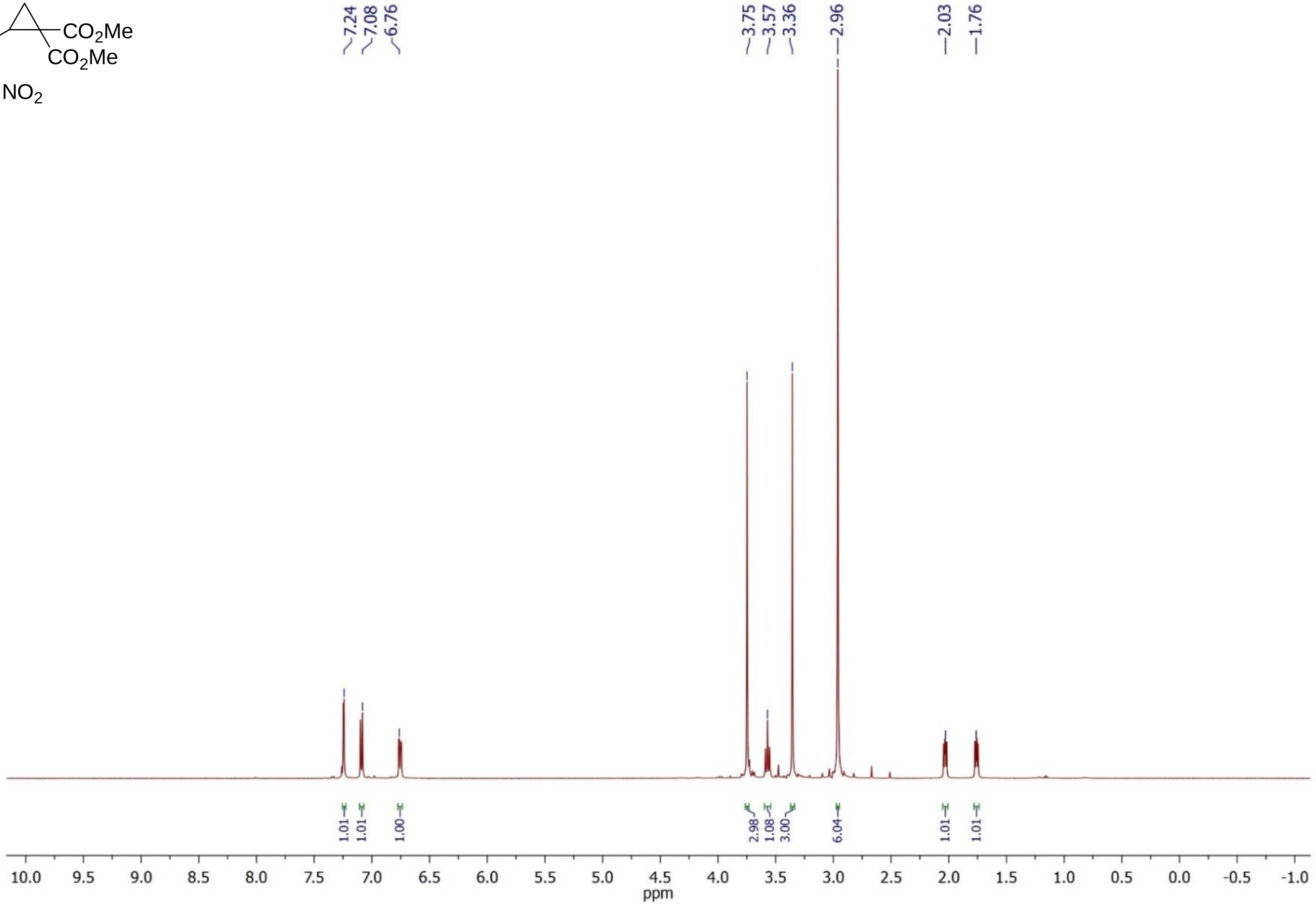

Dimethyl 2-(4-dimethylamino-2-nitrophenyl)cyclopropane-1,1-dicarboxylate (1n)

^{13}C NMR (126 MHz, $CDCl_3$)

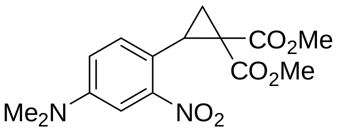

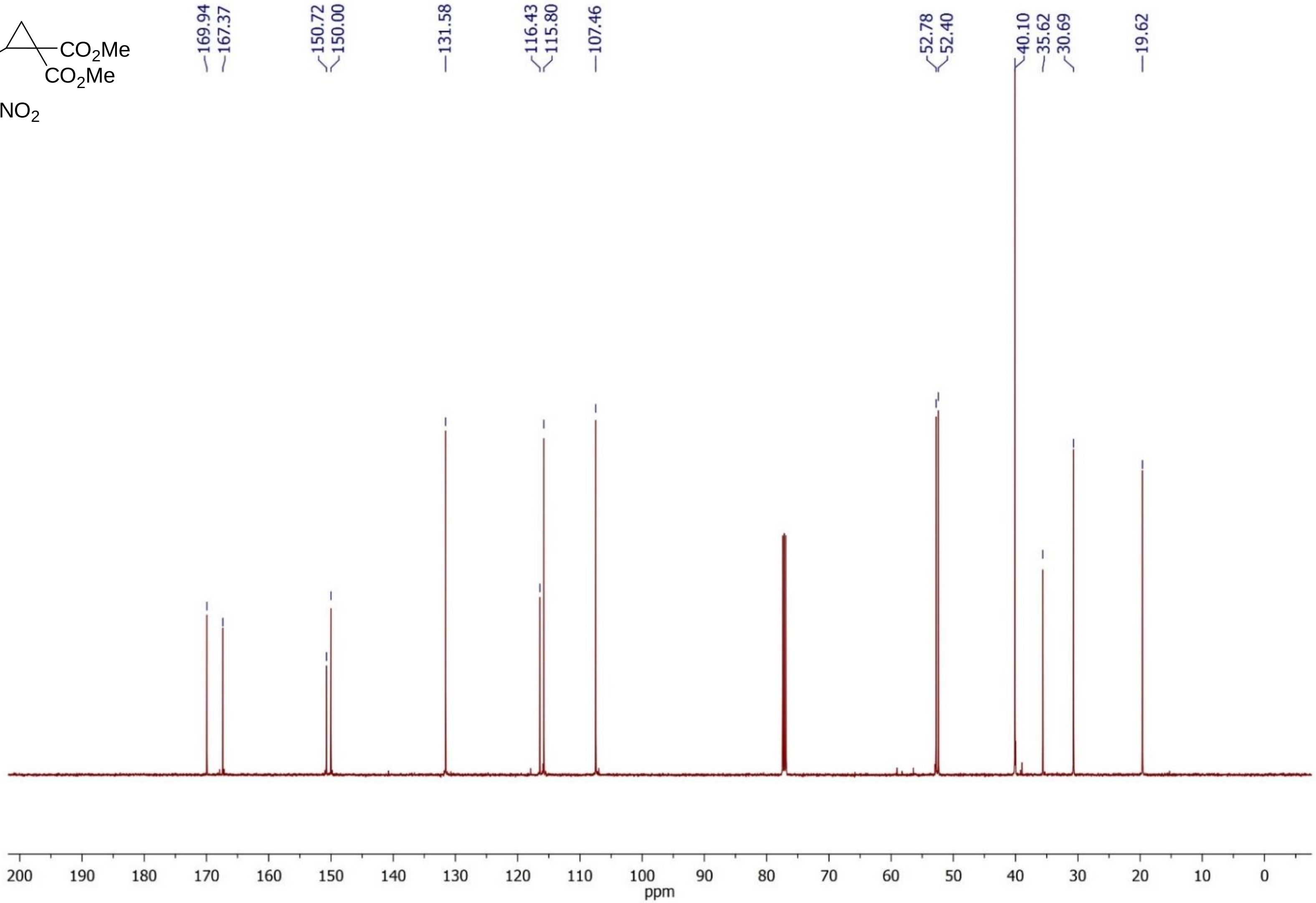

Dimethyl 2-[4′-methoxy-(1,1′-biphenyl)-4-yl]cyclopropane-1,1-dicarboxylate (1o)

^{1}H NMR (500 MHz, $CDCl_3$)

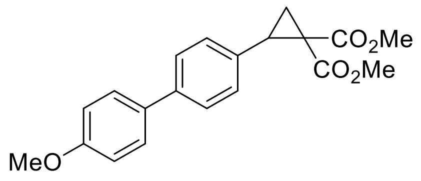

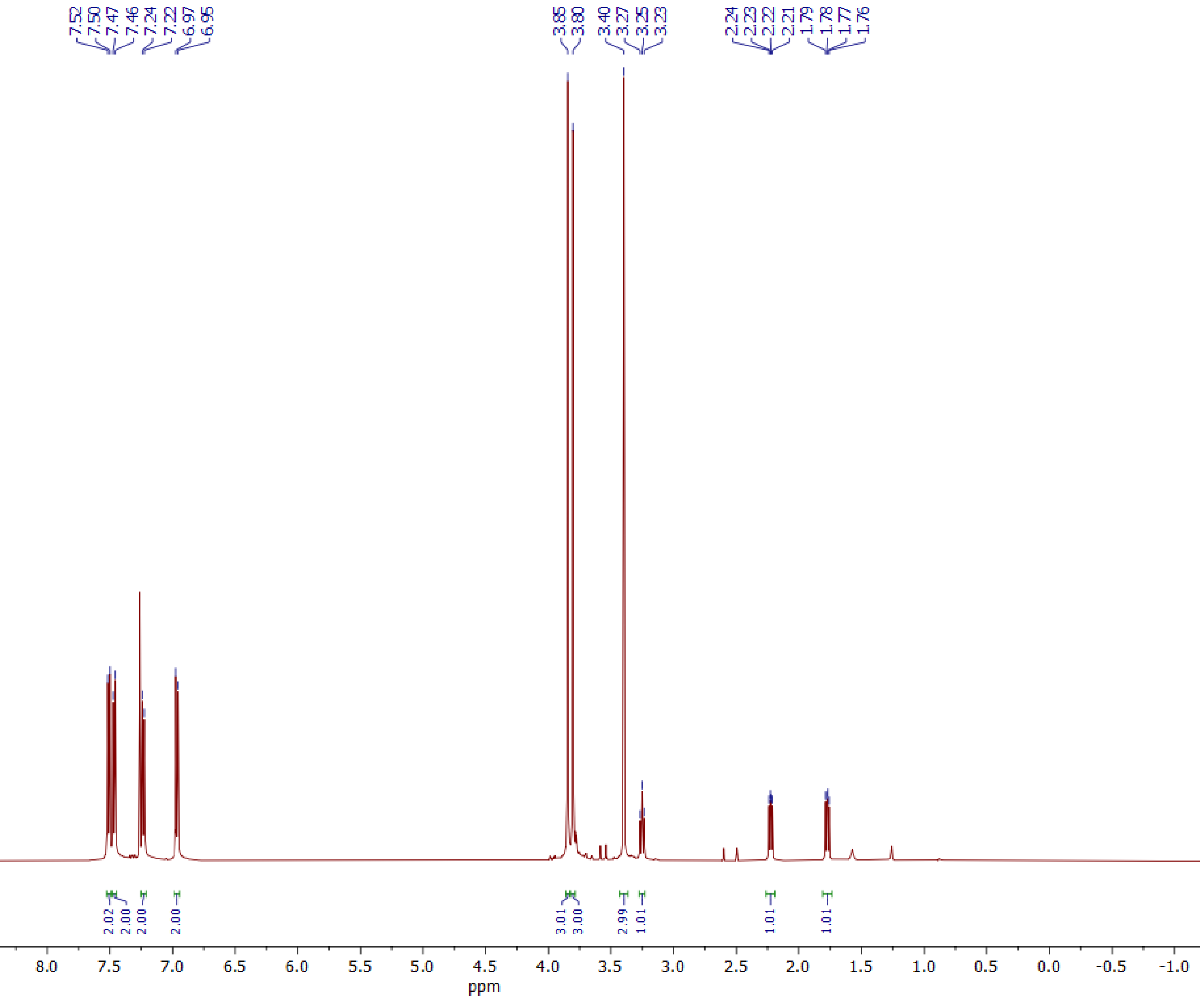

Dimethyl 2-[4′-methoxy-(1,1′-biphenyl)-4-yl]cyclopropane-1,1-dicarboxylate (1o)

^{13}C NMR (126 MHz, $CDCl_3$)

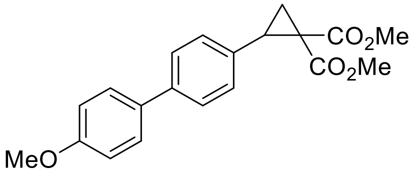

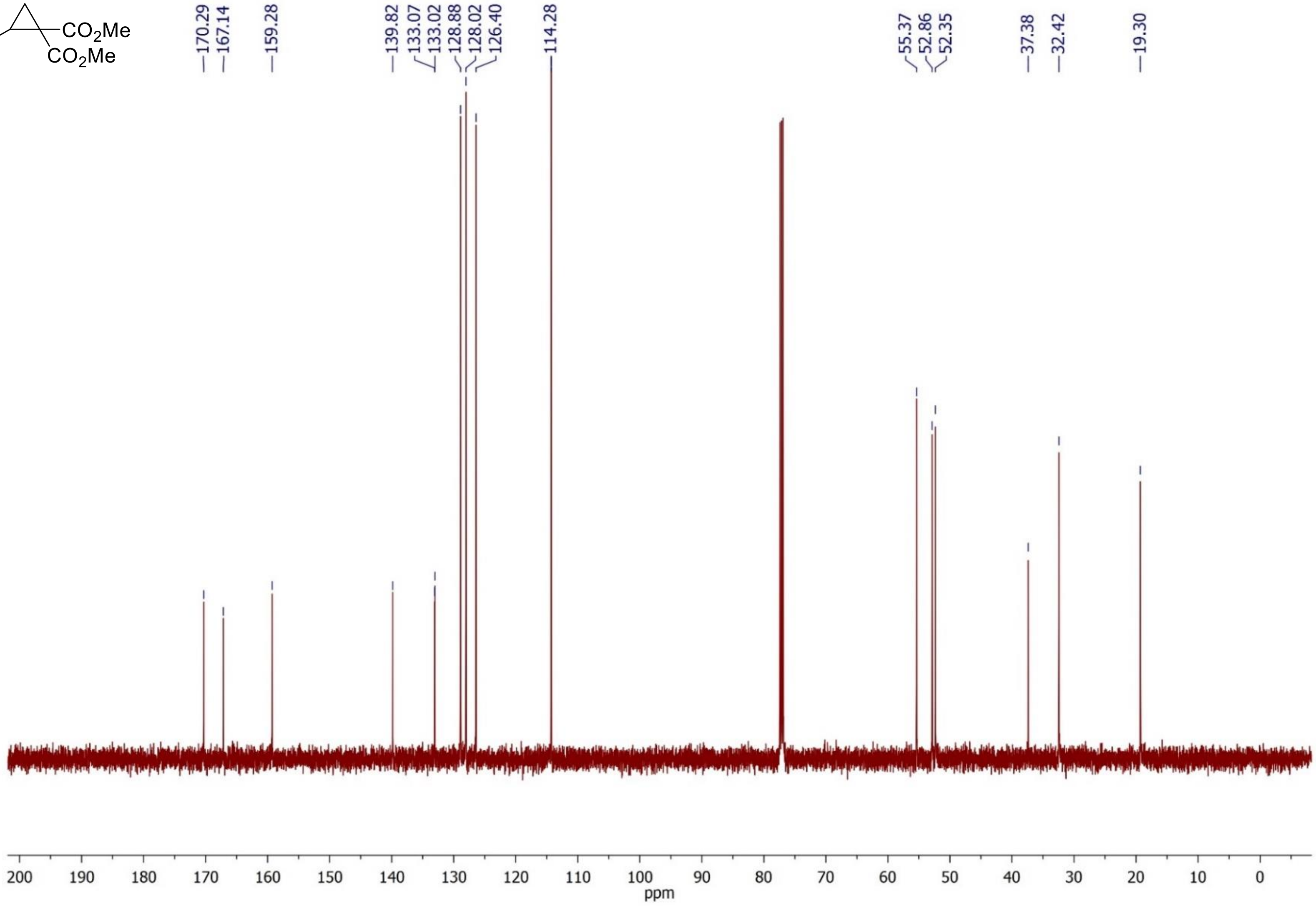

Dimethyl 2-(6-methoxynaphthalen-2-yl)cyclopropane-1,1-dicarboxylate (1p)

^{1}H NMR (500 MHz, $CDCl_3$)

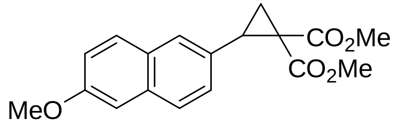

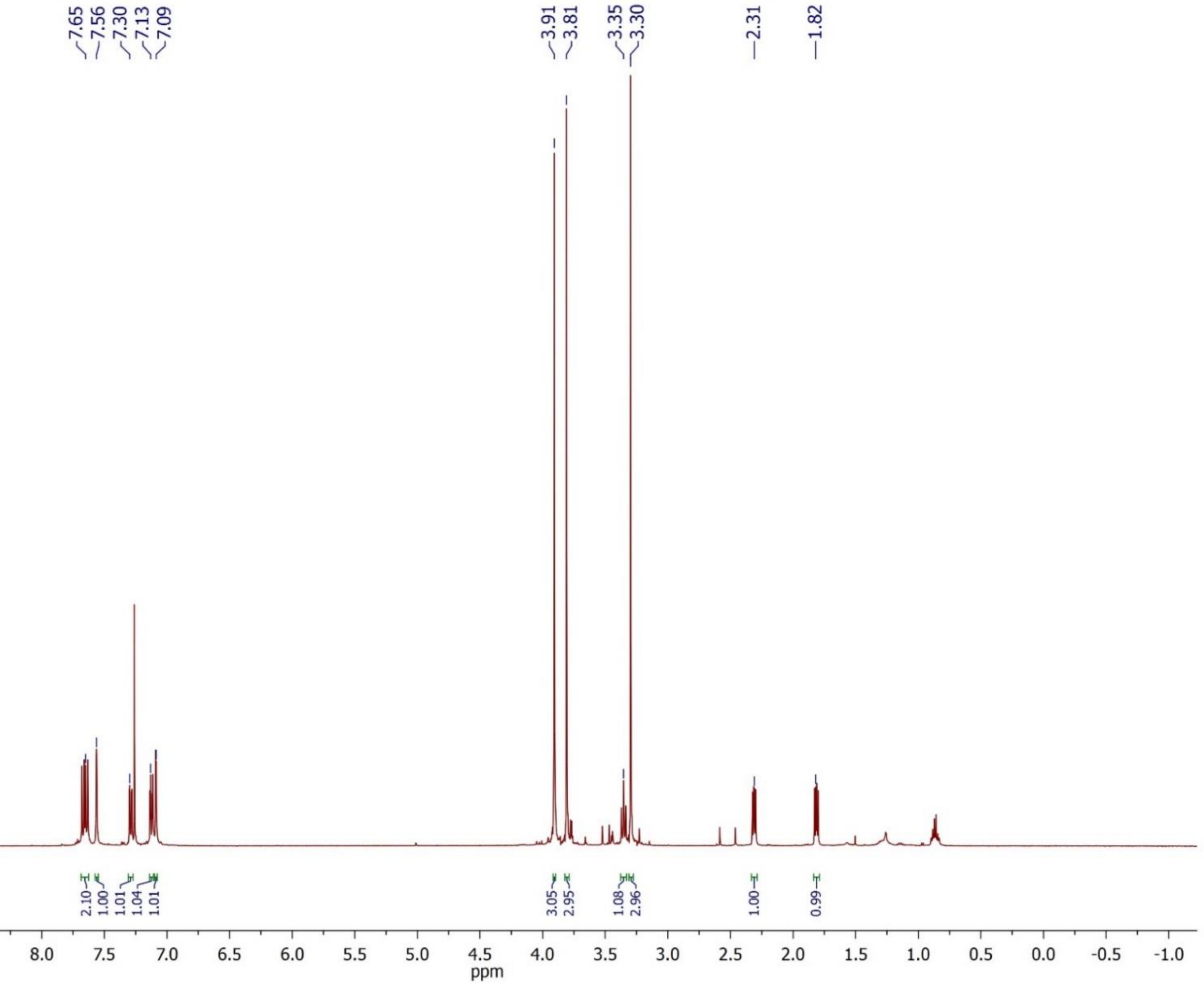

Dimethyl 2-(6-methoxynaphthalen-2-yl)cyclopropane-1,1-dicarboxylate (1p)

^{13}C NMR (126 MHz, $CDCl_3$)

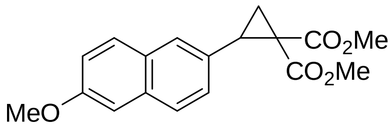

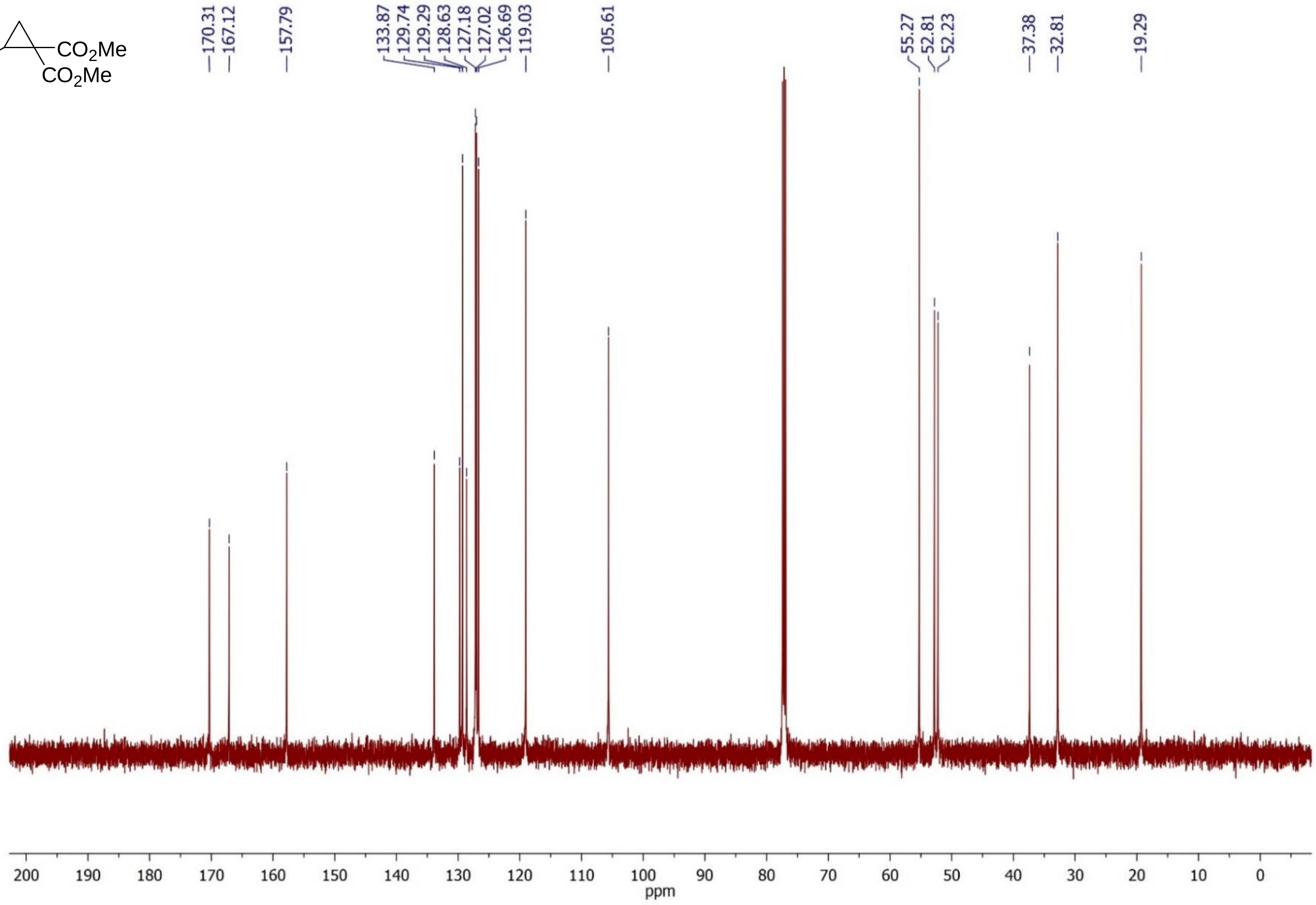

Dimethyl 2-(ferrocenyl)cyclopropane-1,1-dicarboxylate (1x)

^{1}H NMR (500 MHz, $CDCl_3$)

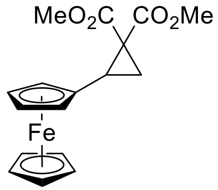

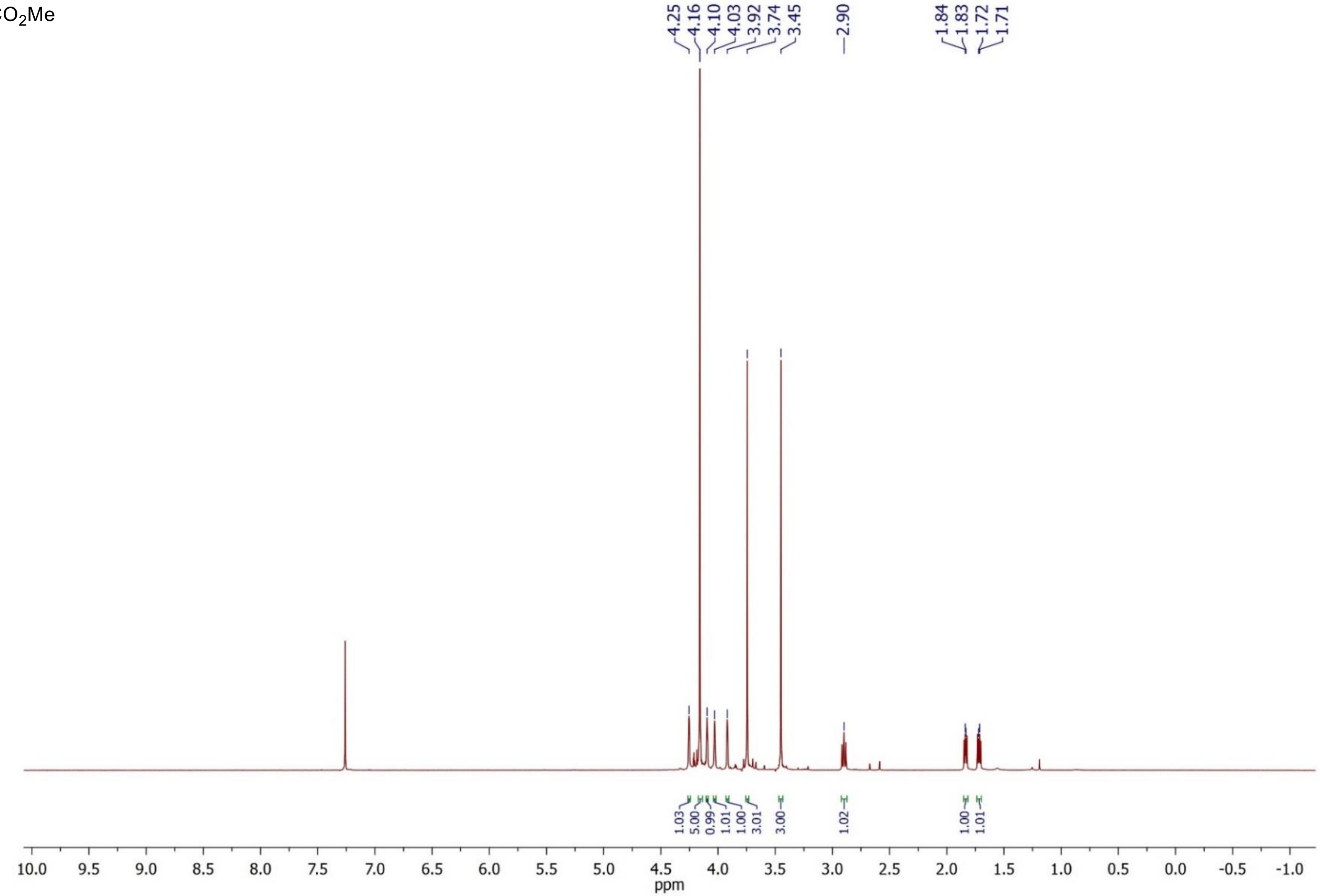

Dimethyl 2-(ferrocenyl)cyclopropane-1,1-dicarboxylate (1x)

^{13}C NMR (126 MHz, $CDCl_3$)

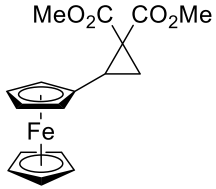

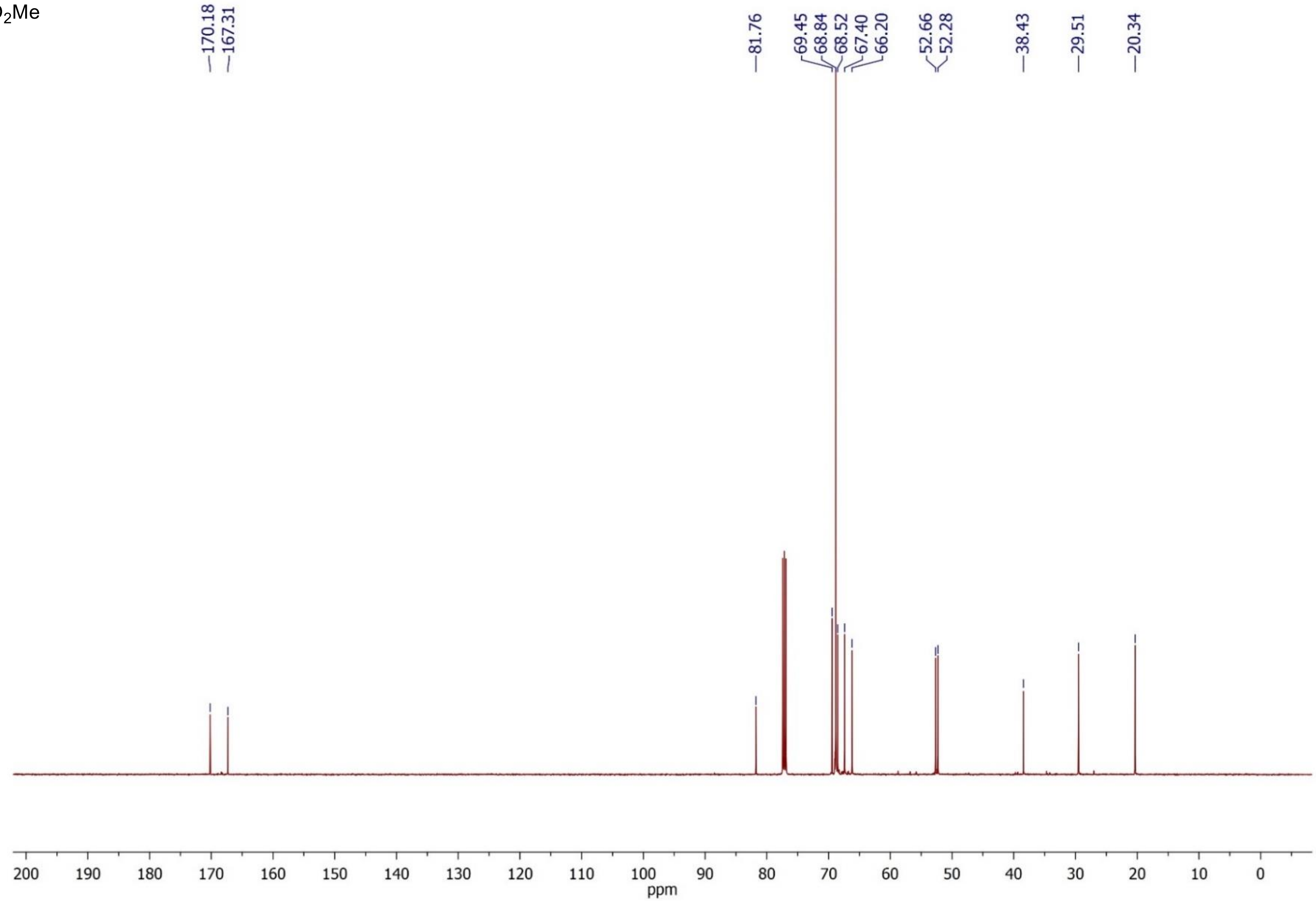

Dimethyl 2-[(*E*)-2-(4-fluorophenyl)ethenyl]cyclopropane-1,1-dicarboxylate (1ab)

^1H NMR (500 MHz, $CDCl_3$)

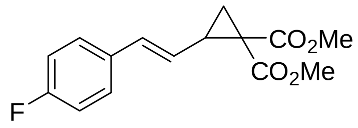

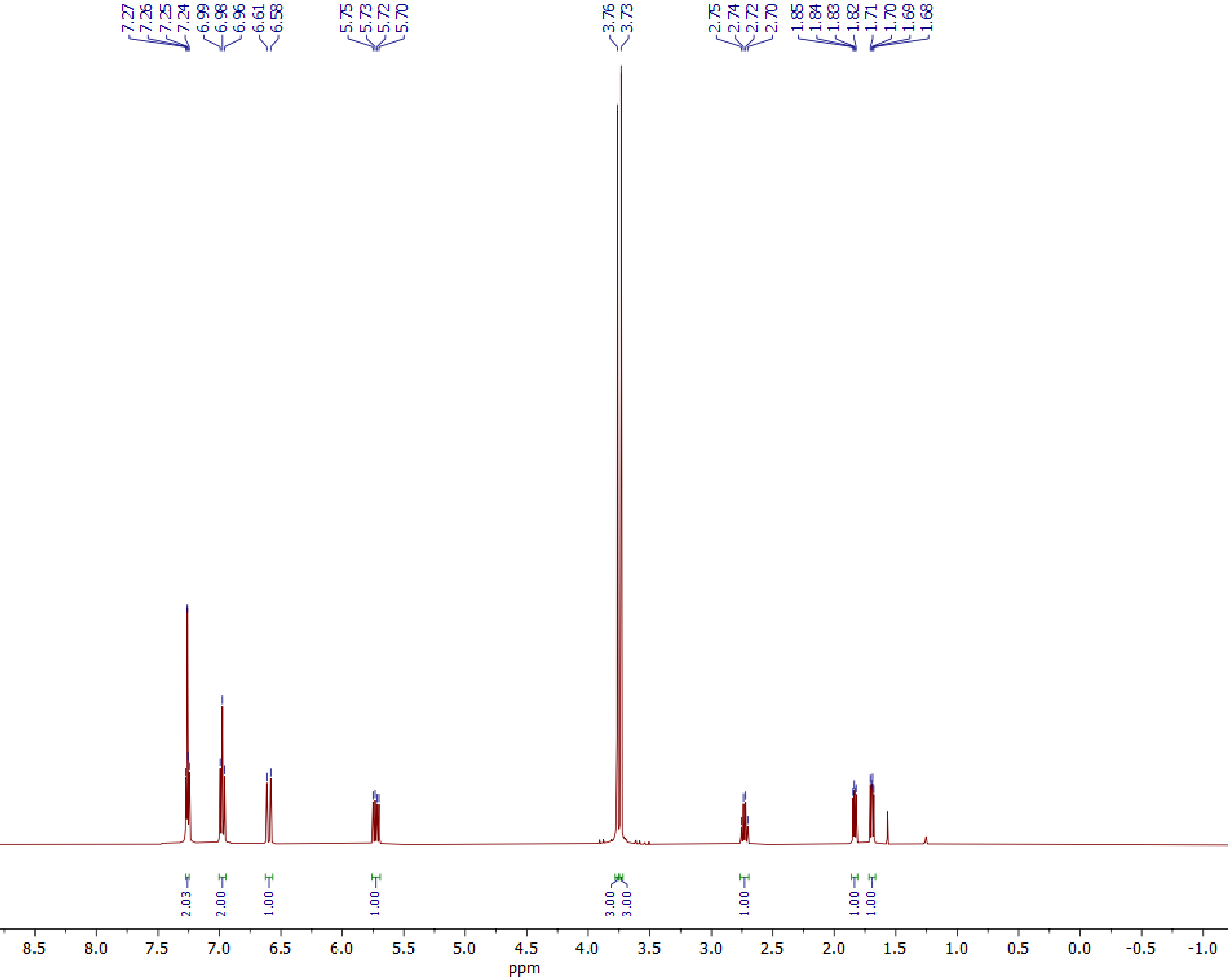

Dimethyl 2-[(*E*)-2-(4-fluorophenyl)ethenyl]cyclopropane-1,1-dicarboxylate (1ab)

^{13}C NMR (126 MHz, $CDCl_3$)

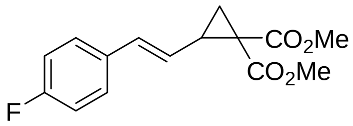

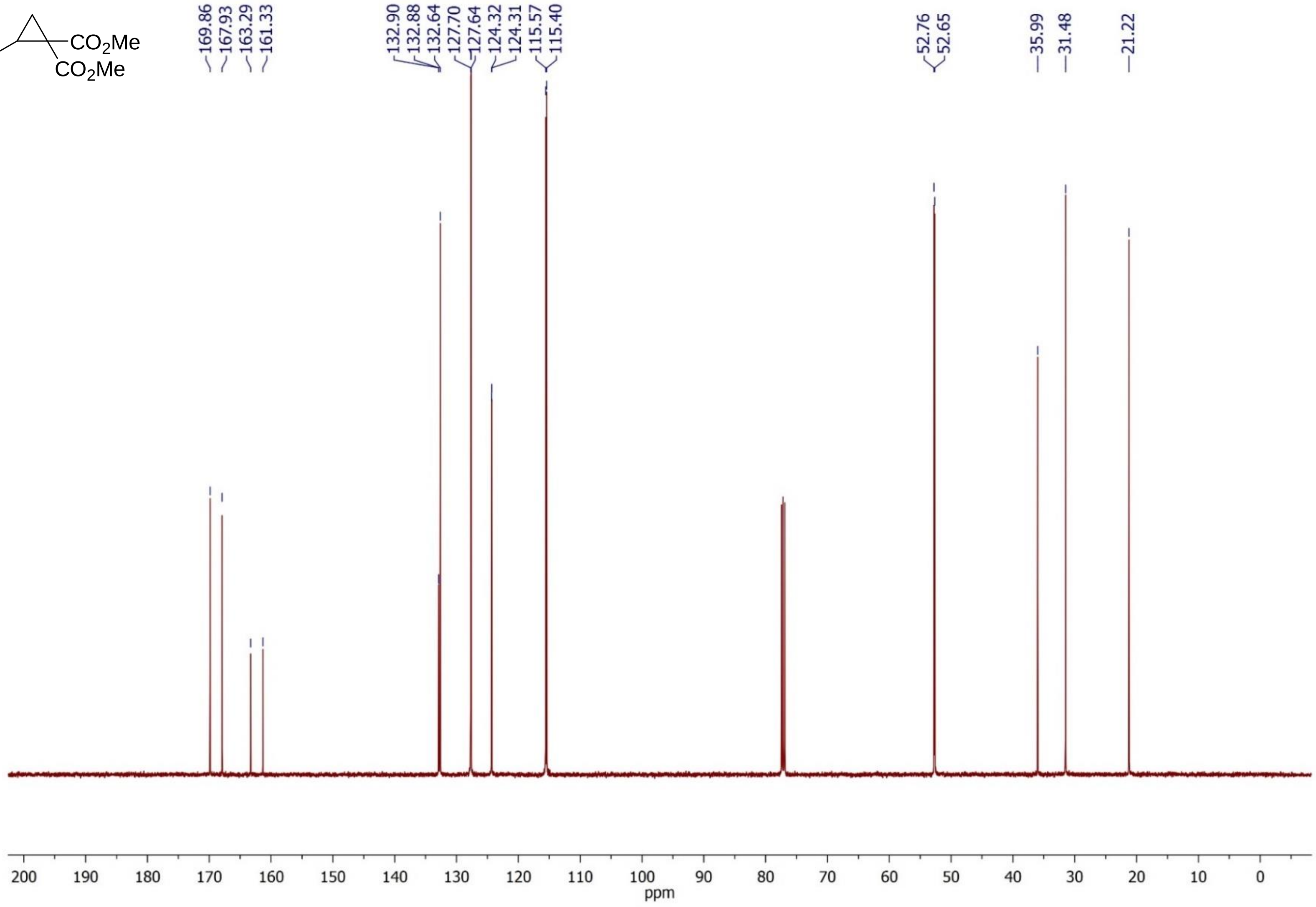

Dimethyl 2-[(*E*)-2-(4-fluorophenyl)ethenyl]cyclopropane-1,1-dicarboxylate (1ab)

^{19}F NMR (470 MHz, $CDCl_3$)

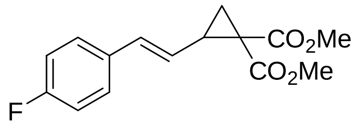

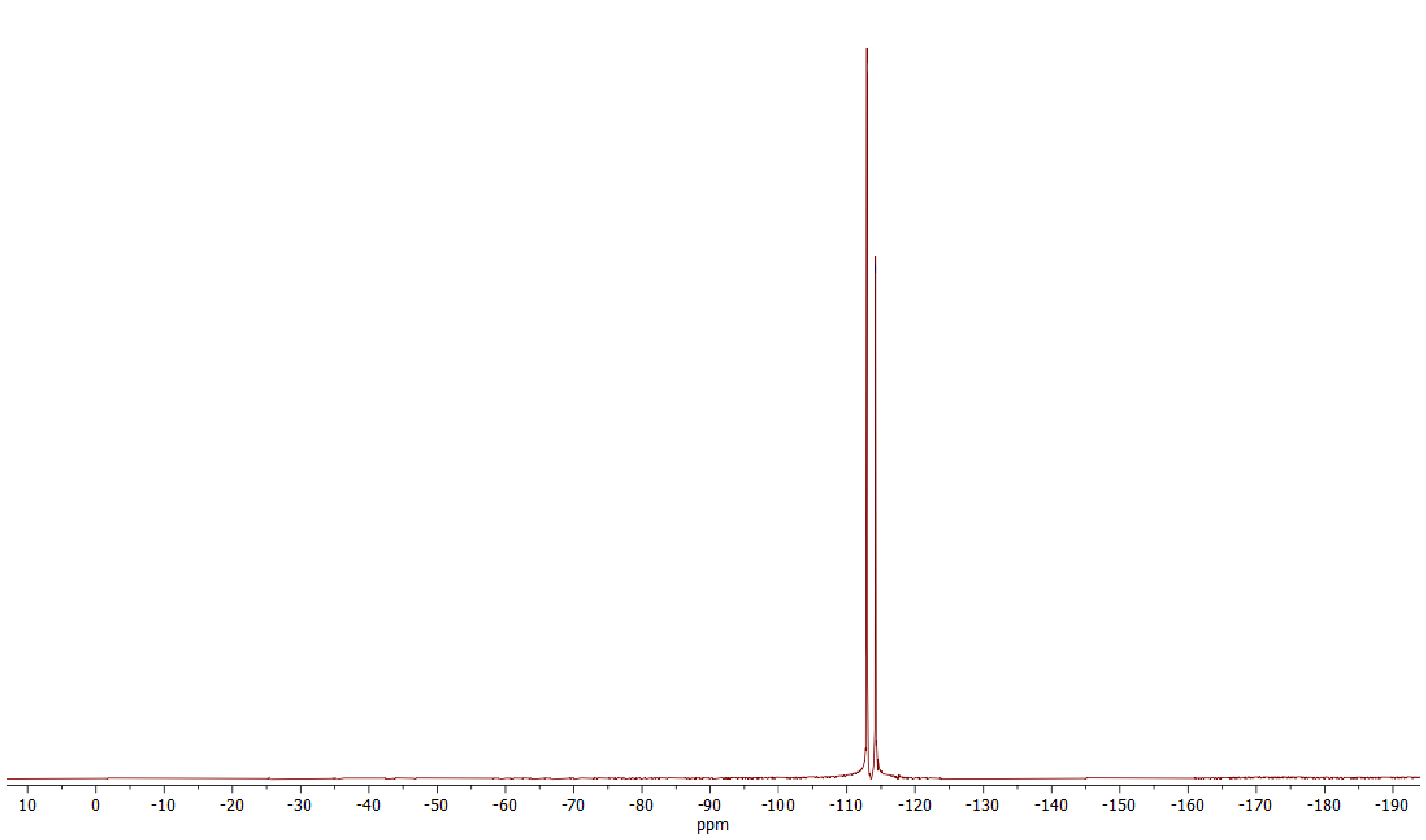

Dimethyl 2-[(*E*)-2-(4-fluorophenyl)ethenyl]cyclopropane-1,1-dicarboxylate (1ab)
^{1}H–^{13}C HSQC ($CDCl_3$)

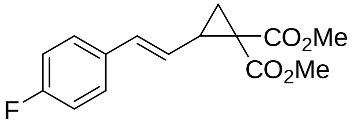

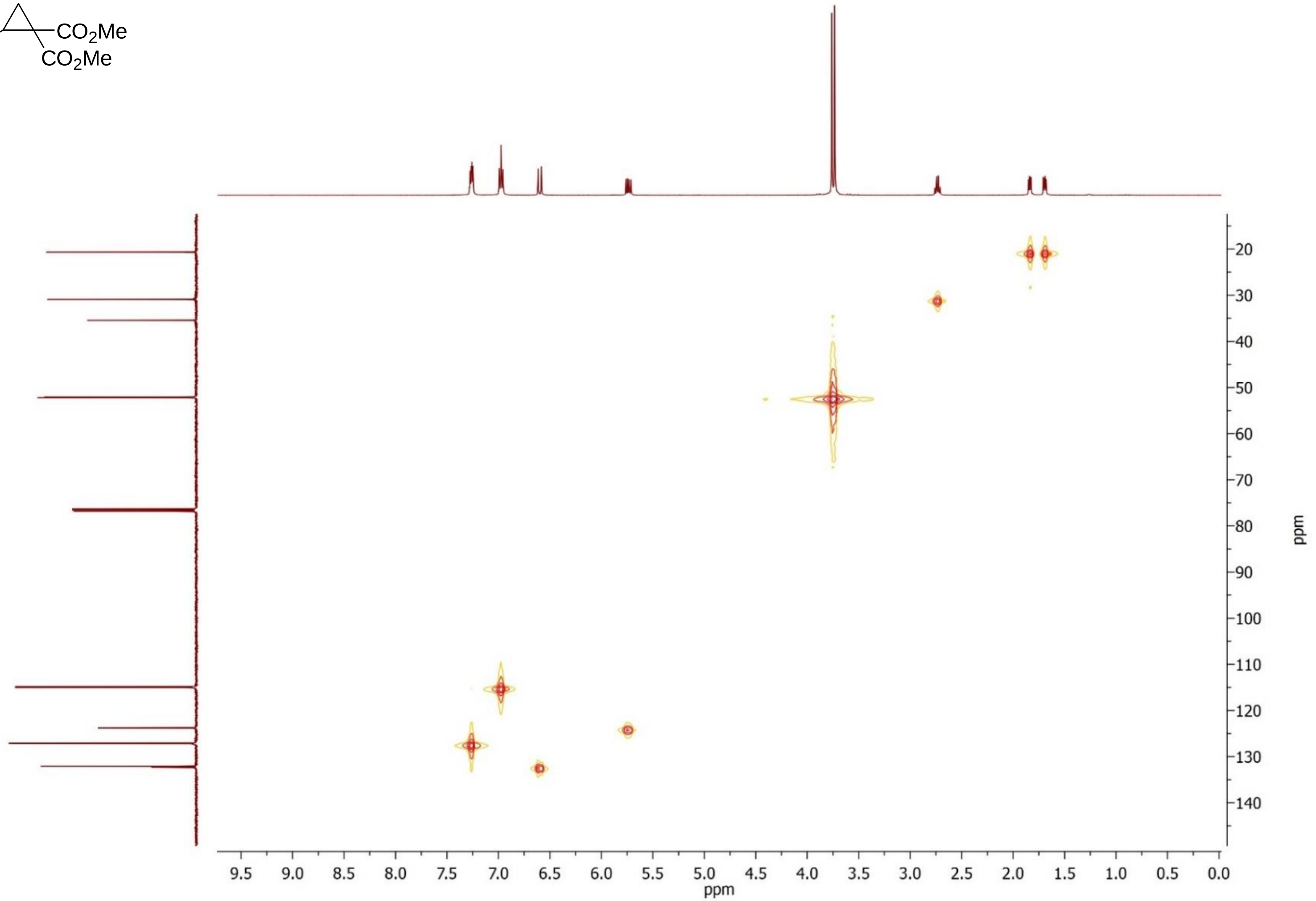

4-(6,6-Dimethyl-4,8-dioxo-5,7-dioxaspiro[2.5]octan-1-yl)benzonitrile (1af)

^{1}H NMR (500 MHz, $CDCl_3$)

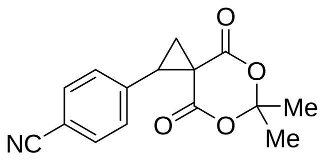

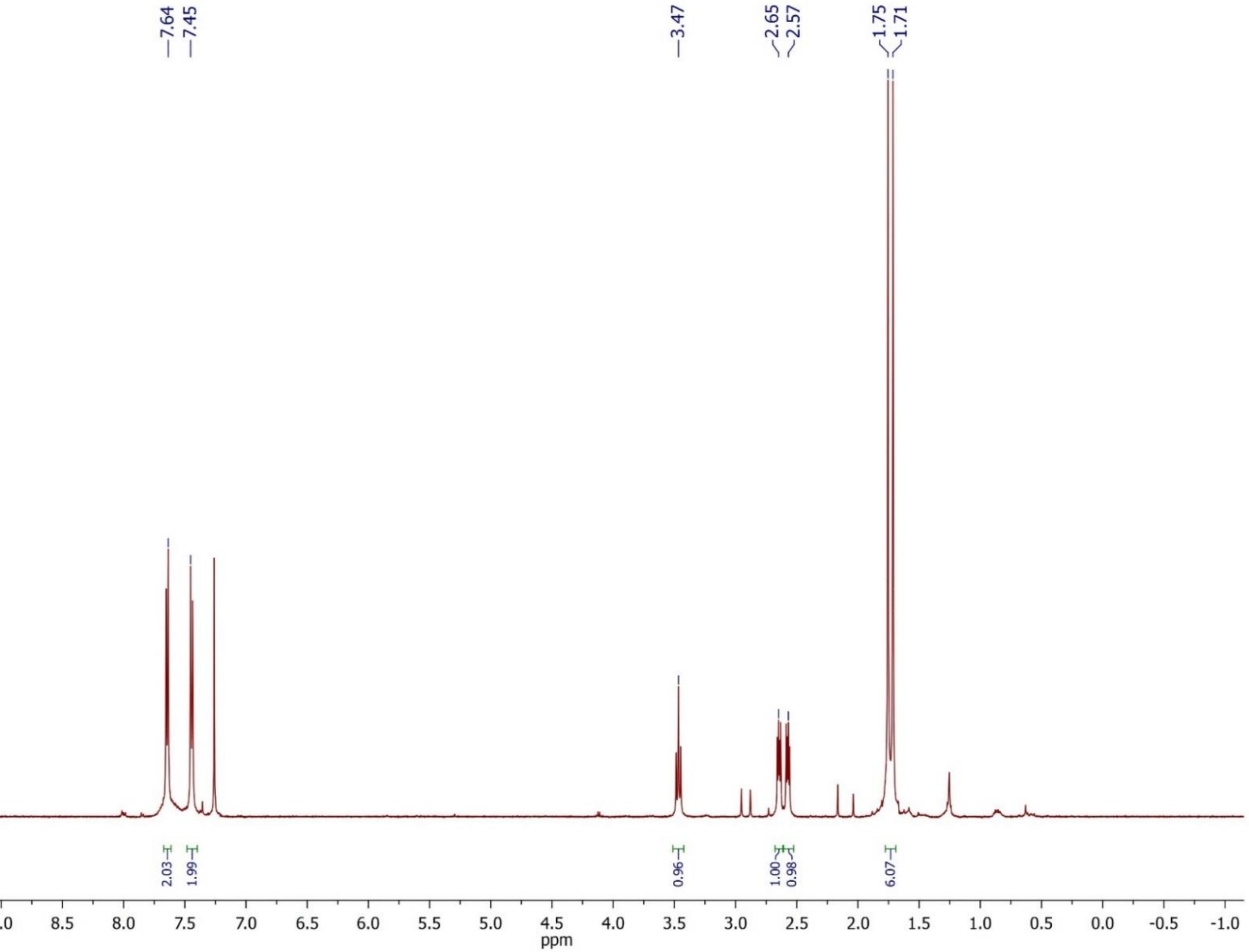

4-(6,6-Dimethyl-4,8-dioxo-5,7-dioxaspiro[2.5]octan-1-yl)benzonitrile (1af)

^{13}C NMR (126 MHz, acetone-d$_6$)

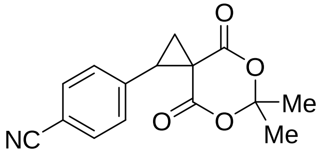

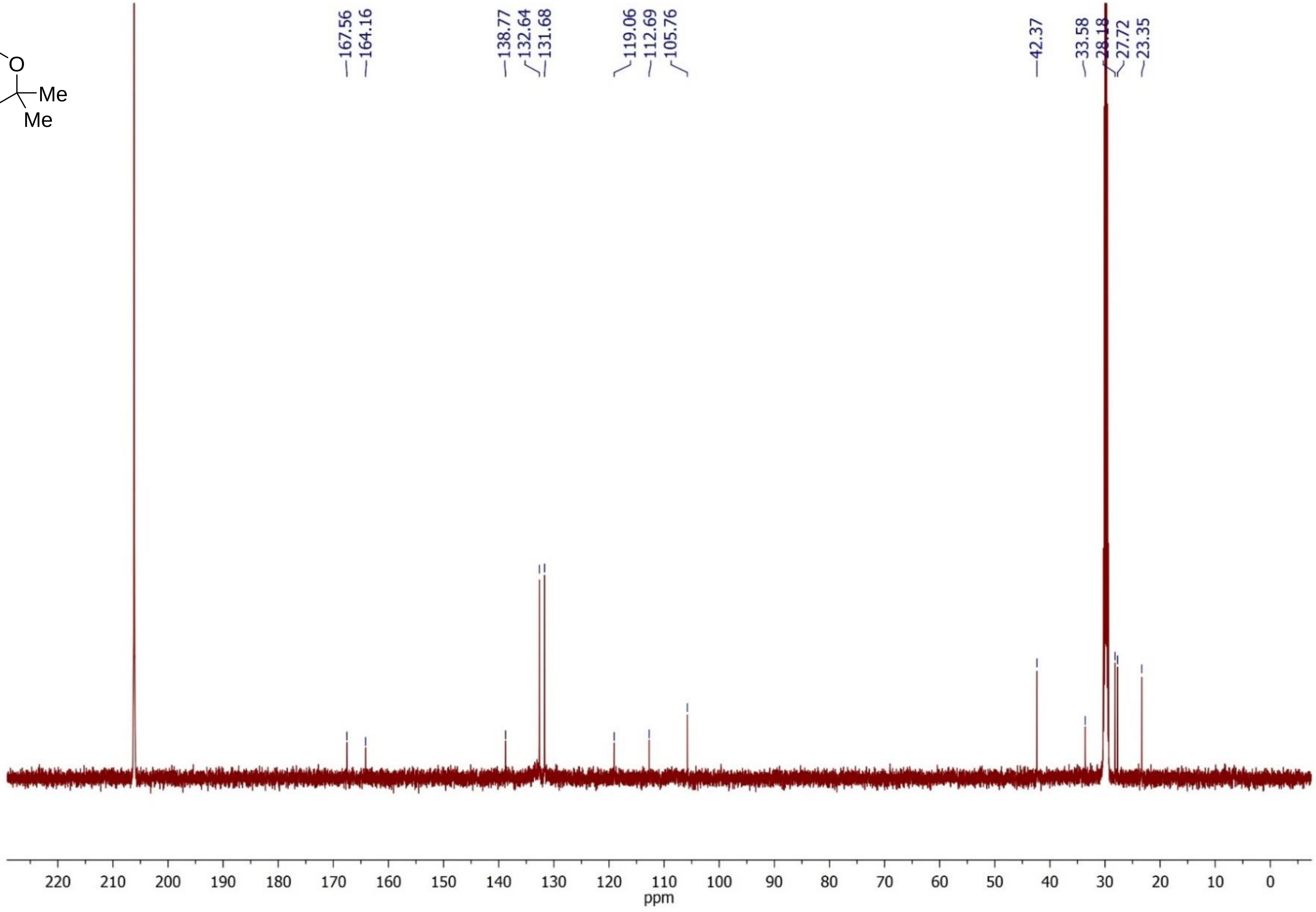

4-(6,6-Dimethyl-4,8-dioxo-5,7-dioxaspiro[2.5]octan-1-yl)benzonitrile (1af)

^{1}H–^{13}C HSQC (acetone-d_6)

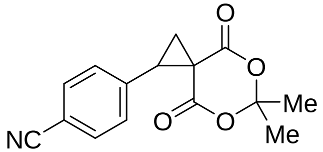

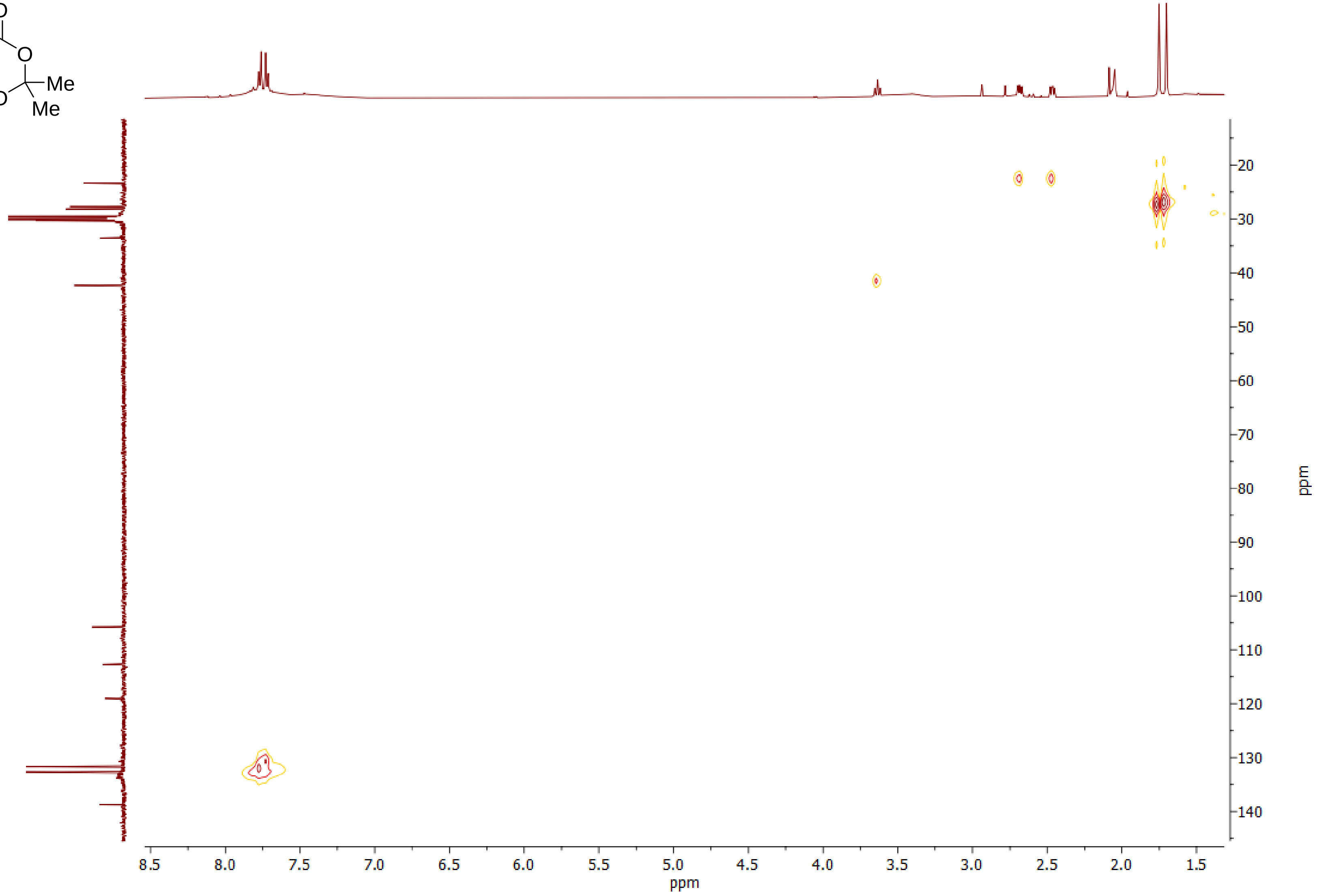

4-(6,6-Dimethyl-4,8-dioxo-5,7-dioxaspiro[2.5]octan-1-yl)benzonitrile (1af)

$^{1}H–^{13}C$ HMBC (acetone-d_6)

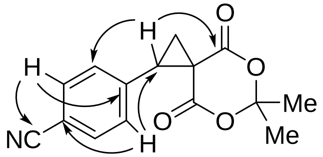

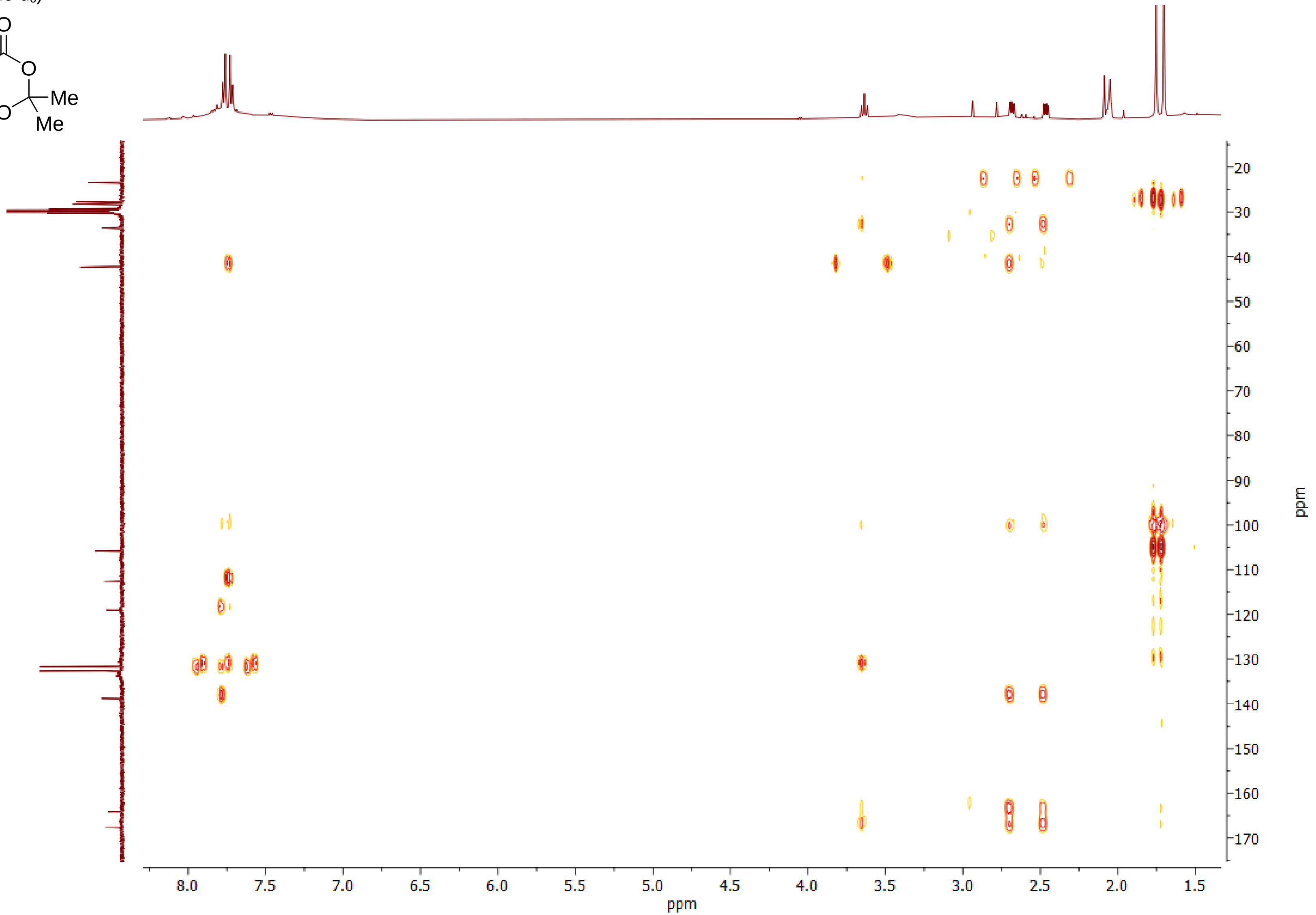

Dimethyl 5-(3,4-dimethoxyphenyl)-2-thioxopyrrolidine-3,3-dicarboxylate (2a)

^{1}H NMR (500 MHz, $CDCl_3$)

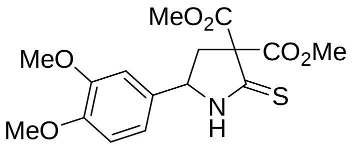

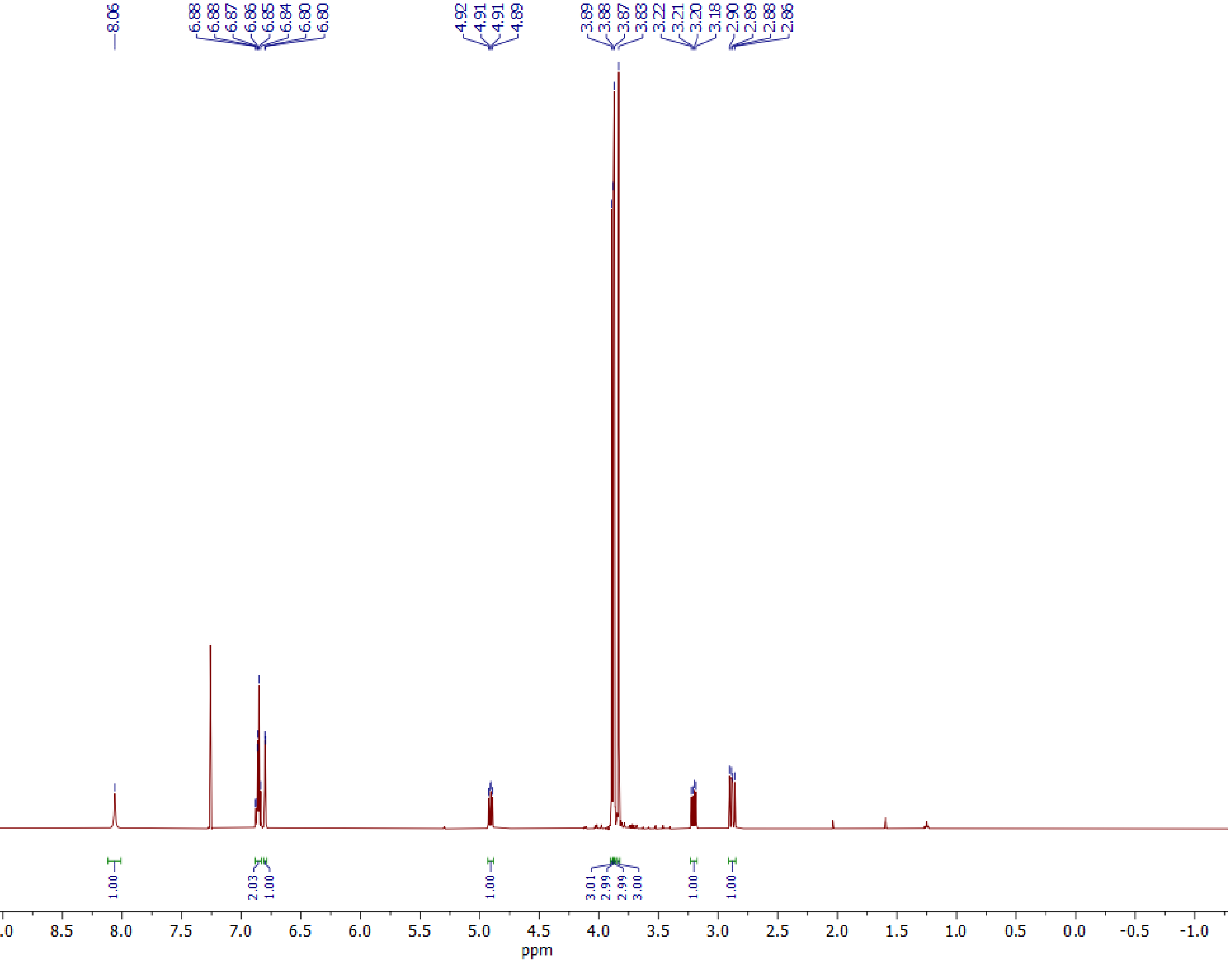

Dimethyl 5-(3,4-dimethoxyphenyl)-2-thioxopyrrolidine-3,3-dicarboxylate (2a)

^{13}C NMR (126 MHz, $CDCl_3$)

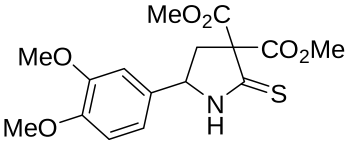

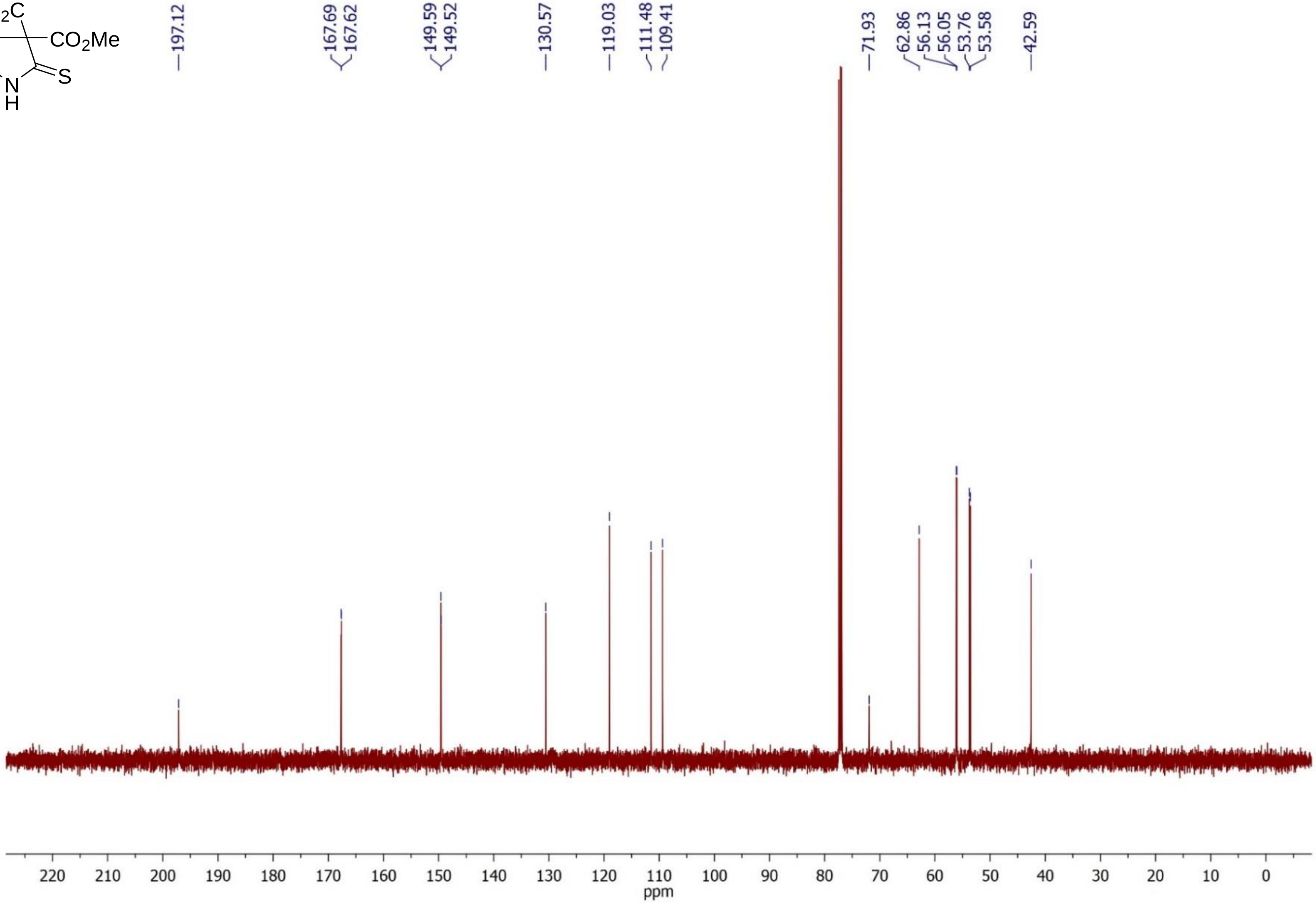

Dimethyl 5-(4-methoxyphenyl)-2-thioxopyrrolidine-3,3-dicarboxylate (2b)

^{1}H NMR (500 MHz, $CDCl_3$)

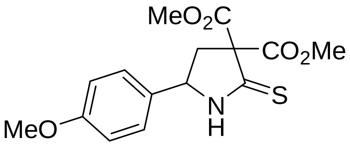

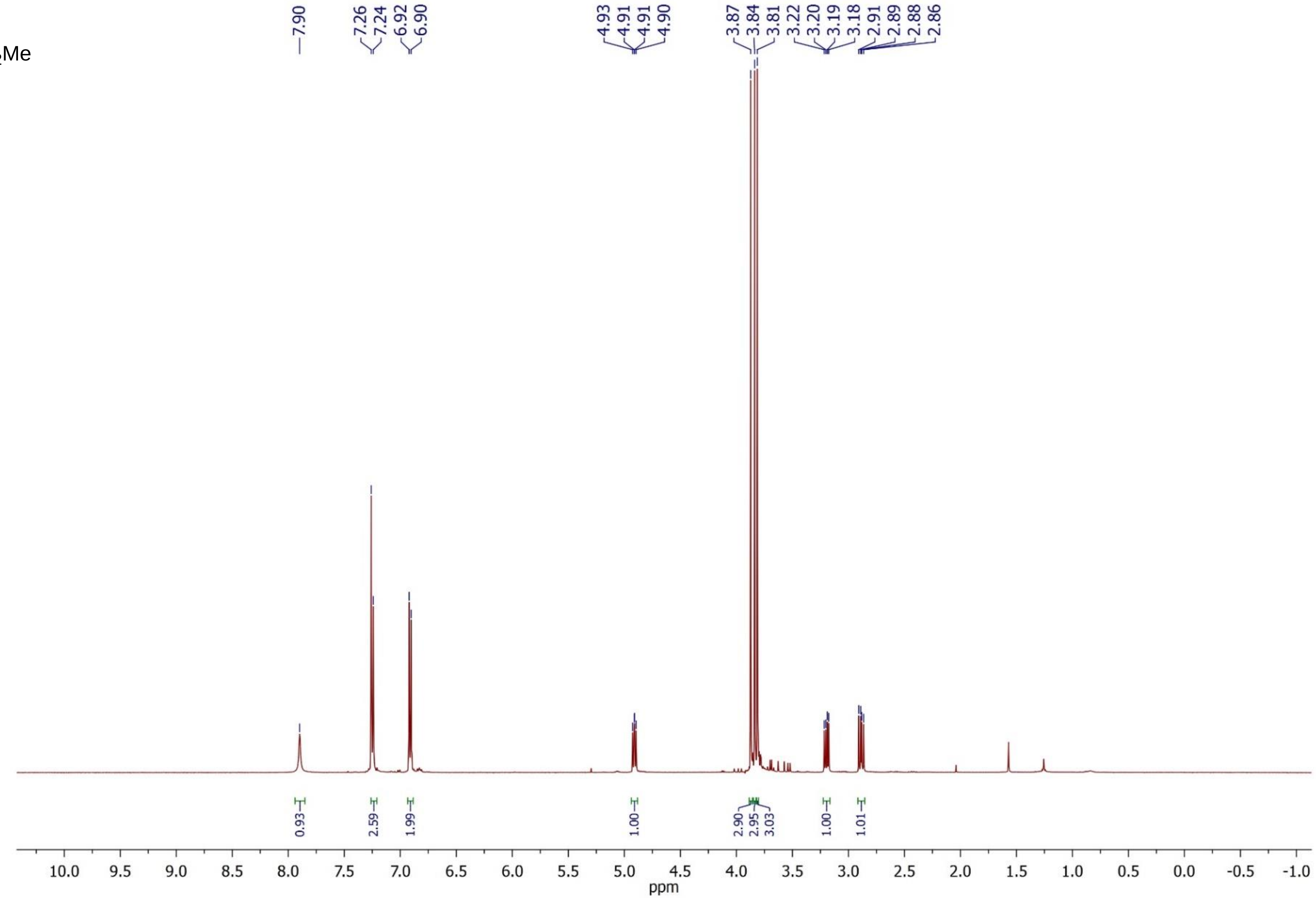

Dimethyl 5-(4-methoxyphenyl)-2-thioxopyrrolidine-3,3-dicarboxylate (2b)

^{1}H NMR (500 MHz, DMSO-d_6)

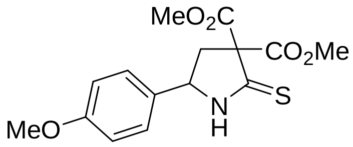

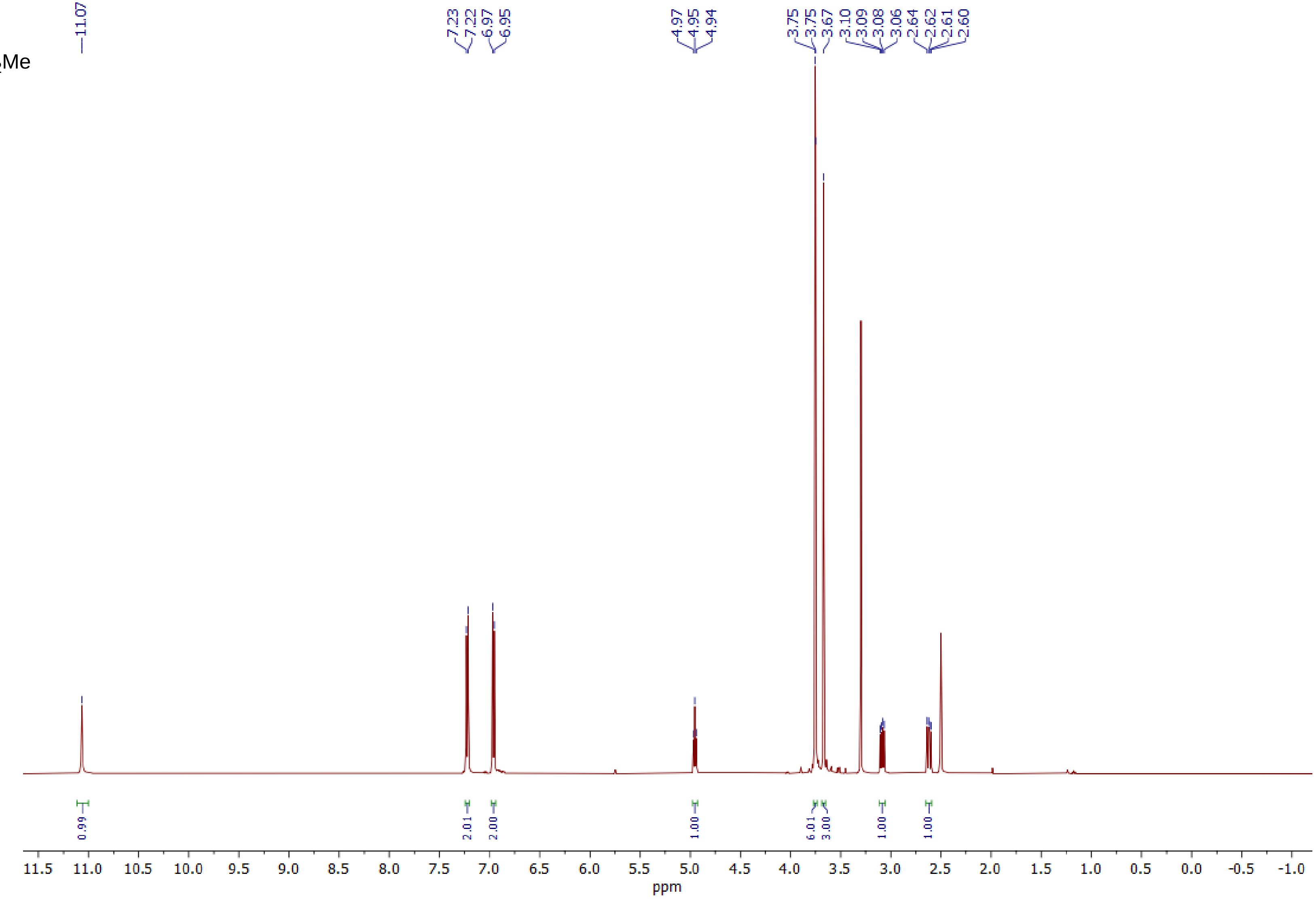

Dimethyl 5-(4-methoxyphenyl)-2-thioxopyrrolidine-3,3-dicarboxylate (2b)

^{13}C NMR (126 MHz, DMSO-d_6)

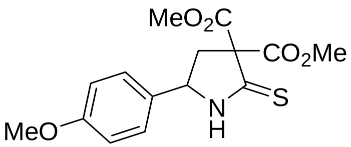

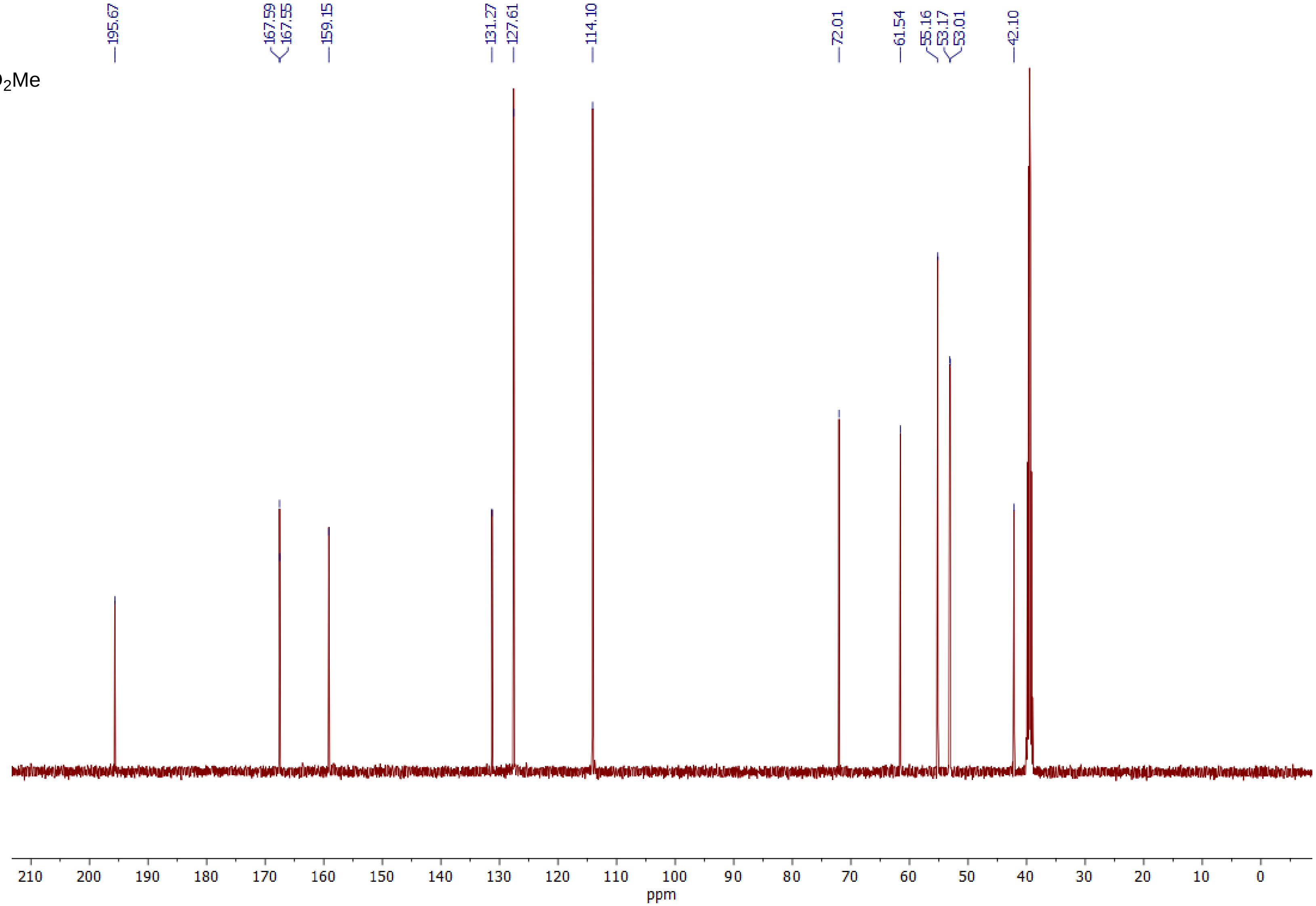

Dimethyl 5-(3,4,5-trimethoxyphenyl)-2-thioxopyrrolidine-3,3-dicarboxylate (2c)

^{1}H NMR (500 MHz, $CDCl_3$)

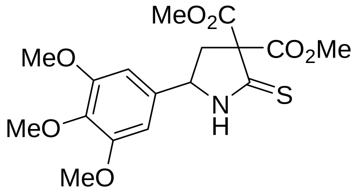

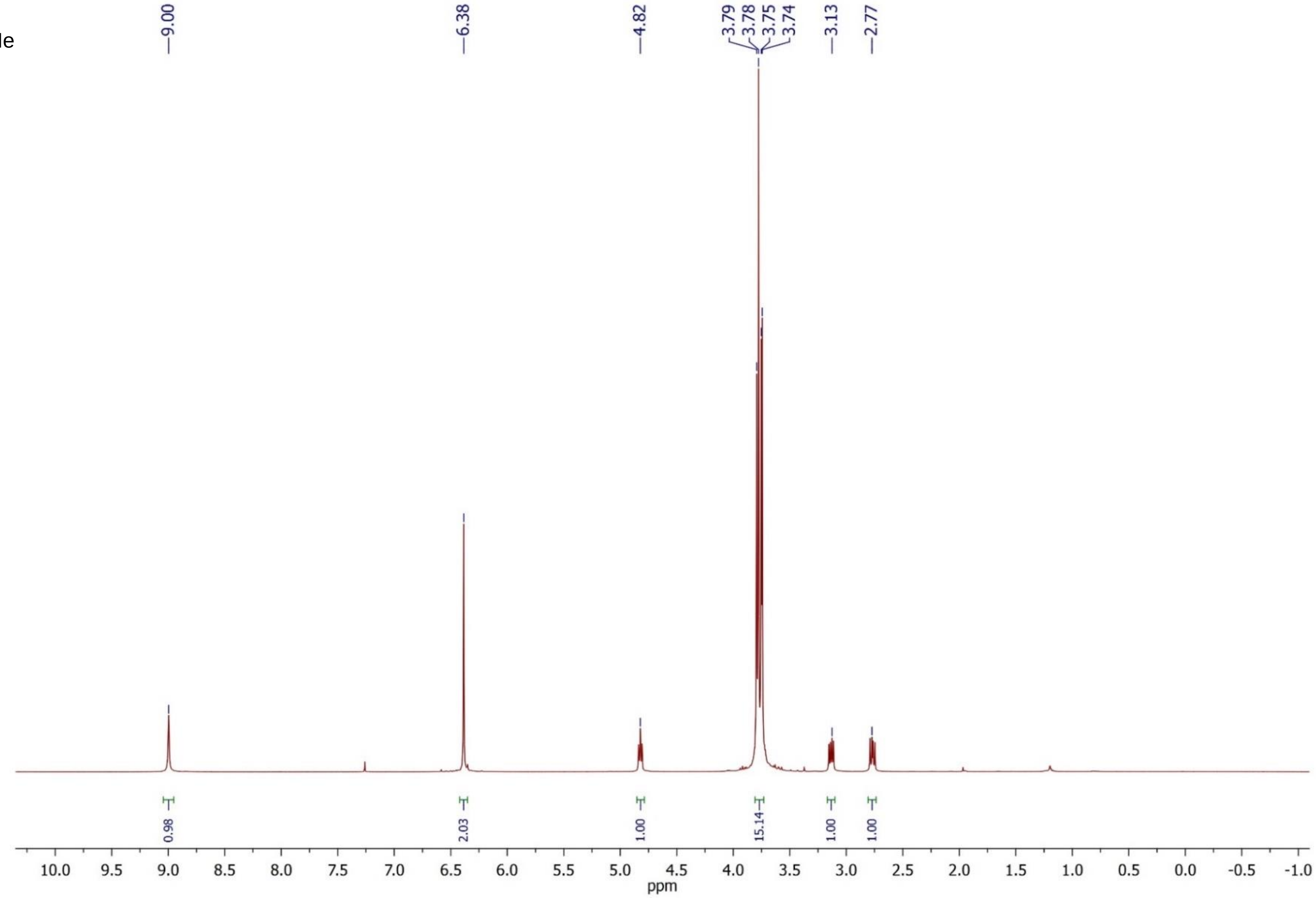

Dimethyl 5-(3,4,5-trimethoxyphenyl)-2-thioxopyrrolidine-3,3-dicarboxylate (2c)

^{13}C NMR (126 MHz, $CDCl_3$)

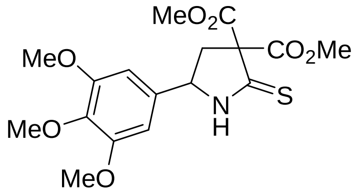

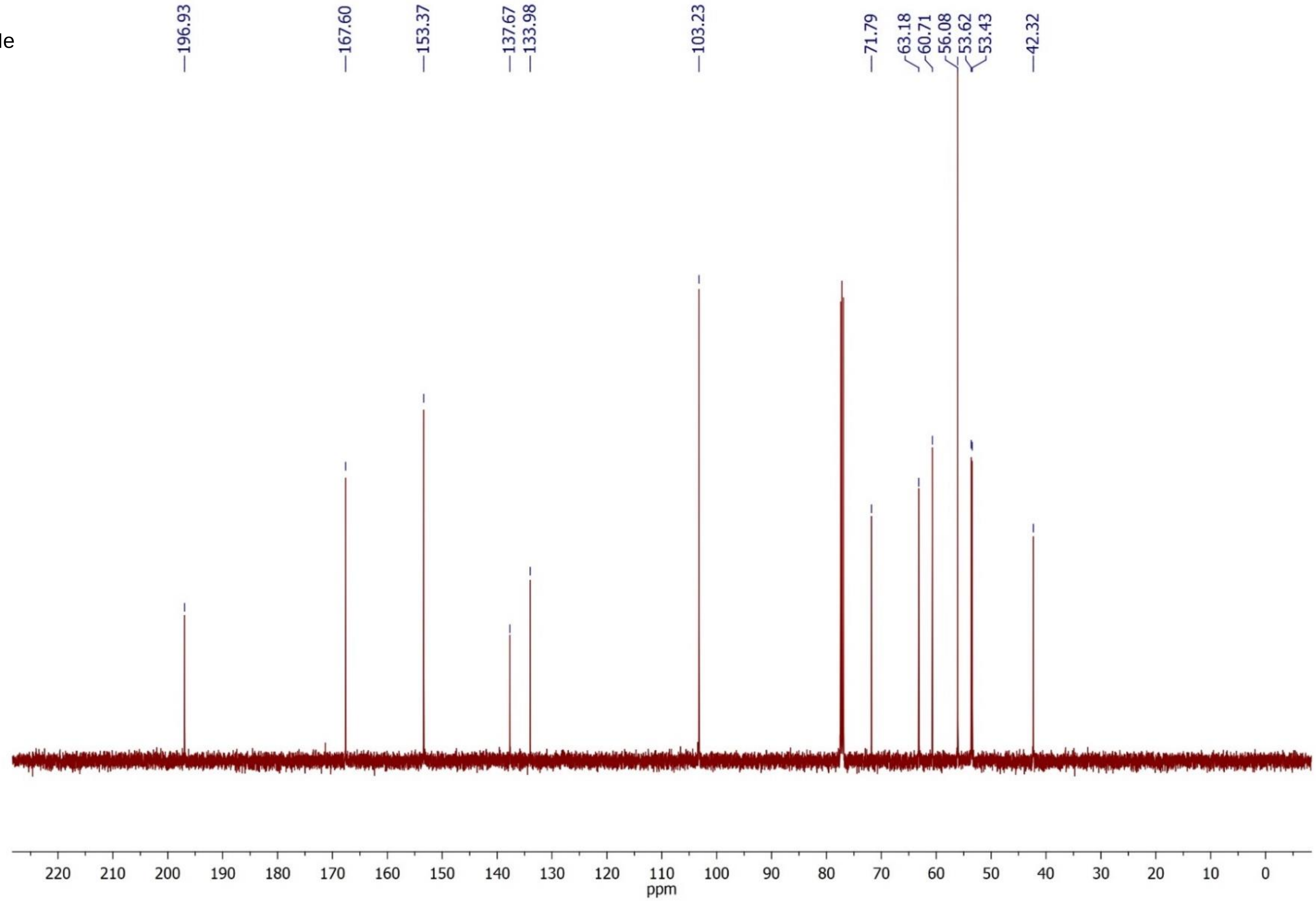

Dimethyl 5-(3,4,5-trimethoxyphenyl)-2-thioxopyrrolidine-3,3-dicarboxylate (2c)

^{1}H–^{13}C HSQC ($CDCl_3$)

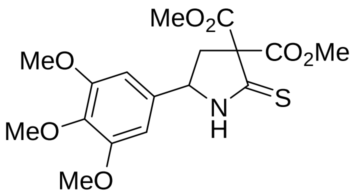

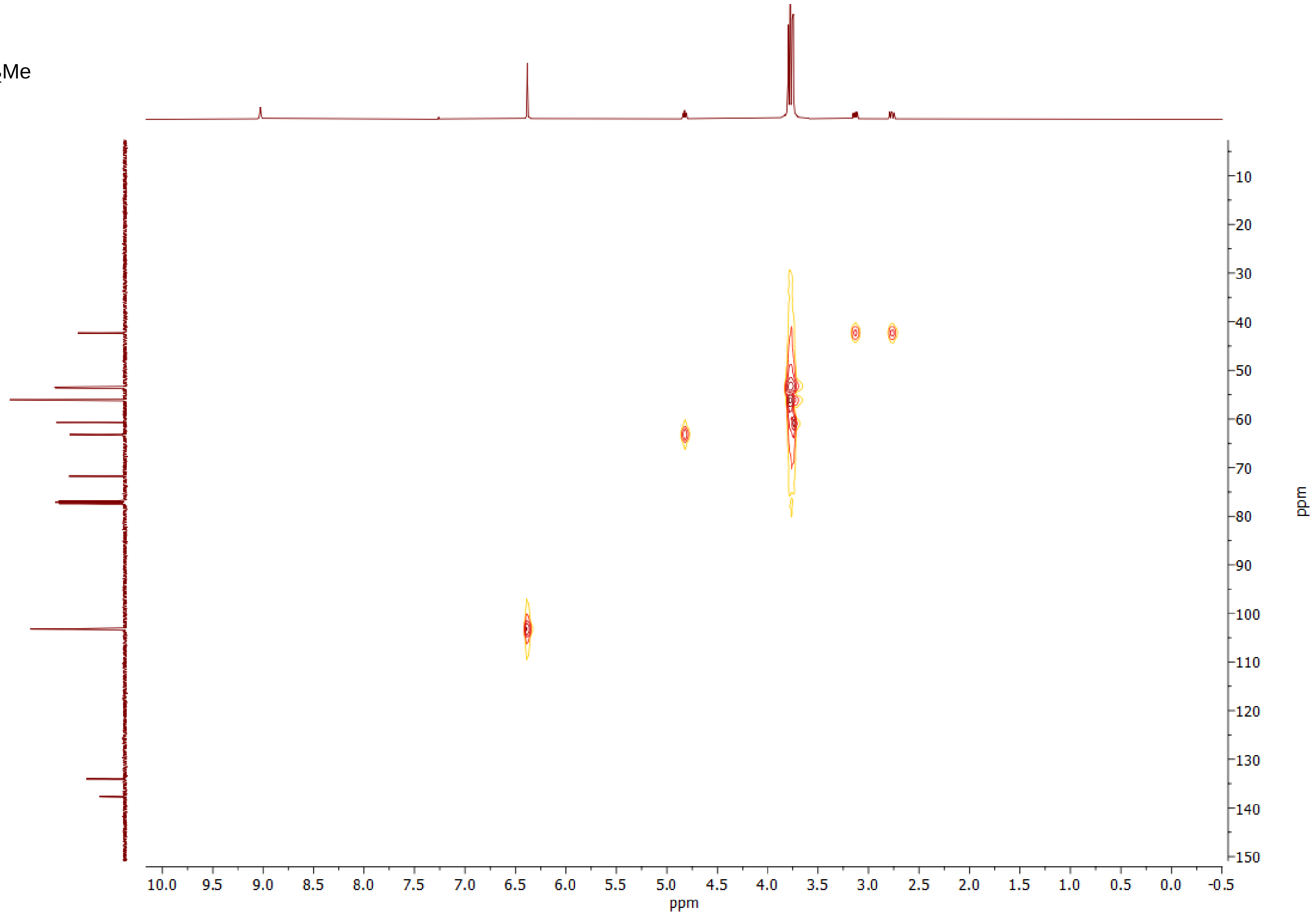

Dimethyl 5-(1,3-benzodioxol-5-yl)-2-thioxopyrrolidine-3,3-dicarboxylate (2d)

^{1}H NMR (500 MHz, DMSO-d_6)

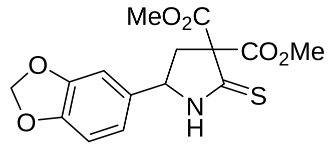

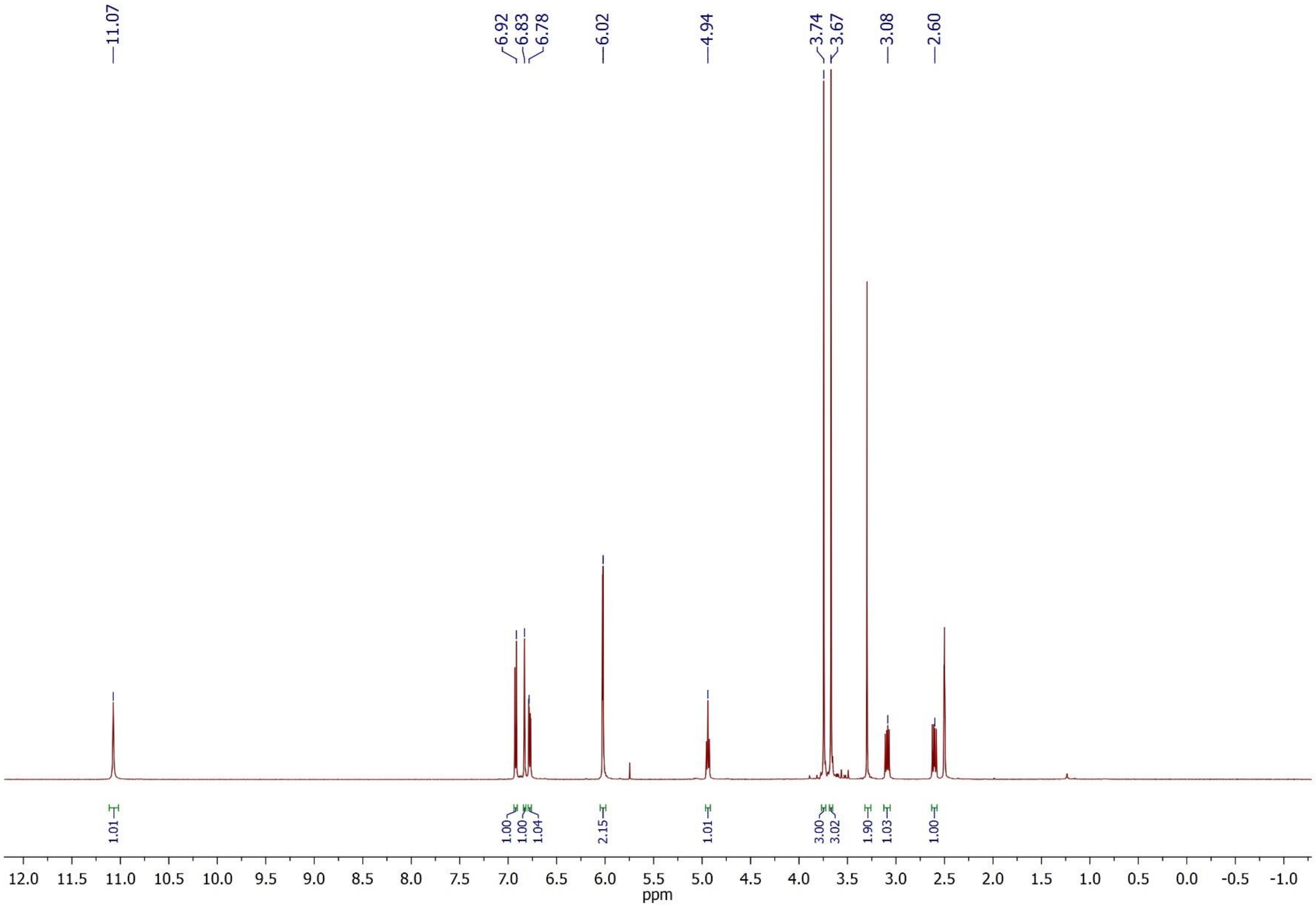

Dimethyl 5-(1,3-benzodioxol-5-yl)-2-thioxopyrrolidine-3,3-dicarboxylate (2d)

^{13}C NMR (126 MHz, DMSO-d_6)

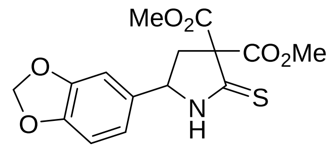

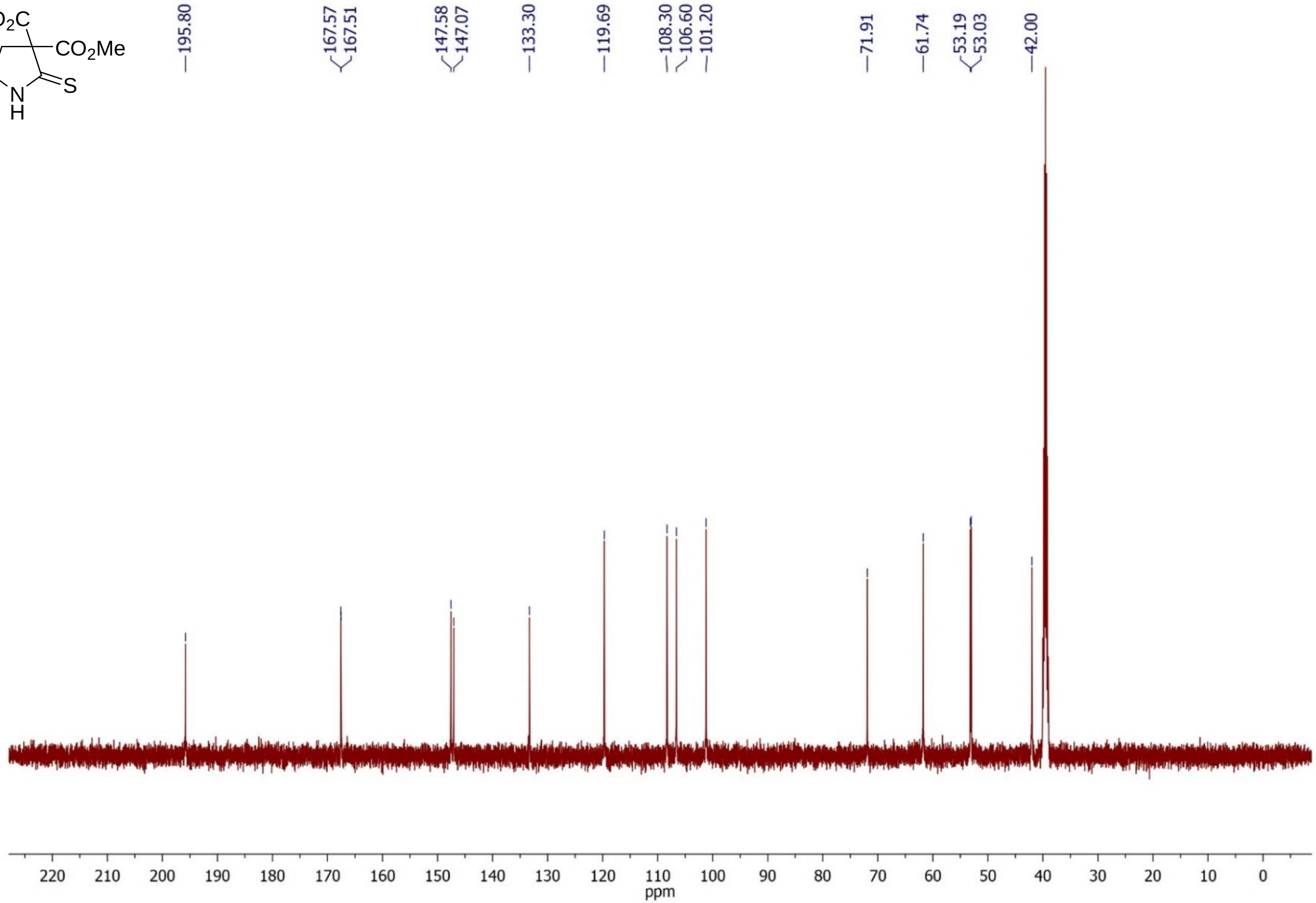

Dimethyl 5-(2,3-dihydrobenzo[*b*][1,4]dioxin-6-yl)-2-thioxopyrrolidine-3,3-dicarboxylate (2e)

^{1}H NMR (500 MHz, DMSO-d_6)

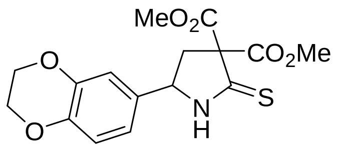

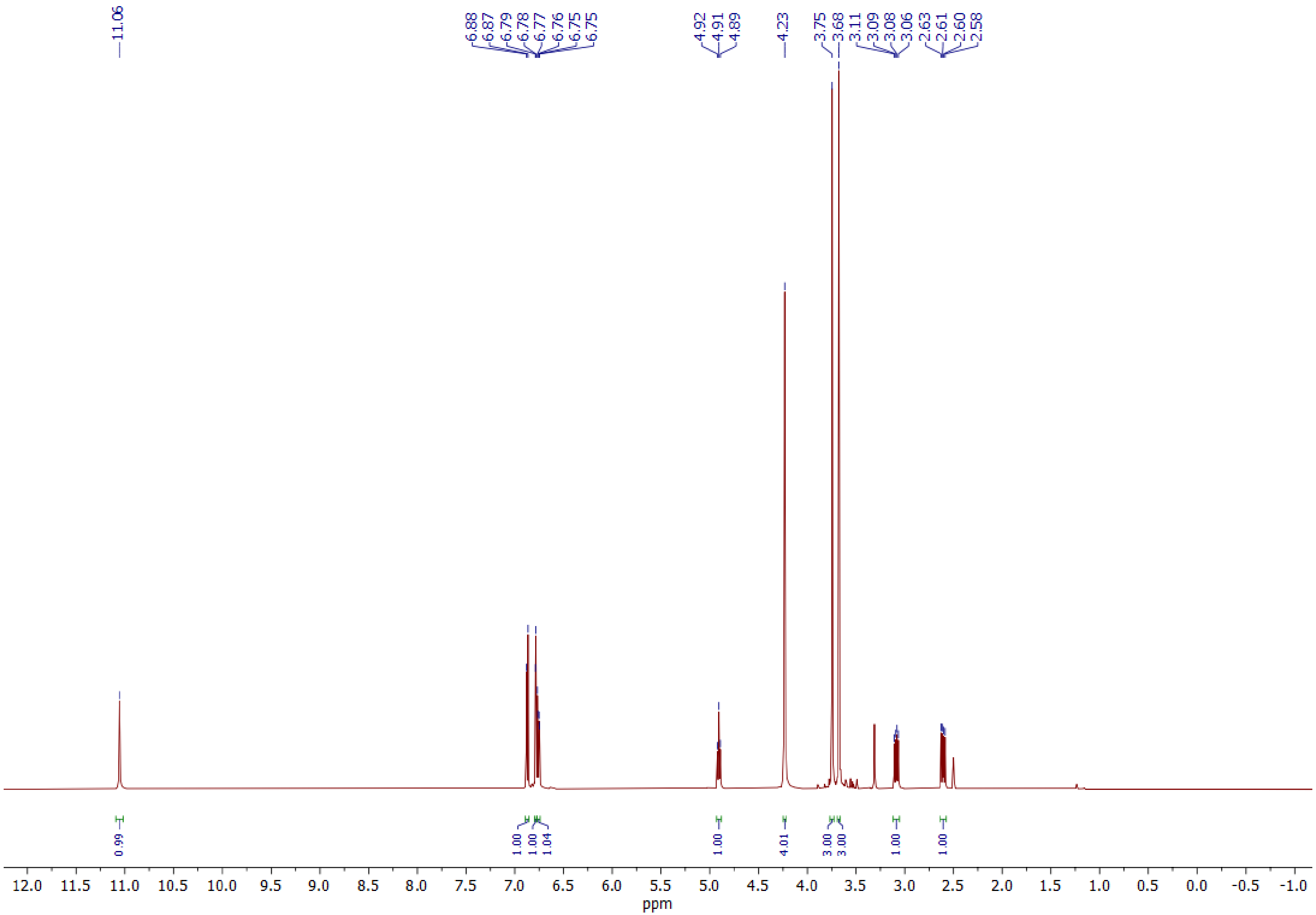

Dimethyl 5-(2,3-dihydrobenzo[*b*][1,4]dioxin-6-yl)-2-thioxopyrrolidine-3,3-dicarboxylate (2e)

^{13}C NMR (126 MHz, DMSO-d_6)

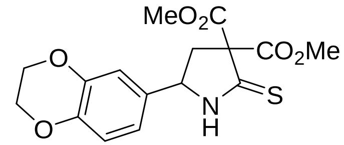

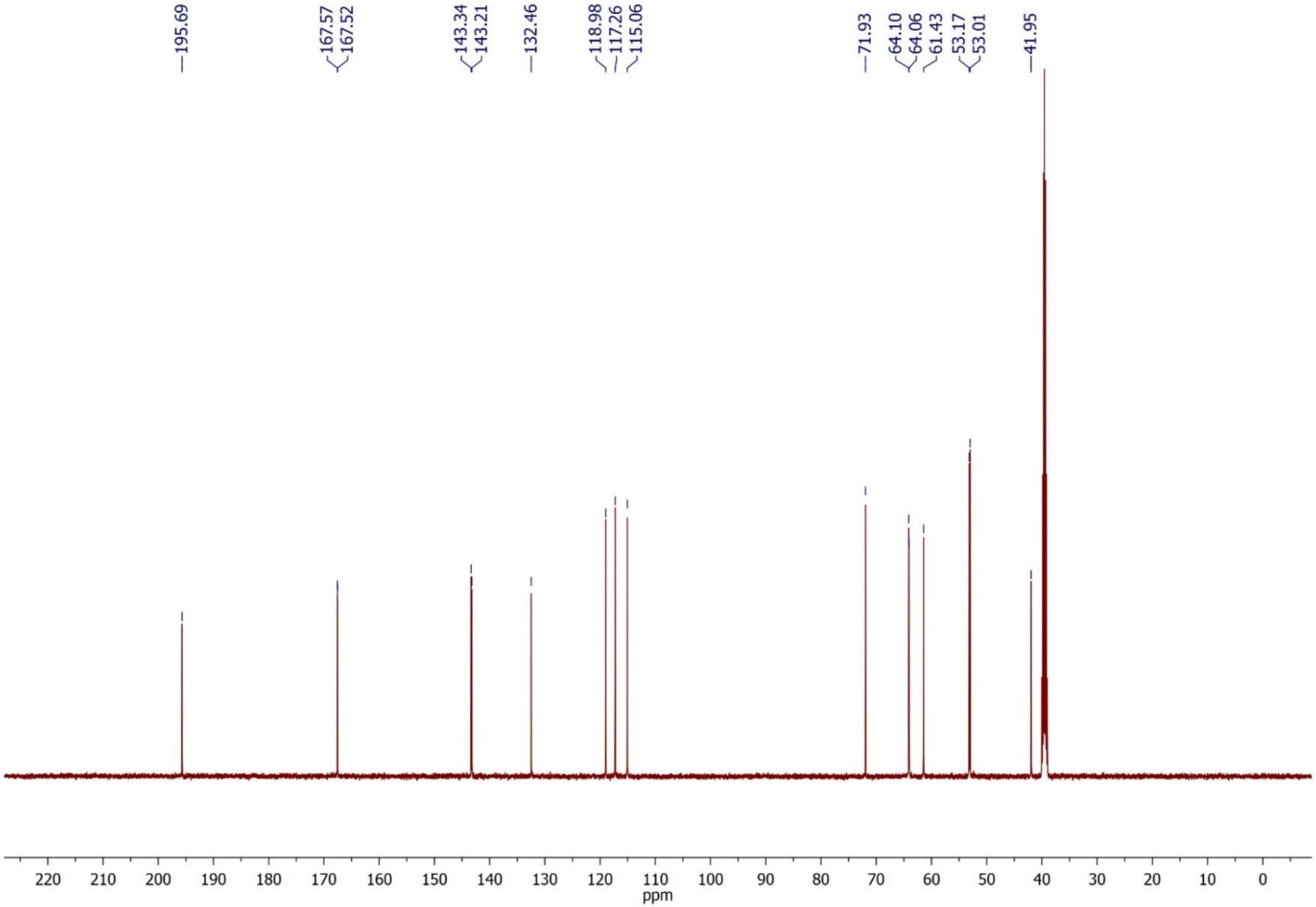

Dimethyl 5-(2,4-dimethoxyphenyl)-2-thioxopyrrolidine-3,3-dicarboxylate (2f)

^{1}H NMR (500 MHz, $CDCl_3$)

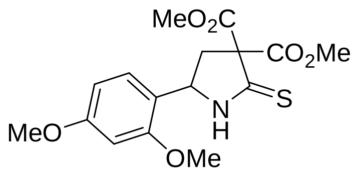

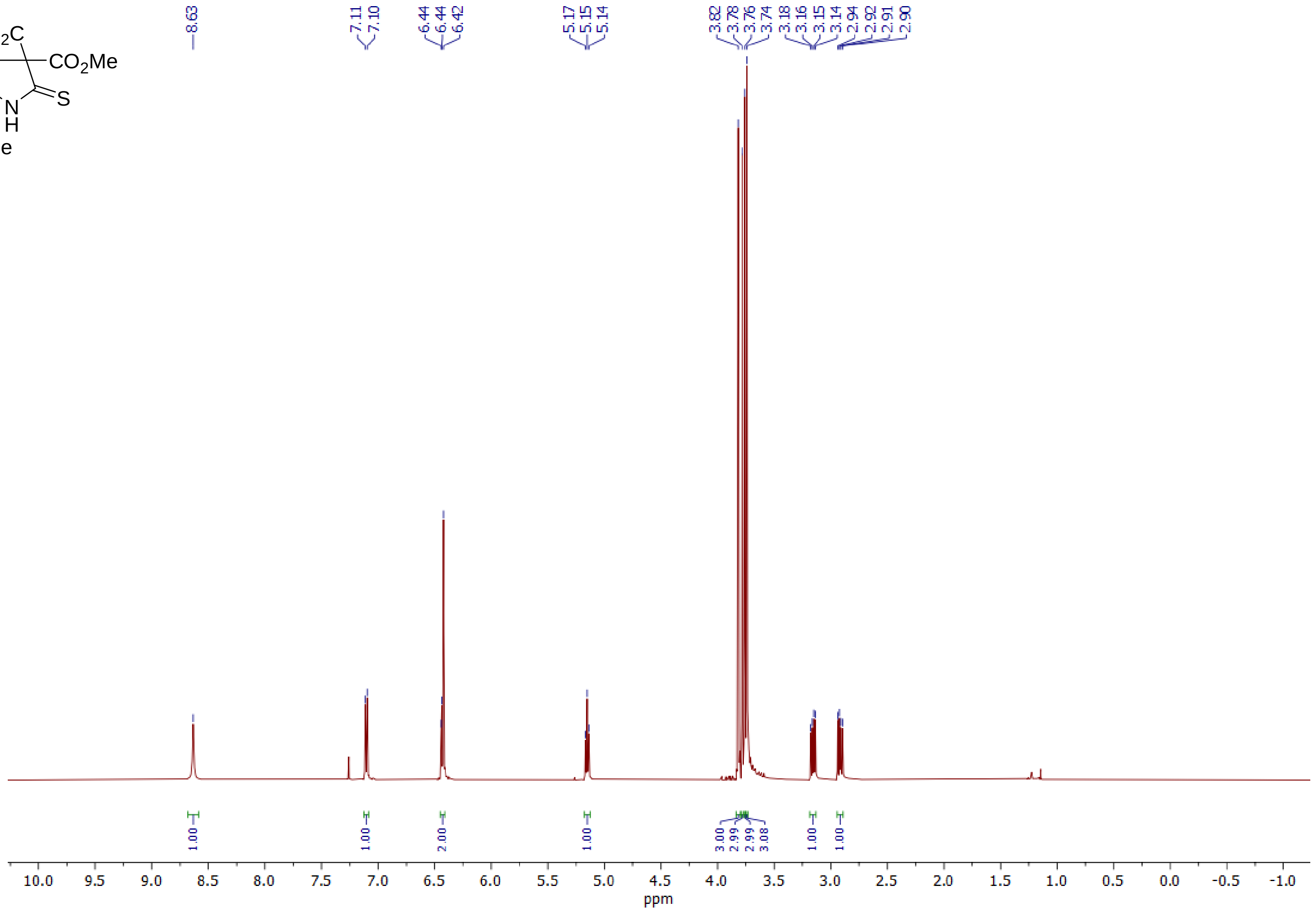

Dimethyl 5-(2,4-dimethoxyphenyl)-2-thioxopyrrolidine-3,3-dicarboxylate (2f)

^{13}C NMR (126 MHz, $CDCl_3$)

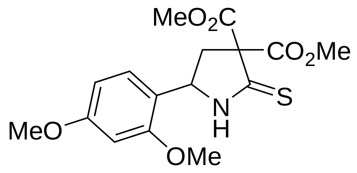

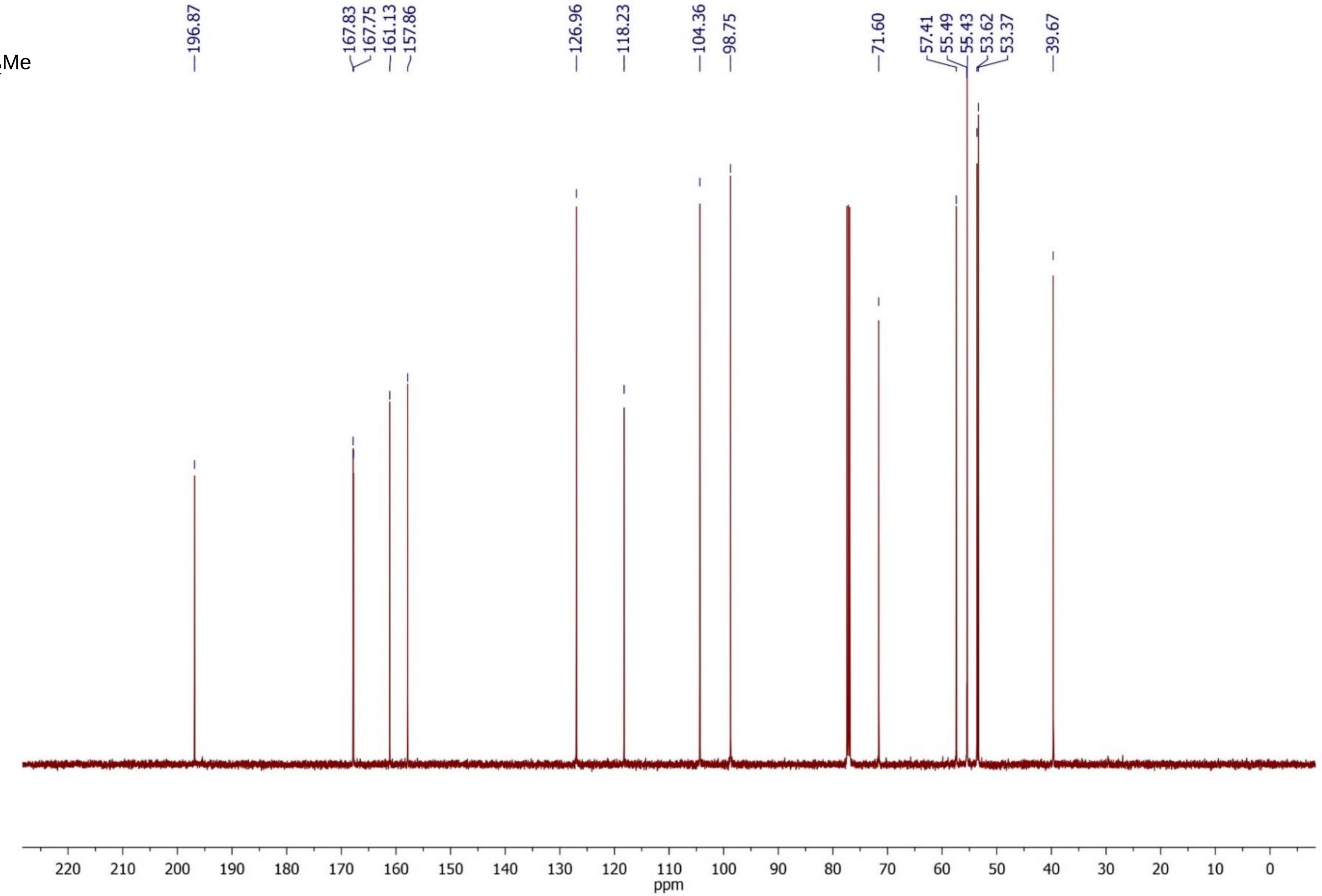

Dimethyl 5-(2,4-dimethoxyphenyl)-2-thioxopyrrolidine-3,3-dicarboxylate (2f)

^{1}H–^{13}C HSQC ($CDCl_3$)

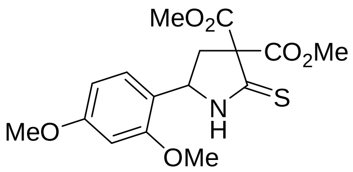

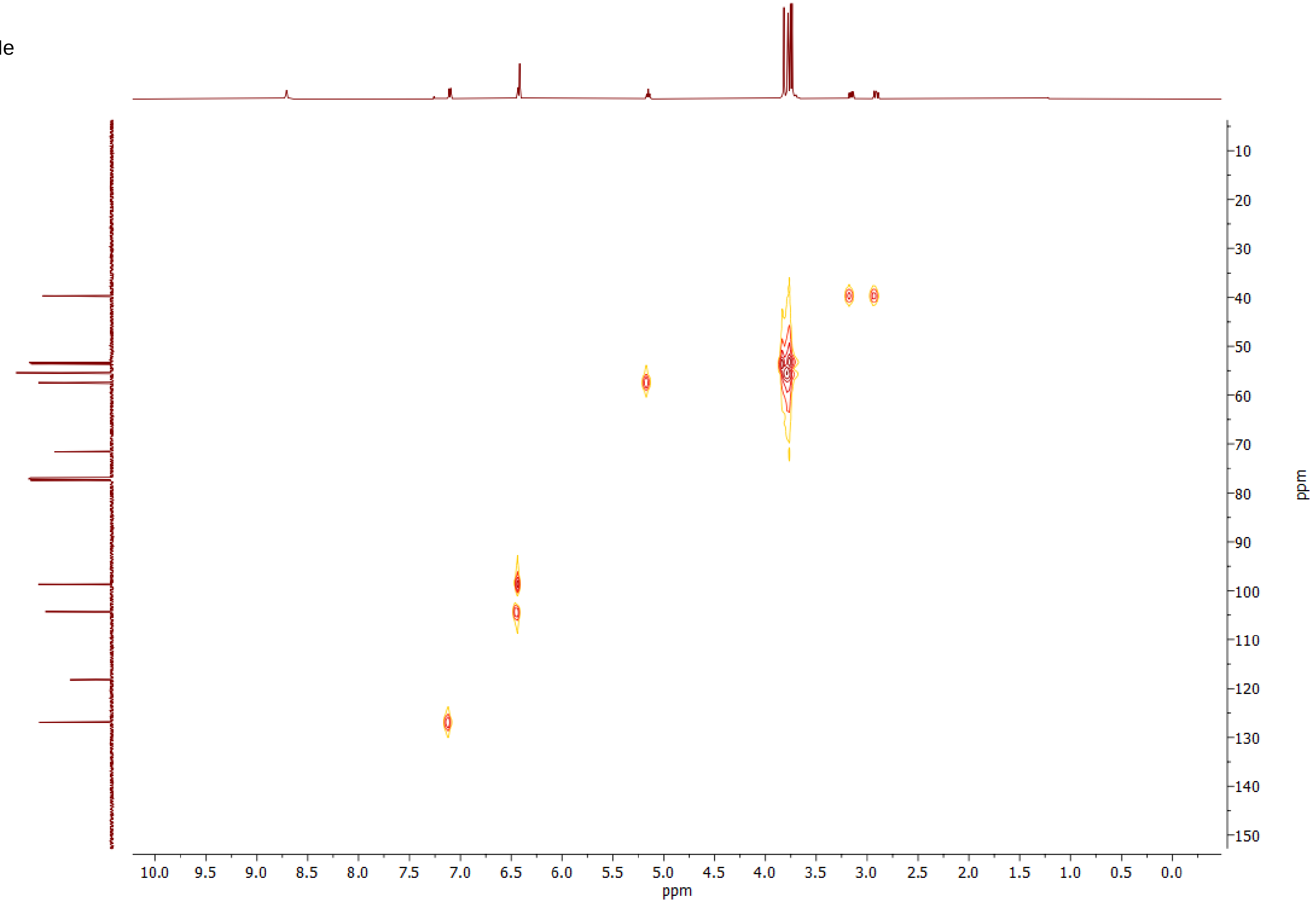

Dimethyl 5-(2,3,4-trimethoxyphenyl)-2-thioxopyrrolidine-3,3-dicarboxylate (2g)

^{1}H NMR (500 MHz, $CDCl_3$)

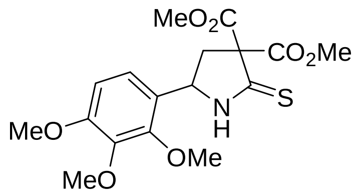

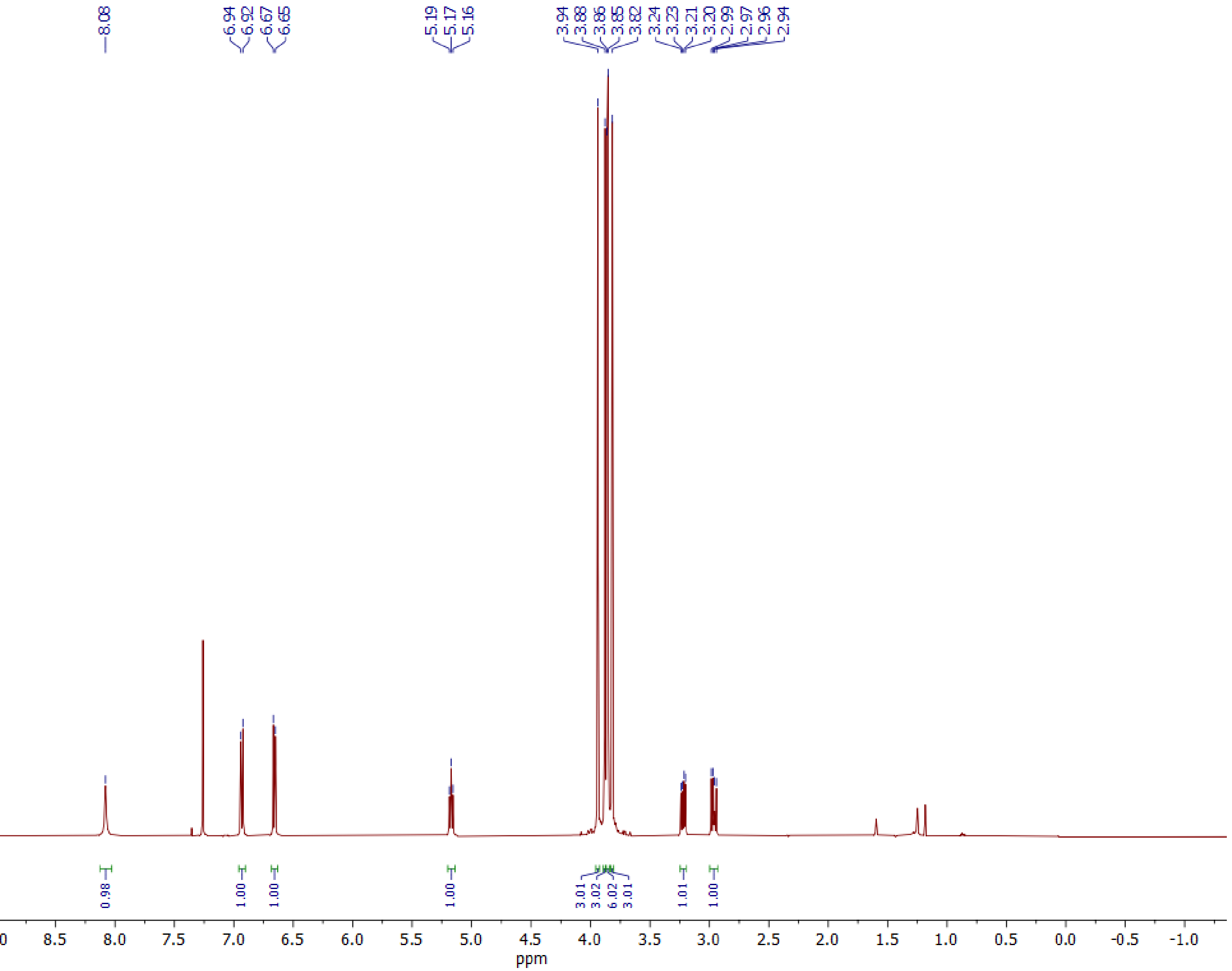

Dimethyl 5-(2,3,4-trimethoxyphenyl)-2-thioxopyrrolidine-3,3-dicarboxylate (2g)

^{13}C NMR (126 MHz, $CDCl_3$)

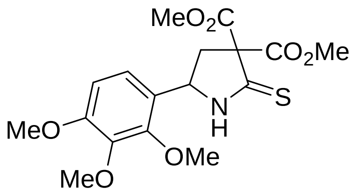

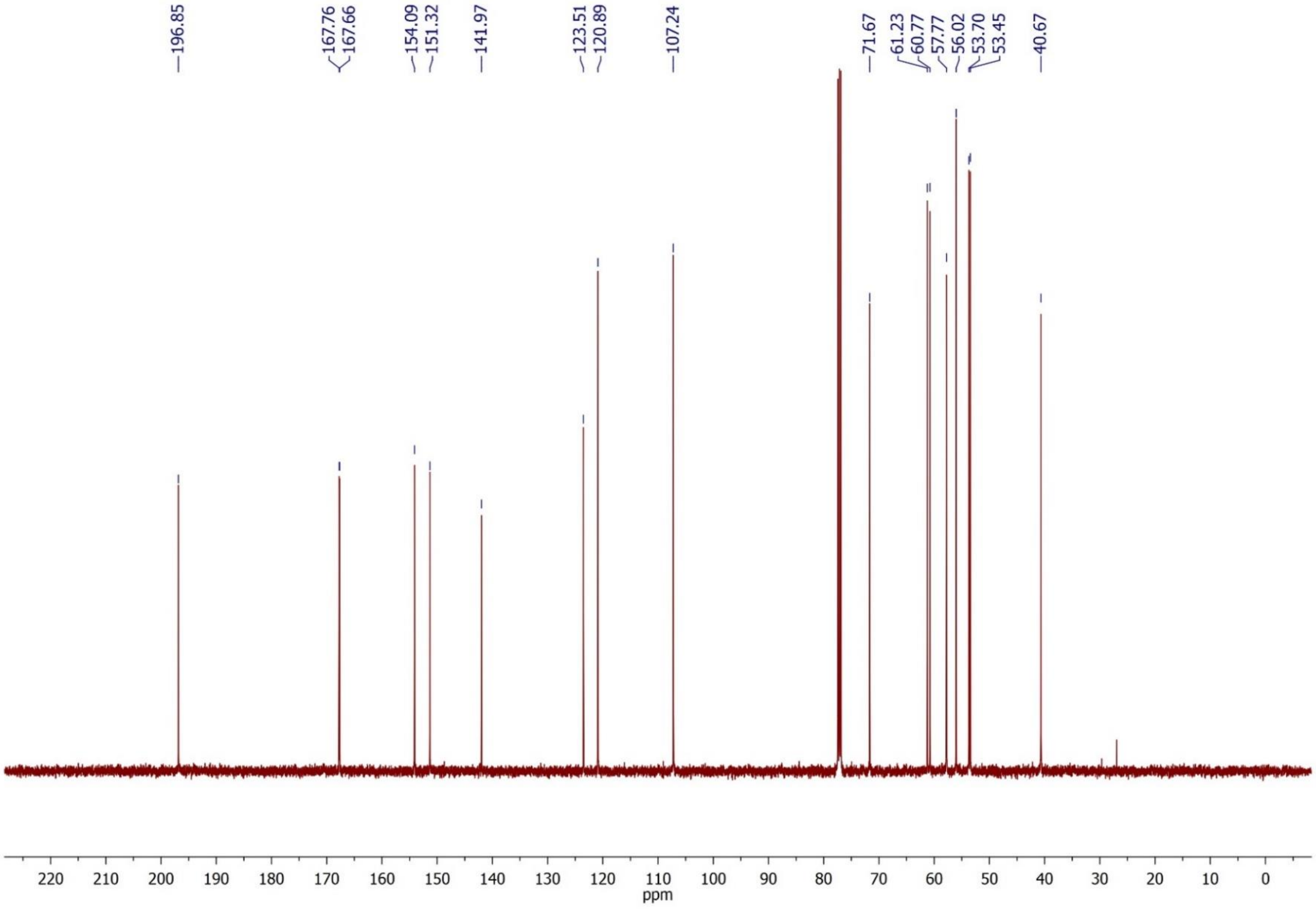

Dimethyl 5-(2,3,4-trimethoxyphenyl)-2-thioxopyrrolidine-3,3-dicarboxylate (2g)

^1H–^{13}C HSQC ($CDCl_3$)

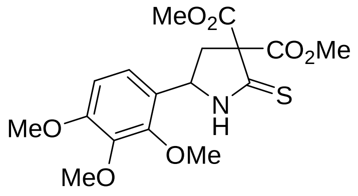

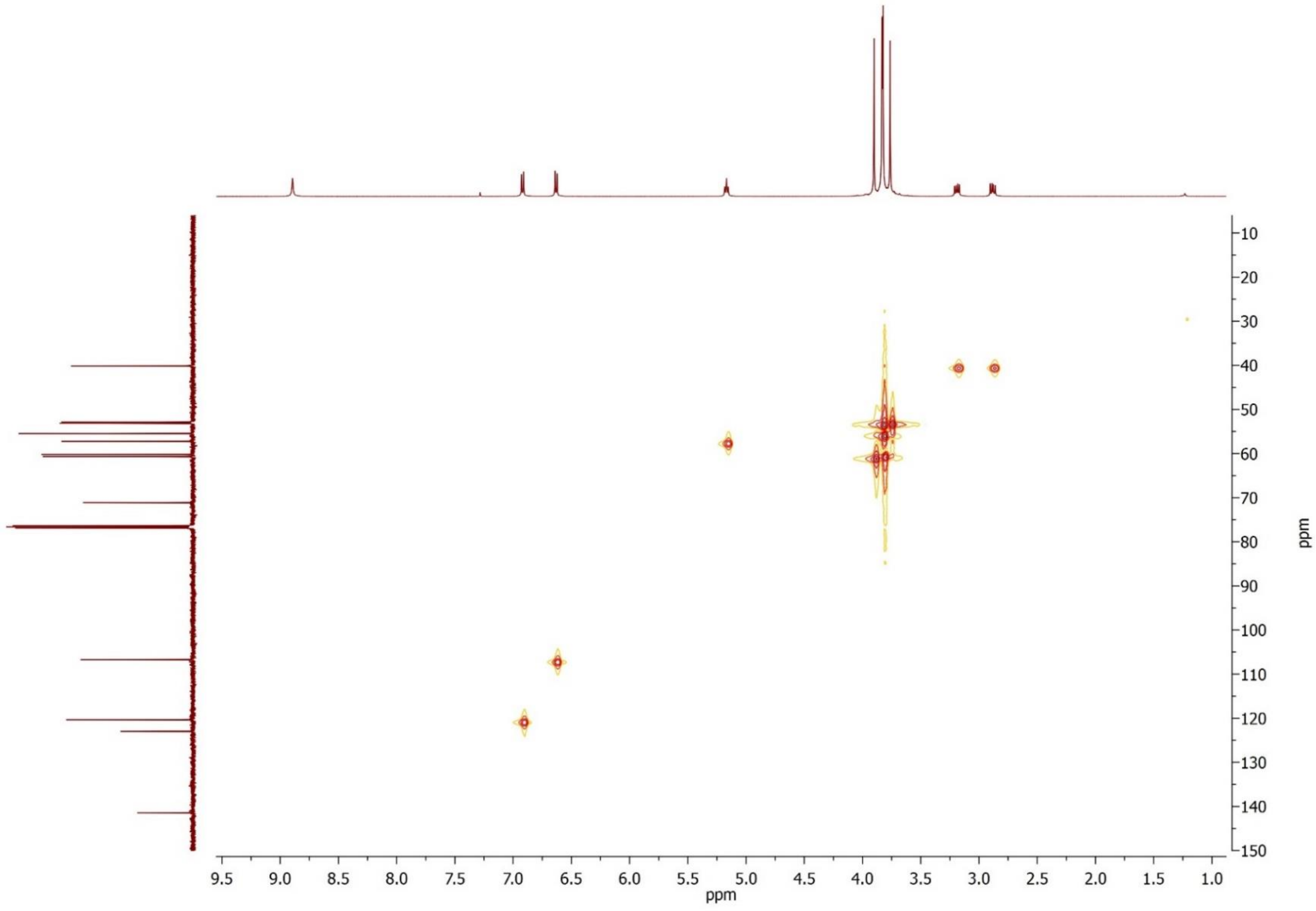

Dimethyl 5-(2-methoxyphenyl)-2-thioxopyrrolidine-3,3-dicarboxylate (2h)

^{1}H NMR (500 MHz, $CDCl_3$)

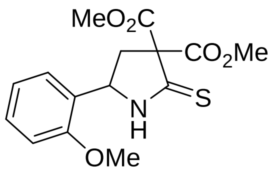

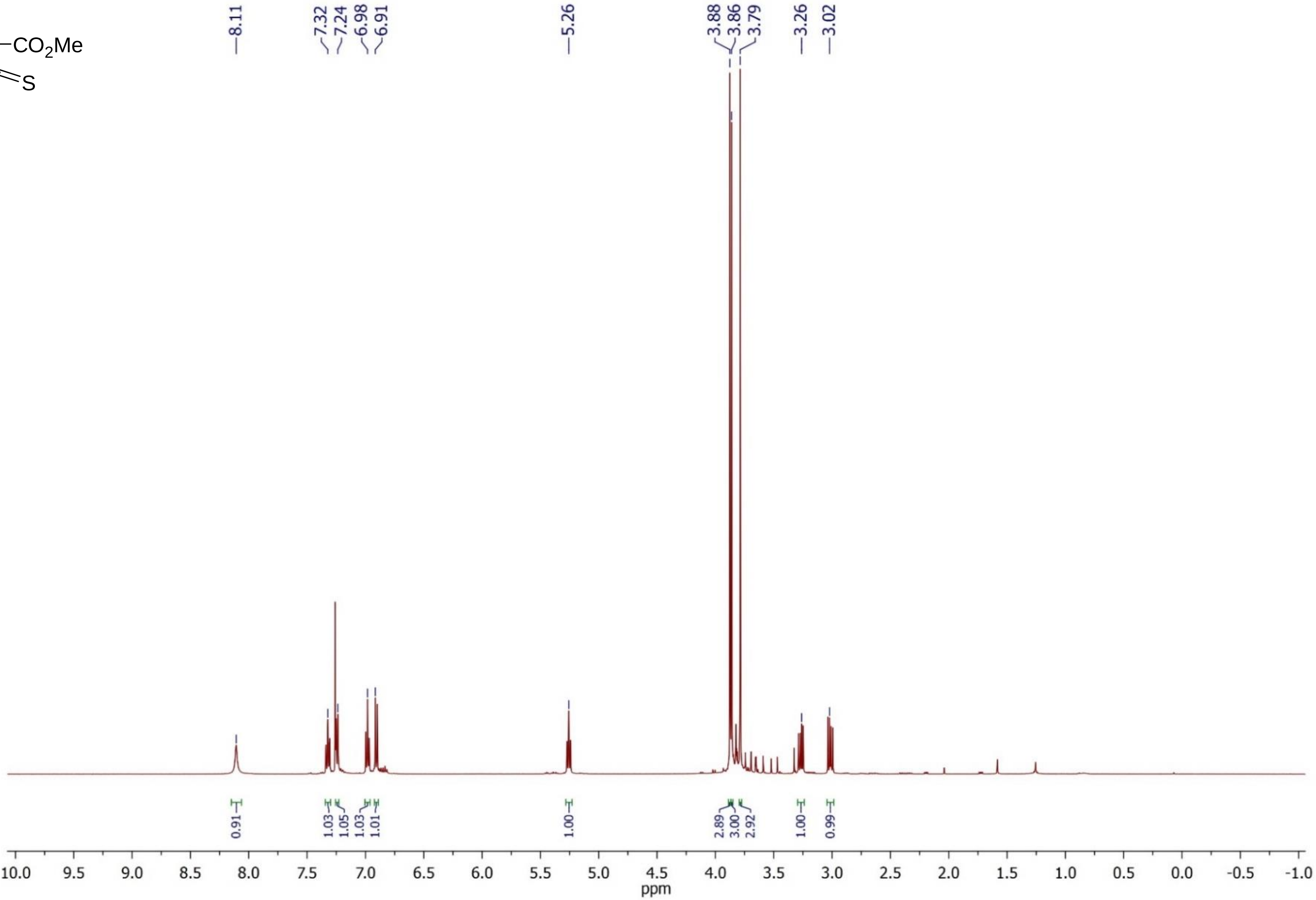

Dimethyl 5-(2-methoxyphenyl)-2-thioxopyrrolidine-3,3-dicarboxylate (2h)

^{1}H NMR (500 MHz, DMSO-d$_6$)

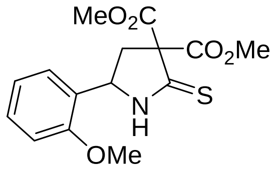

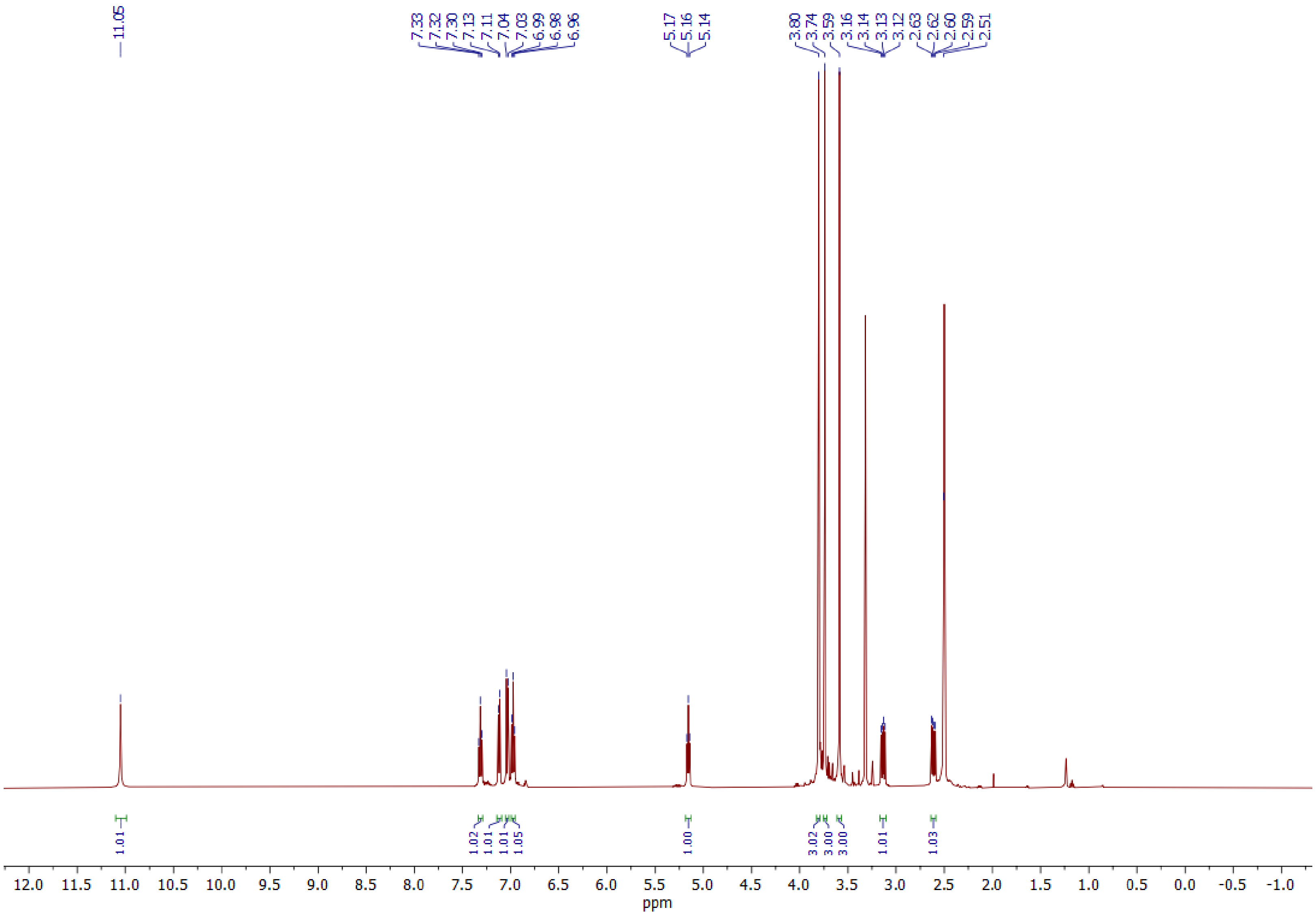

Dimethyl 5-(2-methoxyphenyl)-2-thioxopyrrolidine-3,3-dicarboxylate (2h)

^{13}C NMR (126 MHz, $CDCl_3$/DMSO-d_6, 2:1)

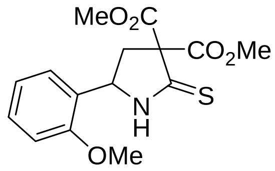

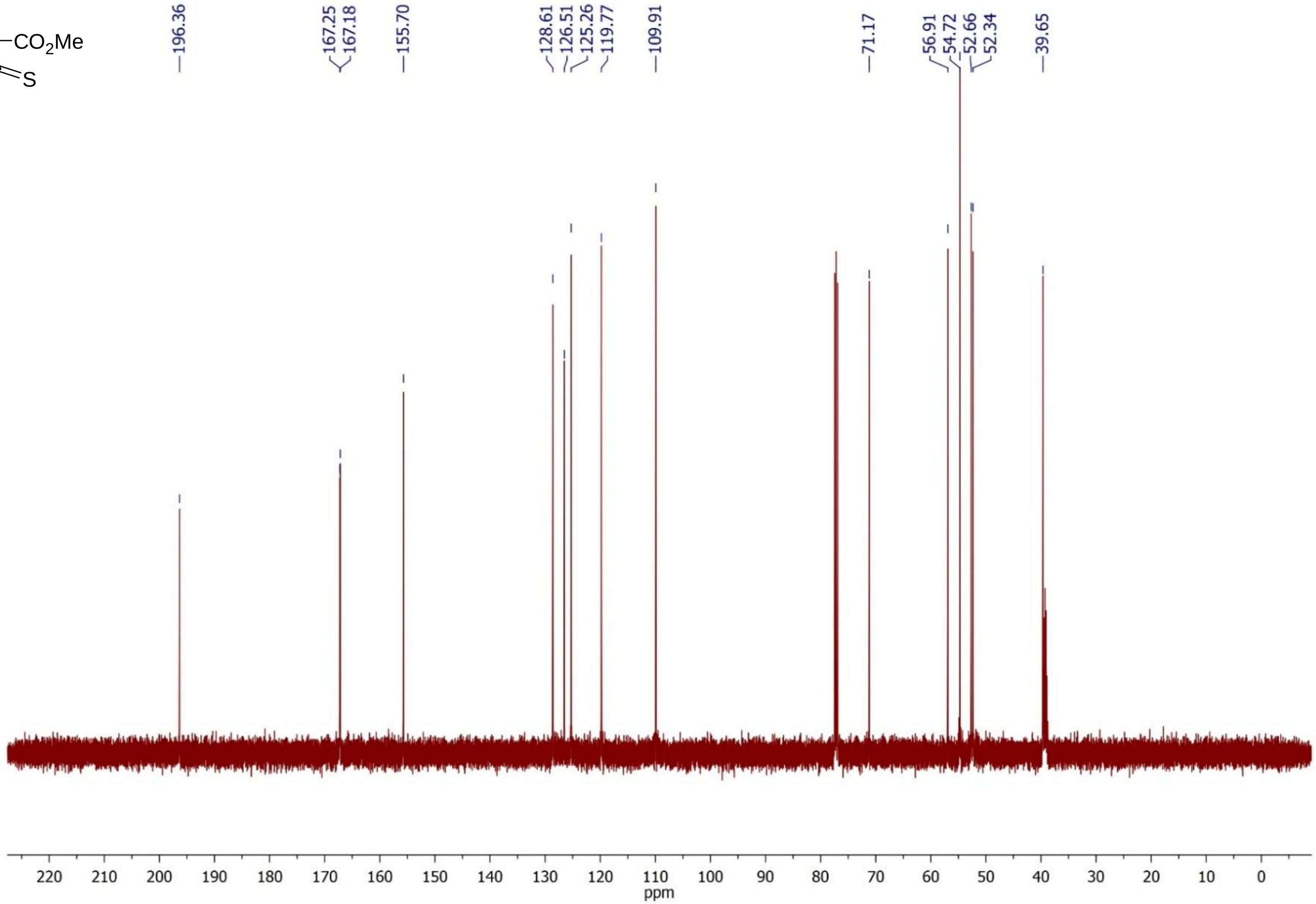

Dimethyl 5-(2-methoxyphenyl)-2-thioxopyrrolidine-3,3-dicarboxylate (2h)

^{13}C NMR (126 MHz, DMSO-d_6)

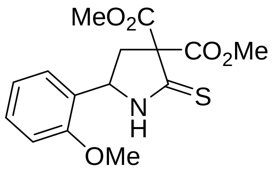

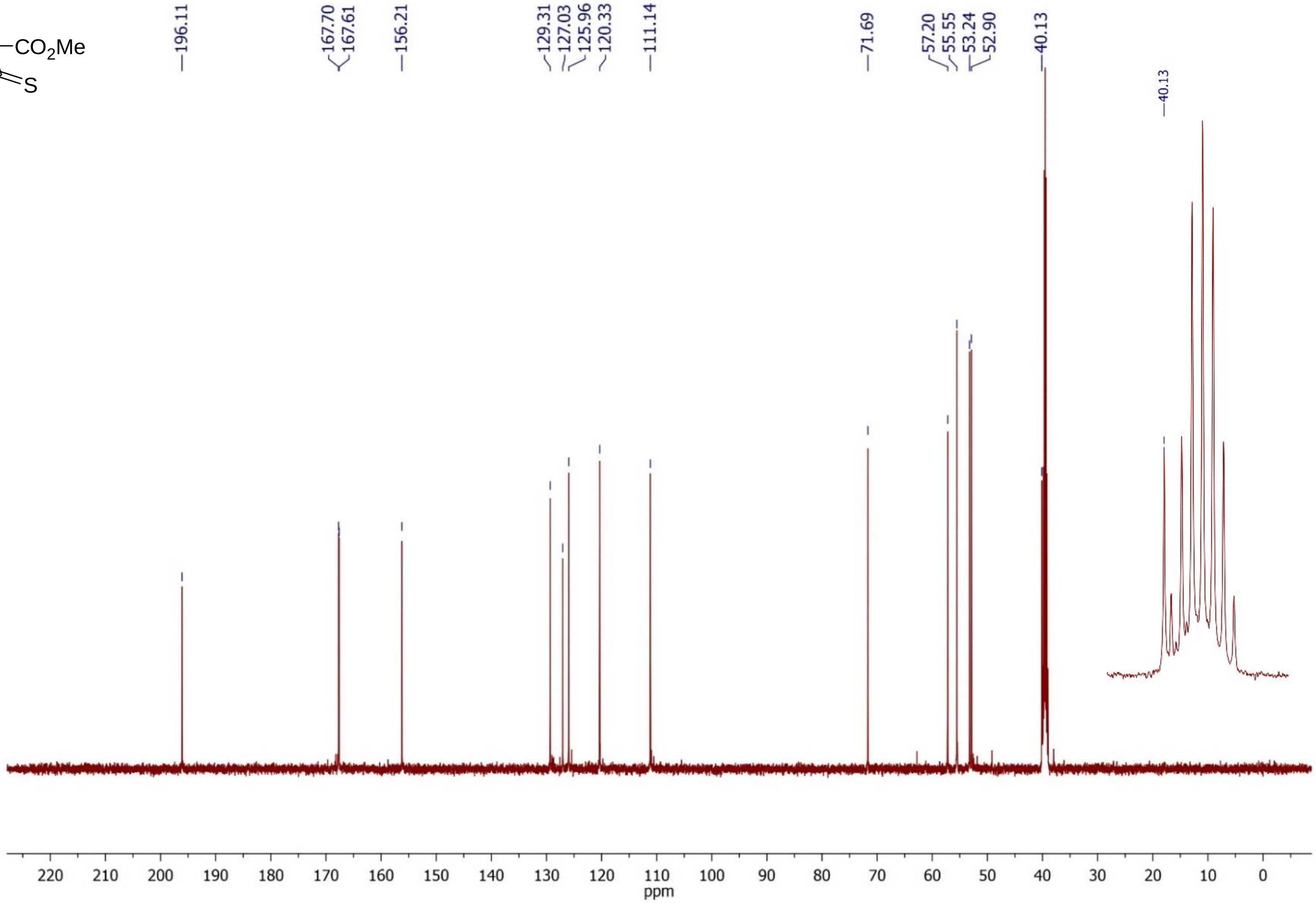

Dimethyl 5-(2-methoxyphenyl)-2-thioxopyrrolidine-3,3-dicarboxylate (2h)

^{1}H–^{13}C HSQC (DMSO-d_6)

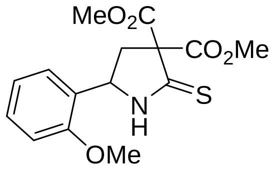

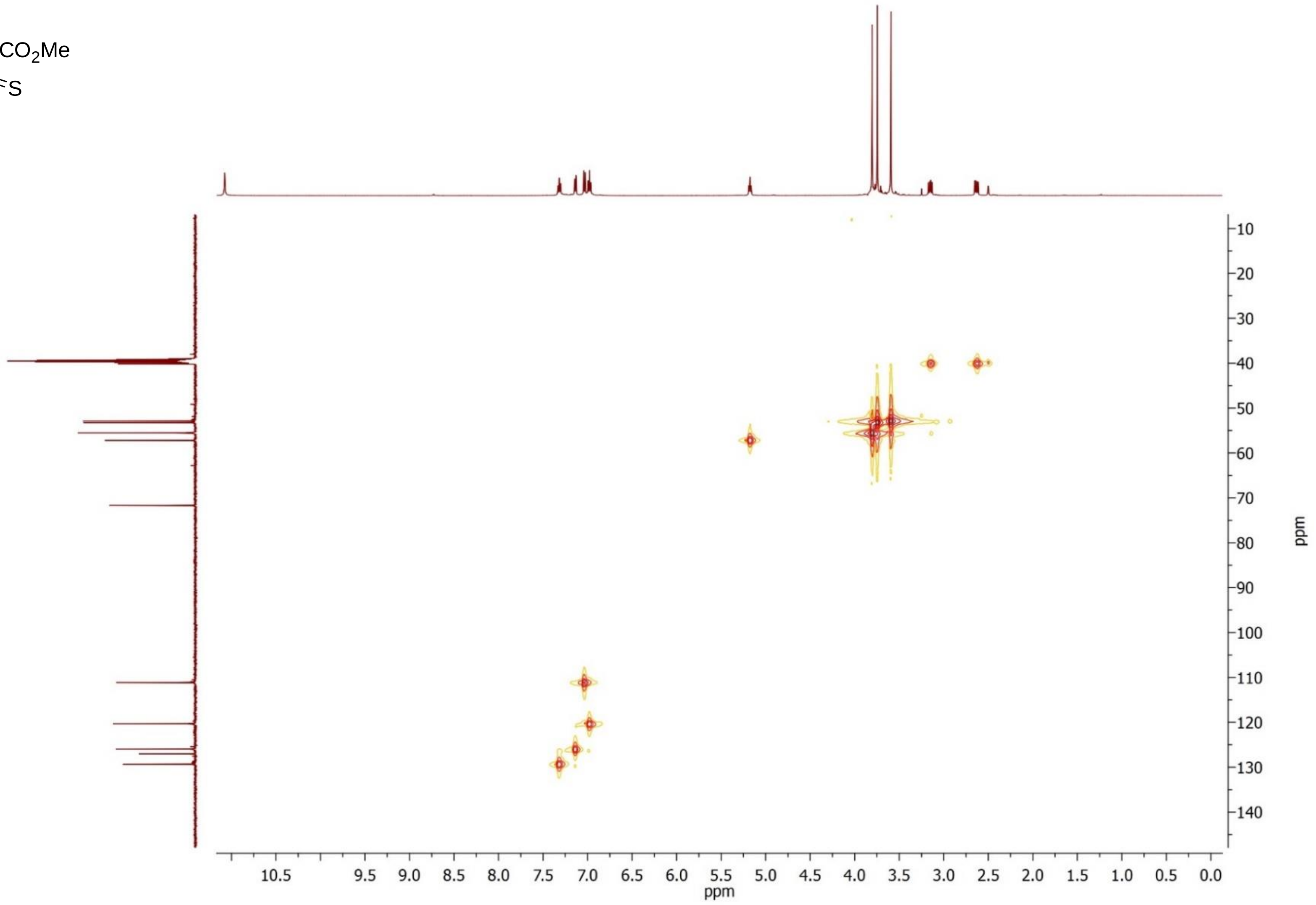

Dimethyl 5-[2-(ethoxymethoxy)phenyl]-2-thioxopyrrolidine-3,3-dicarboxylate (2i)

^{1}H NMR (500 MHz, $CDCl_3$)

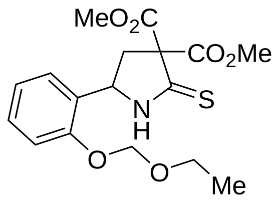

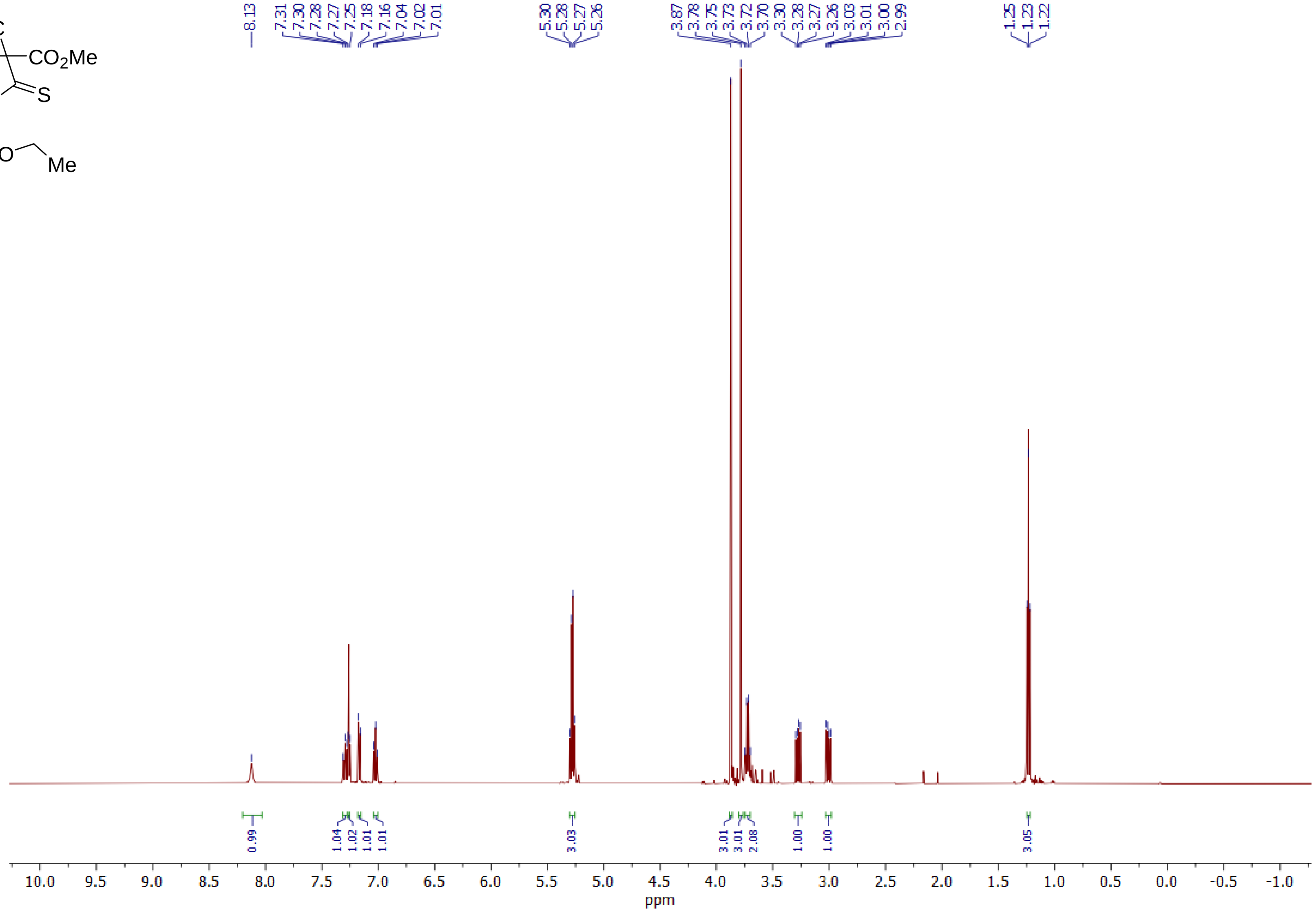

Dimethyl 5-[2-(ethoxymethoxy)phenyl]-2-thioxopyrrolidine-3,3-dicarboxylate (2i)

^{13}C NMR (126 MHz, $CDCl_3$)

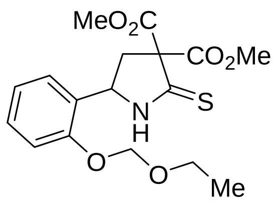

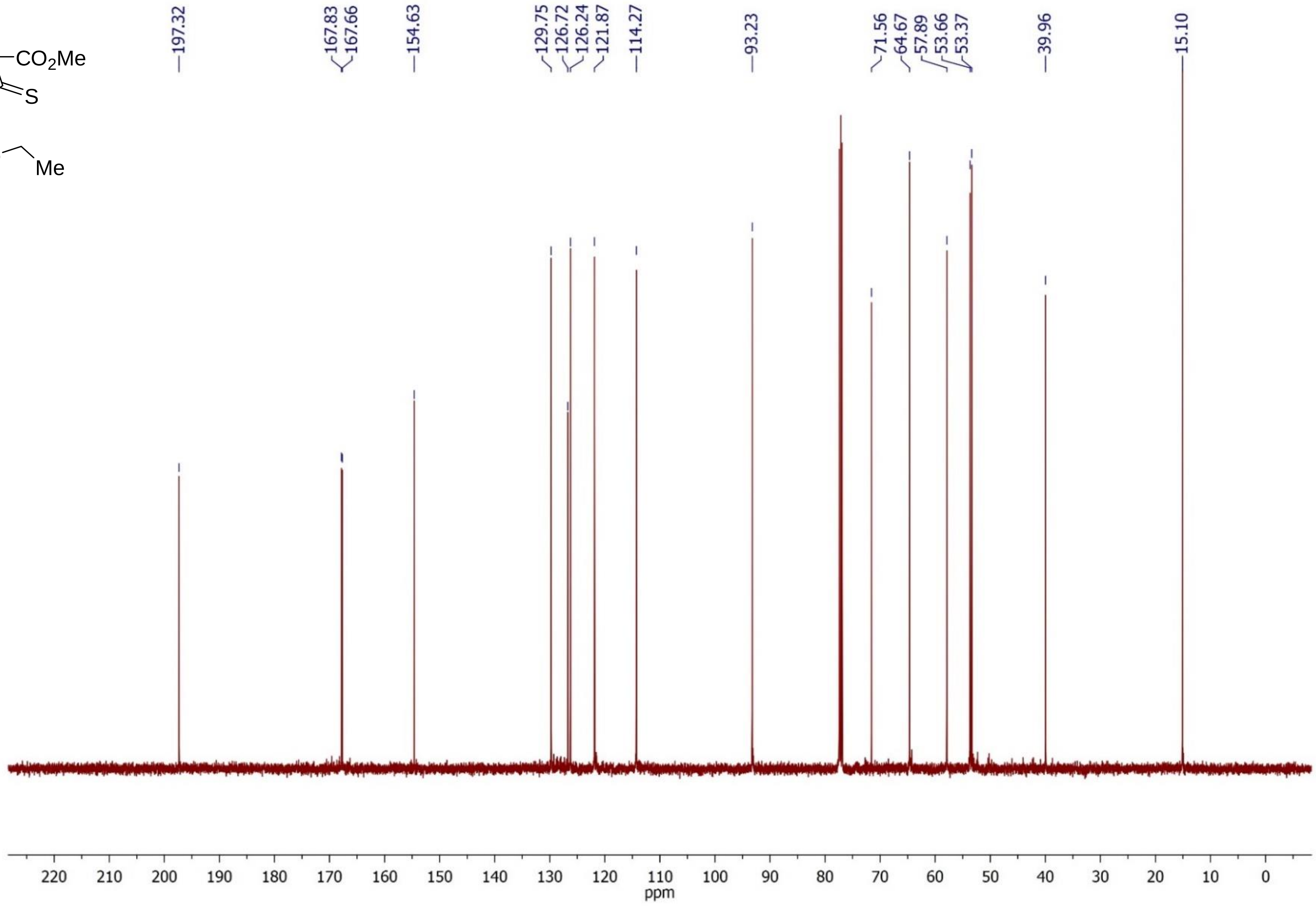

Dimethyl 5-[2-(ethoxymethoxy)phenyl]-2-thioxopyrrolidine-3,3-dicarboxylate (2i)

^1H–^{13}C HSQC ($CDCl_3$)

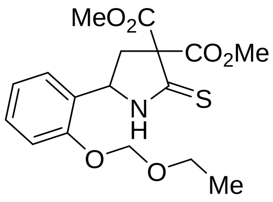

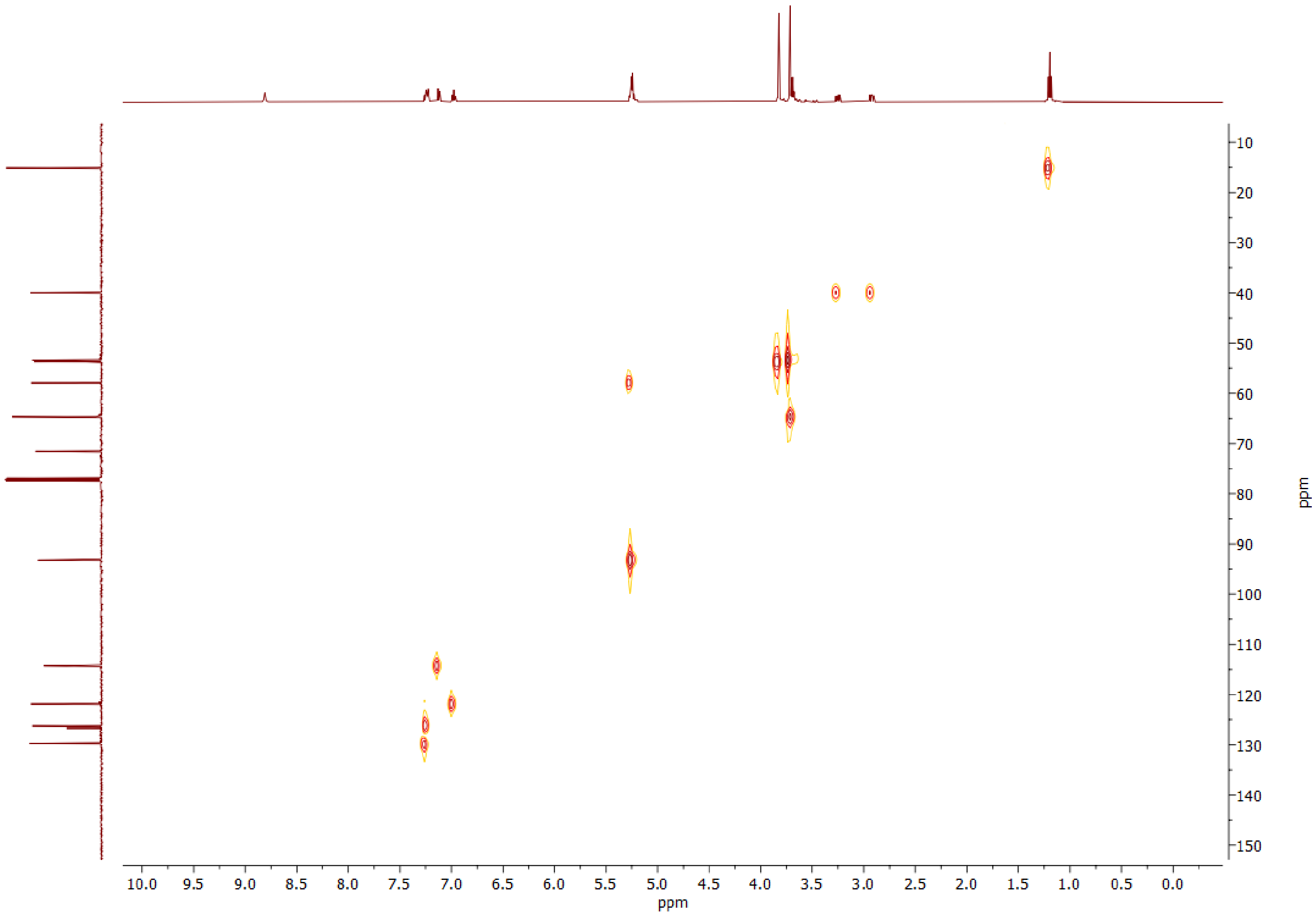

Dimethyl 5-(2-hydroxyphenyl)-2-thioxopyrrolidine-3,3-dicarboxylate (2j)

^{1}H NMR (500 MHz, DMSO-d_6)

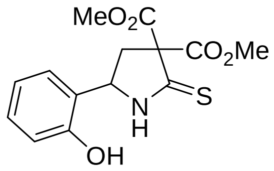

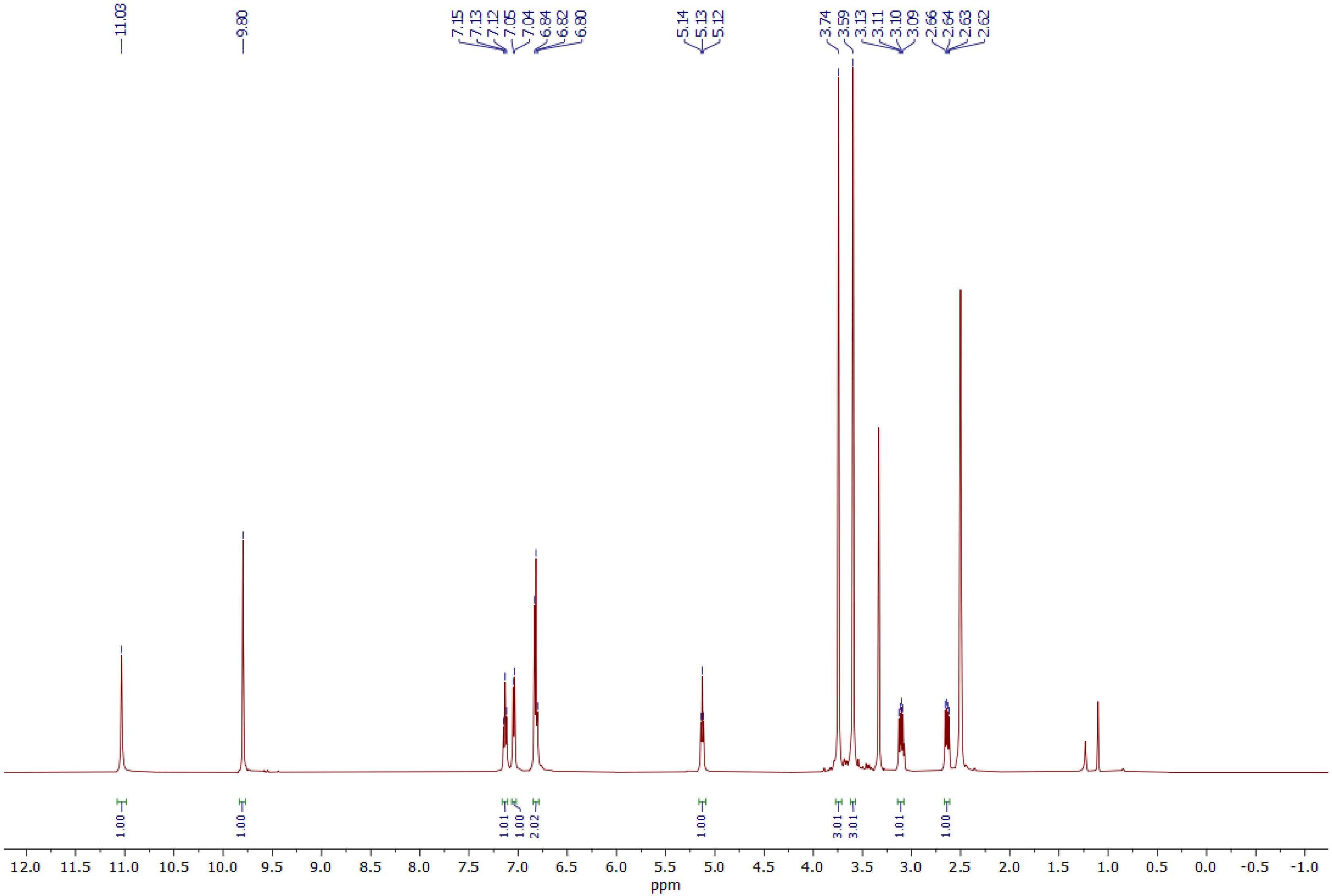

Dimethyl 5-(2-hydroxyphenyl)-2-thioxopyrrolidine-3,3-dicarboxylate (2j)

^{13}C NMR (126 MHz, DMSO-d_6)

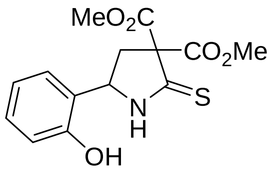

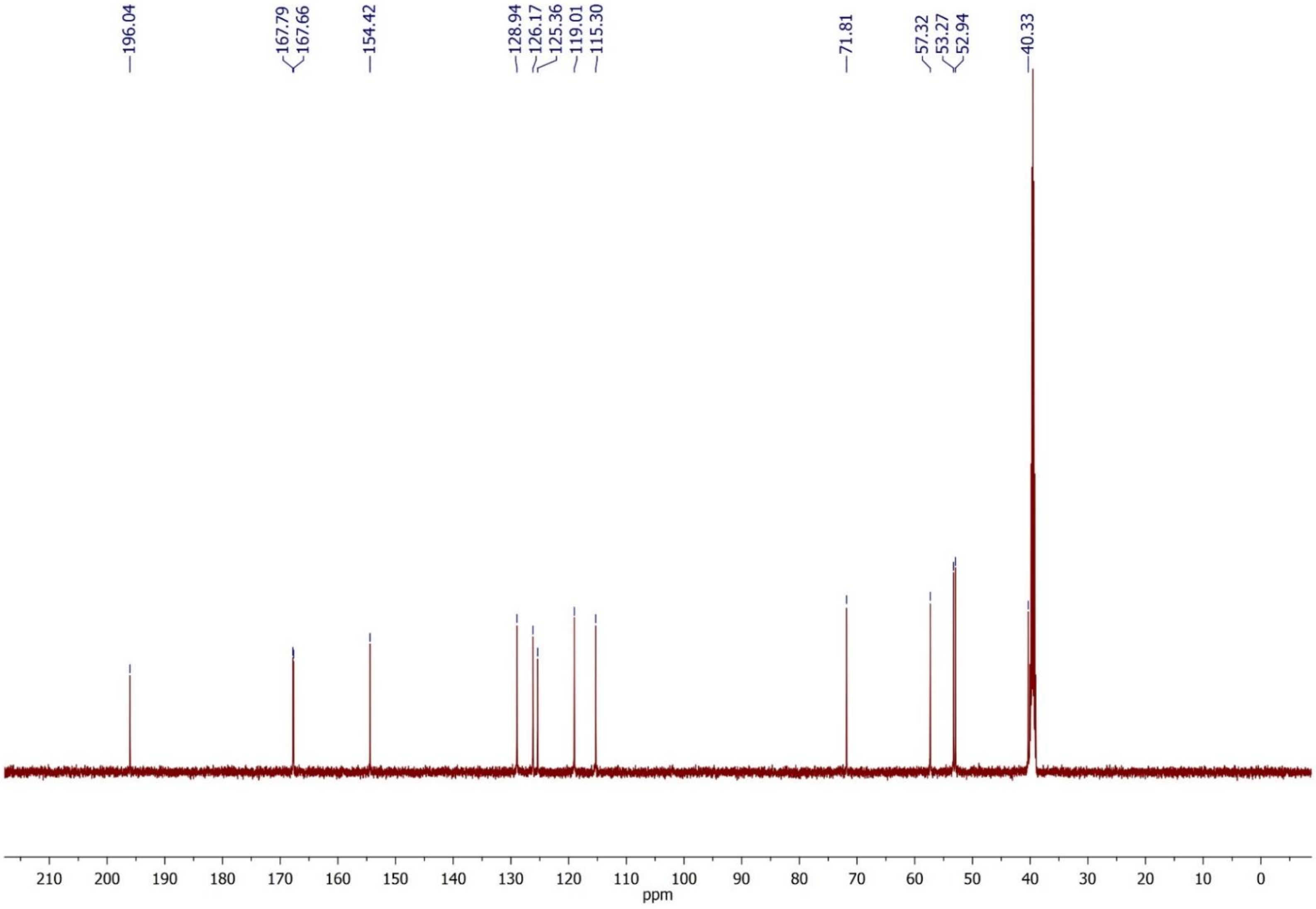

Dimethyl 5-(2,6-dimethoxyphenyl)-2-thioxopyrrolidine-3,3-dicarboxylate (2k)

^{1}H NMR (500 MHz, $CDCl_3$)

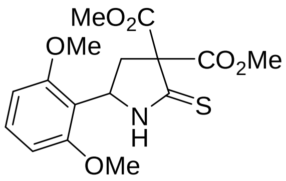

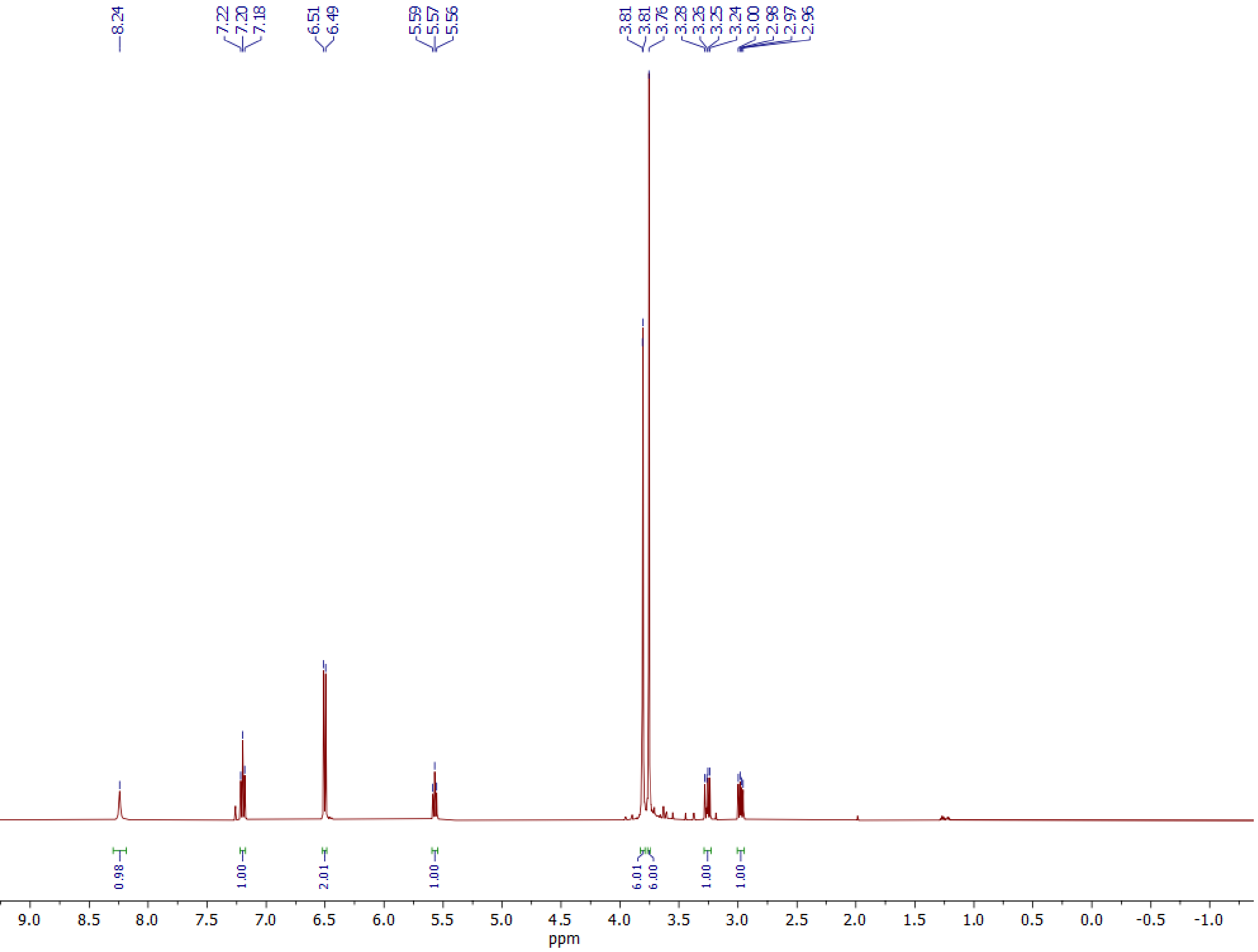

Dimethyl 5-(2,6-dimethoxyphenyl)-2-thioxopyrrolidine-3,3-dicarboxylate (2k)

^{13}C NMR (126 MHz, $CDCl_3$)

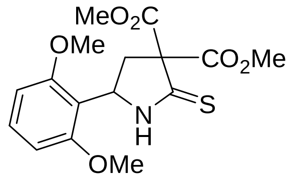

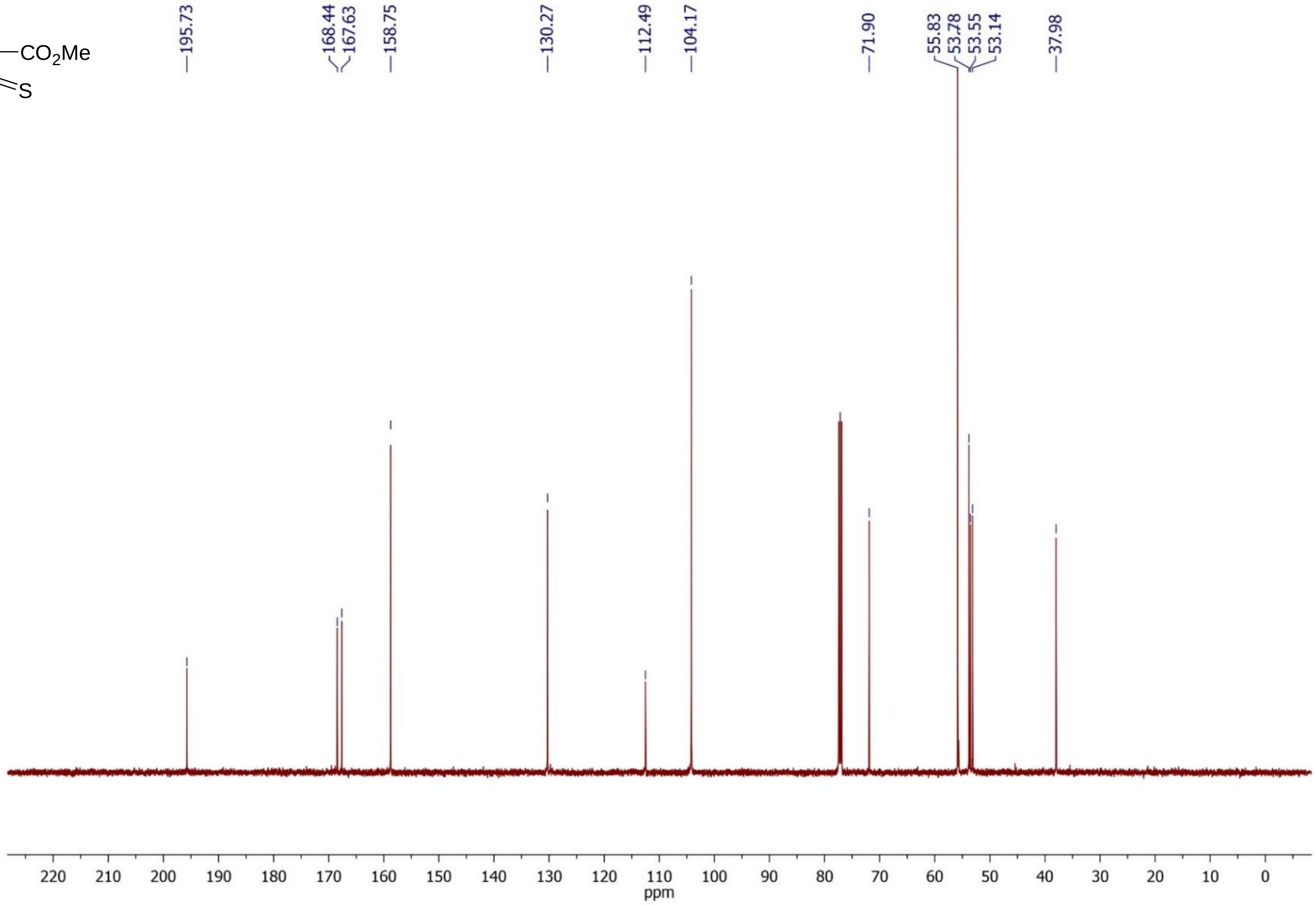

Dimethyl 5-(2,6-dimethoxyphenyl)-2-thioxopyrrolidine-3,3-dicarboxylate (2k)

^{1}H–^{13}C HSQC ($CDCl_3$)

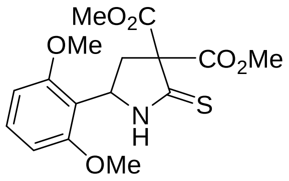

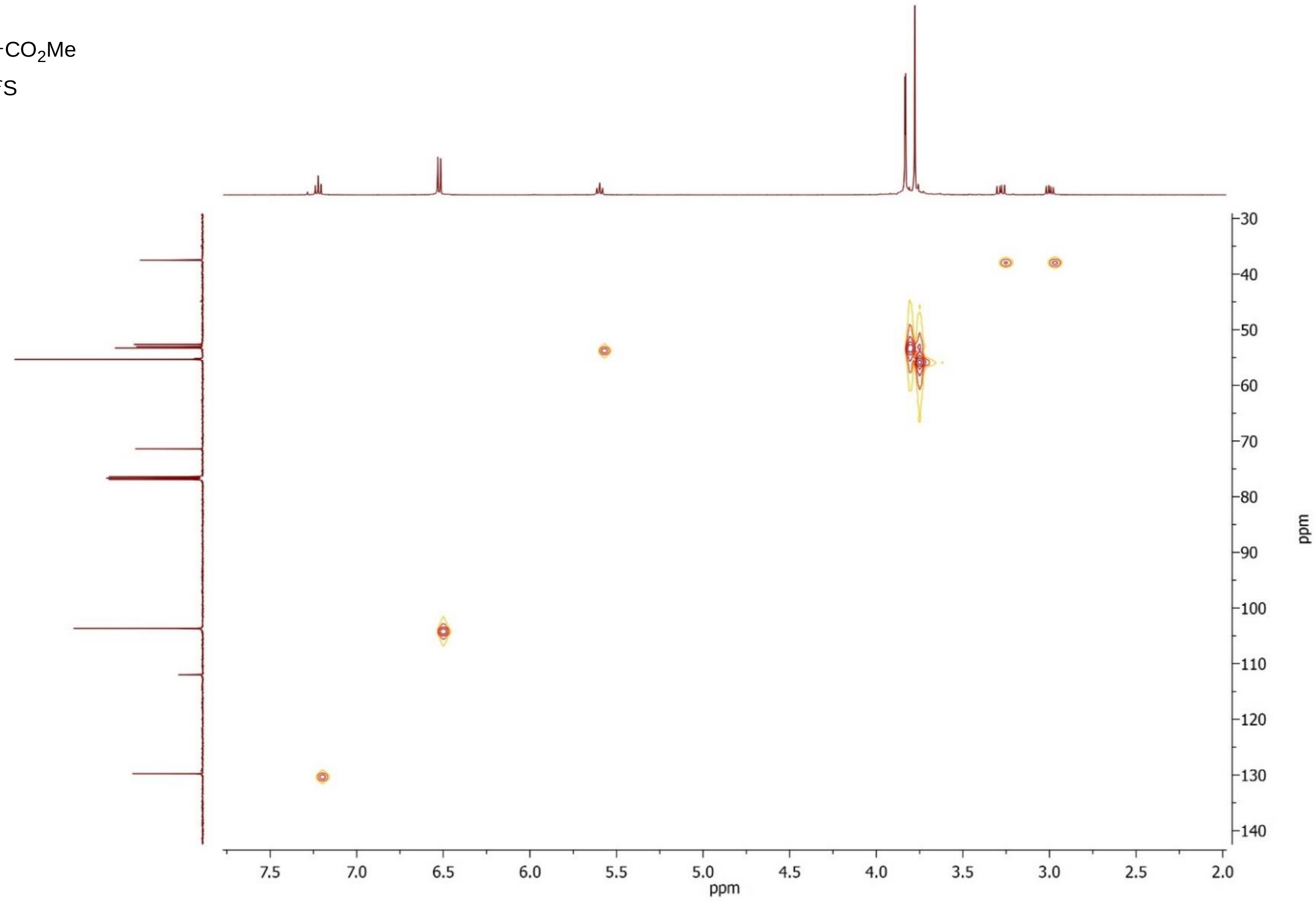

Dimethyl 2-thioxo-5-(2,4,6-trimethoxyphenyl)pyrrolidine-3,3-dicarboxylate (2l)

^{1}H NMR (500 MHz, $CDCl_3$)

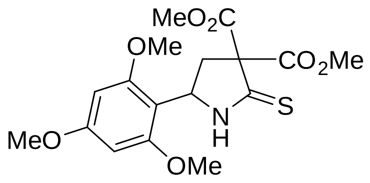

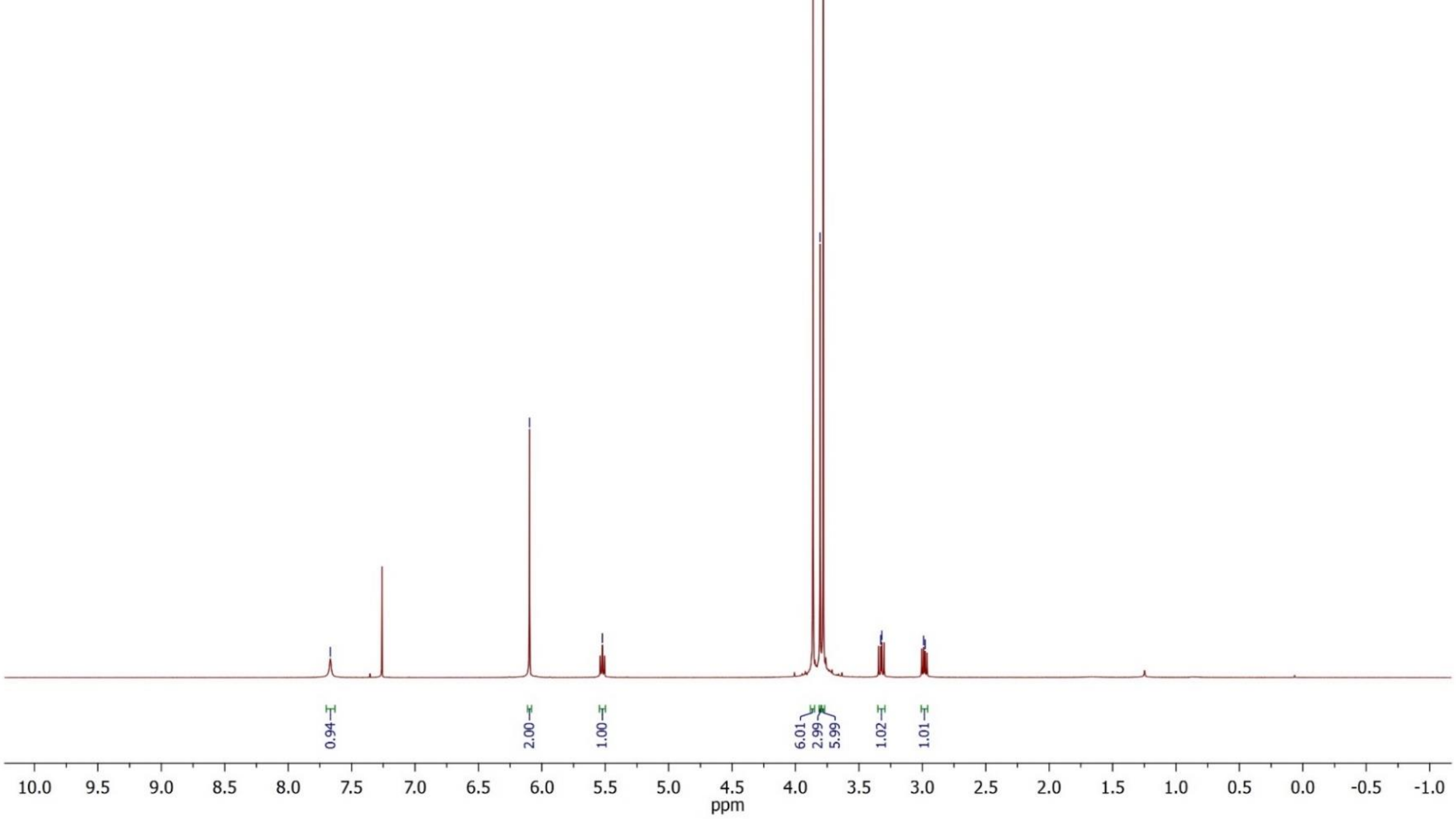

Dimethyl 2-thioxo-5-(2,4,6-trimethoxyphenyl)pyrrolidine-3,3-dicarboxylate (2l)
^{13}C NMR (126 MHz, $CDCl_3$)

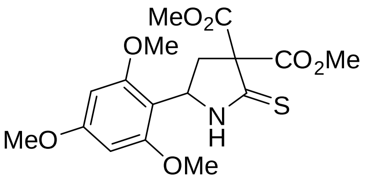

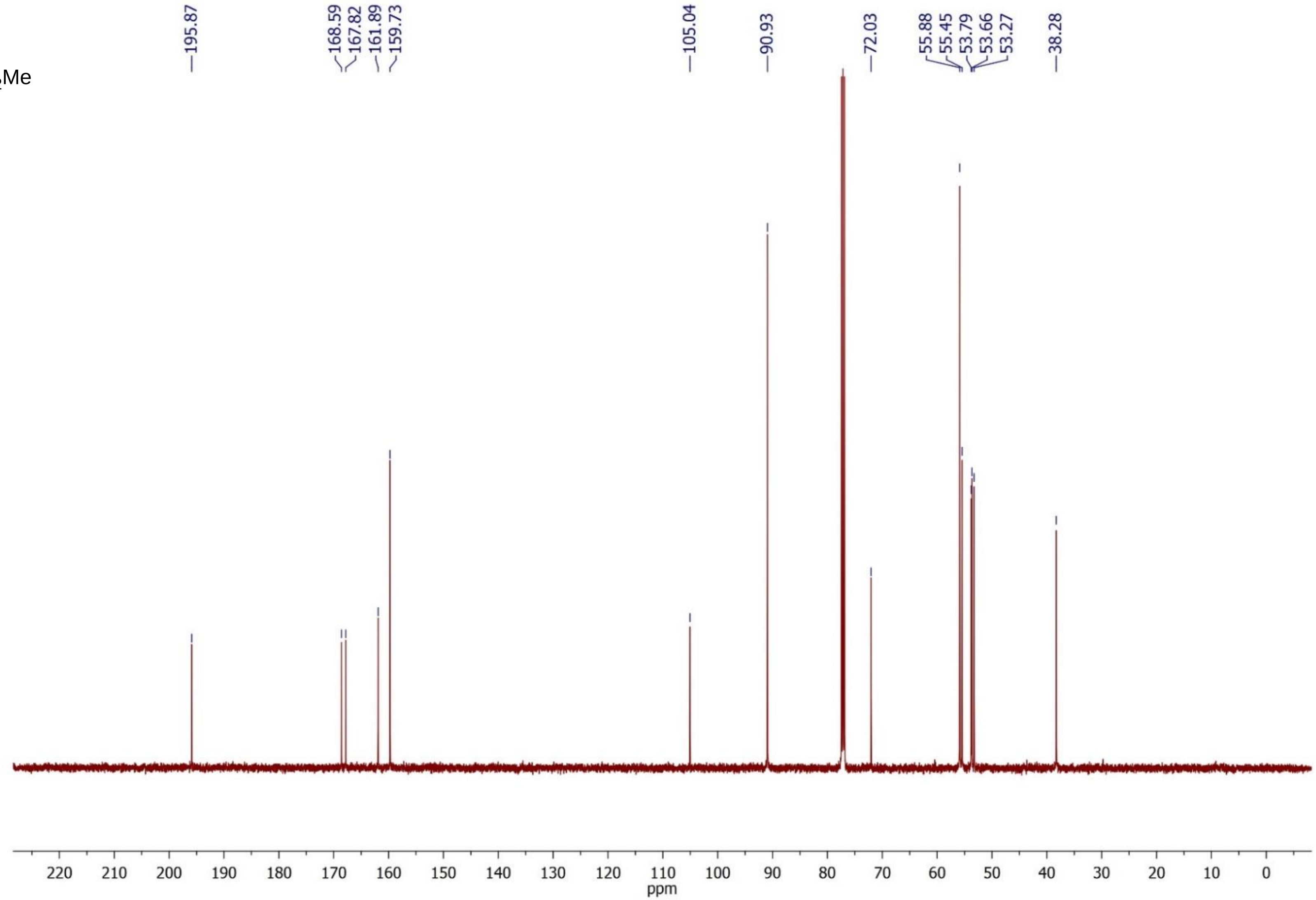

Dimethyl 2-thioxo-5-(2,4,6-trimethoxyphenyl)pyrrolidine-3,3-dicarboxylate (2l)

^{1}H–^{13}C HSQC ($CDCl_3$)

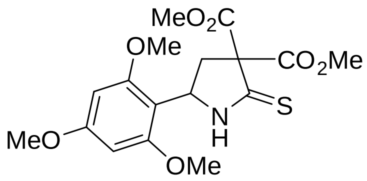

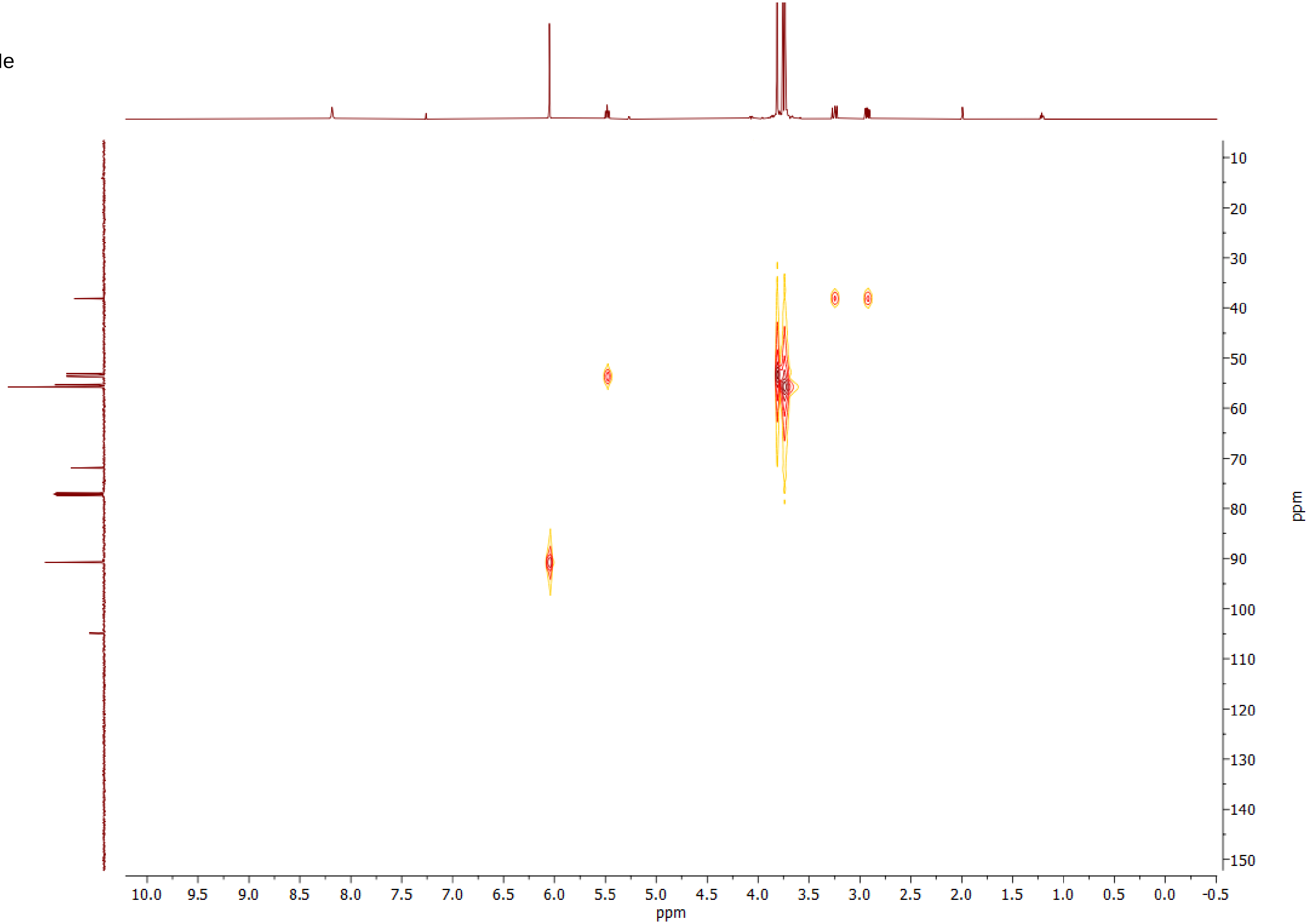

Dimethyl 5-[4-(dimethylamino)phenyl]-2-thioxopyrrolidine-3,3-dicarboxylate (2m)

^{1}H NMR (500 MHz, $CDCl_3$)

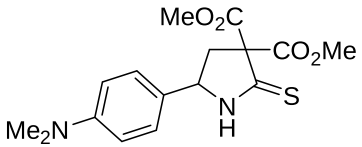

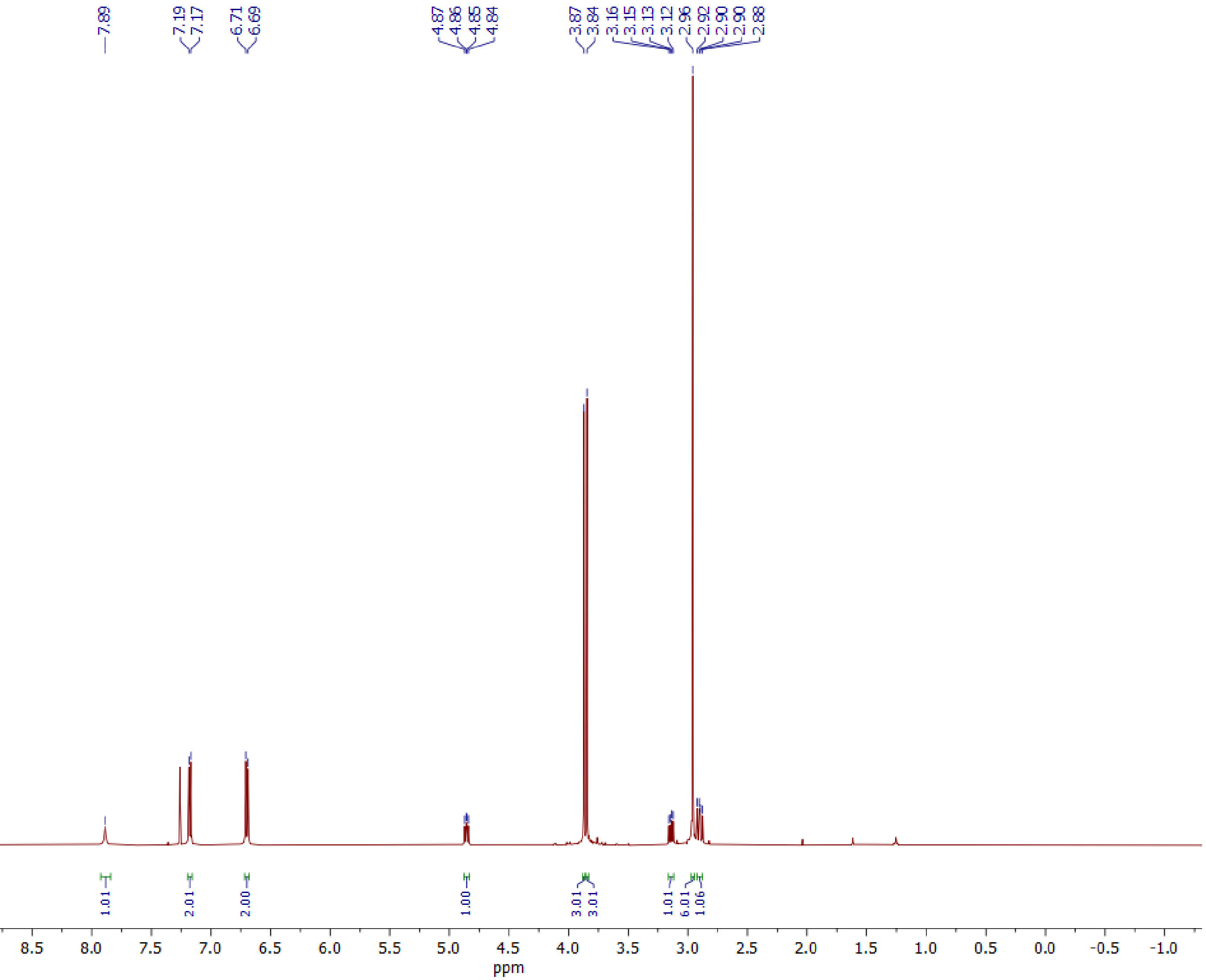

Dimethyl 5-[4-(dimethylamino)phenyl]-2-thioxopyrrolidine-3,3-dicarboxylate (2m)

^{13}C NMR (126 MHz, $CDCl_3$)

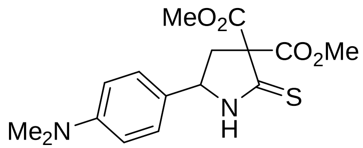

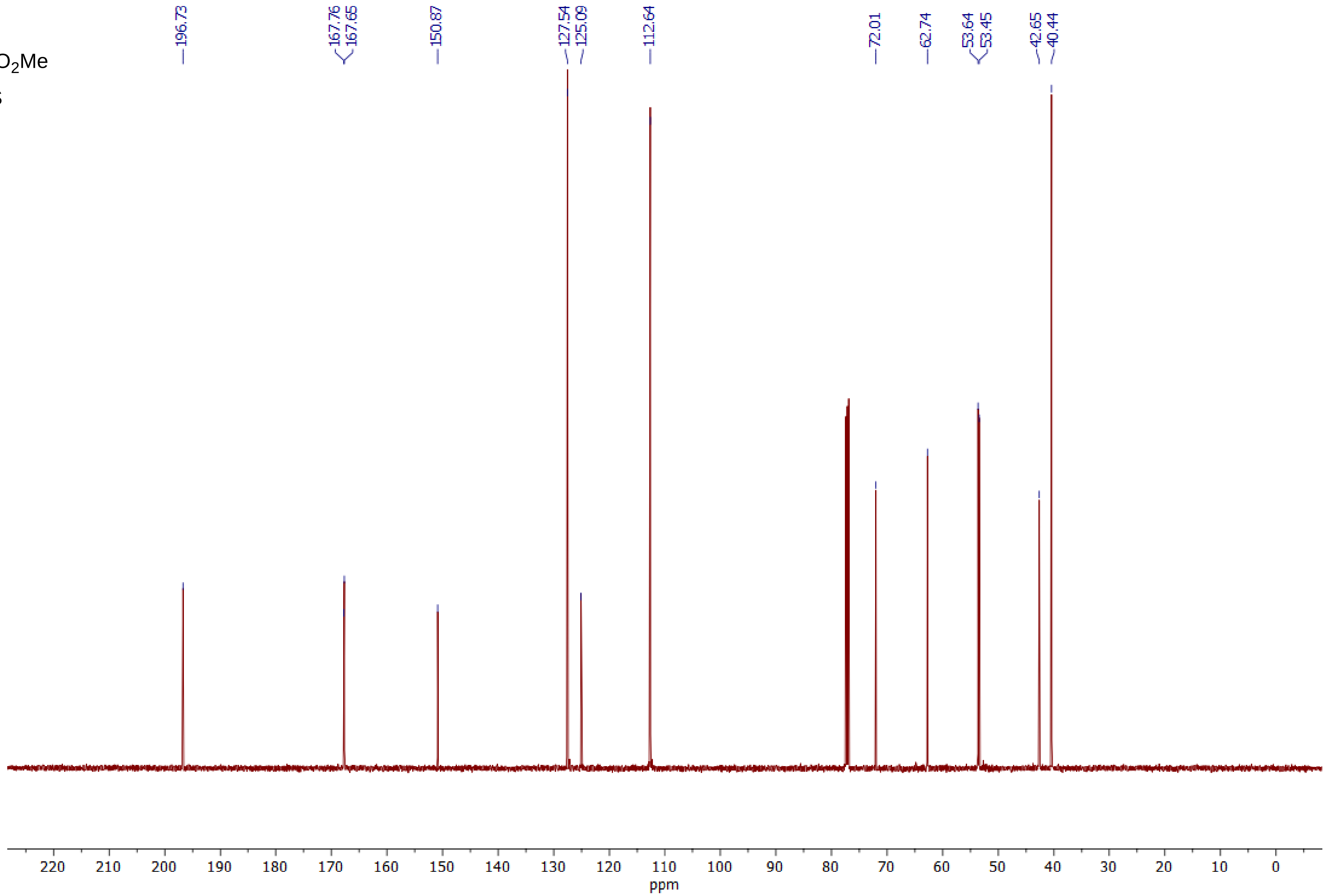

Dimethyl 5-(4-dimethylamino-2-nitrophenyl)-2-thioxopyrrolidine-3,3-dicarboxylate (2n)

^{1}H NMR (500 MHz, $CDCl_3$)

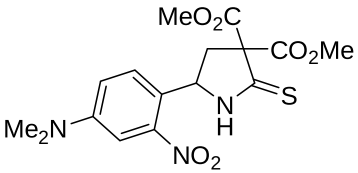

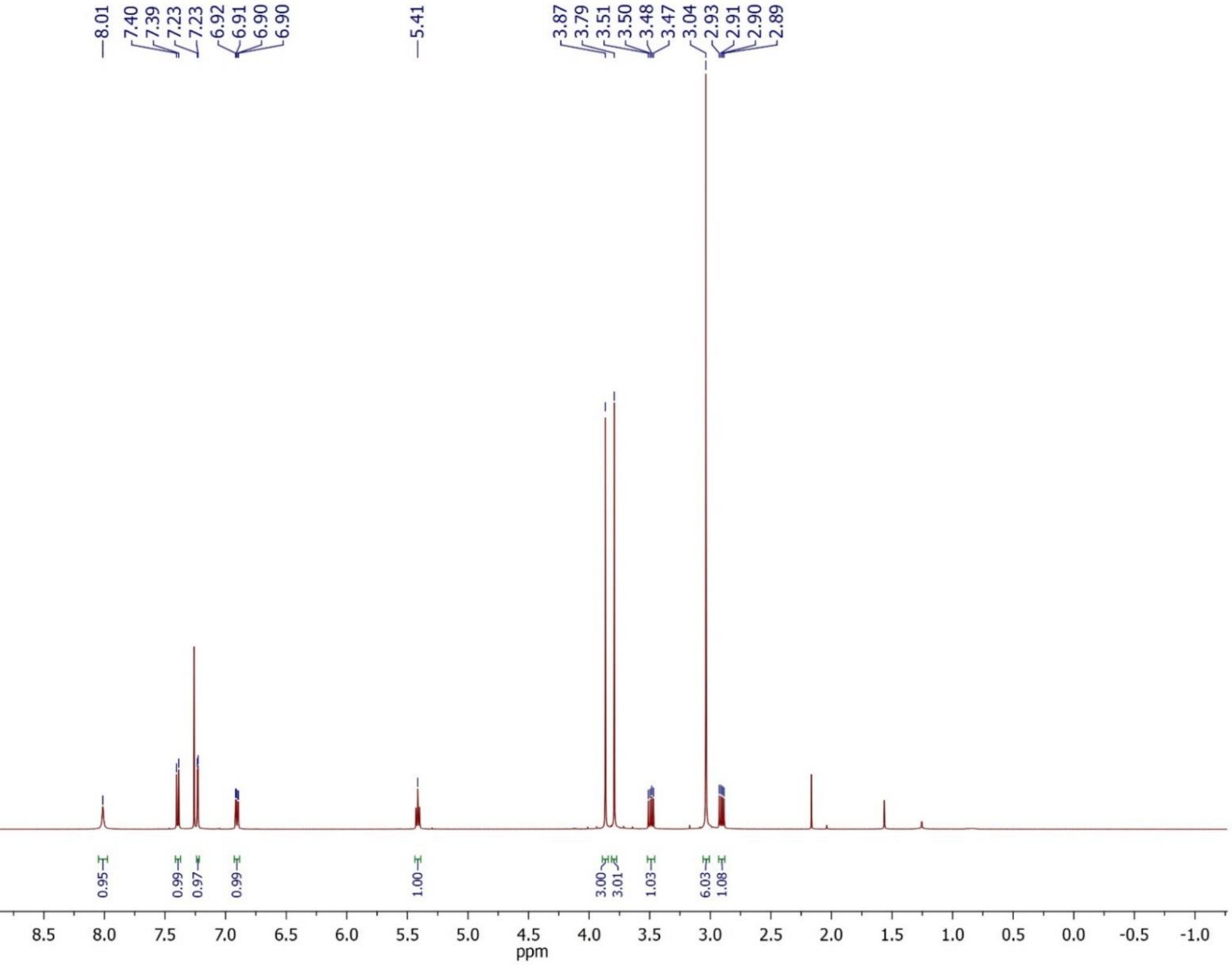

Dimethyl 5-(4-dimethylamino-2-nitrophenyl)-2-thioxopyrrolidine-3,3-dicarboxylate (2n)

^{1}H NMR (500 MHz, DMSO-d$_6$, 46 °C)

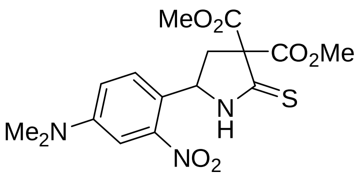

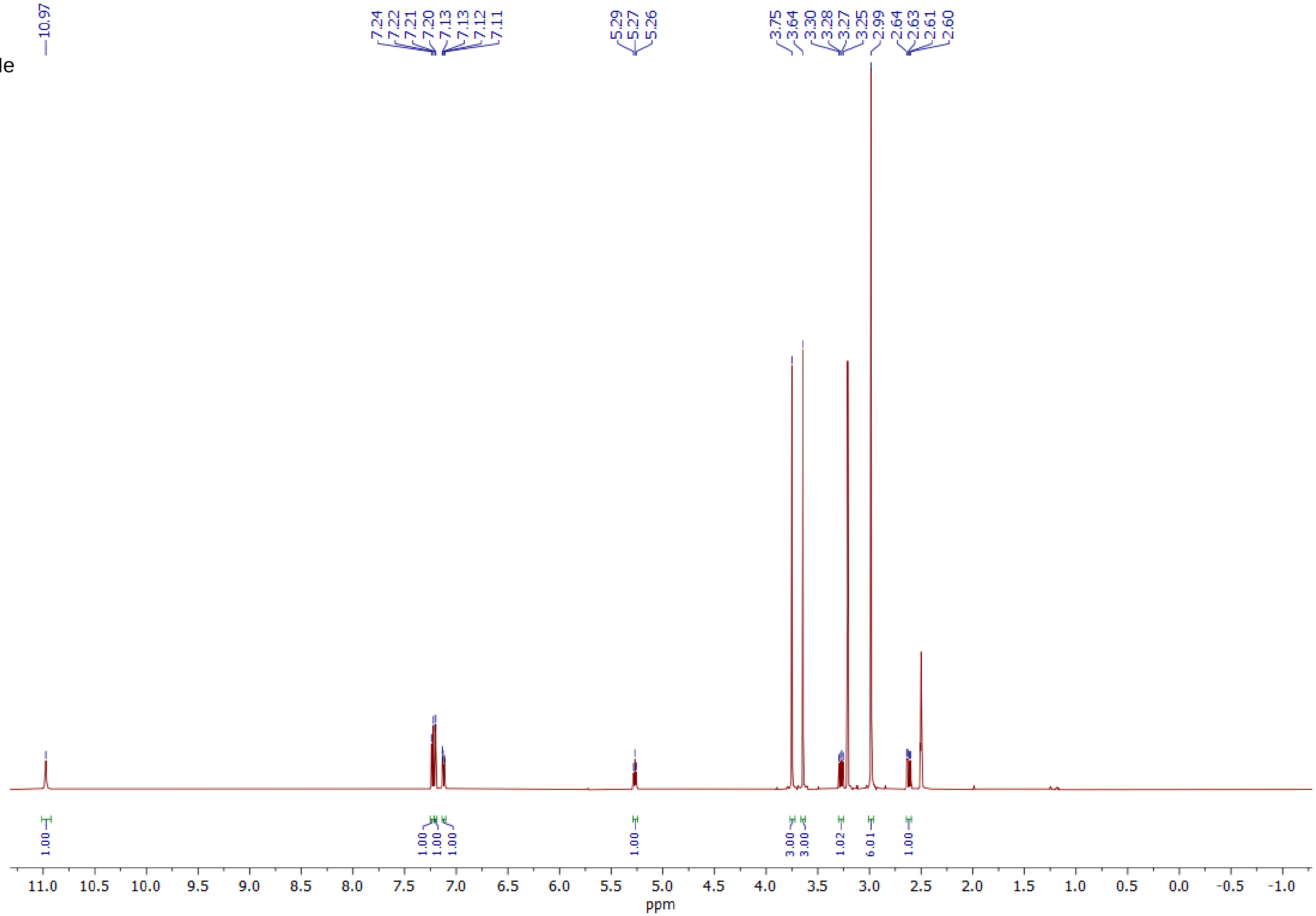

Dimethyl 5-(4-dimethylamino-2-nitrophenyl)-2-thioxopyrrolidine-3,3-dicarboxylate (2n)

^{13}C NMR (126 MHz, DMSO-d_6)

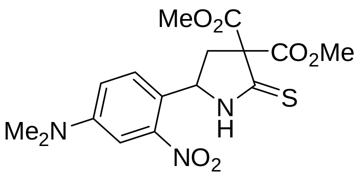

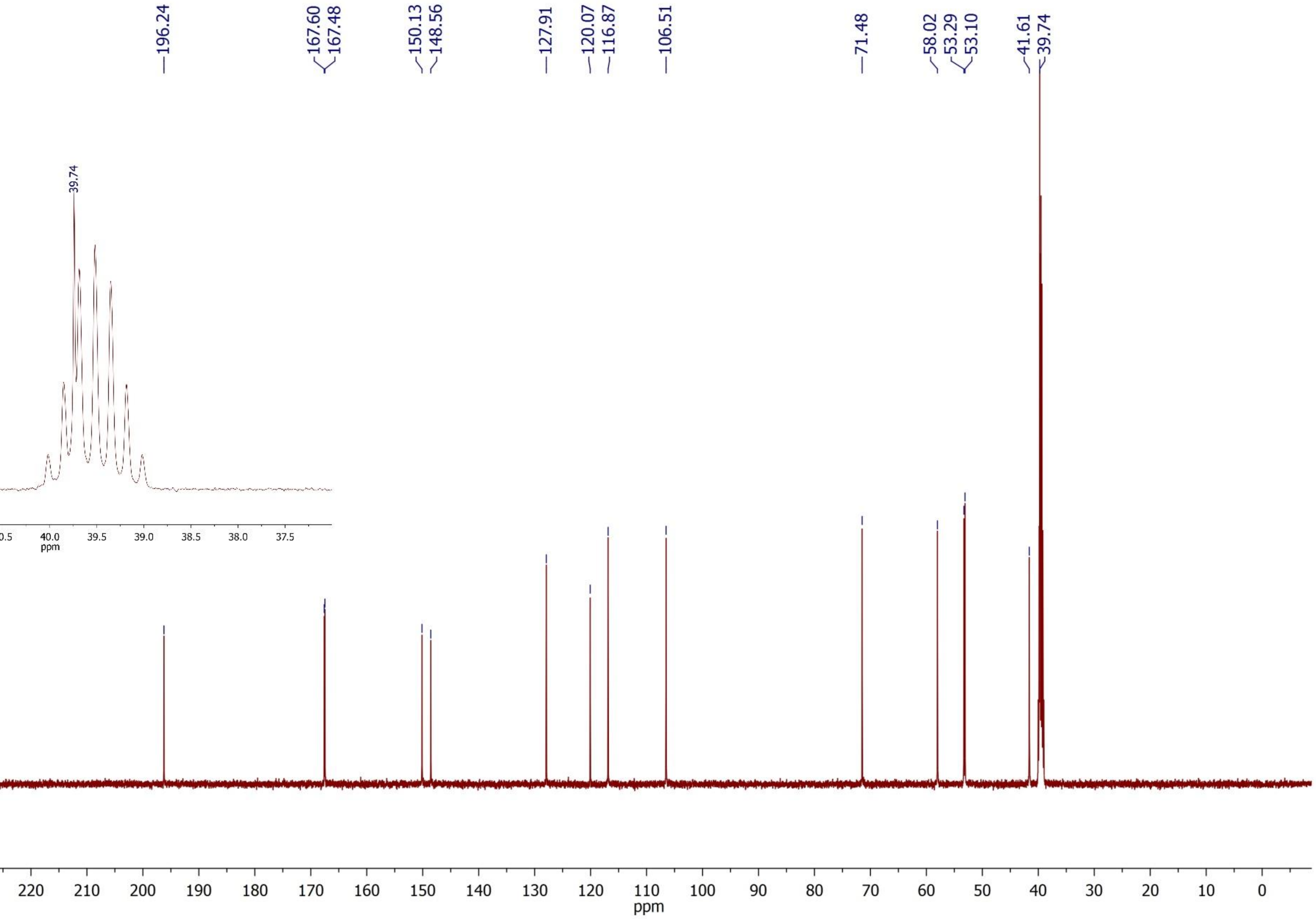

Dimethyl 5-(4-dimethylamino-2-nitrophenyl)-2-thioxopyrrolidine-3,3-dicarboxylate (2n)

^1H–^{13}C HSQC (DMSO-d$_6$)

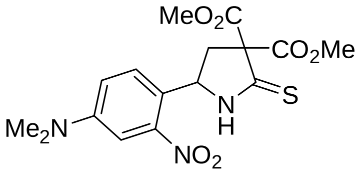

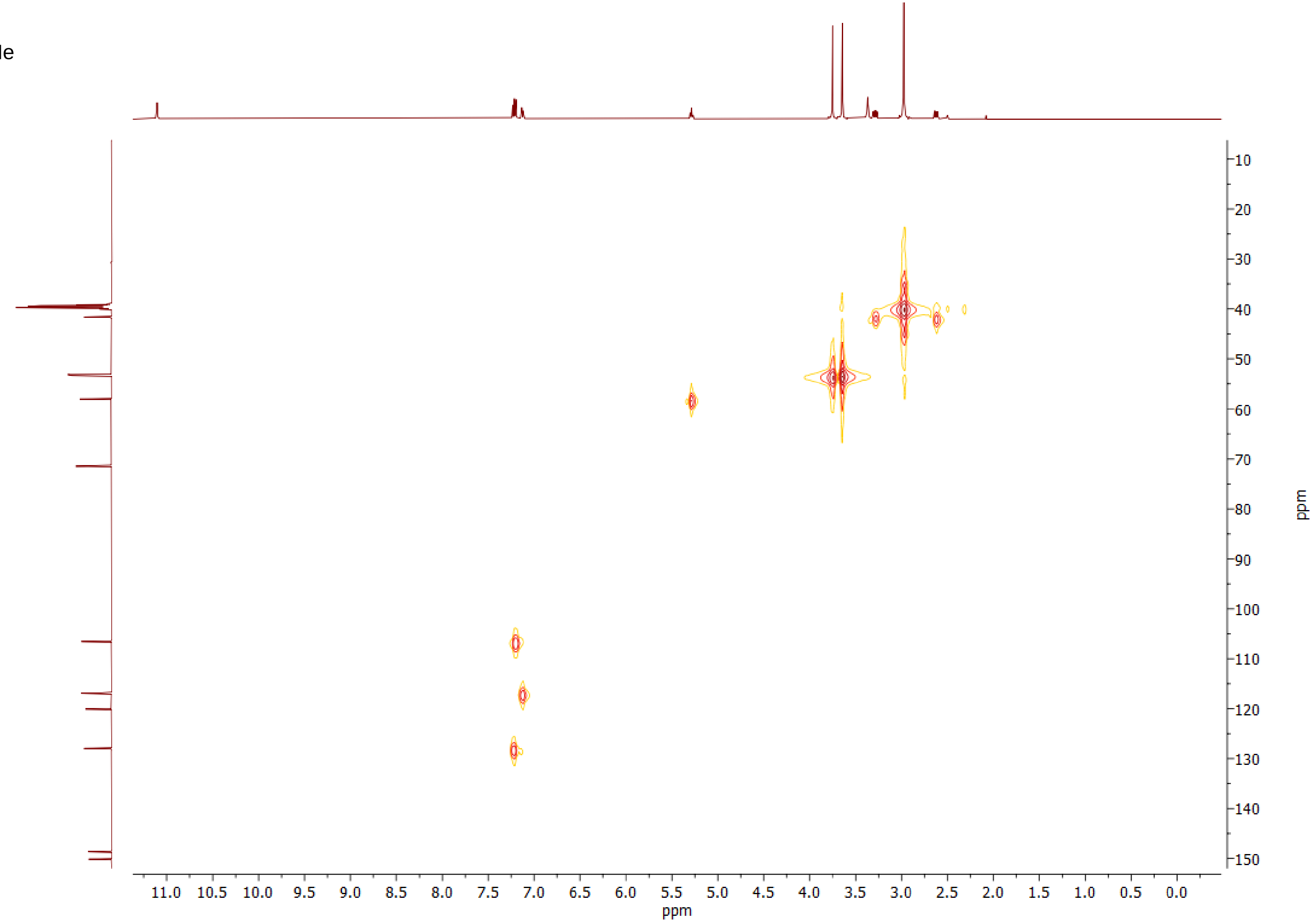

Dimethyl 5-[4′-methoxy-(1,1′-biphenyl)-4-yl]-2-thioxopyrrolidine-3,3-dicarboxylate (2o)
^{1}H NMR (500 MHz, $CDCl_3$)

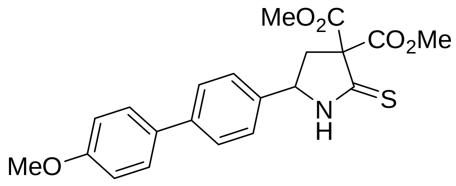

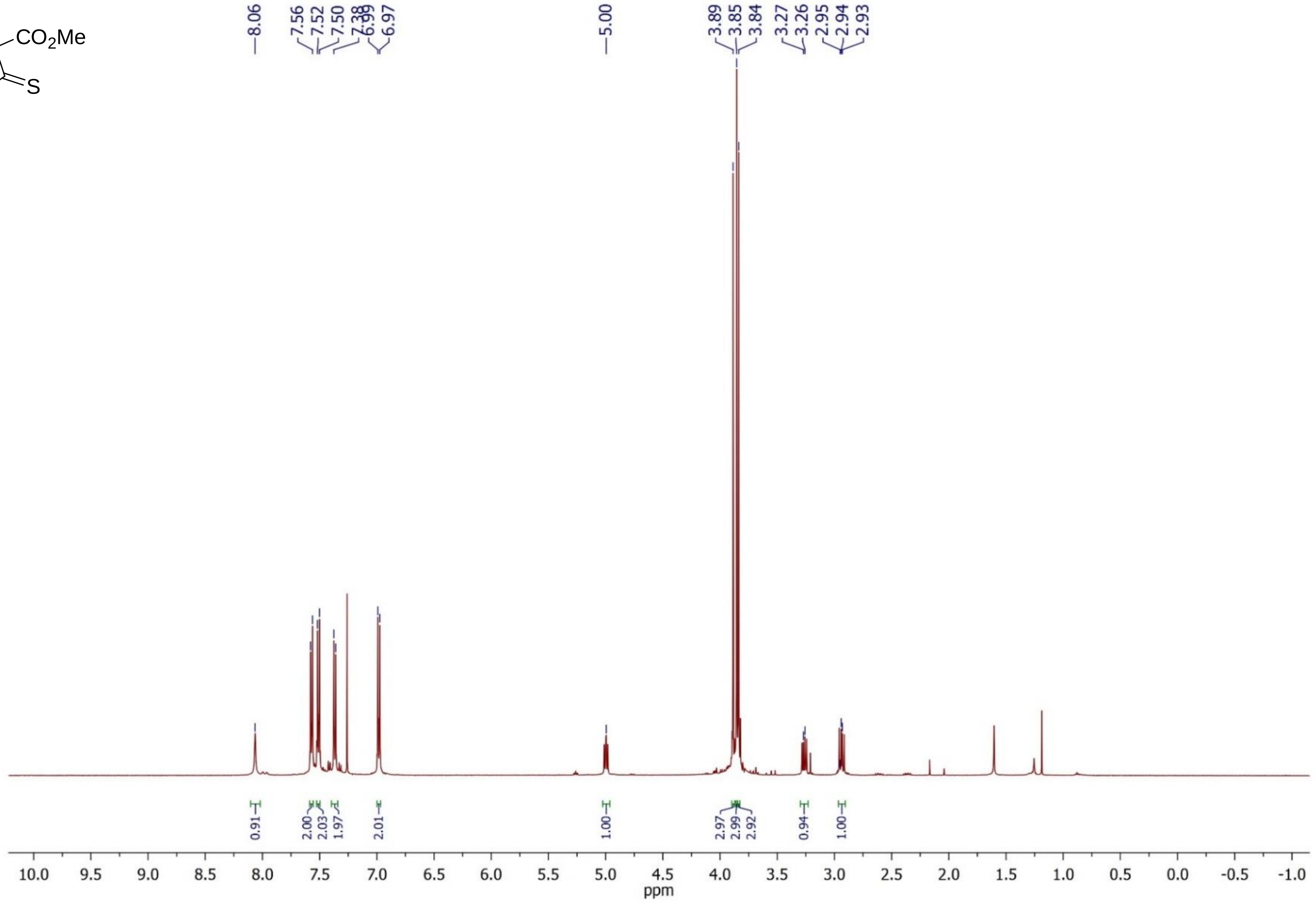

Dimethyl 5-[4′-methoxy-(1,1′-biphenyl)-4-yl]-2-thioxopyrrolidine-3,3-dicarboxylate (2o)

^{13}C NMR (126 MHz, $CDCl_3$)

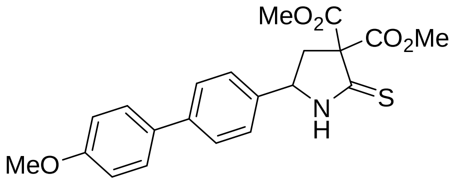

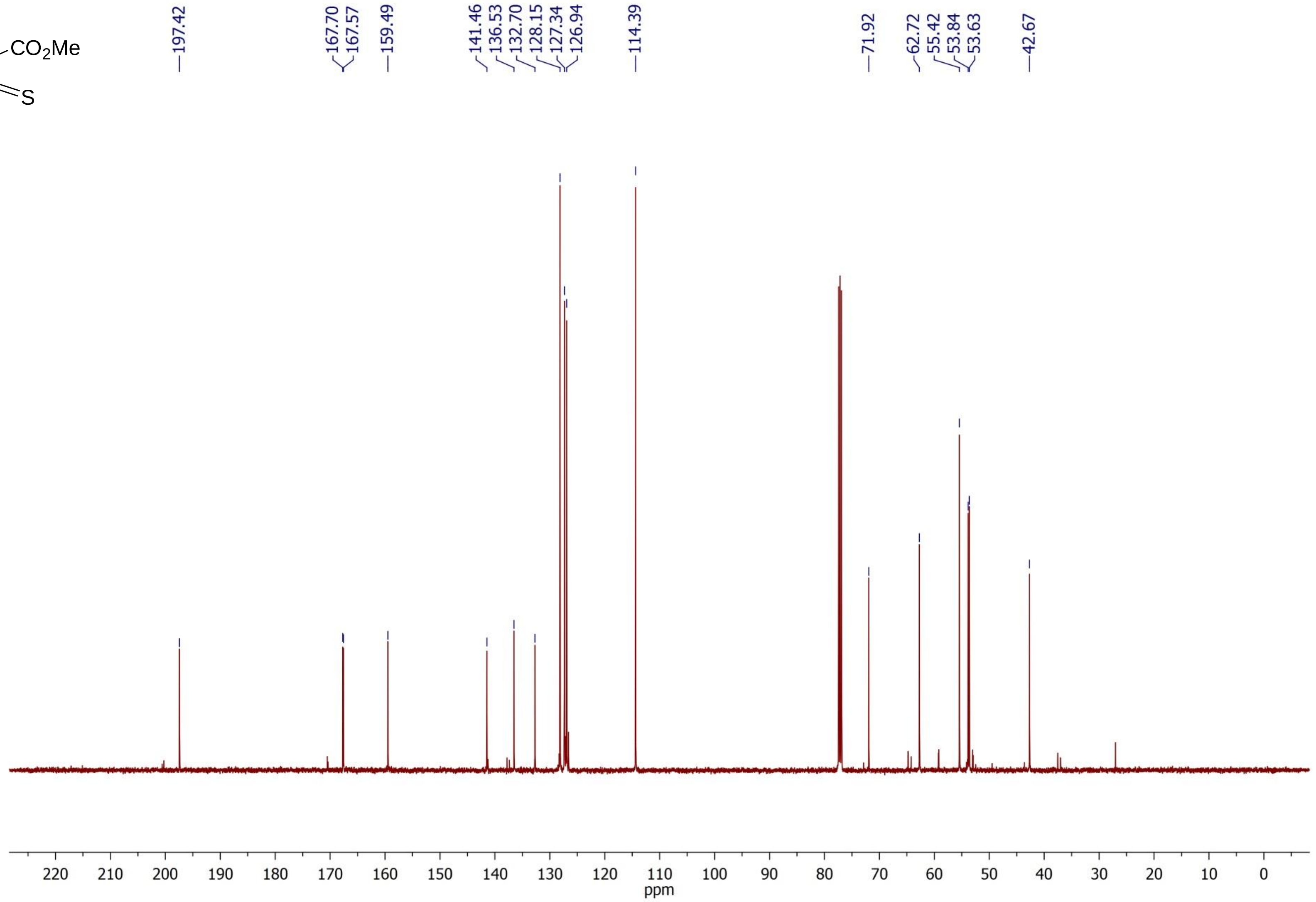

Dimethyl 5-[6-methoxynaphthalen-2-yl]-2-thioxopyrrolidine-3,3-dicarboxylate (2p)

^{1}H NMR (500 MHz, $CDCl_3$)

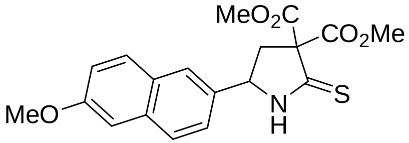

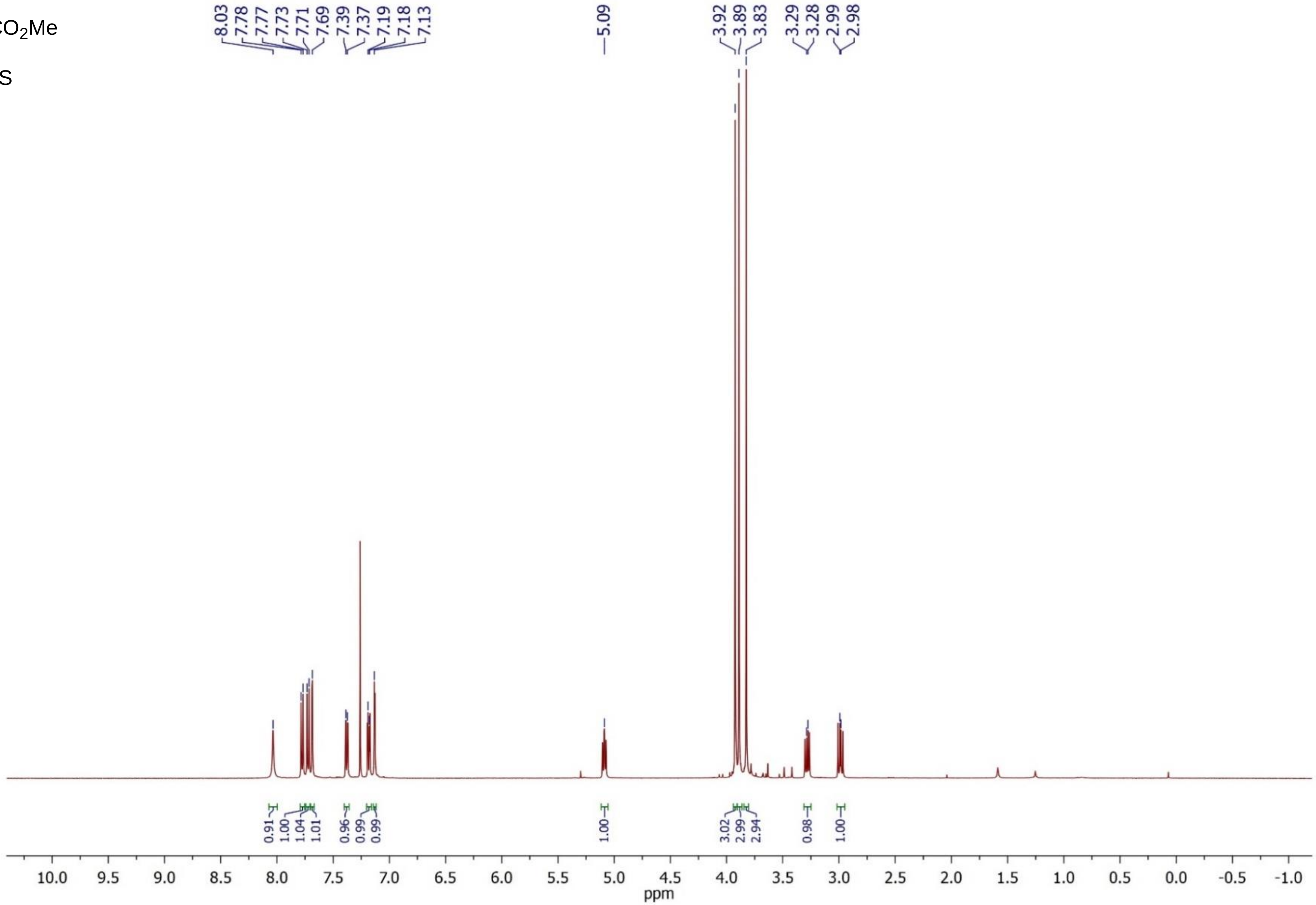

Dimethyl 5-[6-methoxynaphthalen-2-yl]-2-thioxopyrrolidine-3,3-dicarboxylate (2p)

^{13}C NMR (126 MHz, $CDCl_3$/DMSO-d_6, 4:1)

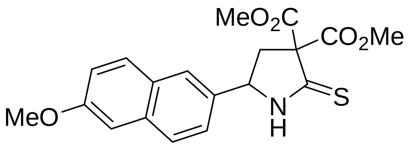

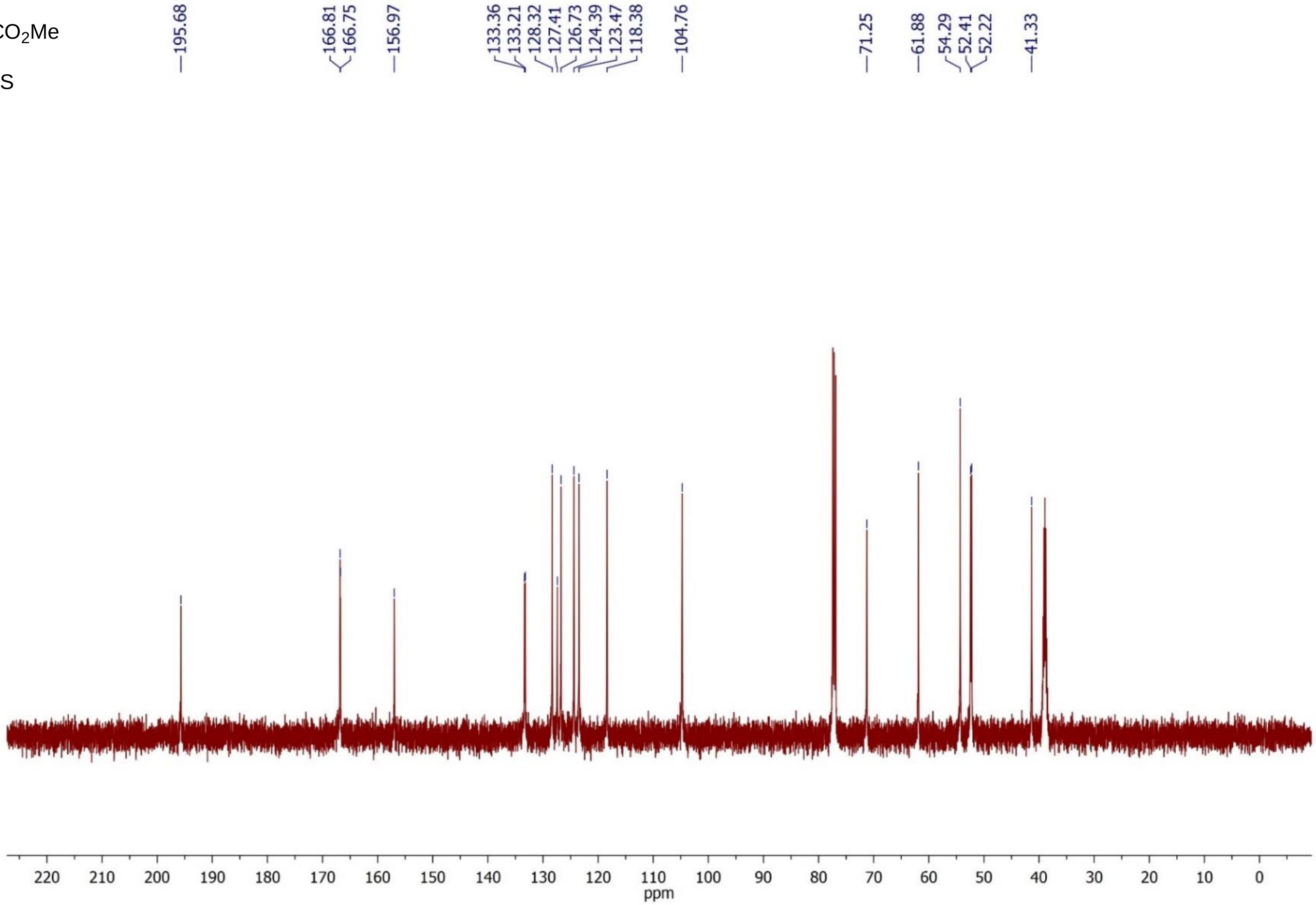

Dimethyl 5-(1,3-dioxoisoindolin-2-yl)-2-thioxopyrrolidine-3,3-dicarboxylate (2q)

^{1}H NMR (500 MHz, DMSO-d$_6$)

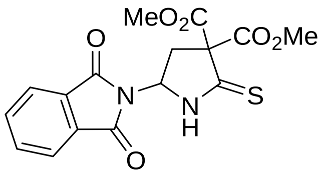

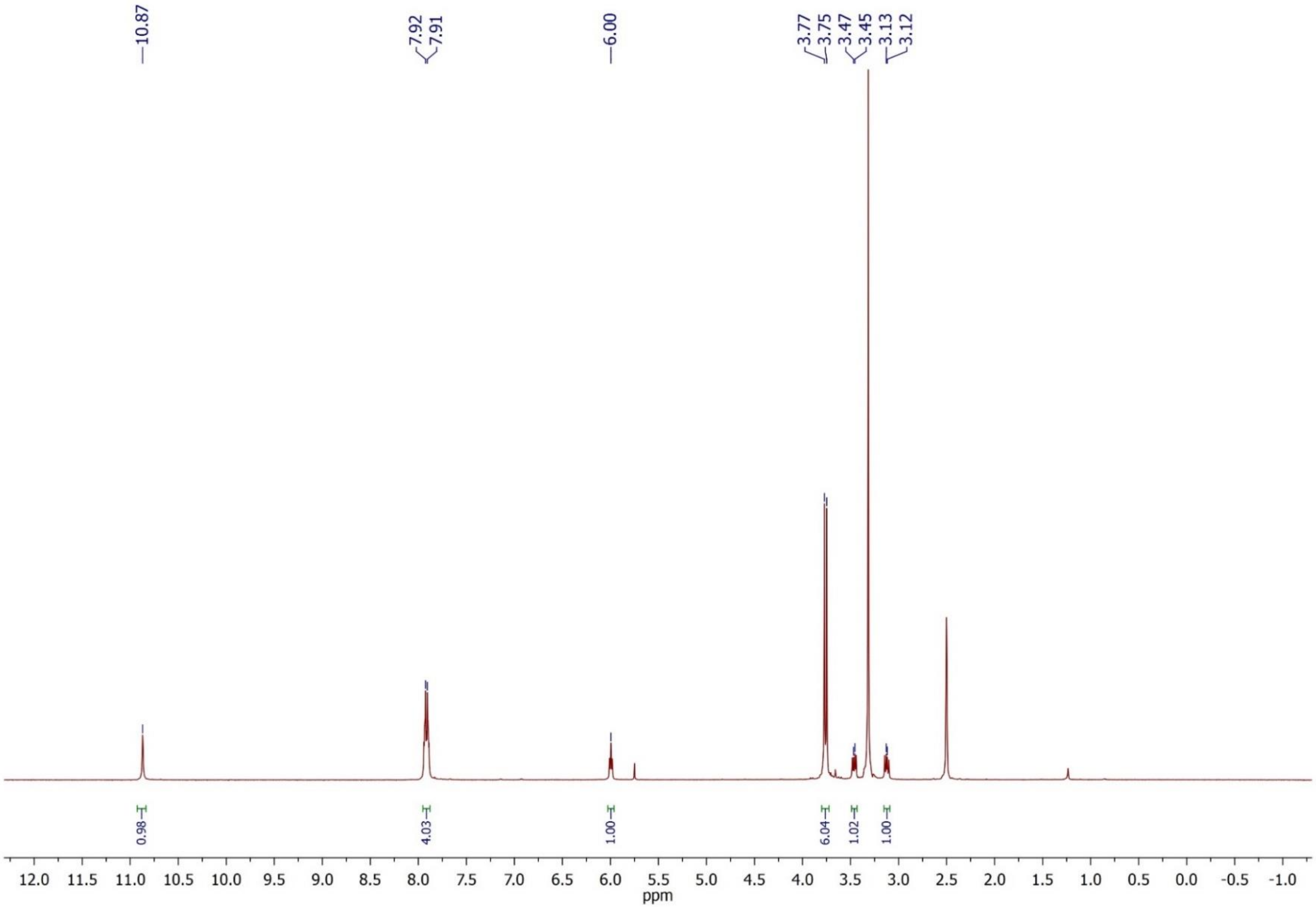

Dimethyl 5-(1,3-dioxoisoindolin-2-yl)-2-thioxopyrrolidine-3,3-dicarboxylate (2q)

^{13}C NMR (126 MHz, DMSO-d_6)

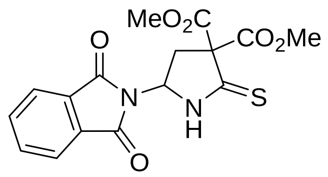

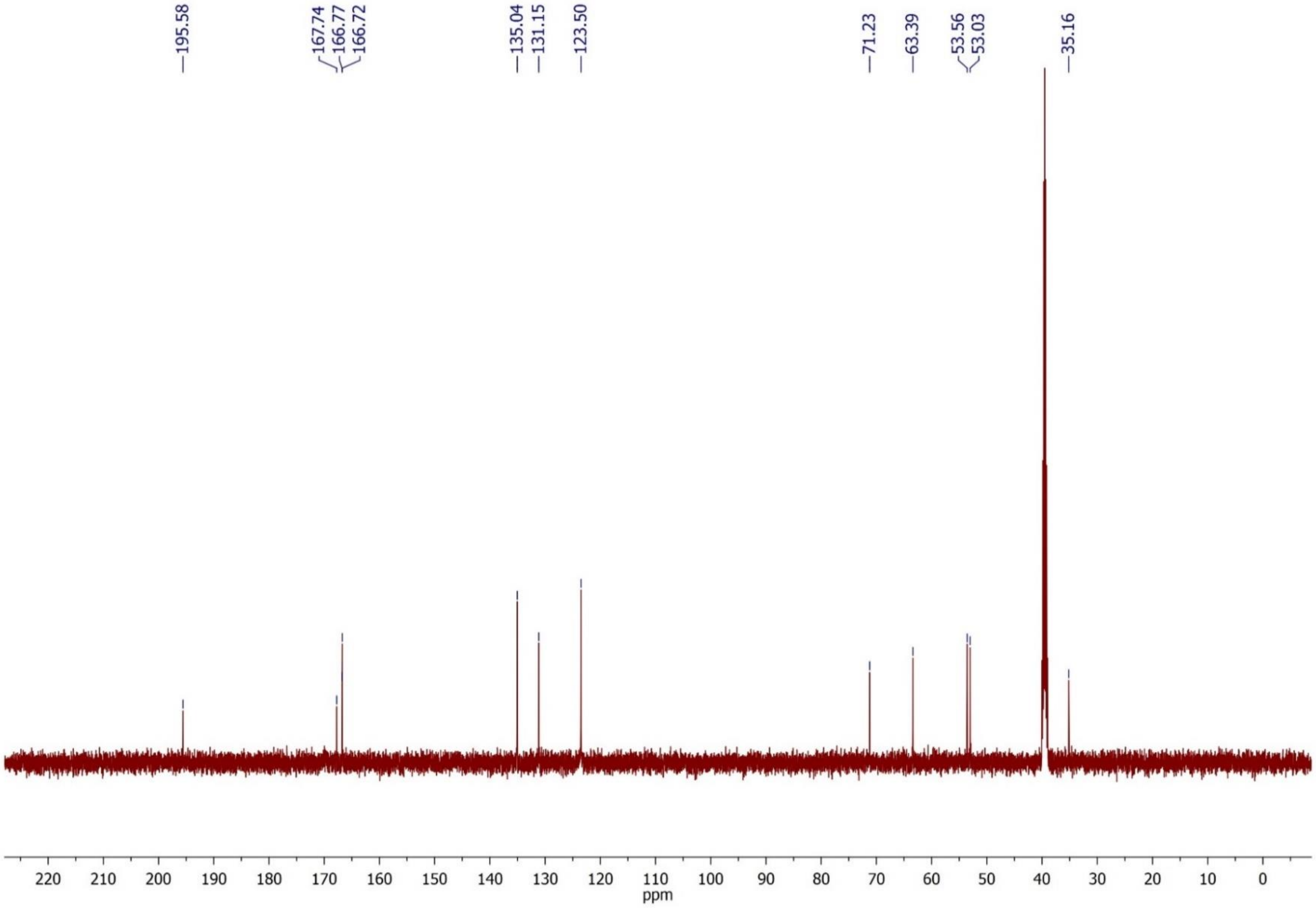

Dimethyl 5-(2,5-dioxopyrrolidin-1-yl)-2-thioxopyrrolidine-3,3-dicarboxylate (2r)

^{1}H NMR (500 MHz, DMSO-d_6)

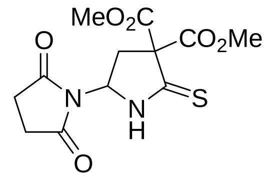

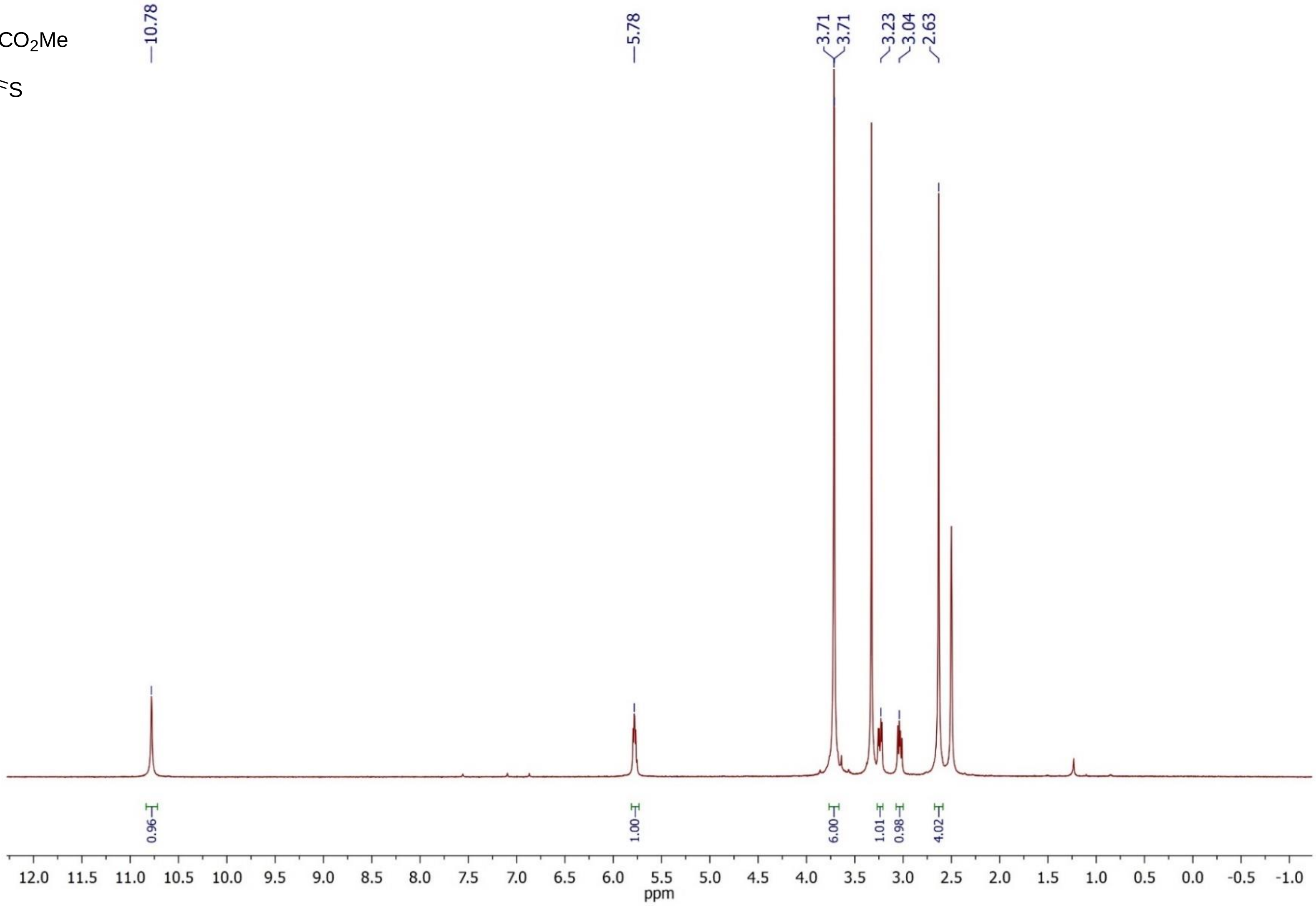

Dimethyl 5-(2,5-dioxopyrrolidin-1-yl)-2-thioxopyrrolidine-3,3-dicarboxylate (2r)

^{13}C NMR (126 MHz, DMSO-d_6)

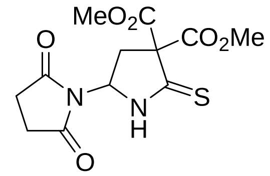

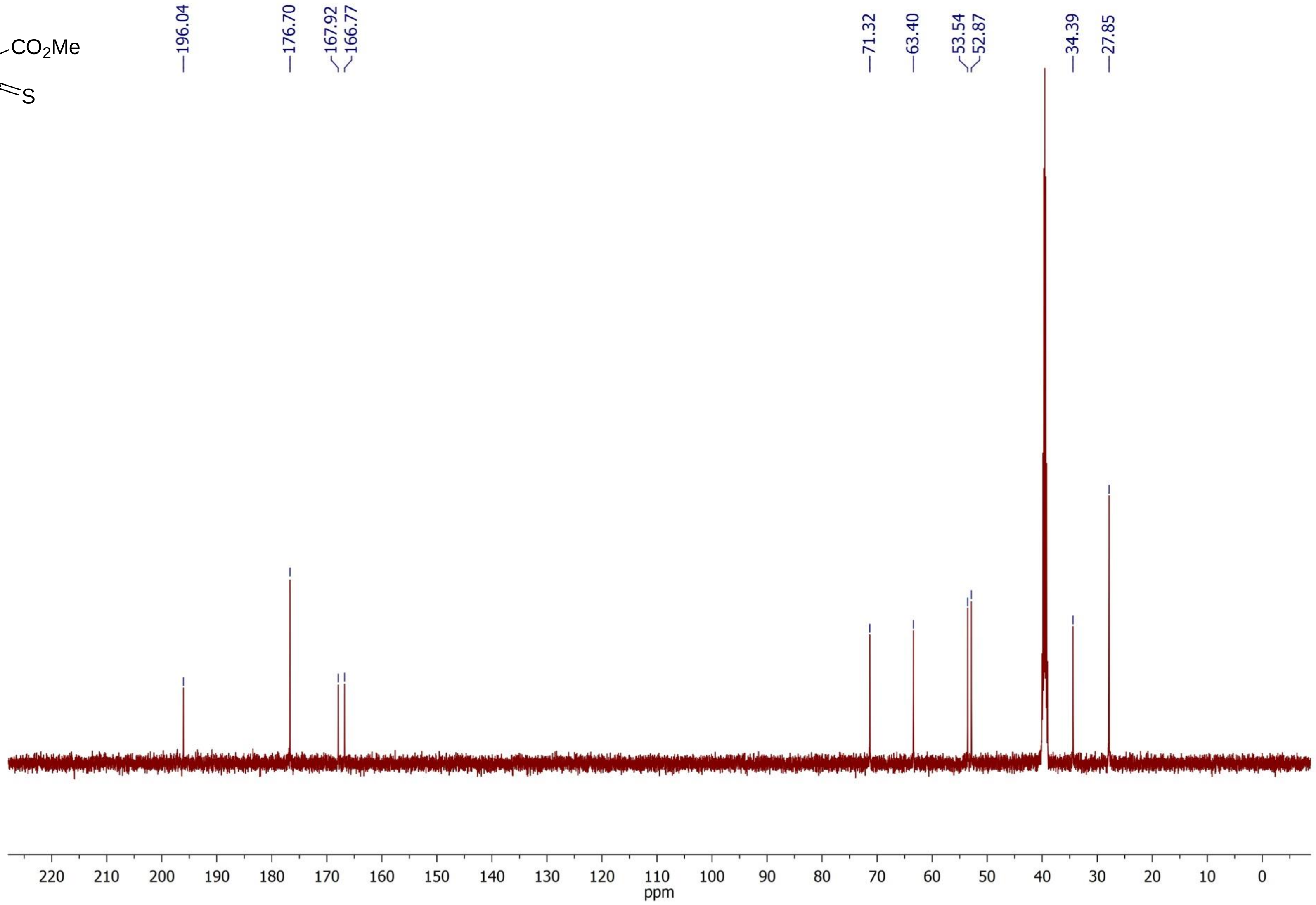

Dimethyl 5-(furan-2-yl)-2-thioxopyrrolidine-3,3-dicarboxylate (2s)

^1H NMR (500 MHz, $CDCl_3$)

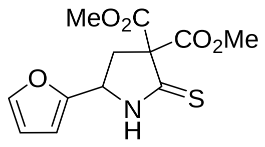

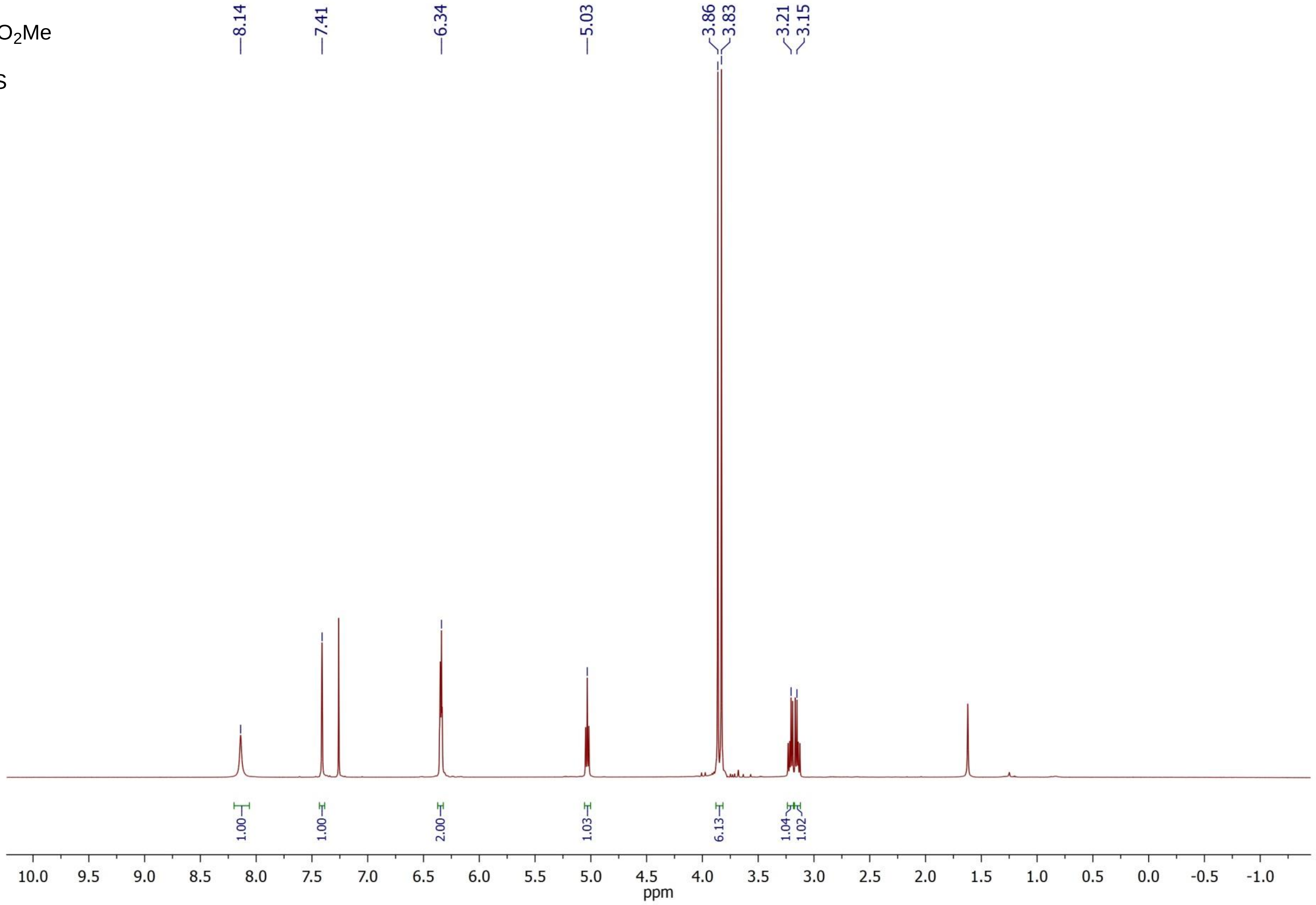

Dimethyl 5-(furan-2-yl)-2-thioxopyrrolidine-3,3-dicarboxylate (2s)

^{13}C NMR (126 MHz, $CDCl_3$)

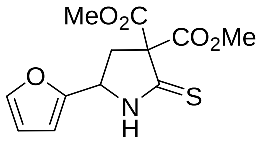

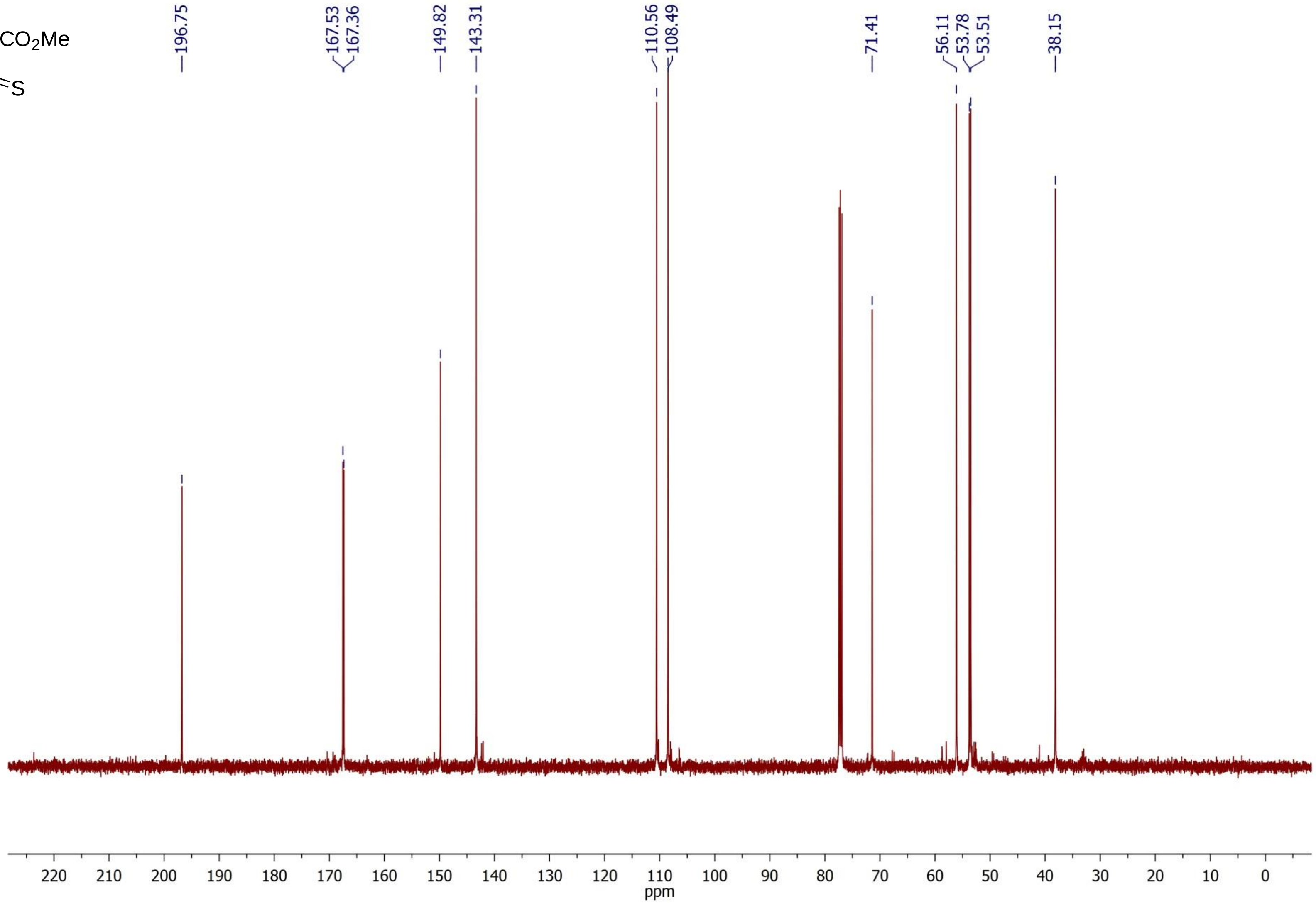

Dimethyl 5-(thiophen-2-yl)-2-thioxopyrrolidine-3,3-dicarboxylate (2t)

^{1}H NMR (500 MHz, $CDCl_3$)

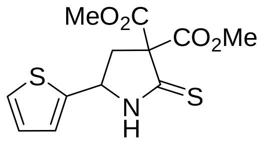

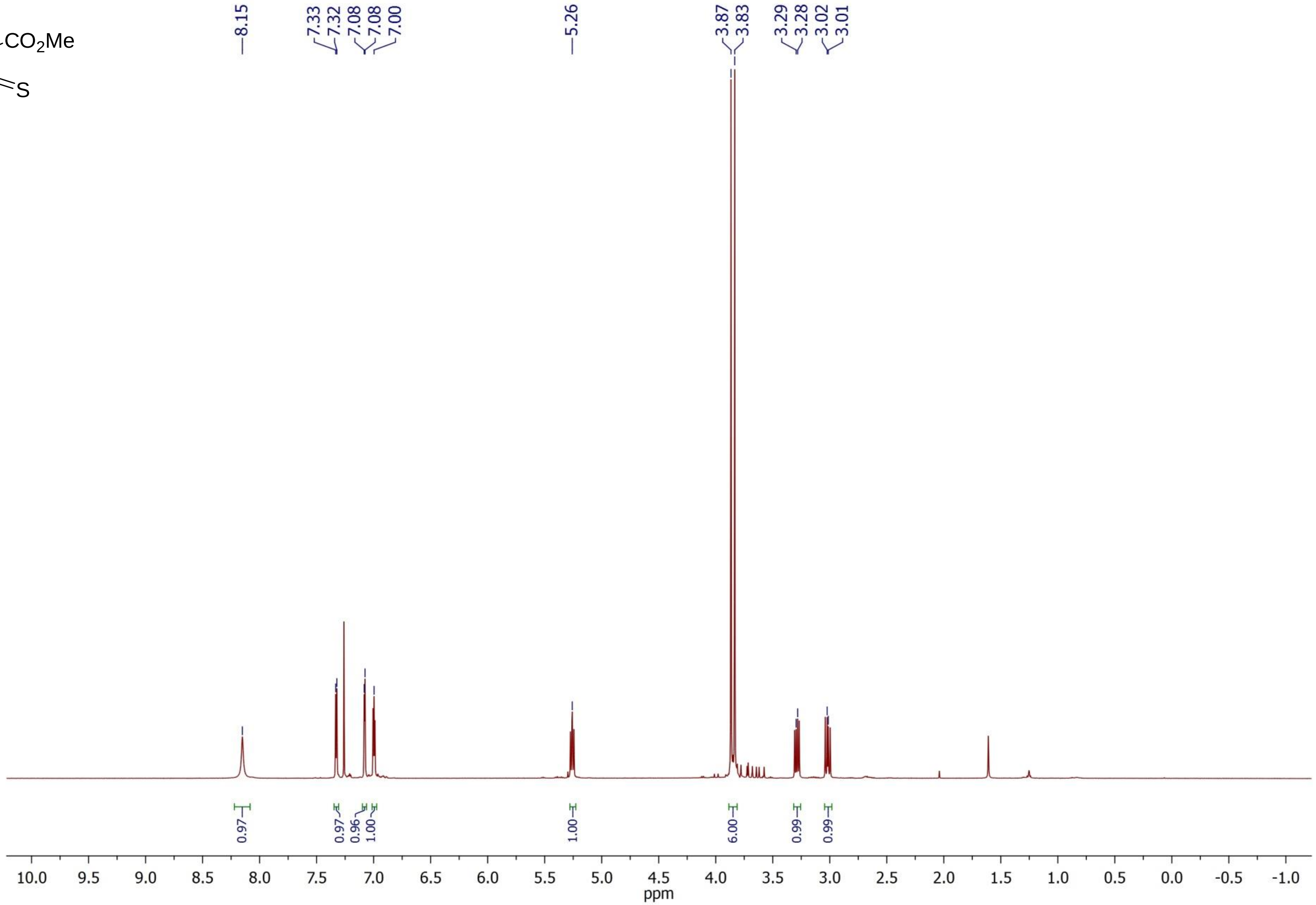

Dimethyl 5-(thiophen-2-yl)-2-thioxopyrrolidine-3,3-dicarboxylate (2t)

^{13}C NMR (126 MHz, $CDCl_3$/DMSO-d_6, 6:1)

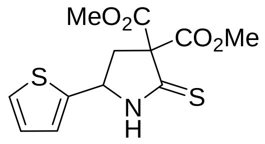

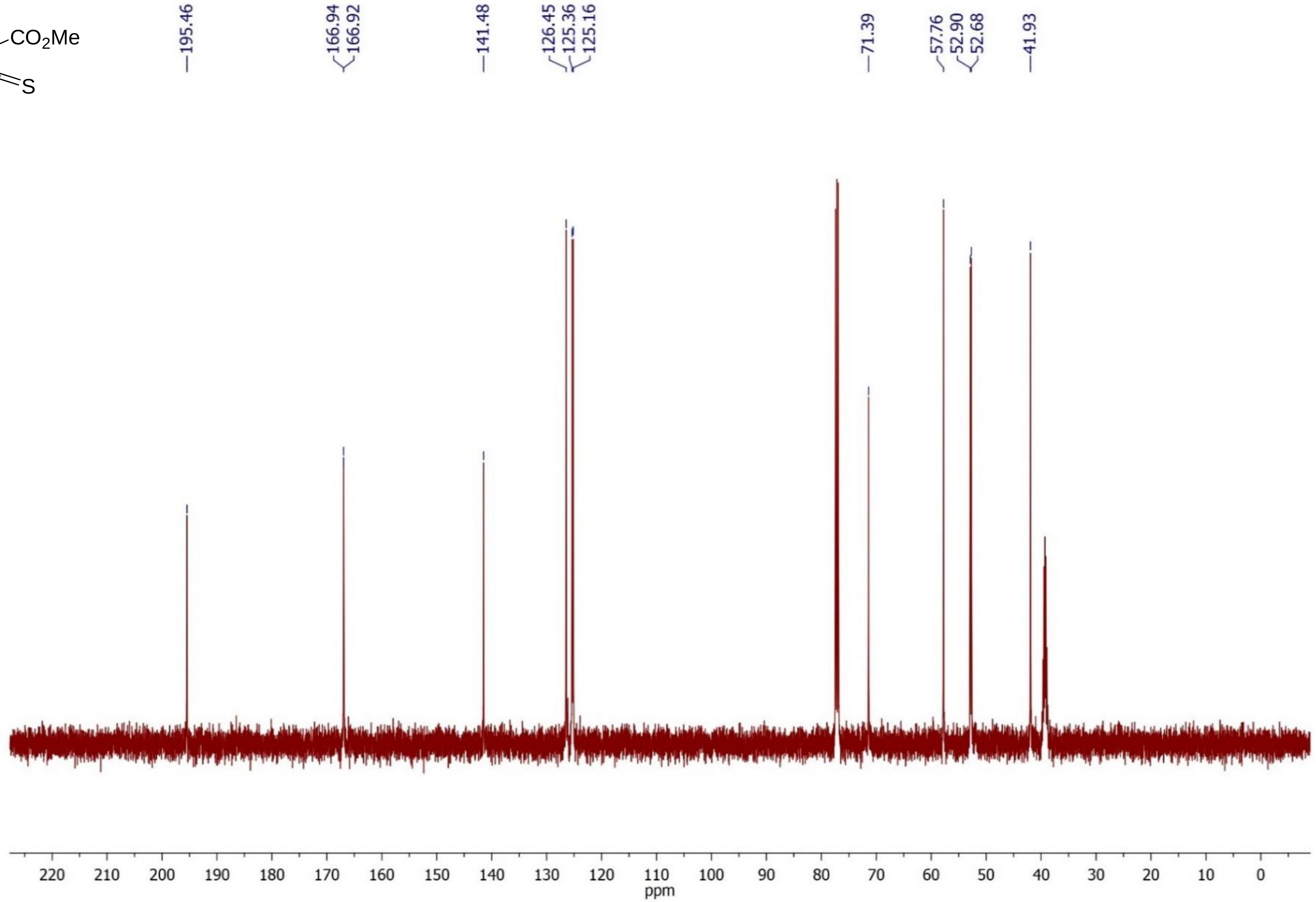

Dimethyl 5-(thiophen-3-yl)-2-thioxopyrrolidine-3,3-dicarboxylate (2u)

^{1}H NMR (500 MHz, $CDCl_3$)

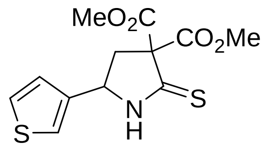

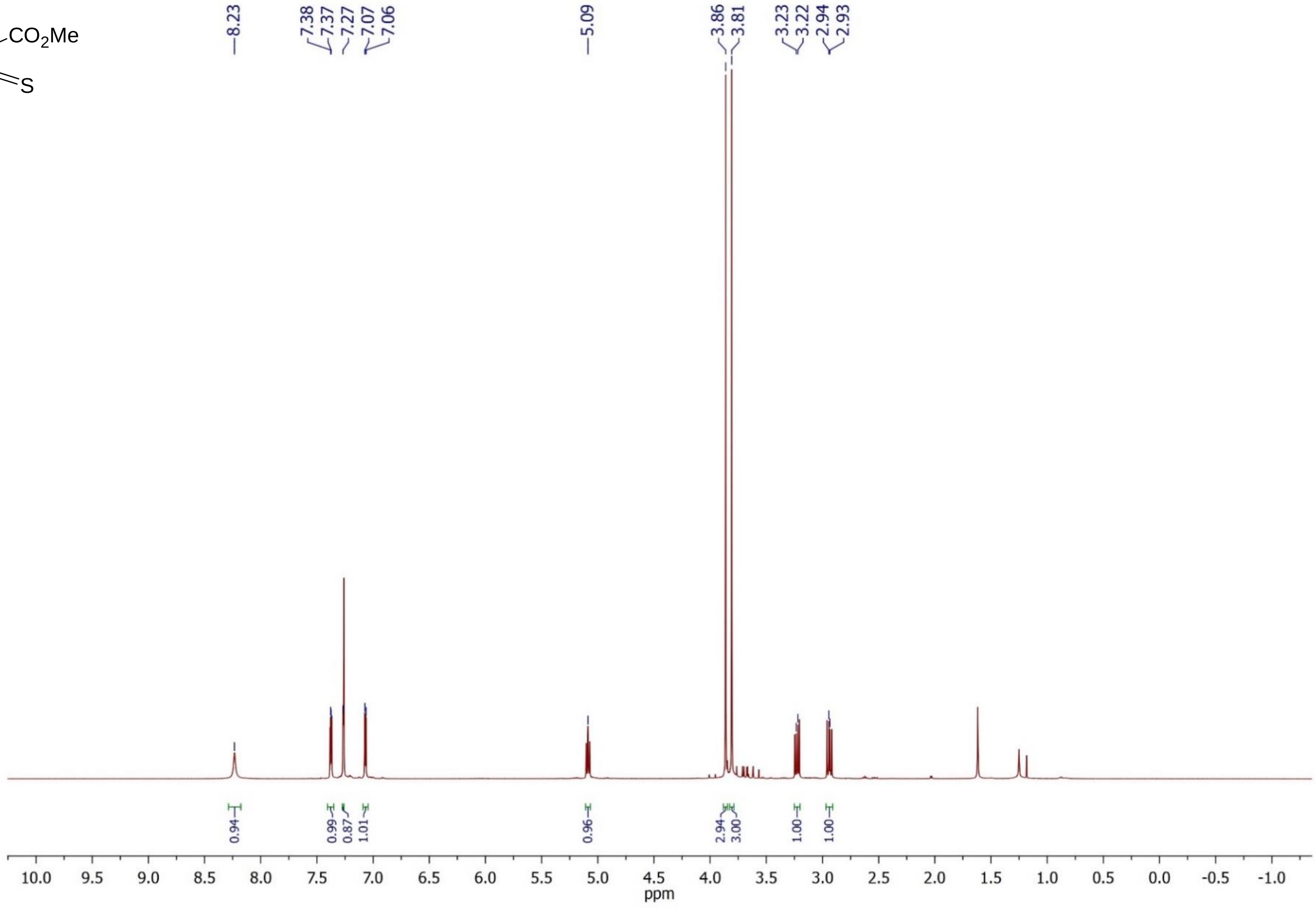

Dimethyl 5-(thiophen-3-yl)-2-thioxopyrrolidine-3,3-dicarboxylate (2u)

^{13}C NMR (126 MHz, $CDCl_3$)

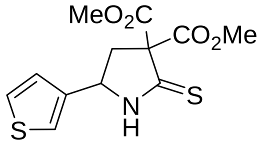

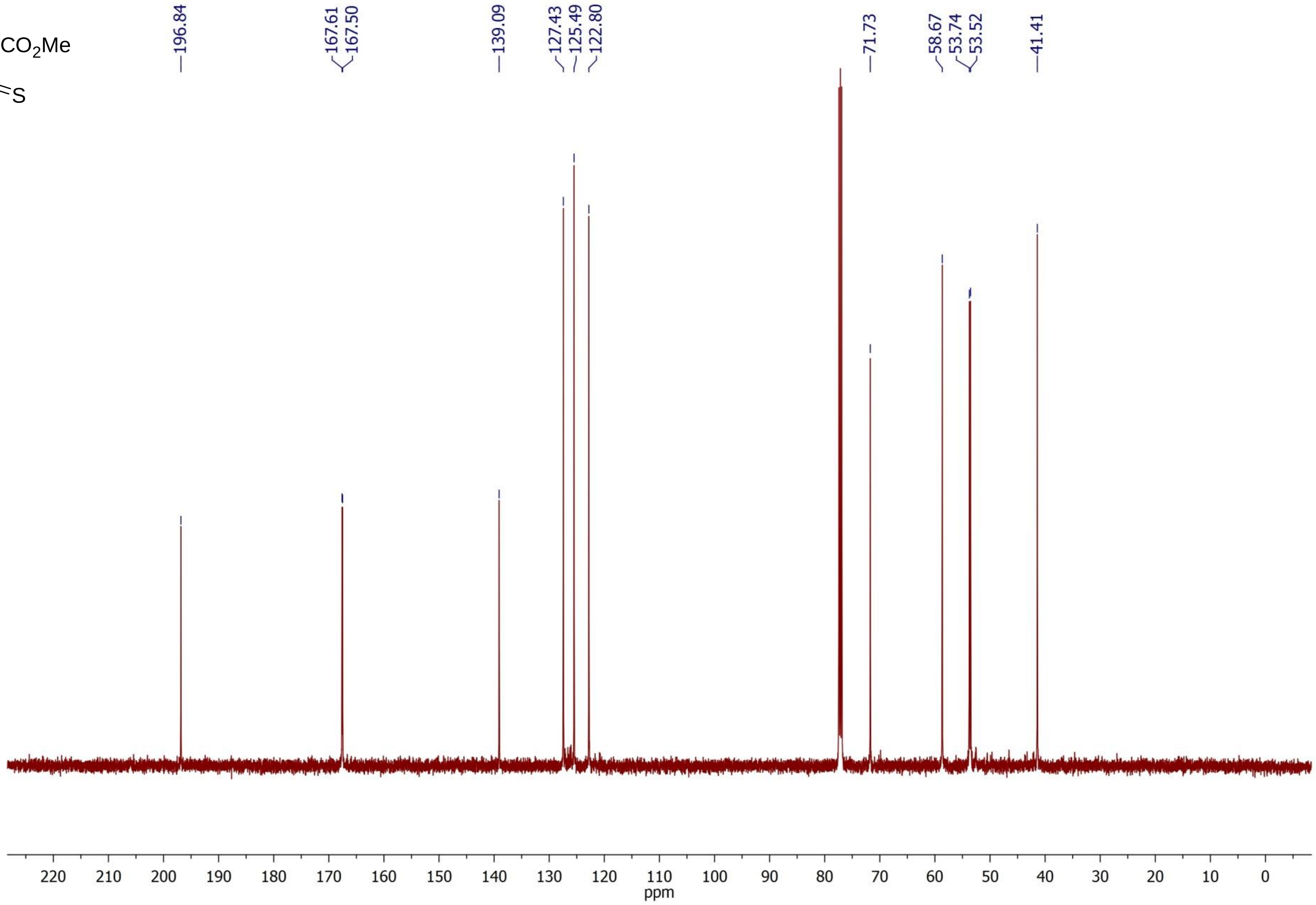

Dimethyl 5-(1-methylpyrrol-2-yl)-2-thioxopyrrolidine-3,3-dicarboxylate (2v)

^{1}H NMR (500 MHz, $CDCl_3$)

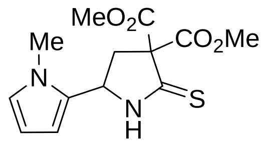

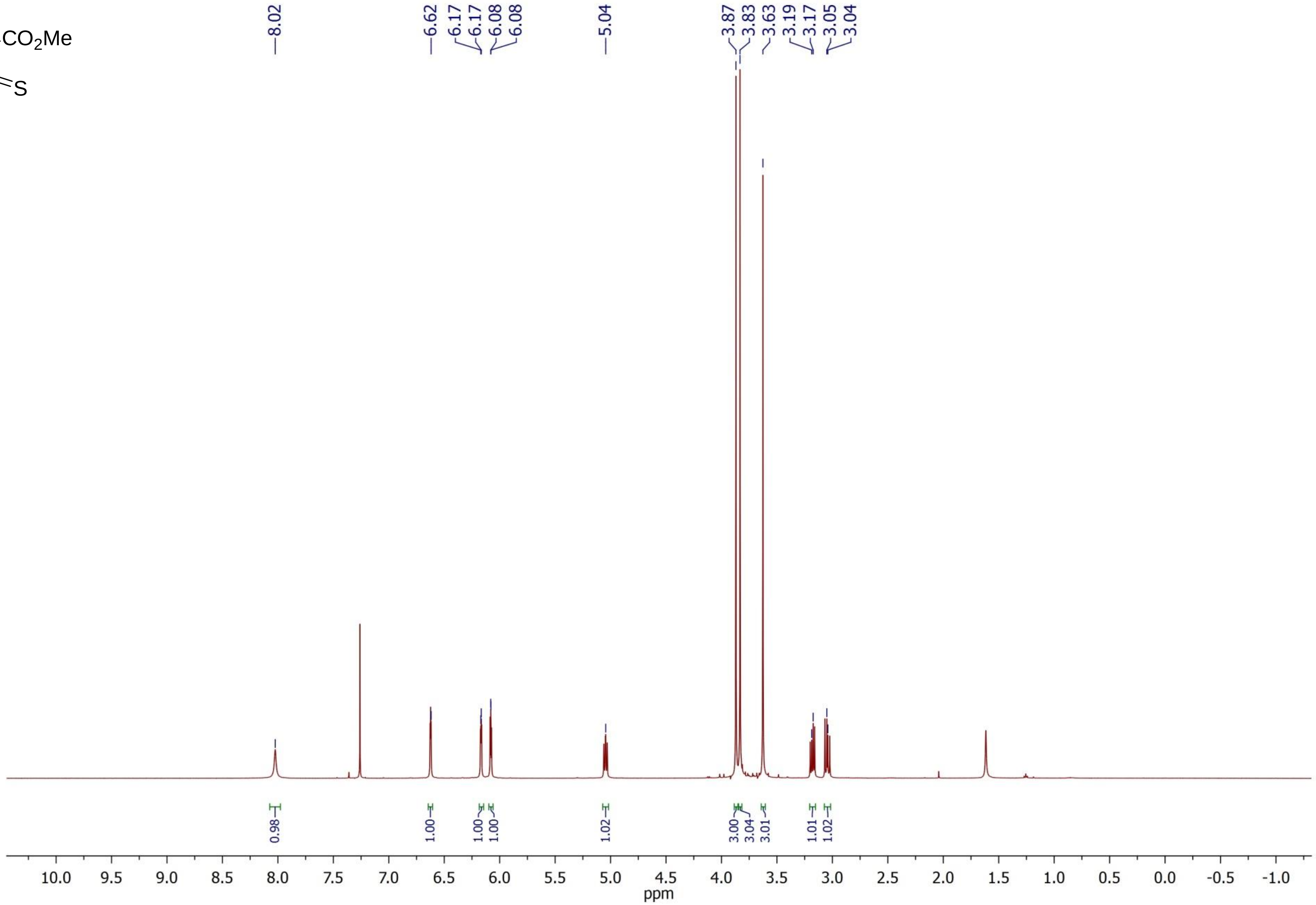

Dimethyl 5-(1-methylpyrrol-2-yl)-2-thioxopyrrolidine-3,3-dicarboxylate (2v)

^{13}C NMR (126 MHz, $CDCl_3$/DMSO-d_6, 10:1)

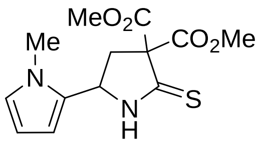

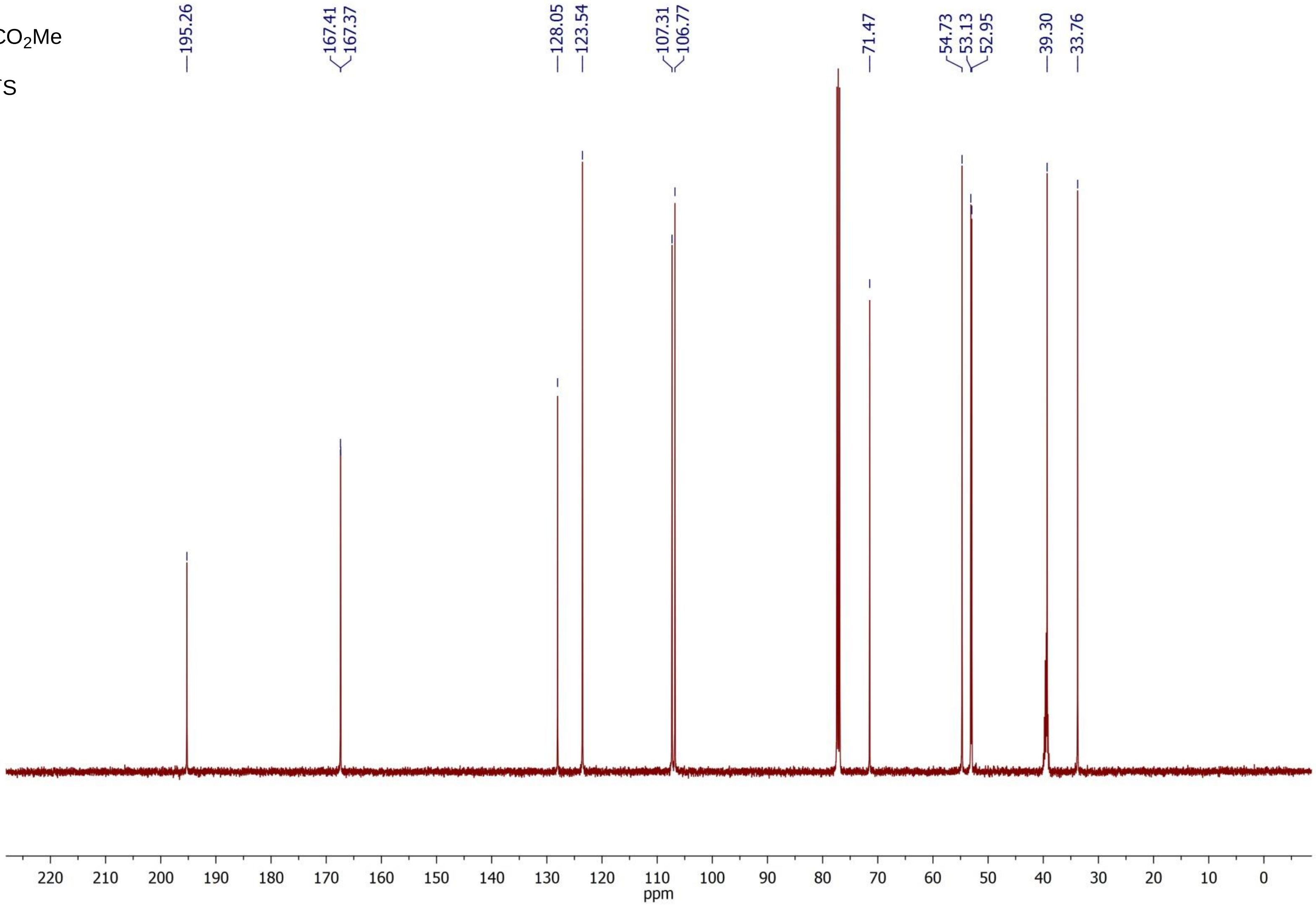

Dimethyl 5-(1-benzyl-1*H*-indol-4-yl)-2-thioxopyrrolidine-3,3-dicarboxylate (2w)

^{1}H NMR (500 MHz, $CDCl_3$)

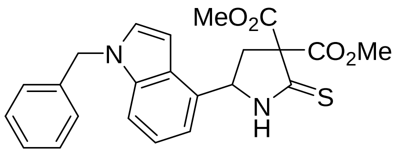

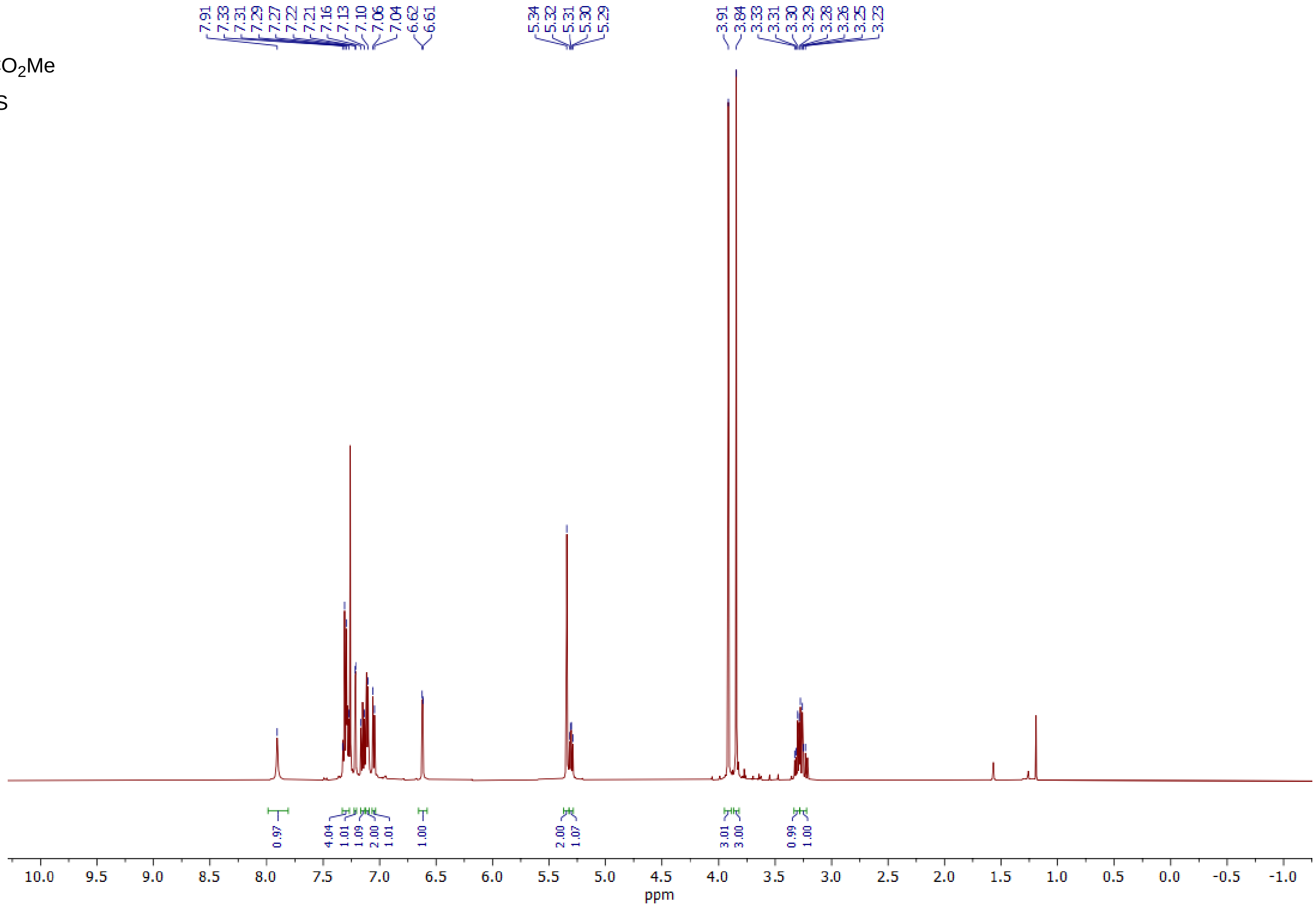

Dimethyl 5-(1-benzyl-1*H*-indol-4-yl)-2-thioxopyrrolidine-3,3-dicarboxylate (2w)

^{13}C NMR (126 MHz, $CDCl_3$)

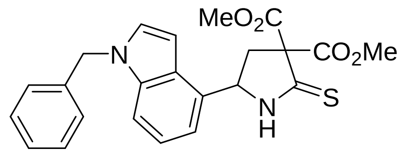

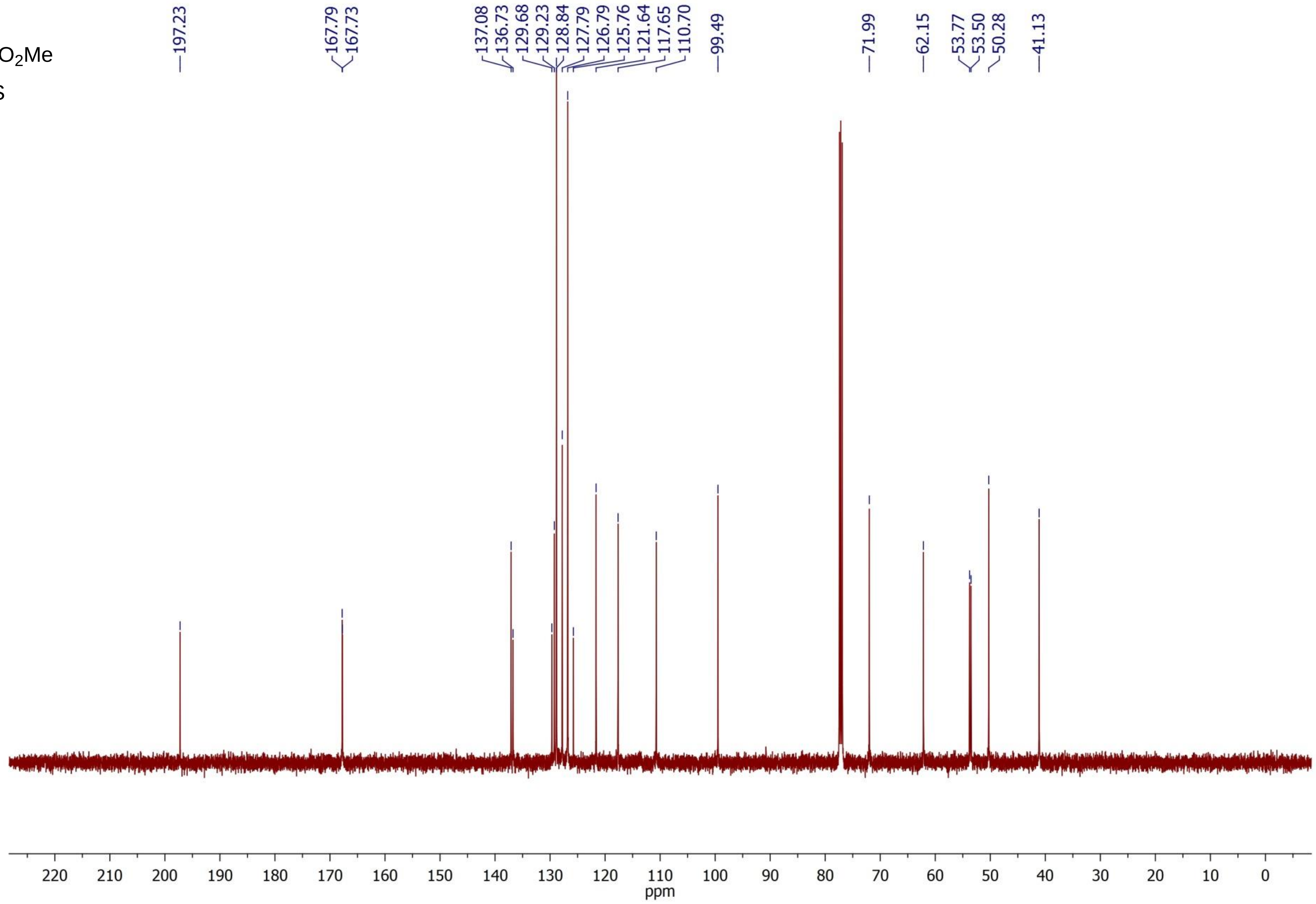

Dimethyl 5-(1-benzyl-1*H*-indol-4-yl)-2-thioxopyrrolidine-3,3-dicarboxylate (2w)

^{1}H–^{13}C HSQC ($CDCl_3$)

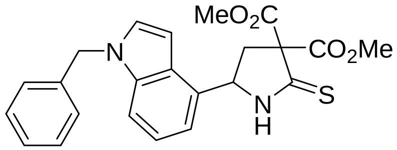

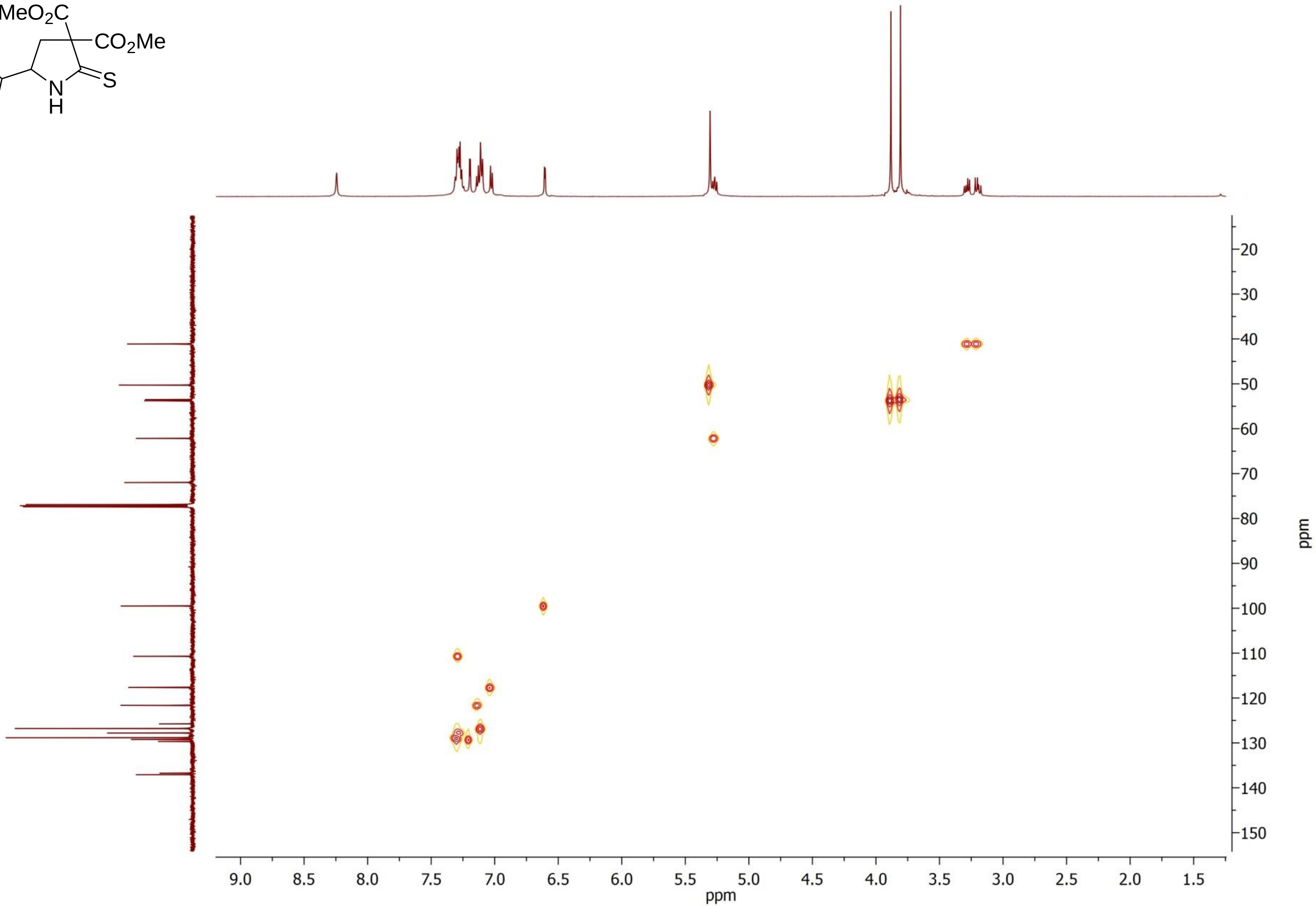

Dimethyl 5-(1-benzyl-1*H*-indol-4-yl)-2-thioxopyrrolidine-3,3-dicarboxylate (2w)

^1H–^{13}C HMBC ($CDCl_3$)

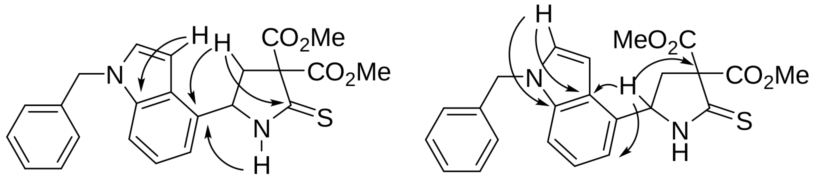

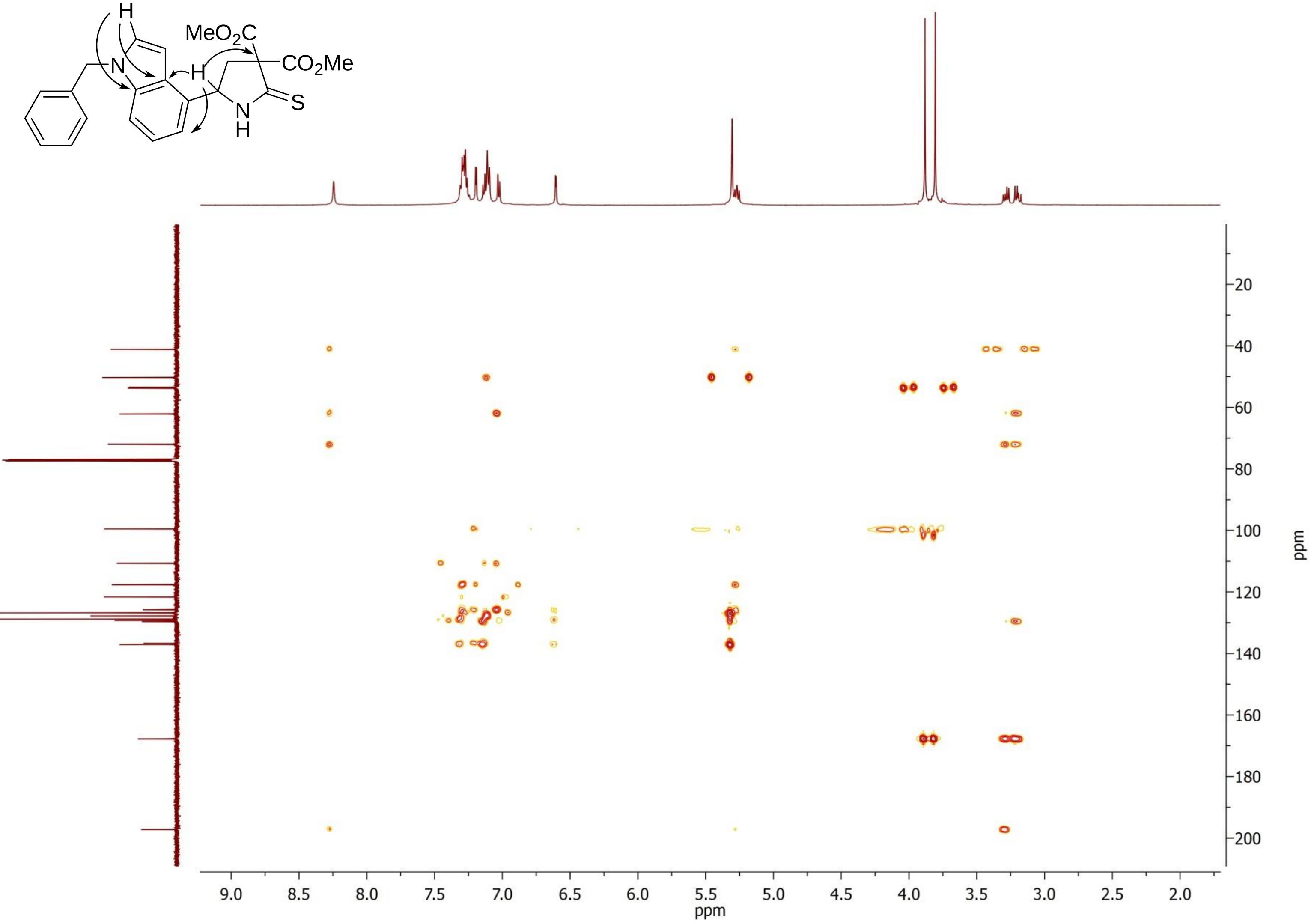

Dimethyl 5-(1-ferrocenyl)-2-thioxopyrrolidine-3,3-dicarboxylate (2x)

^{1}H NMR (500 MHz, $CDCl_3$)

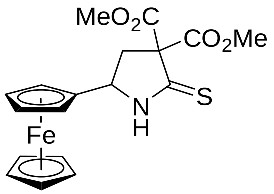

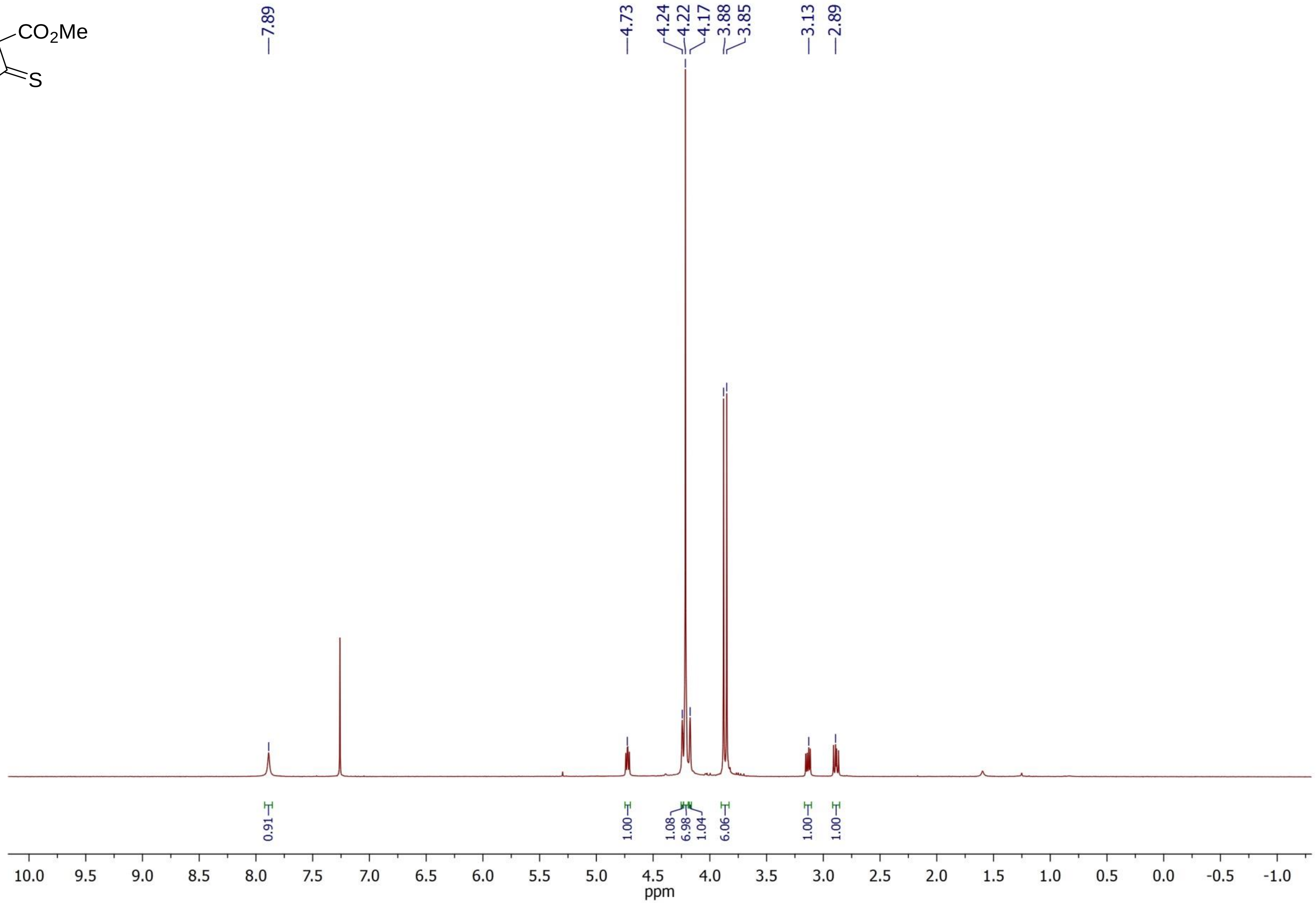

Dimethyl 5-(1-ferrocenyl)-2-thioxopyrrolidine-3,3-dicarboxylate (2x)

^{1}H NMR (500 MHz, DMSO-d_6)

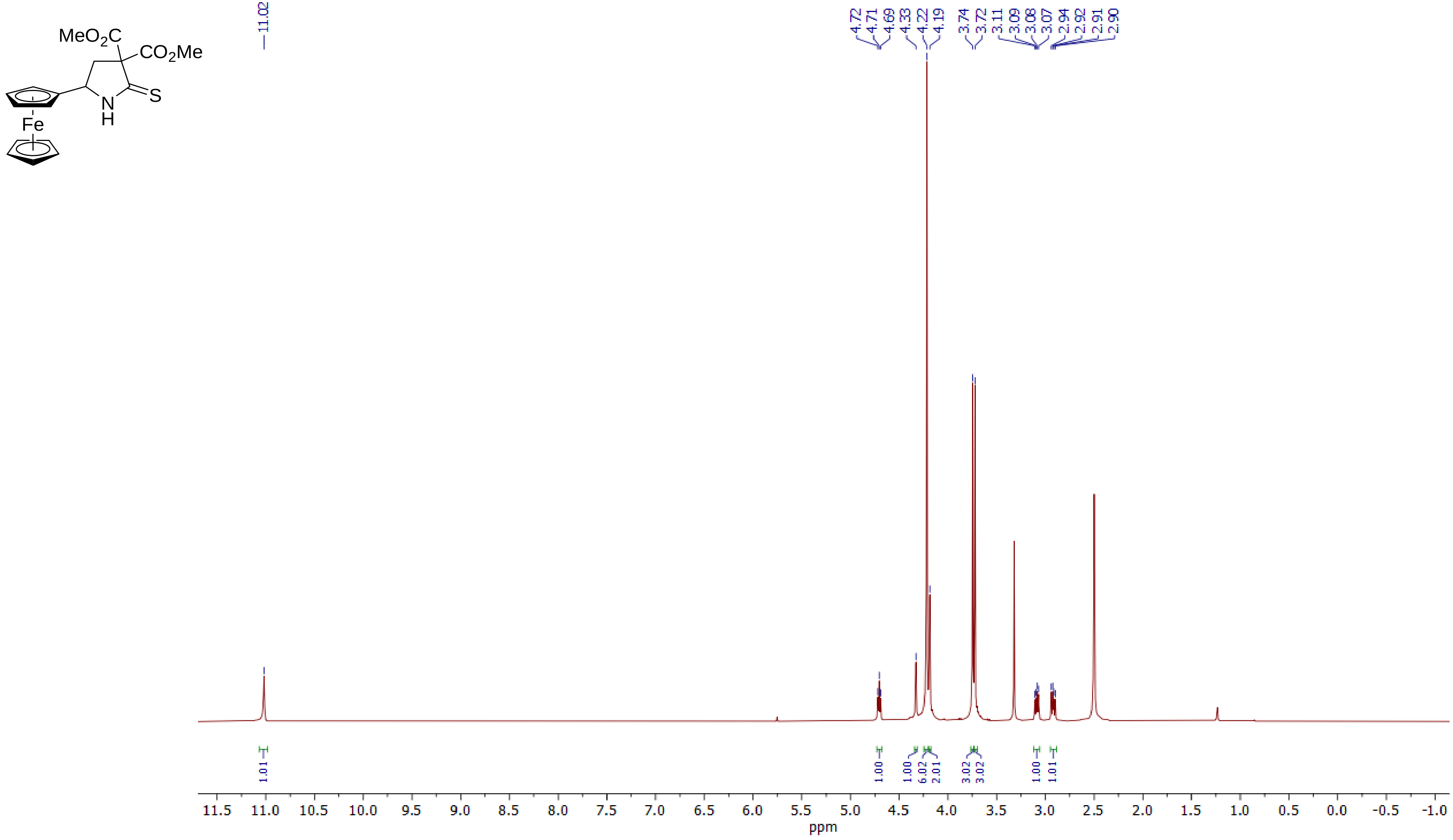

Dimethyl 5-(1-ferrocenyl)-2-thioxopyrrolidine-3,3-dicarboxylate (2x)

^{13}C NMR (126 MHz, DMSO-d_6)

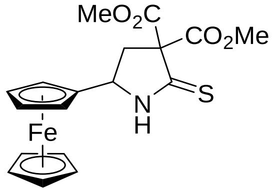

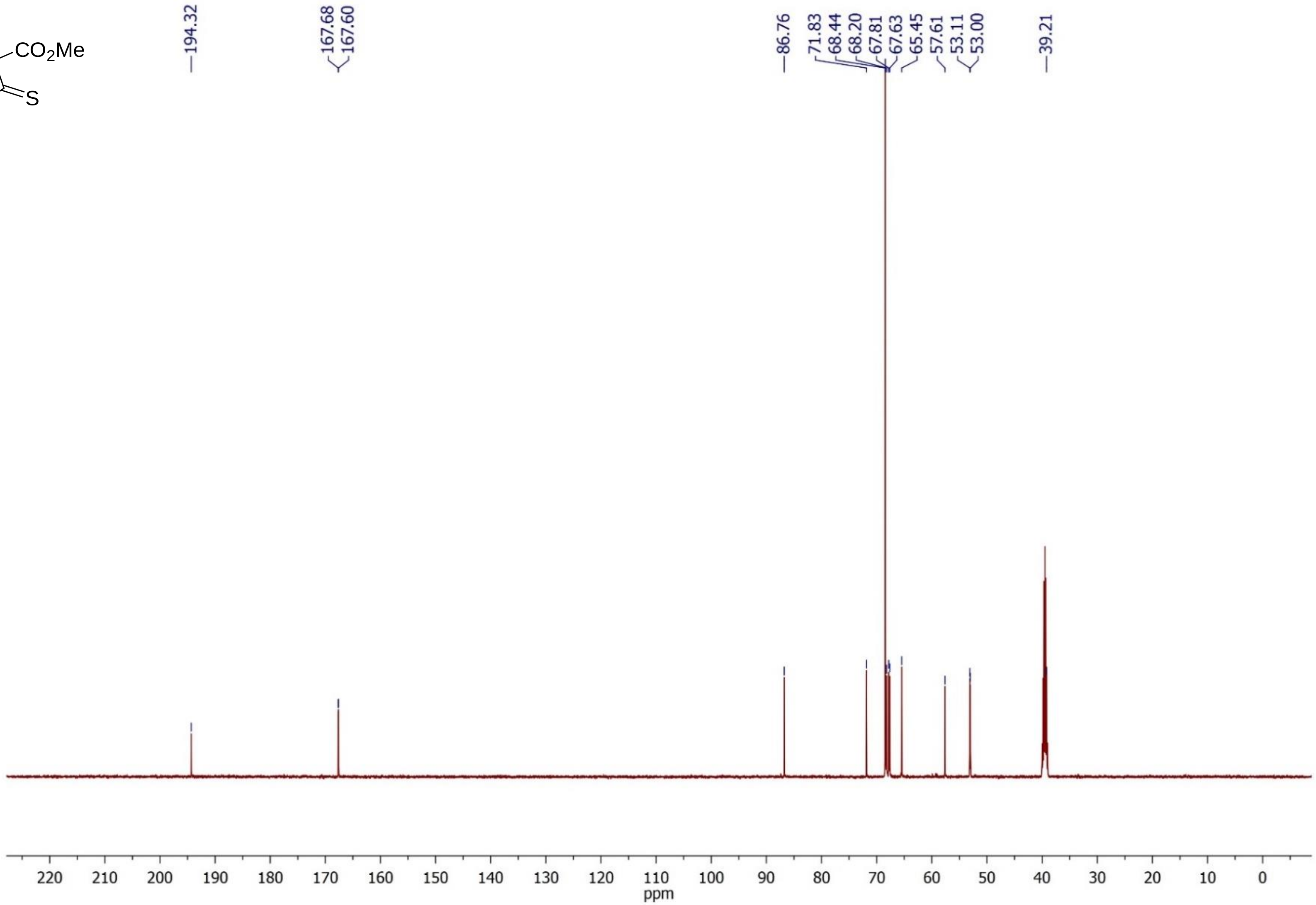

Dimethyl 5-(1-ferrocenyl)-2-thioxopyrrolidine-3,3-dicarboxylate (2x)

^{1}H–^{13}C HSQC ($CDCl_3$)

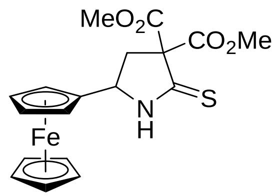

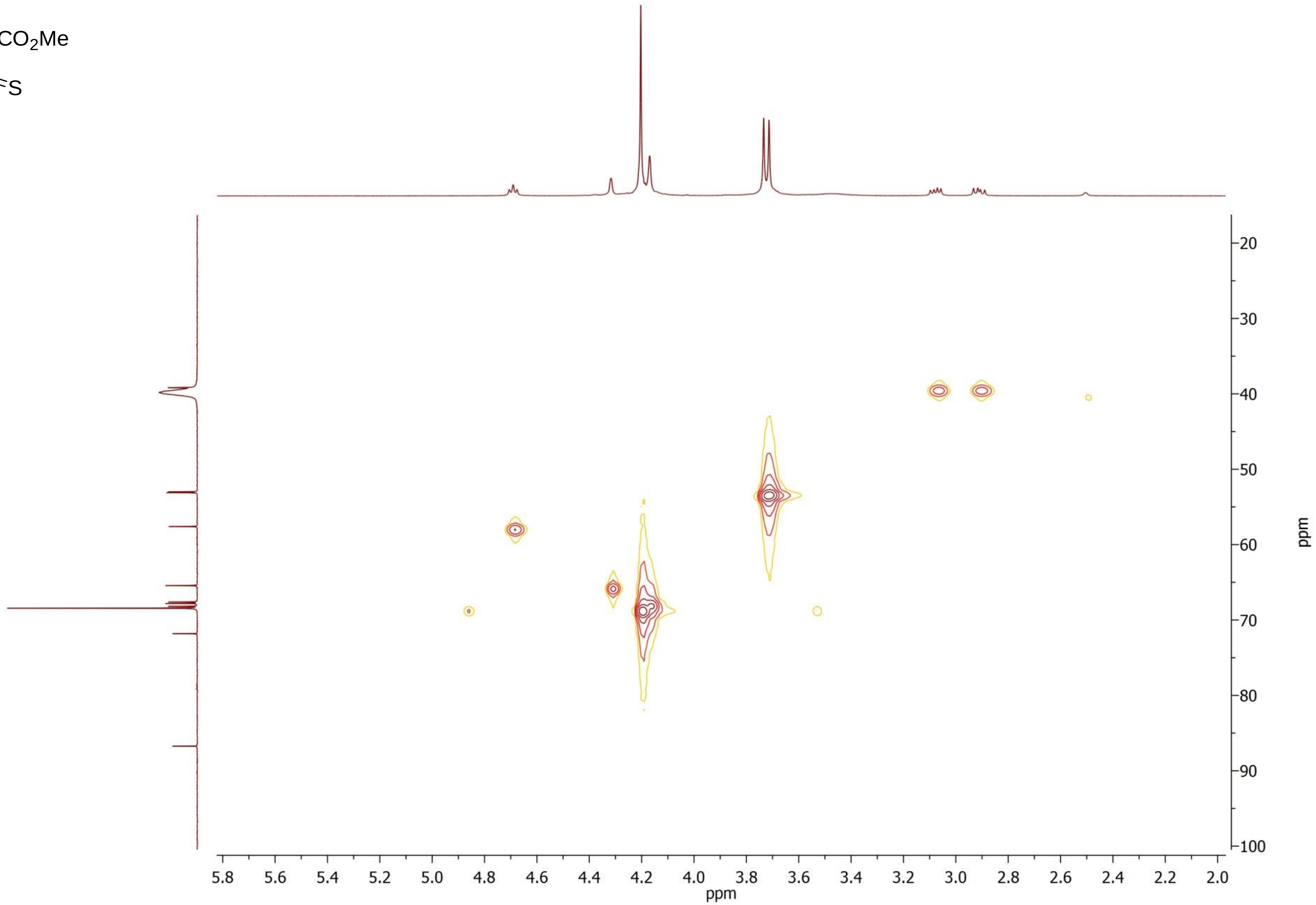

Dimethyl 5-ethenyl-2-thioxopyrrolidine-3,3-dicarboxylate (2y)

^{1}H NMR (500 MHz, $CDCl_3$)

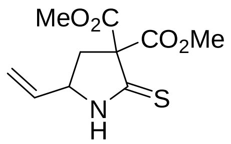

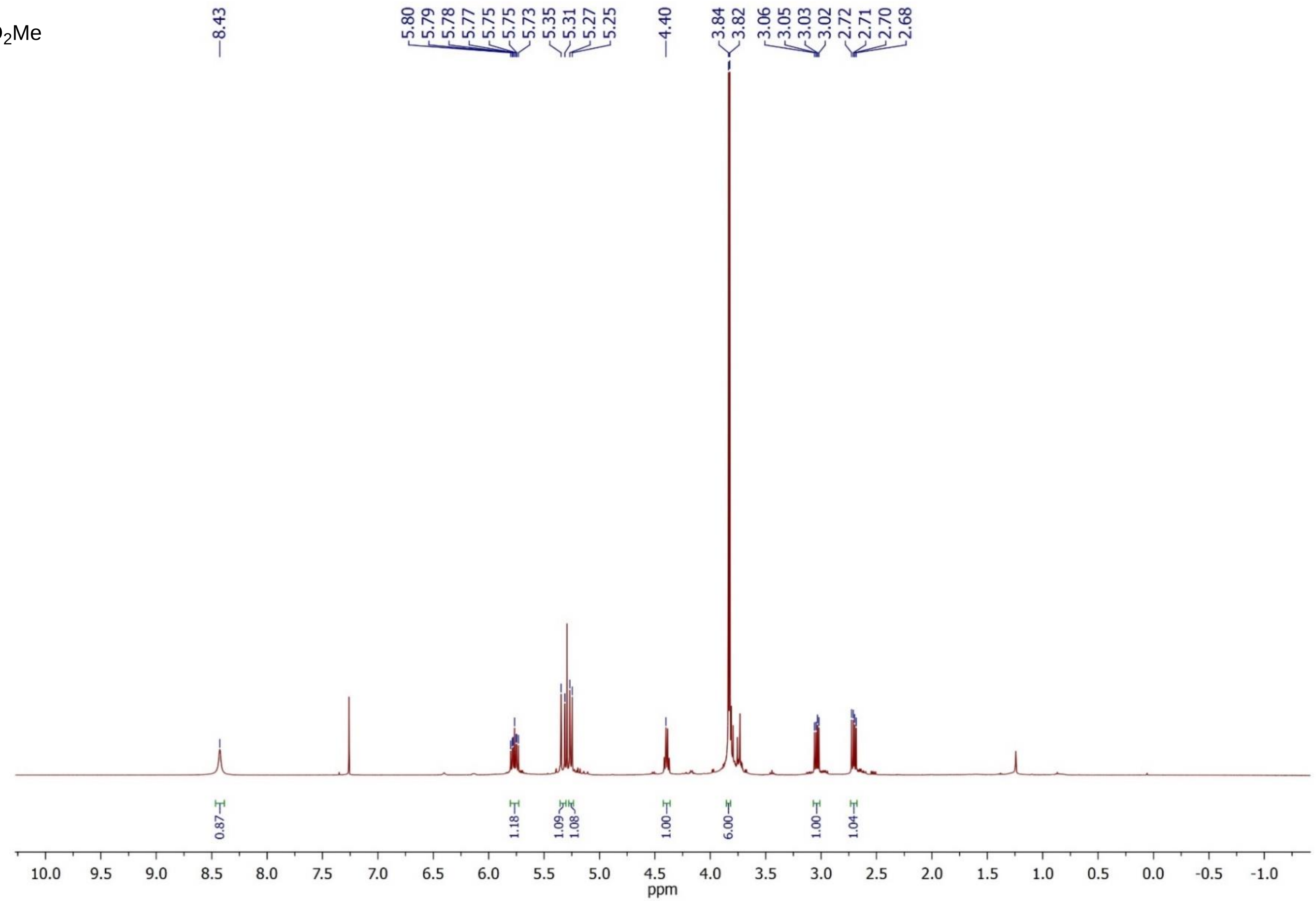

Dimethyl 5-ethenyl-2-thioxopyrrolidine-3,3-dicarboxylate (2y)

^{13}C NMR (126 MHz, $CDCl_3$)

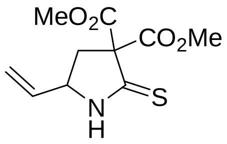

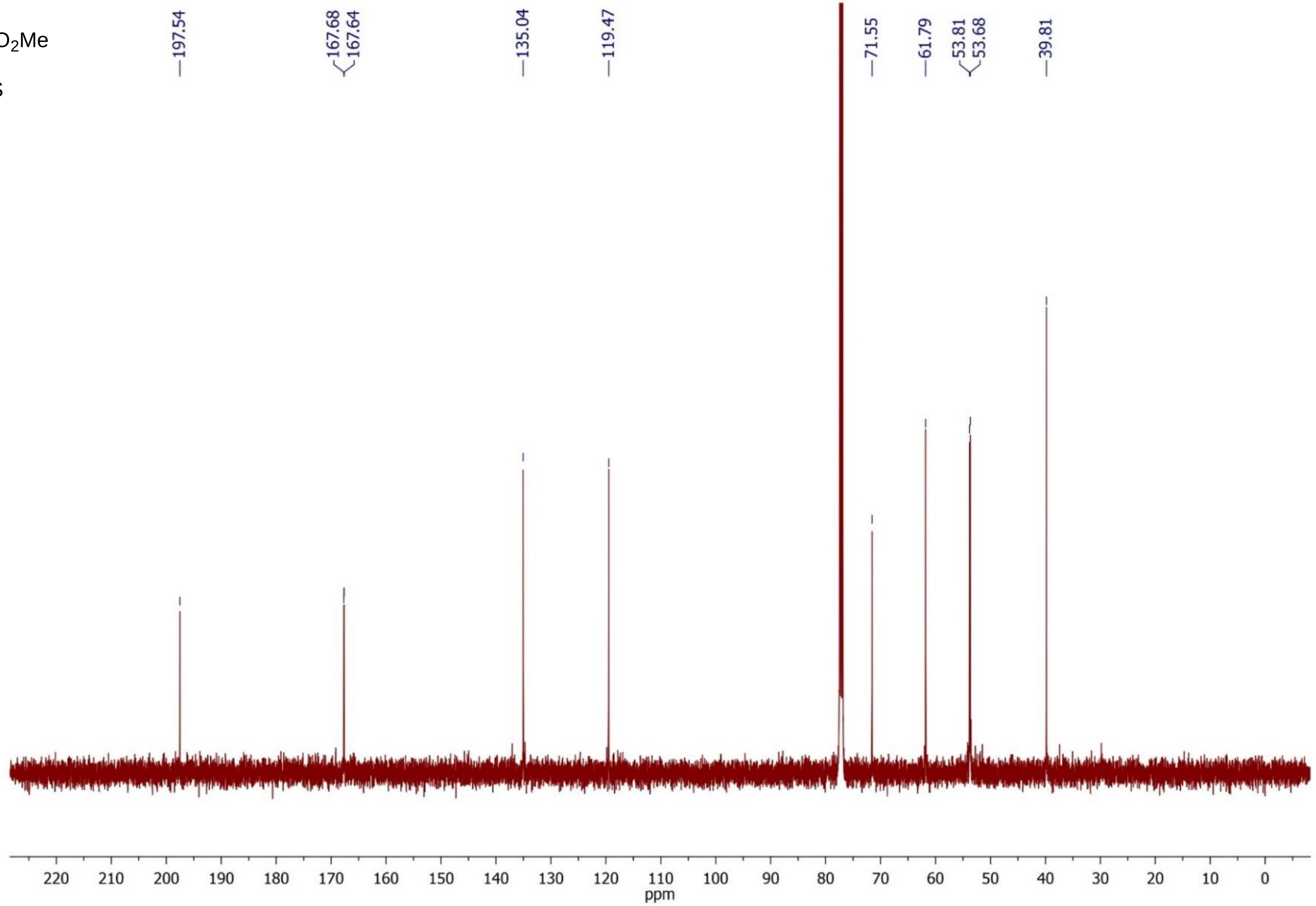

Dimethyl (*E*)-2-(4-isothiocyanatobut-2-en-1-yl)malonate (4)

^{1}H NMR (500 MHz, $CDCl_3$)

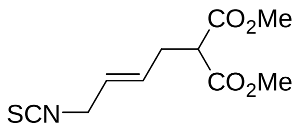

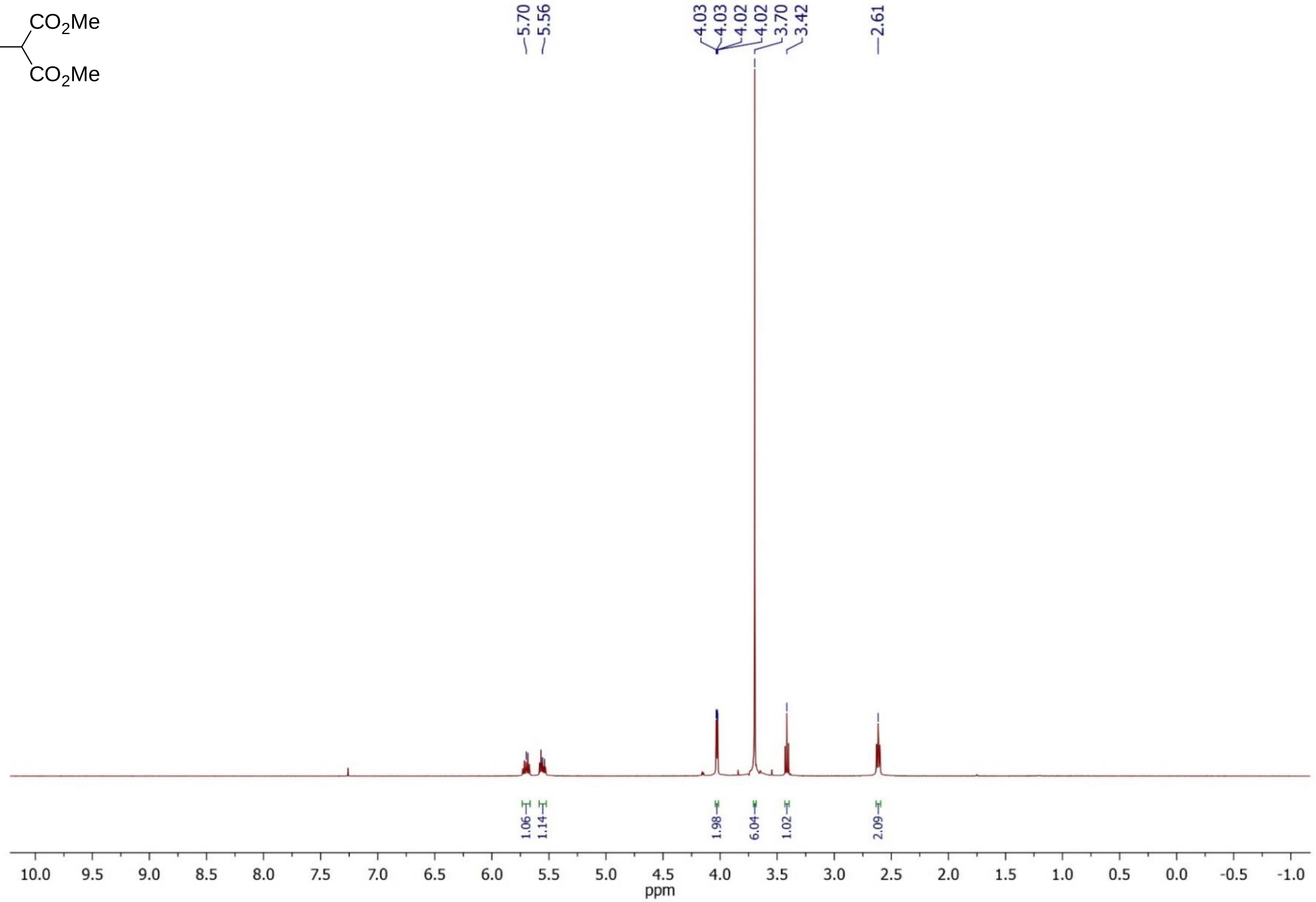

Dimethyl (*E*)-2-(4-isothiocyanatobut-2-en-1-yl)malonate (4)

^{13}C NMR (126 MHz, $CDCl_3$)

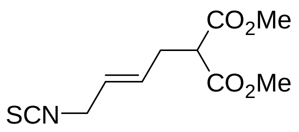

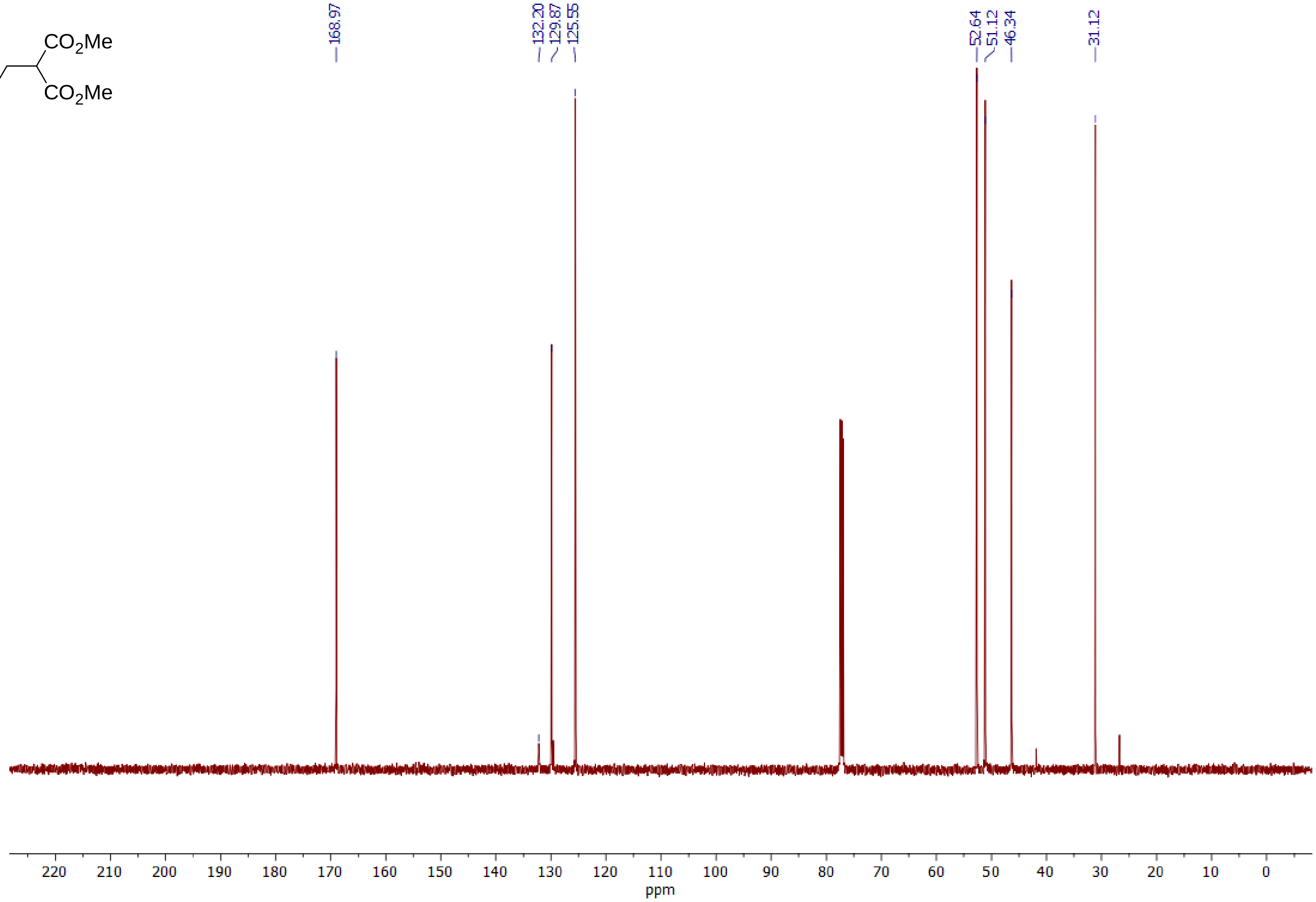

Dimethyl 5-[(*E*)-2-phenylethenyl]-2-thioxopyrrolidine-3,3-dicarboxylate (2z)

^{1}H NMR (500 MHz, $CDCl_3$)

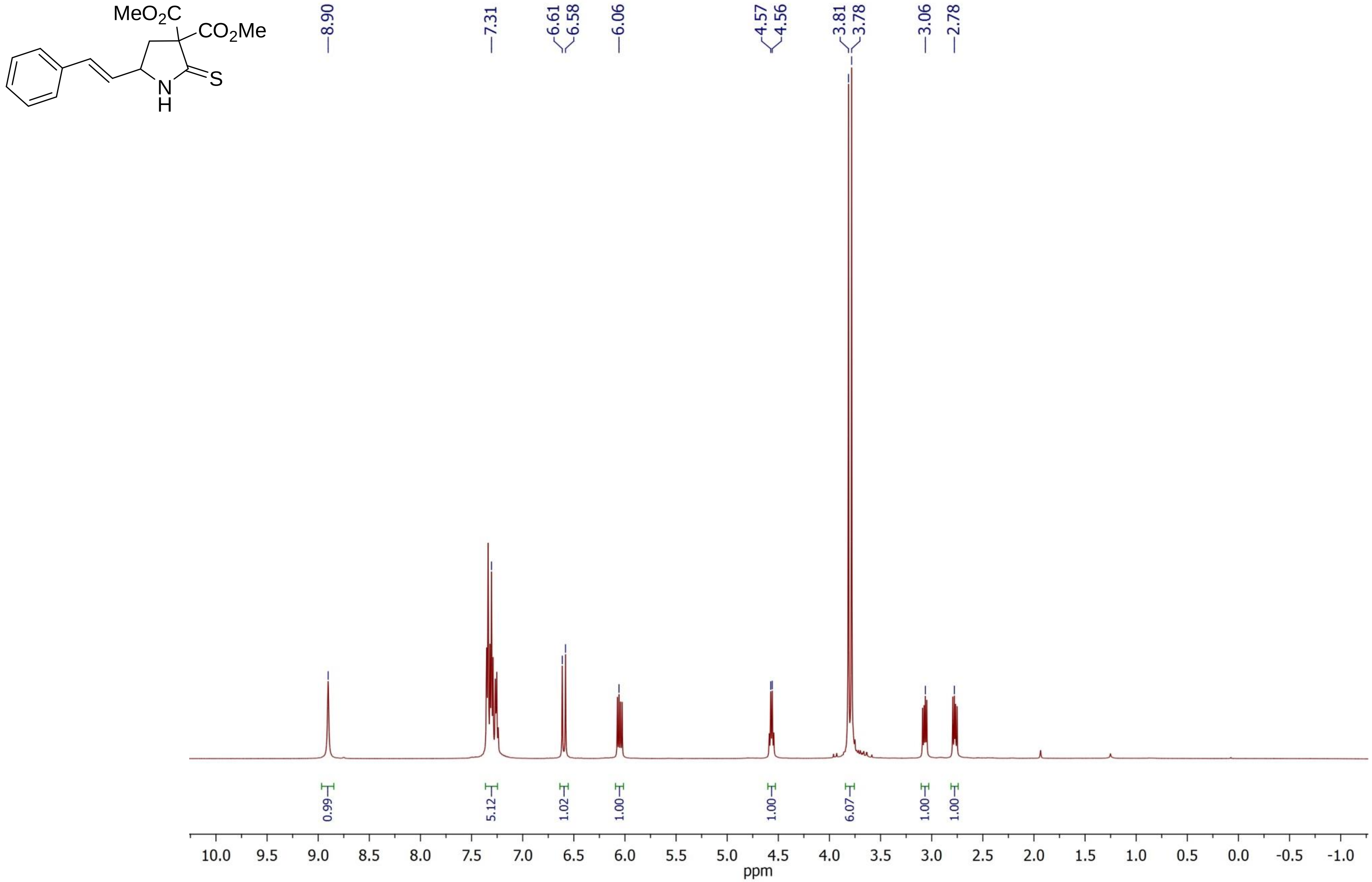

Dimethyl 5-[(*E*)-2-phenylethenyl]-2-thioxopyrrolidine-3,3-dicarboxylate (2z)

^{13}C NMR (126 MHz, $CDCl_3$)

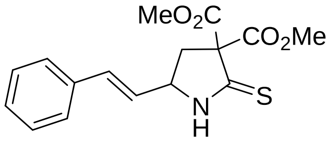

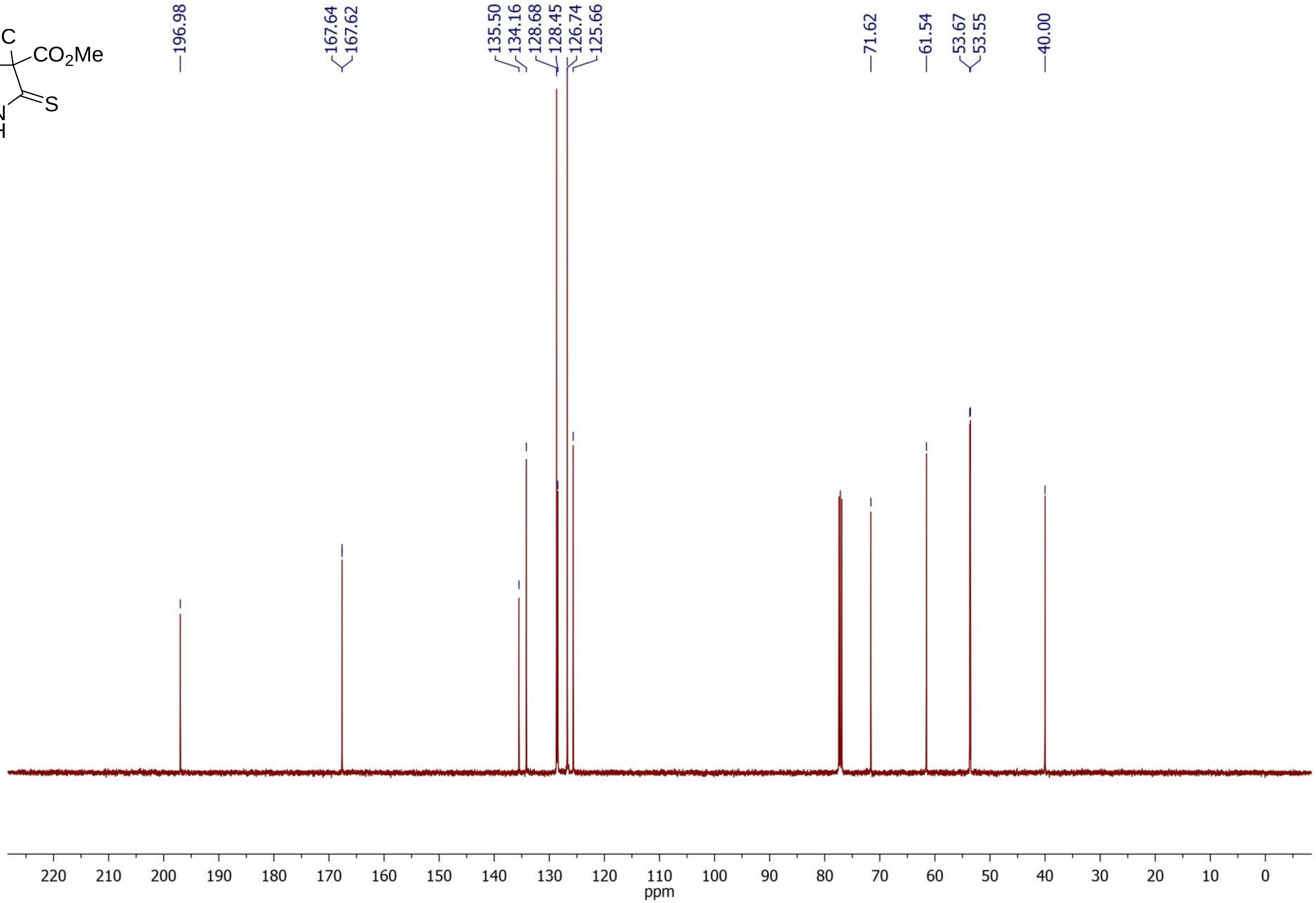

Dimethyl 5-[(*E*)-2-(4-chlorophenyl)ethenyl]-2-thioxopyrrolidine-3,3-dicarboxylate (2aa)

^{1}H NMR (500 MHz, DMSO-d_6)

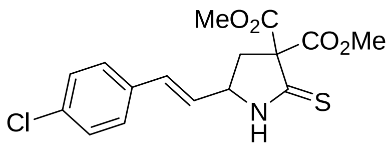

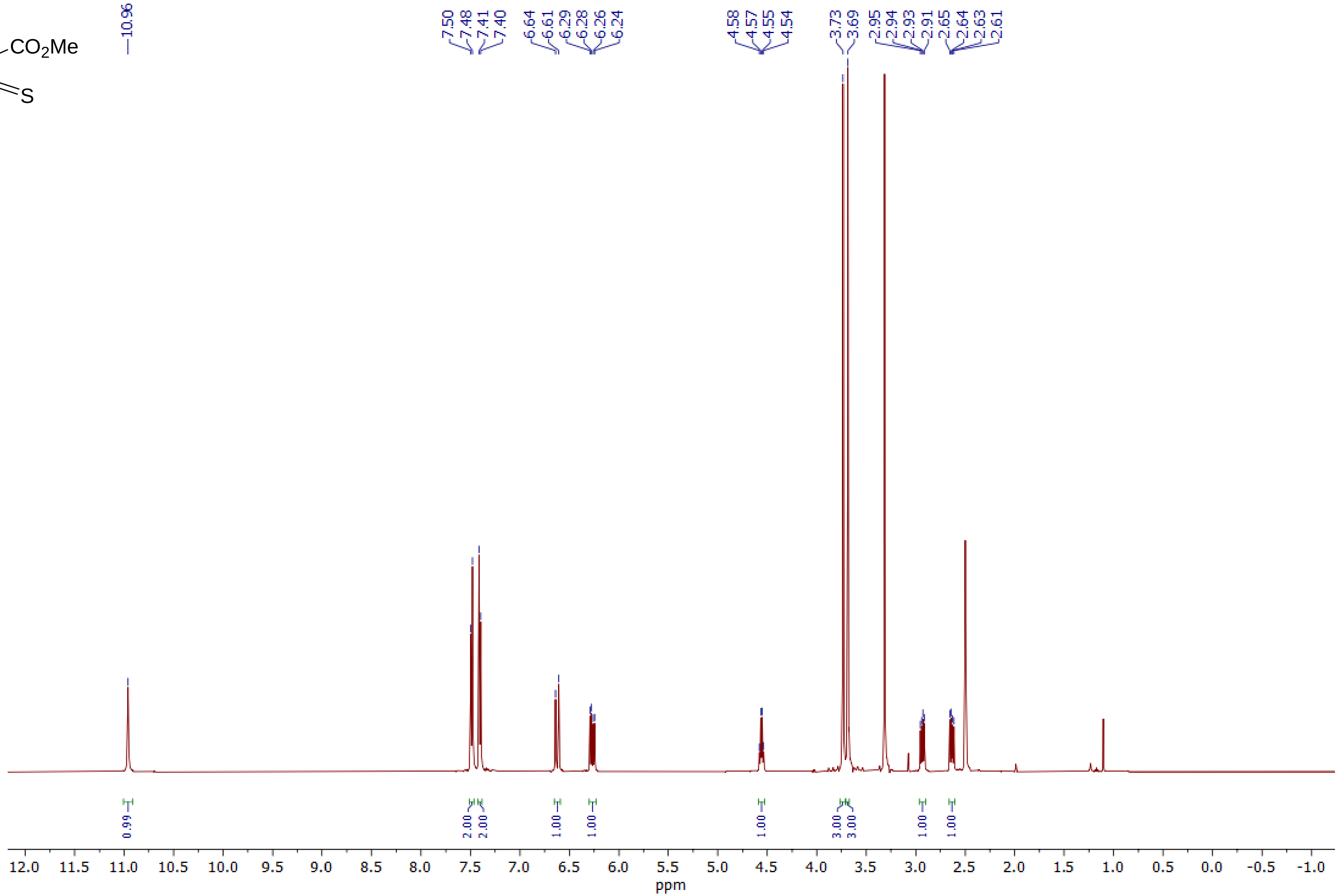

Dimethyl 5-[(*E*)-2-(4-chlorophenyl)ethenyl]-2-thioxopyrrolidine-3,3-dicarboxylate (2aa)

^{13}C NMR (126 MHz, DMSO-d$_6$)

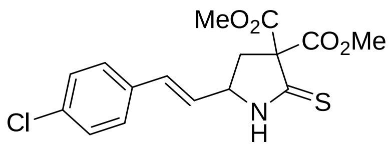

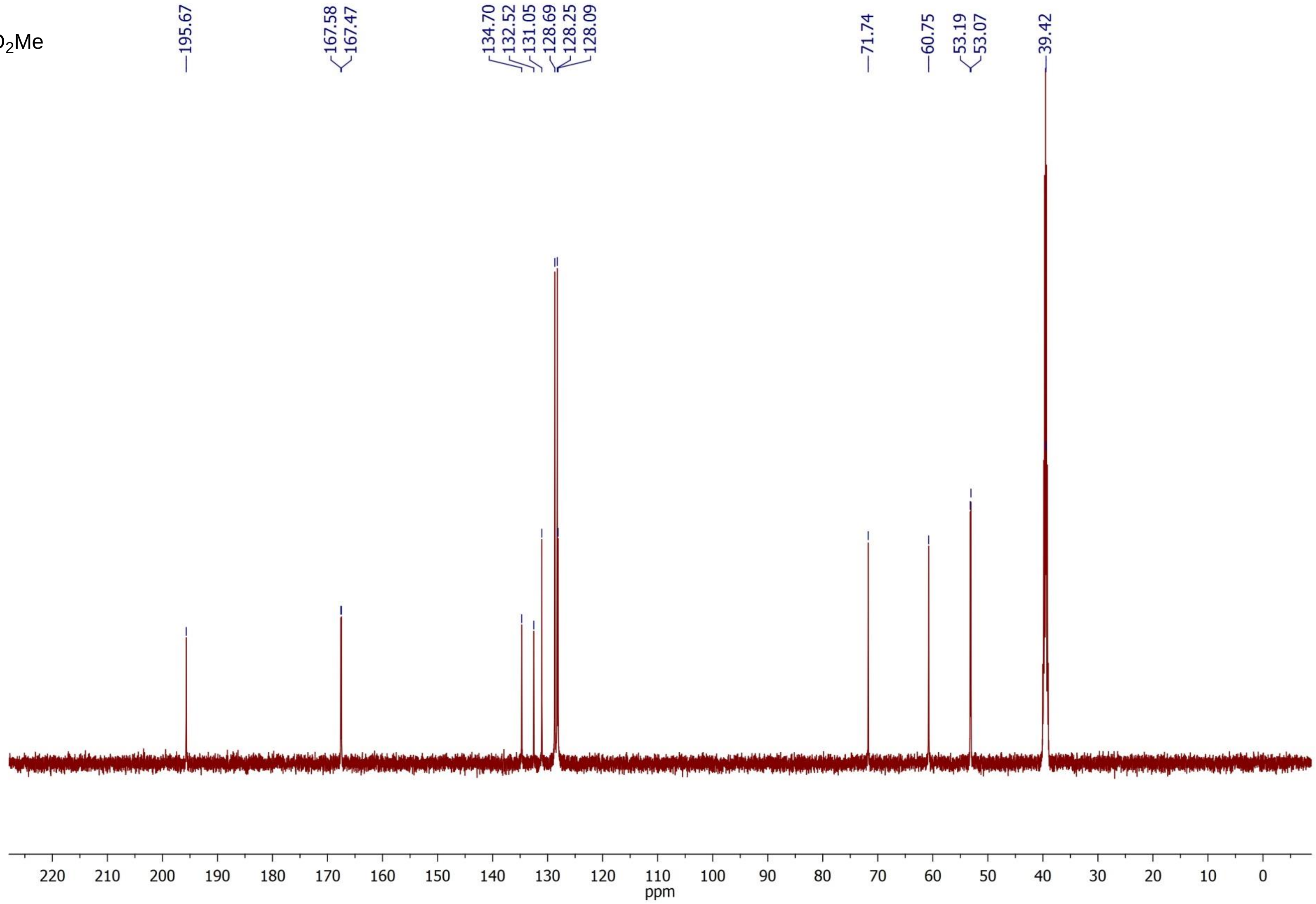

Dimethyl 5-[(*E*)-2-(4-fluorophenyl)ethenyl]-2-thioxopyrrolidine-3,3-dicarboxylate (2ab)

^{1}H NMR (500 MHz, $CDCl_3$)

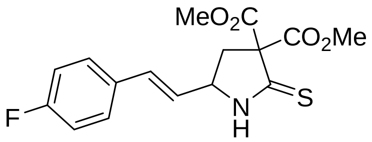

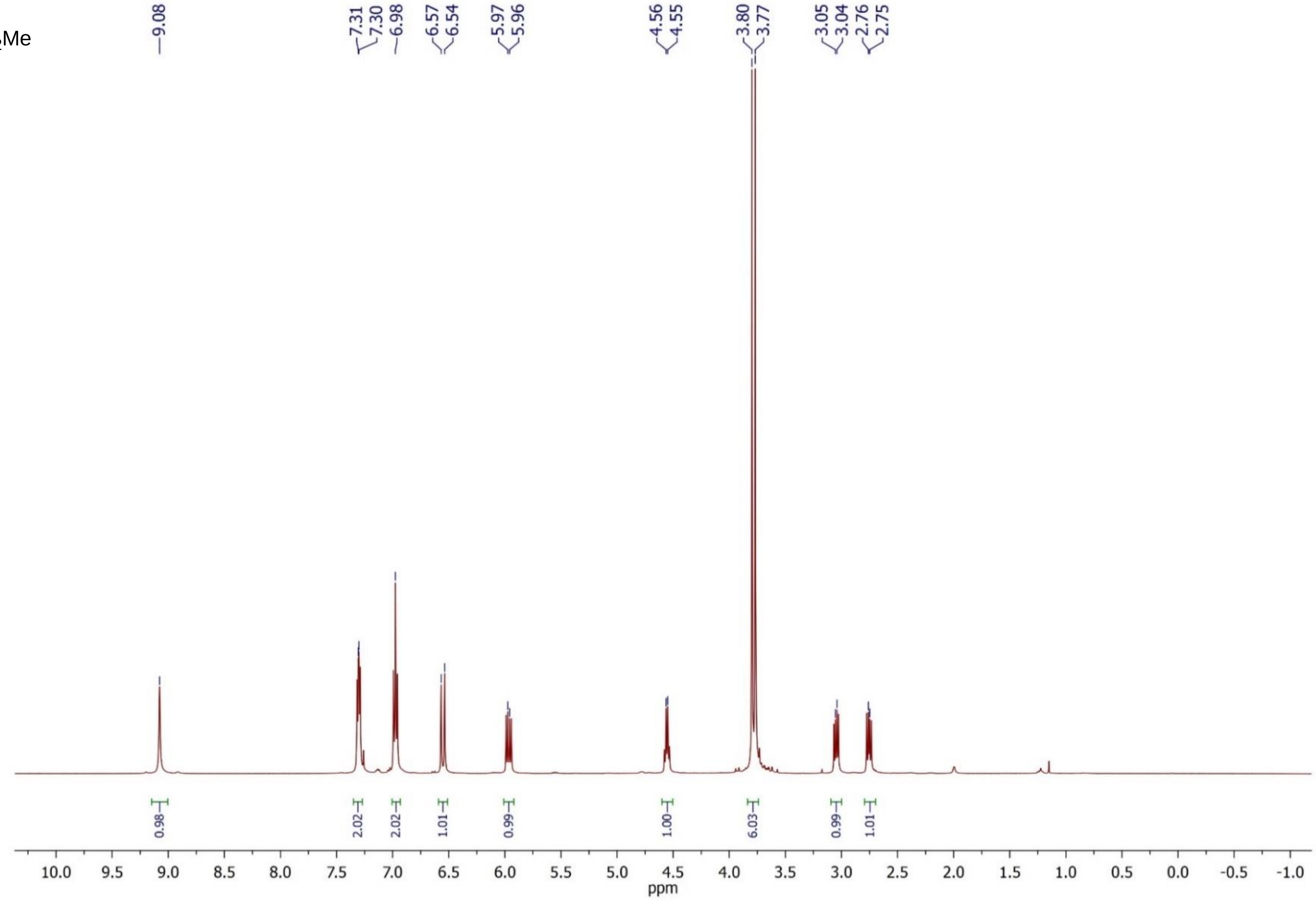

Dimethyl 5-[(*E*)-2-(4-fluorophenyl)ethenyl]-2-thioxopyrrolidine-3,3-dicarboxylate (2ab)

^{13}C NMR (126 MHz, $CDCl_3$)

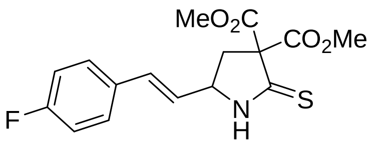

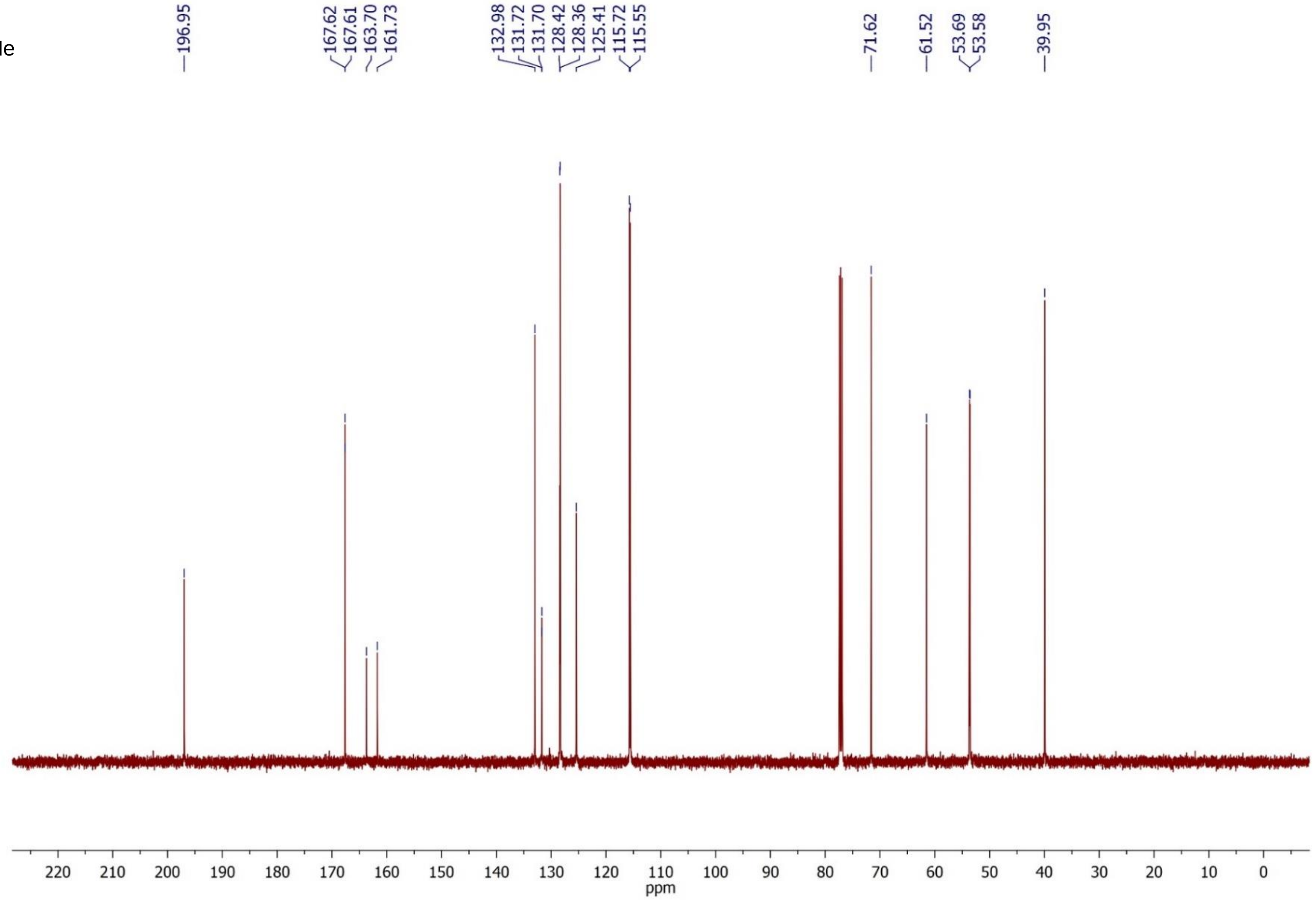

Dimethyl 5-[(*E*)-2-(4-fluorophenyl)ethenyl]-2-thioxopyrrolidine-3,3-dicarboxylate (2ab)

^{19}F NMR (470 MHz, $CDCl_3$)

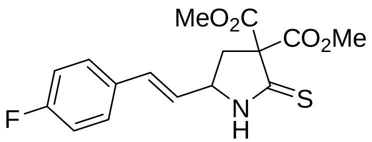

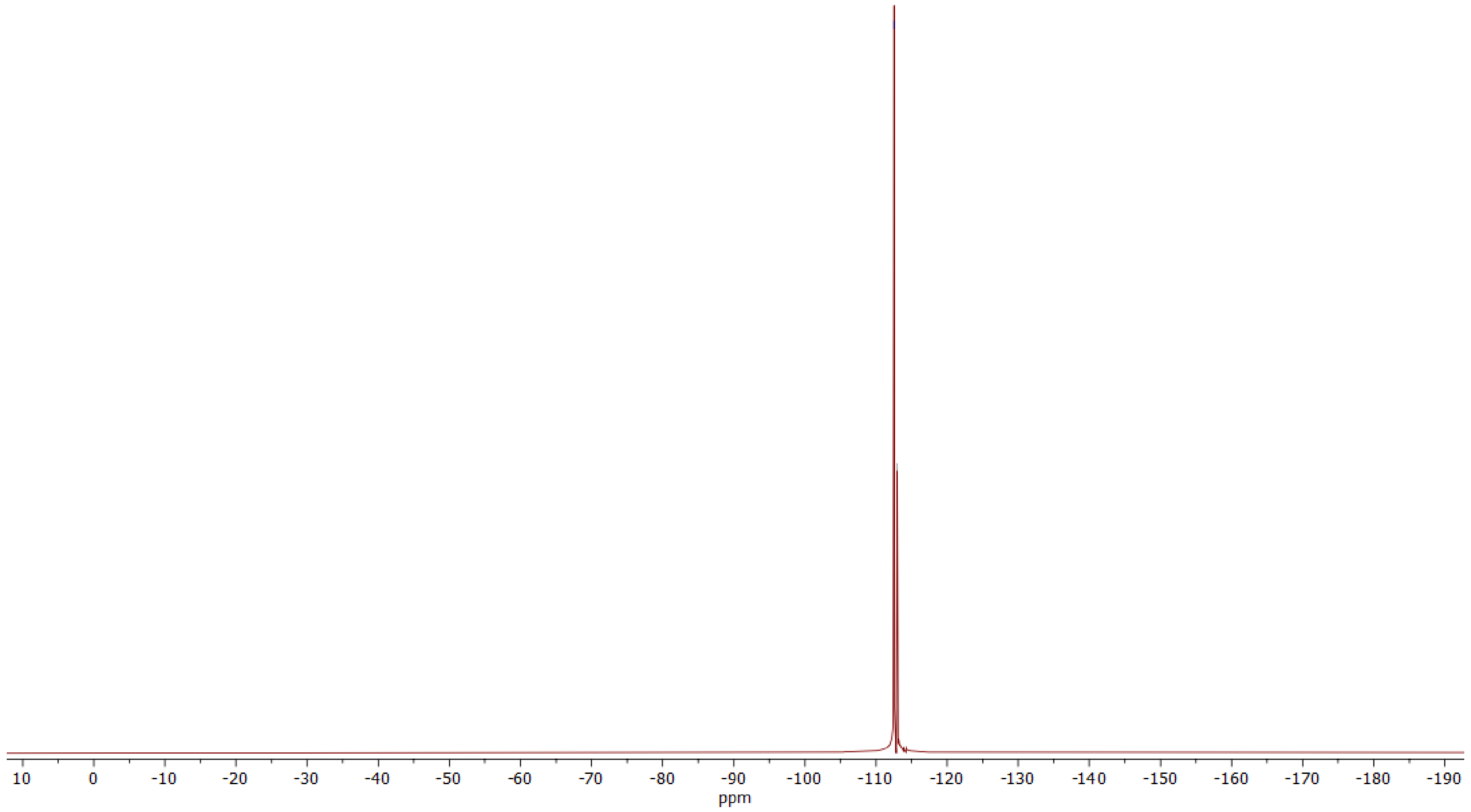

Dimethyl 5-[(*E*)-2-(2-methoxyphenyl)ethenyl]-2-thioxopyrrolidine-3,3-dicarboxylate (2ac)

^{1}H NMR (500 MHz, $CDCl_3$)

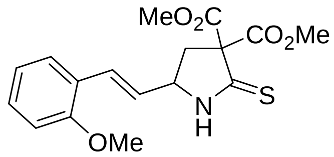

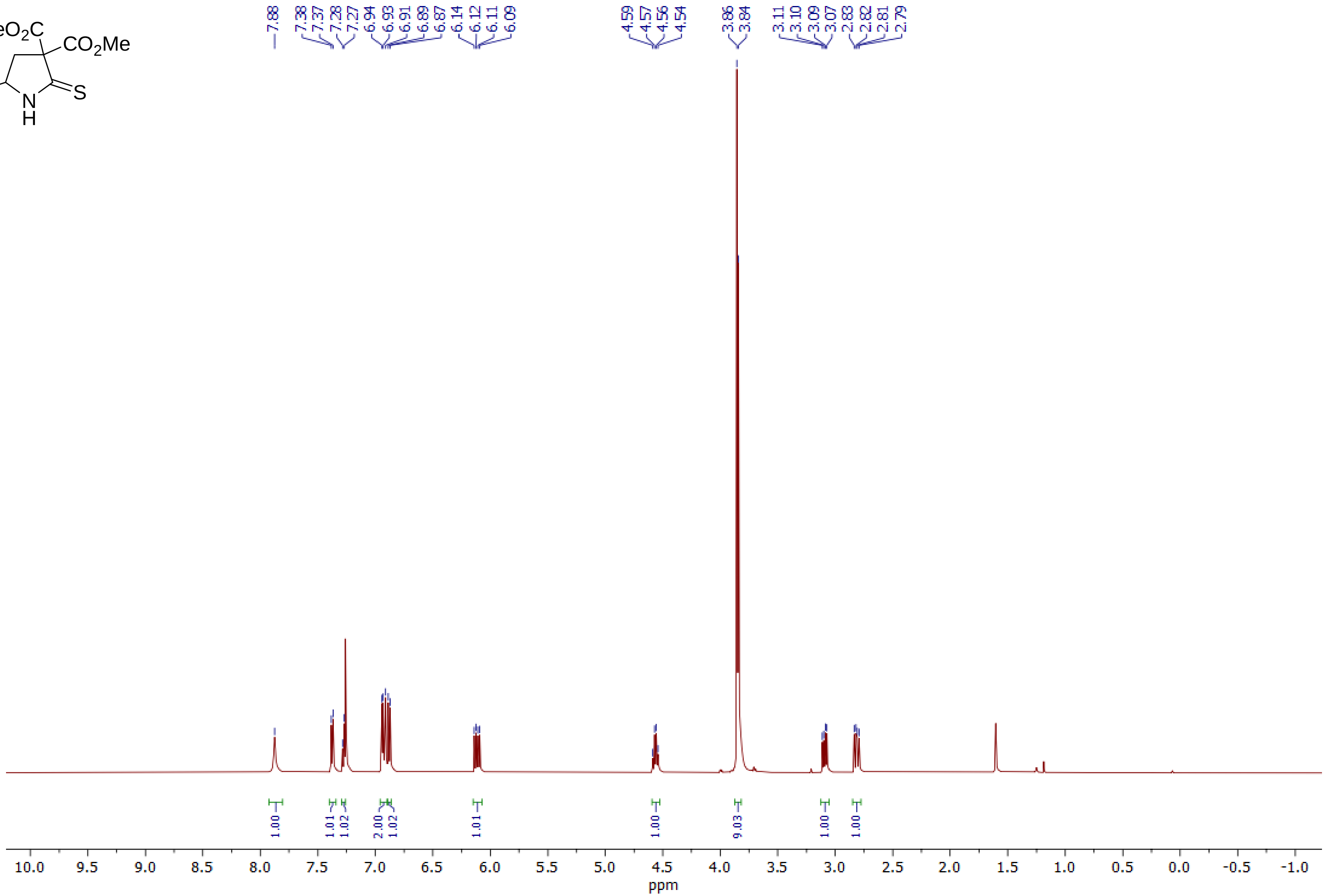

Dimethyl 5-[(*E*)-2-(2-methoxyphenyl)ethenyl]-2-thioxopyrrolidine-3,3-dicarboxylate (2ac)

^{13}C NMR (126 MHz, $CDCl_3$)

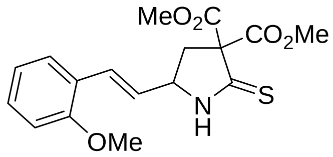

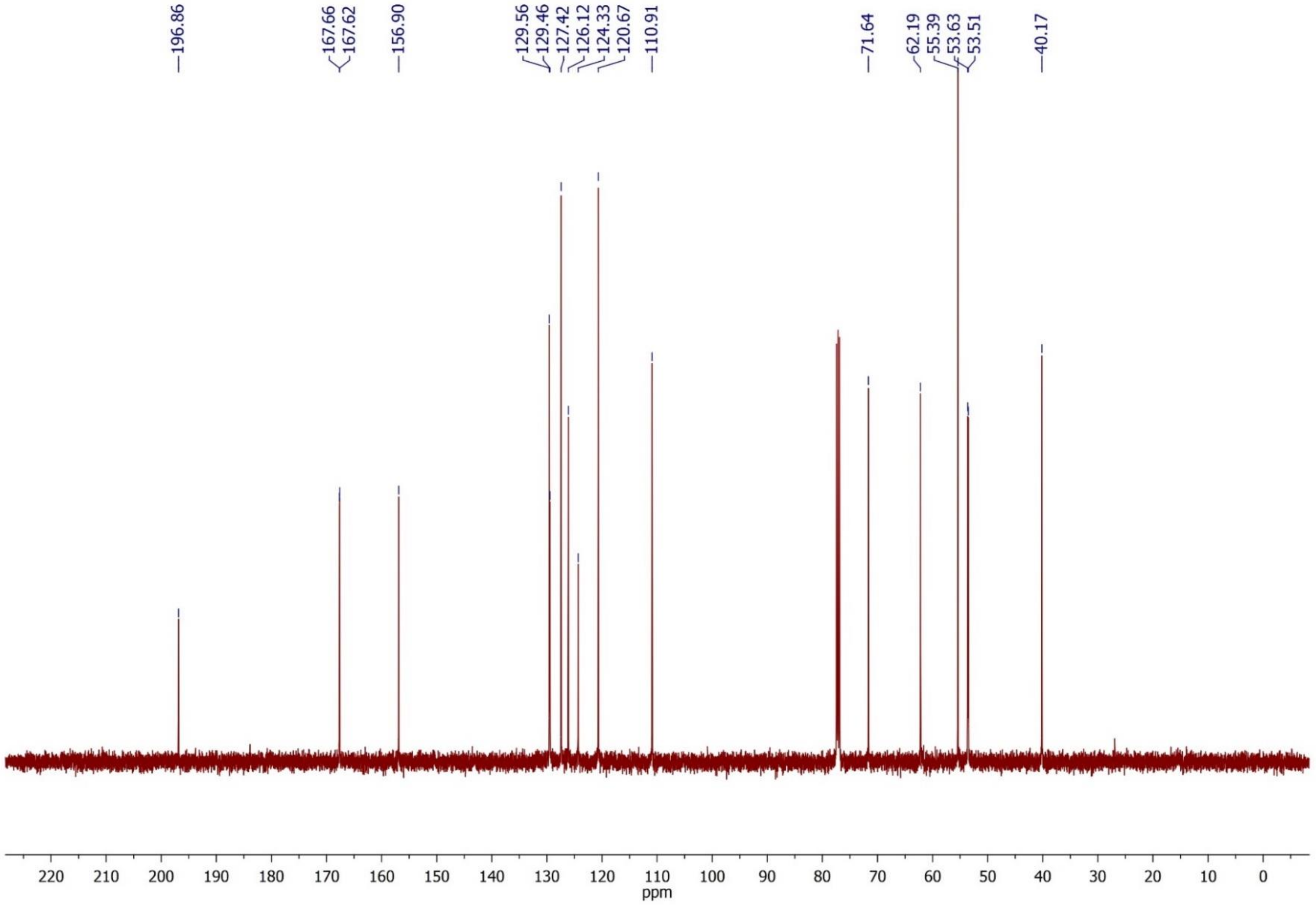

Dimethyl 5-[(*E*)-2-(2-methoxyphenyl)ethenyl]-2-thioxopyrrolidine-3,3-dicarboxylate (2ac)

^{1}H–^{13}C HSQC ($CDCl_3$)

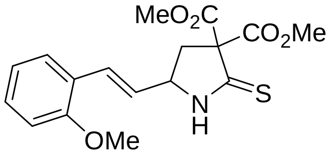

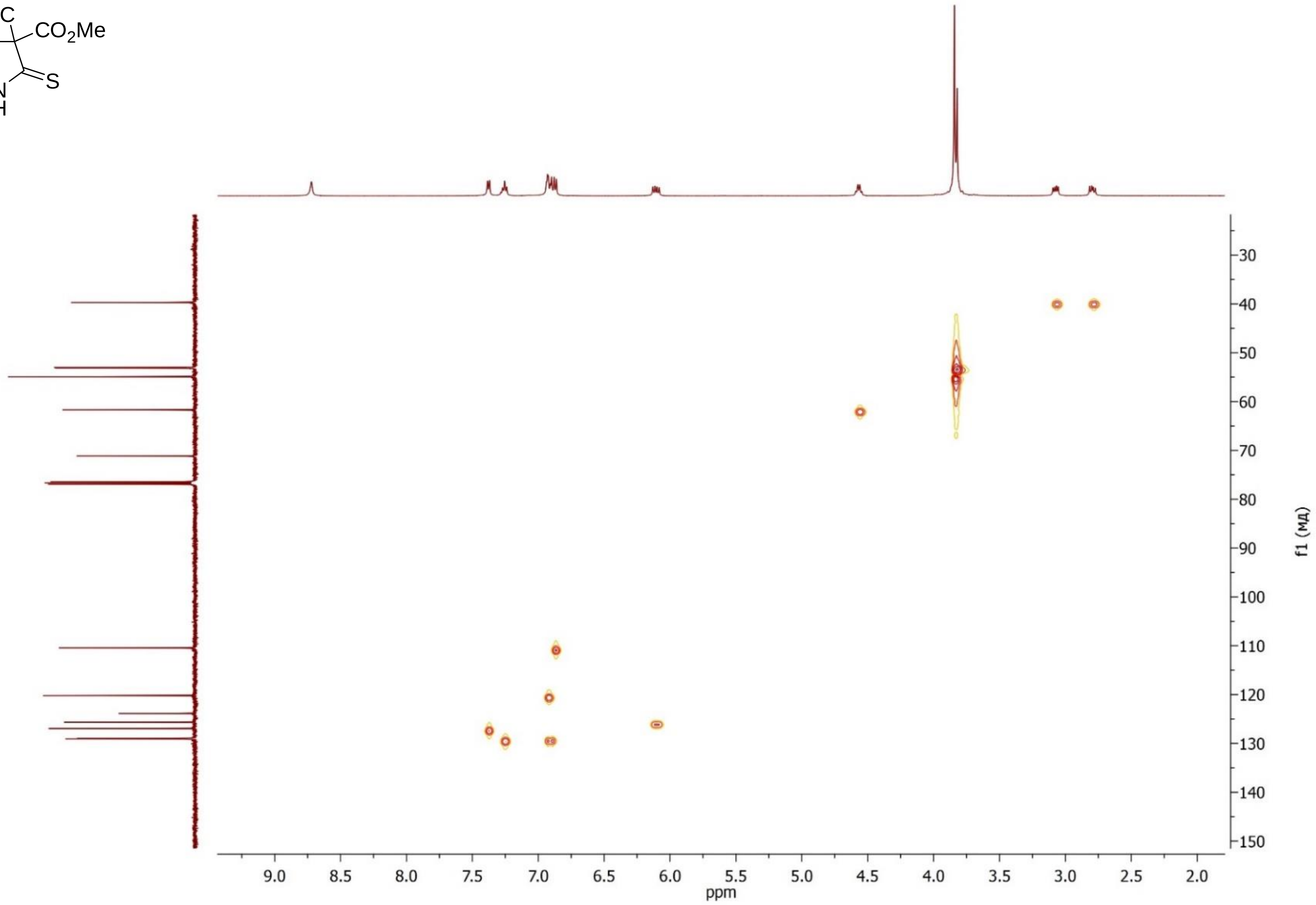

Diethyl 5-(4-methoxyphenyl)-2-thioxopyrrolidine-3,3-dicarboxylate (2ad)

^{1}H NMR (500 MHz, $CDCl_3$)

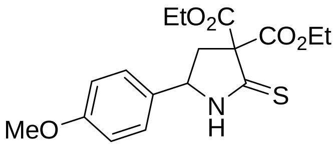

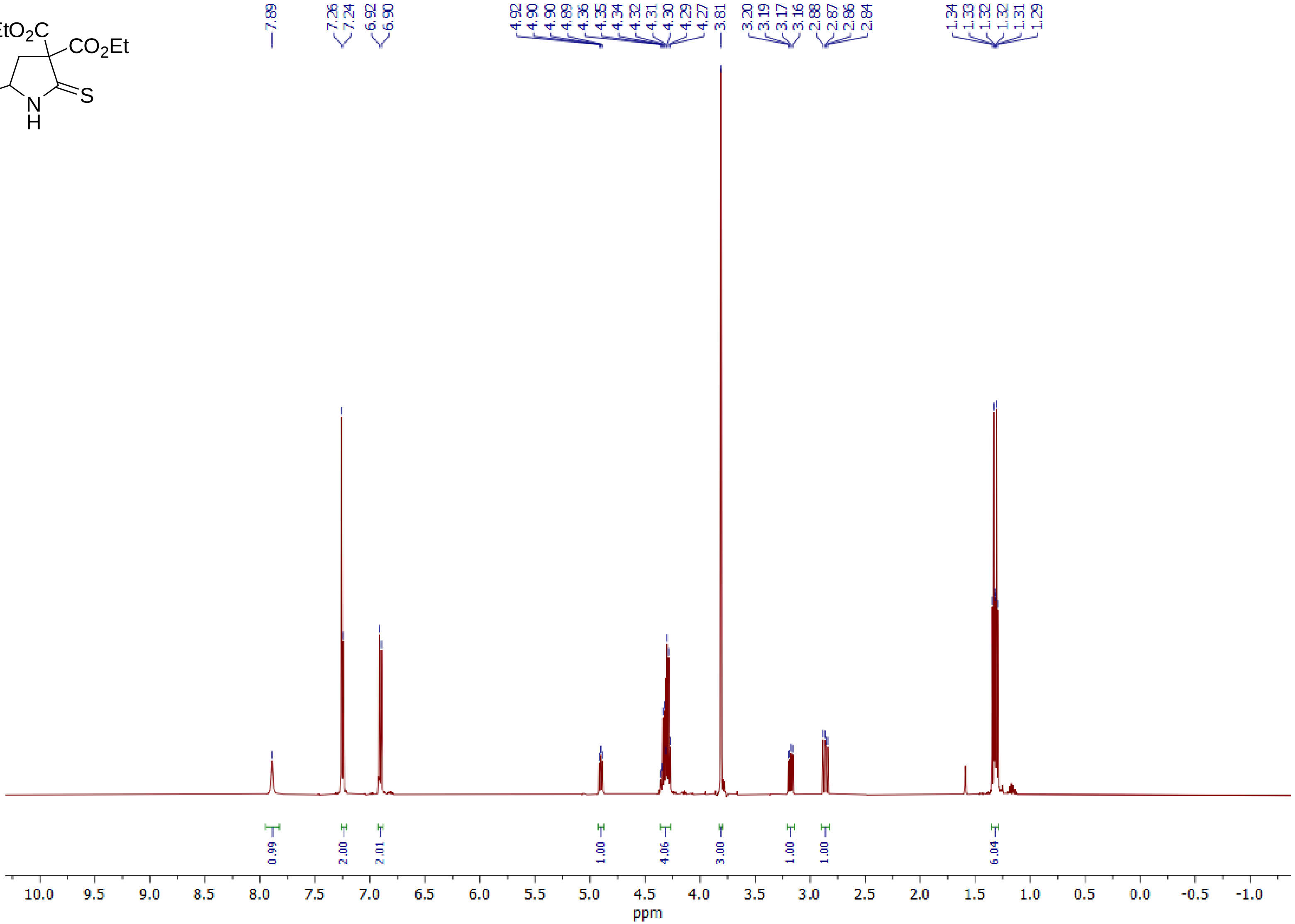

Diethyl 5-(4-methoxyphenyl)-2-thioxopyrrolidine-3,3-dicarboxylate (2ad)

^{13}C NMR (126 MHz, $CDCl_3$)

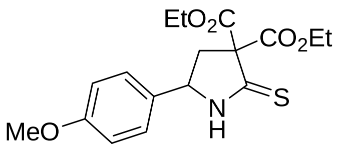

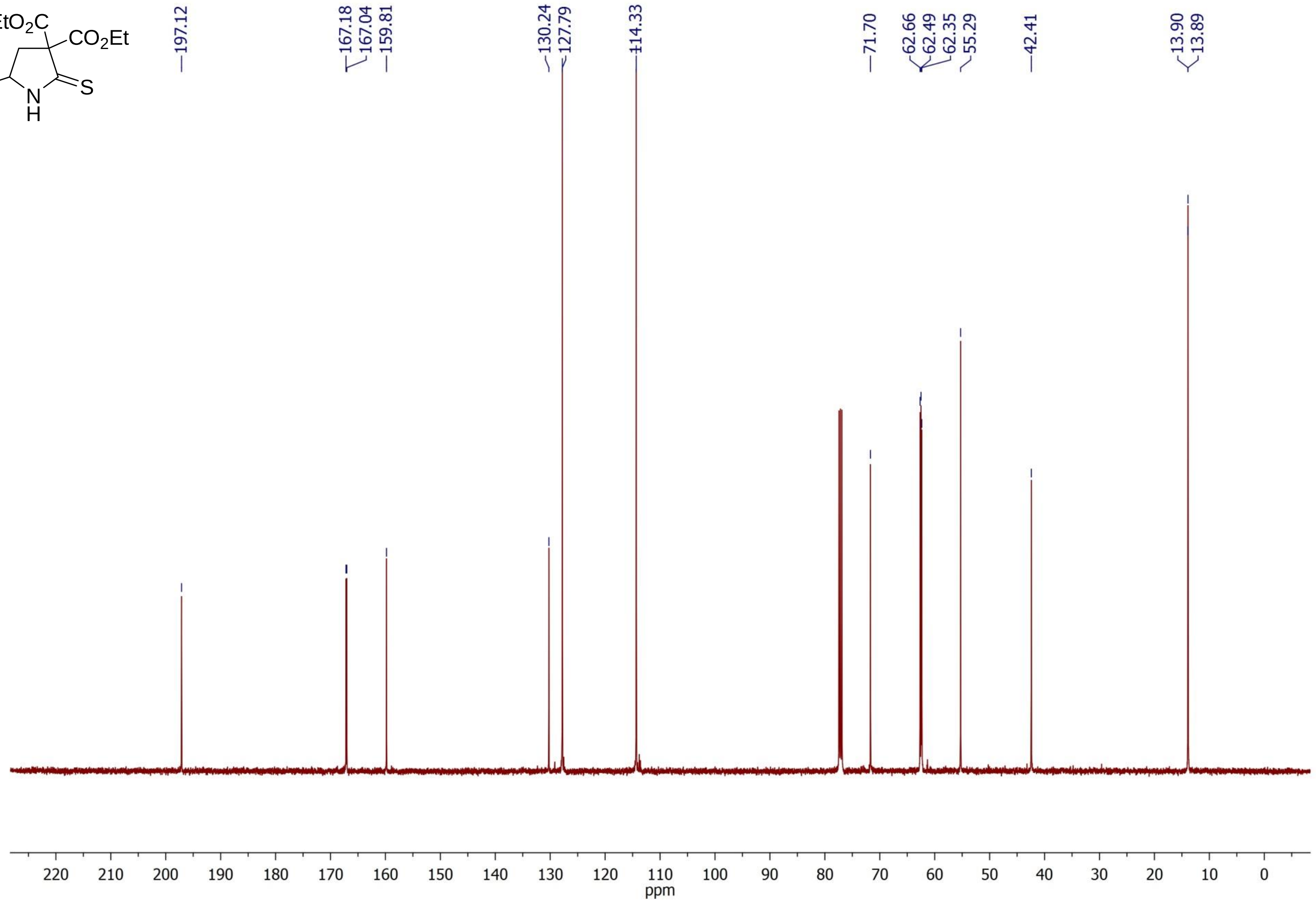

Methyl (3*RS*,5*SR*)-3-cyano-5-(4-methoxyphenyl)-2-thioxopyrrolidine-3-carboxylate (2ae)

^{1}H NMR (500 MHz, acetone-d$_6$)

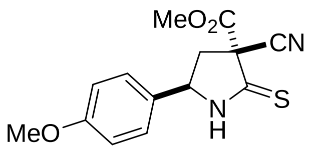

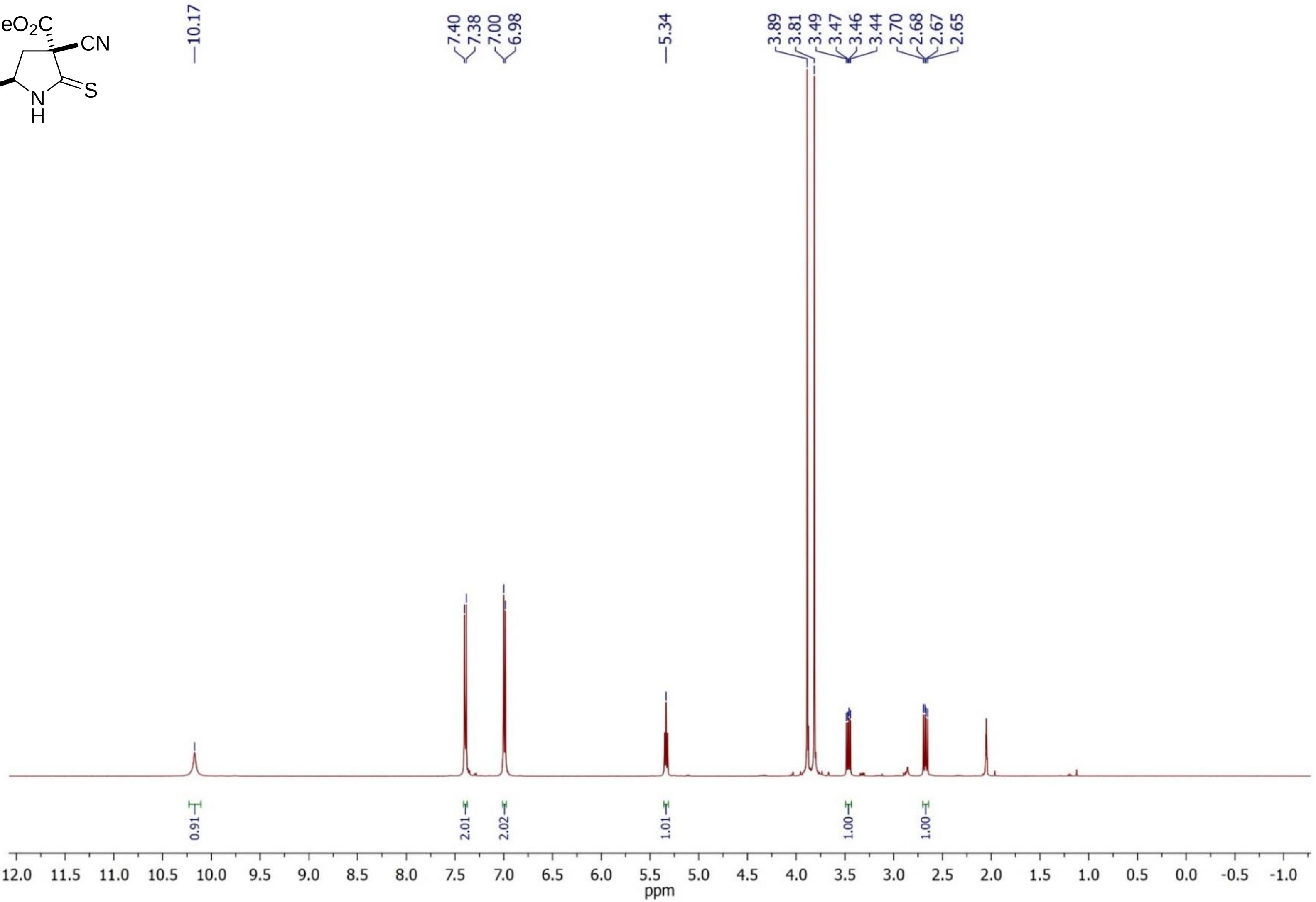

Methyl (3*RS*,5*SR*)-3-cyano-5-(4-methoxyphenyl)-2-thioxopyrrolidine-3-carboxylate (2ae)

^{13}C NMR (126 MHz, acetone-d_6)

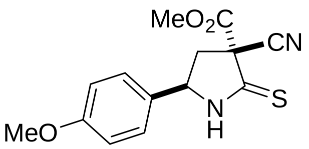

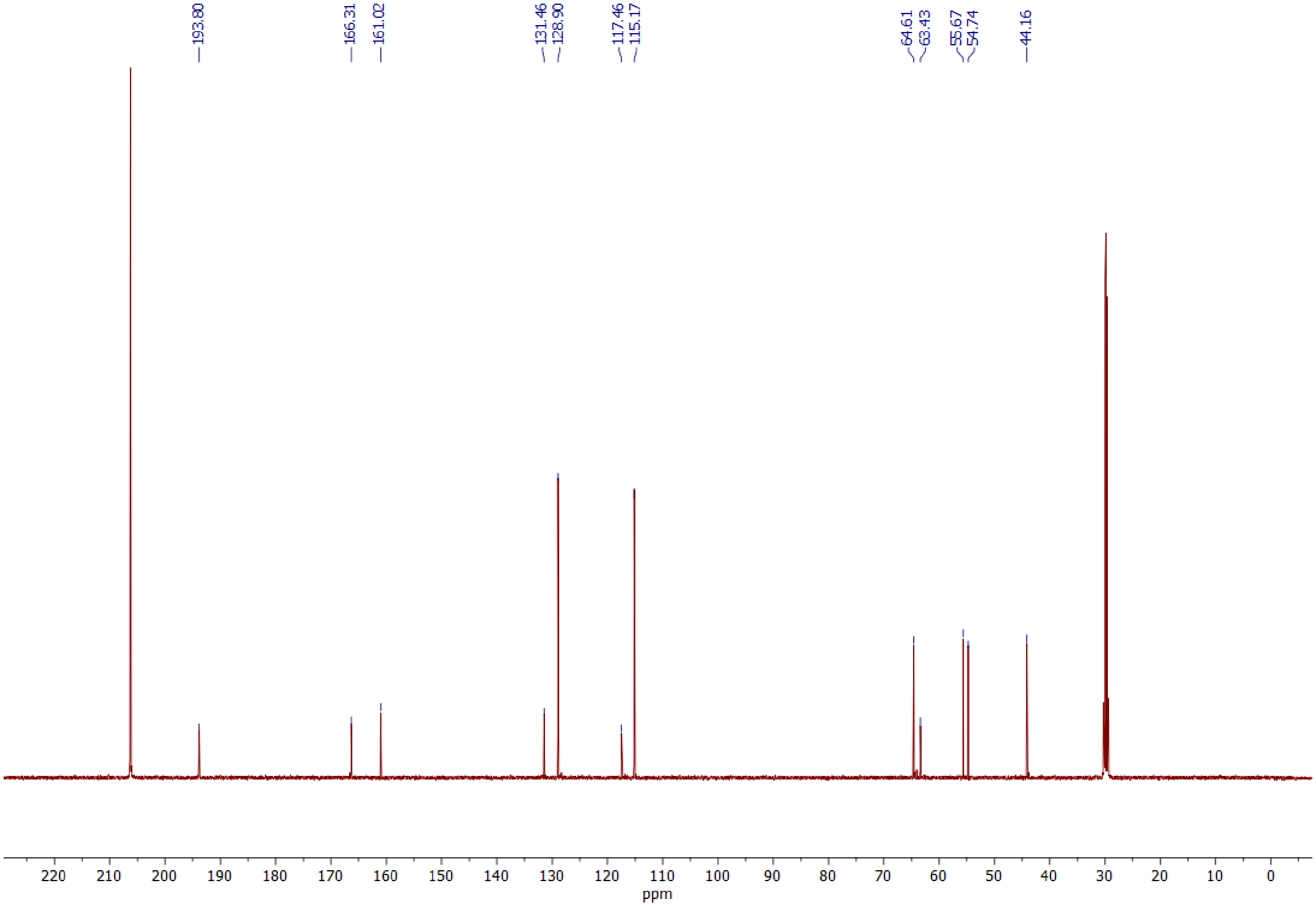

Methyl (3*RS*,5*SR*)-3-cyano-5-(4-methoxyphenyl)-2-thioxopyrrolidine-3-carboxylate (2ae)

^{1}H–^{13}C HSQC (acetone-d_6)

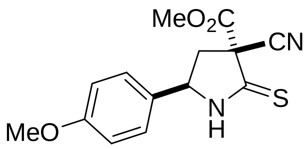

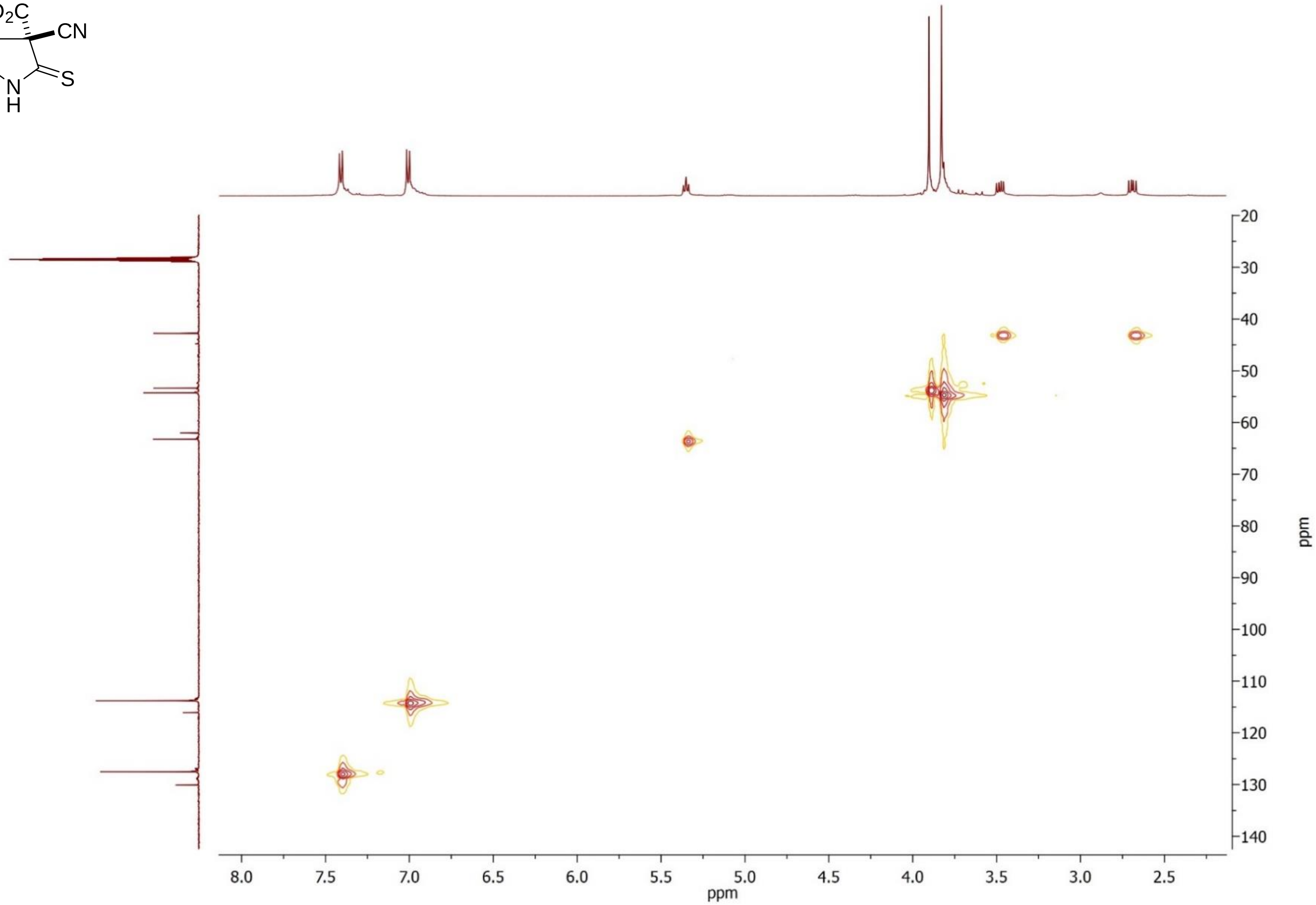

Methyl (3*RS*,5*SR*)-3-cyano-5-(4-methoxyphenyl)-2-thioxopyrrolidine-3-carboxylate (2ae)

^{1}H–^{13}C HMBC (acetone-d_6)

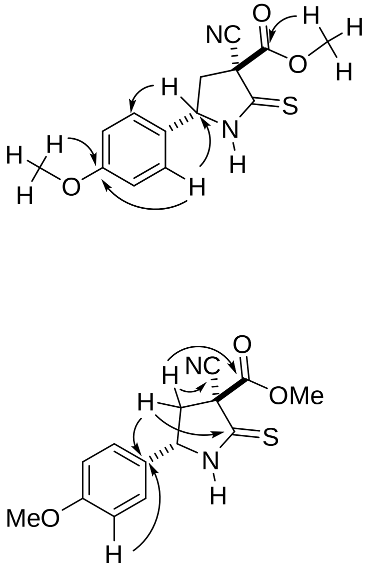

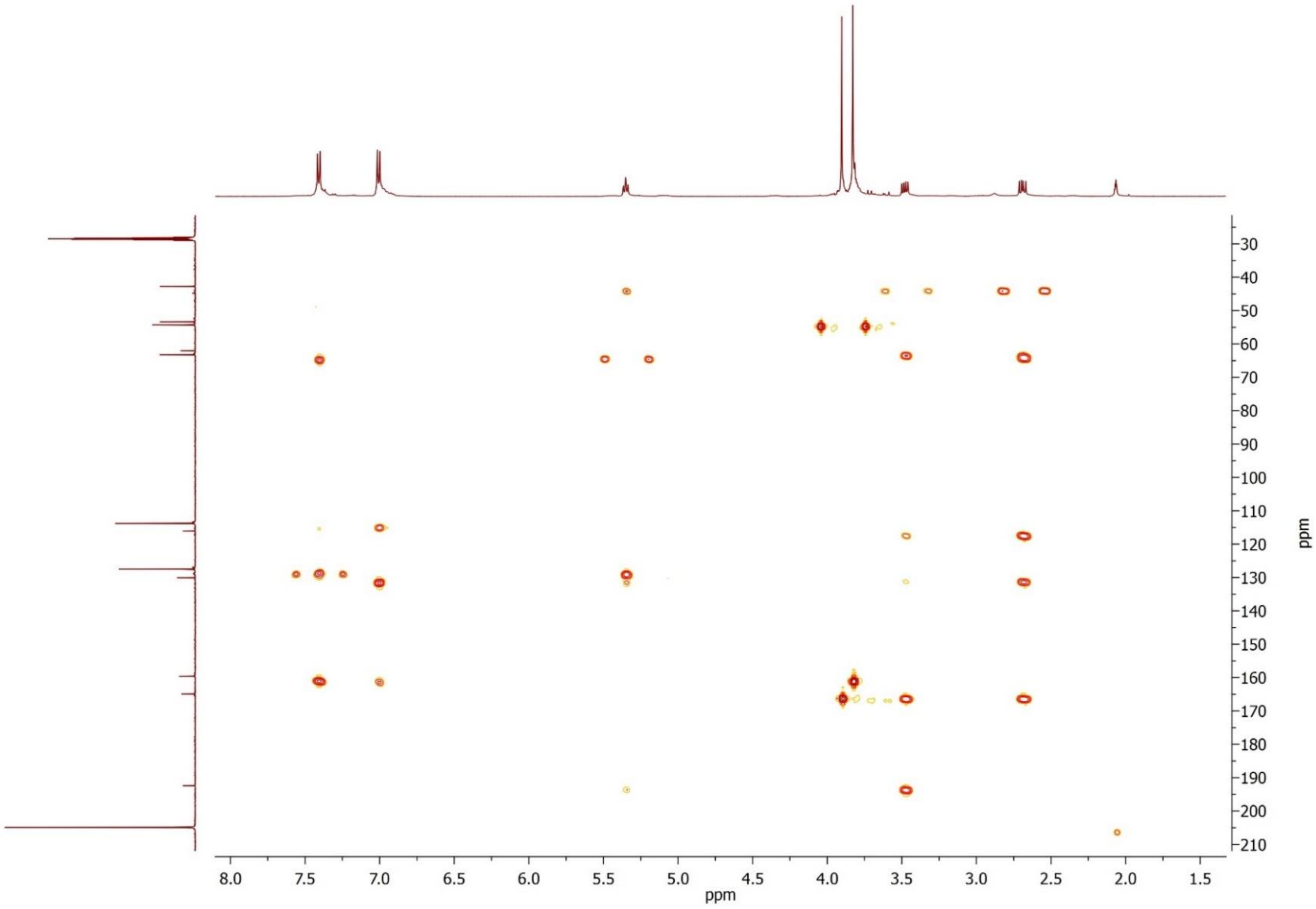

4-(8,8-Dimethyl-6,10-dioxo-1-thioxo-7,9-dioxa-2-azaspiro[4.5]decan-3-yl)benzonitrile (2af)

^{1}H NMR (500 MHz, acetone-d$_6$)

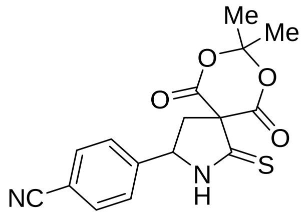

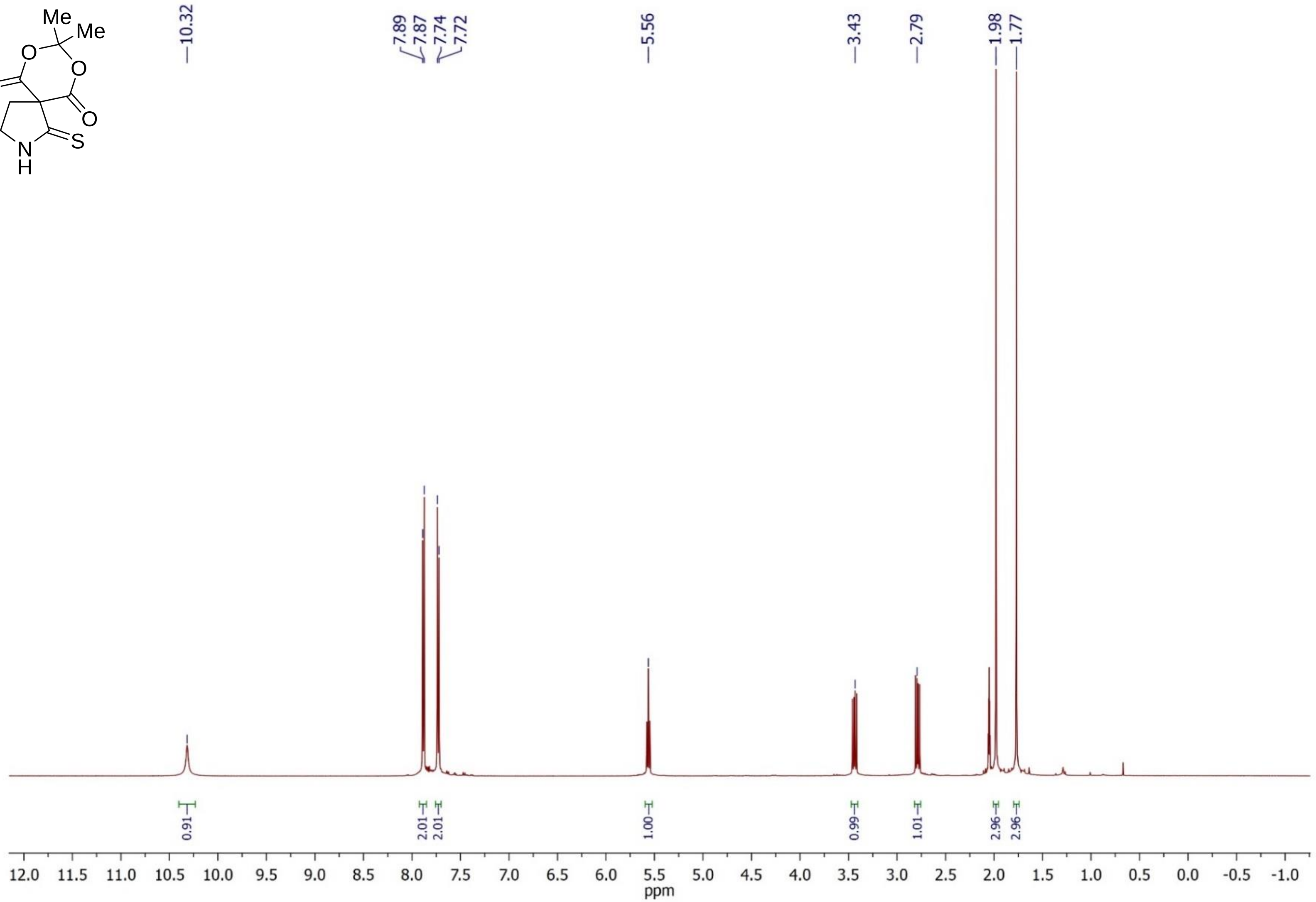

4-(8,8-Dimethyl-6,10-dioxo-1-thioxo-7,9-dioxa-2-azaspiro[4.5]decan-3-yl)benzonitrile (2af)

^{13}C NMR (126 MHz, acetone-d_6)

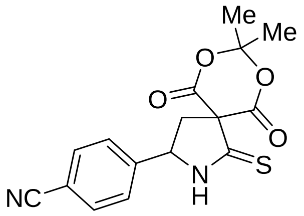

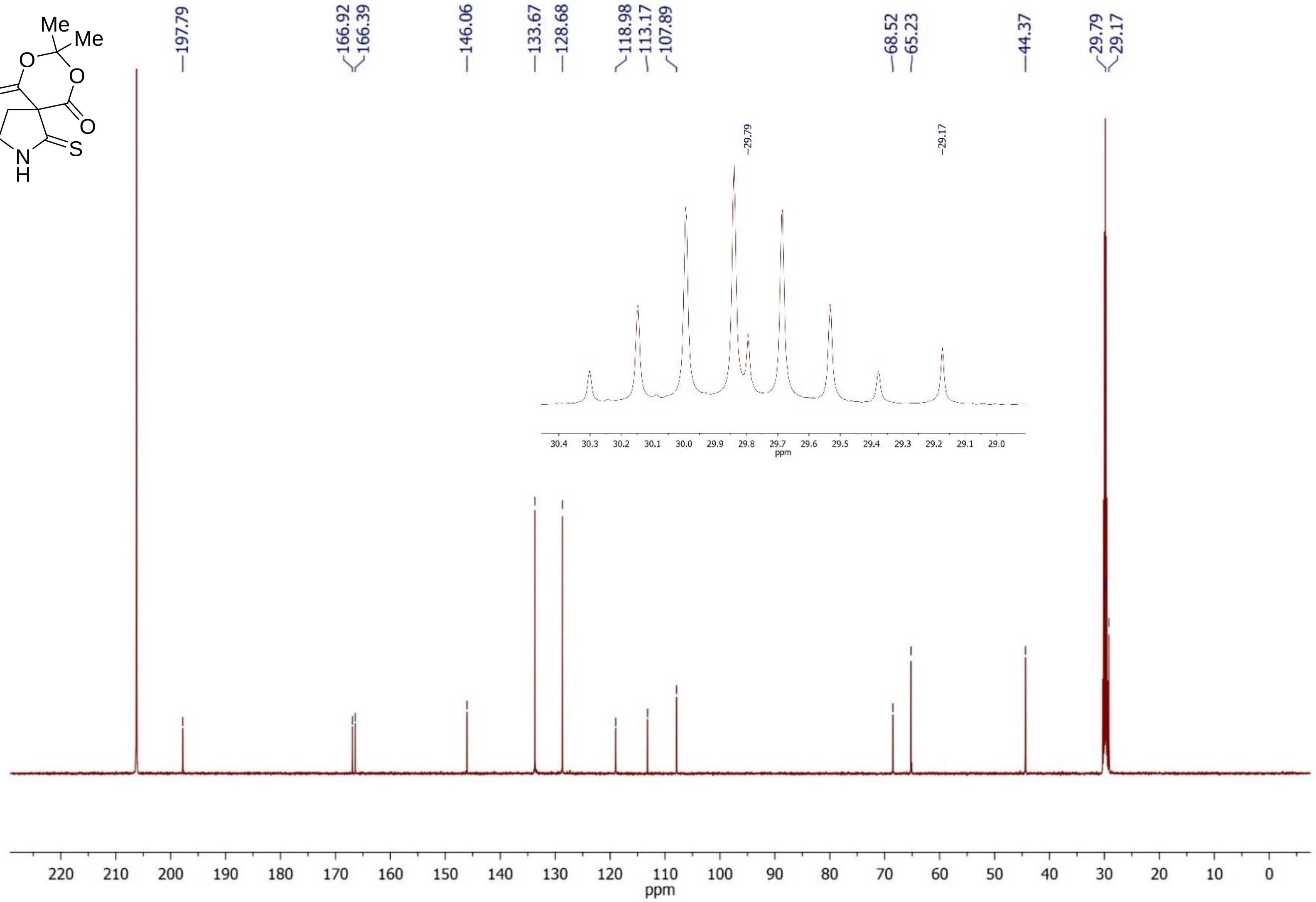

4-(8,8-Dimethyl-6,10-dioxo-1-thioxo-7,9-dioxa-2-azaspiro[4.5]decan-3-yl)benzonitrile (2af)

^{1}H–^{13}C HSQC (acetone-d_6)

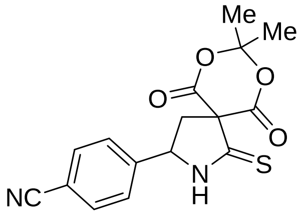

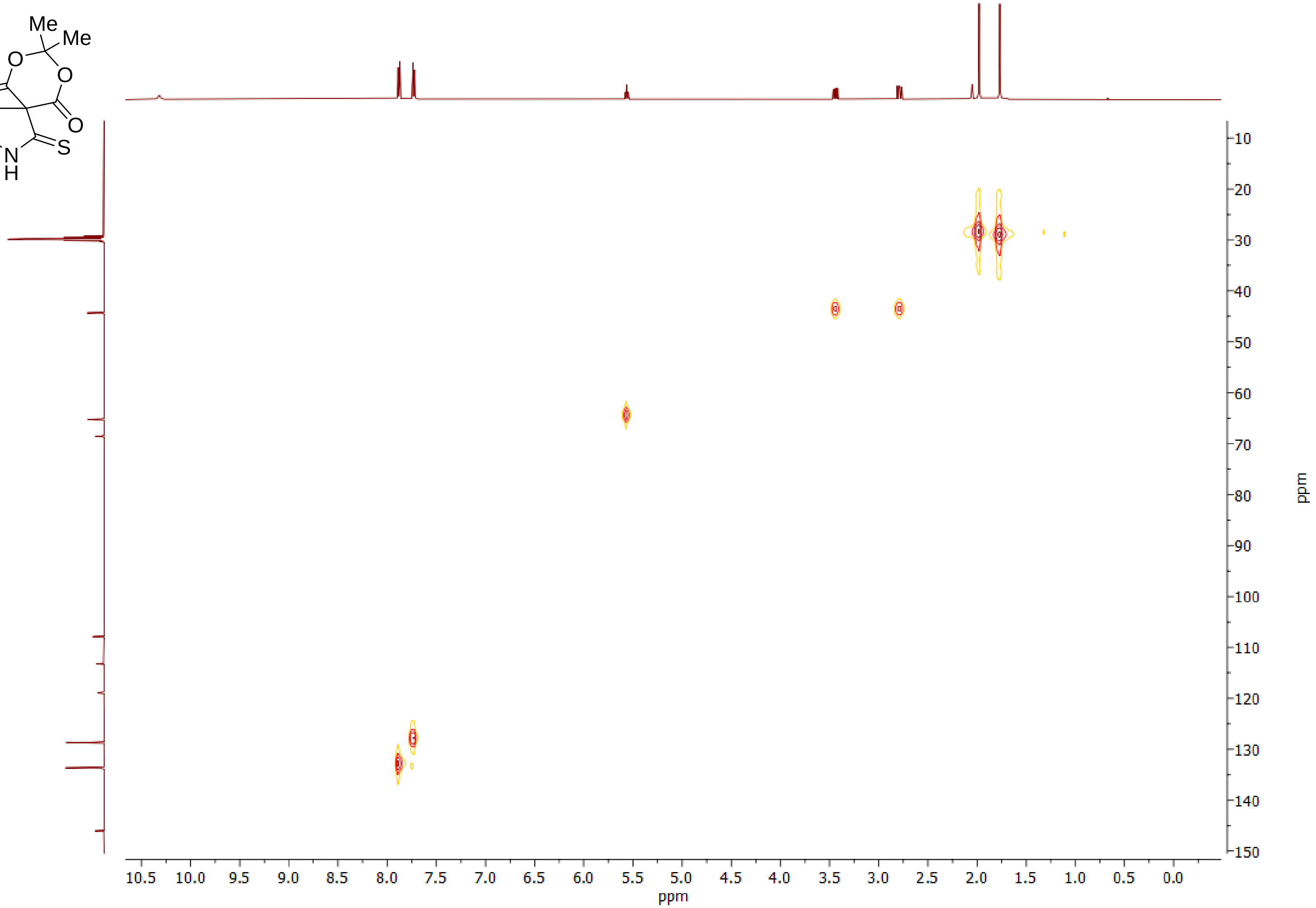

4-(8,8-Dimethyl-6,10-dioxo-1-thioxo-7,9-dioxa-2-azaspiro[4.5]decan-3-yl)benzonitrile (2af)

^1H–^{13}C HMBC (acetone-d_6)

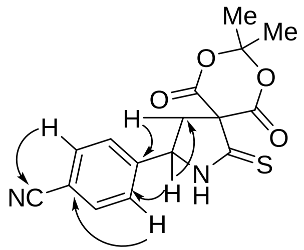

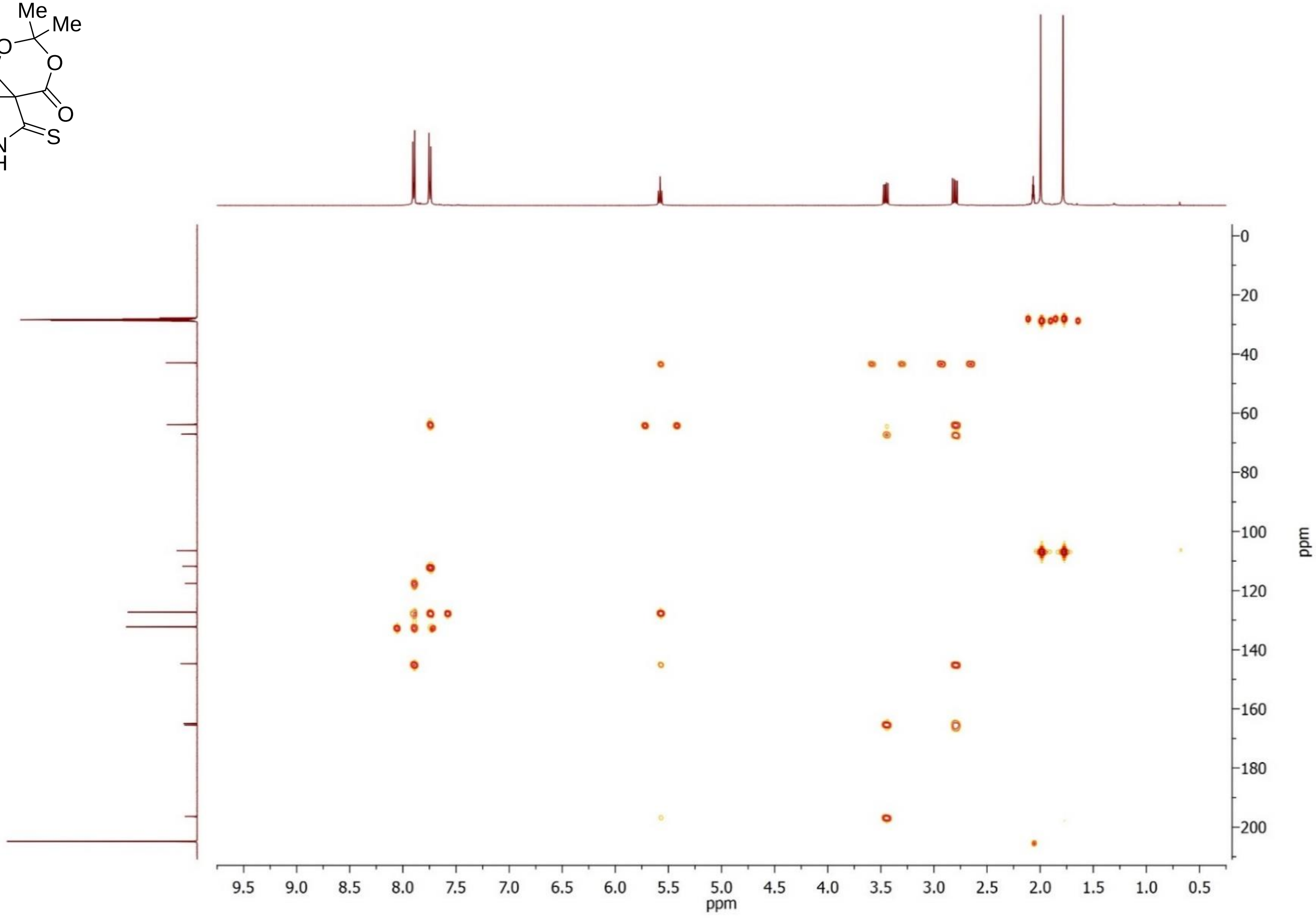

8,8-dimethyl-3-phenyl-1-thioxo-7,9-dioxa-2-azaspiro[4.5]decane-6,10-dione (2ag)

^{1}H NMR (500 MHz, acetone-d_6)

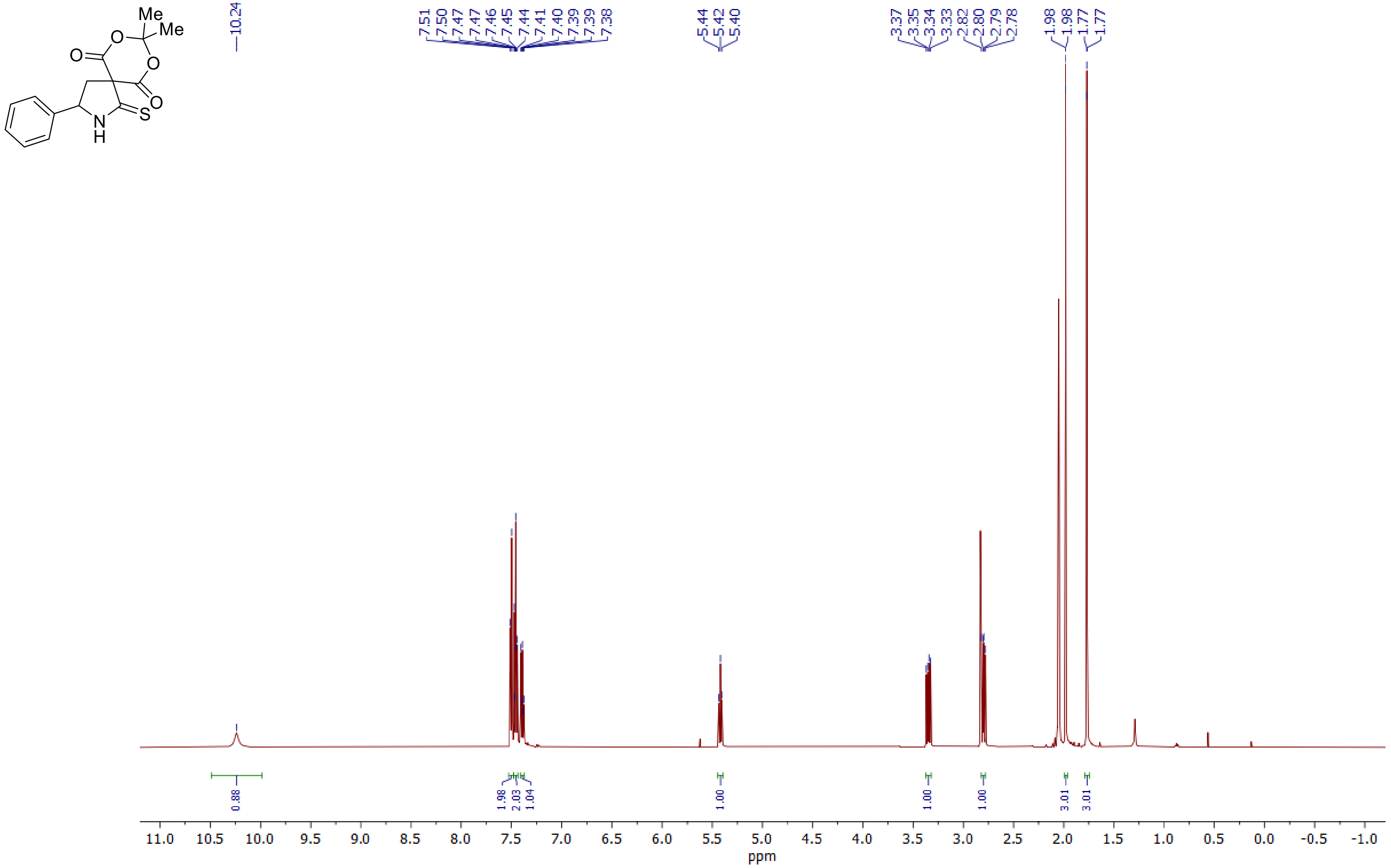

8,8-dimethyl-3-phenyl-1-thioxo-7,9-dioxa-2-azaspiro[4.5]decane-6,10-dione (2ag)

^{13}C NMR (126 MHz, acetone-d_6)

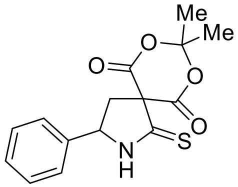

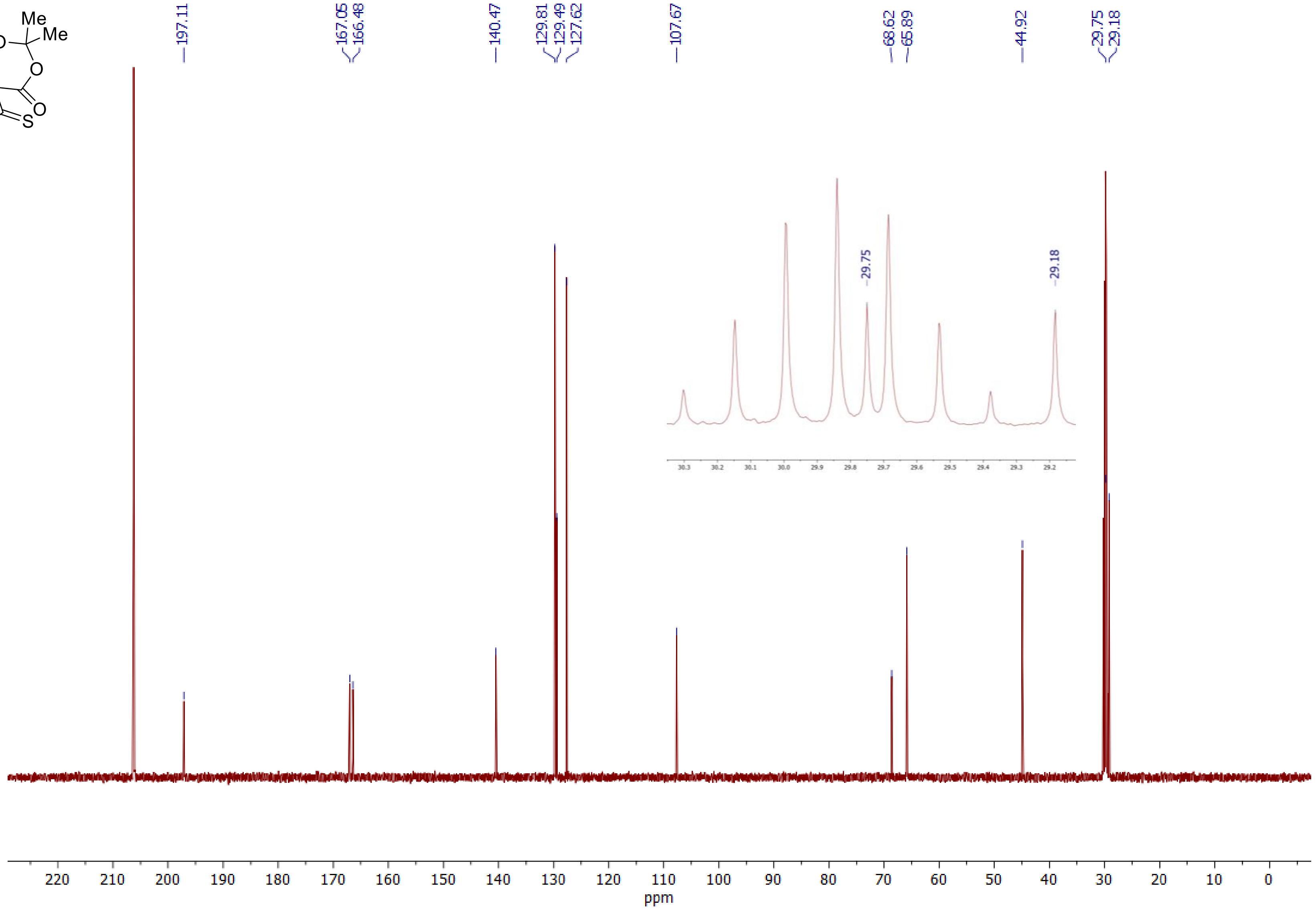

8,8-dimethyl-3-phenyl-1-thioxo-7,9-dioxa-2-azaspiro[4.5]decane-6,10-dione (2ag)

^{1}H–^{13}C HSQC (acetone-d_6)

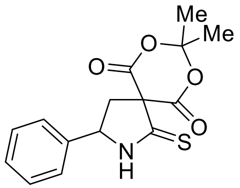

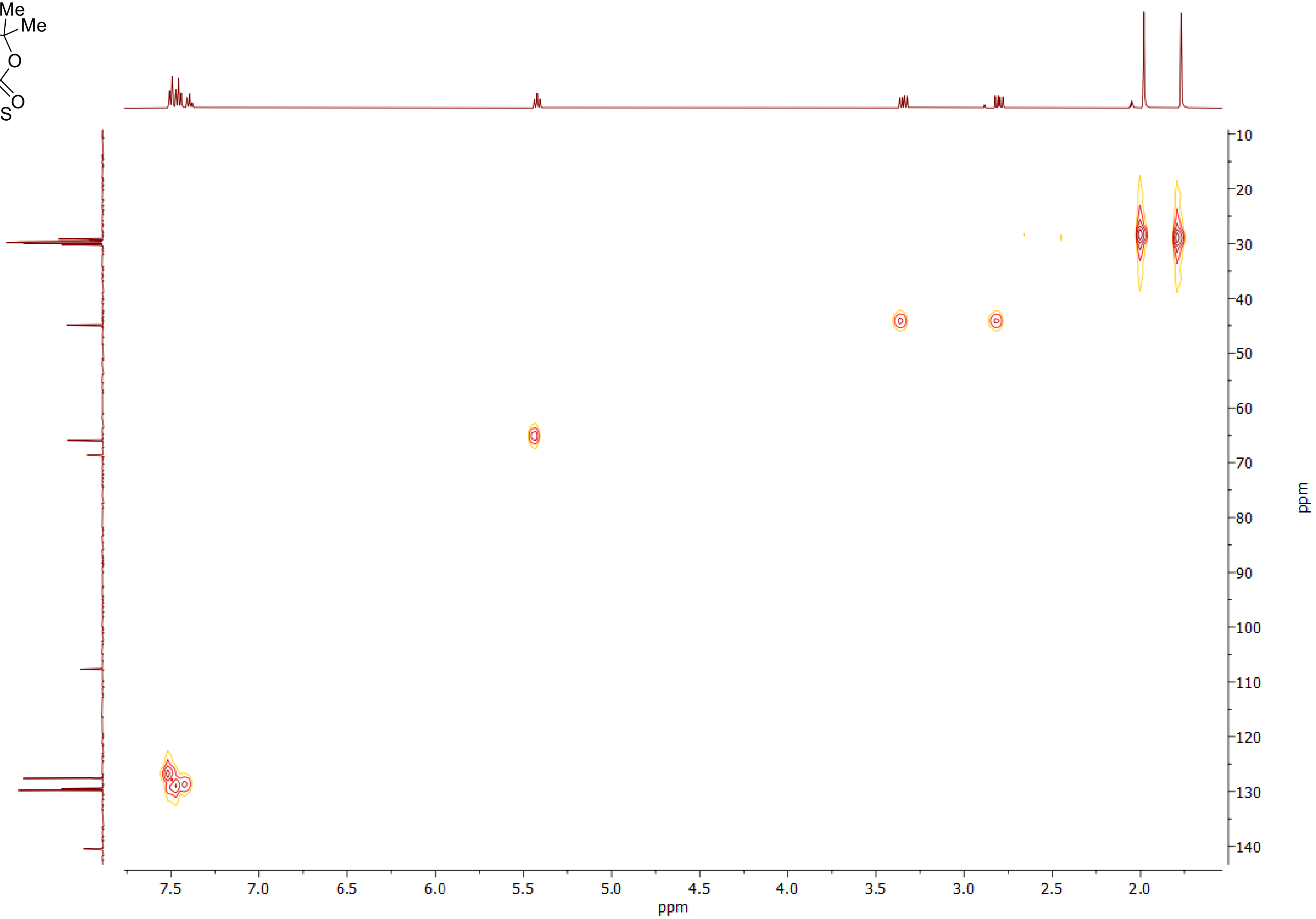

8,8-dimethyl-3-phenyl-1-thioxo-7,9-dioxa-2-azaspiro[4.5]decane-6,10-dione (2ag)

^{1}H–^{13}C HMBC (acetone-d_6)

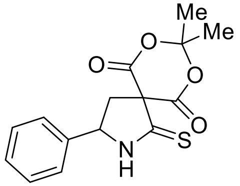

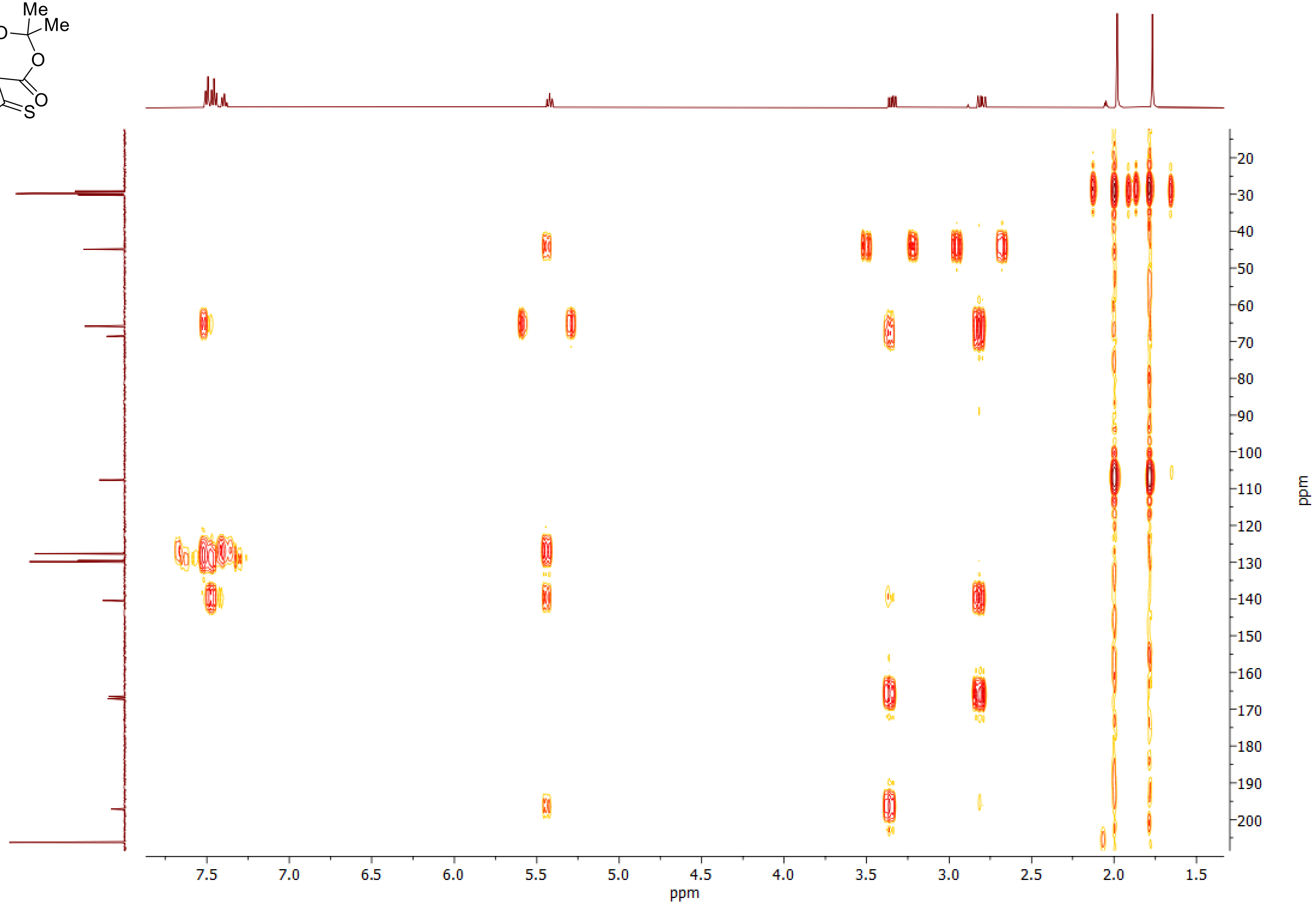

Dimethyl 5-(3,4-dimethoxyphenyl)-2-oxopyrrolidine-3,3-dicarboxylate (5)

^{1}H NMR (500 MHz, $CDCl_3$)

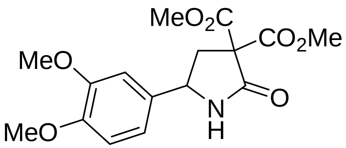

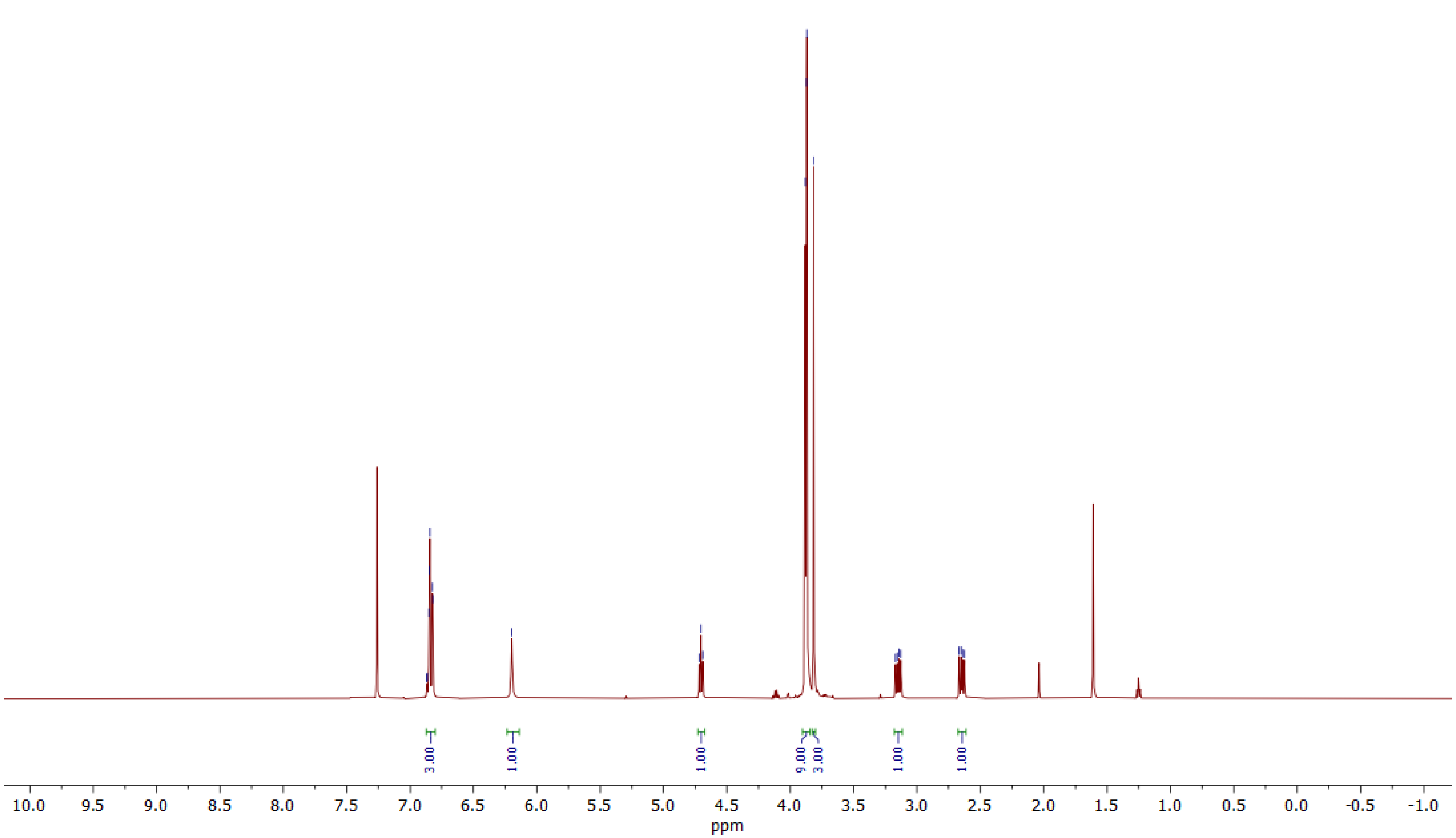

Dimethyl 5-(3,4-dimethoxyphenyl)-2-oxopyrrolidine-3,3-dicarboxylate (5)

^{13}C NMR (126 MHz, $CDCl_3$)

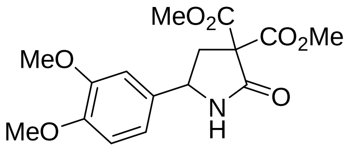

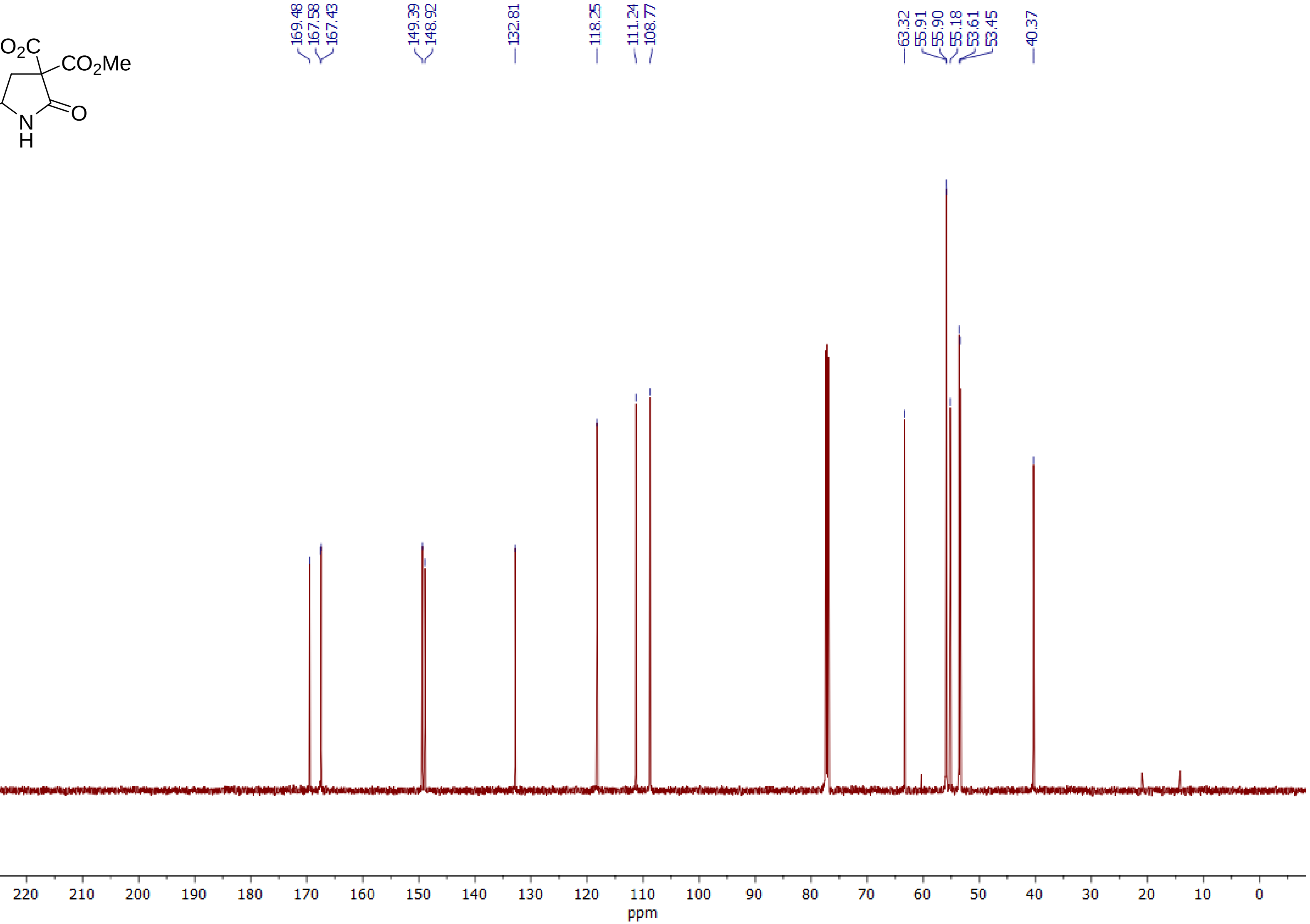

Dimethyl 5-(3,4-dimethoxyphenyl)-2-oxopyrrolidine-3,3-dicarboxylate (5)

^{1}H–^{13}C HSQC ($CDCl_3$)

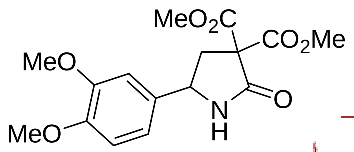

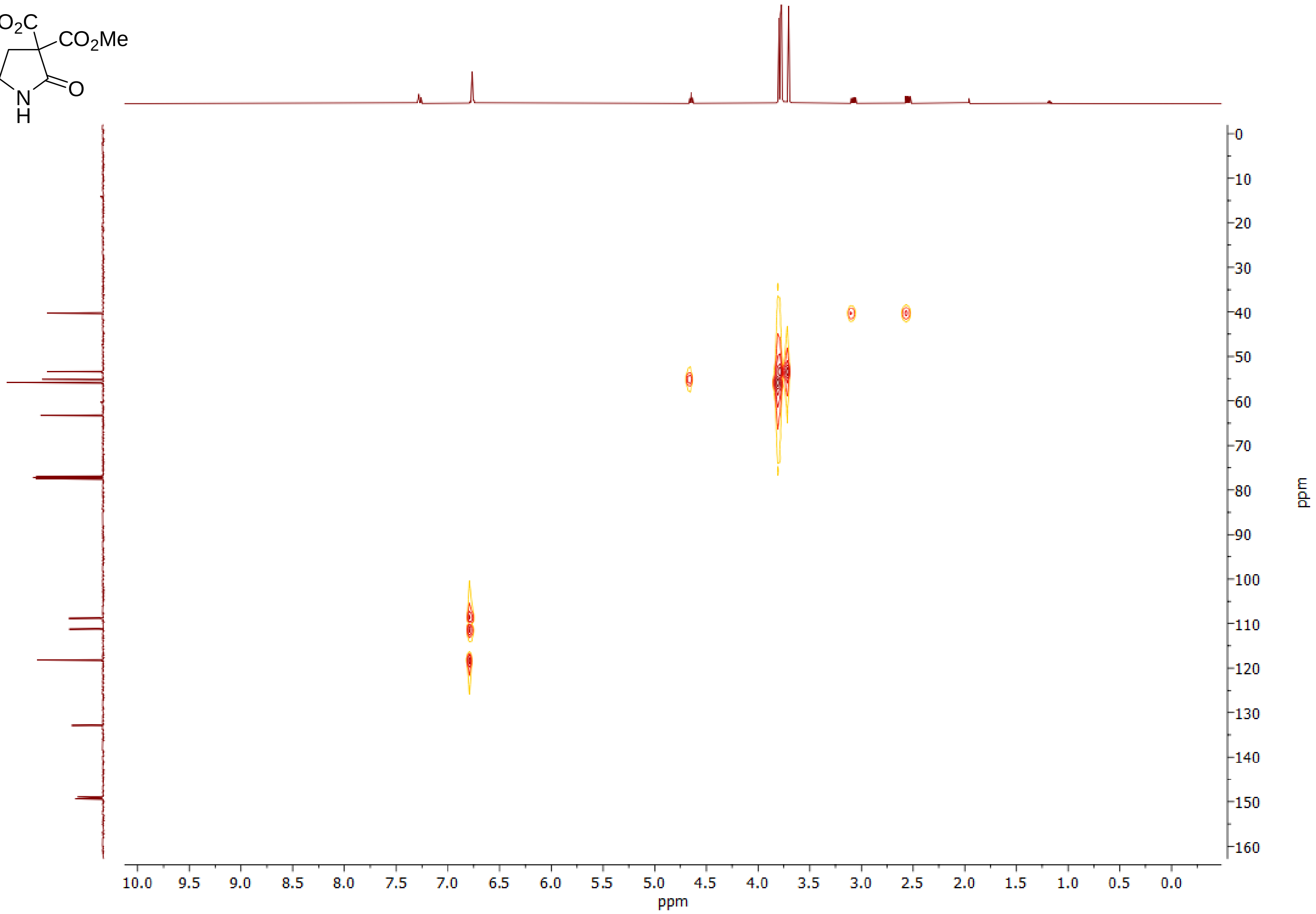

Dimethyl 5-(3,4-dimethoxyphenyl)-2-oxopyrrolidine-3,3-dicarboxylate (5)

^{1}H–^{13}C HMBC ($CDCl_3$)

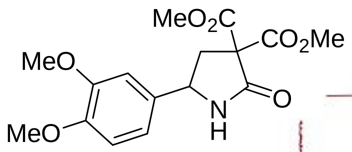

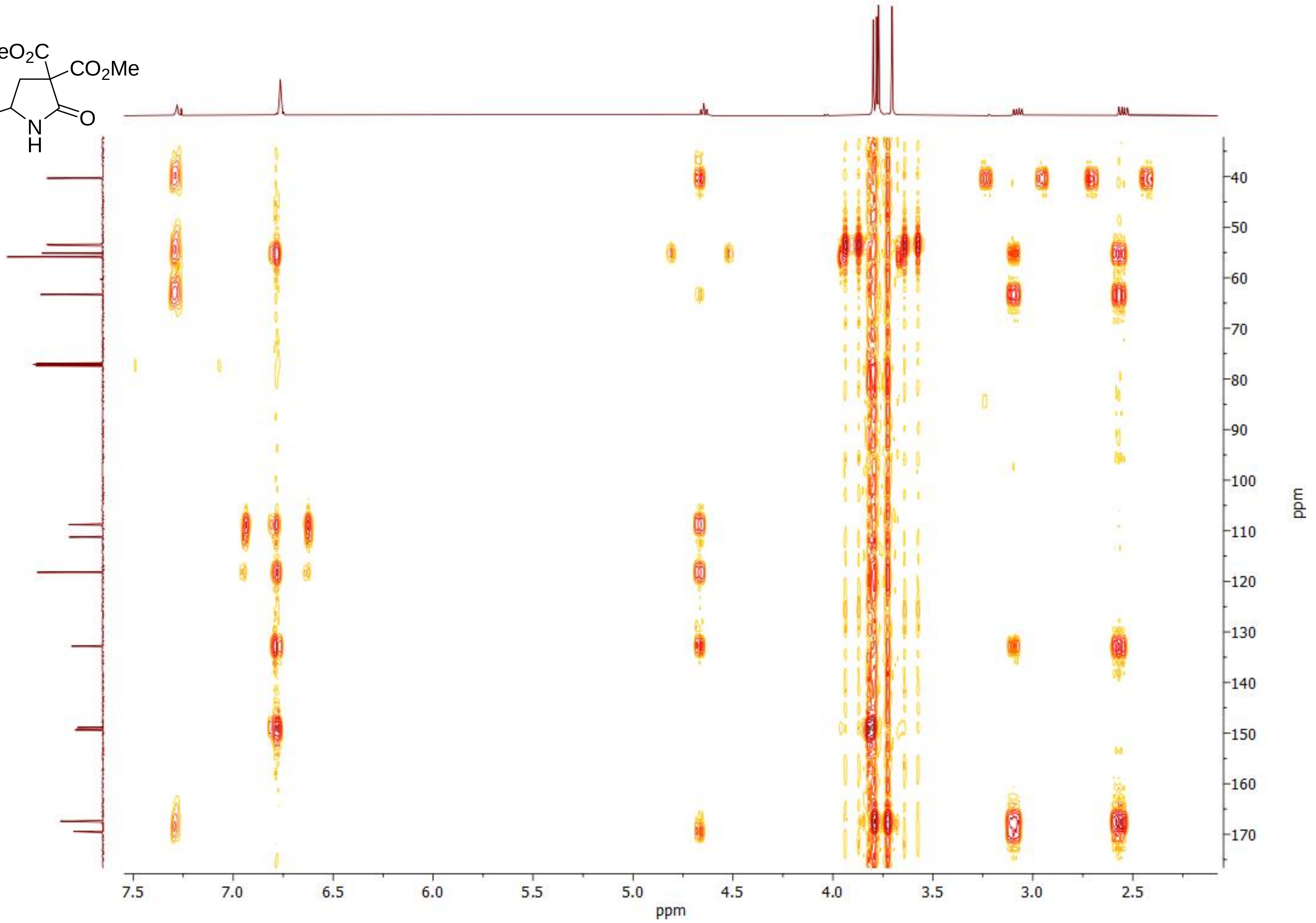

Dimethyl 5-(3,4-dimethoxyphenyl)-2-(methylthio)-4,5-dihydro-3*H*-pyrrole-3,3-dicarboxylate (6)

^{1}H NMR (500 MHz, $CDCl_3$)

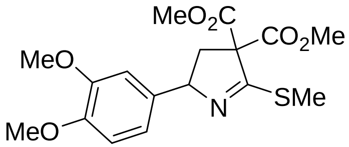

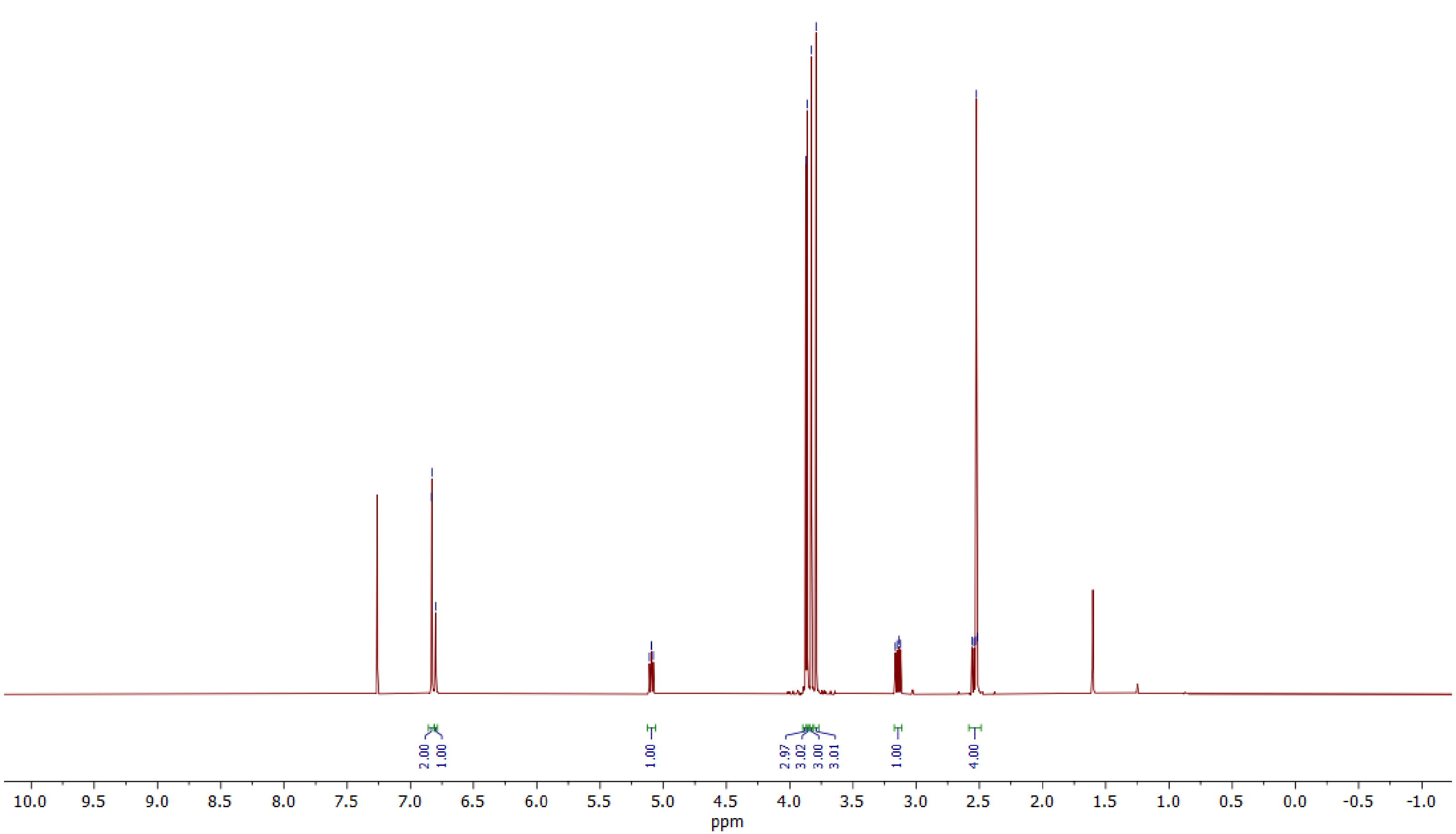

Dimethyl 5-(3,4-dimethoxyphenyl)-2-(methylthio)-4,5-dihydro-3*H*-pyrrole-3,3-dicarboxylate (6)

^{13}C NMR (126 MHz, $CDCl_3$)

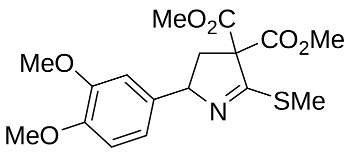

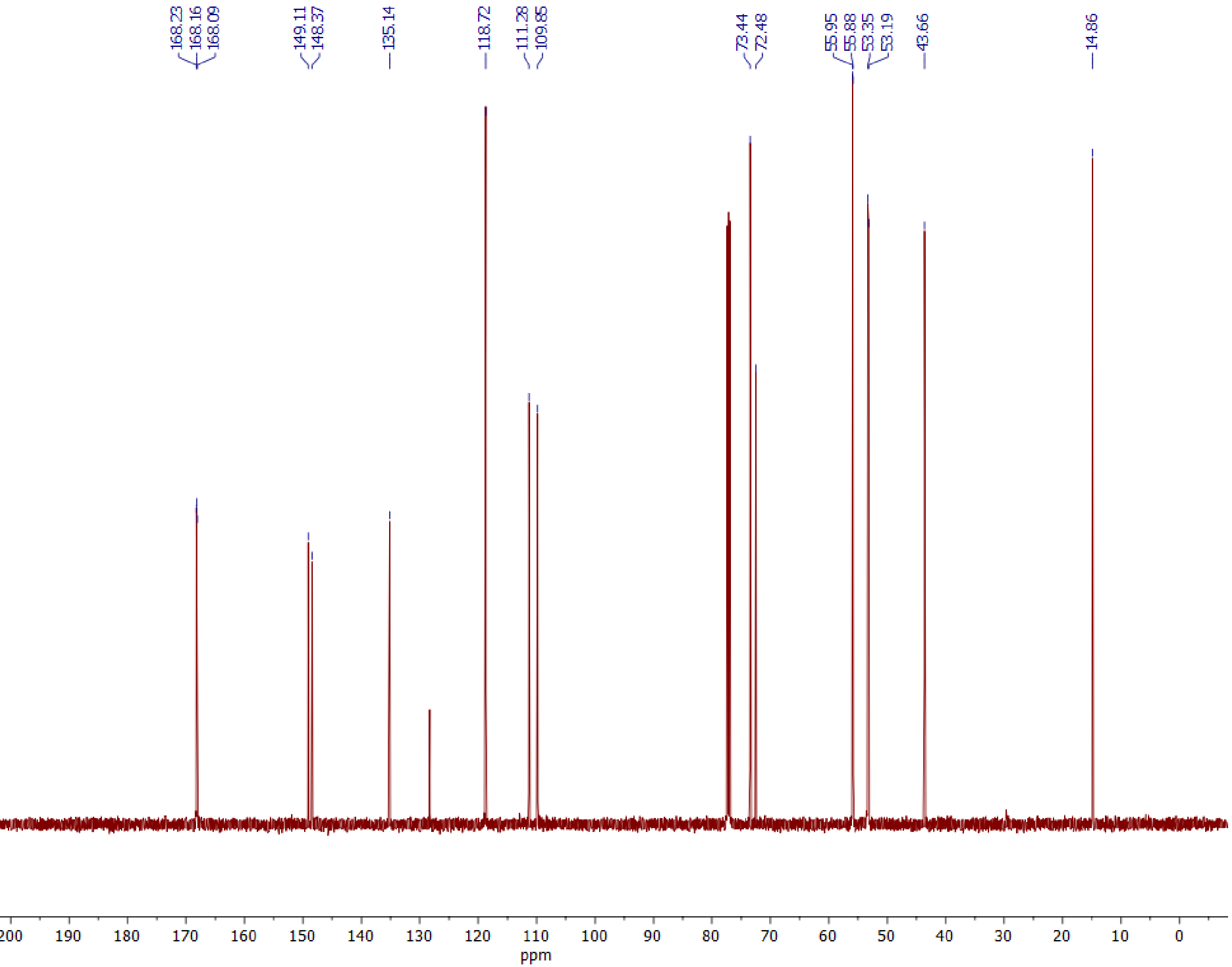

Dimethyl 5-(3,4-dimethoxyphenyl)-2-(methylthio)-4,5-dihydro-3*H*-pyrrole-3,3-dicarboxylate (6)

^{1}H–^{13}C HSQC ($CDCl_3$)

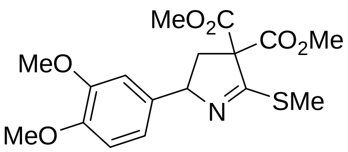

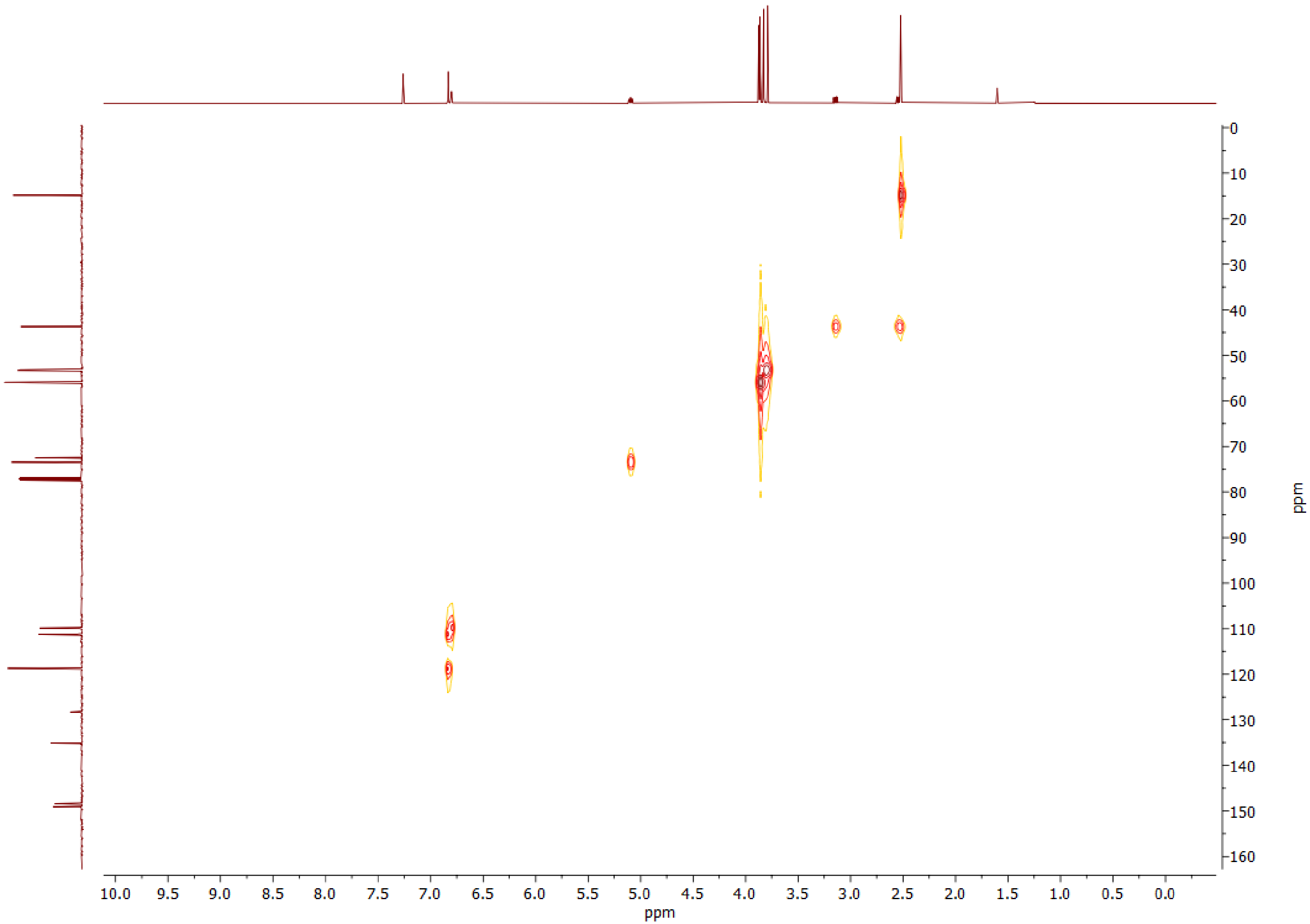

Dimethyl 5-(3,4-dimethoxyphenyl)-2-(methylthio)-4,5-dihydro-3*H*-pyrrole-3,3-dicarboxylate (6)

^{1}H–^{13}C HMBC ($CDCl_3$)

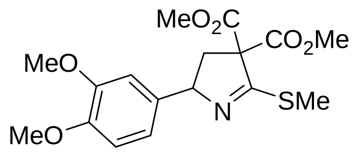

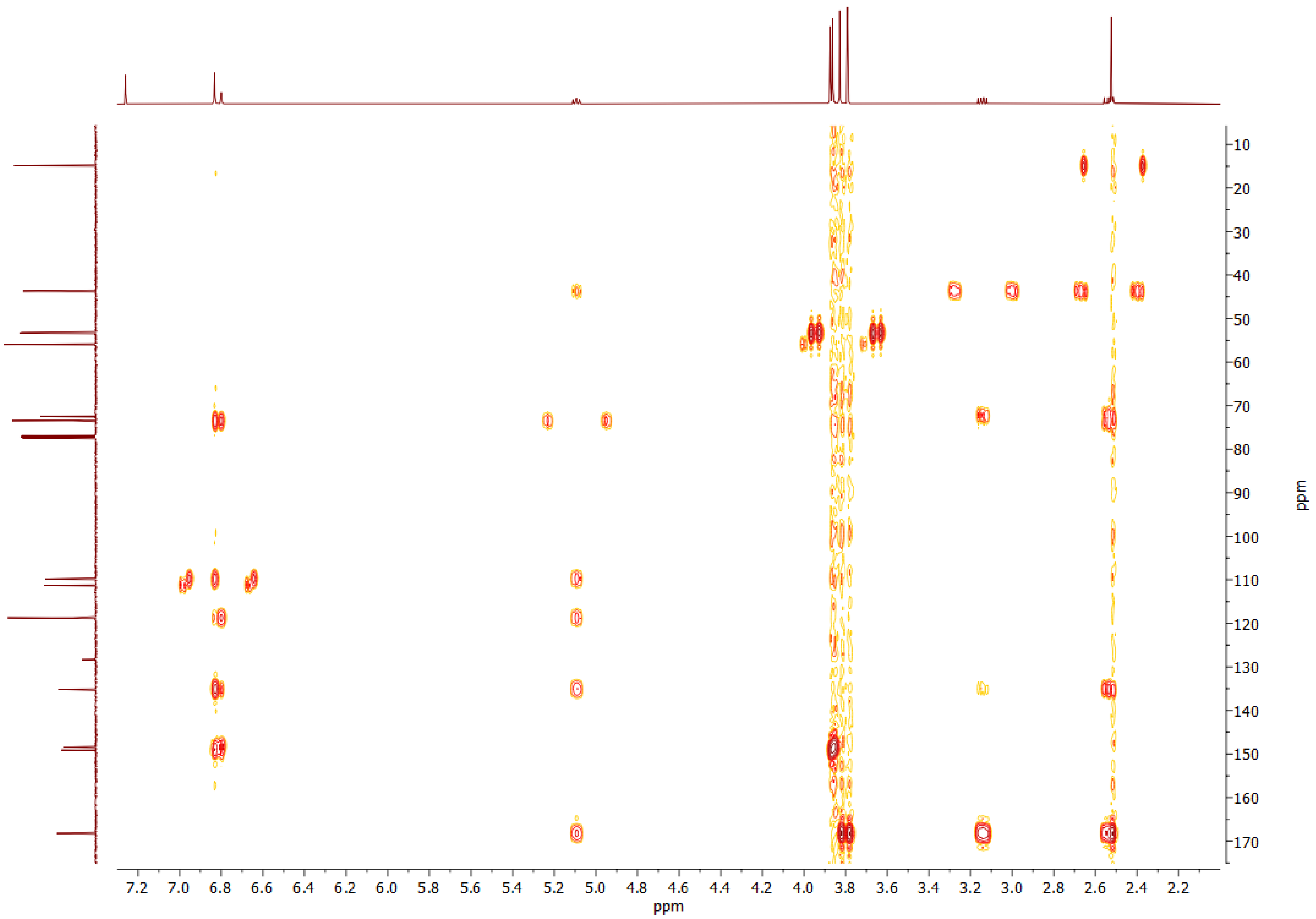

1-Phenyl-3-(2-thioxo-3,4-dihydro-2*H*-benz[*e*][1,3]oxazin-4-yl)propan-1-one (7)

^{1}H NMR (500 MHz, DMSO-d_6)

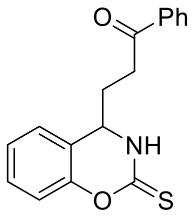

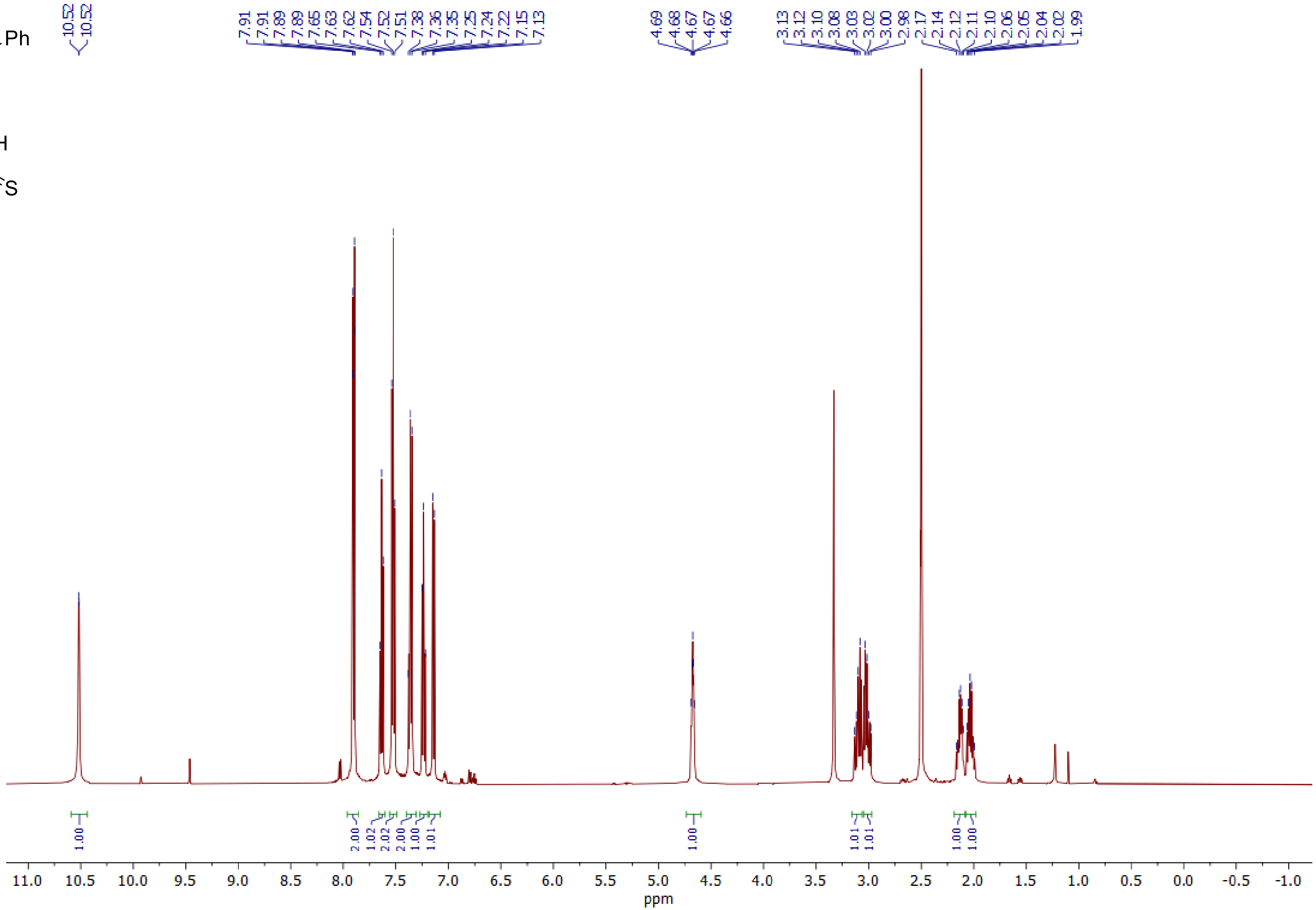

1-Phenyl-3-(2-thioxo-3,4-dihydro-2*H*-benz[*e*][1,3]oxazin-4-yl)propan-1-one (7)

^{13}C NMR (126 MHz, DMSO-d$_6$)

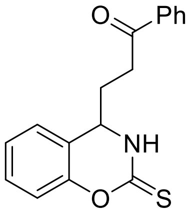

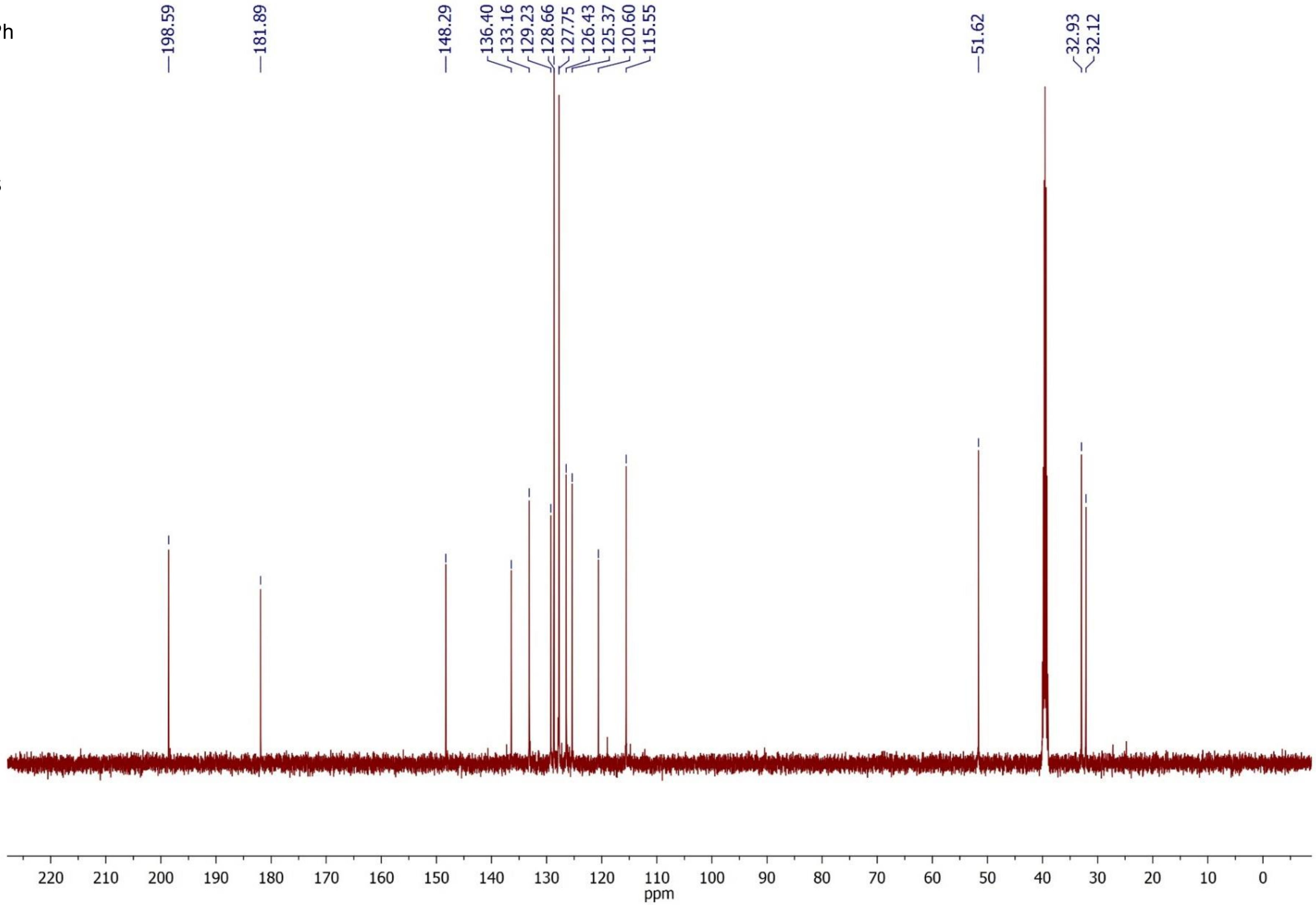

1-Phenyl-3-(2-thioxo-3,4-dihydro-2*H*-benz[*e*][1,3]oxazin-4-yl)propan-1-one (7)

^{1}H–^{13}C HSQC (DMSO-d_6)

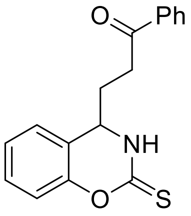

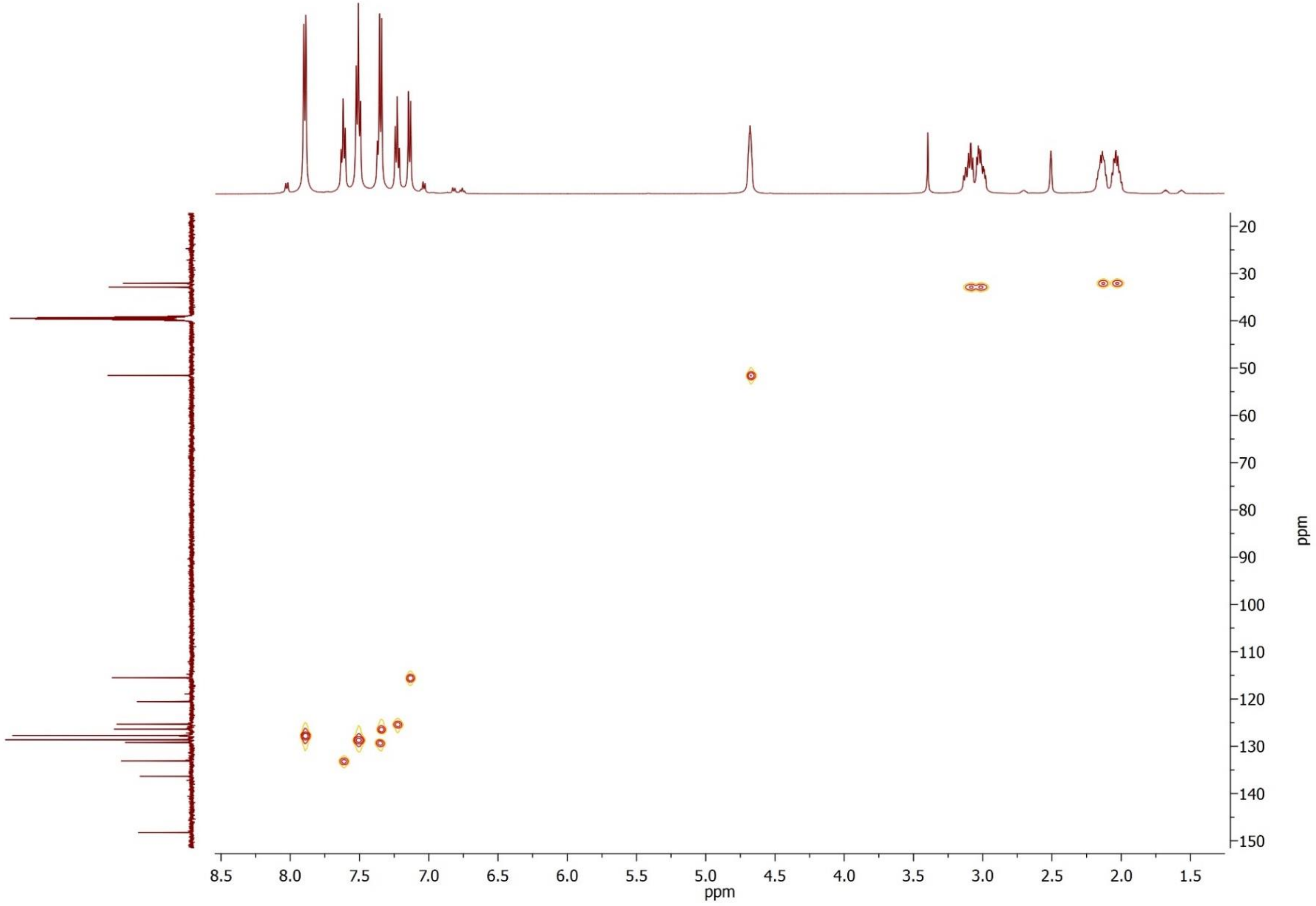

1-Phenyl-3-(2-thioxo-3,4-dihydro-2*H*-benz[*e*][1,3]oxazin-4-yl)propan-1-one (7)

^{1}H–^{13}C HMBC (DMSO-d$_6$)

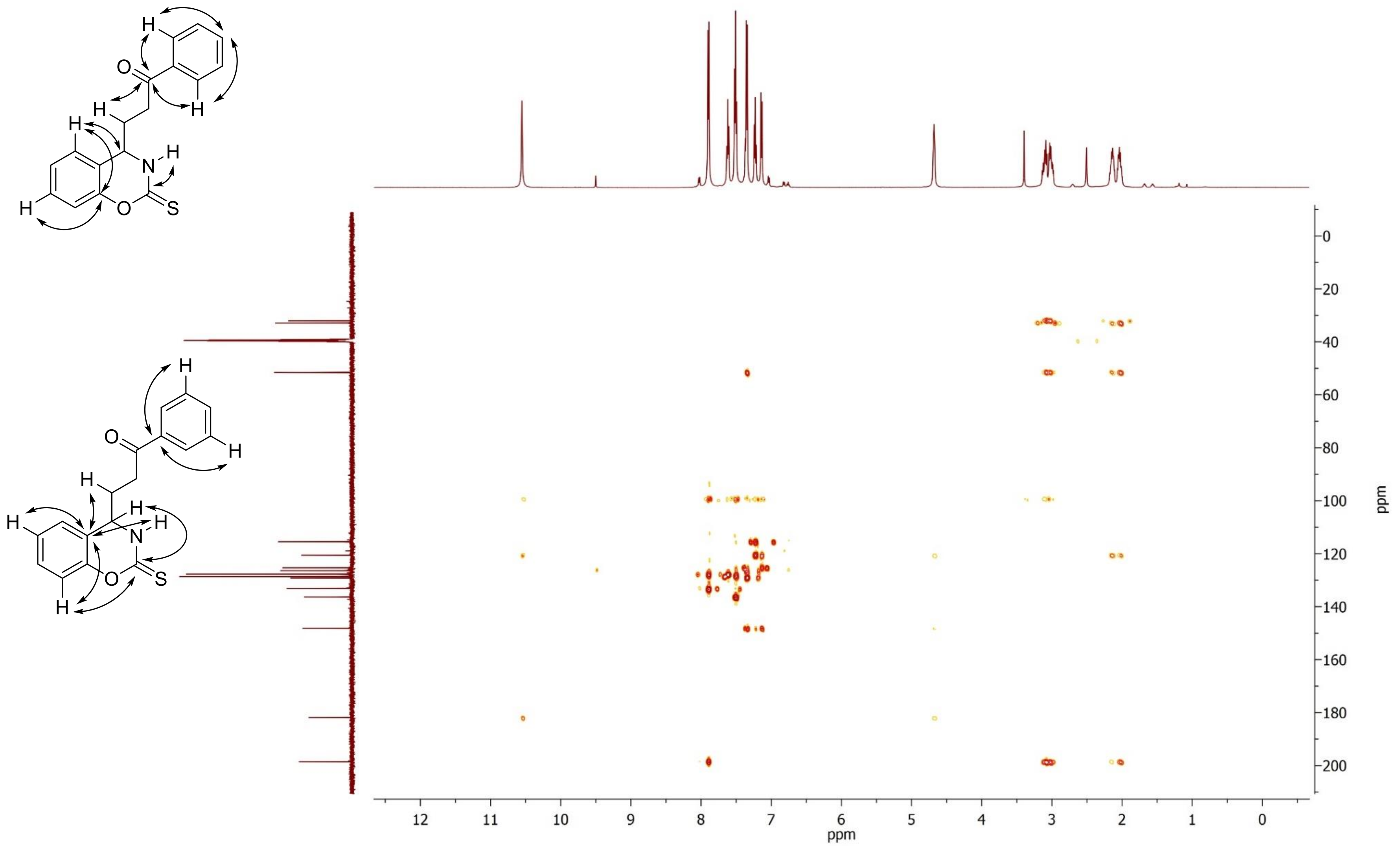

(1*RS*,2*RS*)-2-thiocyanatocyclohexan-1-ol (9)

^{1}H NMR (500 MHz, $CDCl_3$)

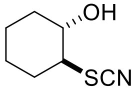

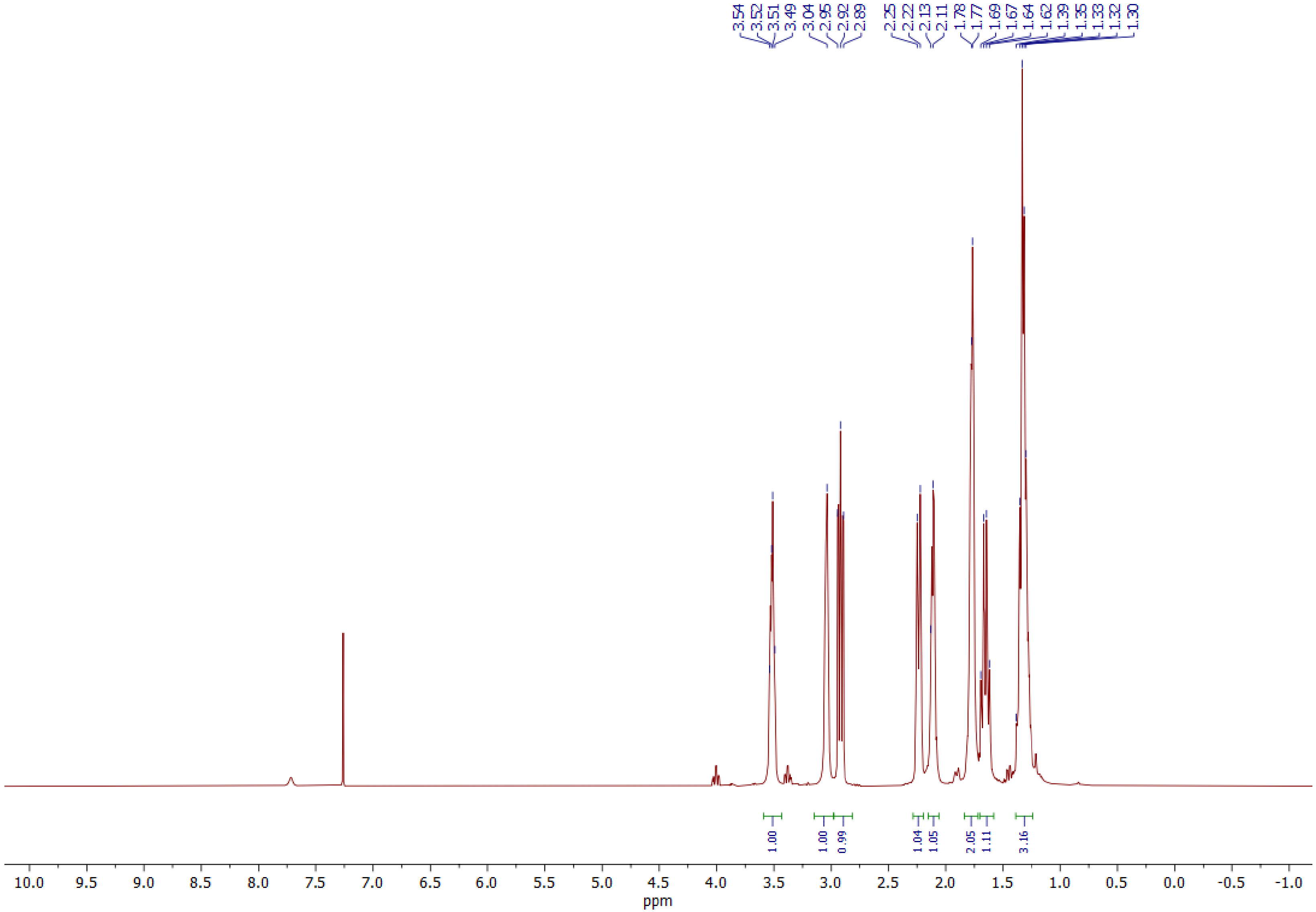

(1*RS*,2*RS*)-2-thiocyanatocyclohexan-1-ol (9)

^{13}C NMR (126 MHz, $CDCl_3$)

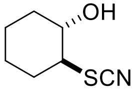

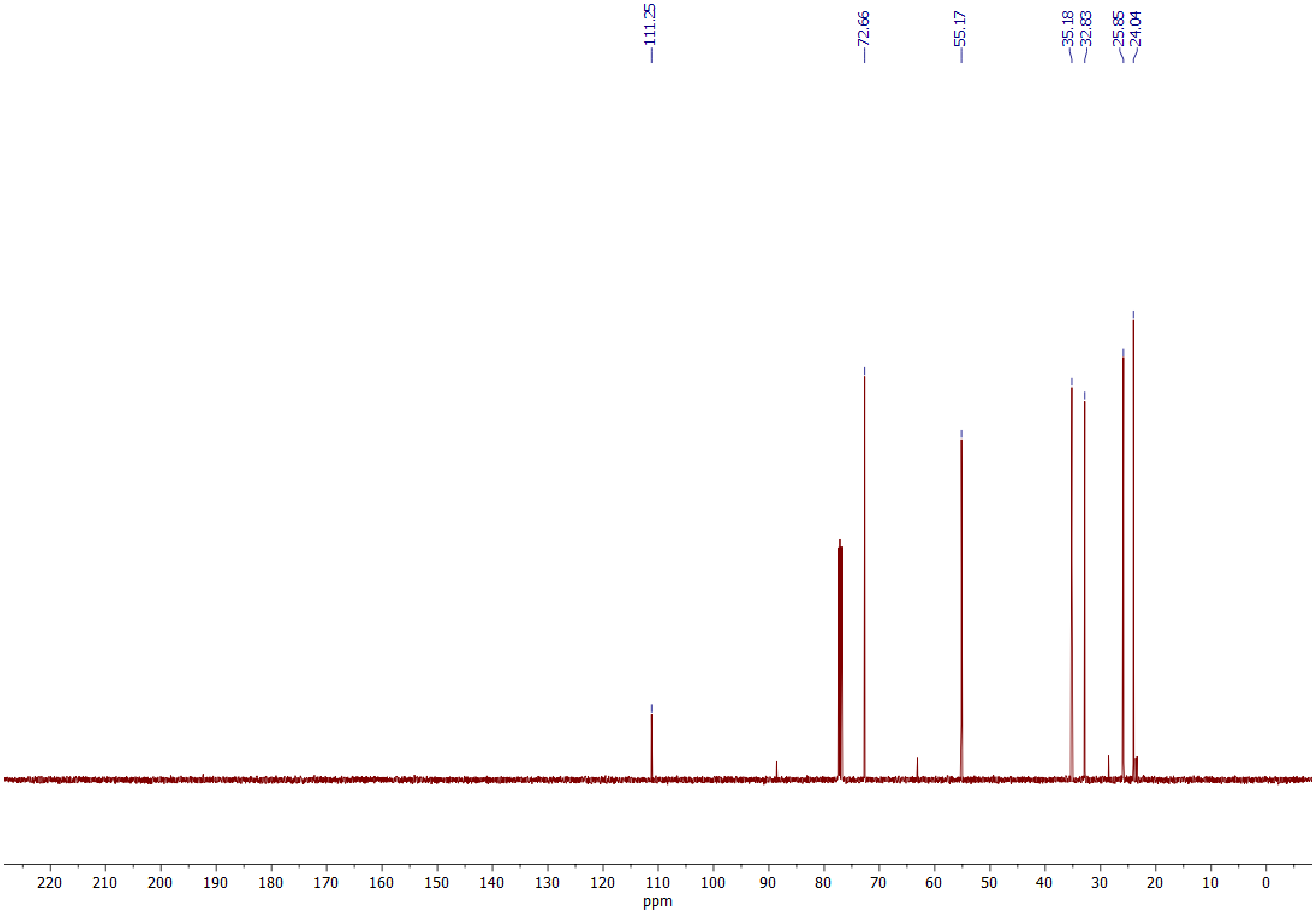

(1*RS*,2*RS*)-2-thiocyanatocyclohexan-1-ol (9)

^{1}H–^{13}C HSQC ($CDCl_3$)

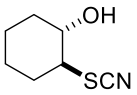

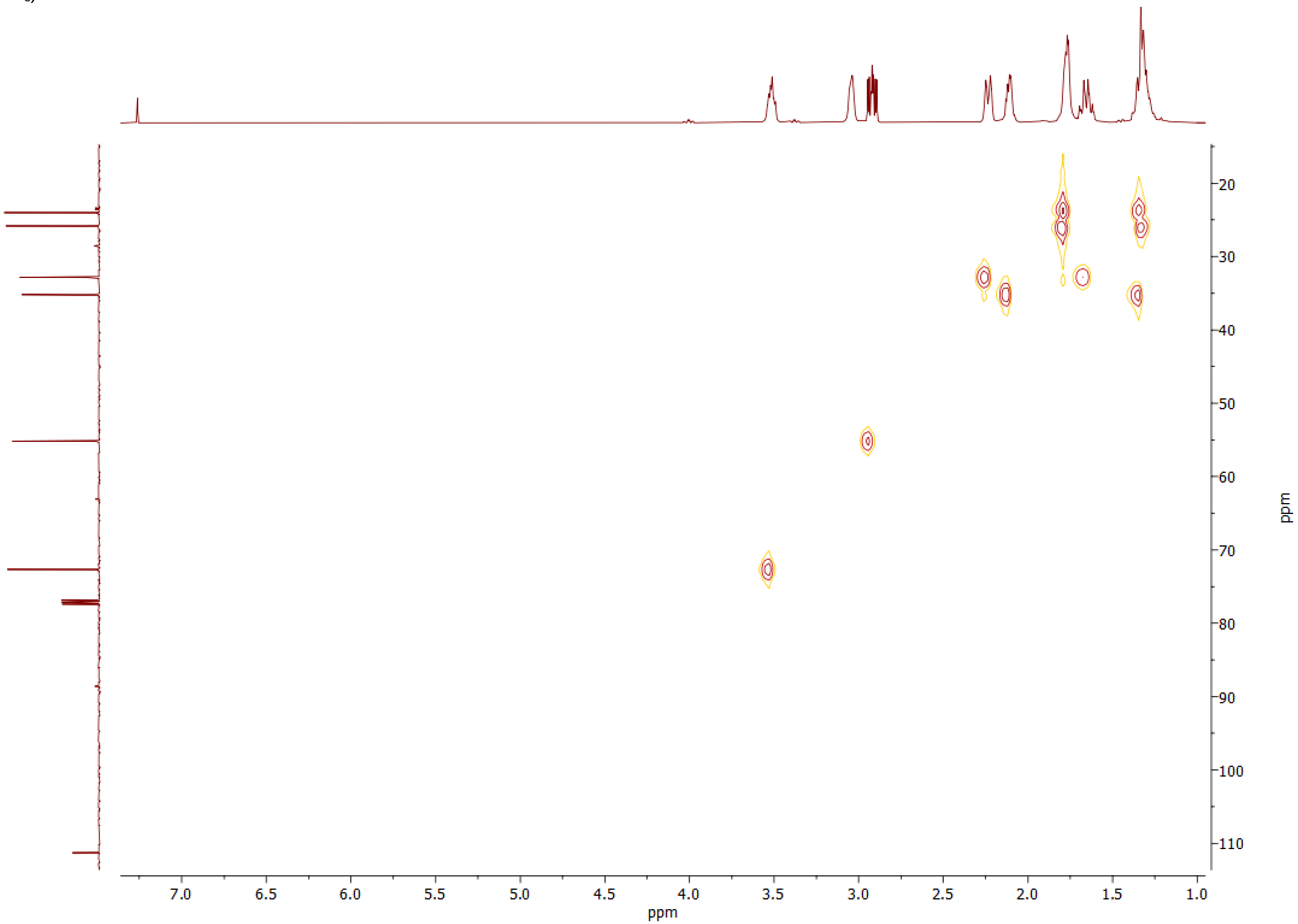

(1*RS*,2*RS*)-2-thiocyanatocyclohexan-1-ol (9)

^{1}H–^{13}C HMBC ($CDCl_3$)

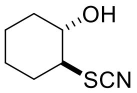

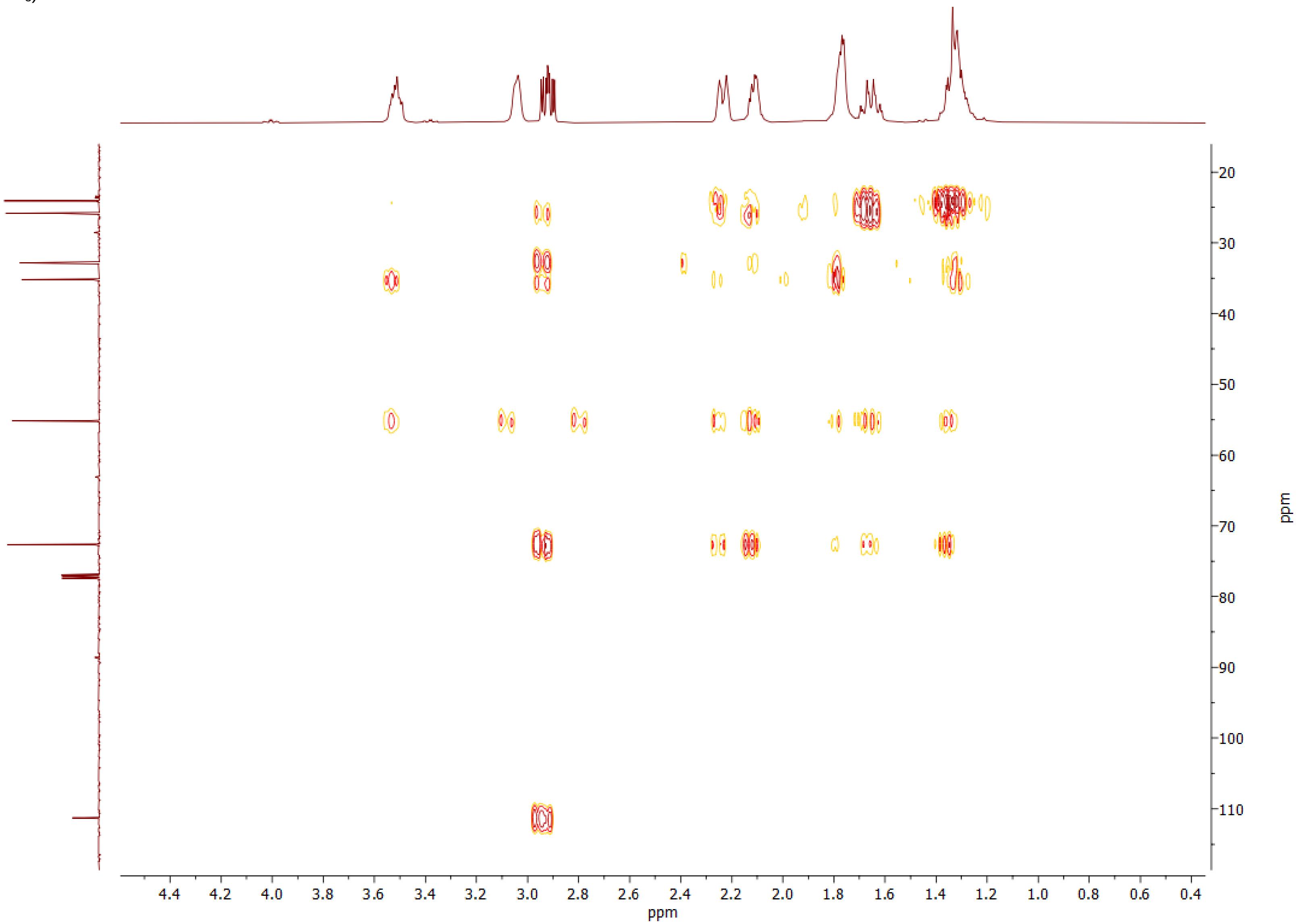

3-(1*H*-imidazol-1-yl)propan-1-ammonium thiocyanate (11)

^{1}H NMR (500 MHz, DMSO-d_6)

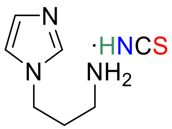

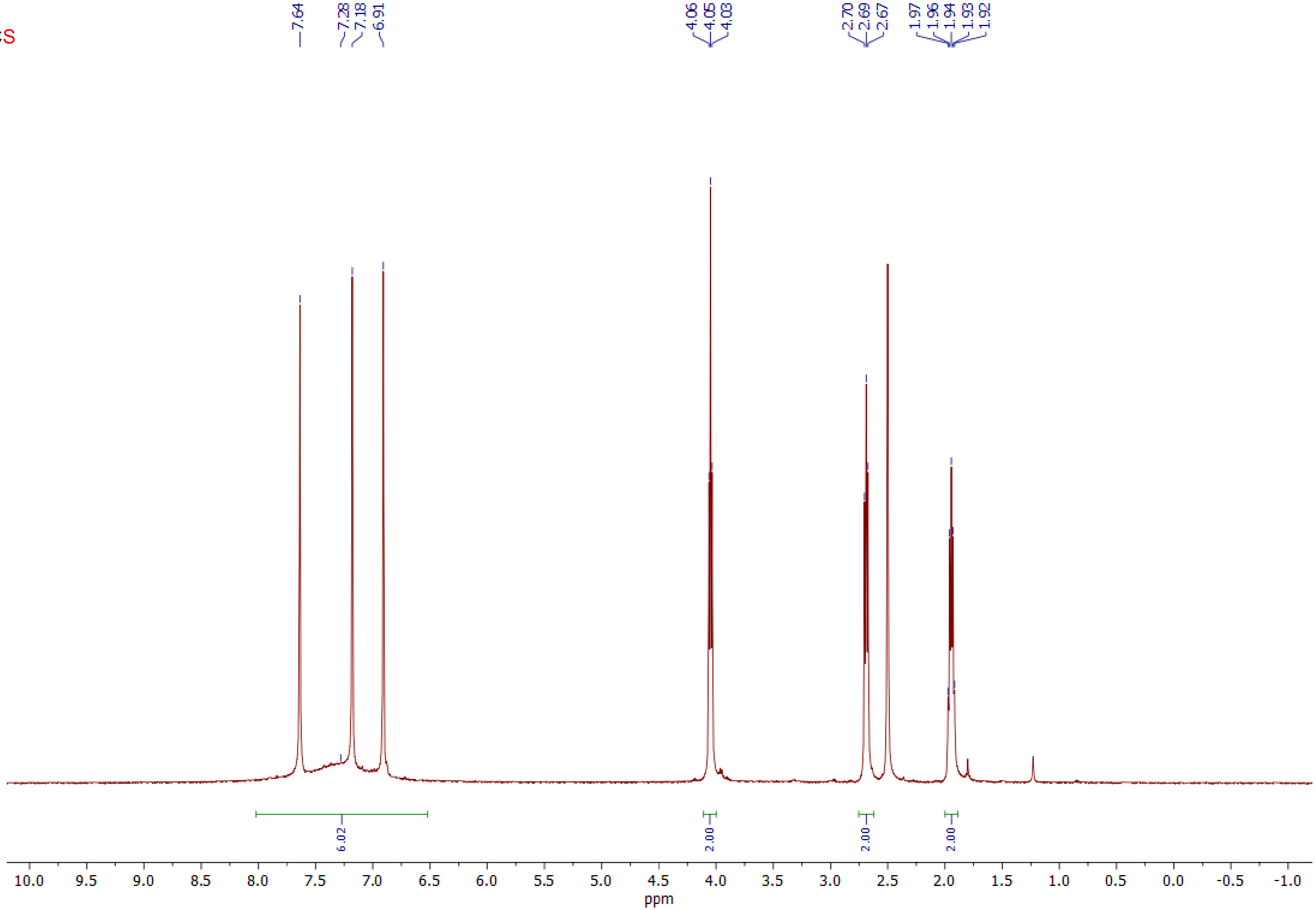

3-(1*H*-imidazol-1-yl)propan-1-ammonium thiocyanate (11)

^{13}C NMR (126 MHz, DMSO-d_6)

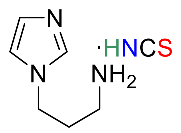

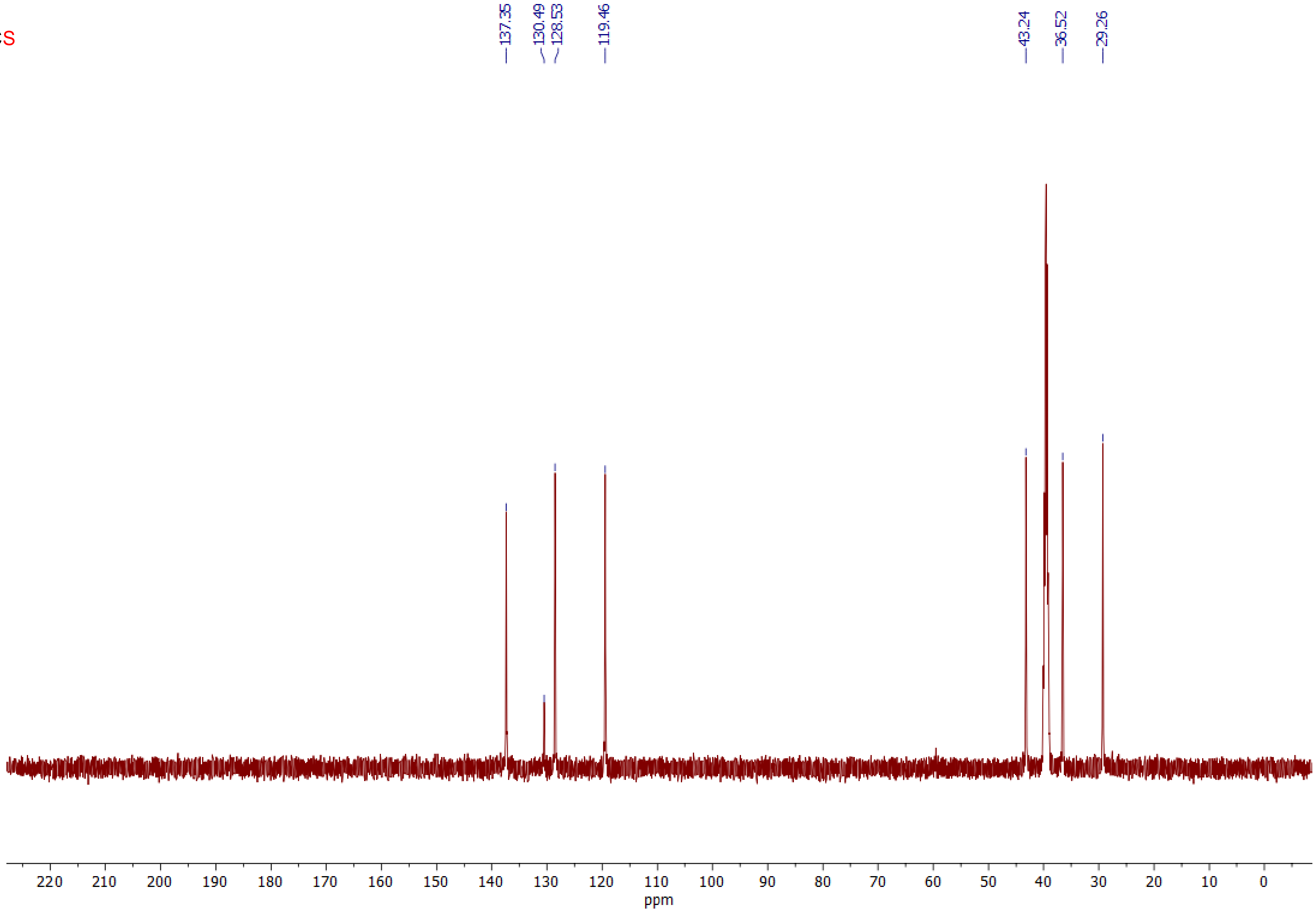

3-(1*H*-imidazol-1-yl)propan-1-ammonium thiocyanate (11)

^{1}H–^{13}C HSQC (DMSO-d_6)

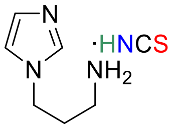

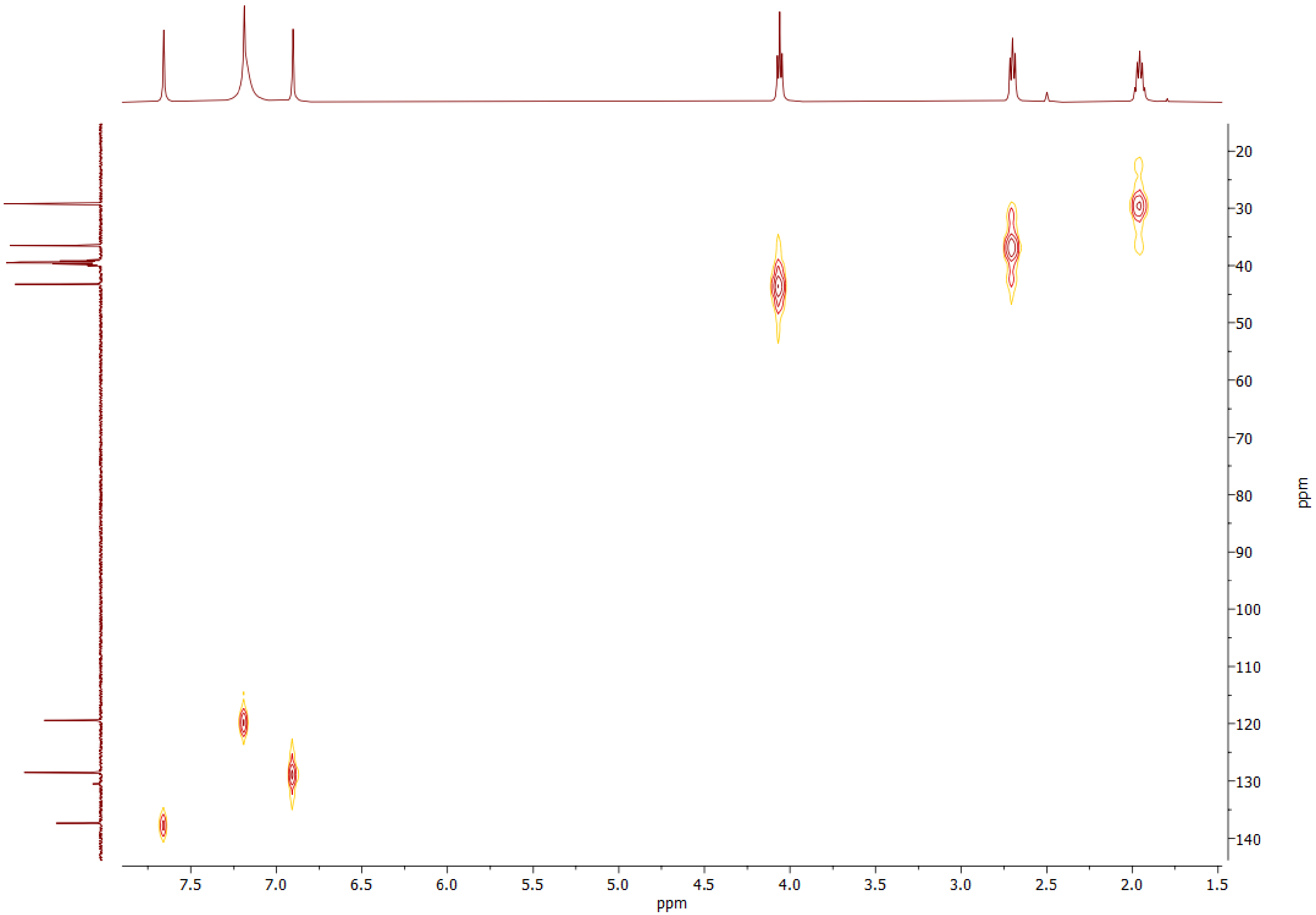

3-(1*H*-imidazol-1-yl)propan-1-ammonium thiocyanate (11)

^{1}H–^{13}C HMBC (DMSO-d_6)

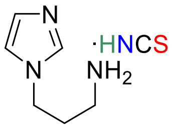

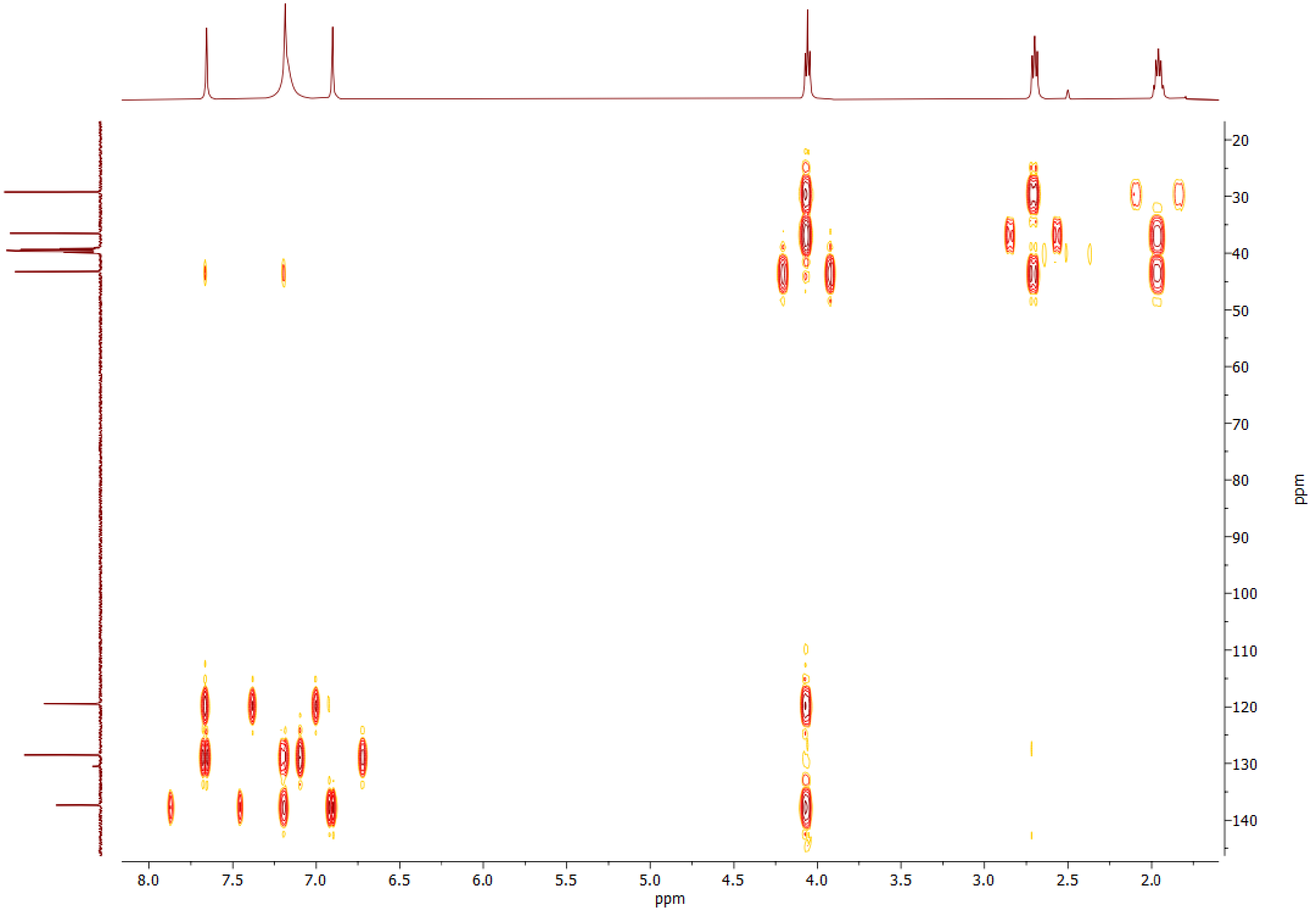

3-Thioxohexahydro-1*H*-pyrrolo[1,2-*c*]imidazol-1-one (13)

^1H NMR (500 MHz, $CDCl_3$)

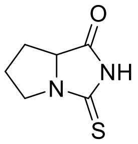

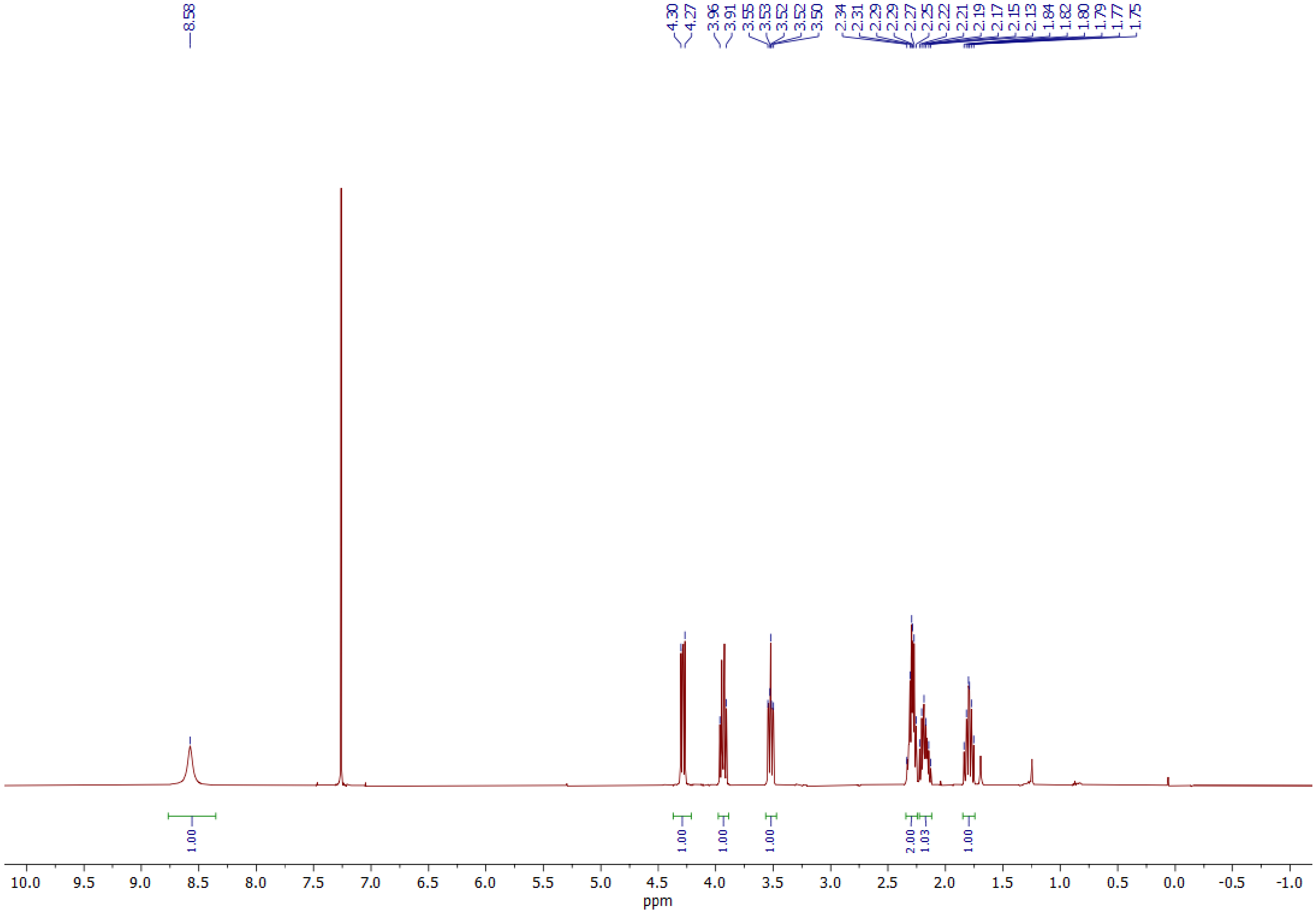

3-Thioxohexahydro-1*H*-pyrrolo[1,2-*c*]imidazol-1-one (13)

^{1}H NMR (500 MHz, acetone-d_6/DMSO-d_6, 10:1)

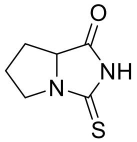

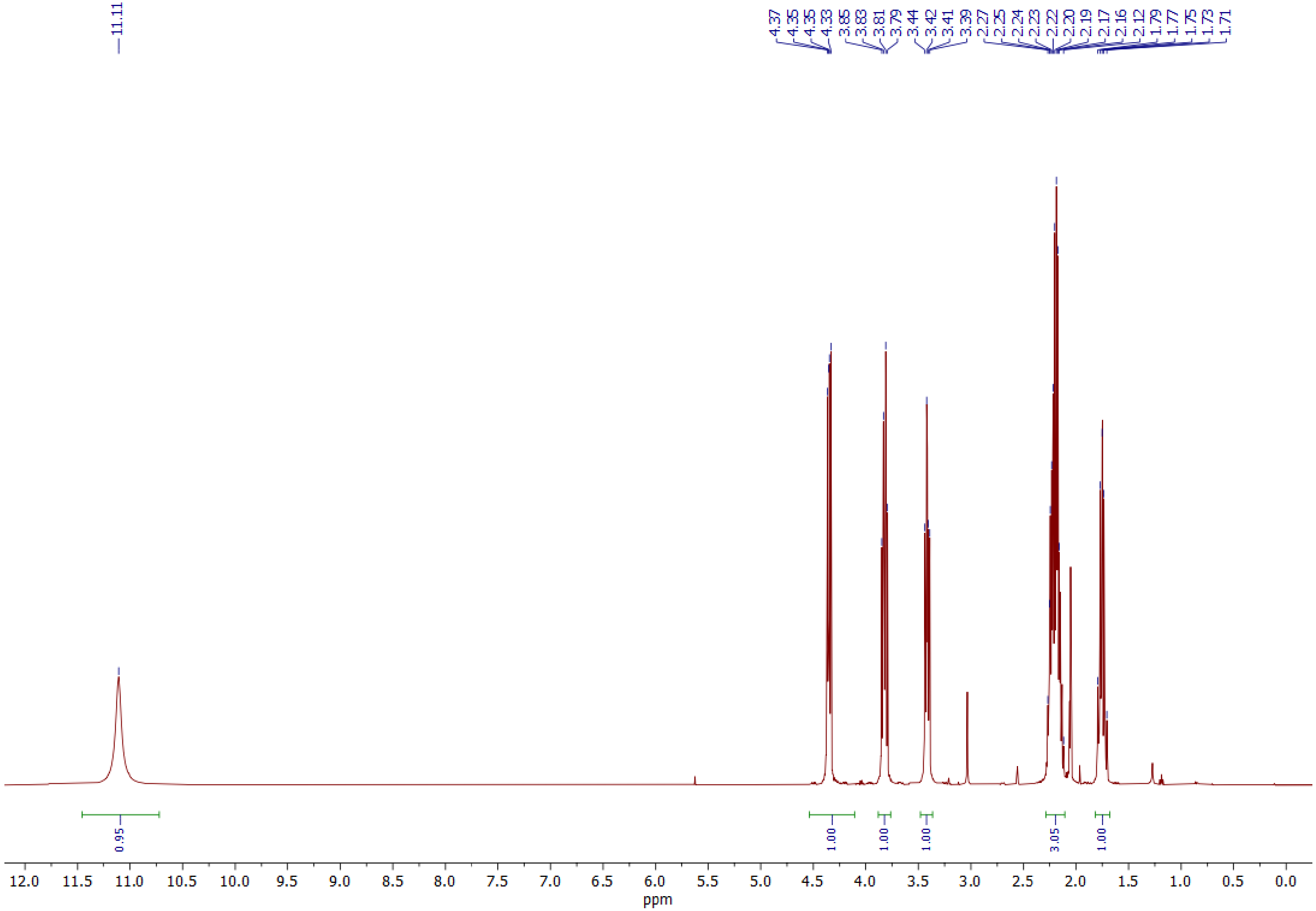

3-Thioxohexahydro-1*H*-pyrrolo[1,2-*c*]imidazol-1-one (13)

^{13}C NMR (126 MHz, acetone-d_6/DMSO-d_6, 10:1)

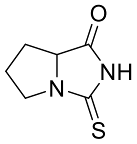

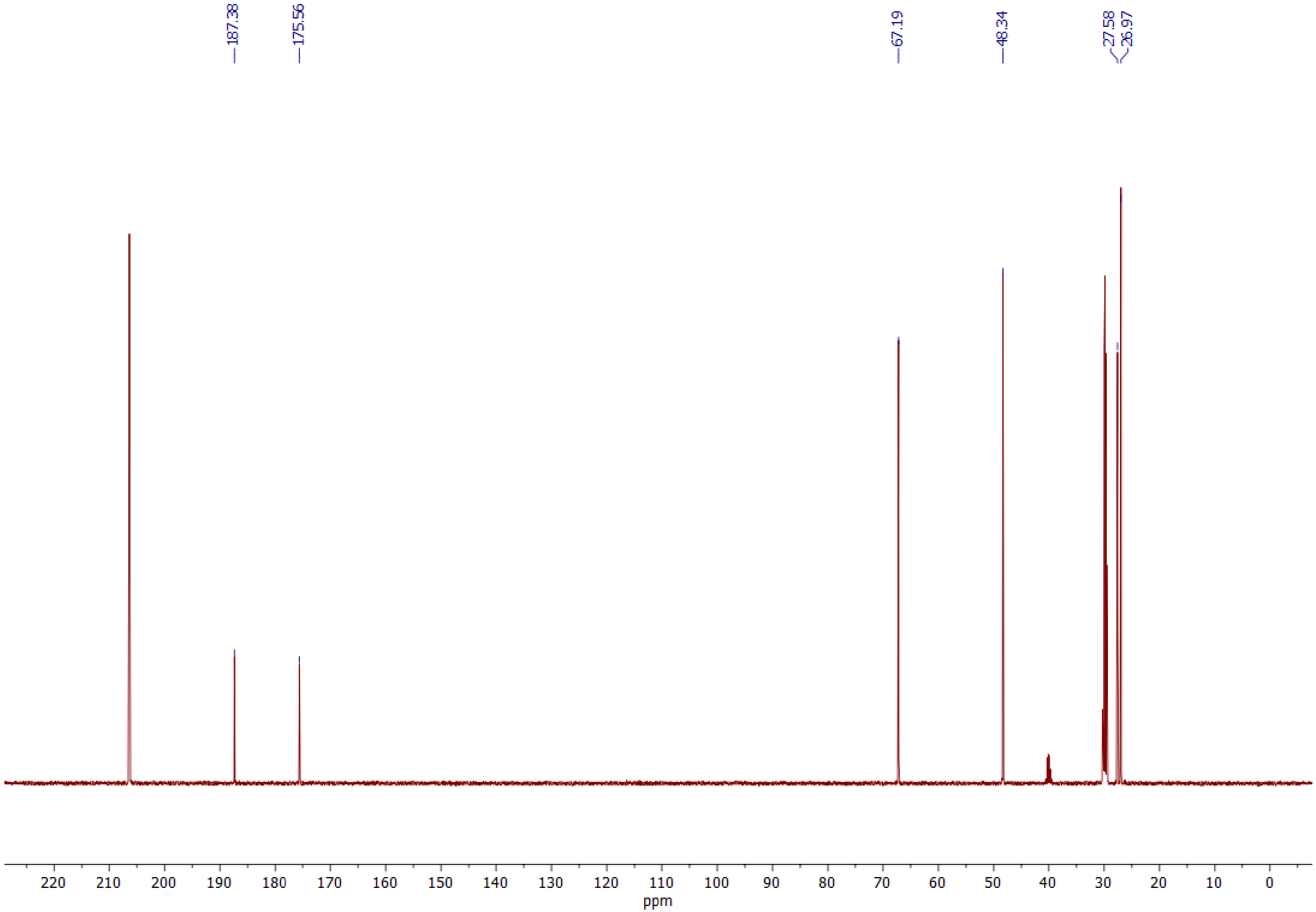

3-Thioxohexahydro-1*H*-pyrrolo[1,2-*c*]imidazol-1-one (13)

^{1}H–^{13}C HSQC (acetone-d_6/DMSO-d_6, 10:1)

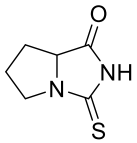

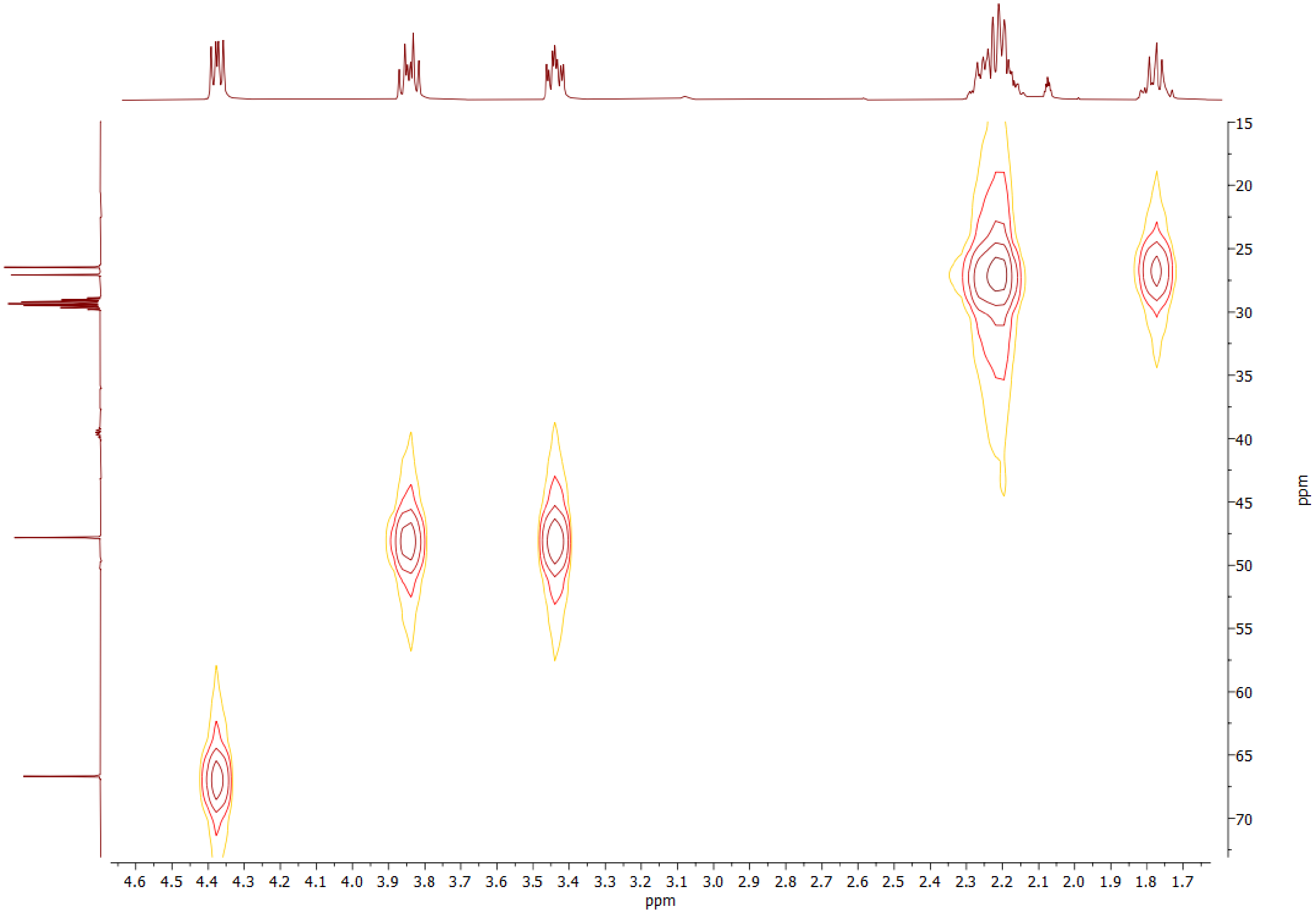

3-Thioxohexahydro-1*H*-pyrrolo[1,2-*c*]imidazol-1-one (13)

^{1}H–^{13}C HMBC (acetone-d_6/DMSO-d_6, 10:1)

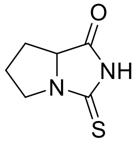

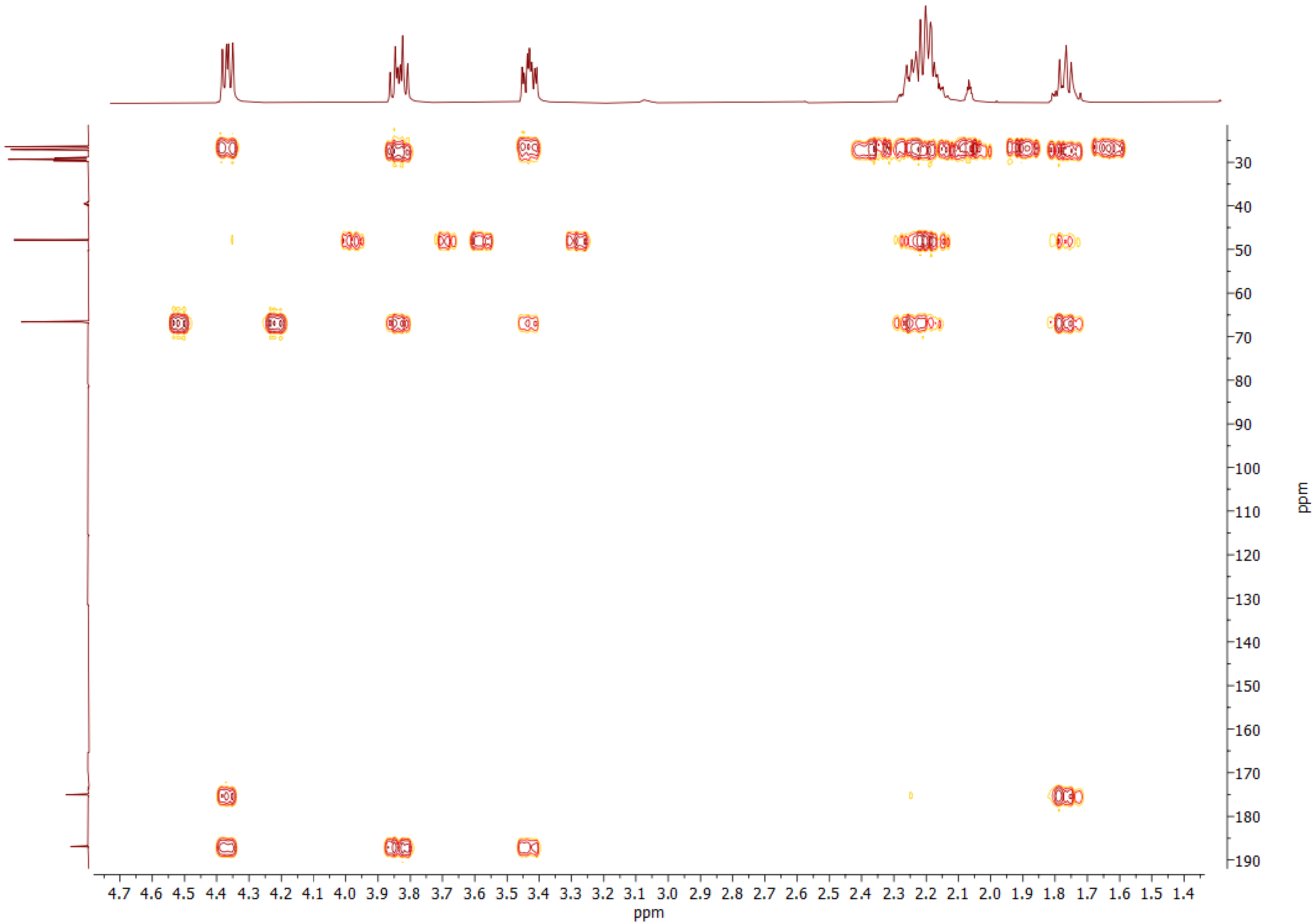

Supplement: Supplementary file 2 — Supplementary [file ANIE-60-7927-s001.pdf]
